# CSF Proteomics of Secondary Phase Spinal Cord Injury in Human Subjects: Perturbed Molecular Pathways Post Injury

**DOI:** 10.1371/journal.pone.0110885

**Published:** 2014-10-28

**Authors:** Mohor Biplab Sengupta, Mahashweta Basu, Sourav Iswarari, Kiran Kumar Mukhopadhyay, Krishna Pada Sardar, Biplab Acharyya, Pradeep K. Mohanty, Debashis Mukhopadhyay

**Affiliations:** 1 Biophysics and Structural Genomics Division, Saha Institute of Nuclear Physics, Kolkata, West Bengal, India; 2 Condensed Matter Physics Division, Saha Institute of Nuclear Physics, Kolkata, West Bengal, India; 3 Department of Physical Medicine & Rehabilitation, Nil Ratan Sircar Medical College & Hospital, Kolkata, West Bengal, India; 4 Department of Orthopaedic Surgery, Nil Ratan Sircar Medical College & Hospital, Kolkata, West Bengal, India; Medical University of South Carolina, United States of America

## Abstract

Recovery of sensory and motor functions following traumatic spinal cord injury (SCI) is dependent on injury severity. Here we identified 49 proteins from cerebrospinal fluid (CSF) of SCI patients, eight of which were differentially abundant among two severity groups of SCI. It was observed that the abundance profiles of these proteins change over a time period of days to months post SCI. Statistical analysis revealed that these proteins take part in several molecular pathways including DNA repair, protein phosphorylation, tRNA transcription, iron transport, mRNA metabolism, immune response and lipid and ATP catabolism. These pathways reflect a set of mechanisms that the system may adopt to cope up with the assault depending on the injury severity, thus leading to observed physiological responses. Apart from putting forward a picture of the molecular scenario at the injury site in a human study, this finding further delineates consequent pathways and molecules that may be altered by external intervention to restrict neural degeneration.

## Introduction

Spinal cord injury (SCI) is one of the leading causes of disability and morbidity worldwide [1] although epidemiological studies are limited in India [2]. In the present study we included a cohort of East Indian population.

SCI due to trauma has two stages: the primary and the secondary injuries [3]. As the acute primary phase is over by seconds to minutes, the secondary injury gives a valuable time window to explore events before interventions are done for stabilizing the patient. Although there are a few well established pathways of secondary injury, most of these are not readily known or accessible for clinical practice.

Right after the initial mechanical damage inflicted by the primary injury, a plethora of molecular changes set in, initiating the secondary injury processes [4]. Various processes like hypoperfusion in the grey matter, glutamate excitotoxicity, plasma membrane failure, ionic perturbation, energy failure, ATP catabolism, inflammatory pathways, demyelination, apoptosis, cell and tissue damage and lipid peroxidation [5] become predominant. Although some of these mechanisms overlap with acute primary injury, myelin associated inhibitory factors (MAIF) [6, 7, and 8] and glial scar formation [9], are known to act in conjunction and limit axonal growth severely, leading to collapse of growth cones.

The processes mentioned above vary in extent depending on the injury severity and hence it is imperative to study human CSF of spinal cord injured patients during secondary phase. Proteins thus found can help speculating on various molecular pathways and their perturbation at the backdrop of neuronal injury. Severity dependent biomarker studies based on American Spinal Injury Association (ASIA) Impairment Scale (AIS) classification [10] have been conducted in human CSF samples [11] where it has been shown that inflammatory cytokine levels are elevated in AIS grade A (complete) injury and an inflammatory profile of CSF from cervical SCI rats [12] has revealed MMP-8 as a biomarker. Other studies have also documented several biomarkers in SCI of rodents [13].

The objective of the present study is to survey the intracellular molecular pathways that are perturbed in severe SCI during the secondary phase. Towards this, we compared CSF from AIS A (complete injury) and AIS C or D (incomplete injury) patients at an early time period after injury to identify proteins having differential abundance among the two severity groups. This is because regeneration outcomes vary widely among the two groups. We further compared their differential abundance at a later time-period post injury as it is presumed that CSF undergoes substantial molecular alteration as the secondary phase progresses. Additionally, a protein-protein interaction network (PPIN) was constructed taking proteins identified from CSF and their interactors as nodes. The network analysis revealed a number of vulnerable molecular pathways which may be regarded as soft targets for further exploration in severe SCI.

## Materials and Methods

### Ethics Statement

The study was conducted as a collaboration of SINP and NRSMC&H, Kolkata, India, after it was approved by ‘Institutional Ethical Committee, NRS Medical College, Kolkata’ and ‘Institutional Ethics Committee, SINP, Kolkata’. An informed written consent was obtained from the subjects as per Helsinki Declaration, 2013.

### Patient selection and scoring

The study was conducted in two patient groups with CSF drawn at two time periods (1–8 days and 15–60 days) post injury respectively. Patients with traumatic spinal cord injury in the secondary phase admitted in the spinal injury ward under the Dept. of Orthopaedic Surgery were enrolled in the two study groups (Table 1), after screening by an orthopaedist and a physiatrist from Dept. of Orthopaedic Surgery and Dept. of Physical Medicine & Rehabilitation respectively. Patients were evaluated according to the International Standards for Neurological Classification of SCI (ISNC SCI). Patients who conformed to the set inclusion and exclusion criteria were selected for study (Table 2). Any patient with factors that have the possibility to alter the regenerative and degenerative process in the injured area as mentioned in Table 2 was excluded from the study. First we determined if it was a complete injury with loss of anal sensation and contraction. Then tests for sensory perception (pin prick and feather touch) and motor activity for upper and lower limbs were done to ascertain the ASIA grade of injury. Motor and sensory levels were determined and scored clinically. Clinical level was matched with radiological level determined with non contrast MRI and X-rays. MRI showing oedema in the cord was considered for complete injury. In incomplete injuries of lower grade where MRI did not show oedema, we considered X-ray for determining radiological level. In case of different sensory and motor levels on clinical examination we fixed the highest clinical level that matched with MRI or X-ray. It was this spinal segment we considered for collecting CSF.

**Table 1:** Study details.

**Type of study**	Cohort study
**Period of study**	December 2011-July 2014
**No of participants**	45
**Total sample drawn**	45 ml
**Included samples**	20

**Table 2:** Patient inclusion and exclusion criteria.

No	Inclusion criteria
1	Patient with SCI due to fall or crush with AIS-A, C and D grade injuries
2	Patient should be at 24 hrs to eight days post injury for the first study group
3	Patient should be at 15–60 days post injury for the second study group
	**Exclusion criteria**
1	Patient in spinal shock stage
2	Other neurodegenerative diseases
3	SCI with lacerated cord or due to electrical injury
4	Associated poly trauma
5	Prior surgical stabilization of spine
6	Infectious diseases
7	Metabolic disorders
8	Patients on molecules that may inhibit Rho-ROCK pathways

Before collection of CSF a complete hemogram, ESR, CRP, serum fasting sugar, post prandial sugar, electrolyte, calcium, urea, creatinine, total protein, albumin and globulin were done along with a lipid profile, liver function test and thyroid profile. A routine urine examination with ultrasound check of lower abdomen was done to assess the bladder and to look out for hidden injury because infection, metabolic disorder, electrolyte imbalance and bladder abnormality could have potential effect on the environment of injured spine under study.

### CSF collection and processing for proteomics experiments

All the vital parameters were checked. The patient's heart rate and ECG recording were noted. Patient's blood pressure, oxygen saturation and signs of postural hypotension were noted in a sterile operation theatre with all resuscitation equipment. This is considered vital because hemodynamic alteration can affect the internal environment of the injured site under study.

CSF was drawn by thecal puncture with 23 G spinal needle (Spinocaine 23/G) to minimize injury. Adequate flow of CSF on spinal tapping suggested normal flow of CSF through the central nervous system bathing the spinal cord, thereby giving us CSF sample that adequately represented the deranged process in the spine we were attempting to study. The patient was made to lie on one side with spine flexed in crouched hand to knee position. This flexed position ensures easy access into thecal space. Median or paramedian approach was taken as per convenience of the procedurist. Sample was only taken when the patient was comfortable with all parameters mentioned in acceptable physiological level. CSF was collected in sterile vials and protease inhibitor cocktail preparation (Roche Diagnostics, USA) was added.

As there might be a breach in blood brain barrier at the injury site, blood infiltration in the CSF was a common occurrence. We tried to deplete albumin but lost most of the other proteins along with it, and moreover our aim was to look at the actual protein scenario for the two injury conditions so we chose not to deplete any abundant protein. Therefore the first few drops of CSF were discarded and the CSF was centrifuged to remove any RBCs and cellular debris. Protein content of the CSF was determined by Bradford (Biorad, CA, USA) reagent, using BSA (Sigma Aldrich, St. Louis, MO, USA) as standard.

We did not pool CSF samples for any study. All proteomics work was conducted using individual samples as per selection criteria. CSF aliquots containing 50 µg and 25 µg protein were acetone precipitated and dissolved in 20 µl and 10 µl DIGE buffer (7 M urea, 2 M thiourea, 4% CHAPS, 30 mM Tris pH 8.8, PI cocktail, Roche diagnostics, USA) respectively and 1500 µg protein containing CSF aliquot was dissolved in 330 µl rehydration buffer (7 M urea, 2 M thiourea, 2% chaps, 60 mM DTT, 0.2% pH 3–10 ampholyte, Biorad, CA, USA). Isoelectric focussing was done with IPG strips (Biorad, CA, USA) of pH gradient 5–8 and 12% SDS-polyacrylamide gels were used for the second dimension separation.

### Differential expression analysis by DIGE

Each AIS A CSF sample was randomly paired with an AIS C or D CSF sample and Difference Gel Electrophoresis (DIGE) was conducted. 50 µg of protein for DIGE experiments, was labelled with 200 nM DMF reconstituted Cy5 dye (GE Healthcare, USA). The counterpart sample was similarly labelled with Cy3 dye. The samples were reverse labelled in half of the total number of DIGE experiments. Internal standard was made for each experiment by pooling 25 µg of protein from two samples followed by Cy2 labelling. DeCyder 2D Differential In-gel Analysis (version 6.5) software (GE Healthcare, USA) was used for the differential in gel analysis (DIA), where a threshold of 1.5 (volume ratio of Cy3 and Cy5 spots) was set and non-protein gel features were excluded manually. The gels were then analysed using the biological variance analysis (BVA) software (DeCyder 6.5). For BVA analysis, intensive repeated landmarking was performed for the gels selected in Cy2 filter. Automated gel to gel spot matching was applied, Student's t-test was performed by the software and spots that showed a particular trend of increase or decrease in three or more gels out of seven, with a p-value<0.1 was taken into consideration during analysis.

### Protein identification using MALDI-MS

For identification of CSF proteins, we used AIS A grade CSF sample. AIS A sample encompasses CSF proteins as well as serum proteins, due to higher serum permeation in complete injury cases. This factor does not confound our analysis because all proteins identified from incomplete injury (AIS C and D) CSF samples are also present in AIS A samples.

1500 µg of protein was separated on a 2D gel and stained with blue-silver stain (10% v/v orthophosphoric acid; Merck, India, 10% w/v ammonium sulphate; SRL, India, 20% v/v methanol; SRL, India, 0.12% Coomassie brilliant blue G-250; SRL, India) and spots were picked using the Proteome works spot cutter (Biorad, CA, USA). Spots were de-stained, processed for MALDI sample preparation using the processing kit (Thermo Scientific, IL, USA), overnight trypsin digested (Thermo Scientific, IL, USA) and lyophilised in Heto Vacuum Centrifuge (Thermo). α-Cyano-4-hydroxycinnamic acid (CHCA) matrix (Thermo Scientific, IL, USA) was mixed in 1∶1 ratio with 50% ACN (Thermo Scientific, IL, USA), 0.1% TFA reconstituted lyophilised spots and spotted on 192 well tungsten MALDI plates (AB Sciex, MA, USA). 4700 MALDI TOF/TOF Analyser, (AB Sciex, MA, USA) was used for matrix assisted laser desorption ionisation (MALDI) mass spectrometry.

Peptide mass fingerprint was obtained in positive MS reflector mode with fixed laser intensity of 5500, 2000–3000 laser hits in the range of 800–4000 Da. Signal to noise ratio was set at 10 and mass exclusion tolerance at 150 ppm [14]. For internal calibration, minimum signal to noise ratio was set at 20 and a mass tolerance of ±300 ppm was set which included monoisotopic peaks only. Peptides of interest were isolated at a relative resolution of 50 (full width at half maximum) and data from 3000 to 5000 laser shots were collected [14]. GPS Explorer version 3.6 (Applied Biosystems, MA, USA) was used for analysis of spectral data. MASCOT database scoring algorithm and NCBI and Swiss-Prot protein databases were used for peptide identification. Search settings: single missed tryptic cut, fixed carbamidomethylation, variable methionine oxidation, N-terminal acetylation and 150 ppm mass accuracy. Autolytic tryptic peaks were excluded in the MASCOT search parameter and p<0.05 was considered significant during identification.

### Validation by western blot

50 µg protein was dissolved in co-ip buffer (50 mM Tris, pH 7.5, 15 mM EDTA, pH 8.0, 100 mM NaCl, 0.1% Triton X 100, PMSF 100 µg/µl) and used for western blot for validation. The protein was transferred from polyacrylamide gel onto polyvinyledene difluoride (PVDF) membrane (Millipore, Billerica, MA, USA) and primary antibodies were added and kept overnight at 4°C. We validated our DIGE results for Zinc alpha 2 glycoprotein (Mouse anti human, Santa Cruz Biotechnology, TX, USA) and Haptoglobin (Mouse anti human, Santa Cruz Biotechnology, TX, USA) and used Transferrin (Mouse anti human, Abcam, Cambridge, MA, USA), as a loading control, since the total amount of this abundant protein showed little variance from patient to patient and we did not find a suitable conventionally used protein as a loading control due to very low abundance of GAPDH and possible involvement of α-Tubulin in cytoskeletal rearrangements post SCI. Goat anti mouse Ig-HRP conjugate secondary antibody (Genei, Bangalore, India) was used for two hours at room temperature after TBST wash after the removal of primary antibody. SuperSignal West Pico Chemiluminescent Substrate kit (Thermo Scientific, IL, USA) was used for X ray film (Kodak, Colorado, USA) exposure.

### Construction of the protein-protein network (PPIN)

We constructed the protein-protein interaction network (PPIN) of spinal cord injury in human subjects (denoted as SCI-PPIN) taking the proteins identified by MALDI-MS and their interacting partners listed in BioGrid (version 3.2.103, Oct 2013) as nodes. For this analysis we had to drop 4 proteins (NTAN1, NRG3, as RBBP8NL and IGHG4) whose interacting partners were not listed in BioGrid and replaced CO4 by two of its aliases namely C4A and C4B. Thus SCI-PPIN had 866 nodes comprising of 45 proteins identified by MALDI-MS and their 821 interacting partners. The nodes were then connected pair-wise when the concerned proteins were interacting partners of each other. In total the network had 7121 links (interactions). For details of the network properties, see Text S1.

### Module Detection

Modules are partitions of a network such that nodes within a partition would have large number of connections among themselves compared to nodes in other partitions. In context of protein interaction network, proteins within a module have more interacting partners and thus it is expected that they would form a complex to work together and achieve some well-defined biological function [15] fairly independently from the rest of the system. Thus, study of modularized set of proteins is relatively less noisy and enhances the significance of enrichment analysis. To find the modules of SCI-PPIN we adopted the commonly used Newman-Girvans modularization (NGM) algorithm [16], [17], [18].

### Enrichment analysis

To identify possible biological function(s) associated with a protein complex [19], we utilized GeneCodis3 [20] for enrichment analysis, where the proteins (or corresponding genes) belonging to each module of SCI-PPIN were taken as different query sets. GeneCodis would take a set of genes as the query field and associate GO terms/pathway ID/cellular component ID for each gene and calculate whether the fraction of genes in a particular item among the input gene list is over-represented compared to the background frequency (the total number of genes involved in that GO term/pathway ID/cellular component ID over the total number gene in the organism). For example, if *K* genes are involved in a particular GO term out of total *N* genes of *Homo sapiens* and if the query set has *n* genes with *k* of them associated with the concerned GO terms, we would evaluate the p-value using the hypergeometric distribution:
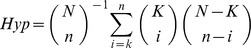
This p-value *Hyp* was further corrected for False Discovery Rate (FDR) and denoted as Hyp*. In our analysis, proteins belonging to any particular module of the SCI-PPIN were taken as input to GeneCodis3 [20] to obtain significantly enriched Gene Ontology (GO) terms for Biological Processes (BPs), KEGG pathways and cellular components. To ensure robustness we used a conservative cut-off Hyp* <0.001.

## Results

### Patient details

- *Group I (1–8 days post injury)*

The mean age of patients participating in this study group was 33.3 years with a standard deviation of 11.7 years (Table 3). All patients were male and hemiplegic. The most reported cause of injury among the participants was fall from trees. For all the participants, the upper limb motor activity was fully retained and the motor level of injury was L2.

**Table 3:** Clinical details of the 14 CSF samples collected at 1-8 days post injury.

No	Age	Sensory level of injury	Motor level of injury	Days post injury	DIGE pair	AIS grade	Cause of injury
1	22	T9	L2	3	DIGE 1	A	fall
2	36	none	L2	2	DIGE 1	D	fall
3	45	none	L2	4	DIGE 2	D	crush
4	20	T6	L2	8	DIGE 2	A	crush
5	30	none	L2	1	DIGE 3	C	fall
6	23	T12	L2	2	DIGE 3	A	fall
7	45	L4	L2	5	DIGE 4	C	fall
8	28	L1	L2	7	DIGE 4	A	fall
9	30	T12	L2	2	DIGE 5	A	fall
10	55	L1	L2	2	DIGE 5	C	fall
11	19	none	L2	3	DIGE 6	C	fall
12	50	T7	L2	8	DIGE 6	A	fall
13	25	L1	L2	6	DIGE 7	A	fall
14	38	L2	L2	4	DIGE 7	C	fall

- *Group II (15–60 days post injury)*

The mean age of patients participating in this study group was 29.5 years with a standard deviation of 12.8 years (Table 4). All patients were male and the cause of injury was fall.

**Table 4:** Clinical details of six CSF samples collected at 15–60 days post injury.

No	Age	Sensory level of injury	Motor level of injury	Days post injury	DIGE pair	AIS grade	Cause of injury
1	55	T8	L1	27	DIGE 1	A	fall
2	23	none	L1	25	DIGE 1	D	fall
3	28	T11	L1	15	DIGE 2	A	fall
4	26	none	L1	60	DIGE 2	C	fall
5	20	T9	L1	16	DIGE 3	A	fall
6	25	none	L3	30	DIGE 3	D	fall

### Eight CSF proteins show differential abundance in complete and incomplete SCI at 1–8 days post injury

On proteome analysis of AIS A grade CSF, we could identify 49 proteins (Table 5) from 129 spots in 2D gel (with identical proteins in multiple spots; see Table S1 and Figure S1). Proteomics data for all identified spots have been provided in Figure S2A and Figure S2B. The set of proteins identified from complete injury were not distinct from those identified from incomplete injury CSF proteome. Only the abundance levels of several proteins differed, as we have found from our DIGE results.

**Table 5:** Identified proteins from AIS A CSF.

Sl no	Spot No	Protein name	Gene name
1	1	Alpha 1B glycoprotein precursor	A1BG
2	4	Serine (or cysteine) proteinase inhibitor, clade C (antithrombin), member 1	SERPINC1
3	12	Alpha-1-antitrypsin	SERPINA1
4	13	Vitamin D-binding protein precursor	GC
5	14	Hemopexin precursor	HPX
6	16	Fibrinogen gamma	FGG
7	20	Fibrinogen beta chain precursor	FGB
8	26	Haptoglobin	HP
9	28	Zinc alpha 2 glycoprotein	AZGP1
10	32	Apolipoprotein E precursor	APOE
11	32	Glial fibrillary acidic protein, astrocyte (GFAP)	GFAP
12	34	Transthyretin precursor	TTR
13	38	Clusterin precursor	CLU
14	42	Ig kappa chain C region	IGKC
15	42	Prostaglandin H2 D-isomerase	PTGDS
16	46	Apolipoprotein A-I precursor	APOA1
17	51	Peroxiredoxin 2	PRDX2
18	52	Complement C4 precursor	CO4
19	54	AMBP protein precursor	AMBP
20	60	Protein N-terminal asparagine amidohydrolase	NTAN1
21	61	Apolipoprotein A-IV precursor	APOA4
22	62	Carbonic anhydrase I	CA1
23	66	Hemoglobin beta chain	HBB
24	71	Ficolin 3 precursor	FCN3
25	73	Creatine kinase, M chain	CKM
26	76	SH3-domain kinase binding protein 1	SH3KBP1
27	81	WD-repeat protein 37	WDR37
28	81	Nonspecific lipid-transfer protein, mitochondrial precursor	SCP2
29	83	Transferrin	TF
30	85	Ig mu chain C region	IGHM
31	86	Gelsolin isoform b	GSN
32	94	Ig alpha-1 chain C region	IGHA1
33	96	Glypican-1 precursor	GPC1
34	96	Phenylalanine-4-hydroxylase	PAH
35	96	Pro-neuregulin-3 precursor (Pro-NRG3)	NRG3
36	97	Heat shock 70 kDa protein 4L (Osmotic stress protein 94)	HSPA4L
37	99	Retinoic acid receptor gamma-1	RARG
38	99	3-hydroxyanthranilate 3,4-dioxygenase	HAAO
39	99	Protein C20orf151 (RBBP8 N-terminal-like protein)	RBBP8NL
40	100	Astrotactin 1 (Fragment)	ASTN1
41	103	Heat shock 70 kDa protein 4L (Osmotic stress protein 94)	HSPA4L
42	104	Beta-2-glycoprotein I precursor (Apolipoprotein H)	APOH
43	104	General transcription factor 3C polypeptide 5	GTF3C5
44	107	Serum albumin precursor	ALB
45	112	Ig gamma-2 chain C region	IGHG2
46	116	TBC1 domain family member 15	TBC1D15
47	119	Ig gamma-1 chain C region	IGHG1
49	121	Ig gamma-3 chain C region	IGHG3
49	126	Ig gamma-4 chain C region	IGHG4
50	128	Serum paraoxonase/arylesterase 1	PON1

L-R: serial number, spot number (the spot number here and in subsequent tables corresponds to Figure S1 and Table S1), protein name and gene name.

**Figure 1:**
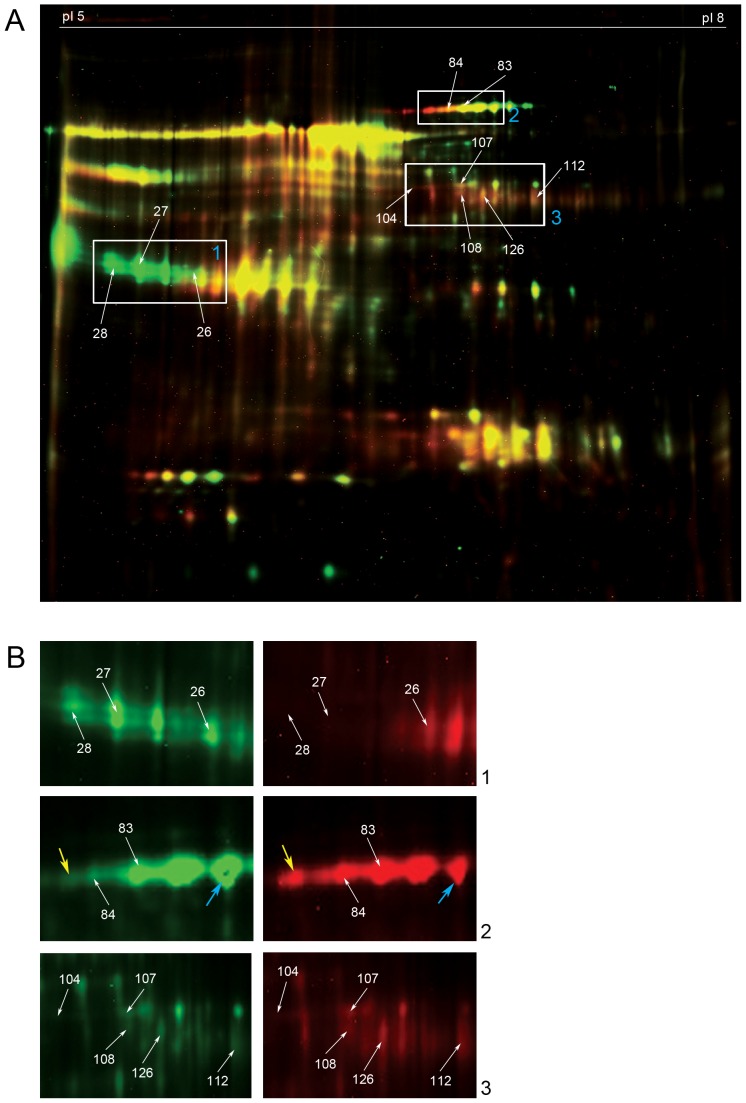
Differential abundance pattern by DIGE at 1–8 days post injury. A. A representative merged image of DIGE experiment with numbers indicating spot numbers. Complete injury CSF sample is labelled with Cy5 and incomplete injury CSF sample is labelled with Cy3. Proteins that show significant differential abundance are marked with white arrows and are boxed. B. Boxes 1, 2 and 3 with significant differential abundance of proteins are enlarged. Left hand panels show incomplete injury (Cy3 channel) image and the right hand panels show complete injury (Cy5 channel) image. Expression patterns of Transferrin are marked with yellow (acidic isoform) and blue (basic isoform) arrows in box 2.

We identified eight proteins which showed differential abundance (Figure 1A) among the two injury severities at 1–8 (average: 4.07 days) days post injury by DIGE and subsequent BVA analysis (n = 7). These eight proteins belonged to ten spots (Figure S3). Two proteins have been identified from spot 104. Three proteins, namely, Haptoglobin (26 and 27), serum albumin precursor (spots 107 and 108) and Transferrin (spots 83 and 84) were present in two different spots each as isoforms (Figure 1B). Curiously, a bias was observed in the isoform distribution of Transferrin (Figure 1B, panel 2), where, a lower pI isoform was more abundant in complete injury CSF whereas a higher pI isoform showed greater abundance in incomplete injury CSF.

**Figure 2:**
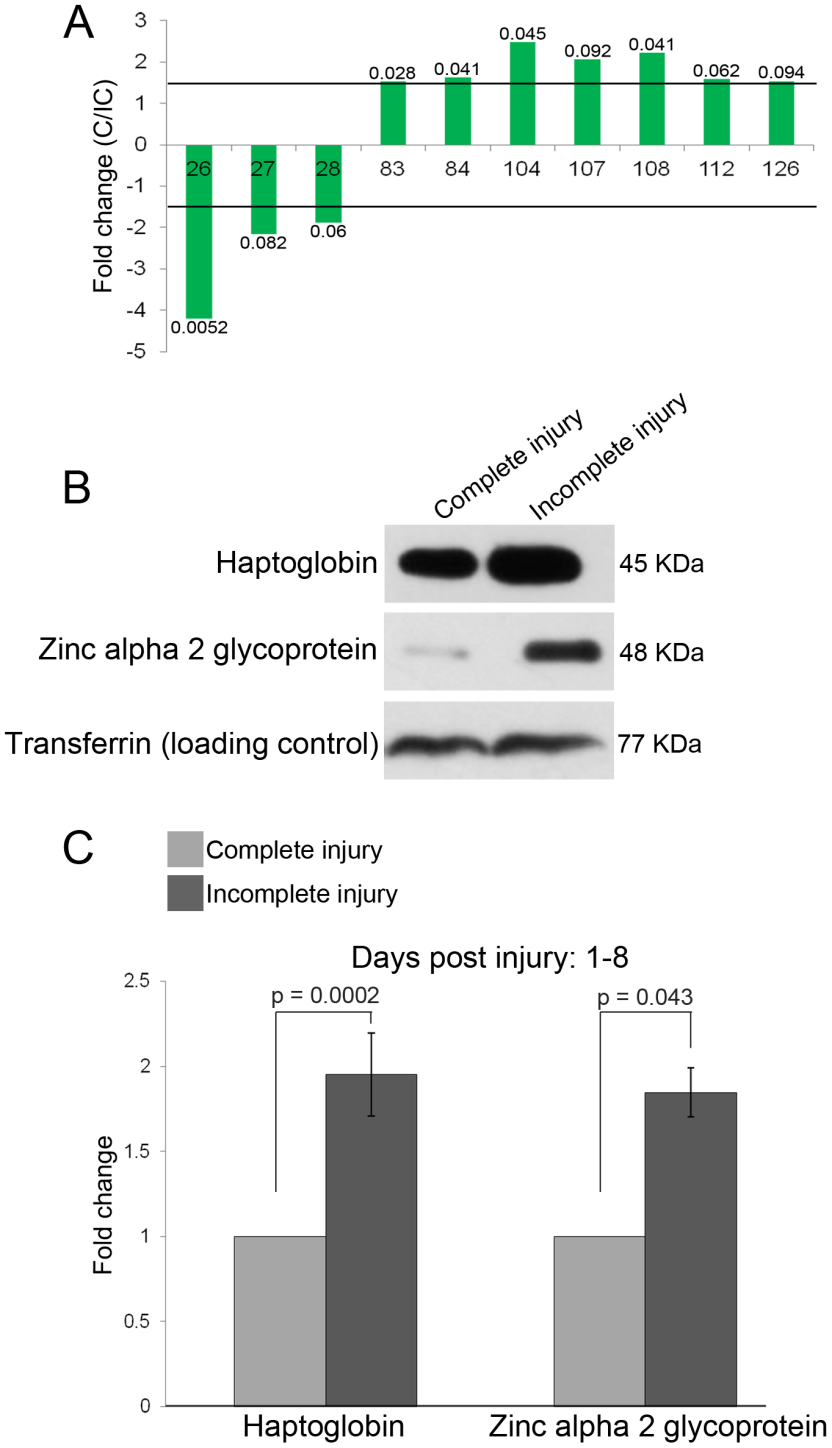
Differentially expressed proteins in complete and incomplete injury at 1–8 days post injury. A. Representation of fold changes (complete injury to incomplete injury) of differentially expressed proteins (≥ ±1.5 fold; outside the range between the lines) with p-values (n = 7) for each spot calculated by BVA (see Figure S3). B. Scanned Western Blot images for validation of fold changes of HP and AZGP. The observed band for AZGP was obtained a few kilo Daltons above the expected region and has been observed (as a possible result of glycosylation) by the manufacturers of the antibody. C. Quantification of the western blot (n = 6) showing significant difference in expressions.

The proteins that were more abundant in complete injury CSF at 1–8 days post injury were Transferrin (acidic isoforms, spots 83 and 84), Beta-2 glycoprotein I precursor and General transcription factor 3C polypeptide 5 (spot 104), Serum albumin precursor (spots 107 and 108), Immunoglobulin gamma-2 chain C region (spot 112) and Immunoglobulin gamma-4 chain C region (spot 126) (Figure 2A, Table 6 and Figure S3) and those less abundant in complete injury CSF were Haptoglobin (spots 26 and 27) and Zinc alpha 2 glycoprotein (spot 28). The total content of Transferrin encompassing all its isoforms was constant, as seen by western blot (Figure 2B and C) and subsequent laser scanning.

**Table 6:** List of proteins showing differential expression by DIGE.

Spot no	Protein name	Average fold change (1–8 DPI)	Average fold change (15–60 DPI)
26	Haptoglobin	−4.21	0.49
27	Haptoglobin	−2.15	1.37
28	Zinc alpha 2 glycoprotein	−1.88	0.66
83	Transferrin	1.52	0.52
84	Transferrin	1.63	0.45
104	Beta-2-glycoprotein I-precursor (Apolipoprotein H)	2.47	−0.6^A^
104	General transcription factor 3C polypeptide 5	2.47	−0.6^A^
107	Serum albumin precursor	2.07	2.23^A^
108	Serum albumin precursor	2.23	1.2^A^
112	Ig gamma-2 chain C region	1.58	1.47^A^
126	Ig gamma-4 chain C region	1.52	2.13^A^

**Figure 3:**
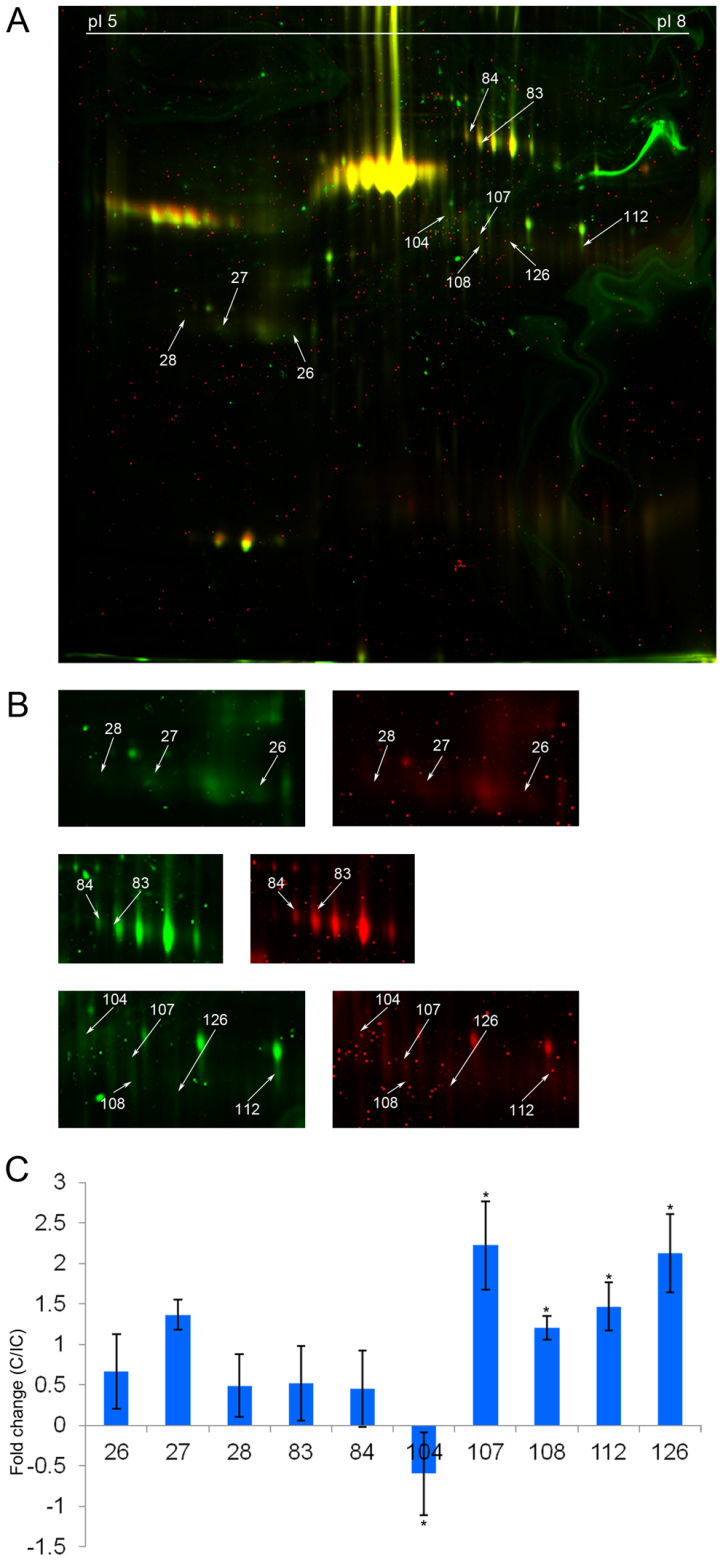
Differential abundance pattern by DIGE at 15–60 days post injury. A. Representative image with spot numbers. Complete injury CSF sample is labelled with Cy3 and incomplete injury CSF sample is labelled with Cy5. B. Enlarged areas of the gel showing the differentially abundant spots in detail. Left hand panel represents complete injury (Cy3 channel) image and right hand panel represents incomplete injury (Cy5 channel) image. C. Average fold change (complete/incomplete injury) for each protein (n = 3). Starred values have been obtained from 2 experiments.

L-R: Spot number, protein name, average fold change of differential abundance at 1–8 days post injury (DPI) (Complete injury/Incomplete injury, n = 7, BVA algorithm used; Figure 2A and Figure S3), average fold change at 15–60 DPI (n = 3, manually calculated; Figure 3C).

A These spots were obtained in two out of three experiments.

### Abundance levels of the eight proteins at 15–60 days post injury

**Figure 4:**
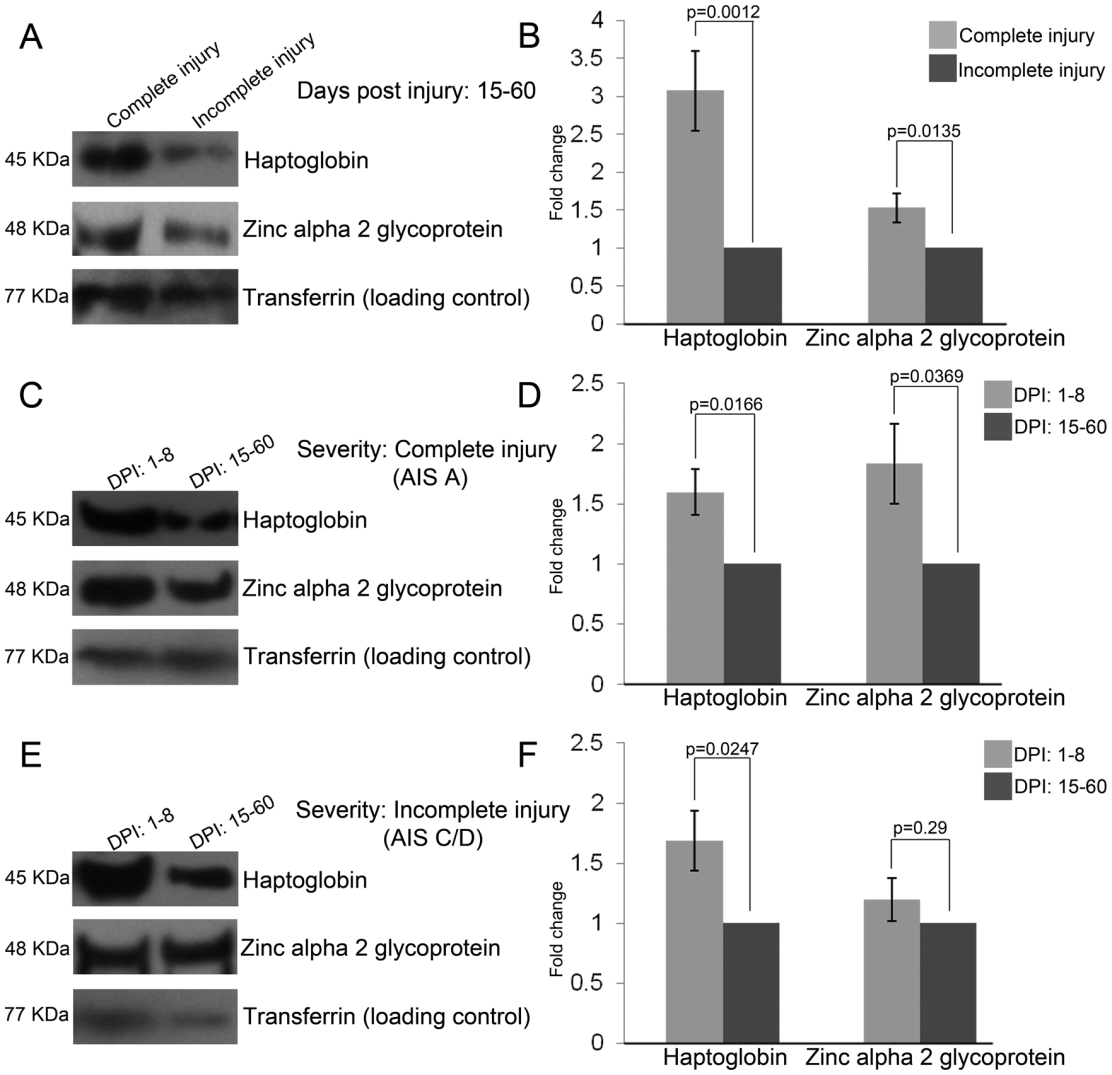
Changes in abundance levels of HP and AZGP on a temporal manner. A. Scanned blot images for these proteins at 15–60 days post injury (DPI) for the two severity groups. B. Quantification of the blots showing significant difference in expression. Comparison of abundance for HP and AZGP in complete injury (C) and incomplete injury (E), between two time periods. Quantification for the same (D, F) respectively (n = 4).

We looked at the levels of the eight proteins in CSF drawn at 15-60 days post injury (average: 28.83 days). Haptoglobin (spots 27 and 28) and Zinc alpha 2 glycoprotein (spot 26) showed a reversal in their abundance profiles. Both proteins were found to be more abundant in complete injury CSF during this time period as seen by DIGE (n = 3) (Figure 3A and B, Table 6). Transferrin levels showed similar abundance (within the threshold of ±1.5 fold) in complete injury CSF (Figure 3C, Table 6). Unlike the findings for Transferrin at 1–8 days post injury, the bias in abundance between different isoforms was not observed among the two severity groups at 15–60 days post injury. Serum albumin precursor (spots 107 and 108), Immunoglobulin gamma 2 chain C region (spot 112) and Immunoglobulin gamma 4 chain C region (spot 126) continued to be more abundant in complete injury CSF, while the spot pertaining to General transcription factor 3 C polypeptide 5 and Beta 2 glycoprotein 1 precursor (spot 104) showed marginal reduction in abundance for complete injury CSF (within the threshold of ±1.5 fold). Western blot for Haptoglobin and Zinc alpha 2 glycoprotein corroborated the findings by DIGE (Figure 4A and B).

### Temporal changes in abundance of Haptoglobin and Zinc alpha 2 glycoprotein in both injury severities

We observed that the overall abundance level of Haptoglobin and Zinc alpha 2 glycoprotein decreased at 15–60 days post injury as compared with their levels at 1–8 days post injury. This trend was observed for both complete injury (Figure 4C and D) and incomplete injury (Figure 4E and F) by western blot.

### Enrichment analysis revealed six functional modules with perturbed members

**Figure 5:**
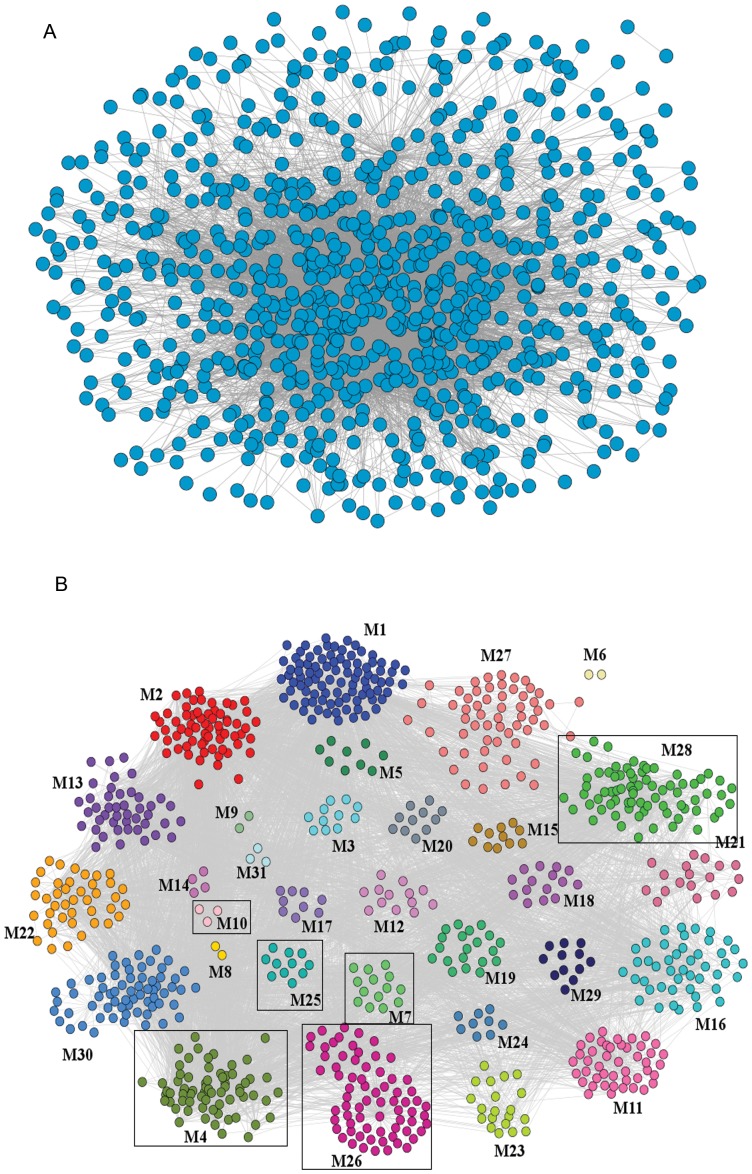
The spinal cord injury protein-protein interaction network. A. SCI-PPIN representation of 866 proteins (blue dots) and 7121 interactions (grey lines). B. Modularised SCI-PPIN consisting of 31 modules, dots representing proteins and coloured differently to demarcate different modules. Module numbers correspond to Table S2. The modules which contain proteins having differential abundance and detected by DIGE are boxed.

The constructed SCI-PPIN was unweighted and dense having about 25% of the total connections possible. The visual presentation of the SCI-PPIN was done using Cytoscape [21] (Figure 5A). We found 31 modules of different sizes in SCI-PPIN with number of members varying in the range 2 to 91 (Figure 5B, Table S2 and Table S3). The enrichment analysis revealed several statistically significant biological processes, cellular components and KEGG pathways, the major ones being those of protein transport and metabolism, DNA repair, cell division, migration and adhesion, immune response, apoptosis, lipid and cholesterol metabolism, transcription, complement activation, vesicle endocytosis, tRNA metabolism, iron transport and axon growth (Table 7). We focussed on 6 out of 31 modules where the differentially expressed proteins from DIGE experiments belonged (Figure 5B, Table 7). These pathways were assumed to be perturbed in the event of severe SCI, as their protein components showed altered abundance in a severe injury scenario.

**Table 7:** Details of 31 modules generated through enrichment analysis.

Module ID	No. of proteins	Enriched Biological process	Enriched Kegg pathways	Enriched Cellular components
M1	91	Protein transport, fatty acid oxidation	Peroxism, PPAR signalling pathway^B^	Peroxism and cytoplasm
M2	65	RNA metabolism, epidermis development	RNA dynamics, ubiquitin mediated proteolysis	Nucleus and cytoplasm
M3	11	mRNA metabolism and transport	mRNA surveillance and transport	Nucleus and cytoplasm
M4^A^	68	Protein phosphorylation, mRNA metabolism	MAPK and Wnt signalling, meiosis	Nucleus and cytoplasm
M5	9	Glucocorticoid receptor signalling^B^	Wnt signalling^B^	Nucleus
M6	2	Complement activation	No annotations	Extracellular region
M7^A^	14	Iron transport, insulin receptor signalling^B^	P53 signalling^B^	Insulin like growth factor binding protein complex
M8	2	Meiotic sister chromatid cohesion, cell division^B^	No annotations	Mitotic cohesion complex
M9	2	No annotations	No annotations	No annotations
M10^A^	3	Lipid and ATP catabolism, immune response^B^	ABC transporters^B^	MHC class I protein complex^B^
M11	45	Vesicle endocytosis	Endocytosis	Cytoplasm and cytoskeleton
M12	13	Cell shape, migration and adhesion	Chemokine signalling and axon guidance	Plasma membrane, cytoskeleton, cytoplasm, membrane rafts
M13	44	NGF and T cell receptor signalling, axon guidance	Cancer	Cytoplasm and plasma membrane
M14	4	Axogenesis, NGF and Rho signalling	Actin cytoskeleton and chemokine signalling	Cytoplasm
M15	11	Lipoprotein metabolism and transport	No annotations	Coated pit
M16	45	Mitosis, blood coagulation, platelet activation	Regulation of actin cytoskeleton^B^	Cytoplasm, cytoskeleton
M17	9	Cerebellum development^B^, meiosis^B^	Type II diabetes, dialeted cardiomyopathy^B^	Cytoplasm^B^
M18	15	Protein metabolism in the ER	Protein processing in the ER	Endoplasmic reticulum
M19	20	Complement activation and Cu ion homeostasis	Complement and immune response	Trans golgi network, membrane attack complex, extracellular space
M20	11	Apoptosis	Neurological disorders, cancer and apoptosis	Mitochondria and cytoplasm
M21	18	Removal of superoxide radicals^B^	Amylotropic lateral sclerosis^B^	Cytoplasm
M22	41	Sarcomere organisation^B^	Cardiomyopathy	Cytoplasm and nucleus
M23	20	Cellular component movement, brain morphogenesis	Adherens junctions	Z disc and ciliary rootlet
M24	9	Mitosis and protein ubiquitination	Cell cycle and ubiquitination	Nucleus and cytoplasm
M25^A^	11	tRNA and rRNA transcription	RNA polymerase^B^	Nucleus
M26^A^	71	DNA repair	P53 pathway and basal transcription factor	Nucleus
M27	58	Lipid and cholesterol metabolism	Complement, coagulation, PPAR signalling	Extracellular space
M28^A^	74	Organ regeneration^B^	E. coli infection	Cytoplasm and extracellular space
M29	12	Apoptosis^B^	Apoptosis and cancer	Cytoplasm and cell membrane
M30	65	Transcription, NGF receptor signalling	Cancer, TGF-beta and PPAR signalling	Nucleus and cytoplasm
M31	3	B and T cell tolerance induction^B^	TGF-beta signalling^B^	TGF-beta receptor complex

A Modules containing the proteins which show differential abundance in DIGE, and therefore, thought to represent perturbed biological pathways. ^B^Biological processes, KEGG pathways and cellular components that are non-significant according to Hyp*<0.001, but are still listed here as they are known to be significant in other modules. For details of enrichment analysis of Biological Process, see Table-S3.

## Discussion

SCI typically throws the normal functioning of Central Nervous System (CNS) and adjoining tissues into a haywire, as is evident from our study and several others [3], [4], [22]–[29], [36]. Two perspectives emerge in this scenario: the off-balance situation of the CNS pathophysiology and the start and progression of efforts to bring back homeostasis and repair. The analysis of protein interaction network constructed from proteins associated with SCI reveals that both categories of pathways are altered.

Starting with the initial impact on the spinal cord, a breach of blood brain barrier may occur along with extensive cell and tissue damage. Consequences are myelin membrane disruption, DNA damage and iron toxicity. Transcriptomics studies of SCI in rodents and humans [22], [23] have reported the involvement of cholesterol biosynthesis, myelination, transcription regulation and apoptosis pathways. Especially cholesterol biosynthesis would be predominant in the post injury scenario as myelination is necessary to replace the damaged axon membranes. In this context, PPAR-α has been shown to be over-expressed at the lesion site [24] mediating anti-inflammatory properties of drugs like Simvastatin [25]. DNA damage is another major consequence of cell disruption after trauma. It necessitates the activation of p53, which minimises inflammatory processes initiated by microglia and promotes DNA repair, cell division and axon genesis [26]. Microglial activation also initiates release of a number of beneficial trophic factors like BDNF following MAPK signalling pathway [27]. Iron homeostasis [28] and blood coagulation pathways are also active during this phase. Administration of Resveratrol in SCI rats has shown upregulation of IGF-1 and Wnt1 mRNA indicating the involvement of insulin receptor signalling and Wnt pathways in post SCI pathophysiology [29]. tRNA transcription, which is a perturbed pathway in our study, probably points towards increased protein synthesis and has not been reported so far.

An interesting finding from our proteomics study is the higher abundance of Zinc alpha 2 glycoprotein (AZGP) and Haptoglobin (HP) in incomplete injury CSF at 1–8 days post injury. Both being serum proteins, they can be presumed to have higher abundance in more severe injury because of serum contamination of CSF. Contrary to this expectation, their predominance in incomplete injury CSF at an early phase of secondary injury suggests possible role in facilitation of recovery process after tissue injury. Furthermore, the revelation of changed abundance pattern of these non inflammatory proteins at a later time period, suggests that the possibility in their likely involvement post injury cannot be ruled out. We also noticed that over the period of weeks during which the secondary injury process progresses, the levels of these proteins decline in the CSF of both injury types. This observation allows us to further hypothesize the involvement of HP and AZGP in an early secondary injury phase.

Many of the proteins identified in CSF do not show significantly different abundance in AIS severity groups despite having roles in neuronal regeneration. Retinoic acid receptors can initiate neurite outgrowth in retinal degeneration cell models [30] and direct the transformation of fibroblasts into neurons [31]. There is increased secretion of ApoE and increased recruitment of ApoA1 by injured peripheral nerves [32] during their regenerative phase. In CSF from complete SCI, we identified several immune response proteins like Glial fibrillary acidic protein (GFAP), an inflammatory biomarker in SCI [11], complement precursors and Ficolin 3 precursor which substantiate the role of immune response post SCI [3]. Ficolin 3, Haptoglobin, Alpha-1-antitrypsin, Hemopexin precursor and IgM chain also showed increased expression in plasma of rheumatoid arthritis patients [33] as an indication of immune response.

A non-sialated version of Transferrin, called Beta-2 Transferrin, is more predominant in CSF than in blood [34] and has been used as a diagnostic biomarker for CSF leakage. This carbohydrate free Beta-2 Transferrin is less acidic than the sialated isoforms and suggests that the preponderance of a higher pI isoform in incomplete injury (Figure 1B, panel 2) is a result of lesser blood infiltration.

In this work we have constructed the protein interaction network comprising of proteins that were identified from CSF of complete injury SCI patients. It turns out that the network has strong underlying modular structure and keeping the proteins which exhibit differential abundance in CSF (complete versus incomplete SCI) in view, several modules were highlighted (Figure 5B). As proteins essentially act in conjunction with one another in the form of pathways, we could identify the molecular pathways that are perturbed in complete SCI.

The modularisation reveals that mRNA metabolism, tRNA and rRNA transcription, protein phosphorylation, lipid catabolism, immune response, iron transport, DNA repair and ATP catabolism pathways are the predominant pathways being perturbed in complete SCI. The picture that emerges here is of the system switching on certain very basic molecular mechanisms on the event of trauma. Chronologically placed, a lot of protein synthesis would have been initiated for repair and regeneration as manifested in our study by mRNA metabolism pathways being perturbed. tRNA and rRNA synthesis should get upregulated to initiate protein synthesis on a larger scale. A number of signalling pathways must follow suit and we see protein phosphorylation pathways emerging. The damaged myelin membrane has to be repaired or replaced necessitating lipid metabolism. It may be noted here that the cholesterol transport protein ApoA1 has shown differential abundance in several of the DIGE experiments (although statistically insignificant in our final analysis) and there have been reports linking ApoA1 to activation of Cdc42 [35] and F-actin polymerisation further downstream. How could these pathways be involved in regenerative mechanisms is worth looking into.

We have also seen that the breach of blood brain barrier during the mechanical trauma elicits immune response [3], [36]. On the damage control front, excess free iron released by RBC rupture is being sequestered and transported away from the damaged site. Damaged cellular DNA repair mechanisms are also being initiated. Finally, as a cumulative effect, the traumatized system initiates these biological processes in expense of energy (ATP catabolism).

This combinatorial analysis therefore creates a picture of the real time molecular map around the injury site in the secondary phase. External intervention by manipulating the potentially important of these pathways could usher in greater regenerative efforts, though such an extrapolation is futuristic at this moment. CSF being a circulating fluid rather than a solid tissue, it is highly likely that the highlighted pathways are not only predominant at the injury site but are general consequences of the SCI trauma. Considering the limitations to obtain solid tissue from living human subjects and the methodology adopted, this study, to the best of our knowledge, is first of its kind in that it reflects the patho-physiological situation in the milieu of the injured cord by means of protein expression and abundance and a statistical analysis rather than by transcriptome analysis.

## Conclusions

Traumatic SCI debilitates thousands of people worldwide every year. There have been many studies to decipher the molecular scenarios in the injured spinal cord and we have approached this problem as an analysis of differentially expressed proteins in complete and incomplete injury forms. Because the prognosis of incomplete injury cases is much better than those of the complete injury types, pathways perturbed among these two scenarios directly point to the molecular processes that may be responsible for the recovery patterns. Consequently, as the study has been conducted in the human system, the results could be attempted to be translated to animal models.

## Supporting Information

Figure S1 **Representative 2D gel of SCI (AIS A) CSF.** Identified protein spots are numbered.
(PDF)

Figure S2 **Mass spectrometry analysis details of all identified spots.** A. MS and MSMS spectra. B. Probability based Mowse scores for each spot.
(PDF)

Figure S3 **Differential abundance ratios of the eight differentially abundant proteins from complete and incomplete injury SCI CSF.**
(PDF)

Table S1 **Details of identified spots.** L-R: Spot number (corresponds to Figure S1), protein name, NCBI/Swiss Prot accession numbers, pI, molecular weight, Mowse score, expect value, sequence coverage, peptide count, peptides matched.
(XLS)

Table S2 **Composition of the modules.** Each module is mentioned per column followed by the number of members in that module. The member proteins are listed in each column. Proteins identified in this study have been highlighted in grey and red. Among these, proteins that show differential abundance in DIGE have been highlighted in red. Note that “module” has been referred to as “group”.
(XLS)

Table S3 **Details of the modules.** Protein id, GO annotations, annotation details and component genes for each module per sheet. GO annotations highlighted in green are statistically significant (Hyp^*^<0.001) and those highlighted in yellow are not statistically significant but included for their significant biological roles in the current perspective.
(XLS)

Text S1 **Properties and parameters of the SCI-PPIN.**
(PDF)
