# Supplementary material for: CSF Proteomics of Secondary Phase Spinal Cord Injury in Human Subjects: Perturbed Molecular Pathways Post Injury
Source: PLoS One. 2014 Oct 28;9(10):e110885. doi: 10.1371/journal.pone.0110885 (PMC4211693; doi:10.1371/journal.pone.0110885)
Supplement: Figure S1 — Representative 2D gel of SCI (AIS A) CSF. Identified protein spots are numbered. (PDF) [file pone.0110885.s001.pdf]

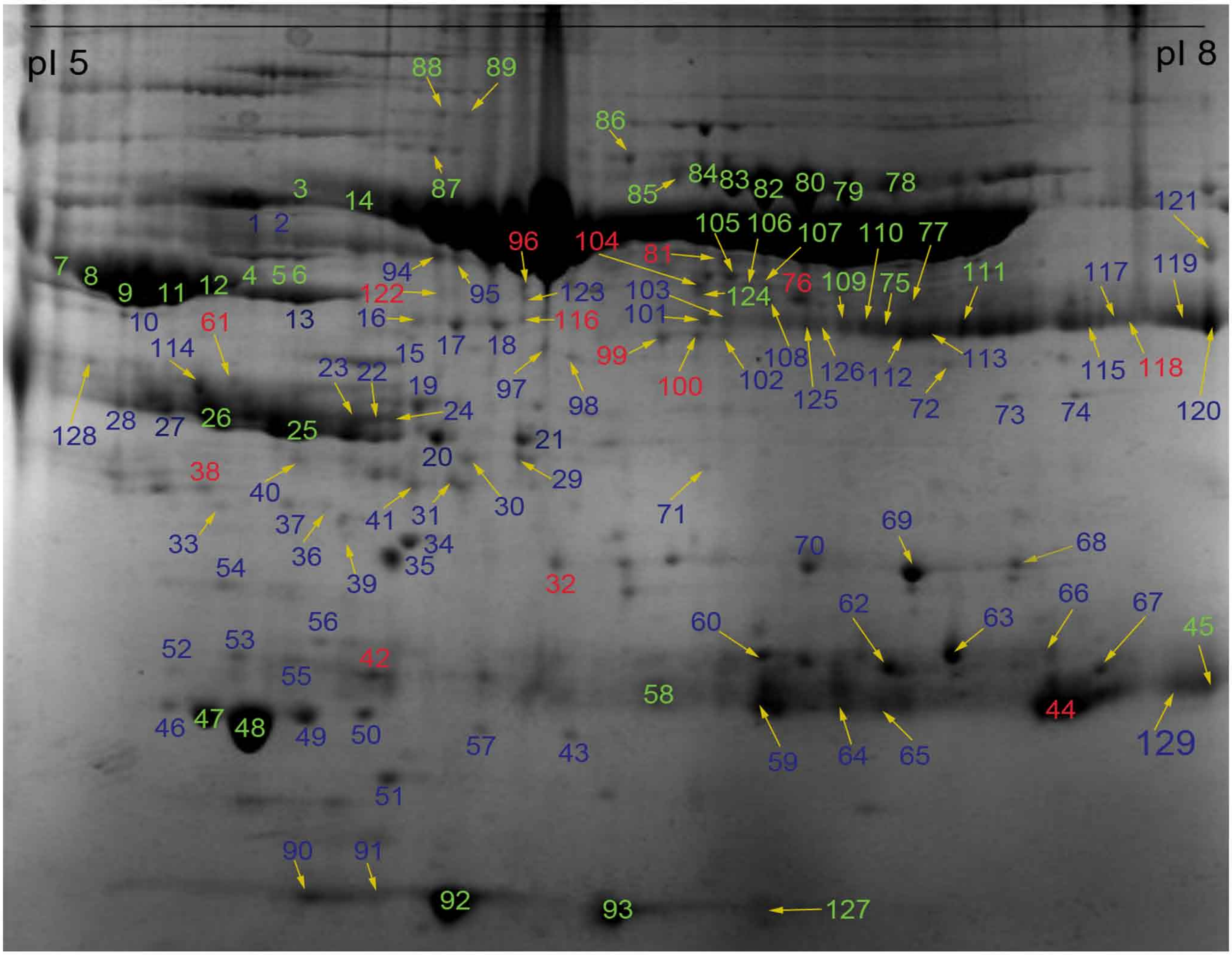


Figure-S1

The identified proteins from AIS A CSF gel. The spots marked in red contain more than one protein. The other colours have been added to facilitate easy visualisation. For the details of all the spots, refer to Table-S1
