# Supplementary material for: CSF Proteomics of Secondary Phase Spinal Cord Injury in Human Subjects: Perturbed Molecular Pathways Post Injury
Source: PLoS One. 2014 Oct 28;9(10):e110885. doi: 10.1371/journal.pone.0110885 (PMC4211693; doi:10.1371/journal.pone.0110885)
Supplement: Figure S2 — Mass spectrometry analysis details of all identified spots. A. MS and MSMS spectra. B. Probability based Mowse scores for each spot. (PDF) [file pone.0110885.s002.pdf]

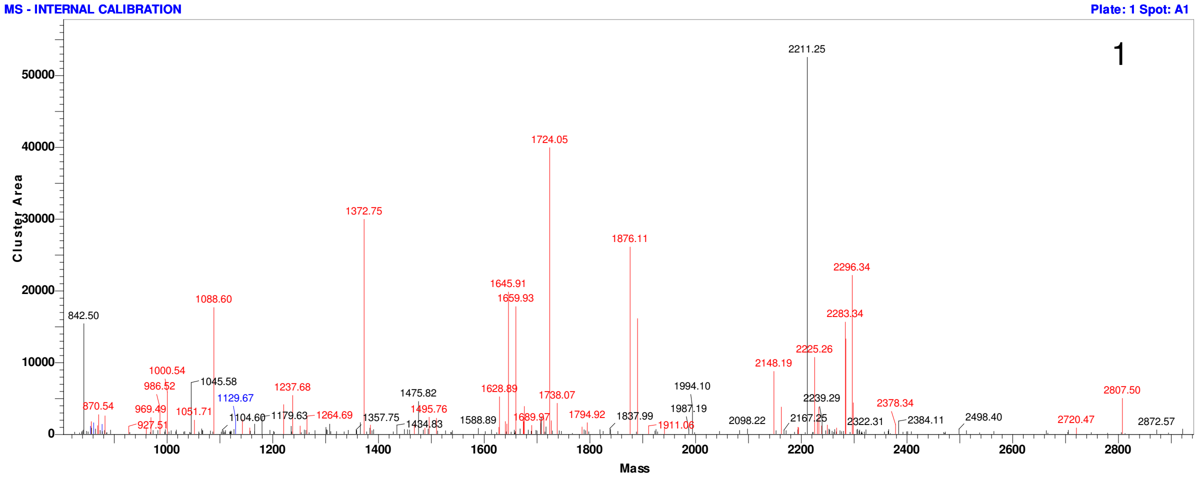
MS - INTERNAL CALIBRATION
Plate: 1 Spot: A1
1
Cluster Area
Mass
2211.25
1724.05
1372.75
1876.11
2296.34
1645.91
1659.93
1088.60
842.50
2283.34
2225.26
2148.19
1000.54
986.52
1045.58
1237.68
1475.82
1628.89
1994.10
2807.50
1129.67
1738.07
870.54
969.49
1051.71
1495.76
1987.19
2239.29
2378.34
927.51
1104.60
1179.63
1264.69
1357.75
1434.83
1588.89
1689.97
1794.92
1837.99
1911.06
2098.22
2167.25
2322.31
2384.11
2498.40
2720.47
2872.57

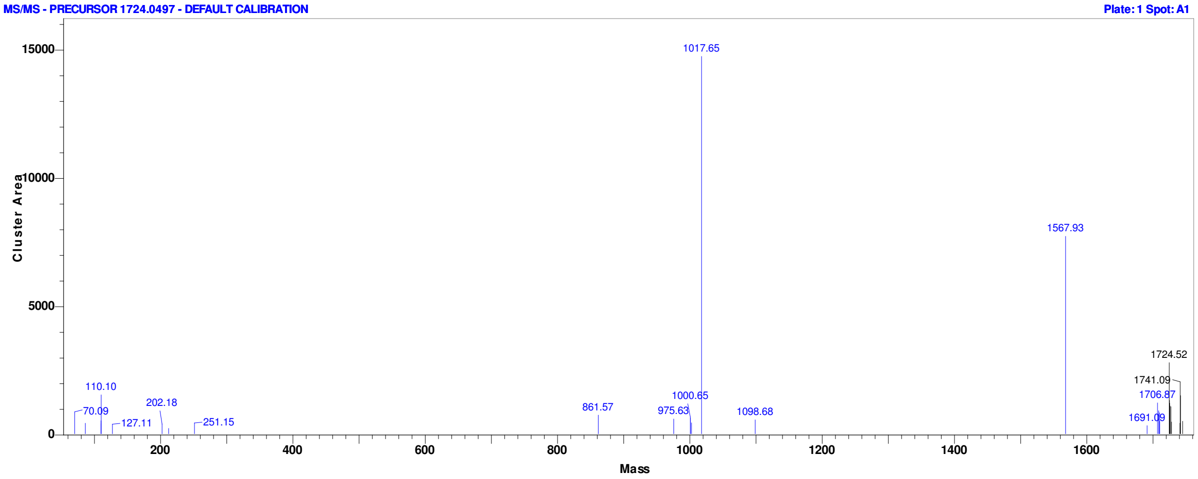
MS/MS - PRECURSOR 1724.0497 - DEFAULT CALIBRATION
Plate: 1 Spot: A1
Cluster Area
Mass
1017.65
1567.93
1724.52
1741.09
1706.87
1691.09
110.10
70.09
202.18
127.11
251.15
861.57
1000.65
975.63
1098.68

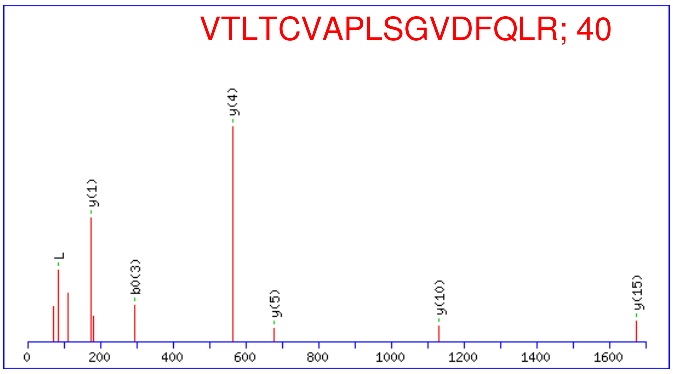
VTLTCVAPLSGVDFQLR; 40
L
y(1)
b0(3)
y(4)
y(5)
y(10)
y(15)

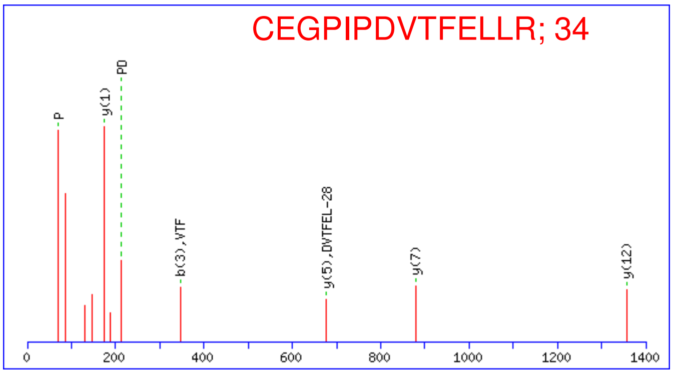
CEGPIPDVTFELLR; 34
P
y(1)
PD
b(3),VTF
y(5),DVTFEL-28
y(7)
y(12)

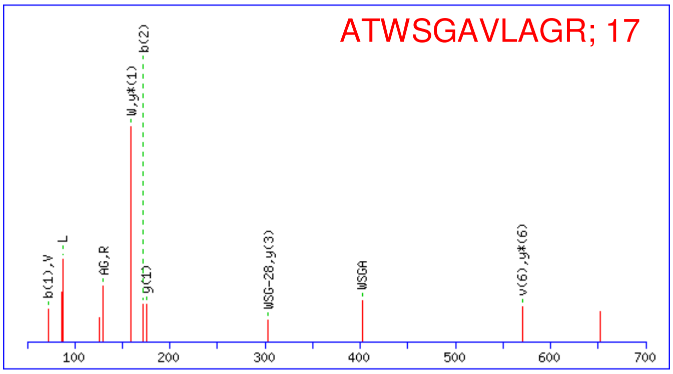
ATWSGAVLAGR; 17
b(1),V
L
AG,R
W,y*(1)
b(2)
y(1)
WSG-28,y(3)
WSGA
y(6),y*(6)

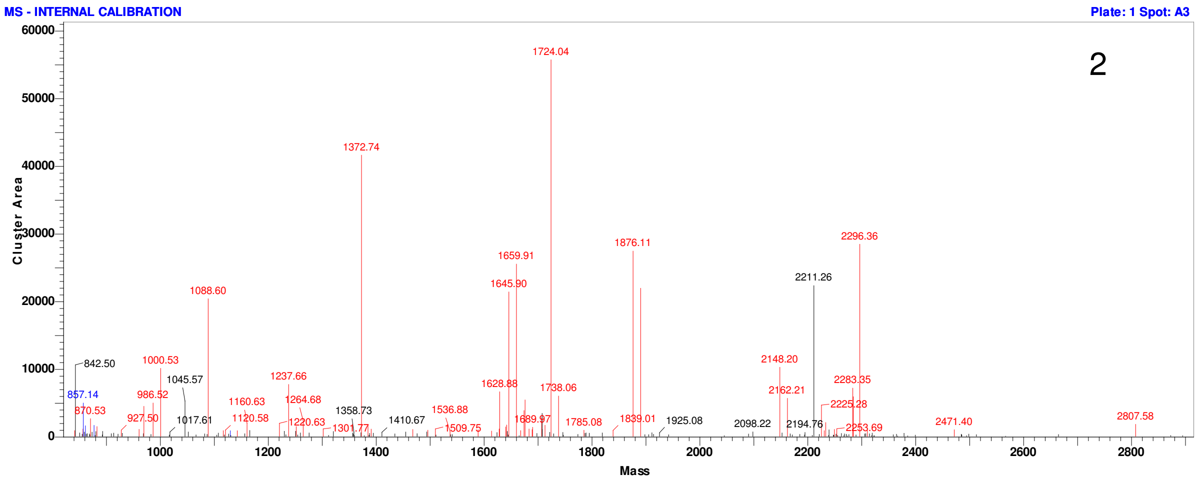

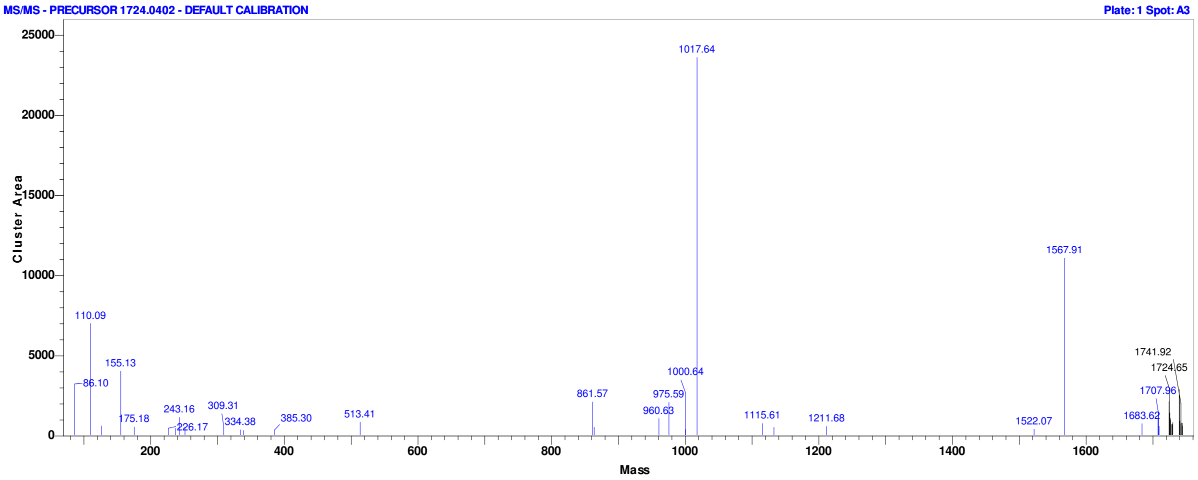

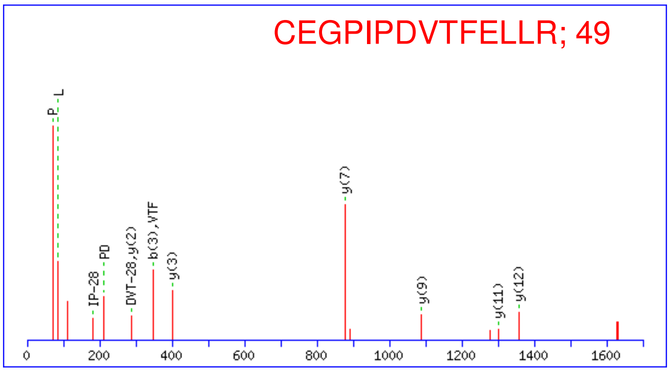

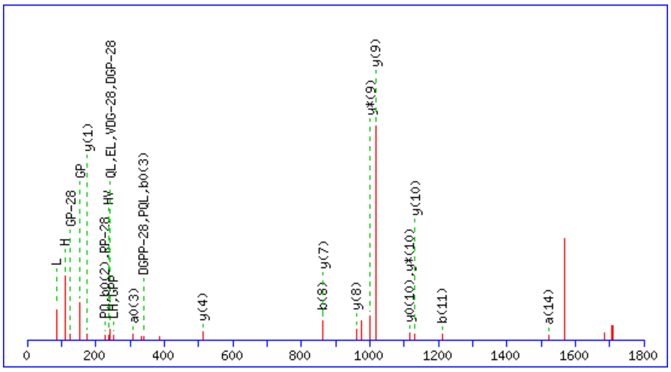

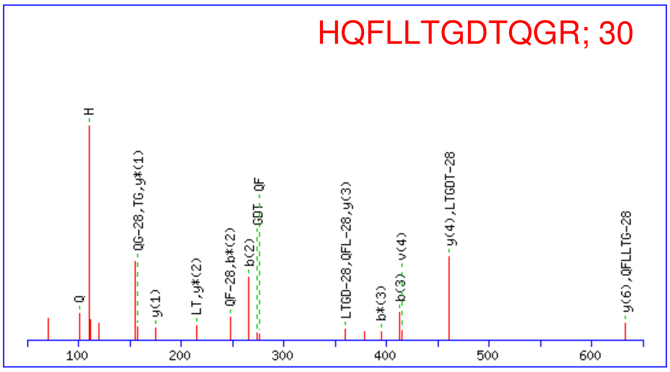

LELHVDGPPPRPQLR; 35

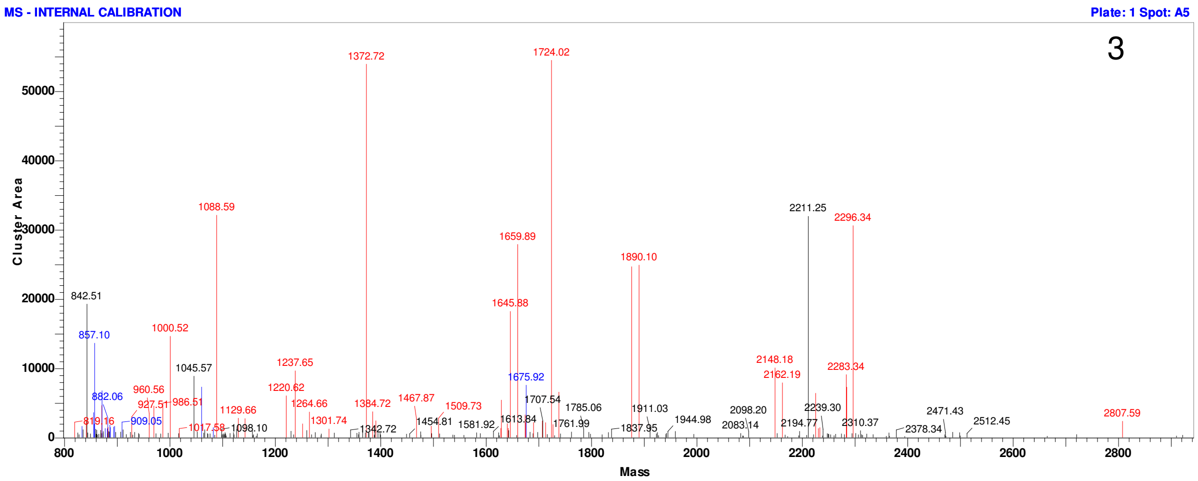
MS - INTERNAL CALIBRATION
Plate: 1 Spot: A5
3
Cluster Area
Mass
1372.72
1724.02
1088.59
2211.25
2296.34
1659.89
1890.10
1645.88
842.51
857.10
1000.52
1045.57
1237.65
2148.18
2162.19
2283.34
1675.92
1220.62
960.56
882.06
927.51
986.51
1264.66
1384.72
1467.87
1509.73
1707.54
1785.06
1911.03
2098.20
2239.30
2471.43
2807.59
819.16
909.05
1017.58
1129.66
1098.10
1301.74
1342.72
1454.81
1581.92
1613.84
1761.99
1837.95
1944.98
2083.14
2194.77
2310.37
2378.34
2512.45

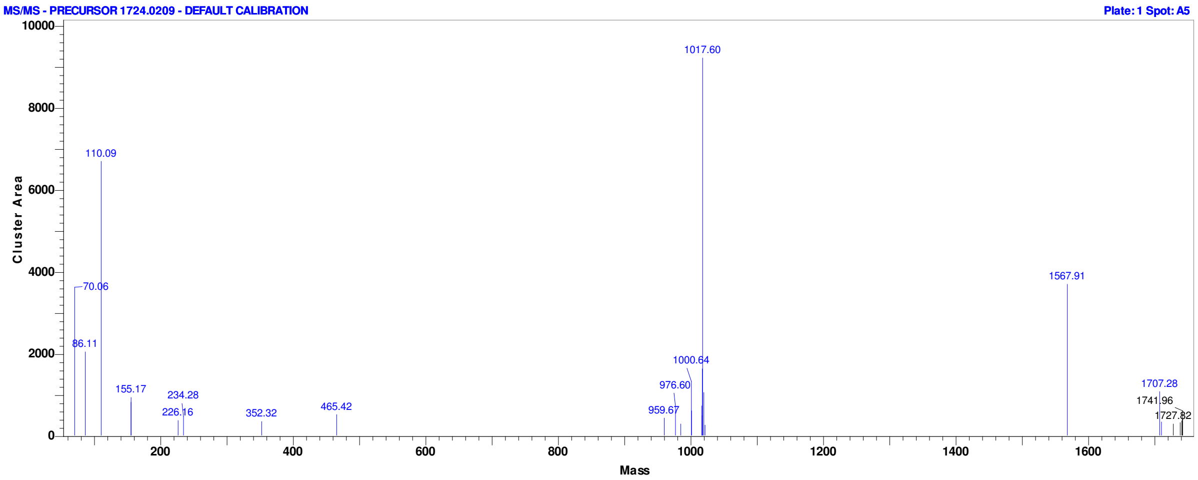
MS/MS - PRECURSOR 1724.0209 - DEFAULT CALIBRATION
Plate: 1 Spot: A5
Cluster Area
Mass
1017.60
110.09
70.06
86.11
155.17
234.28
226.16
352.32
465.42
1000.64
976.60
959.67
1567.91
1707.28
1741.96
1727.82

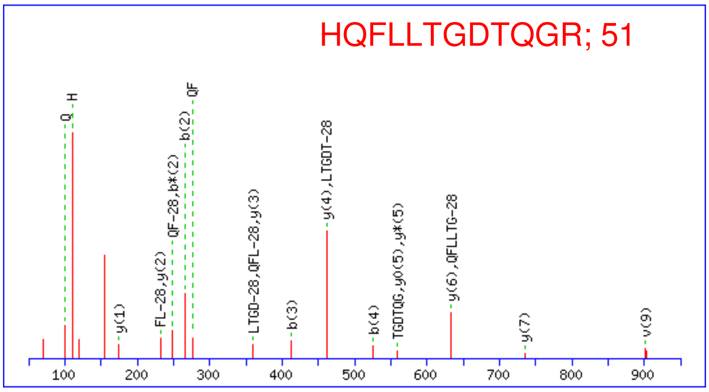
HQFLLTGDTQGR; 51
Q
H
y(1)
FL-28,y(2)
QF-28,b*(2)
b(2)
QF
LTGD-28,QFL-28,y(3)
b(3)
y(4),LTGDT-28
b(4)
TGDTQG,y0(5),y*(5)
y(6),QFLLTG-28
y(7)
y(9)

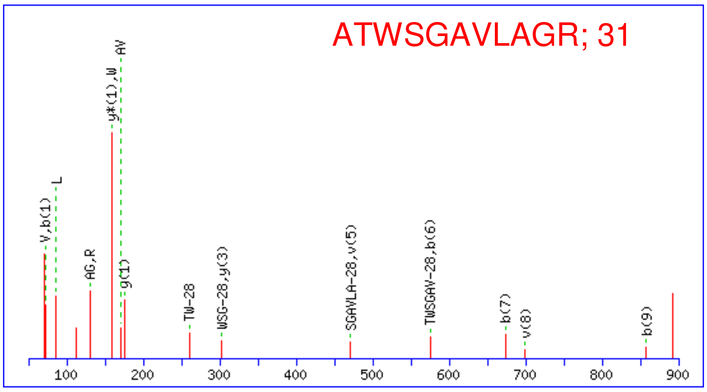
ATWSGAVLAGR; 31
V,b(1)
L
AG,R
y*(1),W
AV
y(1)
TW-28
WSG-28,y(3)
SGAVLA-28,y(5)
TWSGAV-28,b(6)
b(7)
y(8)
b(9)

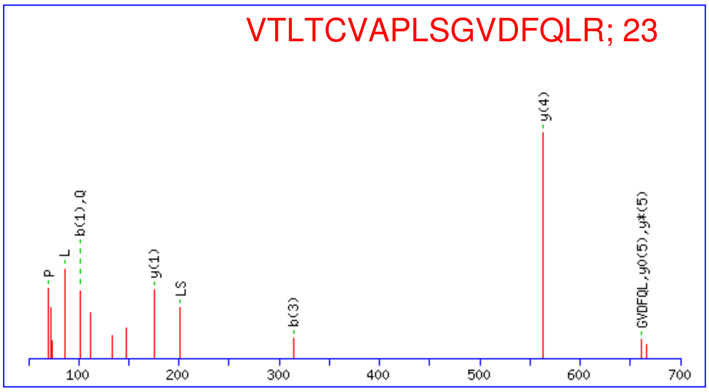
VTLTCVAPLSGVDFQLR; 23
P
L
b(1),Q
y(1)
LS
b(3)
y(4)
GVDFQL,y0(5),y*(5)

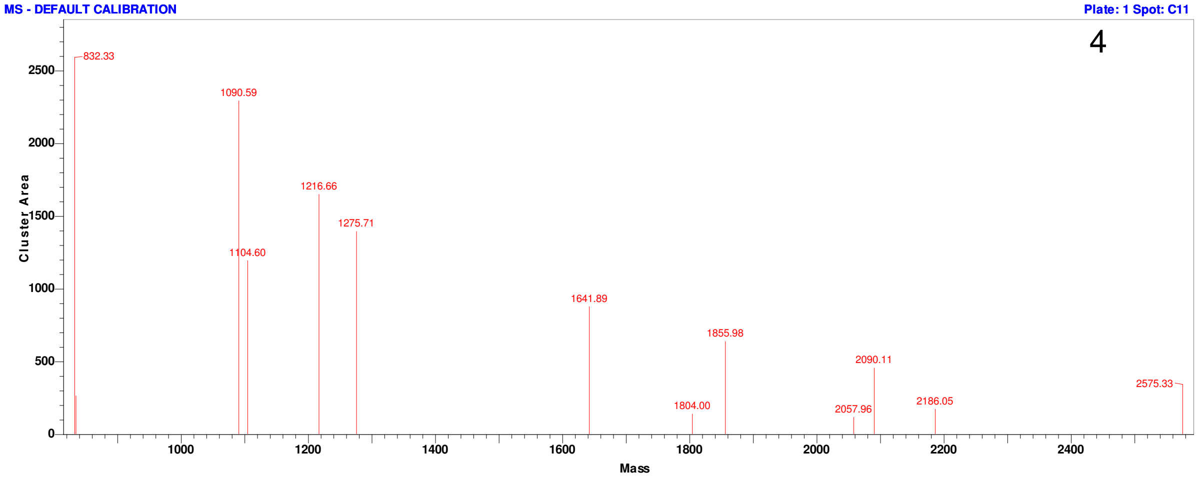

MS - DEFAULT CALIBRATION
Plate: 1 Spot: C11
4
Cluster Area
Mass
832.33
1090.59
1104.60
1216.66
1275.71
1641.89
1804.00
1855.98
2057.96
2090.11
2186.05
2575.33

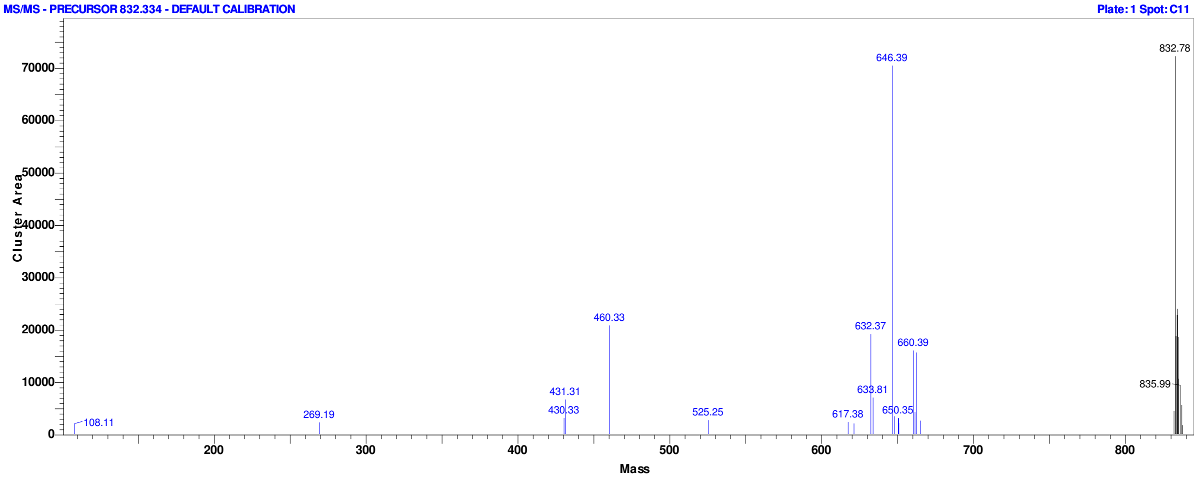

MS/MS - PRECURSOR 832.334 - DEFAULT CALIBRATION
Plate: 1 Spot: C11
Cluster Area
Mass
108.11
269.19
430.33
431.31
460.33
525.25
617.38
632.37
633.81
646.39
650.35
660.39
832.78
835.99

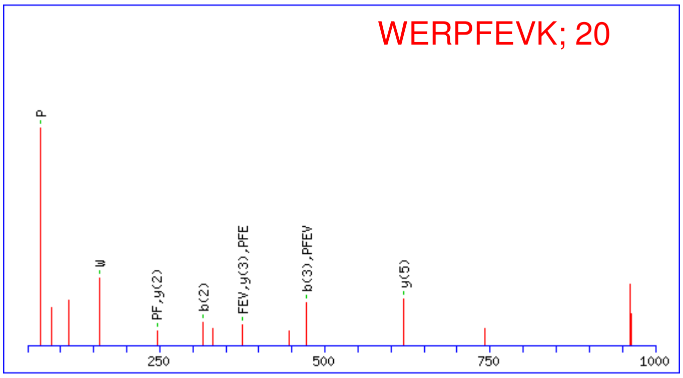

WERPFEVK; 20
P
W
PF,y(2)
b(2)
FEV,y(3),PFE
b(3),PFEV
y(5)

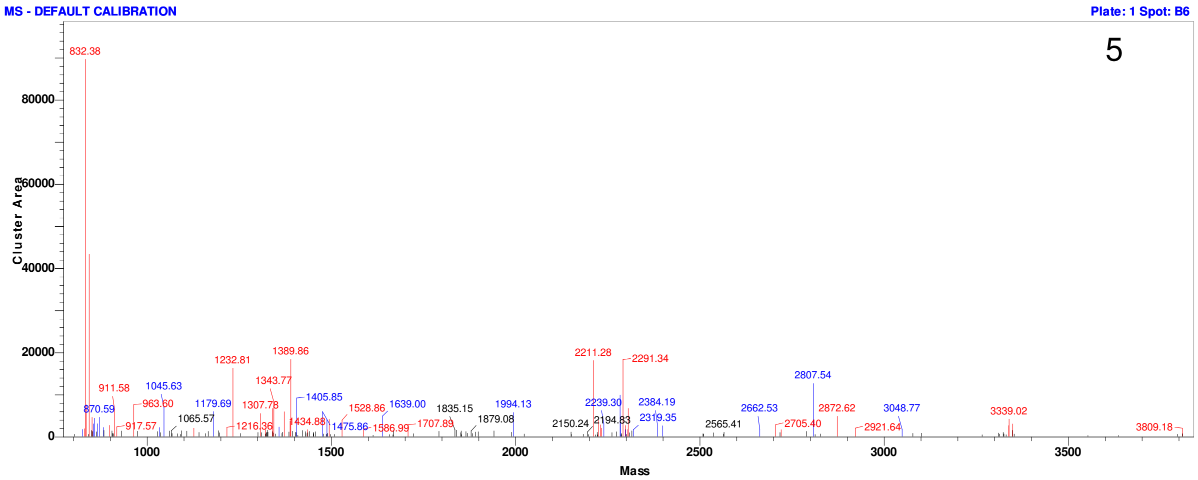
MS - DEFAULT CALIBRATION
Plate: 1 Spot: B6
5
Cluster Area
Mass
832.38
870.59
911.58
917.57
963.60
1045.63
1065.57
1179.69
1216.36
1232.81
1307.78
1343.77
1389.86
1405.85
1434.88
1475.86
1528.86
1586.99
1639.00
1707.89
1835.15
1879.08
1994.13
2150.24
2194.83
2211.28
2239.30
2291.34
2319.35
2384.19
2565.41
2662.53
2705.40
2807.54
2872.62
2921.64
3048.77
3339.02
3809.18

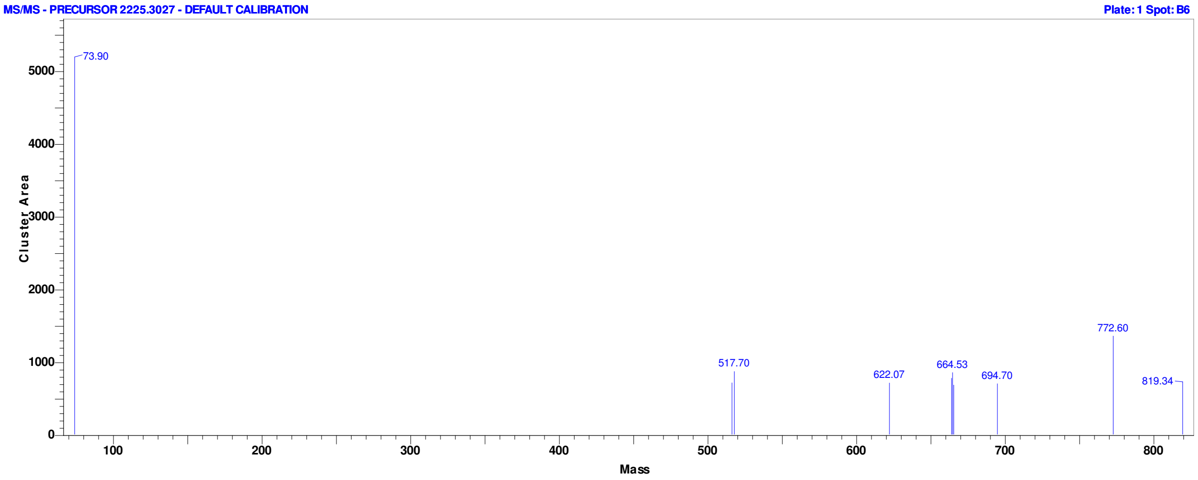
MS/MS - PRECURSOR 2225.3027 - DEFAULT CALIBRATION
Plate: 1 Spot: B6
Cluster Area
Mass
73.90
517.70
622.07
664.53
694.70
772.60
819.34

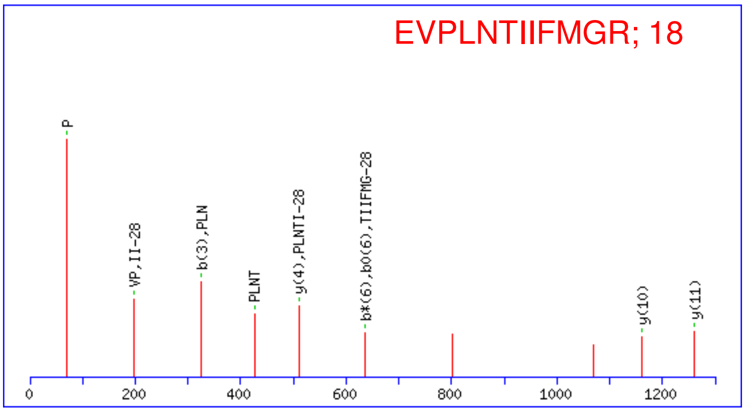
EVPLNTIIFMGR; 18
P
VP,II-28
b(3),PLN
PLNT
y(4),PLNTI-28
b*(6),b0(6),TIIFMG-28
y(10)
y(11)

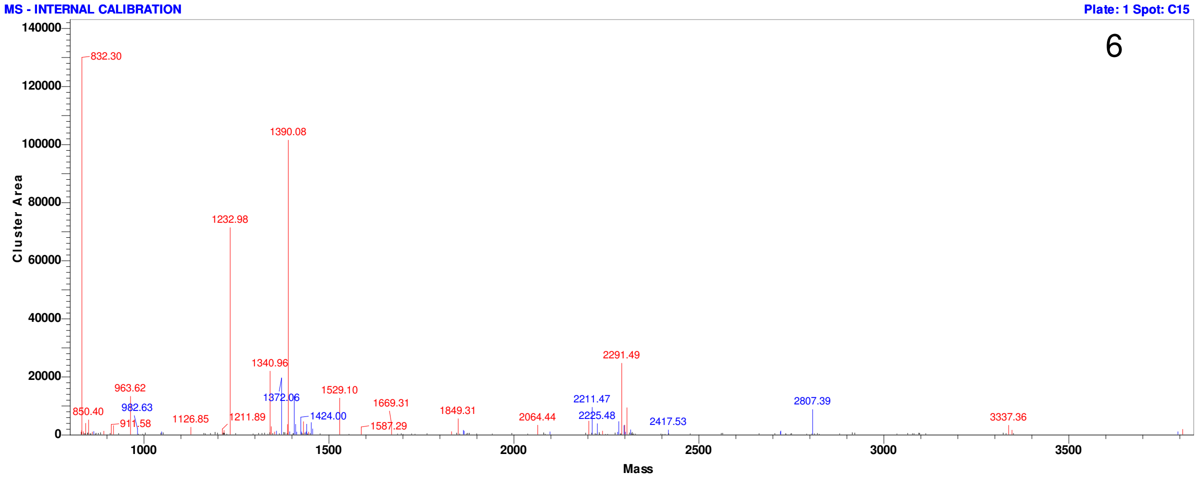
MS - INTERNAL CALIBRATION
Plate: 1 Spot: C15
6
Cluster Area
Mass
832.30
1390.08
1232.98
1340.96
1372.06
963.62
982.63
850.40
911.58
1126.85
1211.89
1424.00
1529.10
1587.29
1669.31
1849.31
2064.44
2211.47
2225.48
2291.49
2417.53
2807.39
3337.36

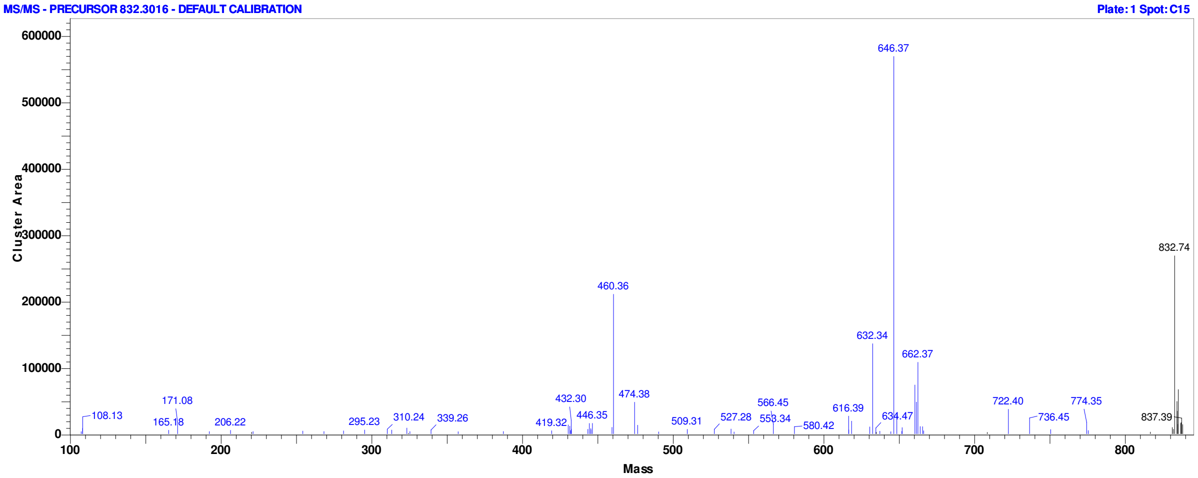
MS/MS - PRECURSOR 832.3016 - DEFAULT CALIBRATION
Plate: 1 Spot: C15
Cluster Area
Mass
646.37
832.74
460.36
632.34
662.37
108.13
171.08
165.18
206.22
295.23
310.24
339.26
419.32
432.30
446.35
474.38
509.31
527.28
553.34
566.45
580.42
616.39
634.47
722.40
736.45
774.35
837.39

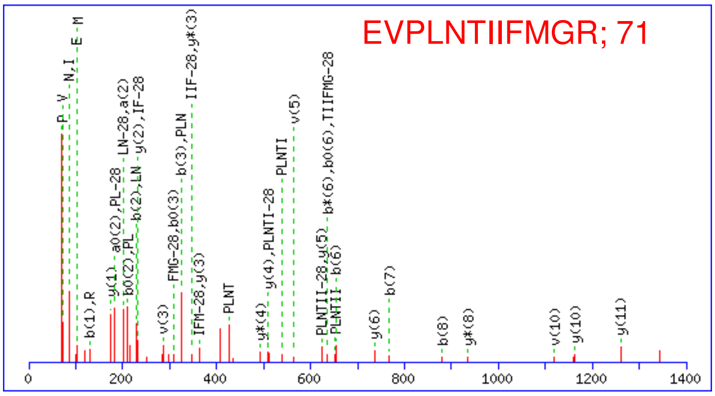
EVPLNTIIFMGR; 71

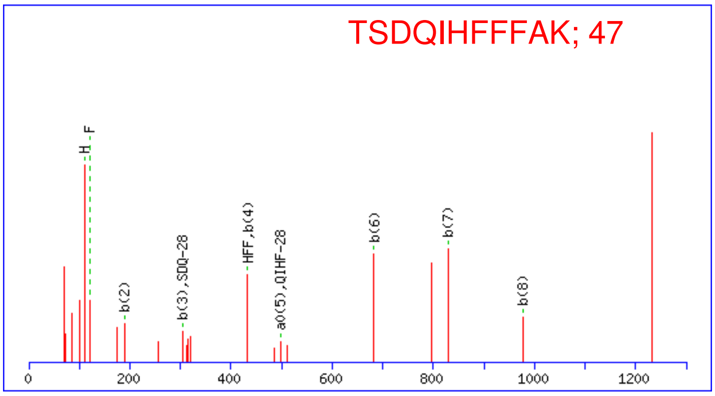
TSDQIHFFFAK; 47

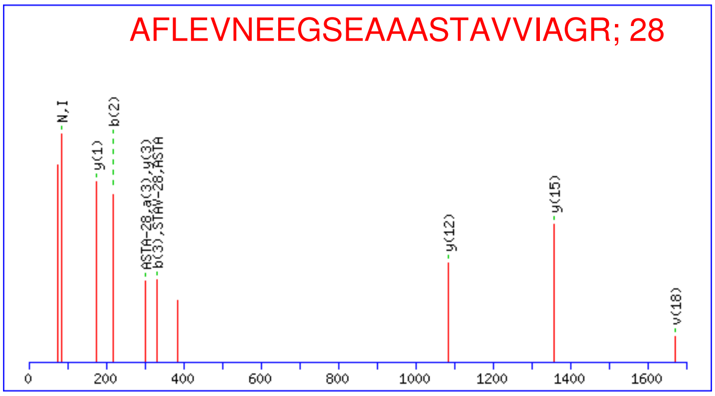
AFLEVNEEGSEAAASTAVVIAGR; 28

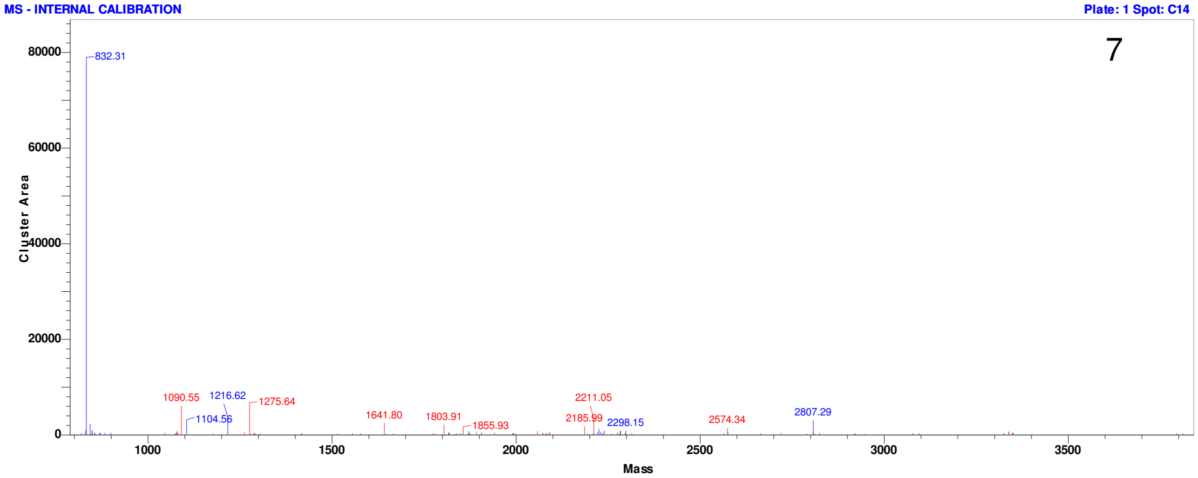
MS - INTERNAL CALIBRATION
Plate: 1 Spot: C14
7
Cluster Area
80000
60000
40000
20000
0
832.31
1090.55
1216.62
1104.56
1275.64
1641.80
1803.91
1855.93
2211.05
2185.99
2298.15
2574.34
2807.29
1000
1500
2000
2500
3000
3500
Mass

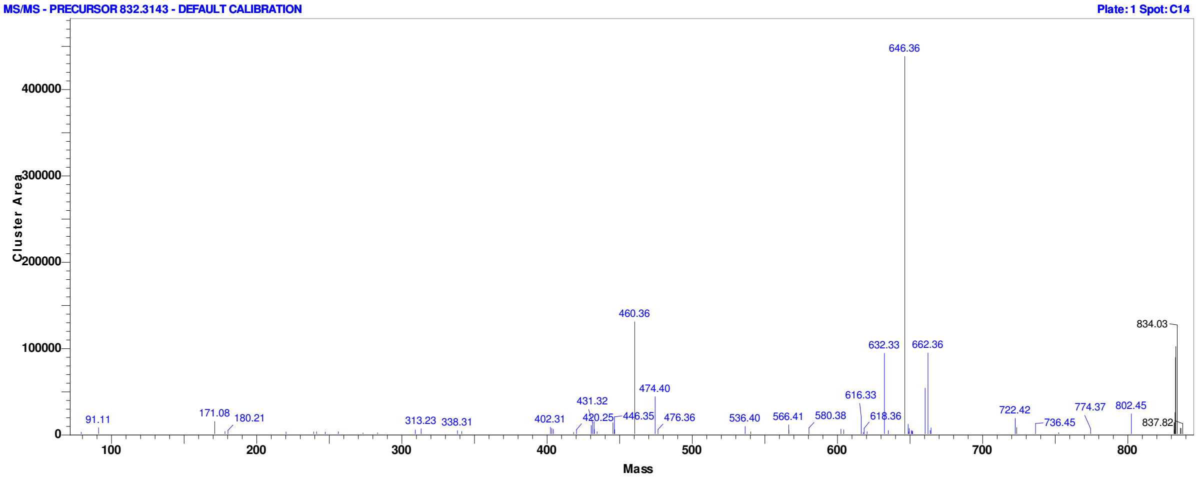
MS/MS - PRECURSOR 832.3143 - DEFAULT CALIBRATION
Plate: 1 Spot: C14
Cluster Area
400000
300000
200000
100000
0
646.36
460.36
834.03
632.33
662.36
474.40
616.33
431.32
91.11
171.08
180.21
313.23
338.31
402.31
420.25
446.35
476.36
536.40
566.41
580.38
618.36
722.42
736.45
774.37
802.45
837.82
100
200
300
400
500
600
700
800
Mass

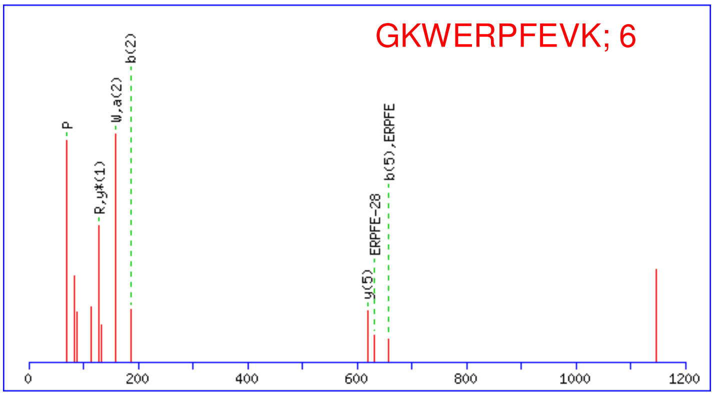
GKWERPFEVK; 6
P
R,y*(1)
W,a(2)
b(2)
y(5)
ERPFE-28
b(5),ERPFE
0
200
400
600
800
1000
1200

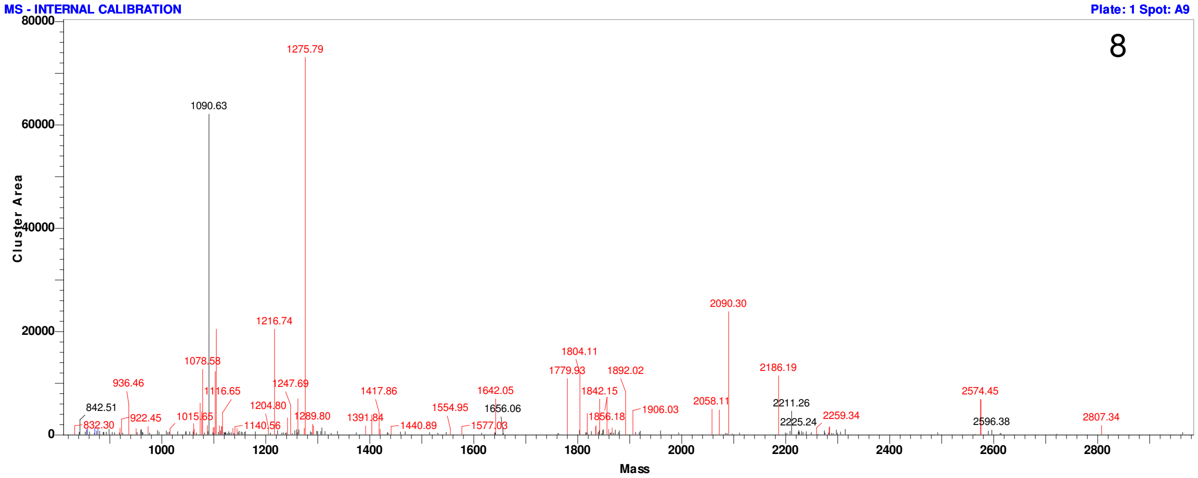
MS - INTERNAL CALIBRATION
Plate: 1 Spot: A9
8
Cluster Area
Mass
1275.79
1090.63
1216.74
1078.58
936.46
842.51
832.30
922.45
1015.65
1116.65
1140.56
1204.80
1247.69
1289.80
1391.84
1417.86
1440.89
1554.95
1577.03
1642.05
1656.06
1779.93
1804.11
1842.15
1856.18
1892.02
1906.03
2058.11
2090.30
2186.19
2211.26
2225.24
2259.34
2574.45
2596.38
2807.34

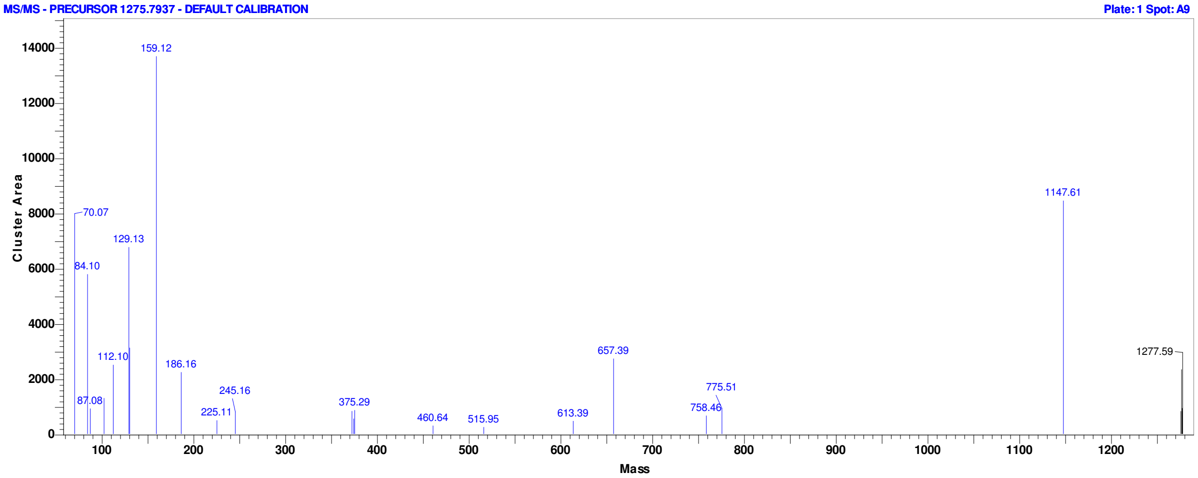
MS/MS - PRECURSOR 1275.7937 - DEFAULT CALIBRATION
Plate: 1 Spot: A9
Cluster Area
Mass
159.12
70.07
84.10
129.13
112.10
87.08
186.16
225.11
245.16
375.29
460.64
515.95
613.39
657.39
758.46
775.51
1147.61
1277.59

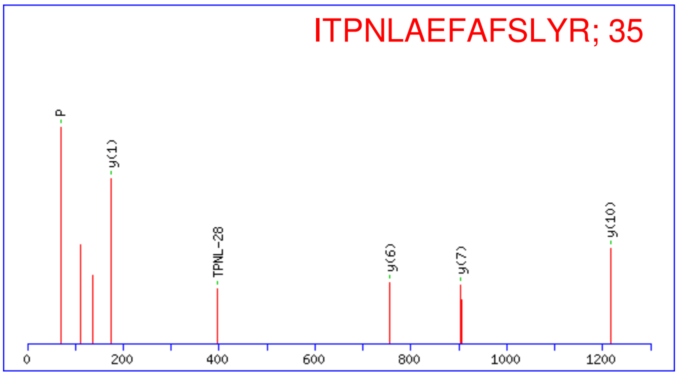
ITPNLAEFAFSLYR; 35
P
y(1)
TPNL-28
y(6)
y(7)
y(10)

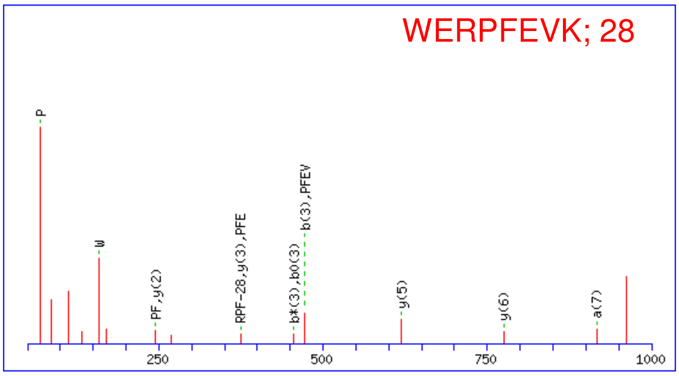
WERPFEVK; 28
P
W
PF,y(2)
RPF-28,y(3),PFE
b*(3),b0(3)
b(3),PFEV
y(5)
y(6)
a(7)

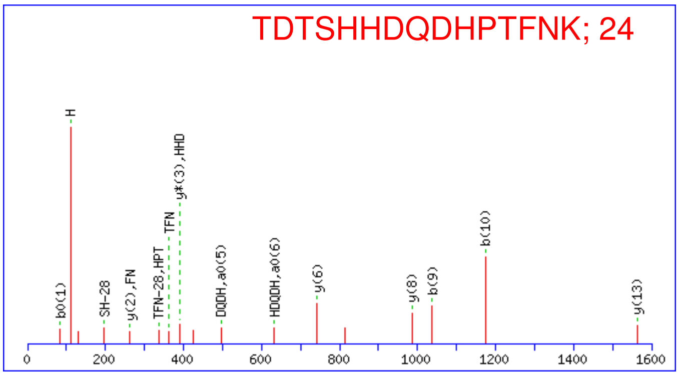
TDTSHHDQDHPTFNK; 24
b0(1)
H
SH-28
y(2),FN
TFN-28,HPT
TFN
y*(3),HHD
DQDH,a0(5)
HDQDH,a0(6)
y(6)
y(8)
b(9)
b(10)
y(13)

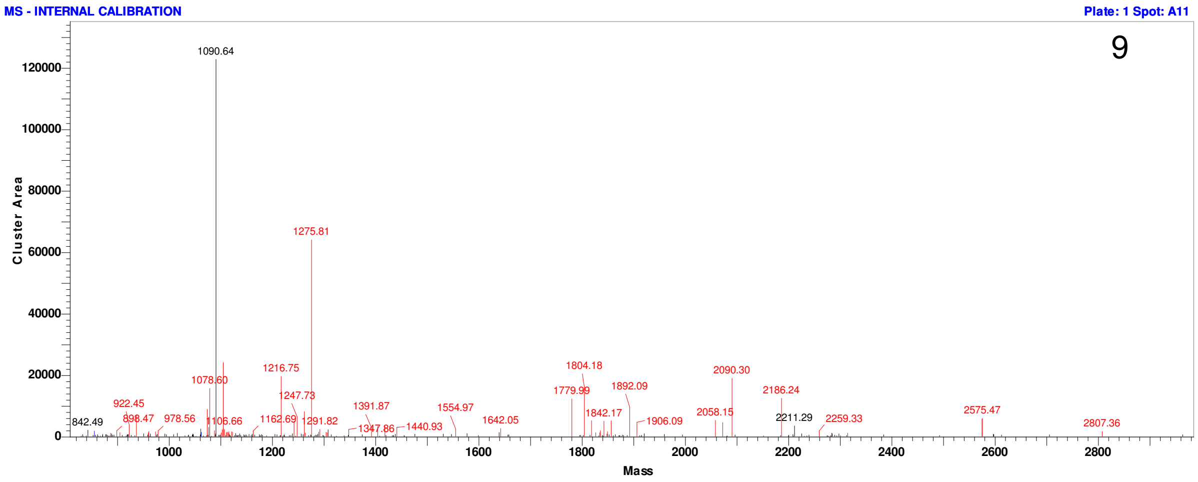
MS - INTERNAL CALIBRATION
Plate: 1 Spot: A11
9
Cluster Area
Mass
1090.64
1275.81
1078.60
1216.75
1247.73
922.45
898.47
978.56
842.49
1106.66
1162.69
1291.82
1391.87
1347.86
1440.93
1554.97
1642.05
1804.18
1779.99
1842.17
1892.09
1906.09
2058.15
2090.30
2186.24
2211.29
2259.33
2575.47
2807.36

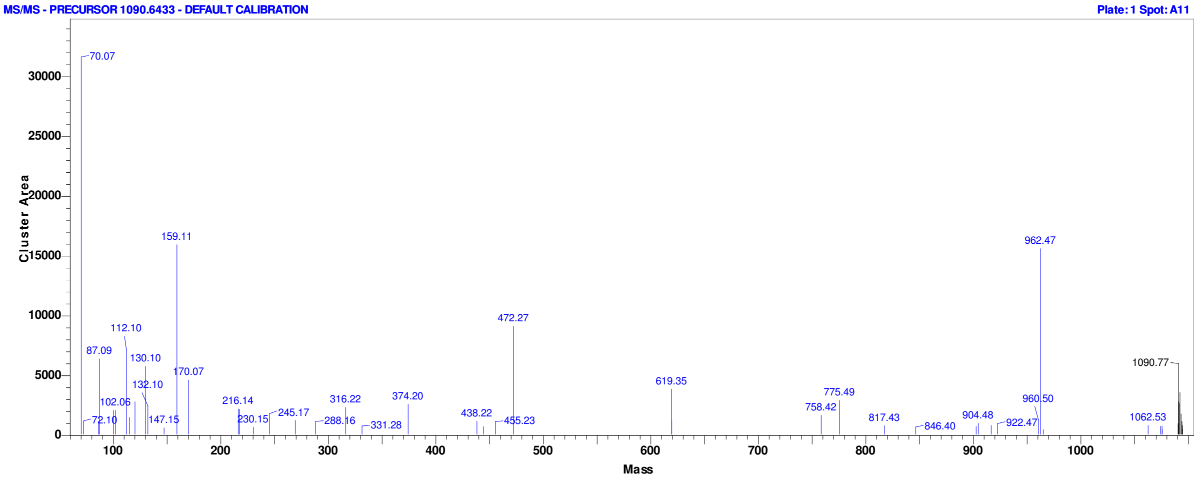
MS/MS - PRECURSOR 1090.6433 - DEFAULT CALIBRATION
Plate: 1 Spot: A11
Cluster Area
Mass
70.07
159.11
962.47
472.27
112.10
87.09
130.10
132.10
170.07
102.06
72.10
147.15
216.14
230.15
245.17
316.22
288.16
331.28
374.20
438.22
455.23
619.35
775.49
758.42
817.43
846.40
904.48
922.47
960.50
1062.53
1090.77

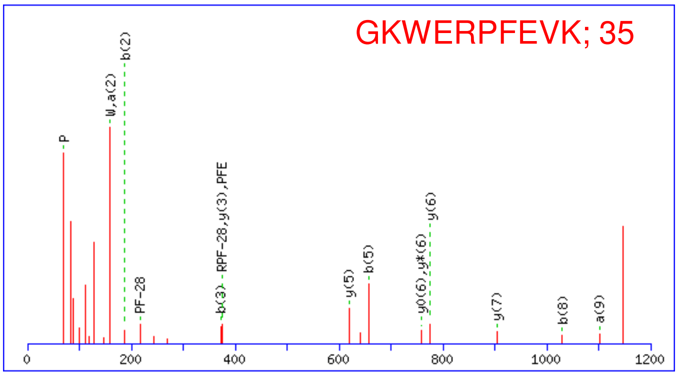
GKWERPFEVK; 35
P
W,a(2)
b(2)
PF-28
b(3),RPF-28,y(3),PFE
y(5)
b(5)
y0(6),y*(6)
y(6)
y(7)
b(8)
a(9)

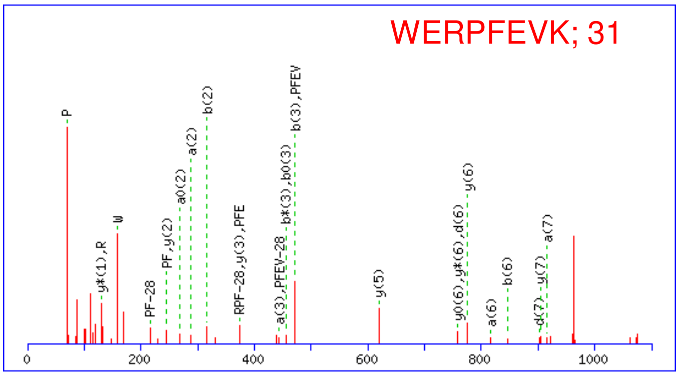
WERPFEVK; 31
P
y*(1),R
W
PF-28
PF,y(2)
a0(2)
a(2)
b(2)
RPF-28,y(3),PFE
a(3),PFEV-28
b*(3),b0(3)
b(3),PFEV
y(5)
y0(6),y*(6),d(6)
y(6)
a(6)
b(6)
d(7)
y(7)
a(7)

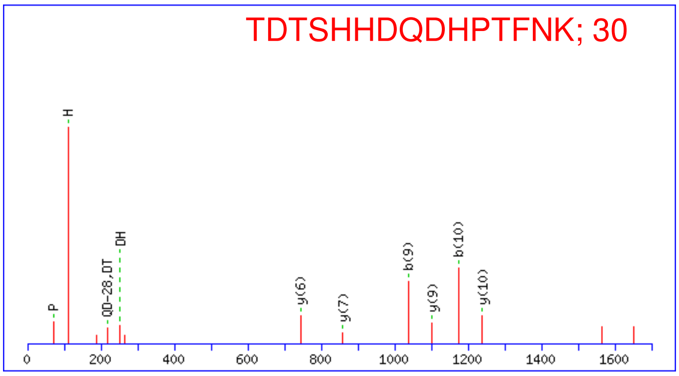
TDTSHHDQDHPTFNK; 30
P
H
QD-28,DT
DH
y(6)
y(7)
b(9)
y(9)
b(10)
y(10)

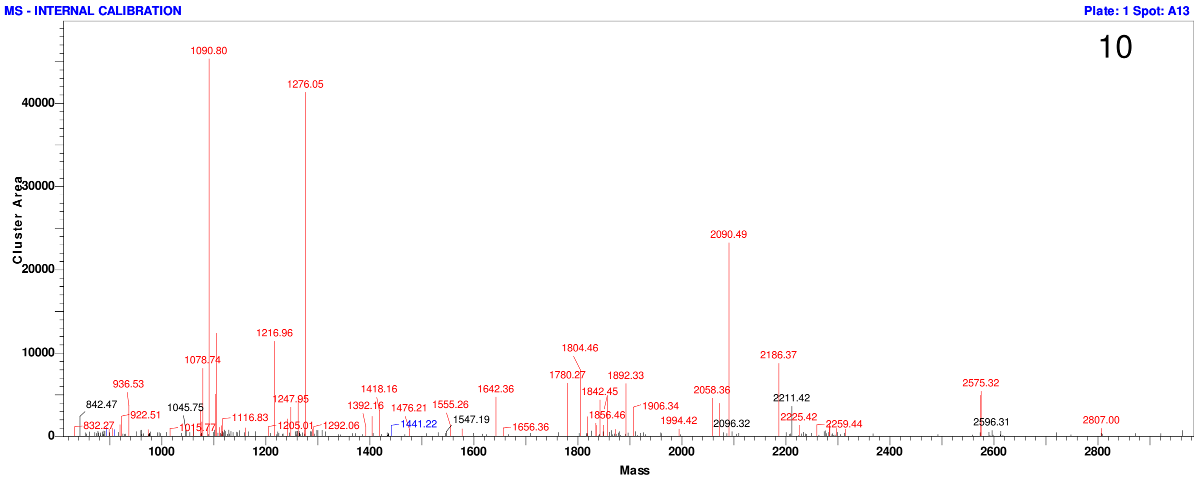
MS - INTERNAL CALIBRATION
Plate: 1 Spot: A13
10
Cluster Area
Mass
1090.80
1276.05
2090.49
1216.96
1078.74
1804.46
2186.37
1780.27
1892.33
936.53
1418.16
1642.36
2058.36
2575.32
842.47
1045.75
1247.95
1842.45
2211.42
922.51
1392.16
1476.21
1555.26
1906.34
1116.83
1856.46
2225.42
832.27
1015.77
1205.01
1292.06
1441.22
1547.19
1656.36
1994.42
2096.32
2259.44
2596.31
2807.00

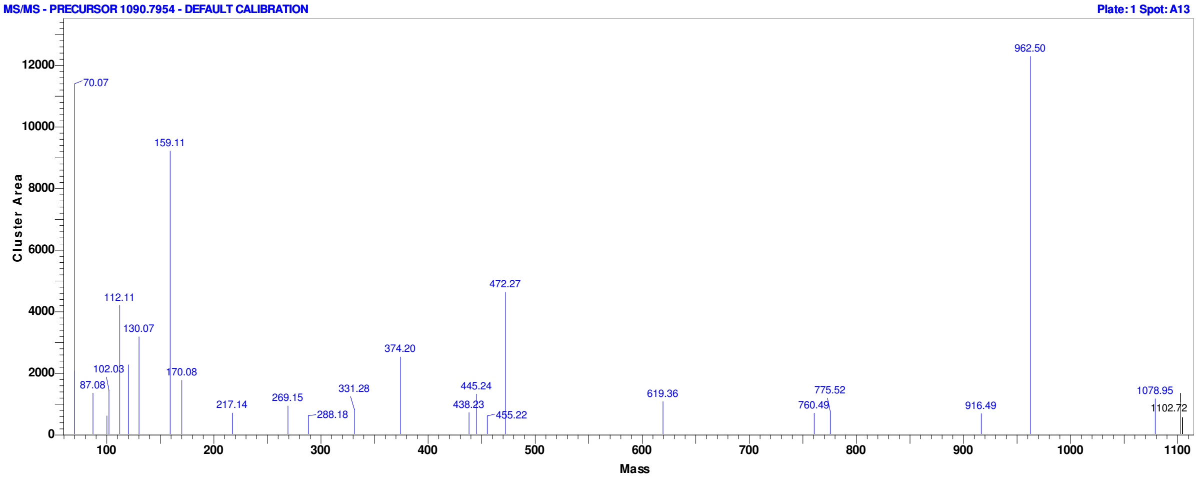
MS/MS - PRECURSOR 1090.7954 - DEFAULT CALIBRATION
Plate: 1 Spot: A13
Cluster Area
Mass
962.50
70.07
159.11
112.11
130.07
472.27
374.20
102.03
87.08
170.08
217.14
269.15
288.18
331.28
445.24
438.23
455.22
619.36
775.52
760.49
916.49
1078.95
1102.72

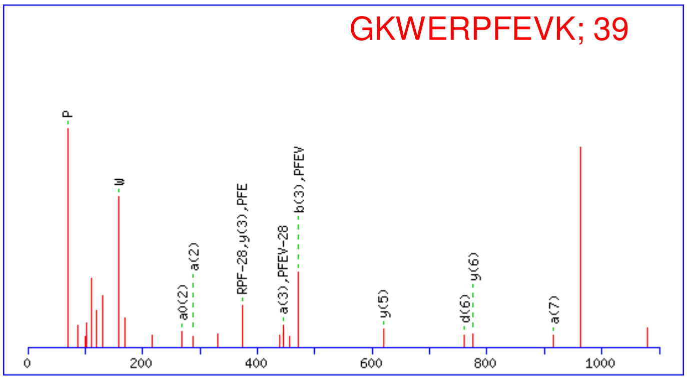
GKWERPFEVK; 39
P
W
a0(2)
a(2)
RPF-28,y(3),PFE
a(3),PFEV-28
b(3),PFEV
y(5)
d(6)
y(6)
a(7)

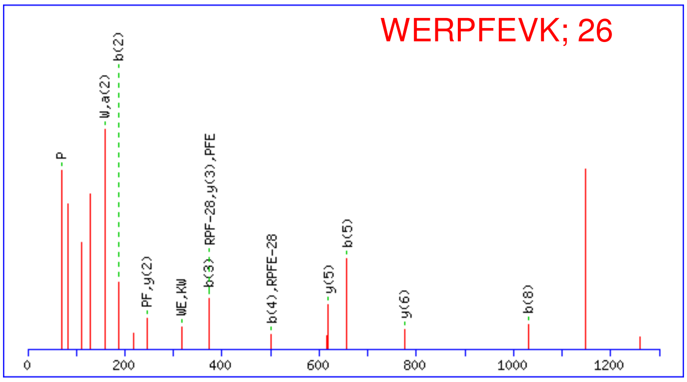
WERPFEVK; 26
P
W,a(2)
b(2)
PF,y(2)
WE,KW
b(3)
RPF-28,y(3),PFE
b(4),RPFE-28
y(5)
b(5)
y(6)
b(8)

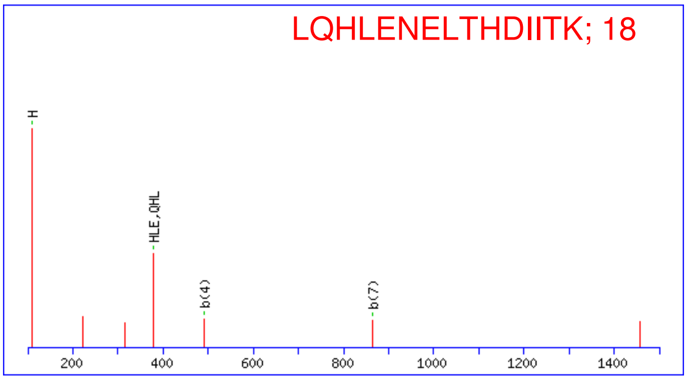
LQHLENELTHDIITK; 18
H
HLE,QHL
b(4)
b(7)

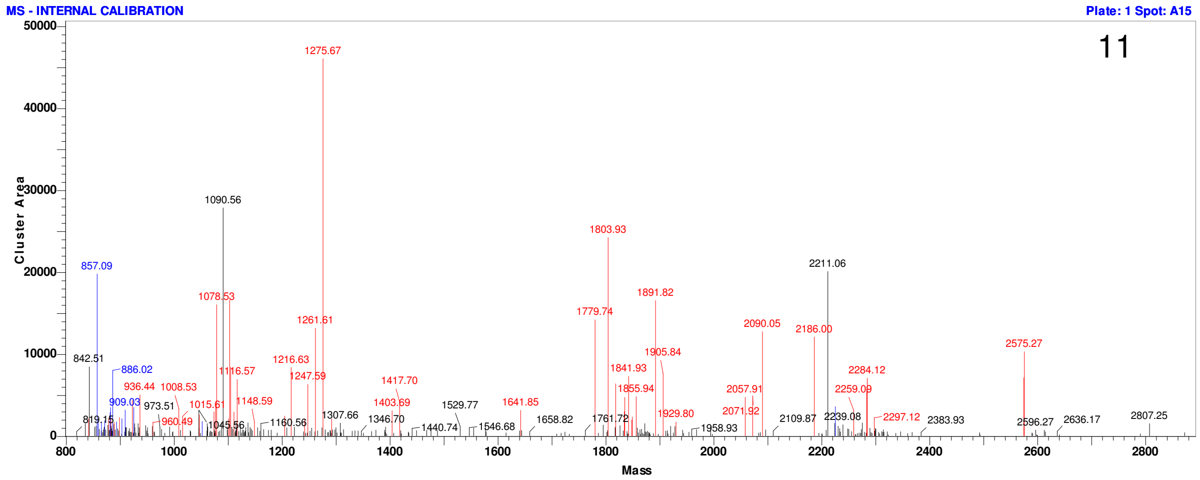
MS - INTERNAL CALIBRATION
Plate: 1 Spot: A15
11
Cluster Area
Mass
1275.67
1090.56
1803.93
857.09
2211.06
1891.82
1078.53
1779.74
1261.61
2090.05
2186.00
2575.27
842.51
1905.84
1216.63
886.02
1116.57
1841.93
2284.12
1247.59
1417.70
936.44
1008.53
2057.91
1855.94
2259.09
909.03
1403.69
1148.59
1641.85
973.51
1015.61
1529.77
2071.92
1929.80
1307.66
2109.87
2239.08
2297.12
2807.25
819.15
960.49
1346.70
1658.82
1761.72
2383.93
2596.27
2636.17
1045.56
1160.56
1440.74
1546.68
1958.93

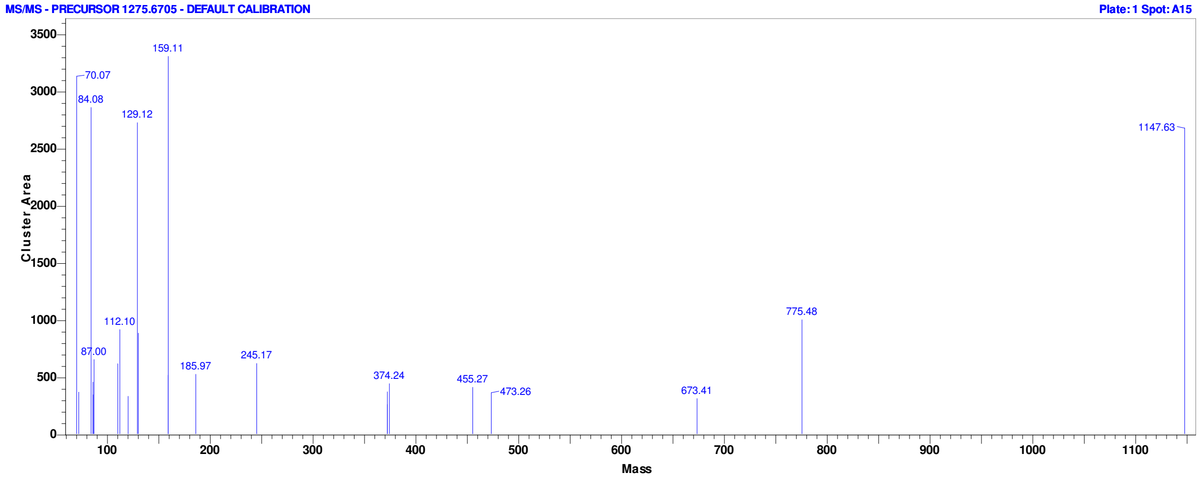
MS/MS - PRECURSOR 1275.6705 - DEFAULT CALIBRATION
Plate: 1 Spot: A15
Cluster Area
Mass
159.11
70.07
84.08
129.12
1147.63
775.48
112.10
87.00
245.17
185.97
374.24
455.27
473.26
673.41

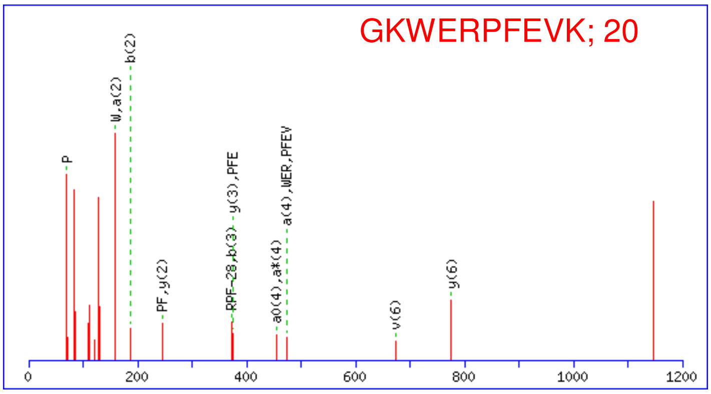
GKWERPFEVK; 20
P
W,a(2)
b(2)
PF,y(2)
RPF-28,b(3),y(3),PFE
a0(4),a*(4)
a(4),WER,PFEV
y(6)
y(6)

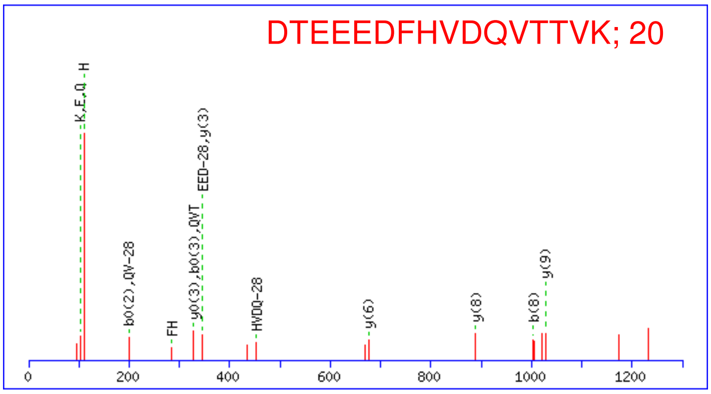
DTEEEDFHVDQVTTVK; 20
K,E,Q,H
b0(2),QV-28
FH
y0(3),b0(3),QVT
EED-28,y(3)
HVDQ-28
y(6)
y(8)
b(8)
y(9)

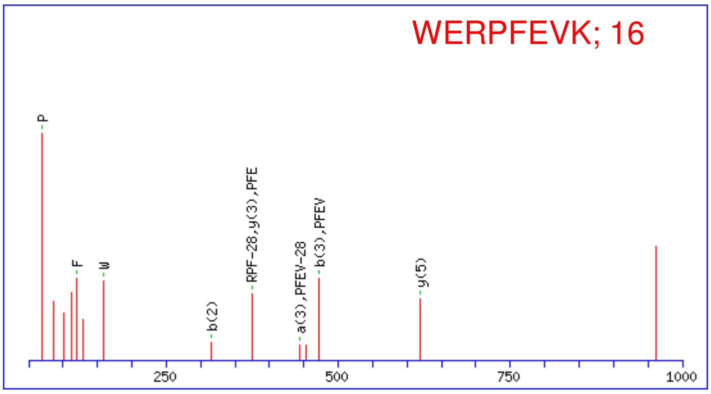
WERPFEVK; 16
P
F
W
b(2)
RPF-28,y(3),PFE
a(3),PFEV-28
b(3),PFEV
y(5)

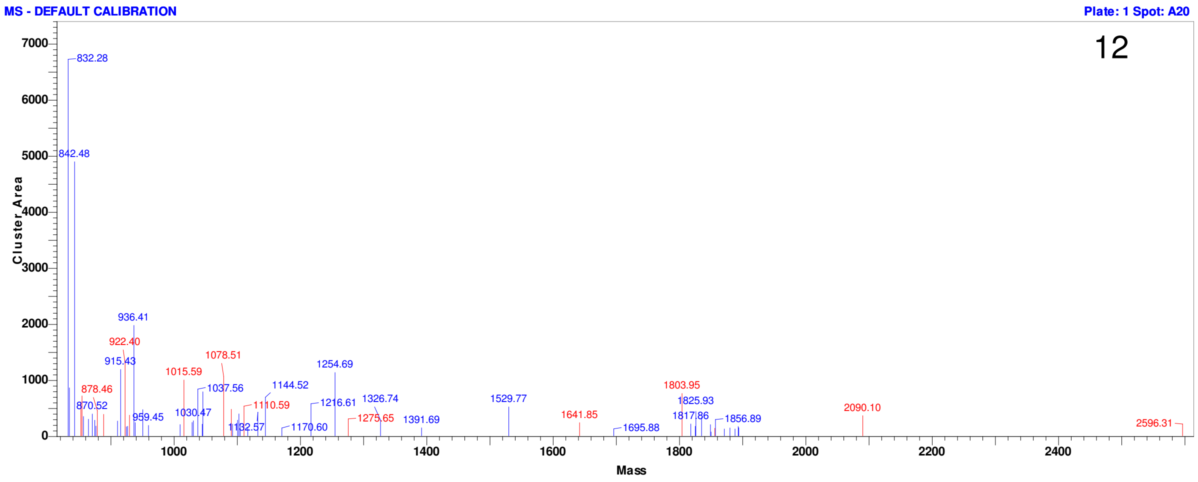
MS - DEFAULT CALIBRATION
Plate: 1 Spot: A20
12
Cluster Area
Mass
832.28
842.48
936.41
922.40
915.43
878.46
870.52
959.45
1015.59
1078.51
1030.47
1037.56
1132.57
1110.59
1144.52
1170.60
1216.61
1254.69
1275.65
1326.74
1391.69
1529.77
1641.85
1695.88
1803.95
1825.93
1817.86
1856.89
2090.10
2596.31

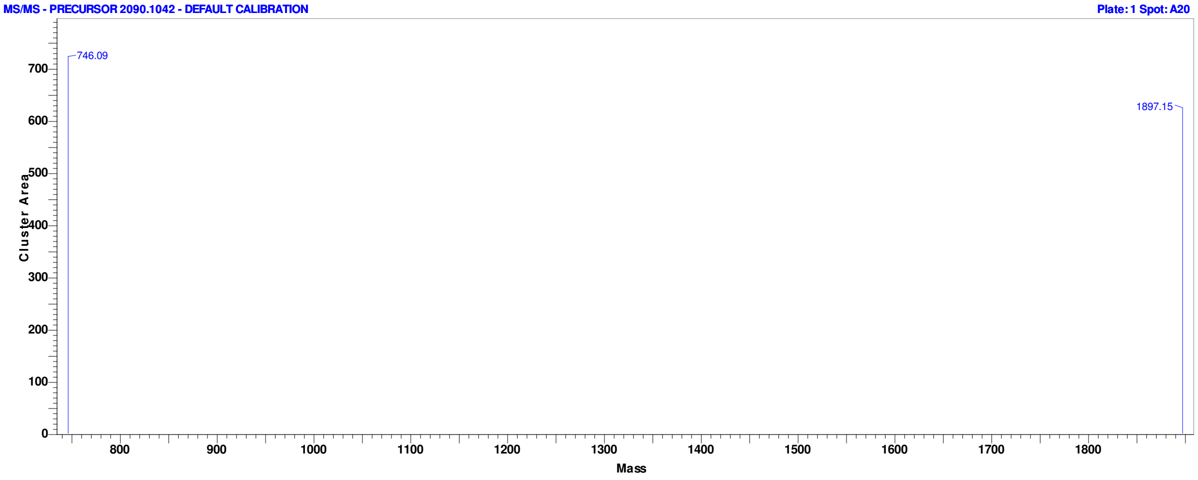
MS/MS - PRECURSOR 2090.1042 - DEFAULT CALIBRATION
Plate: 1 Spot: A20
Cluster Area
Mass
746.09
1897.15

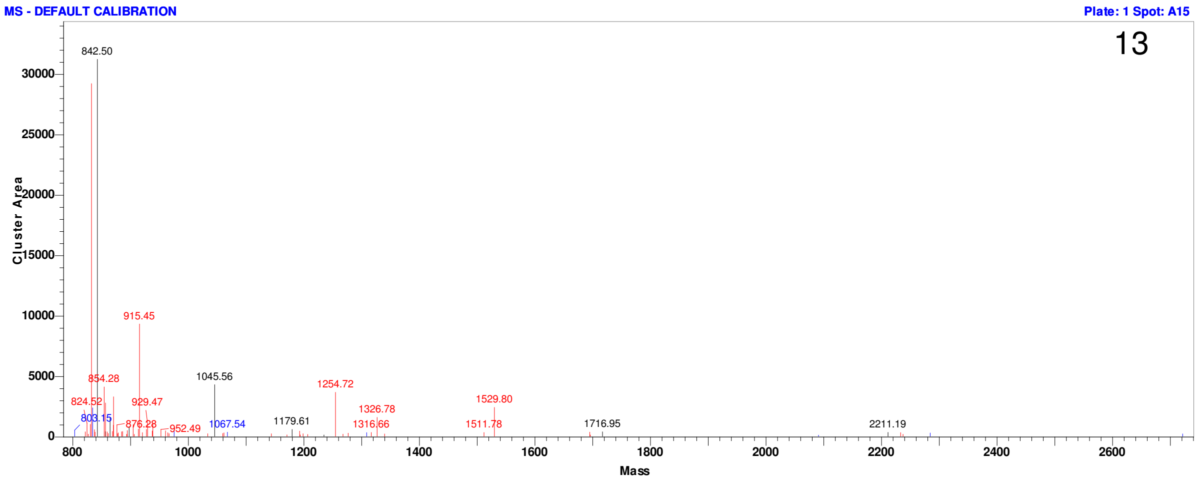
MS - DEFAULT CALIBRATION
Plate: 1 Spot: A15
13
Cluster Area
Mass
842.50
915.45
854.28
824.52
929.47
803.15
876.28
952.49
1045.56
1067.54
1179.61
1254.72
1326.78
1316.66
1529.80
1511.78
1716.95
2211.19

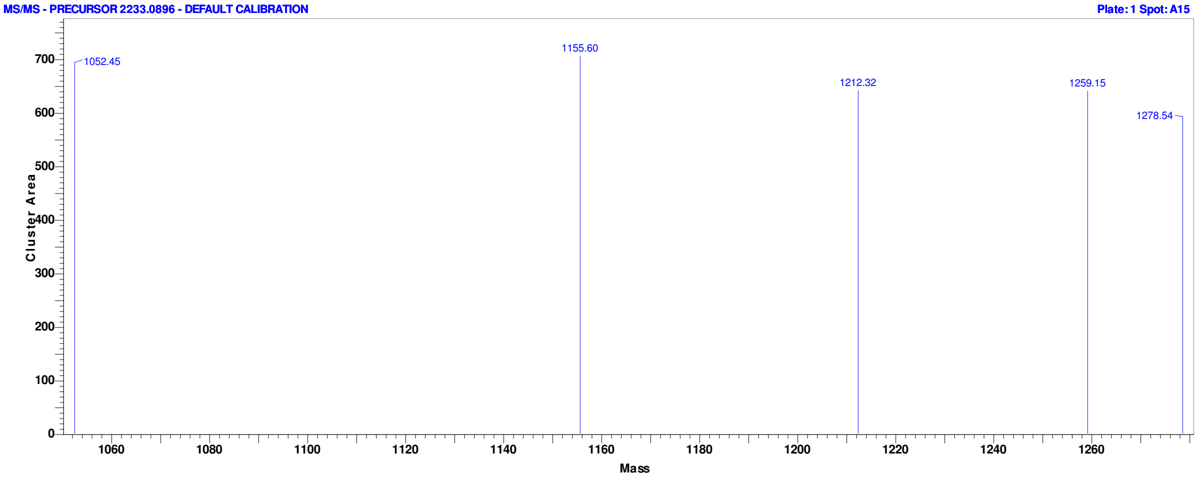
MS/MS - PRECURSOR 2233.0896 - DEFAULT CALIBRATION
Plate: 1 Spot: A15
Cluster Area
Mass
1052.45
1155.60
1212.32
1259.15
1278.54

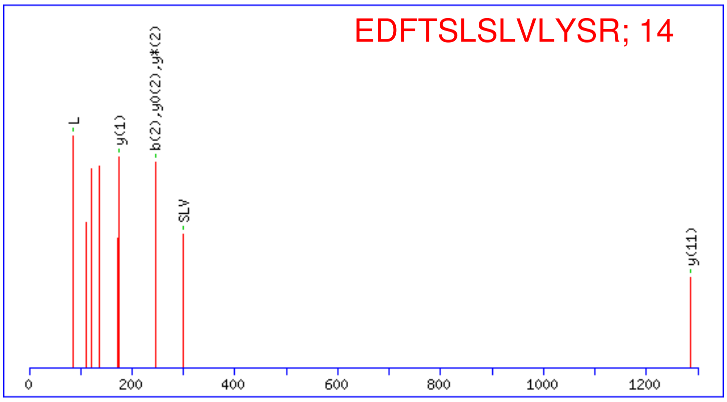
EDFTSLSLVLYSR; 14
L
y(1)
b(2),y0(2),y*(2)
SLV
y(11)

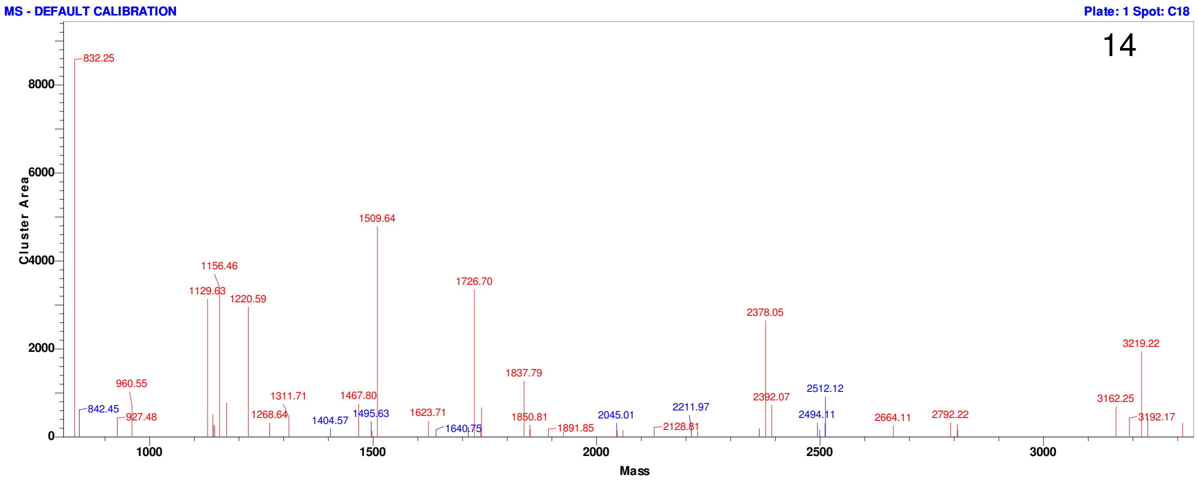
MS - DEFAULT CALIBRATION
Plate: 1 Spot: C18
14
Cluster Area
Mass
832.25
842.45
927.48
960.55
1129.63
1156.46
1220.59
1268.64
1311.71
1404.57
1467.80
1495.63
1509.64
1623.71
1640.75
1726.70
1837.79
1850.81
1891.85
2045.01
2128.81
2211.97
2378.05
2392.07
2494.11
2512.12
2664.11
2792.22
3162.25
3192.17
3219.22

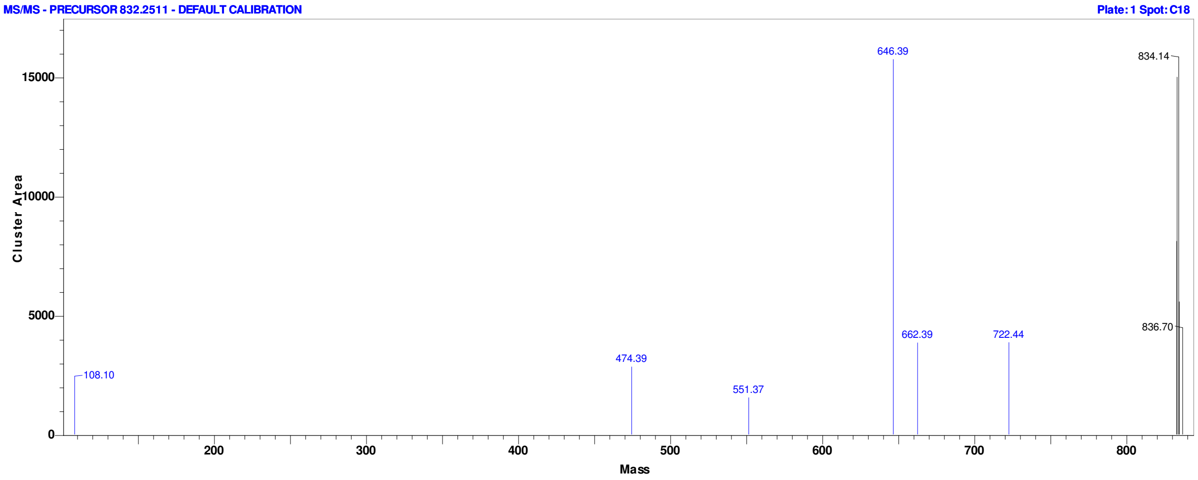
MS/MS - PRECURSOR 832.2511 - DEFAULT CALIBRATION
Plate: 1 Spot: C18
Cluster Area
Mass
108.10
474.39
551.37
646.39
662.39
722.44
834.14
836.70

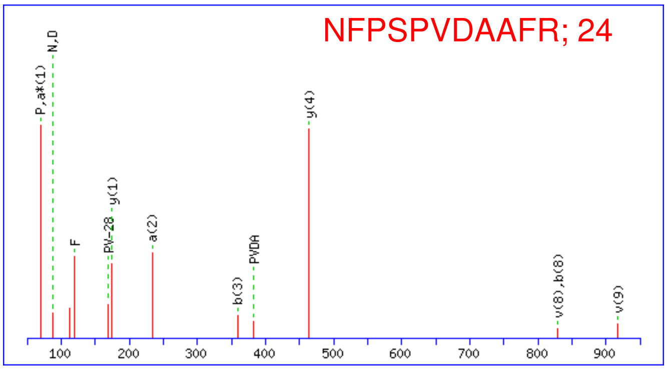
NFPSPVDAAFR; 24
P,a*(1)
N,D
F
PV-28
y(1)
a(2)
b(3)
PVDA
y(4)
y(8),b(8)
y(9)

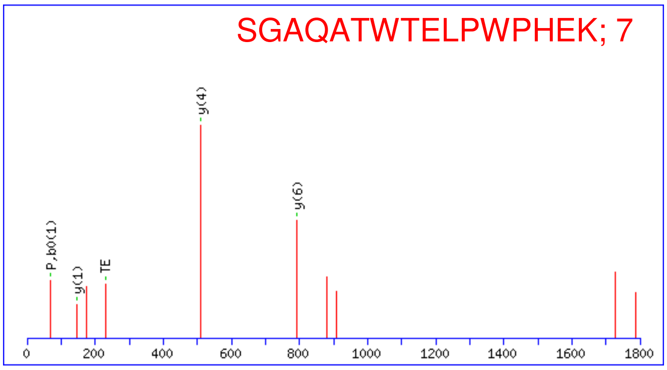
SGAQATWTELPWPHEK; 7
P,b0(1)
y(1)
TE
y(4)
y(6)

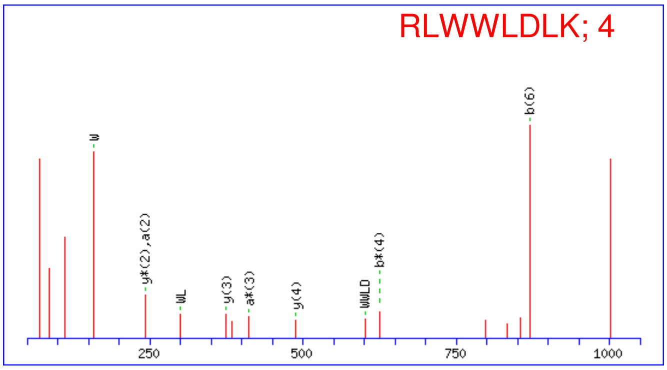
RLWWLDLK; 4
W
y*(2),a(2)
WL
y(3)
a*(3)
y(4)
WWLD
b*(4)
b(6)

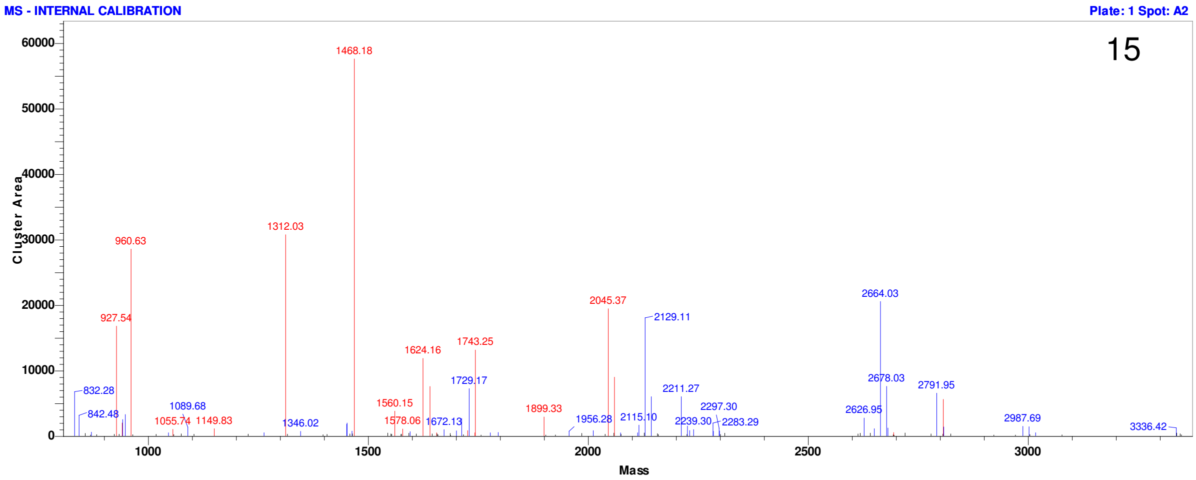
MS - INTERNAL CALIBRATION
Plate: 1 Spot: A2
15
Cluster Area
Mass
1468.18
1312.03
960.63
927.54
2045.37
2129.11
2664.03
1743.25
1624.16
1729.17
2678.03
2791.95
832.28
842.48
1089.68
1055.74
1149.83
1346.02
1560.15
1578.06
1672.13
1899.33
1956.28
2115.10
2211.27
2239.30
2297.30
2283.29
2626.95
2987.69
3336.42

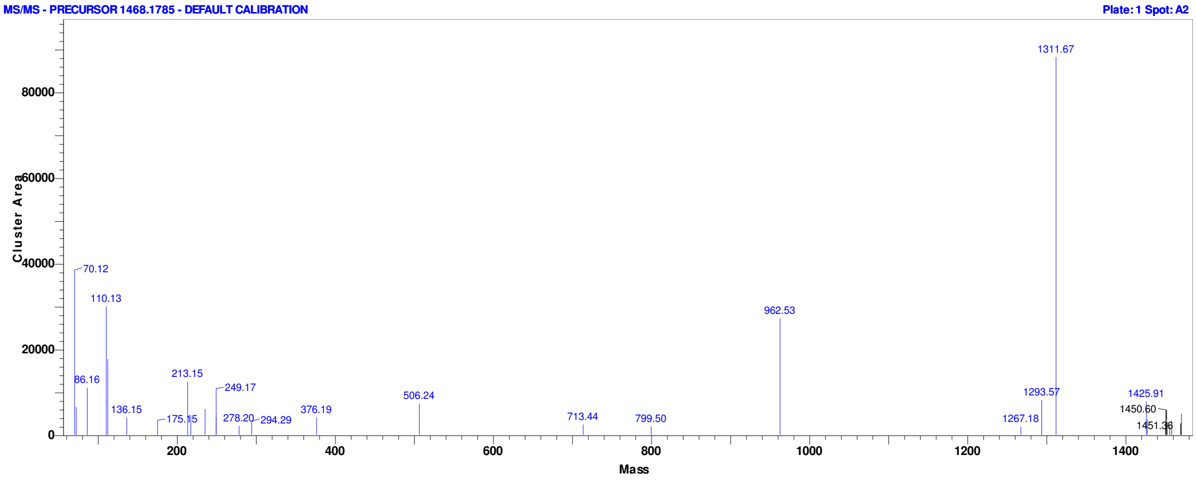
MS/MS - PRECURSOR 1468.1785 - DEFAULT CALIBRATION
Plate: 1 Spot: A2
Cluster Area
Mass
1311.67
70.12
110.13
962.53
86.16
213.15
249.17
136.15
175.15
278.20
294.29
376.19
506.24
713.44
799.50
1267.18
1293.57
1425.91
1450.60
1451.36

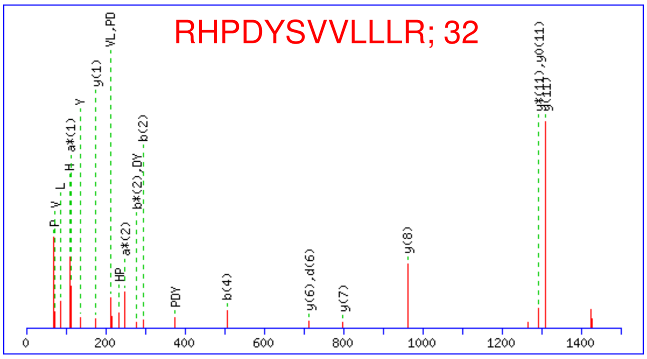
RHPDYSVVLLLR; 32

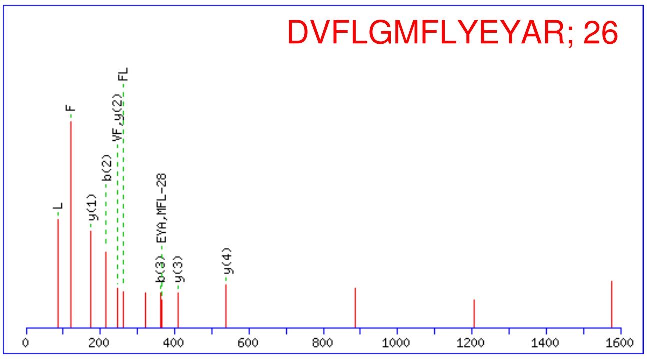
DVFLGMFLYEYAR; 26

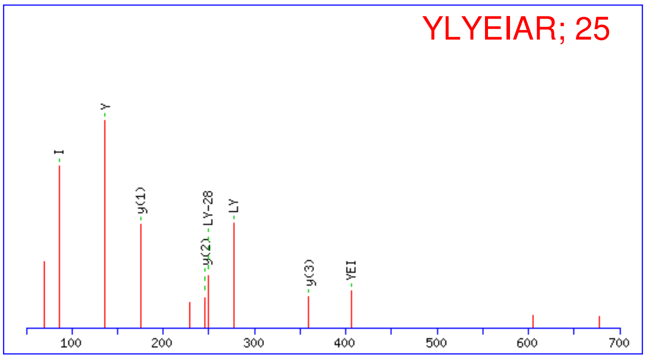
YLYEIAR; 25

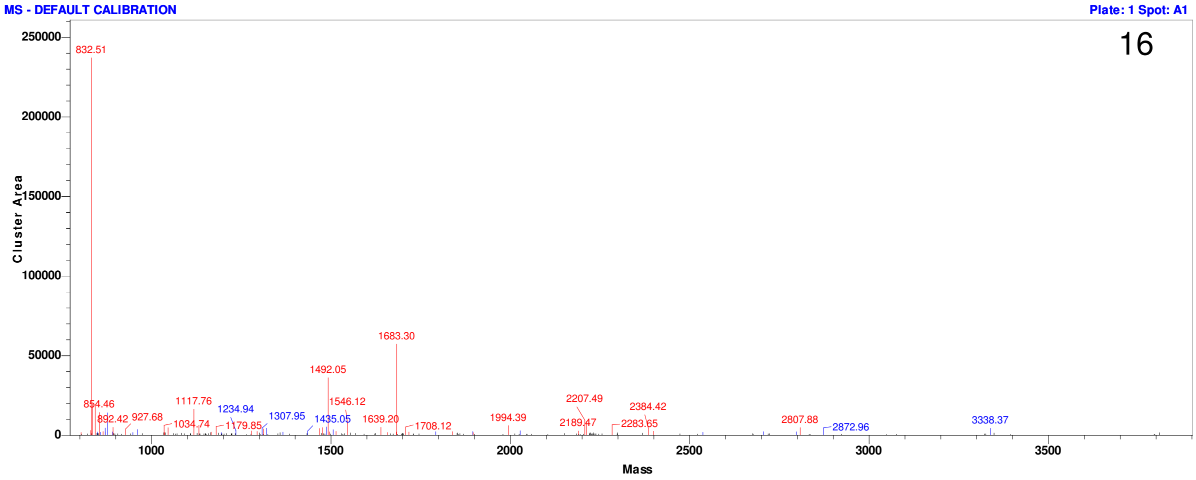
MS - DEFAULT CALIBRATION
Plate: 1 Spot: A1
16
Cluster Area
Mass
832.51
854.46
892.42
927.68
1034.74
1117.76
1179.85
1234.94
1307.95
1435.05
1492.05
1546.12
1639.20
1683.30
1708.12
1994.39
2189.47
2207.49
2283.65
2384.42
2807.88
2872.96
3338.37

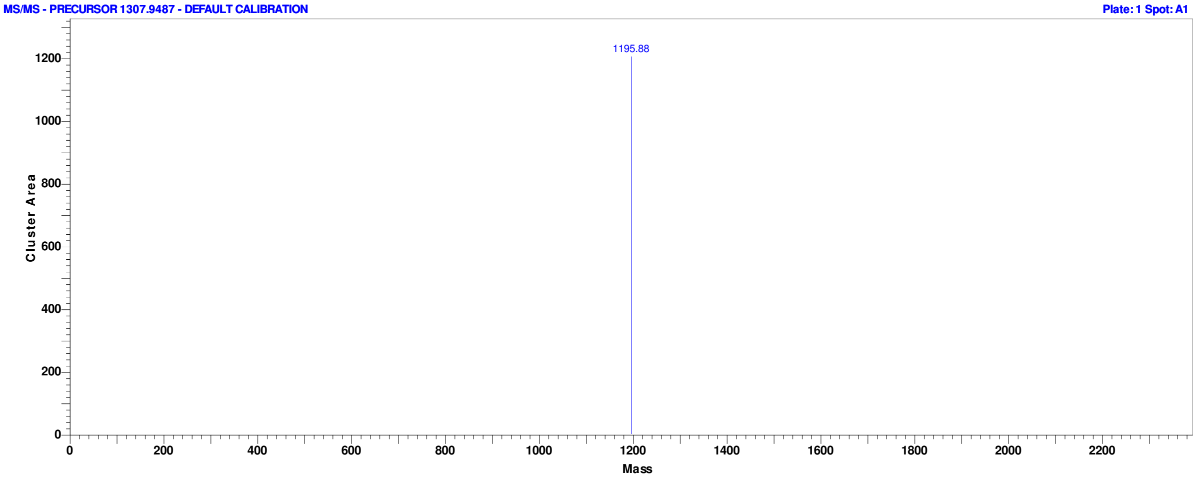
MS/MS - PRECURSOR 1307.9487 - DEFAULT CALIBRATION
Plate: 1 Spot: A1
Cluster Area
Mass
1195.88

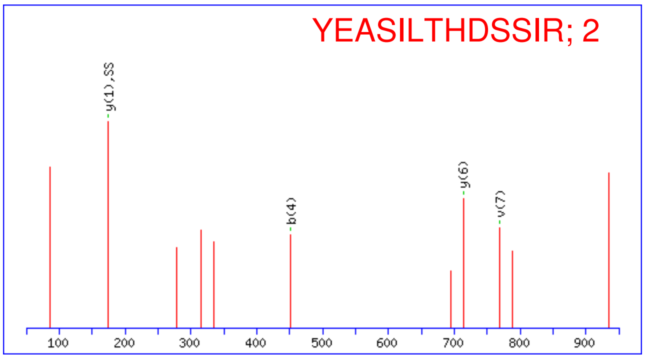
YEASILTHDSSIR; 2
y(1),SS
b(4)
y(6)
y(7)

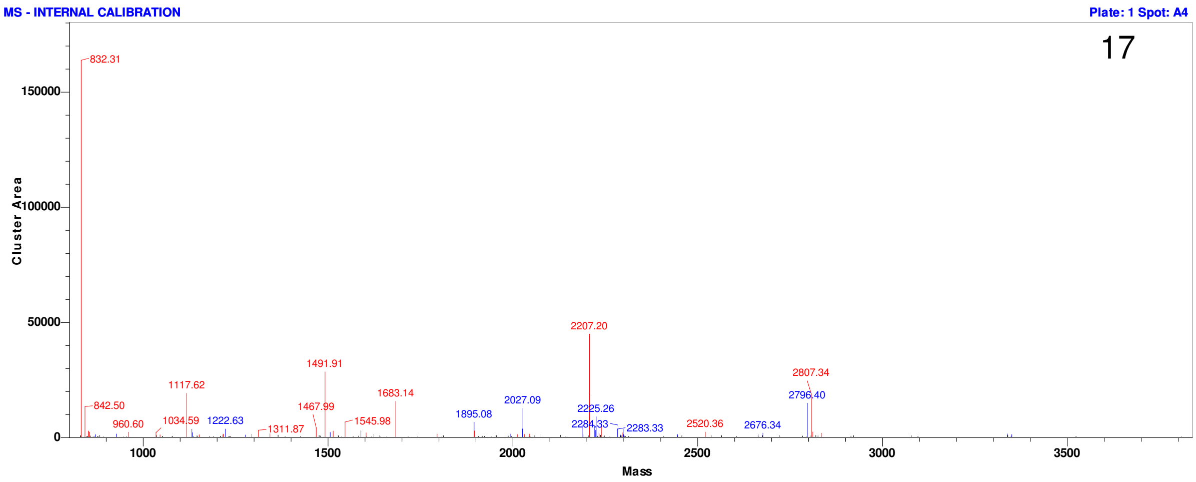
MS - INTERNAL CALIBRATION
Plate: 1 Spot: A4
17
Cluster Area
Mass
832.31
842.50
960.60
1034.59
1117.62
1222.63
1311.87
1467.99
1491.91
1545.98
1683.14
1895.08
2027.09
2207.20
2225.26
2284.33
2283.33
2520.36
2676.34
2796.40
2807.34

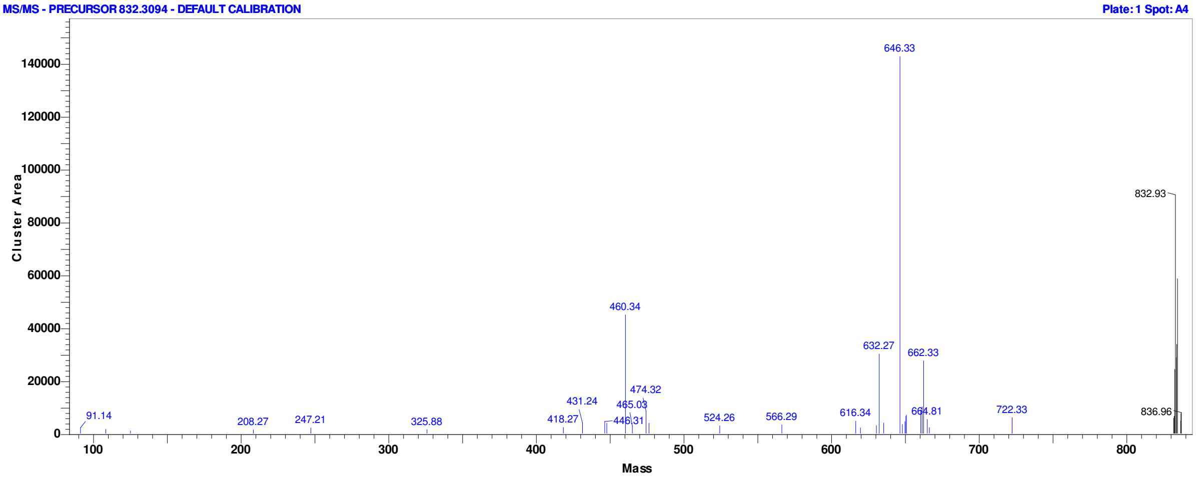
MS/MS - PRECURSOR 832.3094 - DEFAULT CALIBRATION
Plate: 1 Spot: A4
Cluster Area
Mass
91.14
208.27
247.21
325.88
418.27
431.24
446.31
460.34
465.03
474.32
524.26
566.29
616.34
632.27
646.33
662.33
664.81
722.33
832.93
836.96

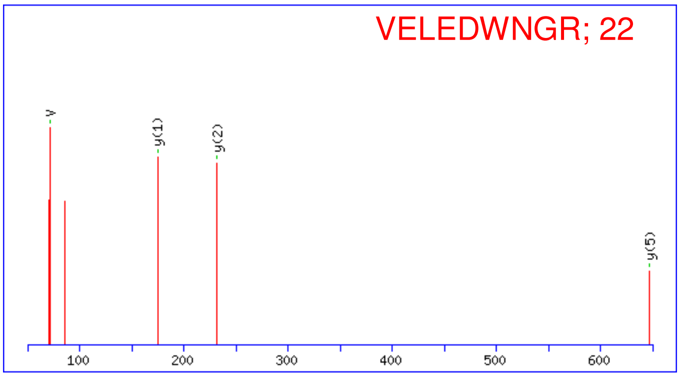
VELEDWNGR; 22
V
y(1)
y(2)
y(5)

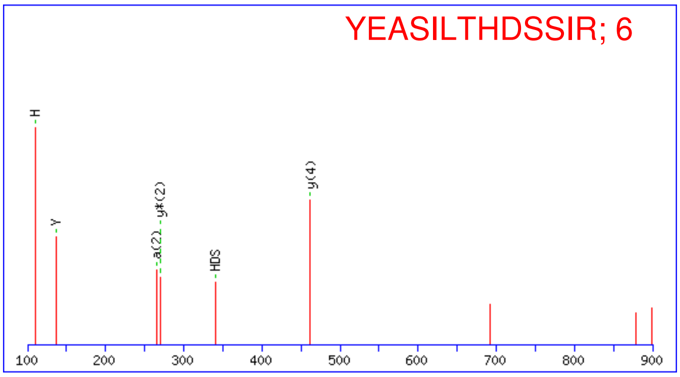
YEASILTHDSSIR; 6
H
Y
a(2)
y*(2)
HDS
y(4)

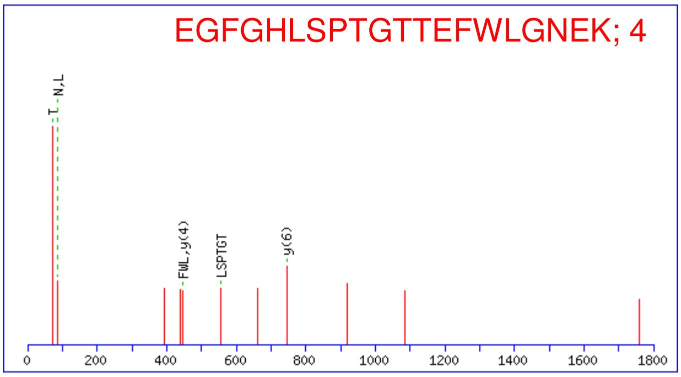
EGFGHLSPTGTTEFWLGNEK; 4
I
N,L
FWL,y(4)
LSPTGT
y(6)

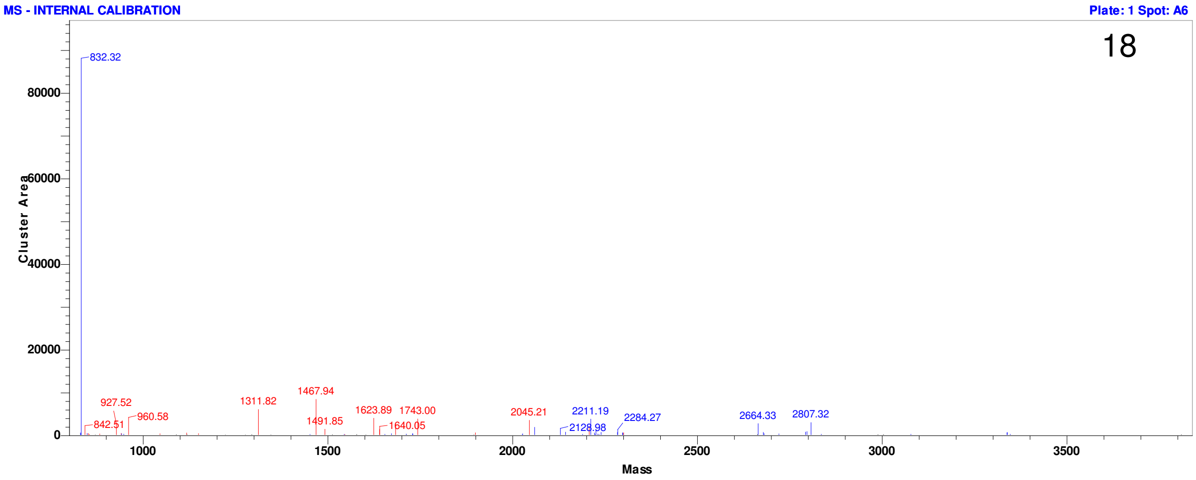
MS - INTERNAL CALIBRATION
Plate: 1 Spot: A6
18
Cluster Area
Mass
832.32
842.51
927.52
960.58
1311.82
1467.94
1491.85
1623.89
1640.05
1743.00
2045.21
2128.98
2211.19
2284.27
2664.33
2807.32

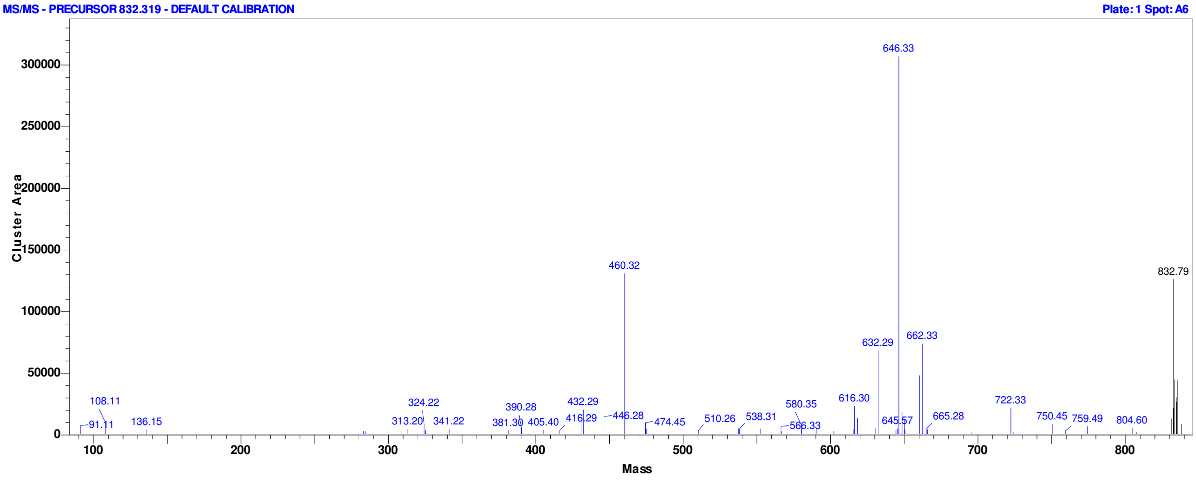
MS/MS - PRECURSOR 832.319 - DEFAULT CALIBRATION
Plate: 1 Spot: A6
Cluster Area
Mass
646.33
460.32
832.79
662.33
632.29
616.30
722.33
108.11
91.11
136.15
324.22
313.20
341.22
390.28
381.30
405.40
432.29
416.29
446.28
474.45
510.26
538.31
580.35
566.33
645.57
665.28
750.45
759.49
804.60

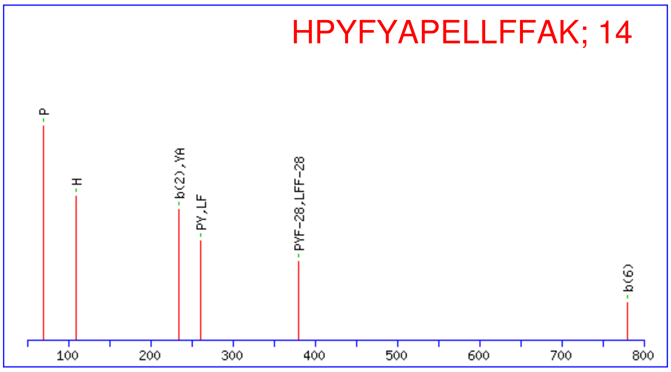
HPYFYAPELLFFAK; 14
P
H
b(2),YA
PY,LF
PYF-28,LFF-28
b(6)

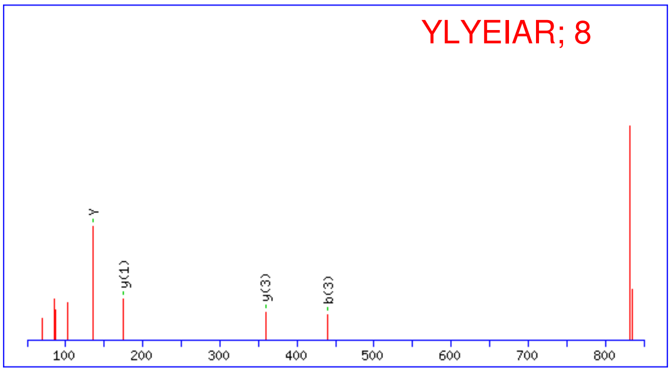
YLYEIAR; 8
Y
y(1)
y(3)
b(3)

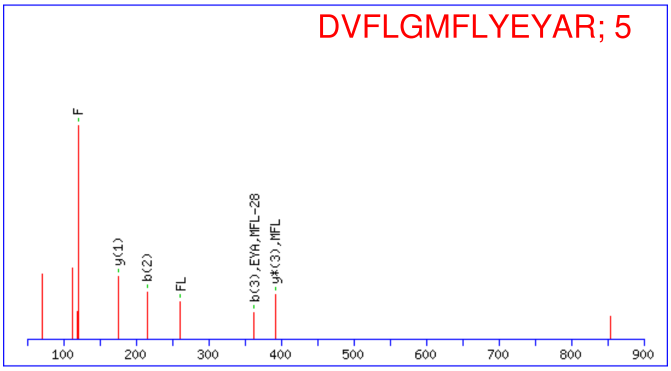
DVFLGMFLYEYAR; 5
F
y(1)
b(2)
FL
b(3),EYA,MFL-28
y*(3),MFL

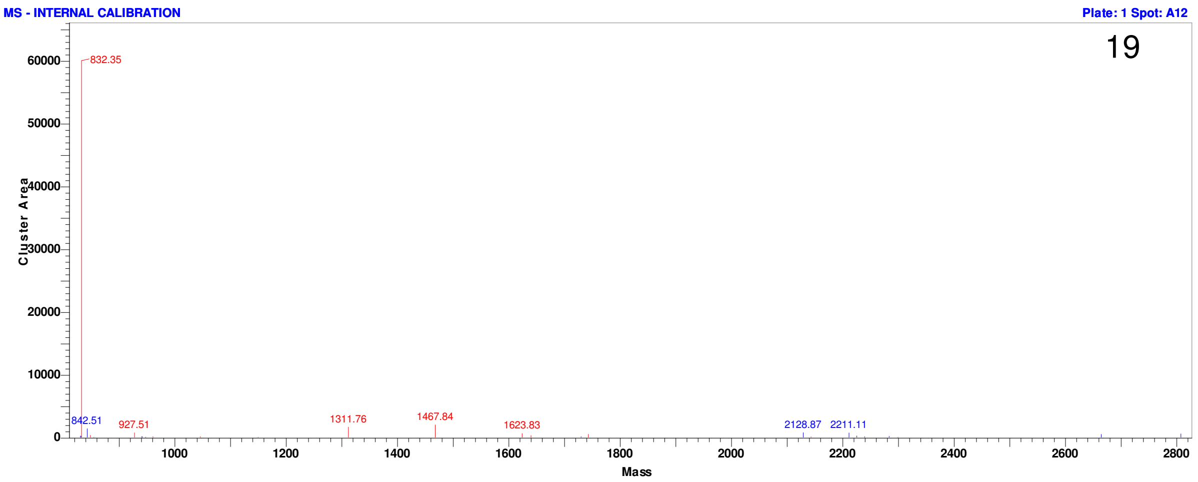
MS - INTERNAL CALIBRATION
Plate: 1 Spot: A12
19
Cluster Area
Mass
832.35
842.51
927.51
1311.76
1467.84
1623.83
2128.87
2211.11

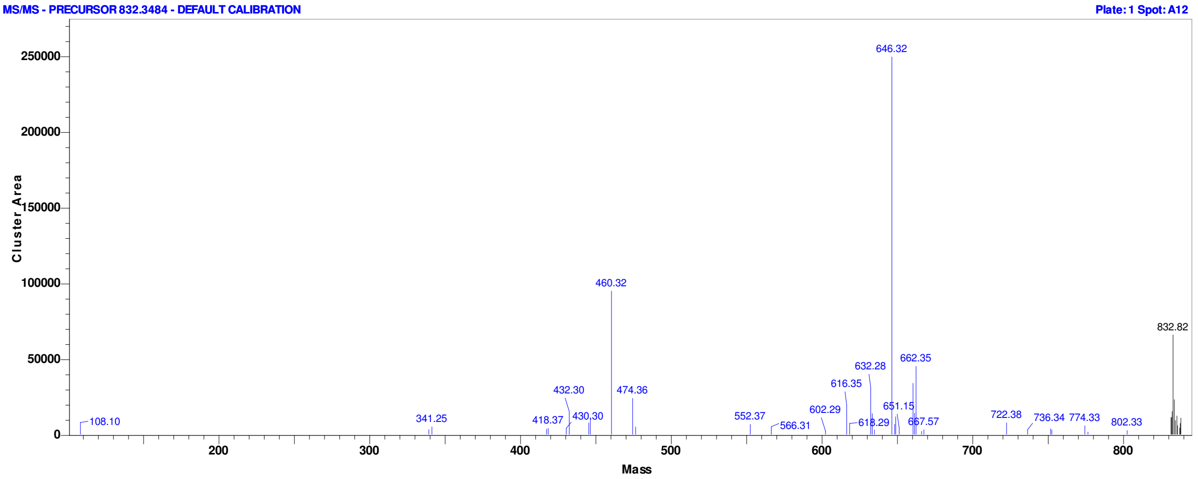
MS/MS - PRECURSOR 832.3484 - DEFAULT CALIBRATION
Plate: 1 Spot: A12
Cluster Area
Mass
646.32
460.32
832.82
662.35
632.28
616.35
602.29
432.30
474.36
651.15
108.10
341.25
418.37
430.30
552.37
566.31
618.29
667.57
722.38
736.34
774.33
802.33

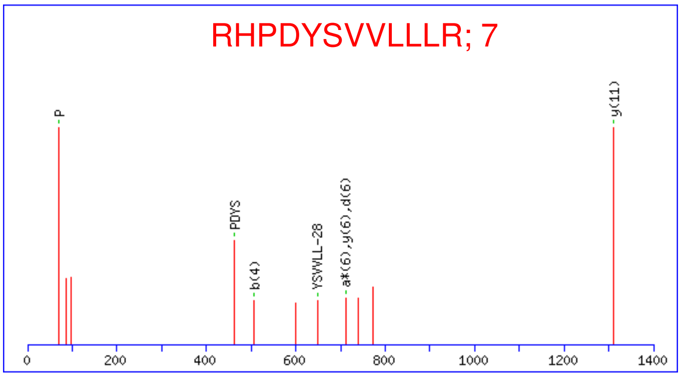
RHPDYSVVLLLR; 7
P
PDYS
b(4)
YSVVLL-28
a*(6),y(6),d(6)
y(11)

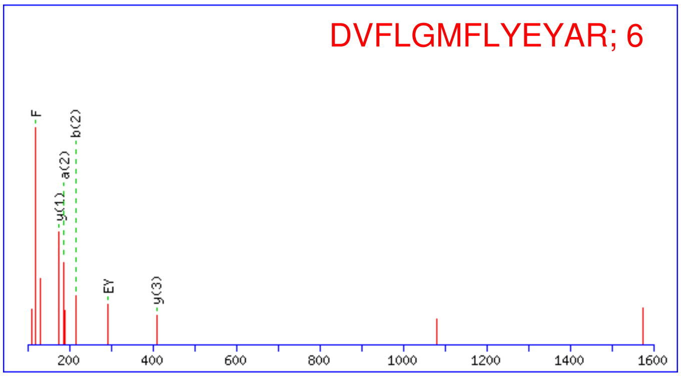
DVFLGMFLYEYAR; 6
F
y(1)
a(2)
b(2)
EY
y(3)

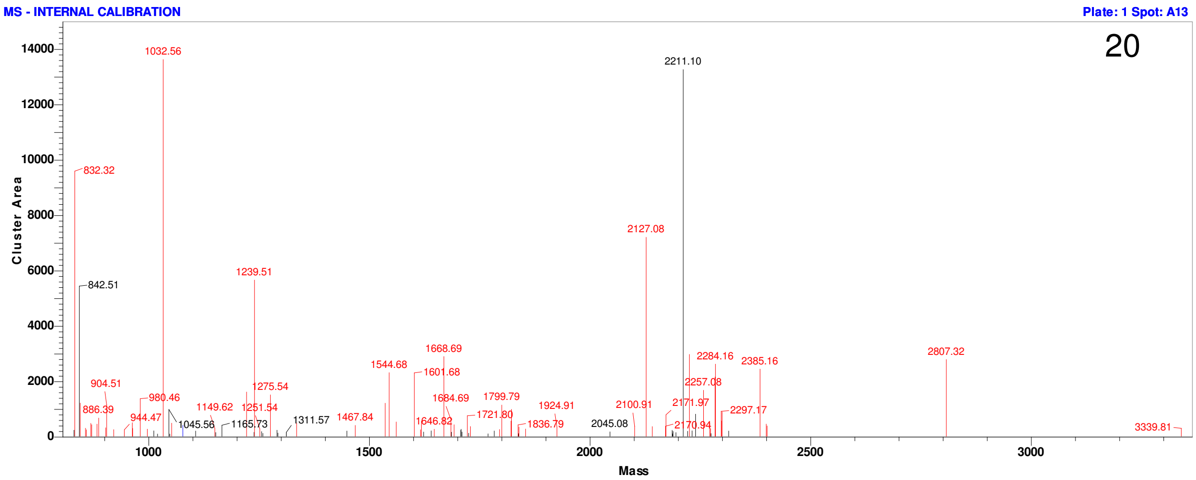
MS - INTERNAL CALIBRATION
Plate: 1 Spot: A13
20
Cluster Area
Mass
1032.56
2211.10
832.32
842.51
2127.08
1239.51
1668.69
1544.68
1601.68
2284.16
2385.16
2807.32
2257.08
904.51
1275.54
980.46
886.39
944.47
1149.62
1251.54
1045.56
1165.73
1311.57
1467.84
1646.82
1684.69
1799.79
1721.80
1836.79
1924.91
2045.08
2100.91
2171.97
2170.94
2297.17
3339.81

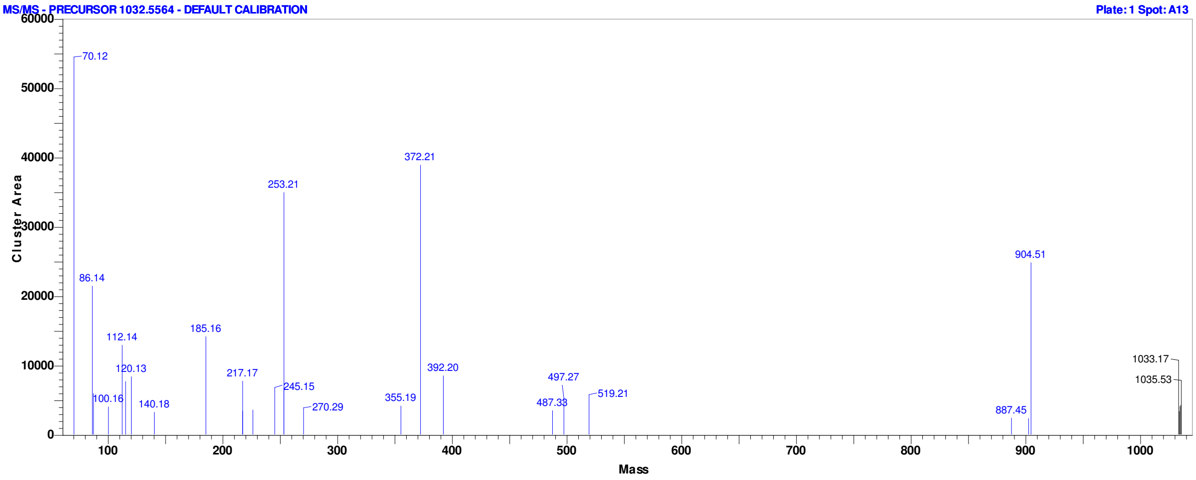
MS/MS - PRECURSOR 1032.5564 - DEFAULT CALIBRATION
Plate: 1 Spot: A13
Cluster Area
Mass
70.12
372.21
253.21
904.51
86.14
185.16
112.14
120.13
217.17
245.15
100.16
140.18
270.29
355.19
392.20
497.27
487.33
519.21
887.45
1033.17
1035.53

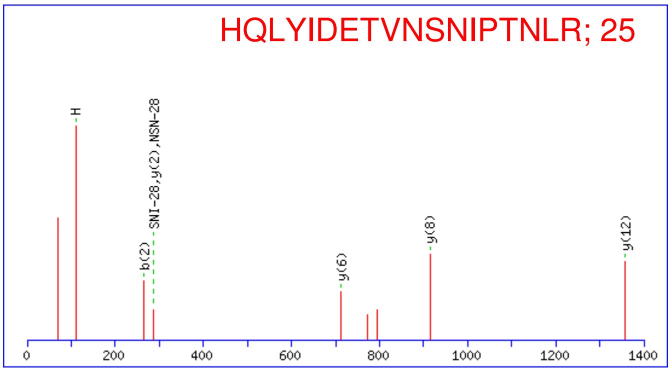
HQLYIDETVNSNIPTNLR; 25
H
SNI-28,y(2),NSN-28
b(2)
y(6)
y(8)
y(12)

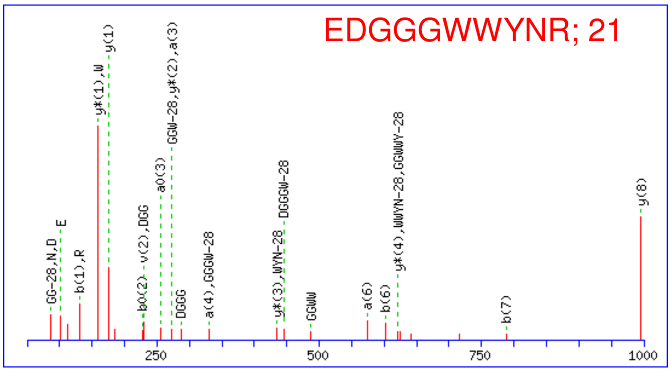
EDGGGWWYNR; 21
y*(1),W
y(1)
GGW-28,y*(2),a(3)
a0(3)
y*(4),WWYN-28,GGWWY-28
DGGGW-28
y(8)
GG-28,N,D
E
b(1),R
b0(2)
y(2),DGG
DGGG
a(4),GGGW-28
y*(3),WYN-28
GGWW
a(6)
b(6)
b(7)

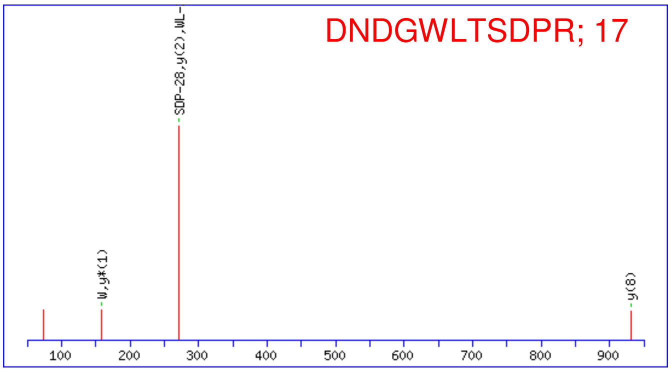
DNDGWLTSDPR; 17
SDP-28,y(2),WL-
W,y*(1)
y(8)

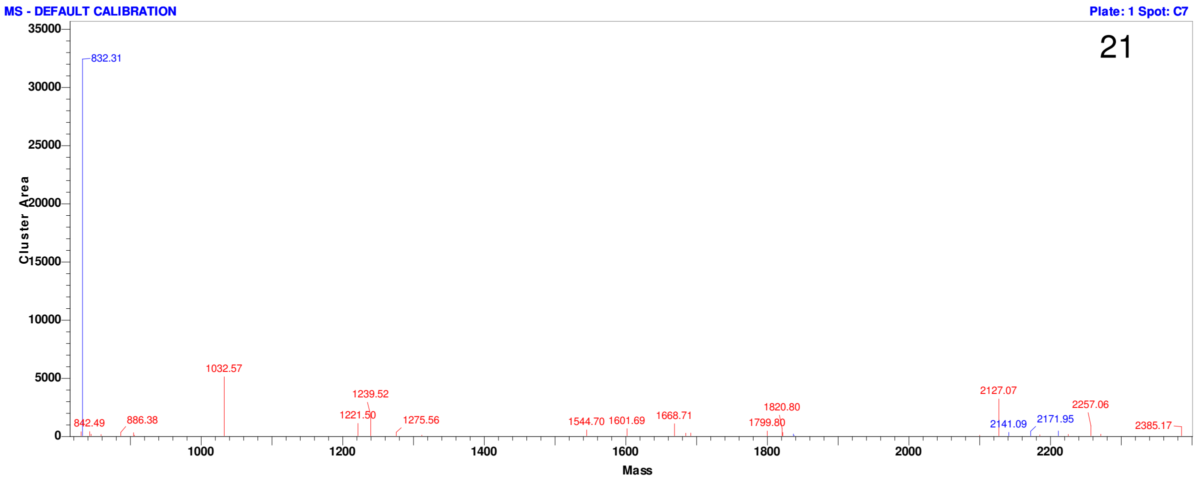
MS - DEFAULT CALIBRATION
Plate: 1 Spot: C7
21
Cluster Area
Mass
832.31
842.49
886.38
1032.57
1221.50
1239.52
1275.56
1544.70
1601.69
1668.71
1799.80
1820.80
2127.07
2141.09
2171.95
2257.06
2385.17

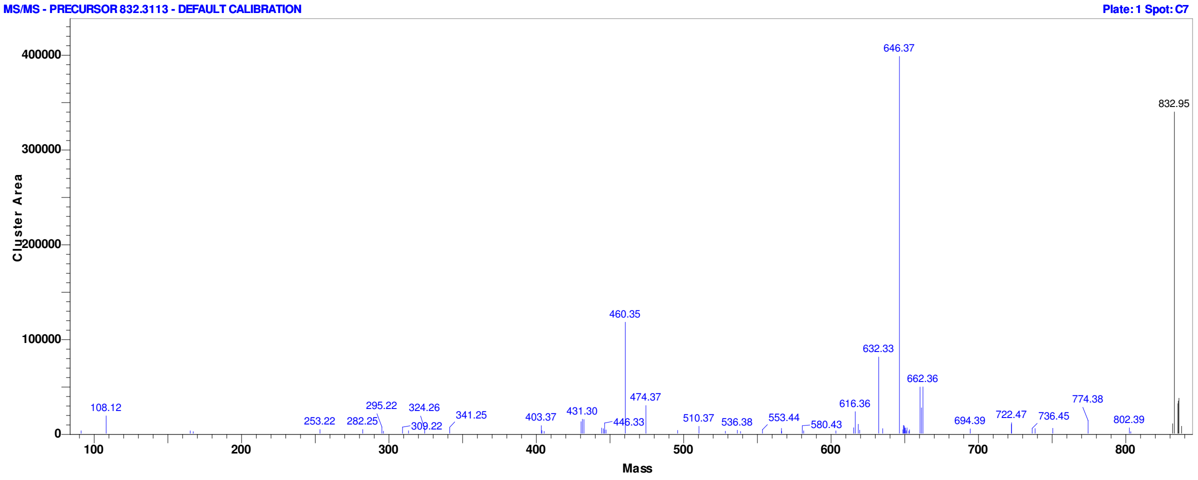
MS/MS - PRECURSOR 832.3113 - DEFAULT CALIBRATION
Plate: 1 Spot: C7
Cluster Area
Mass
646.37
832.95
460.35
632.33
662.36
474.37
108.12
253.22
282.25
295.22
309.22
324.26
341.25
403.37
431.30
446.33
510.37
536.38
553.44
580.43
616.36
694.39
722.47
736.45
774.38
802.39

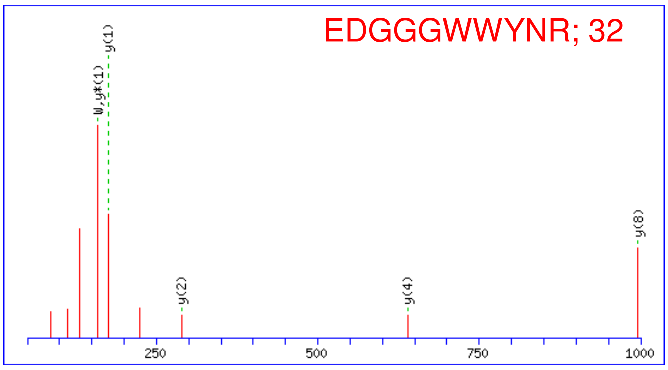
EDGGGWWYNR; 32
W,y*(1)
y(1)
y(2)
y(4)
y(8)

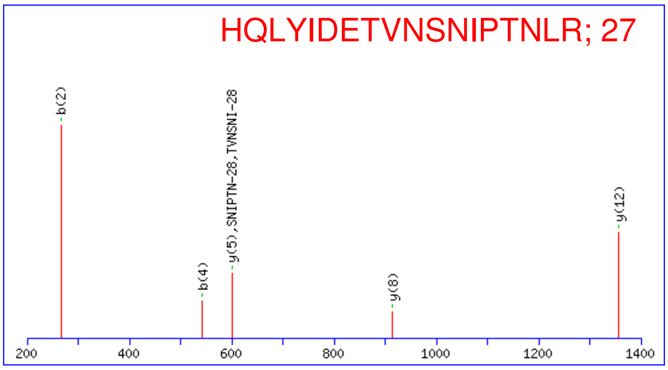
HQLYIDETVNSNIPTNLR; 27
b(2)
b(4)
y(5),SNIPTN-28,TVNSNI-28
y(8)
y(12)

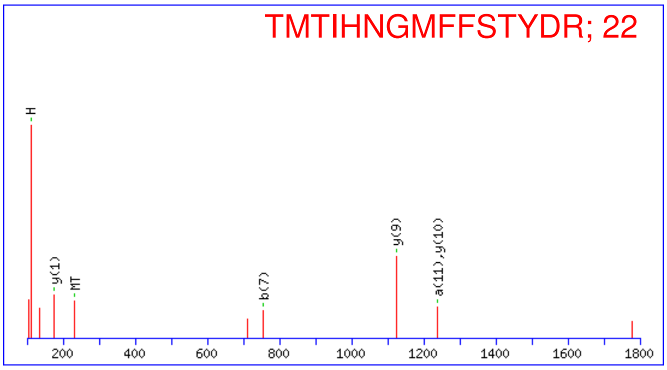
TMTIHNGMFFSTYDR; 22
H
y(1)
MT
b(7)
y(9)
a(11),y(10)

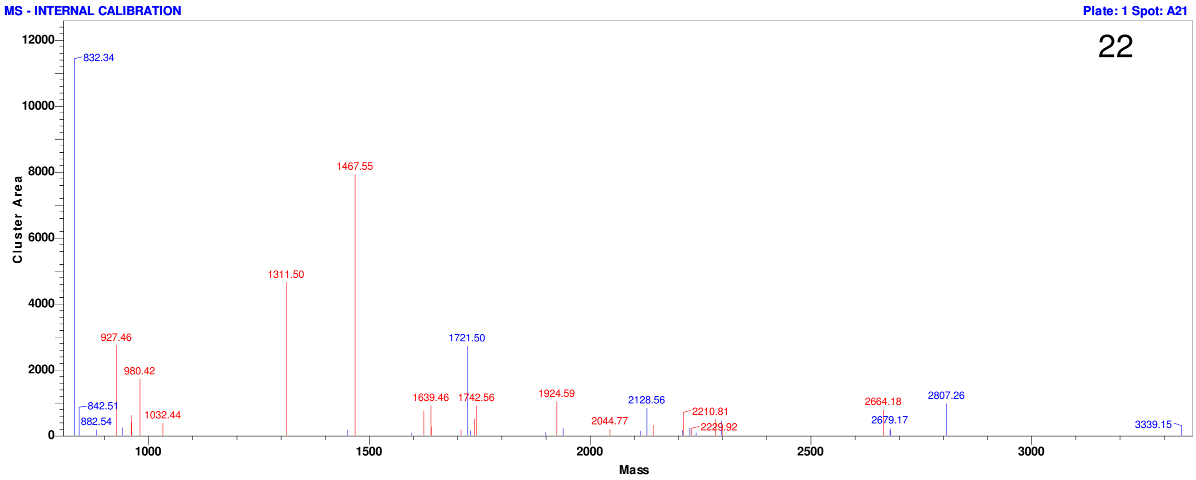

MS - INTERNAL CALIBRATION
Plate: 1 Spot: A21
22
Cluster Area
Mass
832.34
1467.55
1311.50
927.46
980.42
1721.50
842.51
882.54
1032.44
1639.46
1742.56
1924.59
2044.77
2128.56
2210.81
2229.92
2664.18
2679.17
2807.26
3339.15

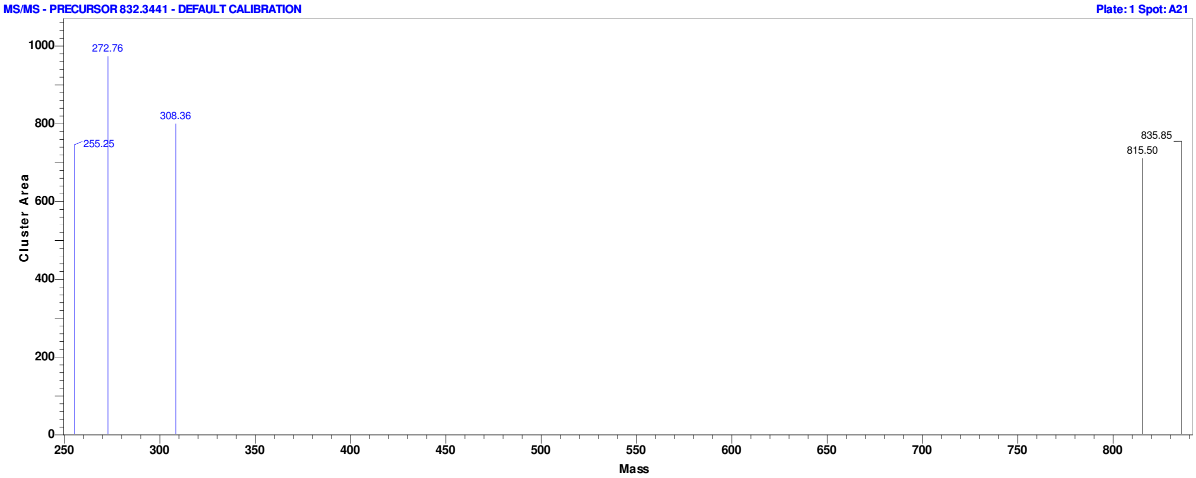

MS/MS - PRECURSOR 832.3441 - DEFAULT CALIBRATION
Plate: 1 Spot: A21
Cluster Area
Mass
272.76
308.36
255.25
835.85
815.50

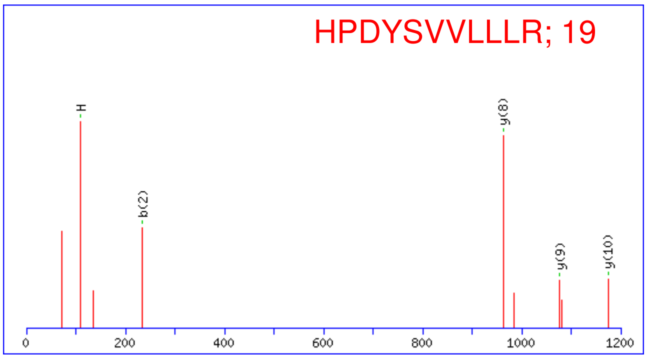

HPDYSVVLLLR; 19
H
b(2)
y(8)
y(9)
y(10)

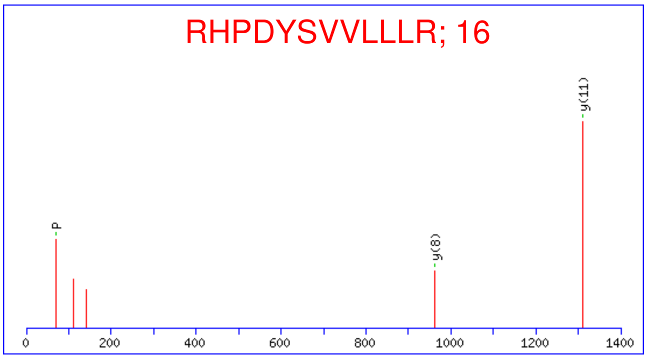

RHPDYSVVLLLR; 16
P
y(8)
y(11)

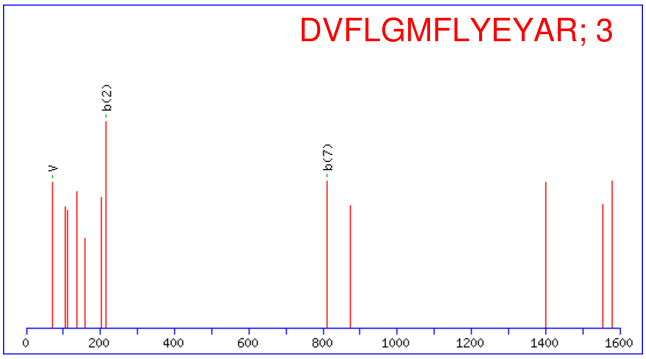

DVFLGMFLYEYAR; 3
V
b(2)
b(7)

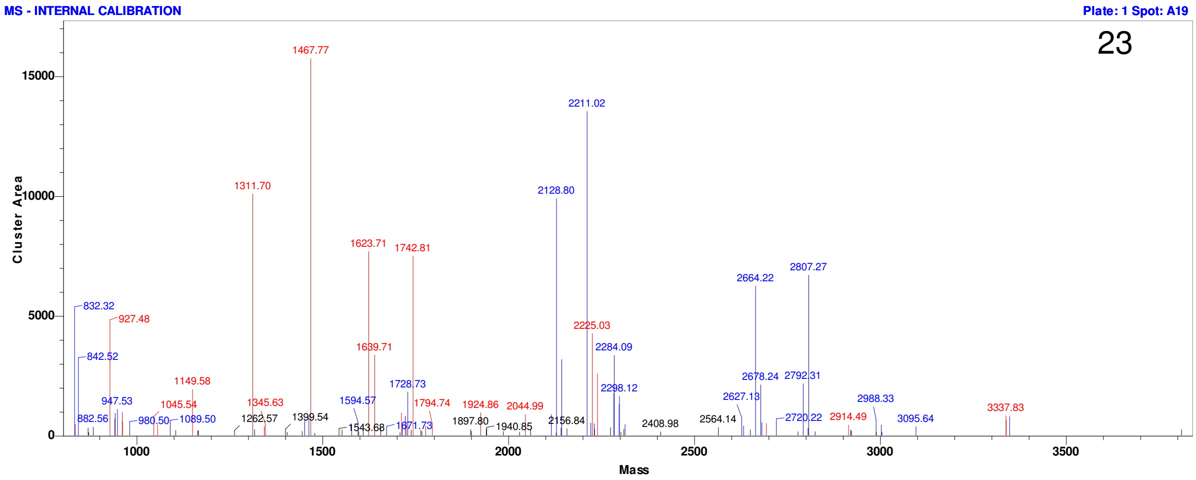
MS - INTERNAL CALIBRATION
Plate: 1 Spot: A19
23
Cluster Area
Mass
1467.77
2211.02
1311.70
2128.80
1623.71
1742.81
2807.27
2664.22
832.32
927.48
2225.03
842.52
1639.71
2284.09
1149.58
1728.73
2678.24
2792.31
2298.12
947.53
1045.54
1345.63
1594.57
1794.74
1924.86
2044.99
2627.13
2988.33
3337.83
882.56
980.50
1089.50
1262.57
1399.54
1543.68
1671.73
1897.80
1940.85
2156.84
2408.98
2564.14
2720.22
2914.49
3095.64

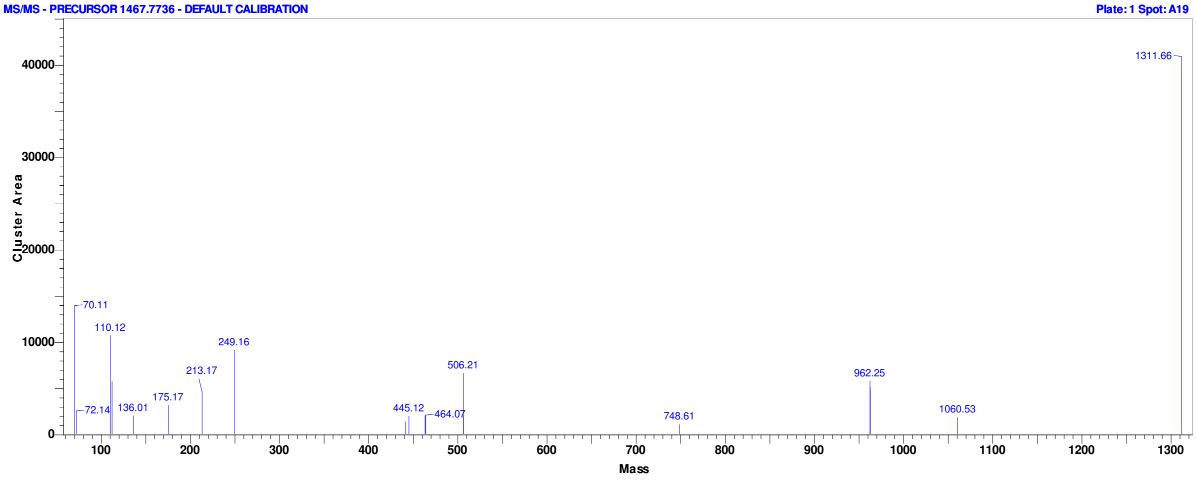
MS/MS - PRECURSOR 1467.7736 - DEFAULT CALIBRATION
Plate: 1 Spot: A19
Cluster Area
Mass
1311.66
70.11
110.12
249.16
213.17
506.21
962.25
72.14
136.01
175.17
445.12
464.07
748.61
1060.53

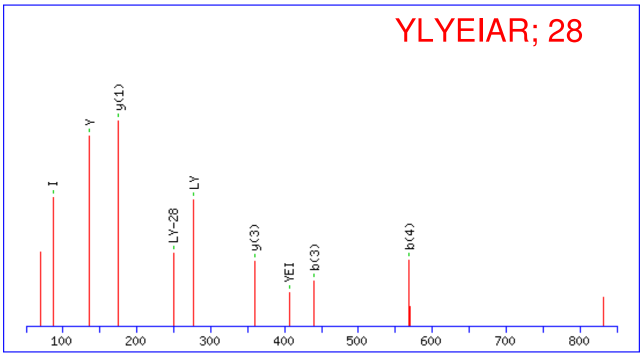
YLYEIAR; 28
I
Y
y(1)
LY-28
LY
y(3)
YEI
b(3)
b(4)

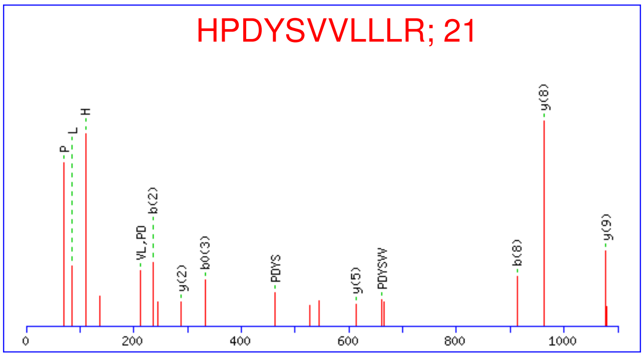
HPDYSVVLLLR; 21
P
L
H
VL,PD
b(2)
y(2)
bo(3)
PDYS
y(5)
PDYSVV
b(8)
y(8)
y(9)

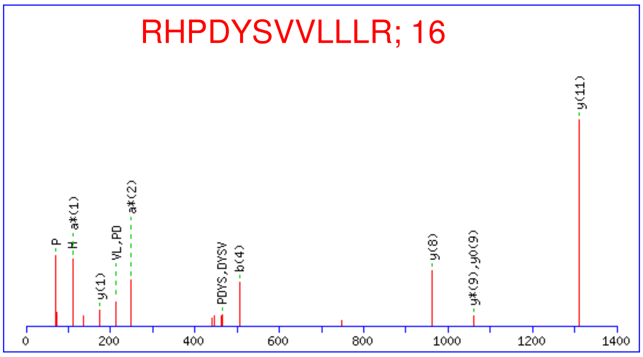
RHPDYSVVLLLR; 16
P
H
a*(1)
y(1)
VL,PD
a*(2)
PDYS,DYSV
b(4)
y(8)
y*(9),yo(9)
y(11)

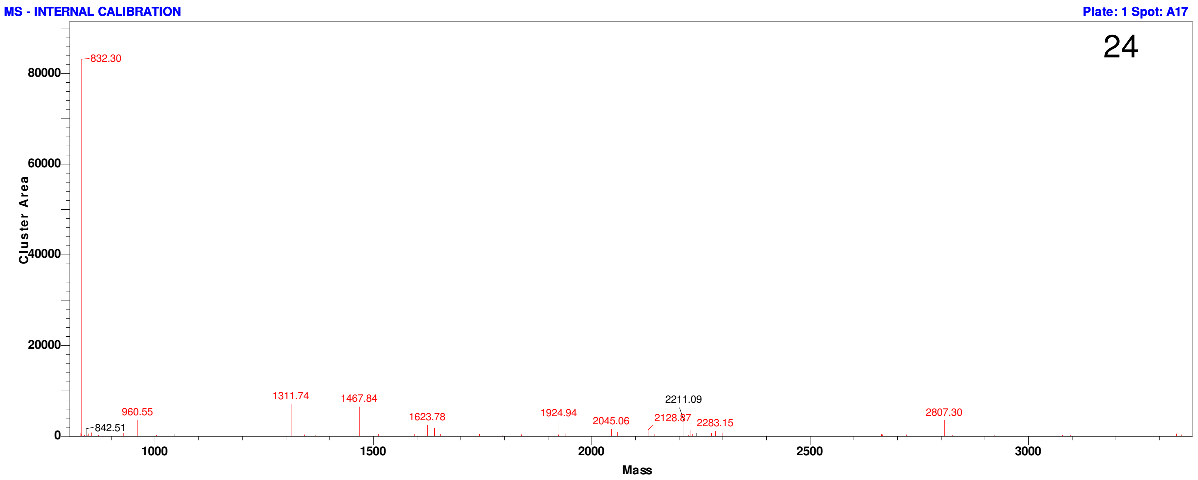
MS - INTERNAL CALIBRATION
Plate: 1 Spot: A17
24
Cluster Area
80000
60000
40000
20000
0
832.30
842.51
960.55
1311.74
1467.84
1623.78
1924.94
2045.06
2128.87
2211.09
2283.15
2807.30
1000
1500
2000
2500
3000
Mass

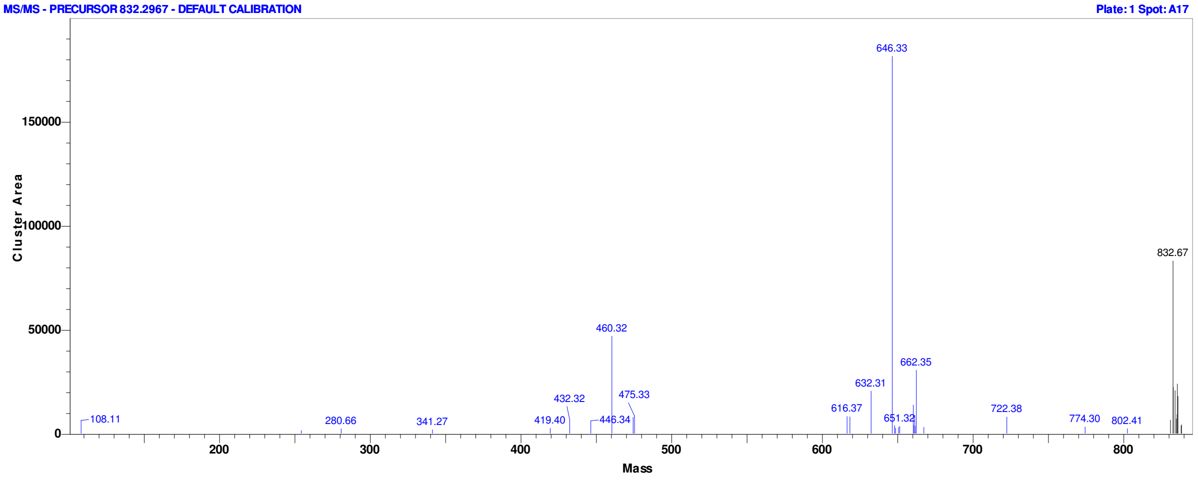
MS/MS - PRECURSOR 832.2967 - DEFAULT CALIBRATION
Plate: 1 Spot: A17
Cluster Area
150000
100000
50000
0
646.33
832.67
460.32
662.35
632.31
475.33
432.32
616.37
722.38
108.11
280.66
341.27
419.40
446.34
651.32
774.30
802.41
200
300
400
500
600
700
800
Mass

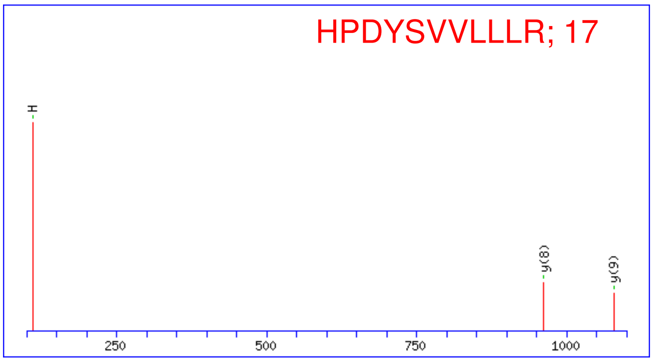
HPDYSVVLLLR; 17
H
y(8)
y(9)
250
500
750
1000

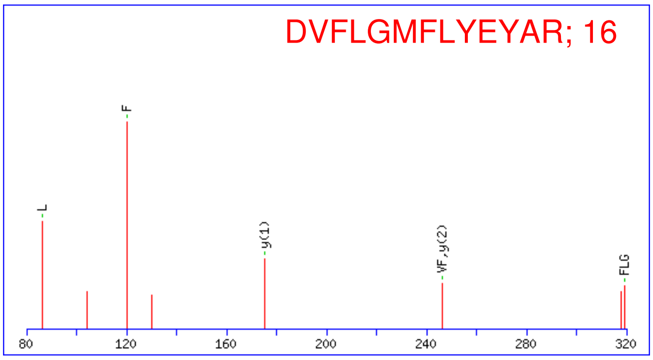
DVFLGMFLYEYAR; 16
F
L
y(1)
VF,y(2)
FLG
80
120
160
200
240
280
320

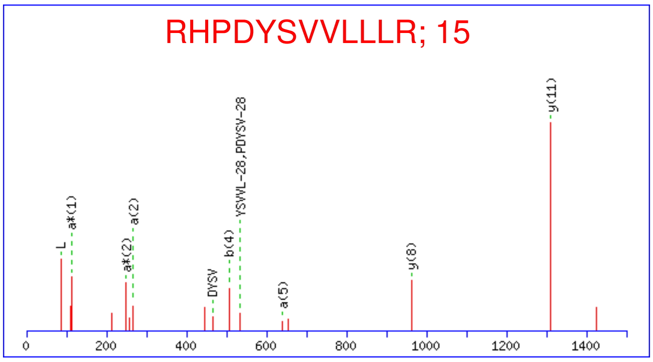
RHPDYSVVLLLR; 15
y(11)
YSVVL-28,PDYSV-28
L
a*(1)
a(2)
a*(2)
b(4)
DYSV
y(8)
a(5)
0
200
400
600
800
1000
1200
1400

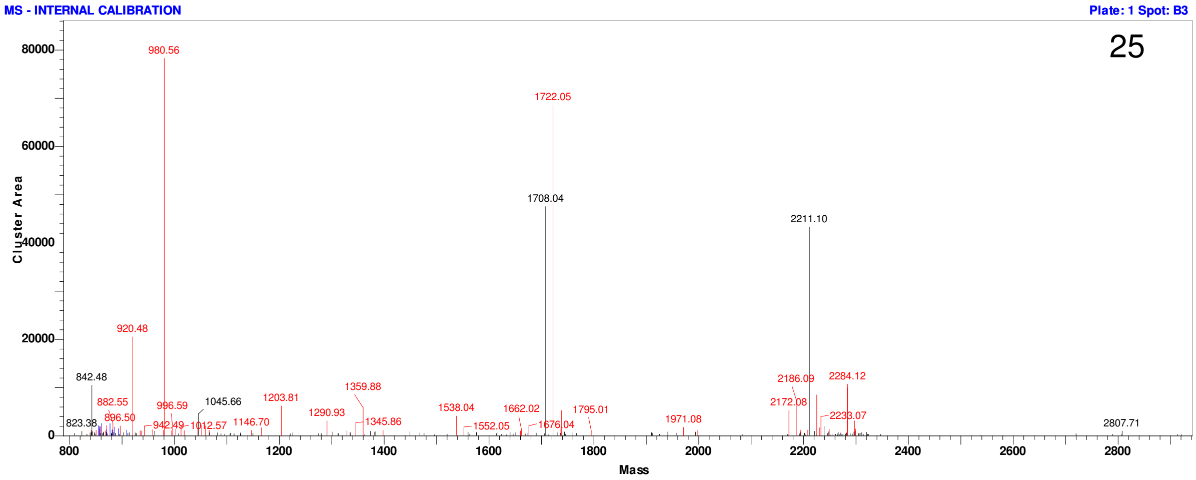
MS - INTERNAL CALIBRATION
Plate: 1 Spot: B3
25
Cluster Area
Mass
980.56
1722.05
1708.04
2211.10
920.48
842.48
2186.09
2284.12
882.55
996.59
1045.66
1203.81
1359.88
1538.04
1662.02
1795.01
2172.08
2233.07
823.38
896.50
942.49
1012.57
1146.70
1290.93
1345.86
1552.05
1676.04
1971.08
2807.71

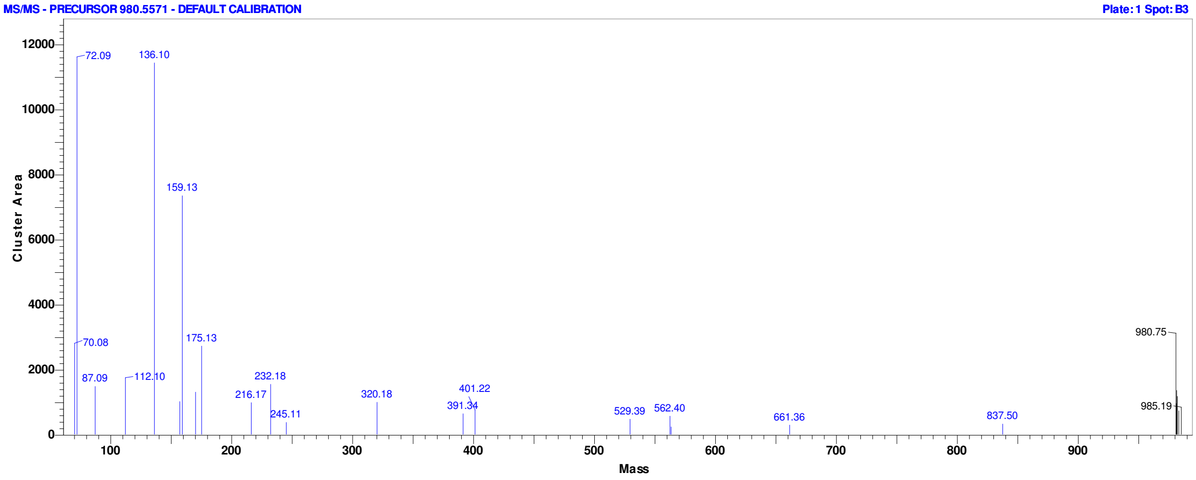
MS/MS - PRECURSOR 980.5571 - DEFAULT CALIBRATION
Plate: 1 Spot: B3
Cluster Area
Mass
72.09
136.10
159.13
70.08
175.13
87.09
112.10
232.18
216.17
245.11
320.18
401.22
391.34
529.39
562.40
661.36
837.50
980.75
985.19

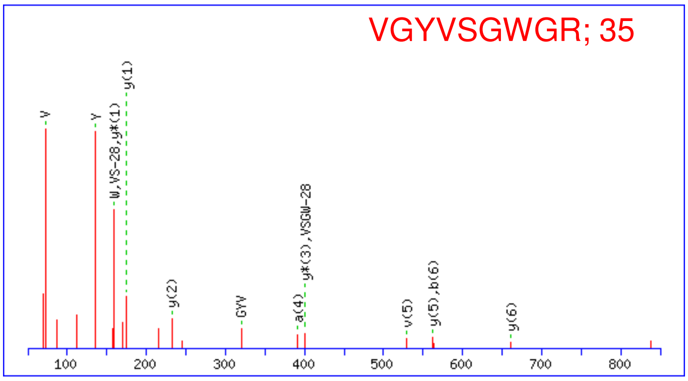
VGYVSGWGR; 35
V
Y
W,VS-28,y*(1)
y(1)
y(2)
GYV
a(4)
y*(3),VSGW-28
y(5)
y(5),b(6)
y(6)

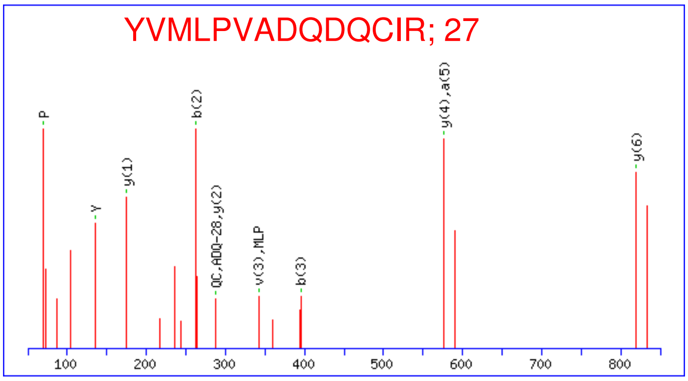
YVMLPVADQDQCIR; 27
P
Y
y(1)
b(2)
QC,ADQ-28,y(2)
y(3),MLP
b(3)
y(4),a(5)
y(6)

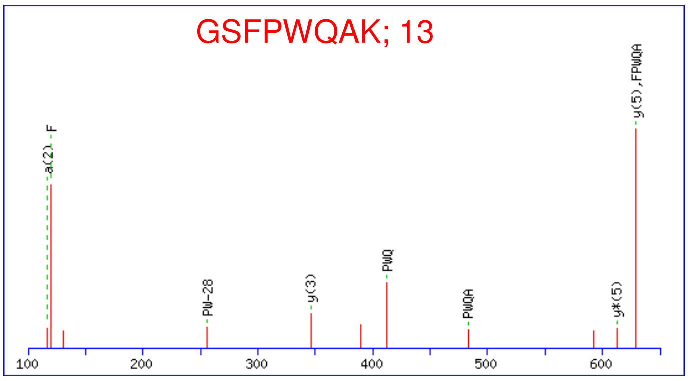
GSFPWQAK; 13
a(2)
F
PW-28
y(3)
PWQ
PWQA
y*(5)
y(5),FPWQA

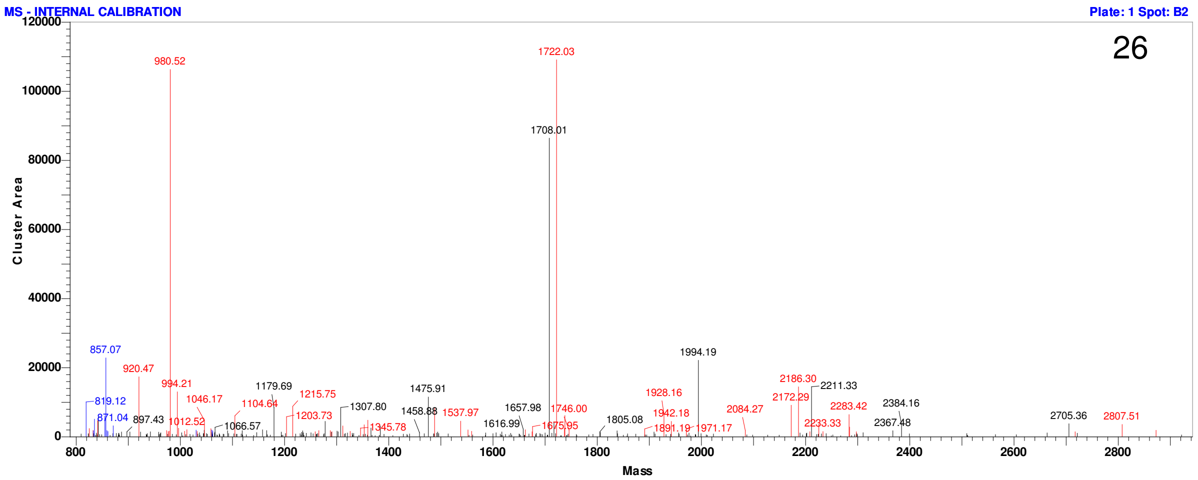
MS - INTERNAL CALIBRATION
Plate: 1 Spot: B2
26
Cluster Area
Mass
980.52
1722.03
1708.01
857.07
920.47
994.21
819.12
1046.17
871.04
897.43
1012.52
1066.57
1104.64
1179.69
1215.75
1203.73
1307.80
1345.78
1475.91
1458.88
1537.97
1657.98
1616.99
1675.95
1746.00
1805.08
1994.19
1928.16
1942.18
1891.19
1971.17
2084.27
2186.30
2172.29
2211.33
2233.33
2283.42
2384.16
2367.48
2705.36
2807.51

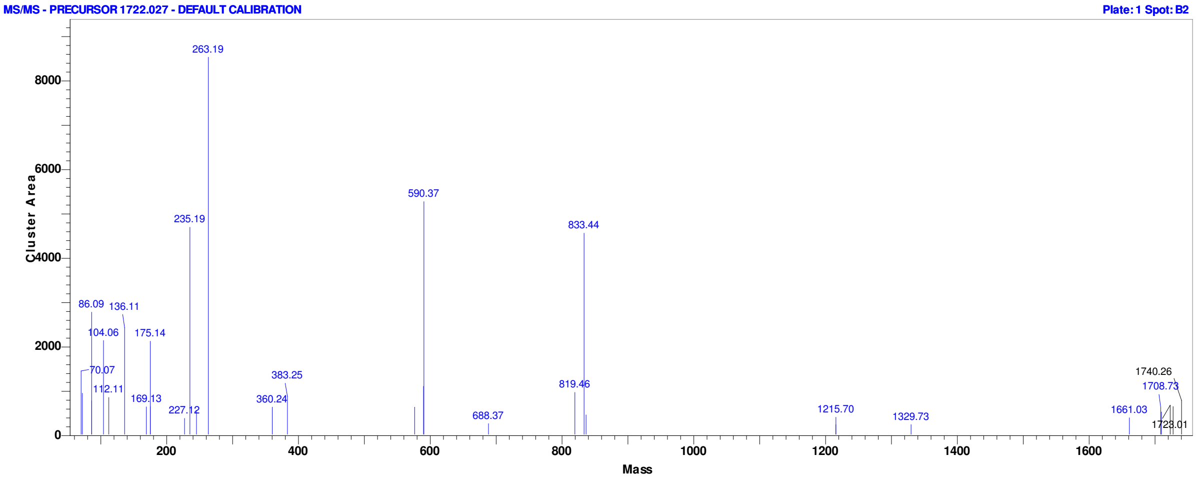
MS/MS - PRECURSOR 1722.027 - DEFAULT CALIBRATION
Plate: 1 Spot: B2
Cluster Area
Mass
263.19
235.19
590.37
833.44
86.09
136.11
104.06
175.14
70.07
112.11
169.13
227.12
383.25
360.24
688.37
819.46
1215.70
1329.73
1661.03
1740.26
1708.73
1723.01

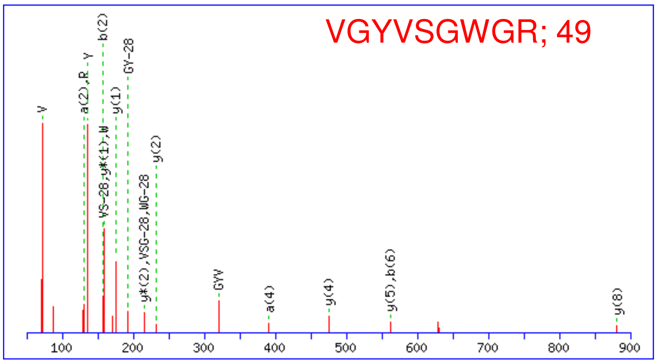
VGYVSGWGR; 49

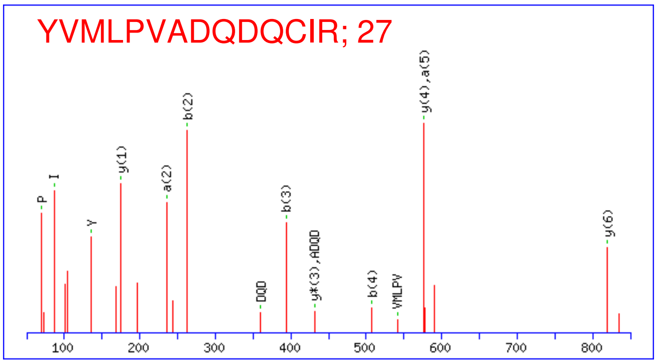
YVMLPVADQDQCIR; 27

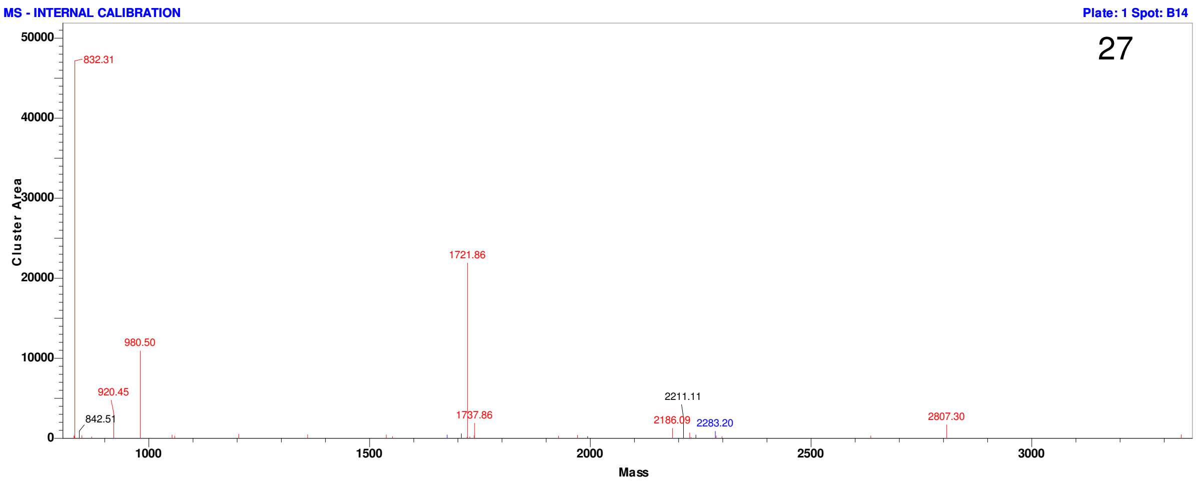
MS - INTERNAL CALIBRATION
Plate: 1 Spot: B14
27
Cluster Area
Mass
832.31
842.51
920.45
980.50
1721.86
1737.86
2186.09
2211.11
2283.20
2807.30
0
10000
20000
30000
40000
50000
1000
1500
2000
2500
3000

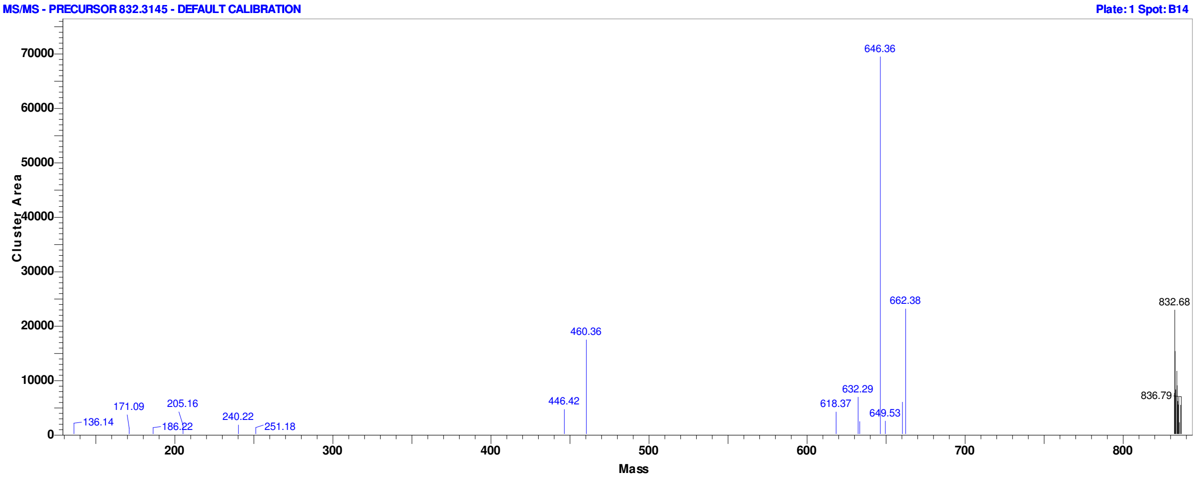
MS/MS - PRECURSOR 832.3145 - DEFAULT CALIBRATION
Plate: 1 Spot: B14
Cluster Area
Mass
136.14
171.09
186.22
205.16
240.22
251.18
446.42
460.36
618.37
632.29
646.36
649.53
662.38
832.68
836.79
0
10000
20000
30000
40000
50000
60000
70000
200
300
400
500
600
700
800

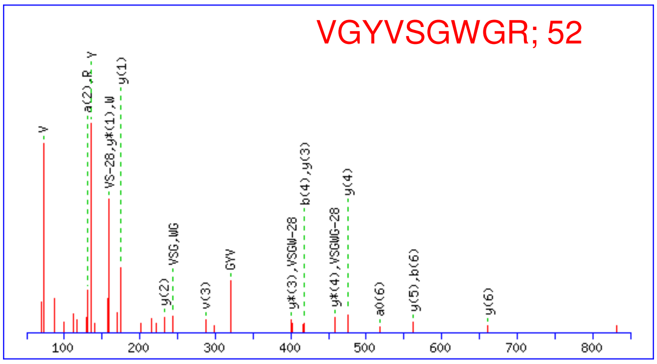
VGYVSGWGR; 52
V
a(2),R-Y
VS-28,y*(1),W
y(1)
y(2)
VSG,WG
v(3)
GYV
y*(3),VSGW-28
b(4),y(3)
y*(4),VSGWG-28
y(4)
a0(6)
y(5),b(6)
y(6)
100
200
300
400
500
600
700
800

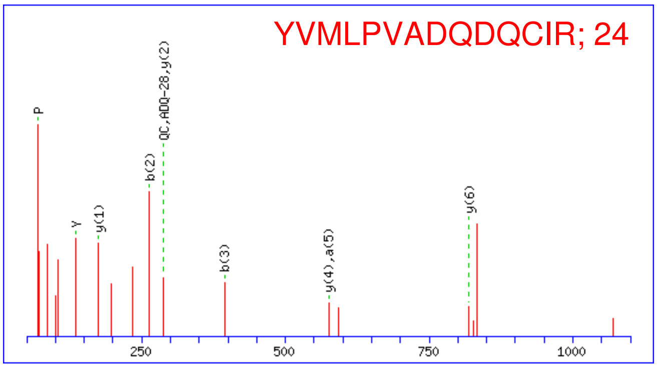
YVMLPVADQDQCIR; 24
P
Y
y(1)
b(2)
QC,ADQ-28,y(2)
b(3)
y(4),a(5)
y(6)
250
500
750
1000

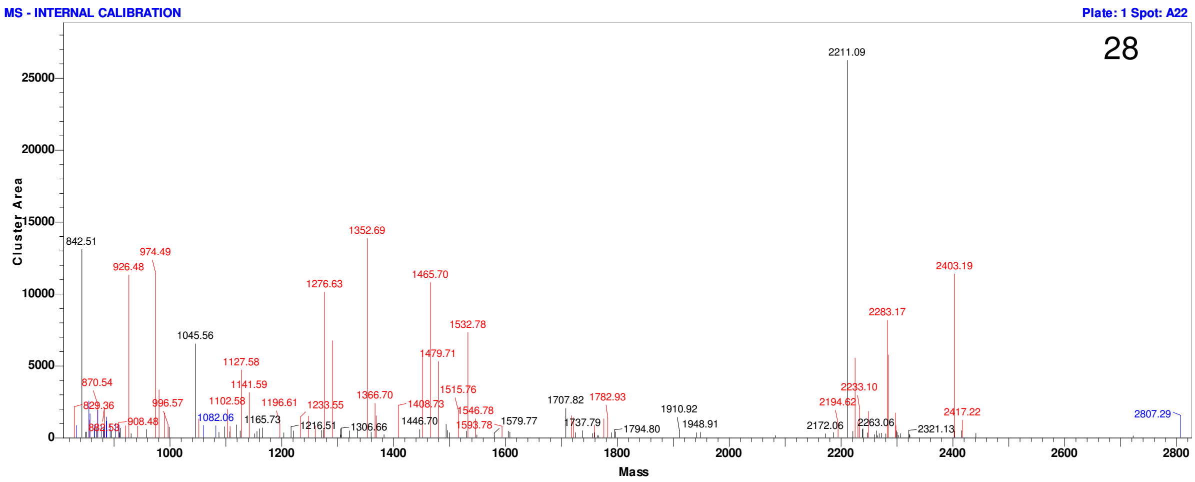
MS - INTERNAL CALIBRATION
Plate: 1 Spot: A22
28
Cluster Area
Mass
2211.09
842.51
974.49
926.48
1352.69
1276.63
1465.70
2403.19
2283.17
1532.78
1045.56
1479.71
1127.58
1141.59
870.54
1515.76
2233.10
1366.70
829.36
996.57
1102.58
1196.61
1233.55
1408.73
1707.82
1782.93
2194.62
1910.92
1546.78
2417.22
2807.29
1082.06
1165.73
1446.70
1579.77
1737.79
1948.91
2263.06
908.48
1593.78
1216.51
1306.66
2172.06
1794.80
2321.13

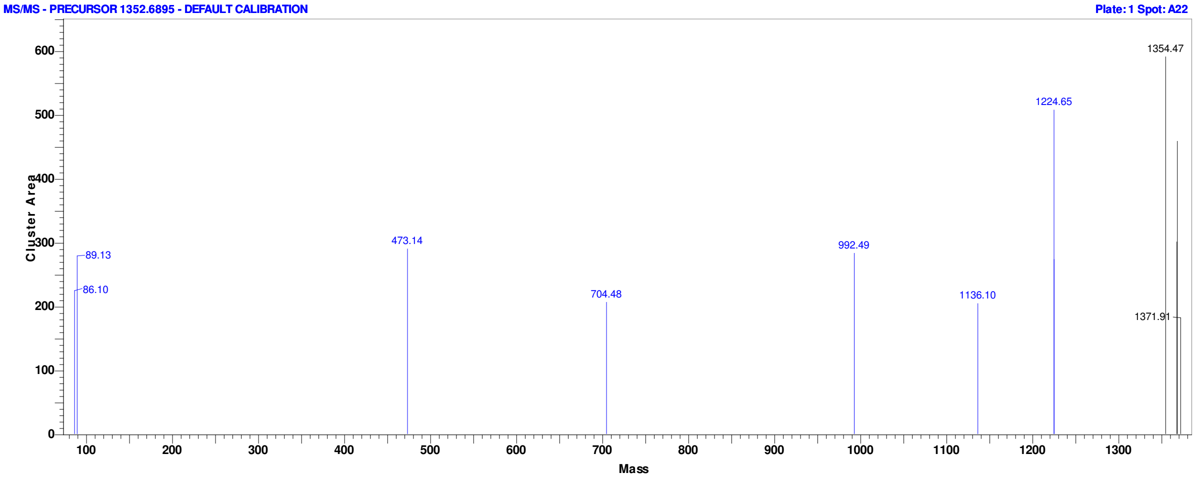
MS/MS - PRECURSOR 1352.6895 - DEFAULT CALIBRATION
Plate: 1 Spot: A22
Cluster Area
Mass
1354.47
1224.65
89.13
86.10
473.14
992.49
704.48
1136.10
1371.91

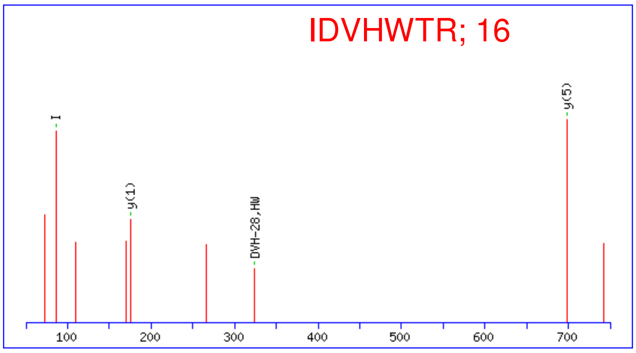
IDVHWTR; 16
I
y(1)
DWH-28,HW
y(5)

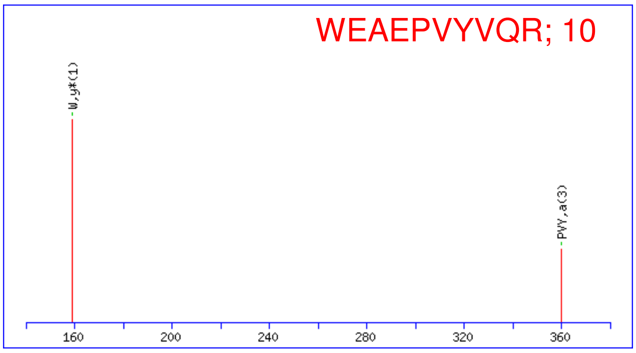
WEAEPVYVQR; 10
W,y*(1)
PVY,a(3)

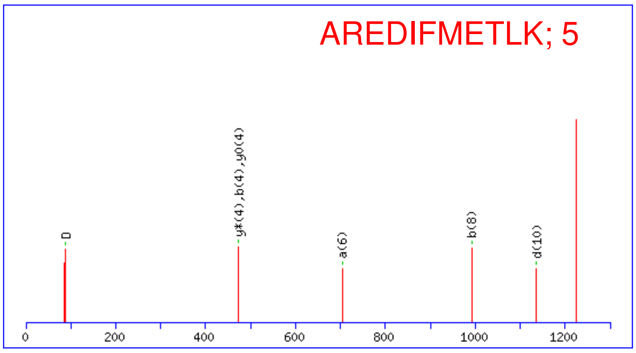
AREDIFMETLK; 5
D
y*(4),b(4),y0(4)
a(6)
b(8)
d(10)

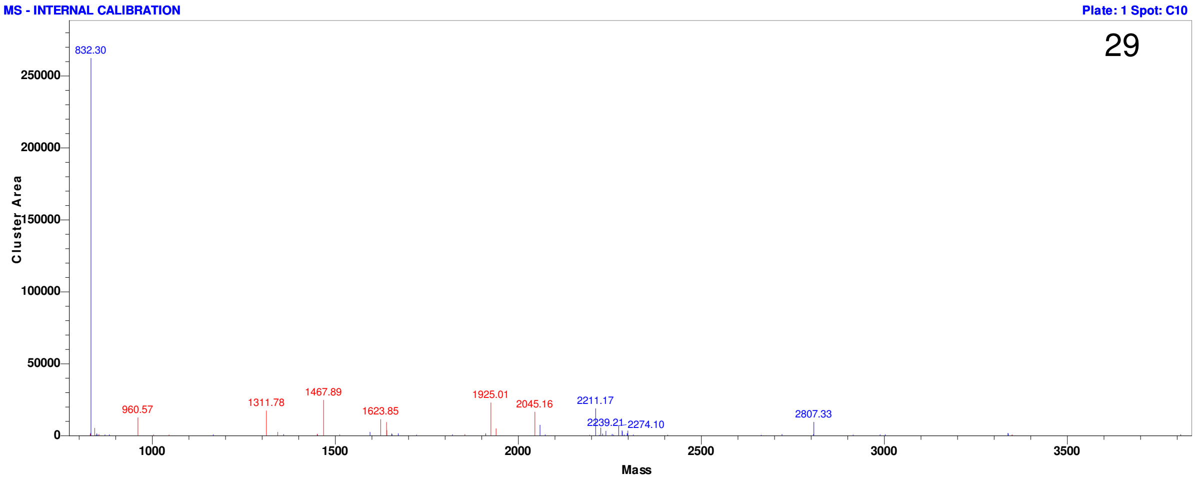
MS - INTERNAL CALIBRATION
Plate: 1 Spot: C10
29
Cluster Area
Mass
832.30
960.57
1311.78
1467.89
1623.85
1925.01
2045.16
2211.17
2239.21
2274.10
2807.33

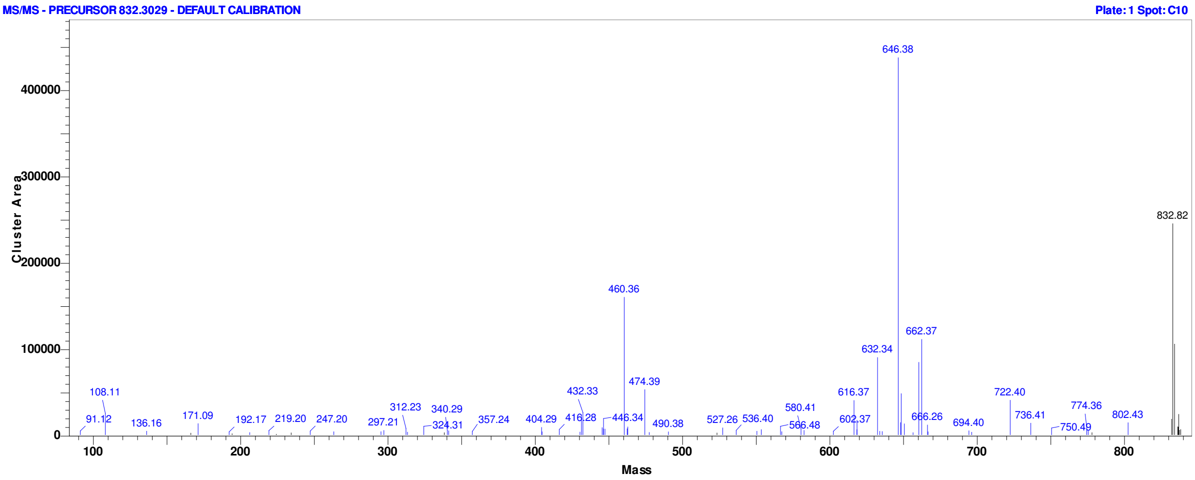
MS/MS - PRECURSOR 832.3029 - DEFAULT CALIBRATION
Plate: 1 Spot: C10
Cluster Area
Mass
646.38
832.82
460.36
662.37
632.34
474.39
722.40
616.37
774.36
802.43
736.41
750.49
694.40
666.26
602.37
580.41
566.48
536.40
527.26
490.38
446.34
432.33
416.28
404.29
357.24
340.29
324.31
312.23
297.21
247.20
219.20
192.17
171.09
136.16
108.11
91.12

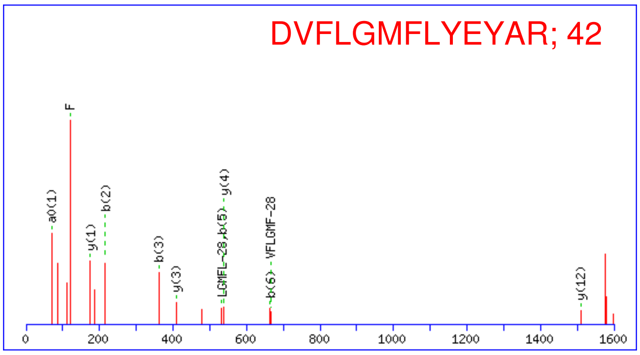
DVFLGMFLYEYAR; 42
a0(1)
F
y(1)
b(2)
b(3)
y(3)
LGMFL-28,b(5)
y(4)
b(6)
VFLGMF-28
y(12)

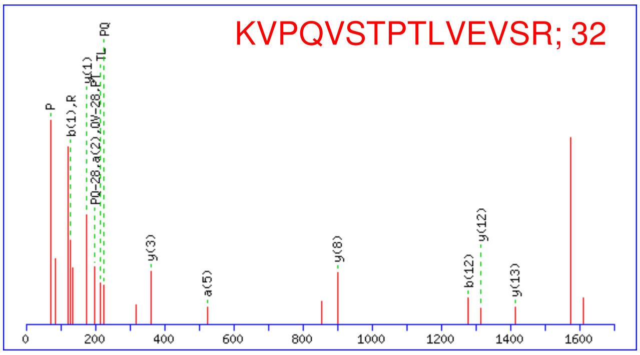
KVPQVSTPTLVEVSR; 32
P
b(1),R
PQ-28,a(2),QV-28,PT
y(1)
TL
PQ
y(3)
a(5)
y(8)
b(12)
y(12)
y(13)

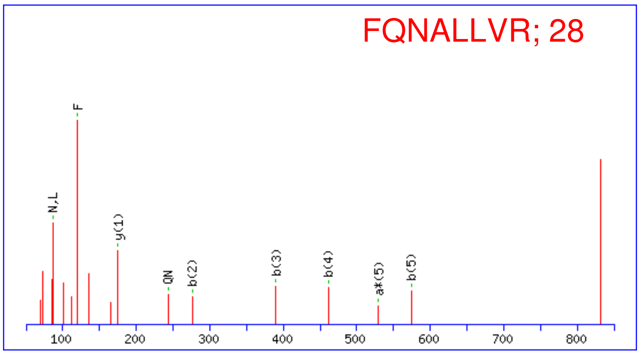
FQNALLVR; 28
N,L
F
y(1)
QN
b(2)
b(3)
b(4)
a*(5)
b(5)

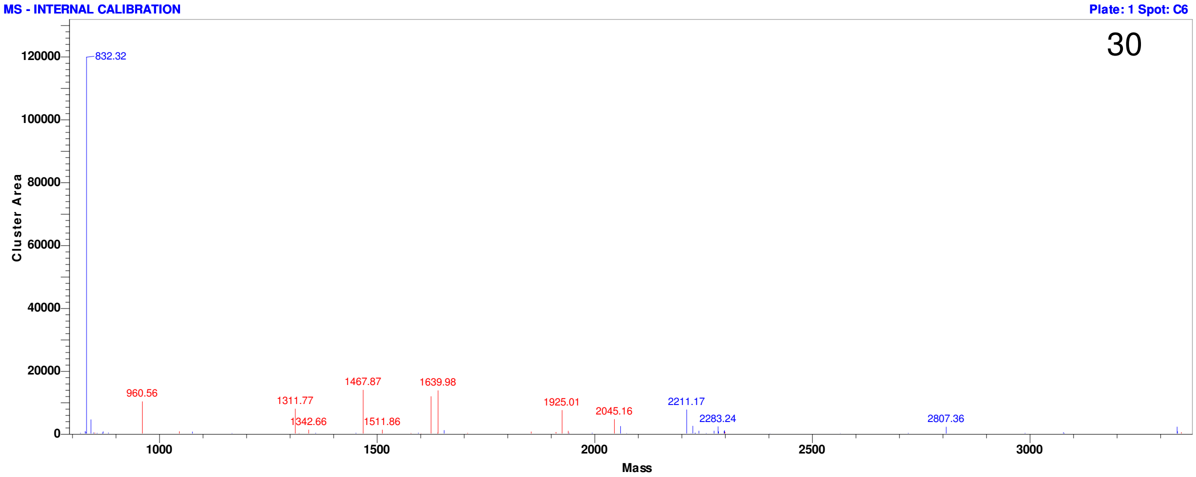
MS - INTERNAL CALIBRATION
Plate: 1 Spot: C6
30
Cluster Area
Mass
832.32
960.56
1311.77
1342.66
1467.87
1511.86
1639.98
1925.01
2045.16
2211.17
2283.24
2807.36

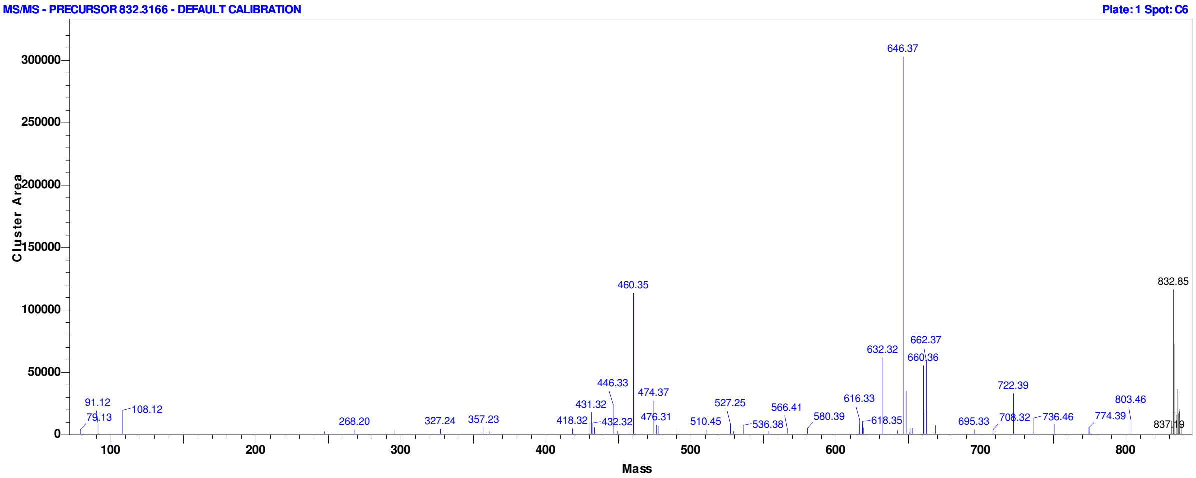
MS/MS - PRECURSOR 832.3166 - DEFAULT CALIBRATION
Plate: 1 Spot: C6
Cluster Area
Mass
646.37
460.35
832.85
662.37
660.36
632.32
446.33
474.37
431.32
527.25
566.41
616.33
722.39
803.46
91.12
79.13
108.12
268.20
327.24
357.23
418.32
432.32
476.31
510.45
536.38
580.39
618.35
695.33
708.32
736.46
774.39
837.19

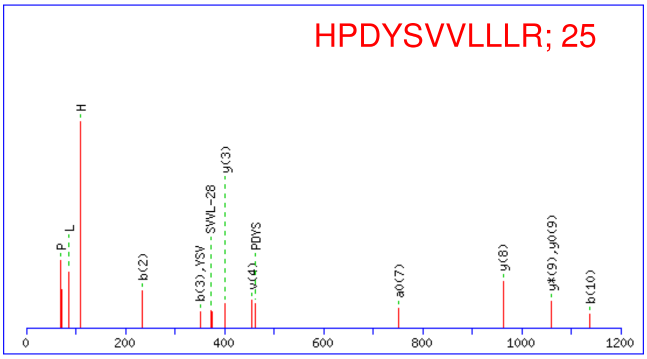
HPDYSVVLLLR; 25
H
P
L
b(2)
b(3),YSV
SVVL-28
y(3)
y(4)
PDYS
a0(7)
y(8)
y*(9),y0(9)
b(10)

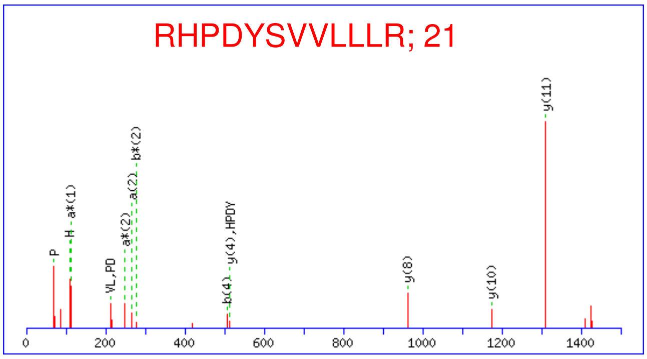
RHPDYSVVLLLR; 21
P
H
a*(1)
VL,PD
a*(2)
a(2)
b*(2)
b(4)
y(4),HPDY
y(8)
y(10)
y(11)

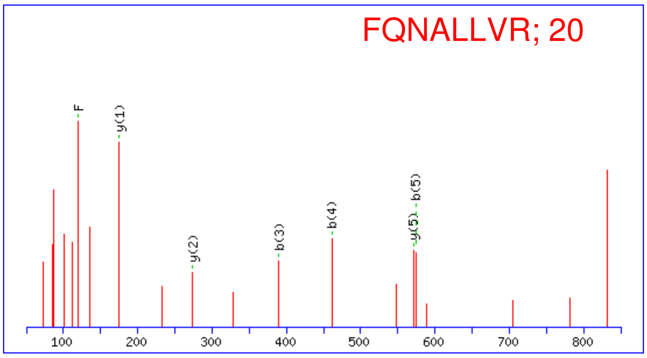
FQNALLVR; 20
F
y(1)
y(2)
b(3)
b(4)
y(5)
b(5)

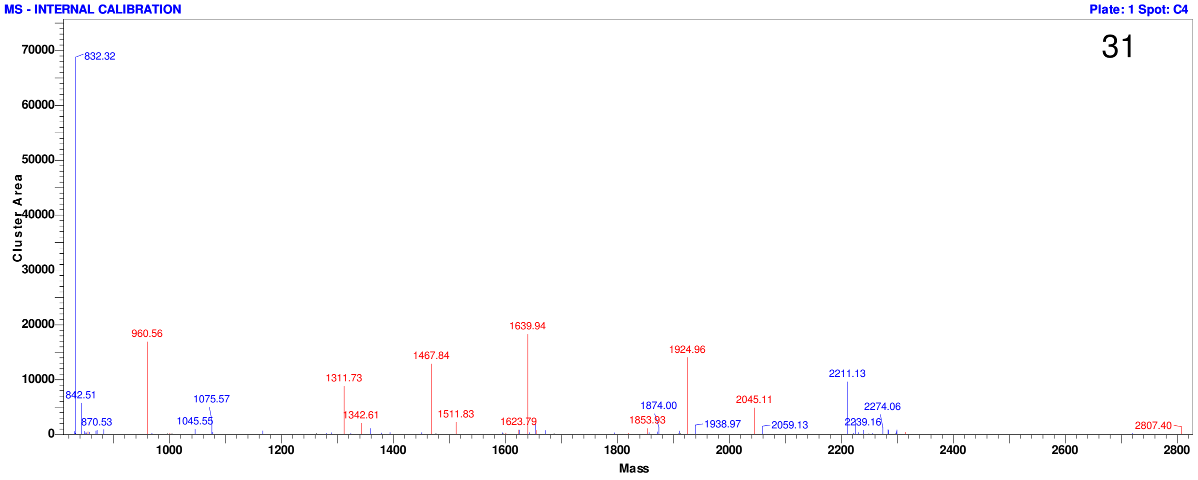
MS - INTERNAL CALIBRATION
Plate: 1 Spot: C4
31
Cluster Area
Mass
832.32
842.51
870.53
960.56
1045.55
1075.57
1311.73
1342.61
1467.84
1511.83
1623.79
1639.94
1853.93
1874.00
1924.96
1938.97
2045.11
2059.13
2211.13
2239.16
2274.06
2807.40

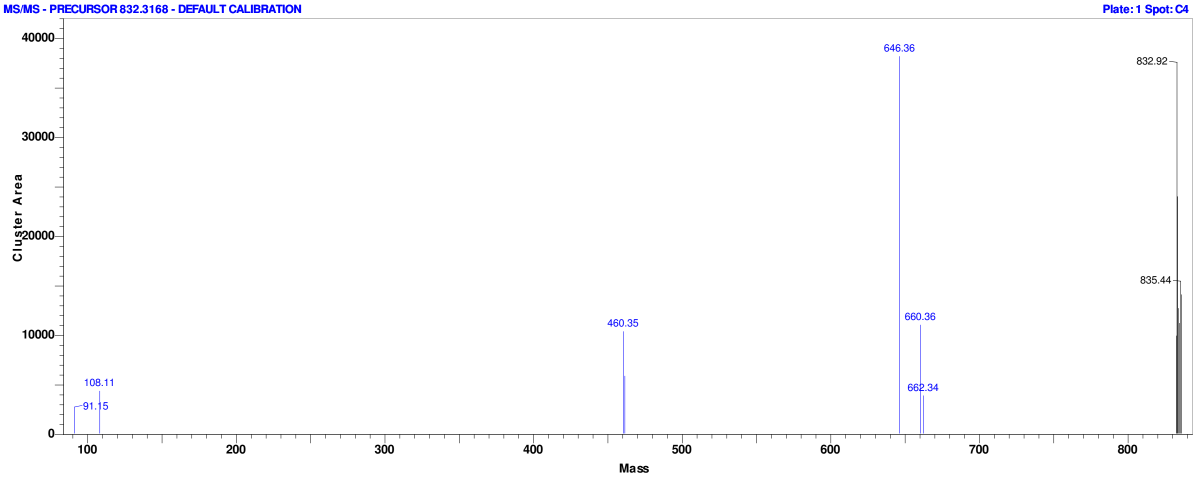
MS/MS - PRECURSOR 832.3168 - DEFAULT CALIBRATION
Plate: 1 Spot: C4
Cluster Area
Mass
91.15
108.11
460.35
646.36
660.36
662.34
832.92
835.44

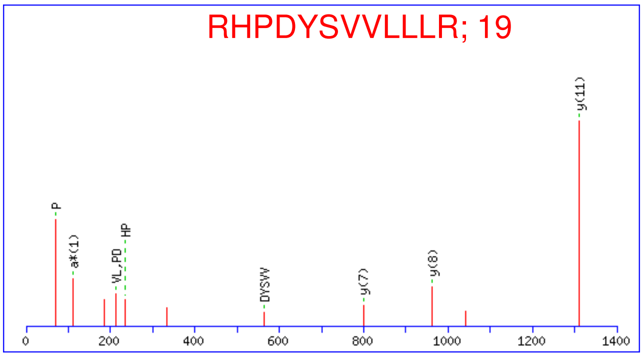
RHPDYSVVLLLR; 19
P
a*(1)
VL,PD
HP
DYSVV
y(7)
y(8)
y(11)

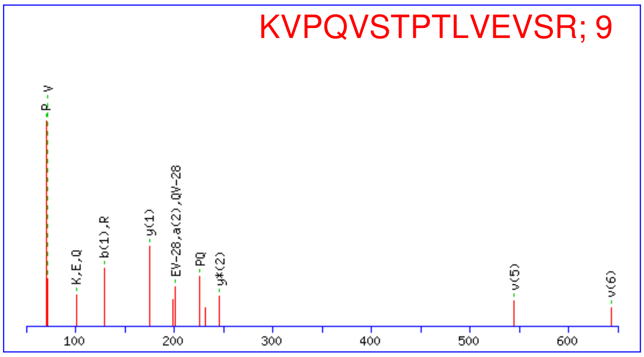
KVPQVSTPTLVEVSR; 9
P,V
K,E,Q
b(1),R
y(1)
EV-28,a(2),QV-28
PQ
y*(2)
y(5)
y(6)

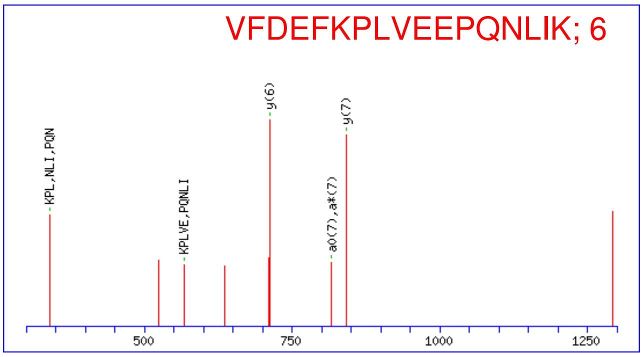
VFDEFKPLVEEPQNLIK; 6
KPL,NLI,PQN
KPLVE,PQNLI
y(6)
a0(7),a*(7)
y(7)

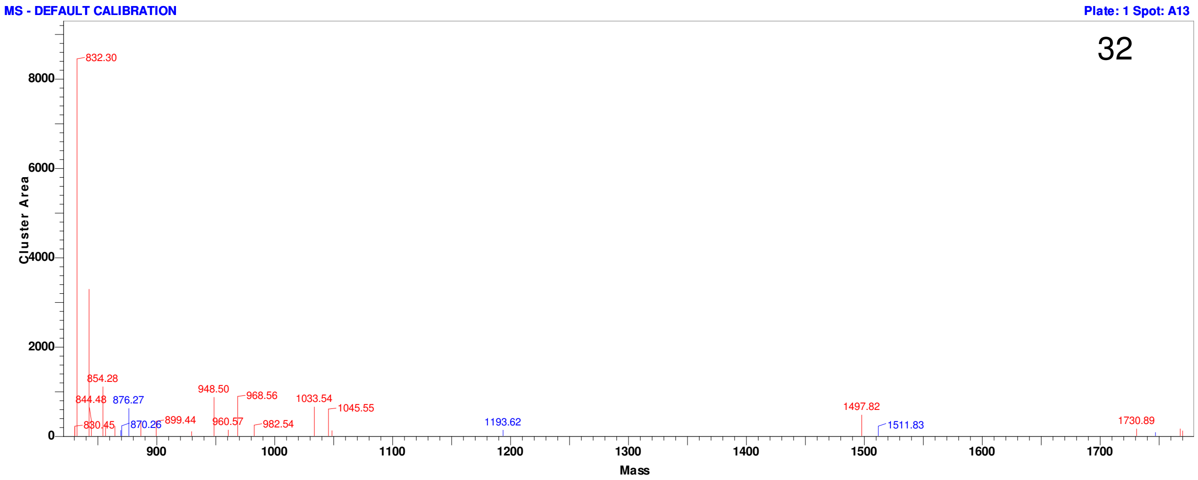

MS - DEFAULT CALIBRATION
Plate: 1 Spot: A13
32
Cluster Area
Mass
832.30
854.28
844.48
876.27
870.26
830.45
899.44
948.50
960.57
968.56
982.54
1033.54
1045.55
1193.62
1497.82
1511.83
1730.89

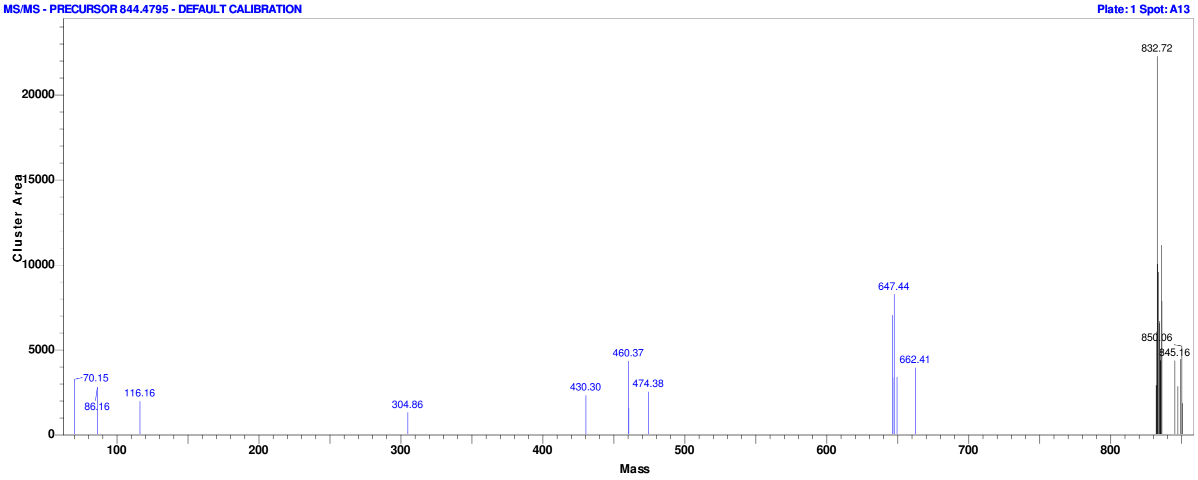

MS/MS - PRECURSOR 844.4795 - DEFAULT CALIBRATION
Plate: 1 Spot: A13
Cluster Area
Mass
832.72
647.44
850.06
845.16
662.41
460.37
474.38
430.30
304.86
70.15
116.16
86.16

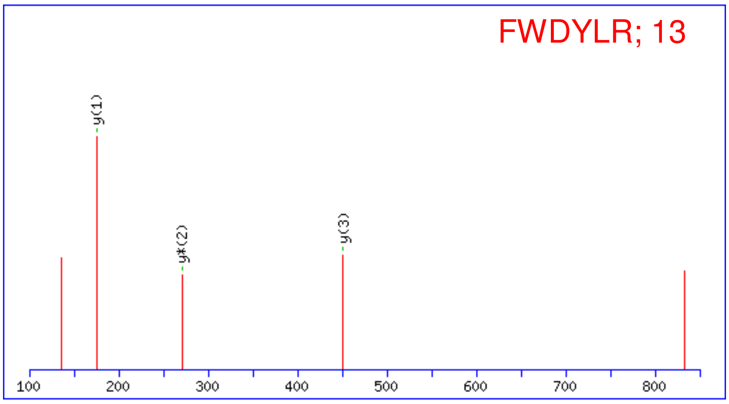

FWDYLR; 13
y(1)
y*(2)
y(3)

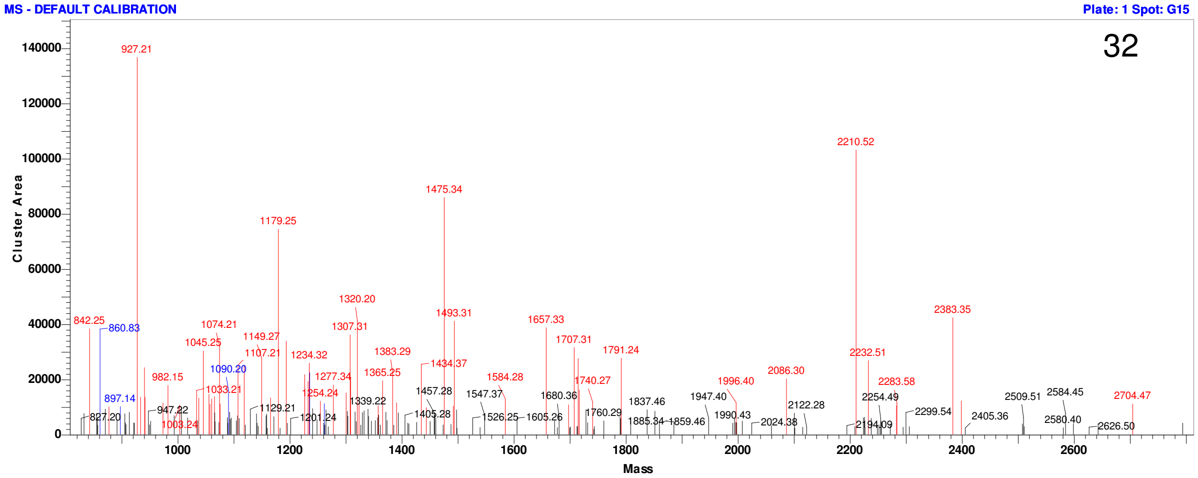
MS - DEFAULT CALIBRATION
Plate: 1 Spot: G15
32
Cluster Area
Mass

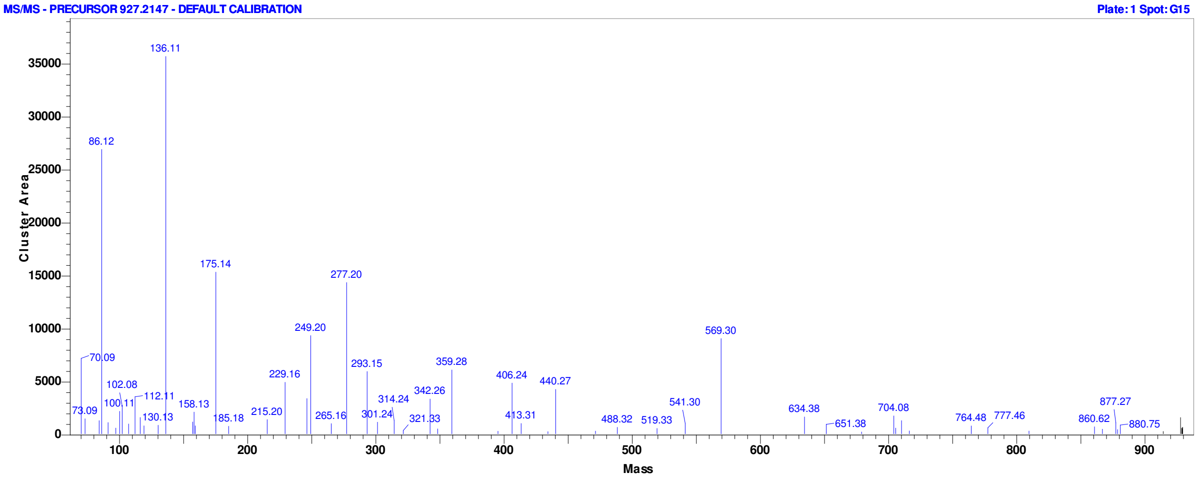
MS/MS - PRECURSOR 927.2147 - DEFAULT CALIBRATION
Plate: 1 Spot: G15
Cluster Area
Mass

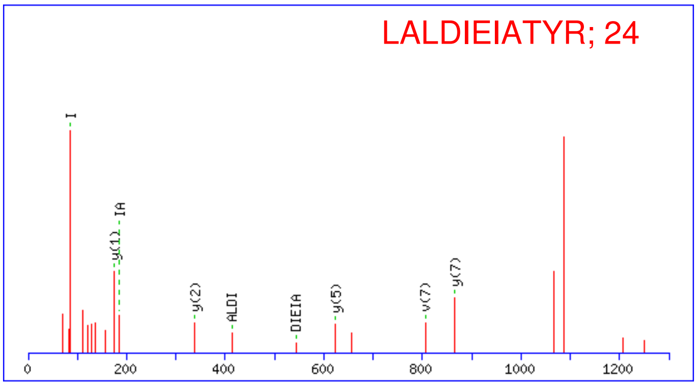
LALDIEIATYR; 24

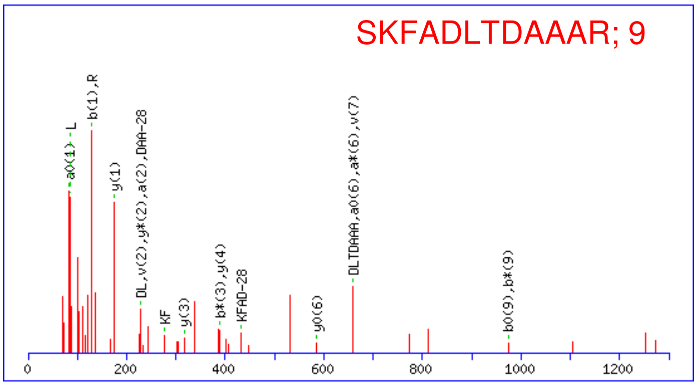
SKFADLTDAAAR; 9

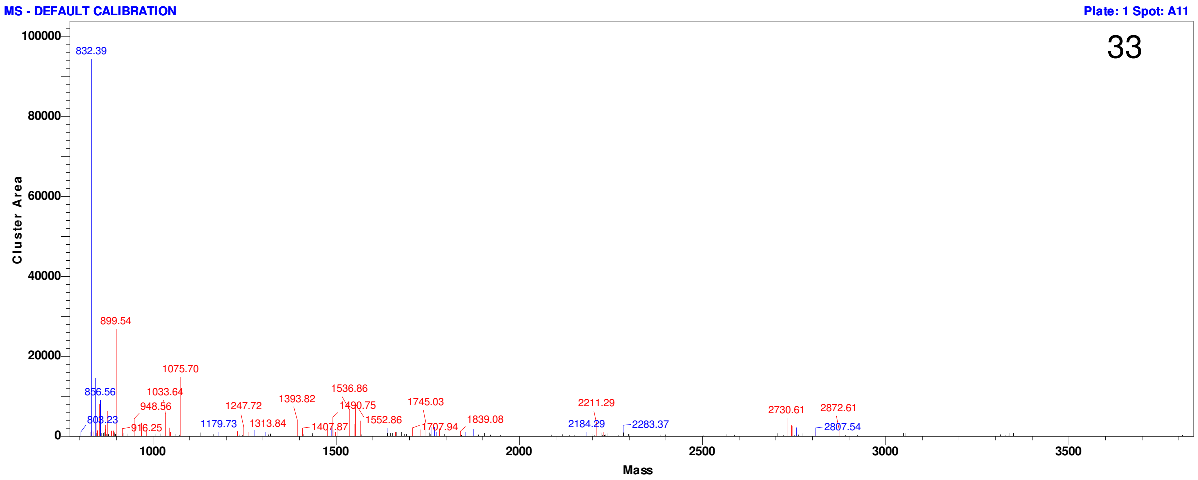
MS - DEFAULT CALIBRATION
Plate: 1 Spot: A11
33
Cluster Area
Mass
832.39
899.54
1075.70
856.56
1033.64
948.56
803.23
916.25
1179.73
1247.72
1313.84
1393.82
1407.87
1536.86
1490.75
1552.86
1745.03
1707.94
1839.08
2184.29
2211.29
2283.37
2730.61
2872.61
2807.54

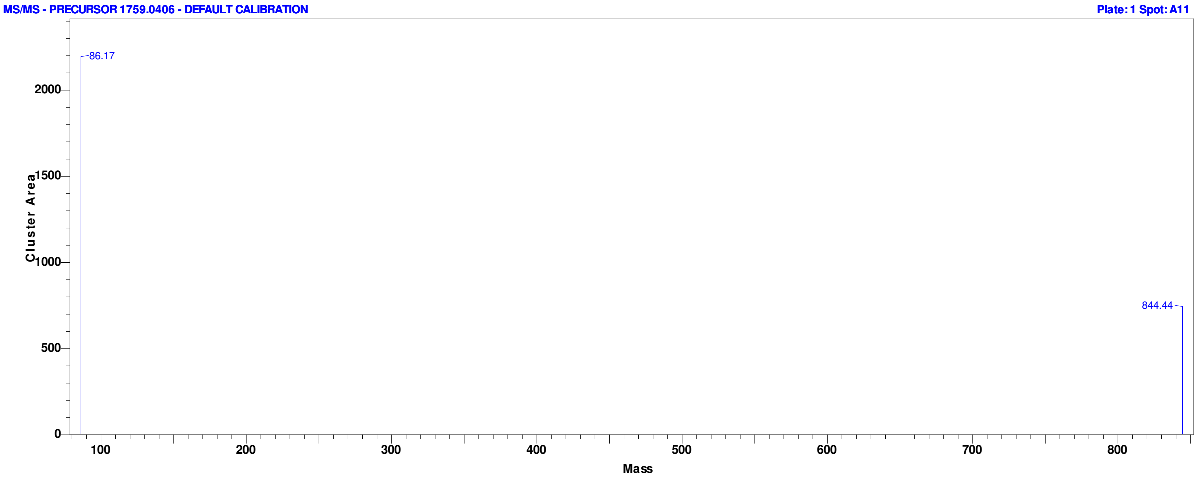
MS/MS - PRECURSOR 1759.0406 - DEFAULT CALIBRATION
Plate: 1 Spot: A11
Cluster Area
Mass
86.17
844.44

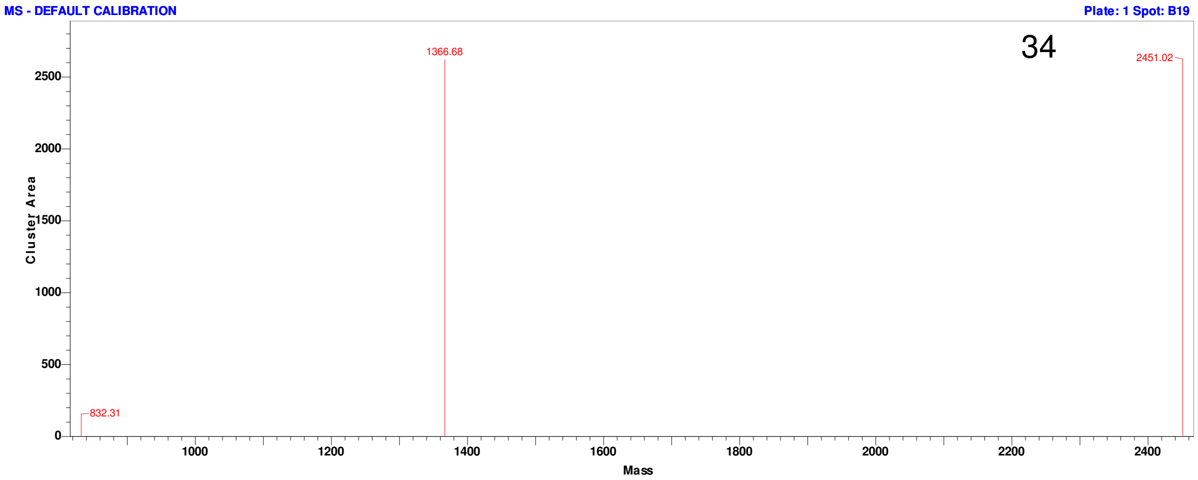
MS - DEFAULT CALIBRATION
Plate: 1 Spot: B19
34
Cluster Area
Mass
1366.68
2451.02
832.31

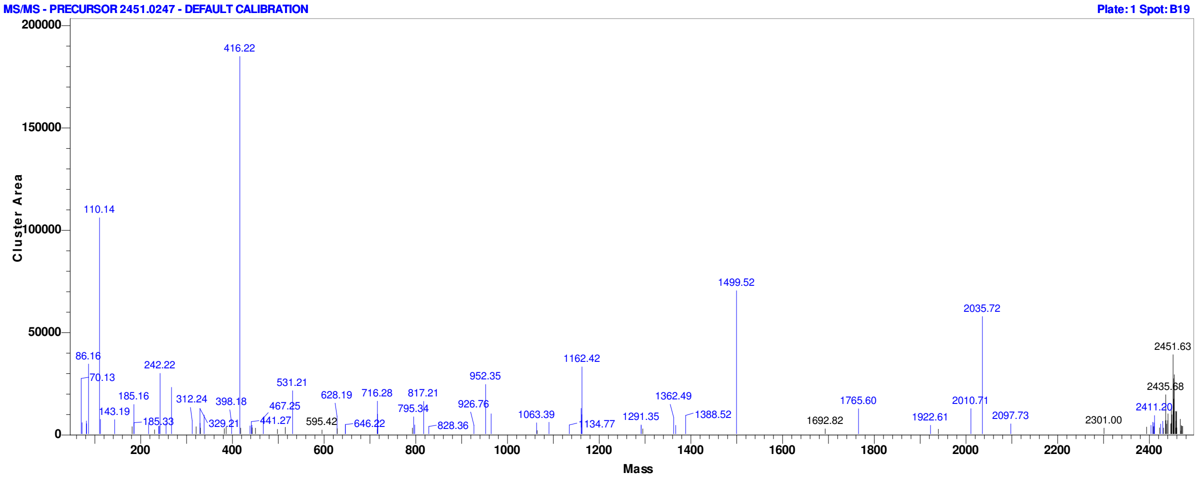
MS/MS - PRECURSOR 2451.0247 - DEFAULT CALIBRATION
Plate: 1 Spot: B19
Cluster Area
Mass
416.22
110.14
1499.52
2035.72
1162.42
2451.63

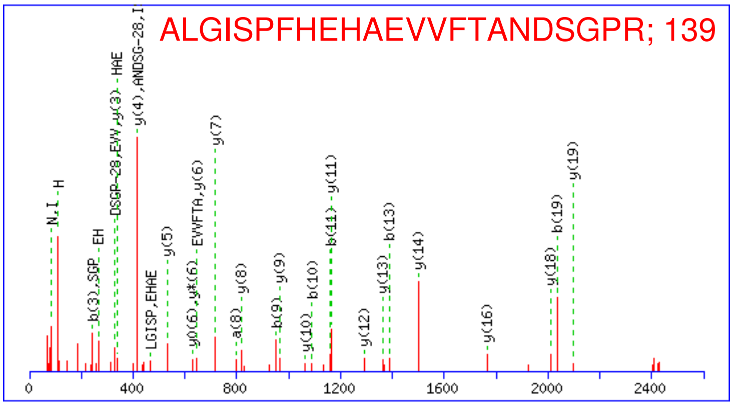
ALGISPFHEHAEVVFTANDSGPR; 139

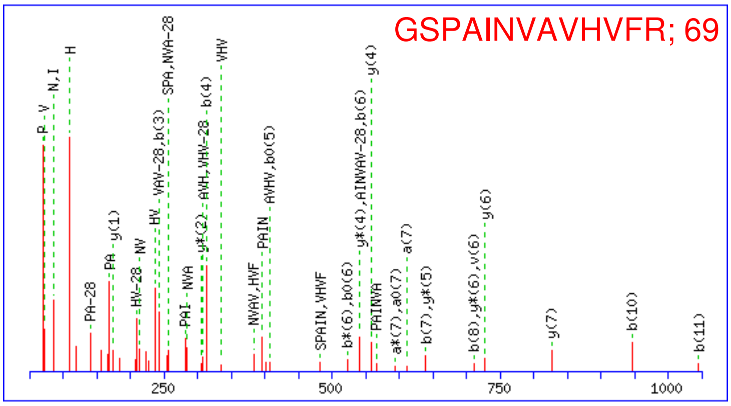
GSPAINVAVHVFR; 69

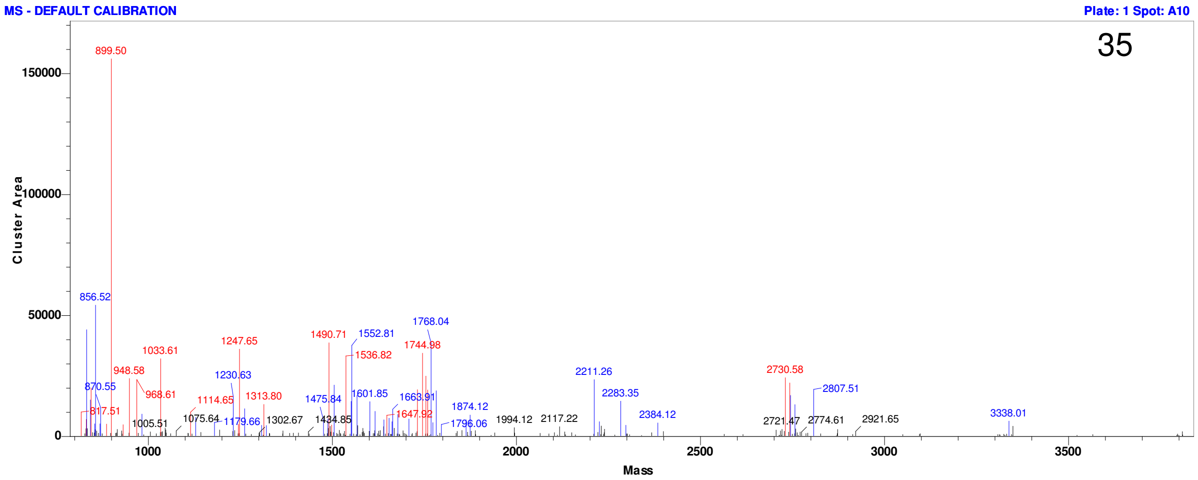
MS - DEFAULT CALIBRATION
Plate: 1 Spot: A10
35
Cluster Area
Mass
899.50
856.52
870.55
817.51
948.58
968.61
1033.61
1005.51
1075.64
1114.65
1179.66
1230.63
1247.65
1313.80
1302.67
1475.84
1434.85
1490.71
1552.81
1536.82
1601.85
1663.91
1647.92
1744.98
1768.04
1874.12
1796.06
1994.12
2117.22
2211.26
2283.35
2384.12
2730.58
2807.51
2721.47
2774.61
2921.65
3338.01

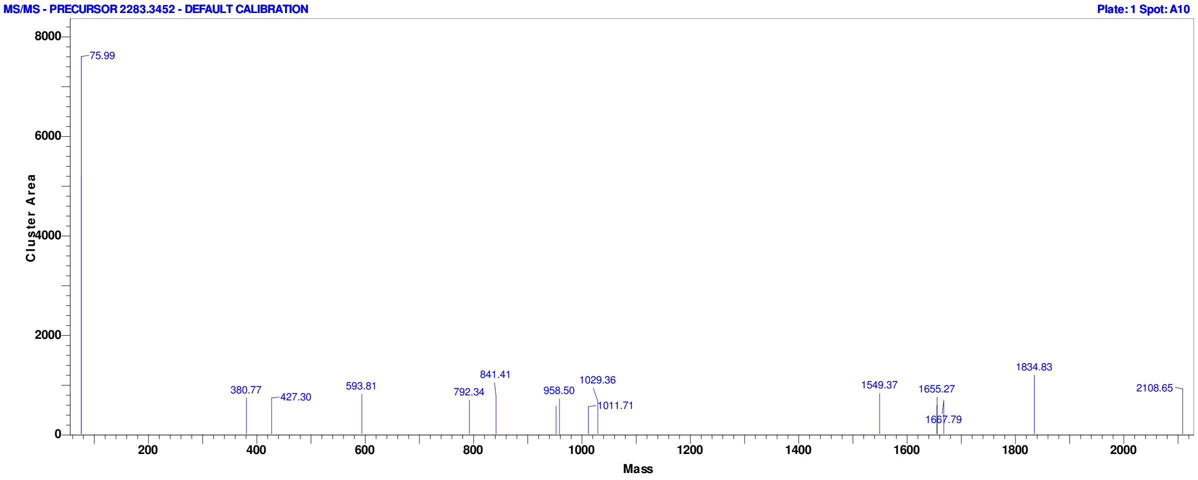
MS/MS - PRECURSOR 2283.3452 - DEFAULT CALIBRATION
Plate: 1 Spot: A10
Cluster Area
Mass
75.99
380.77
427.30
593.81
792.34
841.41
958.50
1029.36
1011.71
1549.37
1655.27
1667.79
1834.83
2108.65

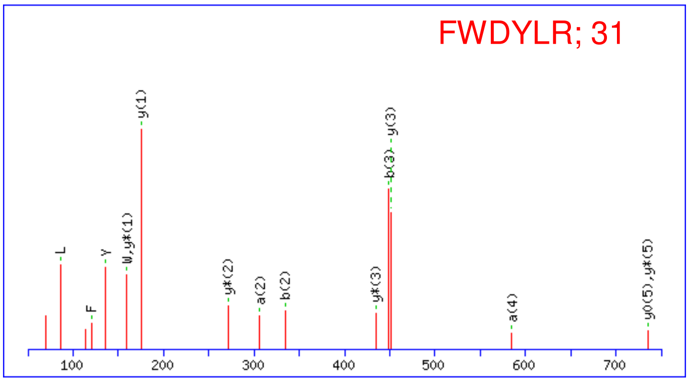
FWDYLR; 31
L
F
Y
W,y*(1)
y(1)
y*(2)
a(2)
b(2)
y*(3)
b(3)
y(3)
a(4)
y0(5),y*(5)

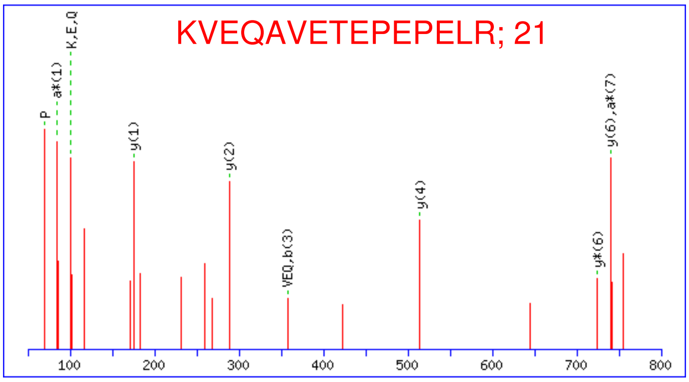
KVEQAVETEPEPELR; 21
P
a*(1)
K,E,Q
y(1)
y(2)
VEQ,b(3)
y(4)
y*(6)
y(6),a*(7)

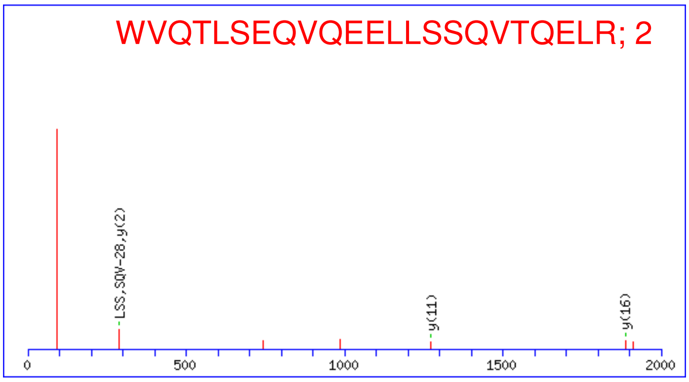
WVQTLSEQVQEELLSSQVTQELR; 2
LSS,SQW-28,y(2)
y(11)
y(16)

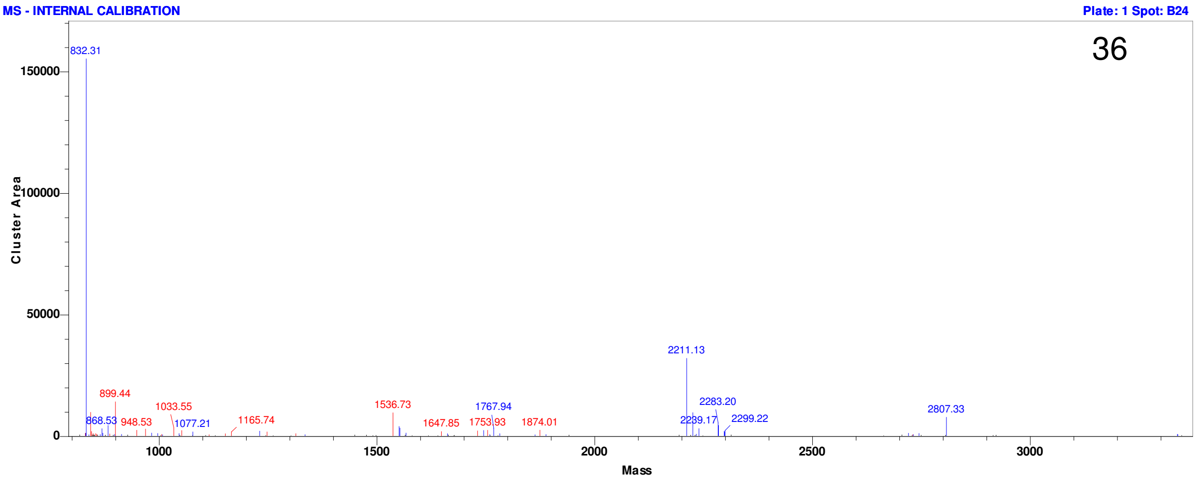
MS - INTERNAL CALIBRATION
Plate: 1 Spot: B24
36
Cluster Area
Mass
832.31
899.44
868.53
948.53
1033.55
1077.21
1165.74
1536.73
1647.85
1767.94
1753.93
1874.01
2211.13
2283.20
2239.17
2299.22
2807.33

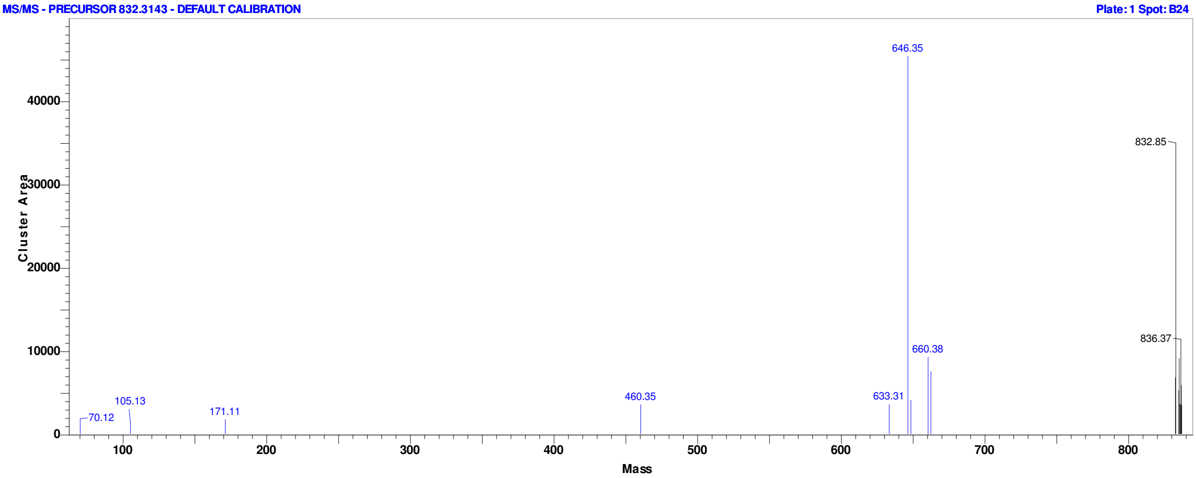
MS/MS - PRECURSOR 832.3143 - DEFAULT CALIBRATION
Plate: 1 Spot: B24
Cluster Area
Mass
646.35
832.85
836.37
660.38
633.31
460.35
171.11
105.13
70.12

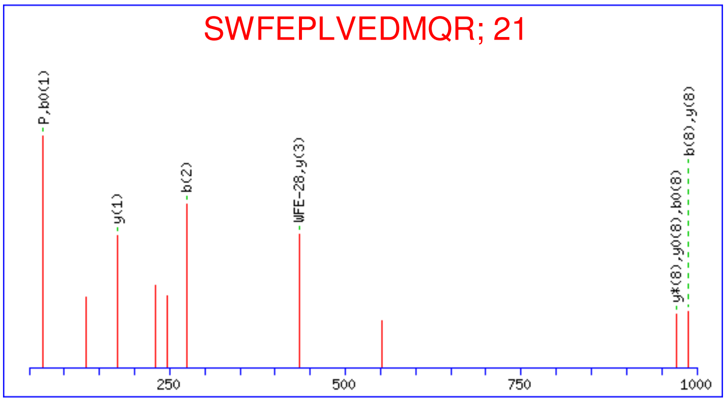
SWFEPLVEDMQR; 21
P,b0(1)
y(1)
b(2)
WFE-28,y(3)
y*(8),y0(8),b0(8)
b(8),y(8)

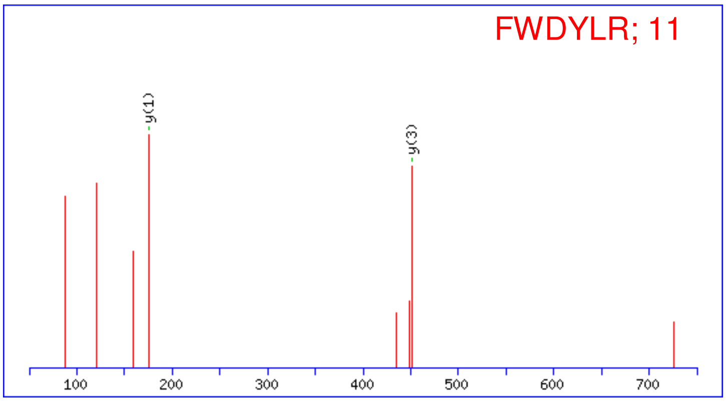
FWDYLR; 11
y(1)
y(3)

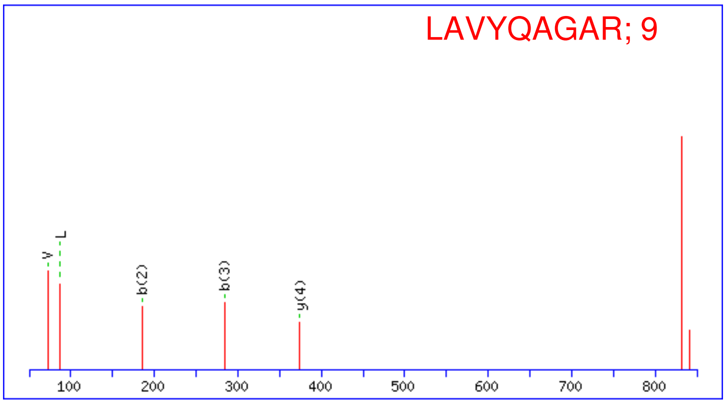
LAVYQAGAR; 9
V
L
b(2)
b(3)
y(4)

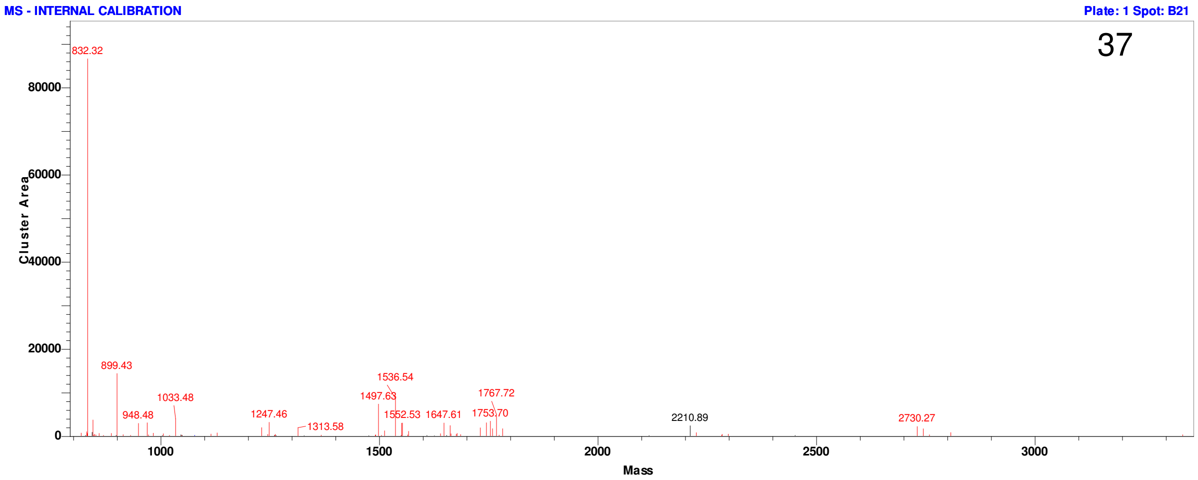
MS - INTERNAL CALIBRATION
Plate: 1 Spot: B21
37
Cluster Area
Mass
832.32
899.43
948.48
1033.48
1247.46
1313.58
1497.63
1536.54
1552.53
1647.61
1753.70
1767.72
2210.89
2730.27
0
20000
40000
60000
80000
1000
1500
2000
2500
3000

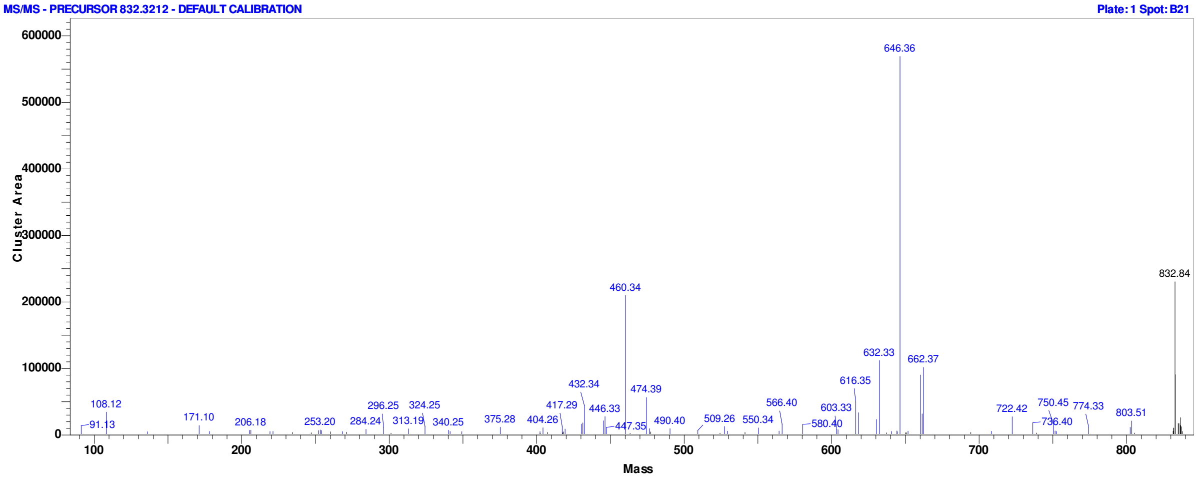
MS/MS - PRECURSOR 832.3212 - DEFAULT CALIBRATION
Plate: 1 Spot: B21
Cluster Area
Mass
646.36
832.84
460.34
632.33
662.37
616.35
432.34
474.39
446.33
447.35
417.29
404.26
375.28
340.25
324.25
313.19
296.25
284.24
253.20
206.18
171.10
108.12
91.13
490.40
509.26
550.34
566.40
580.40
603.33
722.42
750.45
736.40
774.33
803.51
0
100000
200000
300000
400000
500000
600000
100
200
300
400
500
600
700
800

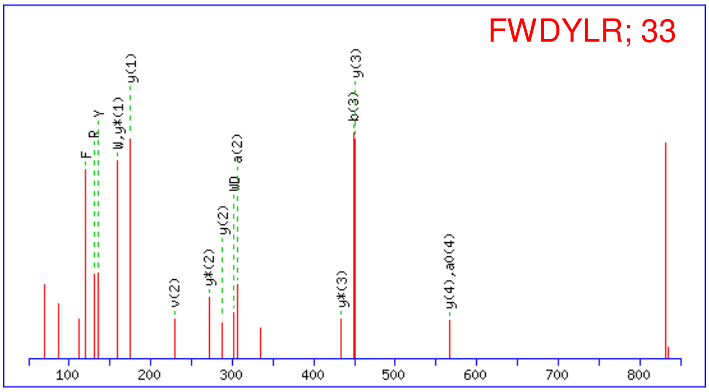
FWDYLR; 33
F
R
Y
W,y*(1)
y(1)
v(2)
y*(2)
y(2)
WD
a(2)
y*(3)
b(3)
y(3)
y(4),a0(4)
100
200
300
400
500
600
700
800

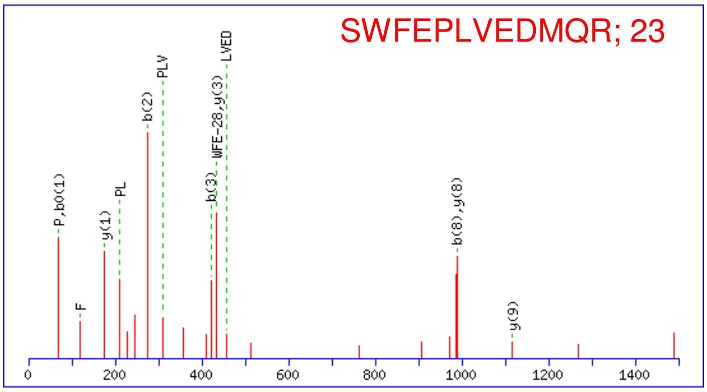
SWFEPLVEDMQR; 23
P,b0(1)
F
y(1)
PL
b(2)
PLV
b(3)
WFE-28,y(3)
LVED
b(8),y(8)
y(9)
0
200
400
600
800
1000
1200
1400

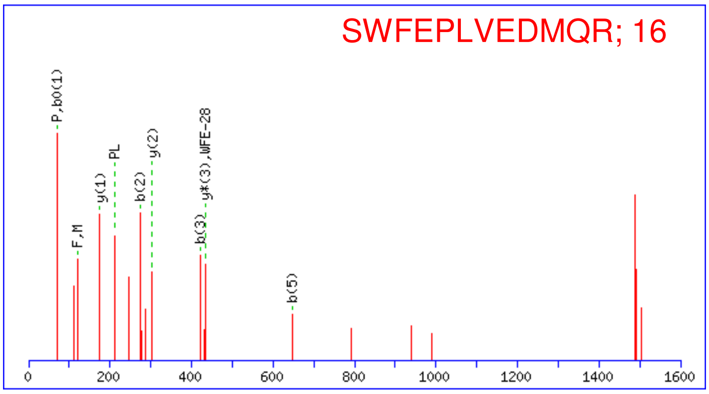
SWFEPLVEDMQR; 16
P,b0(1)
F,M
y(1)
PL
b(2)
y(2)
b(3)
y*(3),WFE-28
b(5)
0
200
400
600
800
1000
1200
1400
1600

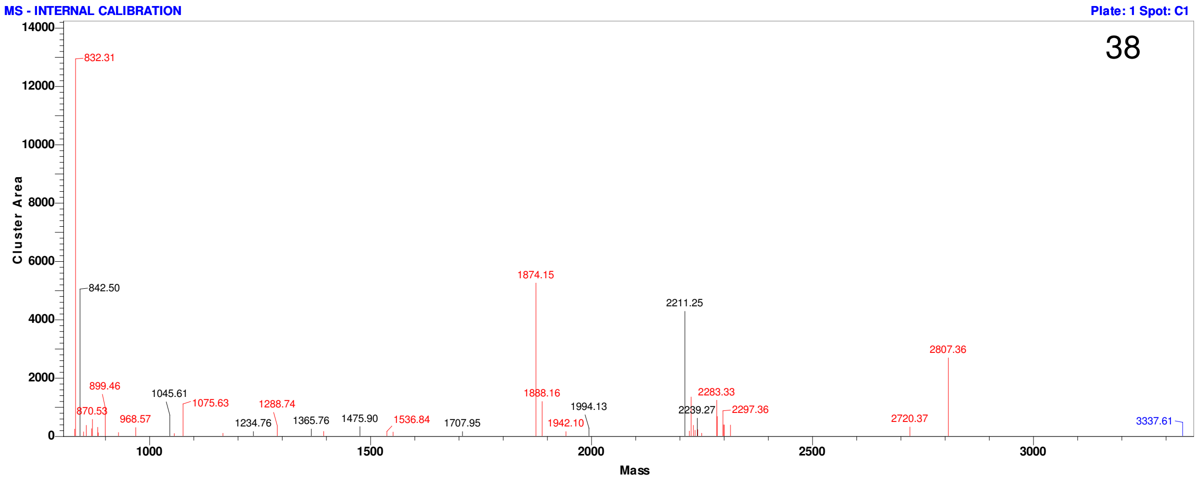

MS - INTERNAL CALIBRATION
Plate: 1 Spot: C1
38
Cluster Area
Mass
832.31
842.50
870.53
899.46
968.57
1045.61
1075.63
1234.76
1288.74
1365.76
1475.90
1536.84
1707.95
1874.15
1888.16
1942.10
1994.13
2211.25
2239.27
2283.33
2297.36
2720.37
2807.36
3337.61

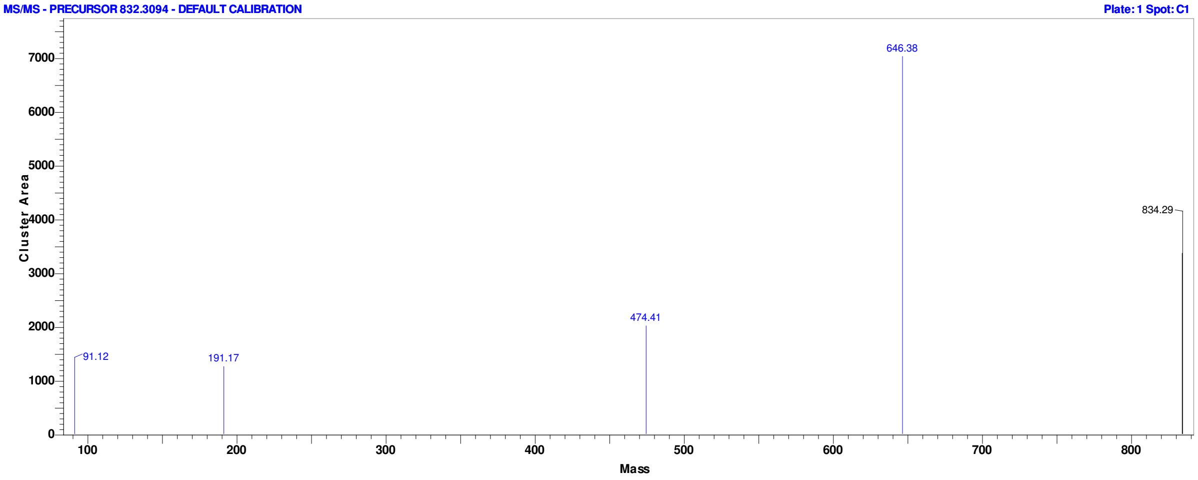

MS/MS - PRECURSOR 832.3094 - DEFAULT CALIBRATION
Plate: 1 Spot: C1
Cluster Area
Mass
91.12
191.17
474.41
646.38
834.29

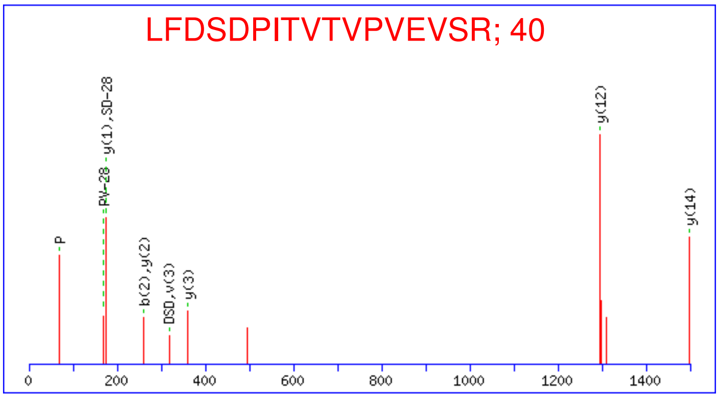

LFDSDPITVTVPVEVSR; 40
P
PV-28
y(1),SD-28
b(2),y(2)
DSD,y(3)
y(3)
y(12)
y(14)

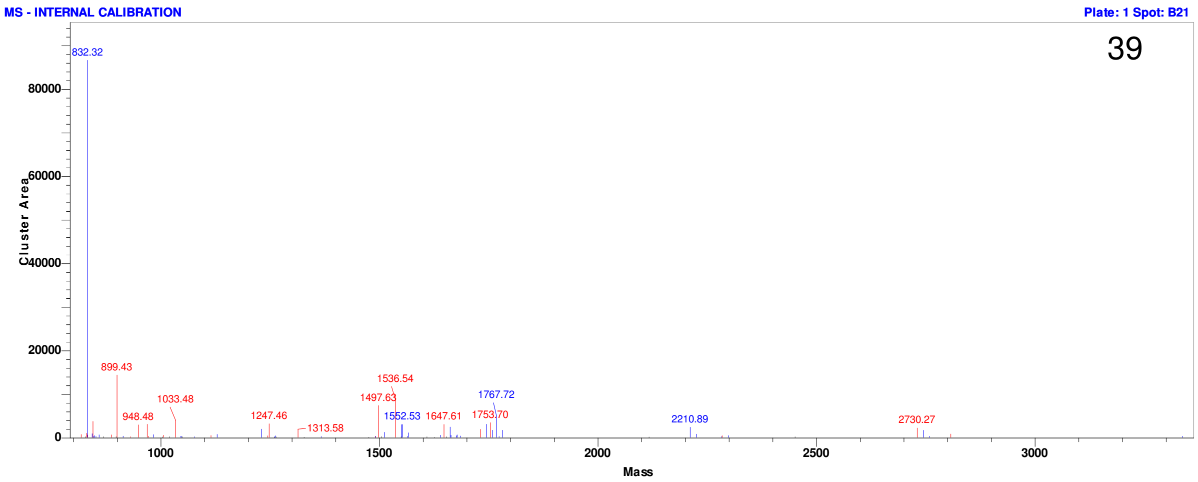
MS - INTERNAL CALIBRATION
Plate: 1 Spot: B21
39
Cluster Area
Mass
832.32
899.43
948.48
1033.48
1247.46
1313.58
1497.63
1536.54
1552.53
1647.61
1753.70
1767.72
2210.89
2730.27
0
20000
40000
60000
80000
1000
1500
2000
2500
3000

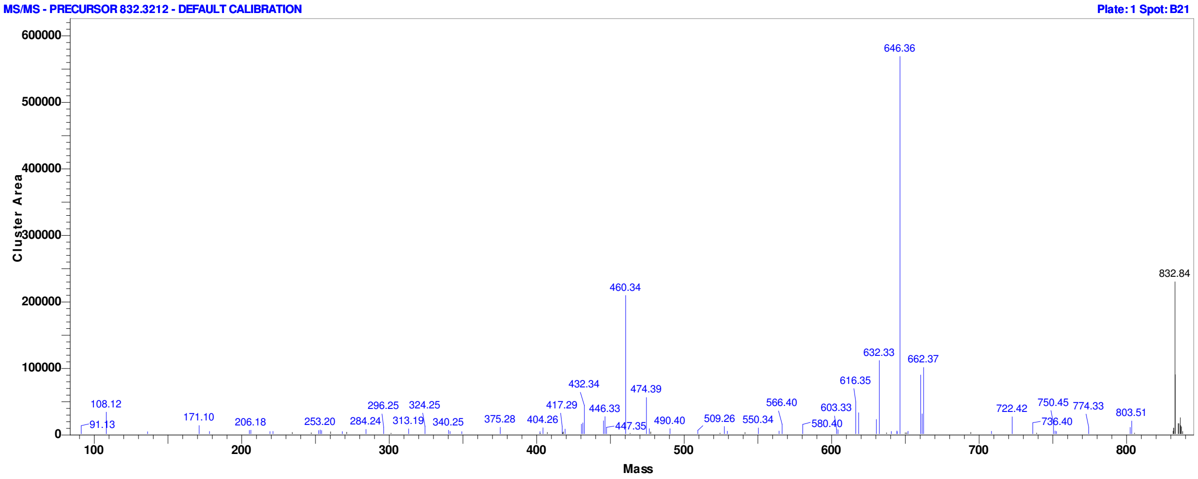
MS/MS - PRECURSOR 832.3212 - DEFAULT CALIBRATION
Plate: 1 Spot: B21
Cluster Area
Mass
646.36
832.84
460.34
632.33
662.37
616.35
432.34
474.39
446.33
417.29
108.12
91.13
171.10
206.18
253.20
284.24
296.25
313.19
324.25
340.25
375.28
404.26
447.35
490.40
509.26
550.34
566.40
580.40
603.33
722.42
736.40
750.45
774.33
803.51
0
100000
200000
300000
400000
500000
600000
100
200
300
400
500
600
700
800

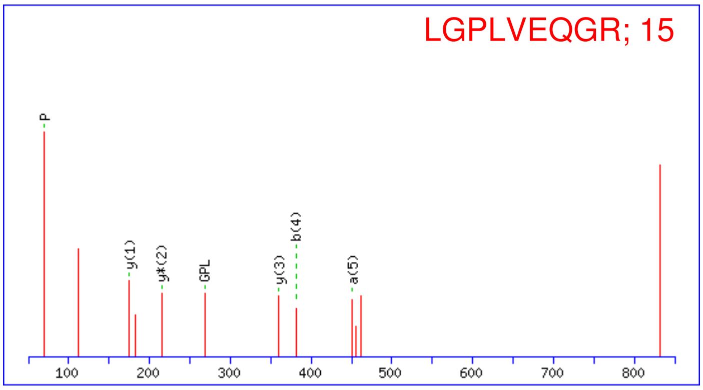
LGPLVEQGR; 15
P
y(1)
y*(2)
GPL
y(3)
b(4)
a(5)
100
200
300
400
500
600
700
800

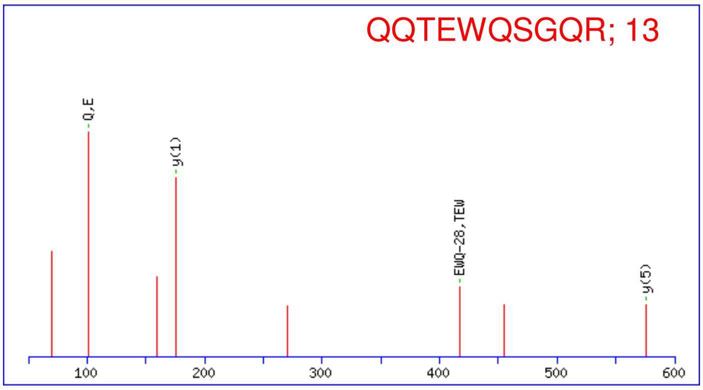
QQTEWQSGQR; 13
Q,E
y(1)
EWQ-28,TEW
y(5)
100
200
300
400
500
600

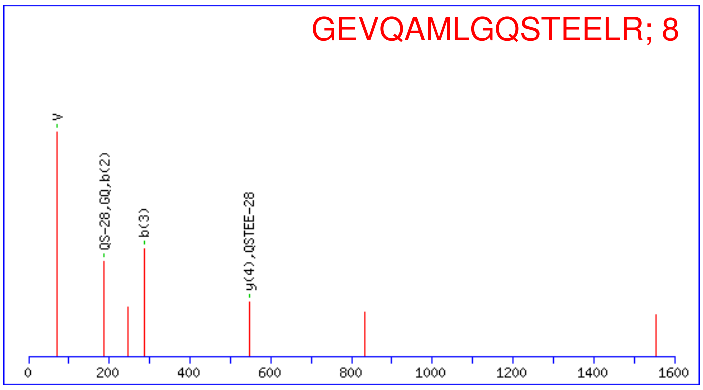
GEVQAMLGQSTEELR; 8
V
QS-28,GQ,b(2)
b(3)
y(4),QSTEE-28
0
200
400
600
800
1000
1200
1400
1600

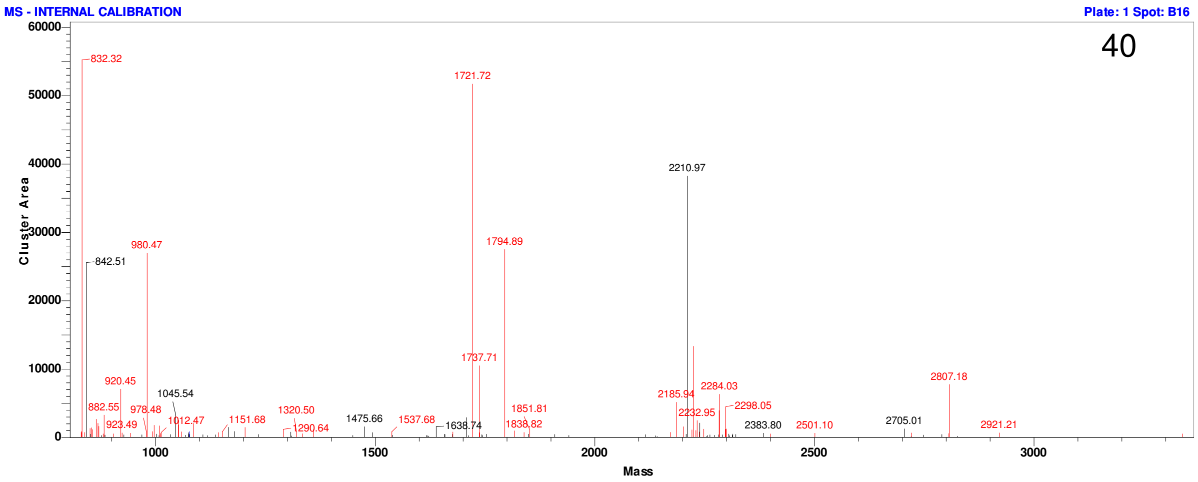
MS - INTERNAL CALIBRATION
Plate: 1 Spot: B16
40
Cluster Area
Mass
832.32
842.51
882.55
920.45
923.49
978.48
980.47
1012.47
1045.54
1151.68
1290.64
1320.50
1475.66
1537.68
1638.74
1721.72
1737.71
1794.89
1838.82
1851.81
2185.94
2210.97
2232.95
2284.03
2298.05
2383.80
2501.10
2705.01
2807.18
2921.21

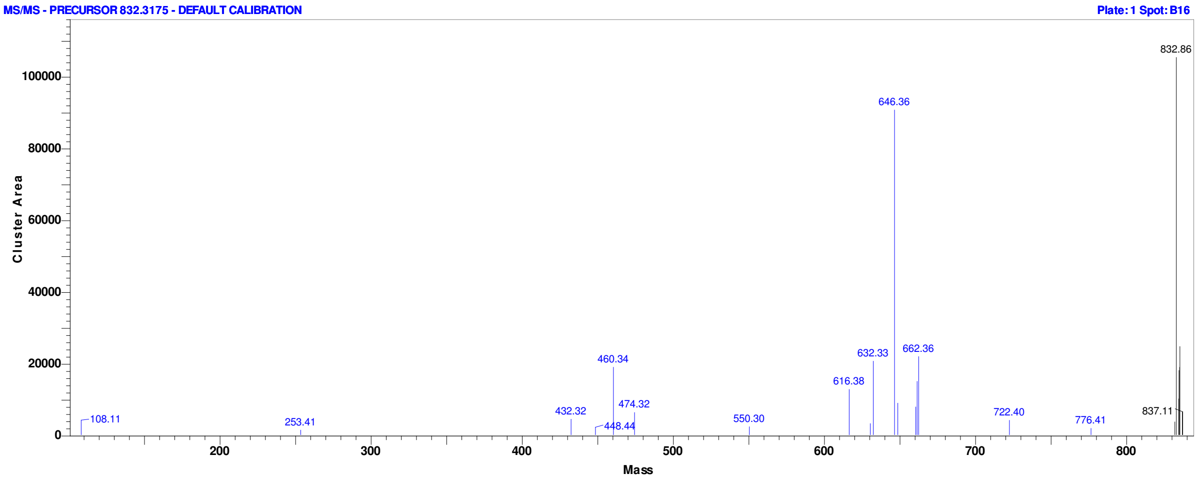
MS/MS - PRECURSOR 832.3175 - DEFAULT CALIBRATION
Plate: 1 Spot: B16
Cluster Area
Mass
108.11
253.41
432.32
448.44
460.34
474.32
550.30
616.38
632.33
646.36
662.36
722.40
776.41
832.86
837.11

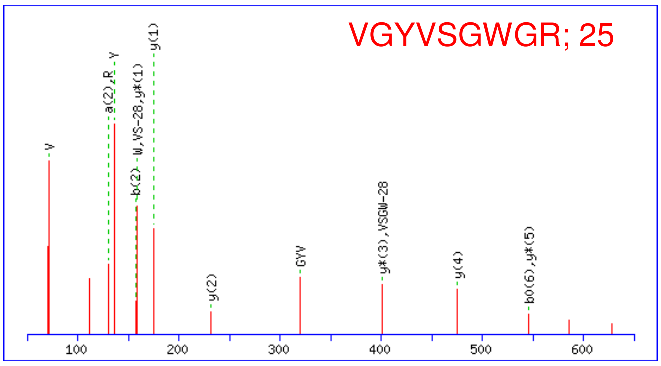
VGYVSGWGR; 25
V
a(2),R
Y
b(2)
W,VS-28,y*(1)
y(1)
y(2)
GYV
y*(3),VSGW-28
y(4)
b0(6),y*(5)

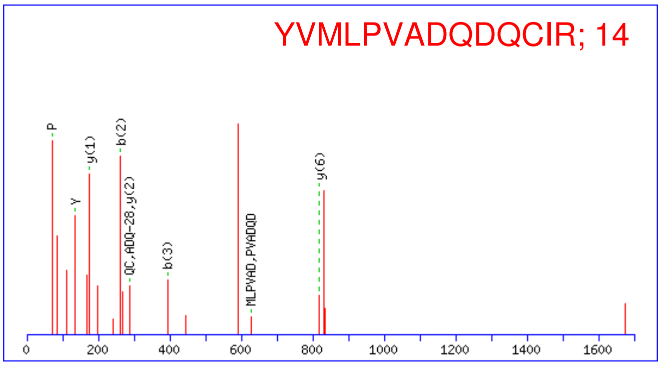
YVMLPVADQDQCIR; 14
P
Y
y(1)
b(2)
QC,ADQ-28,y(2)
b(3)
MLPVAD,PVADQD
y(6)

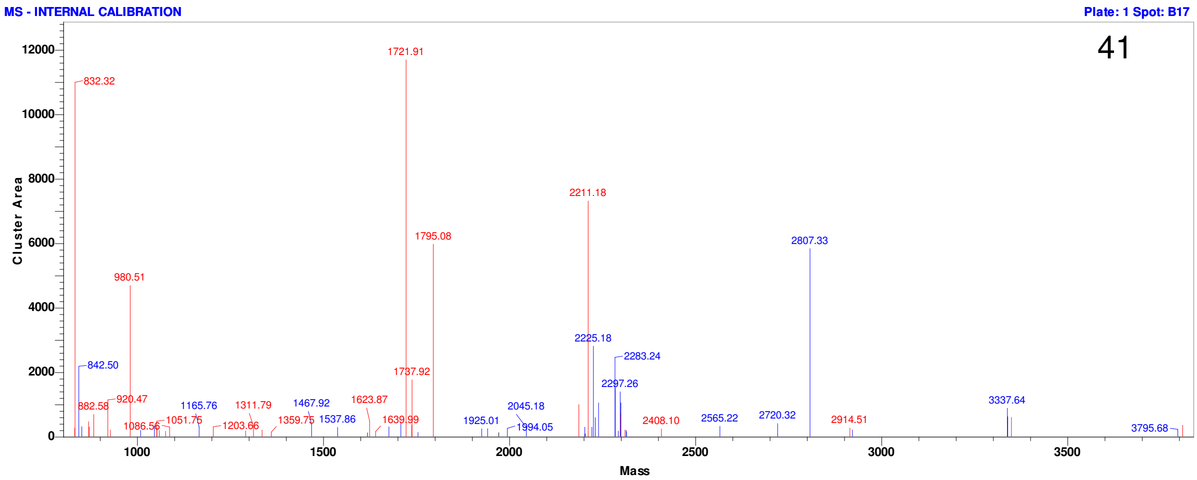
MS - INTERNAL CALIBRATION
Plate: 1 Spot: B17
41
Cluster Area
Mass
832.32
1721.91
2211.18
1795.08
2807.33
980.51
2225.18
2283.24
842.50
1737.92
2297.26
920.47
882.58
1165.76
1311.79
1467.92
1623.87
2045.18
3337.64
1051.75
1359.75
1537.86
1639.99
1925.01
2408.10
2565.22
2720.32
2914.51
1086.56
1203.66
1994.05
3795.68

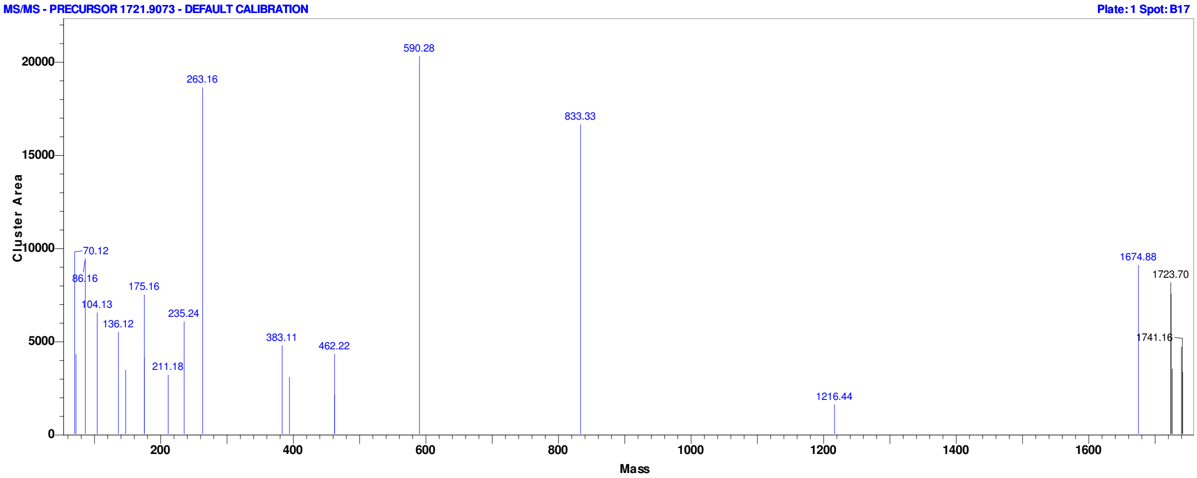
MS/MS - PRECURSOR 1721.9073 - DEFAULT CALIBRATION
Plate: 1 Spot: B17
Cluster Area
Mass
590.28
263.16
833.33
70.12
86.16
104.13
175.16
136.12
235.24
211.18
383.11
462.22
1216.44
1674.88
1723.70
1741.16

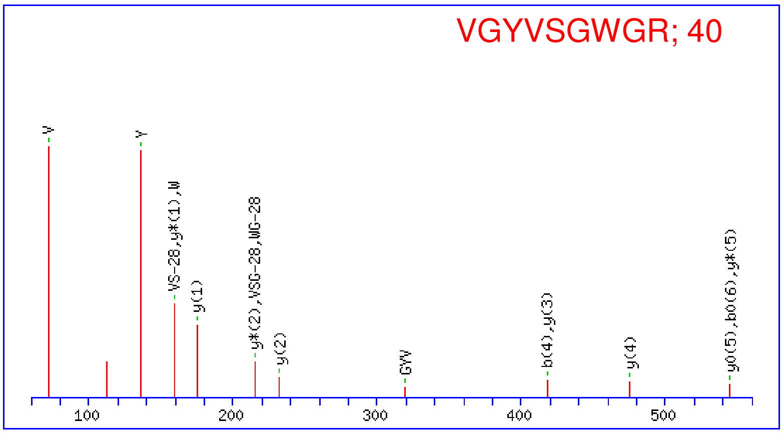
VGYVSGWGR; 40
V
Y
VS-28,y*(1),W
y(1)
y*(2),VSG-28,WG-28
y(2)
GYV
b(4),y(3)
y(4)
yO(5),bO(6),y*(5)

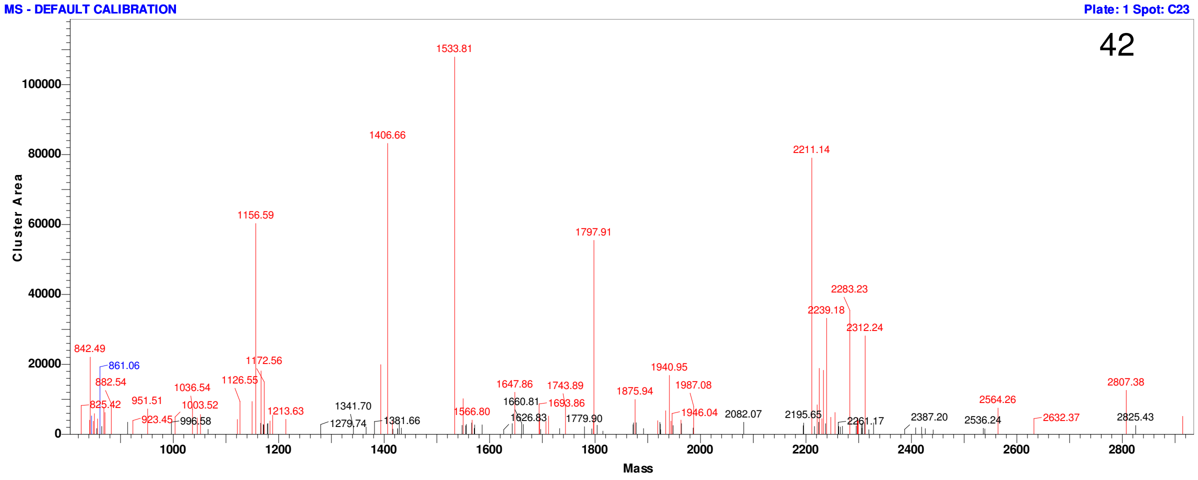
MS - DEFAULT CALIBRATION
Plate: 1 Spot: C23
42
Cluster Area
Mass
1533.81
1406.66
2211.14
1156.59
1797.91
2283.23
2239.18
2312.24
842.49
861.06
882.54
825.42
1172.56
1126.55
1036.54
951.51
1003.52
923.45
996.58
1213.63
1341.70
1279.74
1381.66
1566.80
1647.86
1660.81
1626.83
1743.89
1693.86
1779.90
1875.94
1940.95
1987.08
1946.04
2082.07
2195.65
2261.17
2387.20
2536.24
2564.26
2632.37
2807.38
2825.43

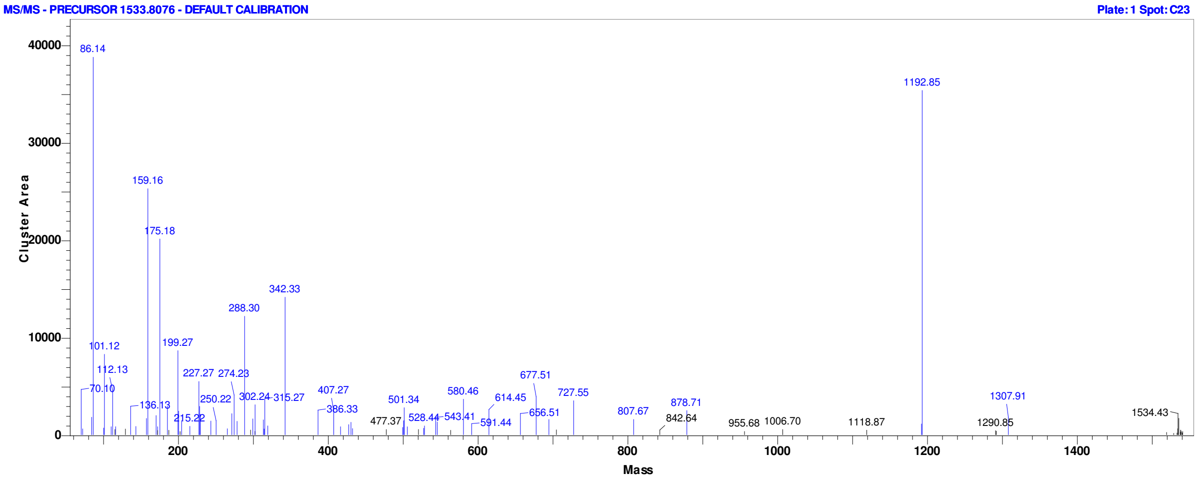
MS/MS - PRECURSOR 1533.8076 - DEFAULT CALIBRATION
Plate: 1 Spot: C23
Cluster Area
Mass
86.14
1192.85
159.16
175.18
342.33
288.30
199.27
101.12
112.13
70.10
227.27
274.23
250.22
302.24
315.27
136.13
215.22
407.27
386.33
477.37
501.34
528.44
543.41
580.46
591.44
614.45
677.51
656.51
727.55
807.67
842.64
878.71
955.68
1006.70
1118.87
1290.85
1307.91
1534.43

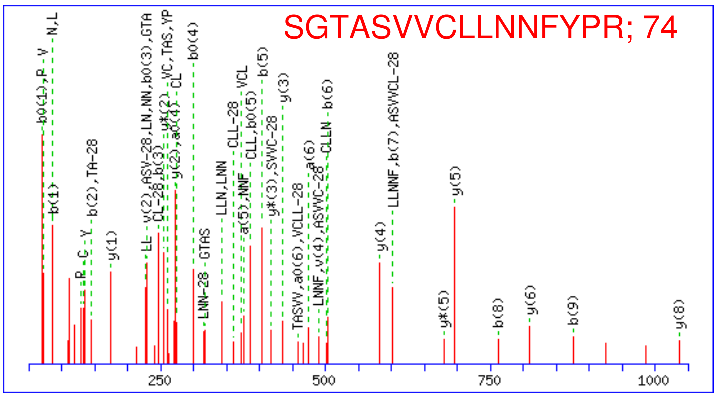
SGTASVVCLLNNFYPR; 74
bo(1),P-V
N,L
b(1)
P
G
Y
b(2),TA-28
y(1)
LL
y(2),ASV-28,LN,NN,bo(3),GTA
CL-28
b(3)
y*(2)
VC,TAS,YP
y(2),ao(4)
CL
bo(4)
LNN-28
GTAS
LLN,LNN
CLL-28
VCL
a(5),NNF
CLL,bo(5)
b(5)
y*(3),SVVC-28
y(3)
TASVV,ao(6),VCLL-28
LNNF,y(4),ASVVC-28
a(6)
CLLN
b(6)
y(4)
LLNNF,b(7),ASVVCL-28
y*(5)
y(5)
b(8)
y(6)
b(9)
y(8)
250
500
750
1000

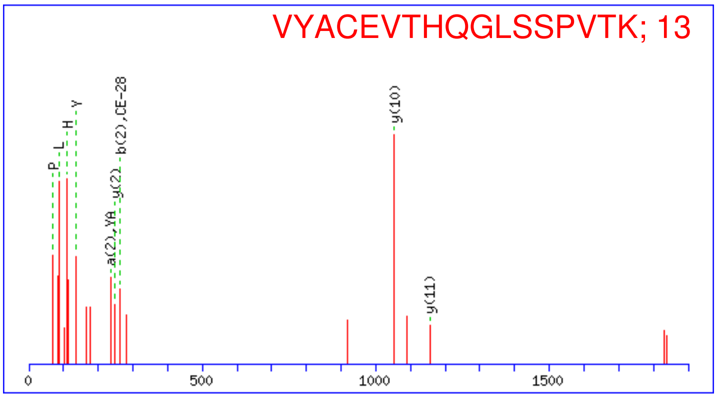
VYACEVTHQGLSSPVTK; 13
P
L
H
Y
a(2),YA
y(2)
b(2),CE-28
y(10)
y(11)
0
500
1000
1500

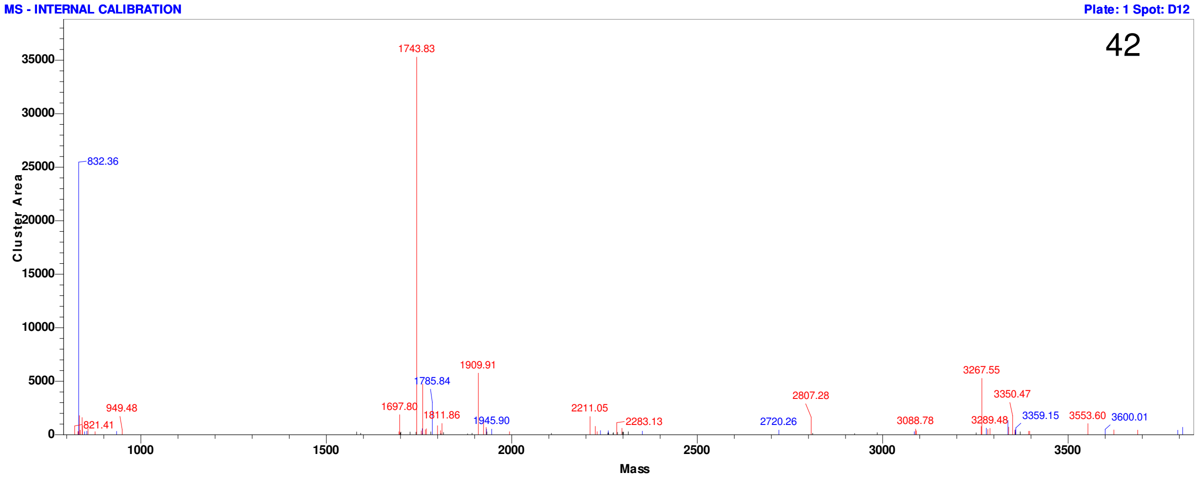
MS - INTERNAL CALIBRATION
Plate: 1 Spot: D12
42
Cluster Area
Mass
35000
30000
25000
20000
15000
10000
5000
0
1000
1500
2000
2500
3000
3500
1743.83
832.36
821.41
949.48
1697.80
1785.84
1811.86
1909.91
1945.90
2211.05
2283.13
2720.26
2807.28
3088.78
3267.55
3289.48
3350.47
3359.15
3553.60
3600.01

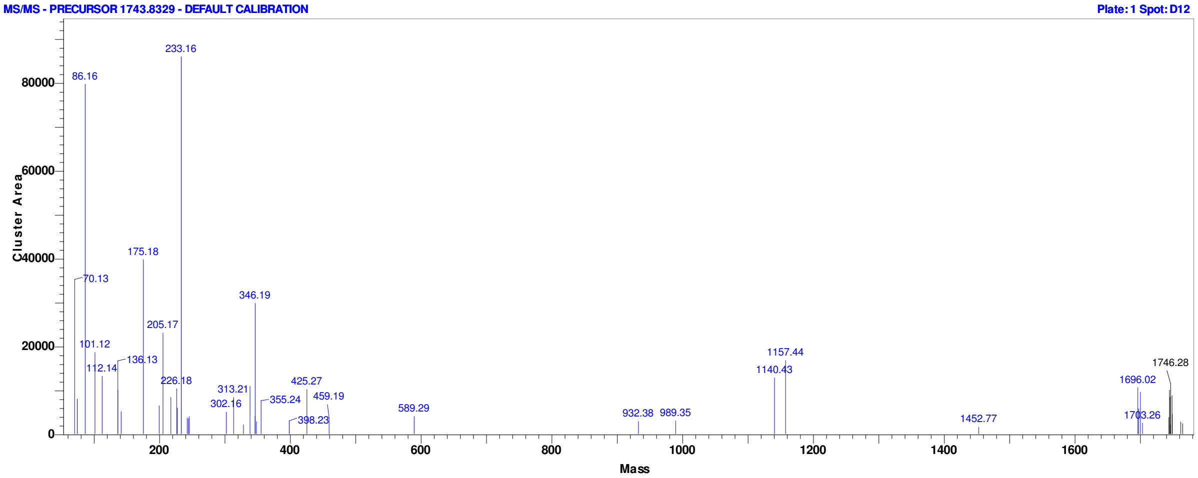
MS/MS - PRECURSOR 1743.8329 - DEFAULT CALIBRATION
Plate: 1 Spot: D12
Cluster Area
Mass
80000
60000
40000
20000
0
200
400
600
800
1000
1200
1400
1600
233.16
86.16
175.18
70.13
346.19
205.17
101.12
112.14
136.13
226.18
313.21
302.16
355.24
398.23
425.27
459.19
589.29
932.38
989.35
1140.43
1157.44
1452.77
1696.02
1703.26
1746.28

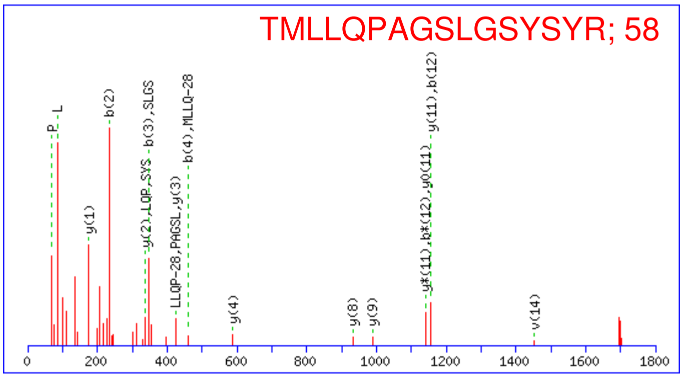
TMLLQPAGSLGSYSYR; 58
P
L
b(2)
y(1)
y(2),LQP,SYS
b(3),SLGS
LLQP-28,PAGSL,y(3)
b(4),MLLQ-28
y(4)
y(8)
y(9)
y*(11),b*(12),y0(11)
y(11),b(12)
y(14)
0
200
400
600
800
1000
1200
1400
1600
1800

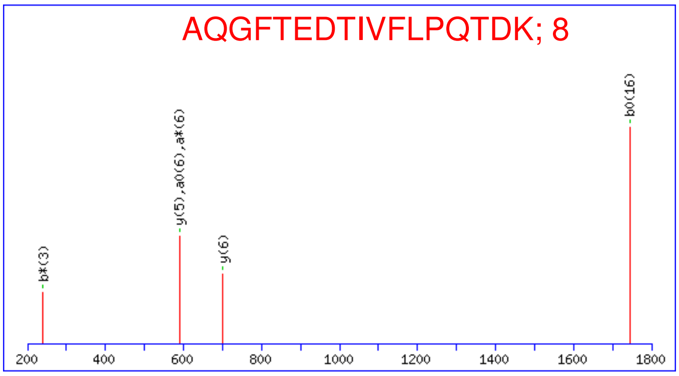
AQGFTEDTIVFLPQTDK; 8
b*(3)
y(5),a0(6),a*(6)
y(6)
b0(16)
200
400
600
800
1000
1200
1400
1600
1800

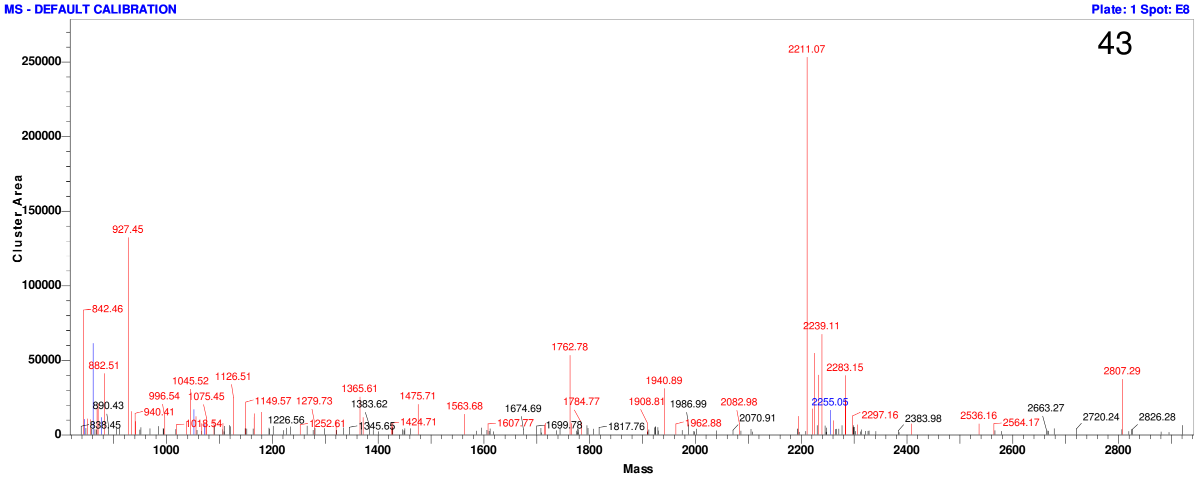
MS - DEFAULT CALIBRATION
Plate: 1 Spot: E8
43
Cluster_Area
Mass
2211.07
927.45
842.46
882.51
890.43
838.45
940.41
996.54
1045.52
1075.45
1018.54
1126.51
1149.57
1226.56
1279.73
1252.61
1365.61
1383.62
1345.65
1475.71
1424.71
1563.68
1674.69
1607.77
1762.78
1784.77
1699.78
1817.76
1940.89
1908.81
1986.99
1962.88
2082.98
2070.91
2239.11
2283.15
2255.05
2297.16
2383.98
2536.16
2564.17
2663.27
2720.24
2807.29
2826.28

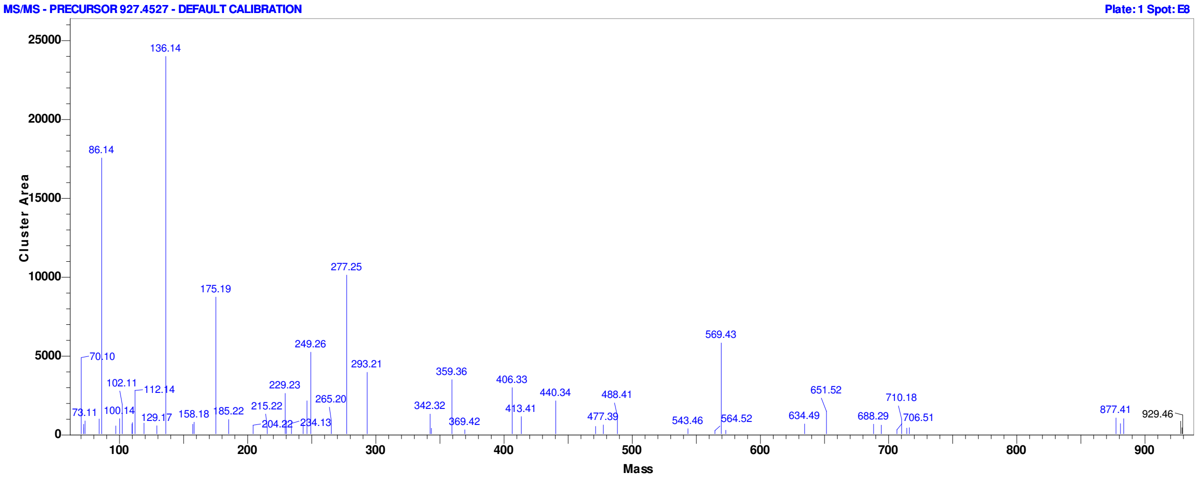
MS/MS - PRECURSOR 927.4527 - DEFAULT CALIBRATION
Plate: 1 Spot: E8
Cluster Area
Mass
136.14
86.14
70.10
73.11
102.11
100.14
112.14
129.17
158.18
175.19
185.22
215.22
204.22
229.23
234.13
249.26
265.20
277.25
293.21
342.32
359.36
369.42
406.33
413.41
440.34
477.39
488.41
543.46
569.43
564.52
634.49
651.52
688.29
710.18
706.51
877.41
929.46

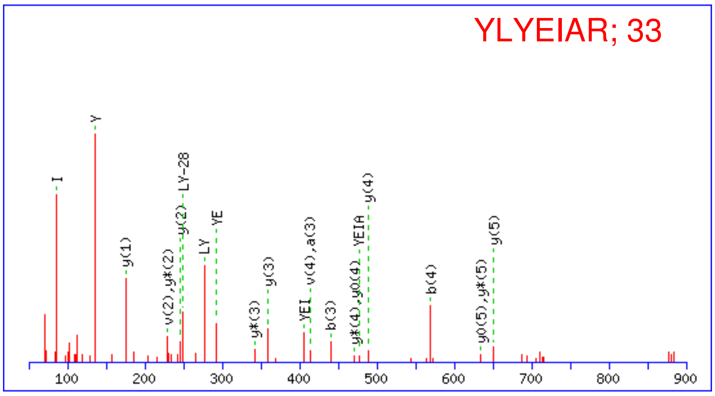
YLYEIAR; 33

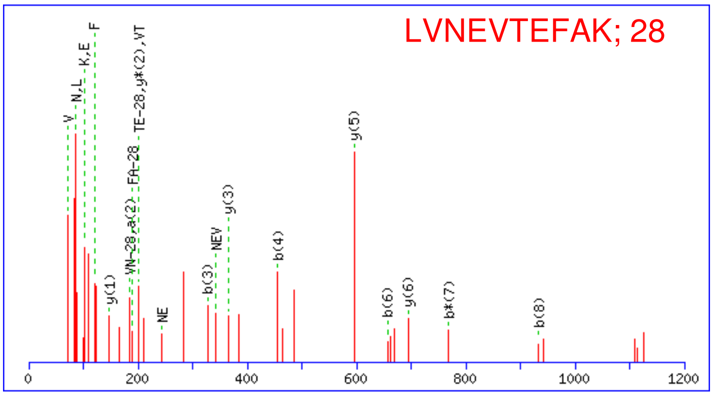
LVNEVTEFAK; 28

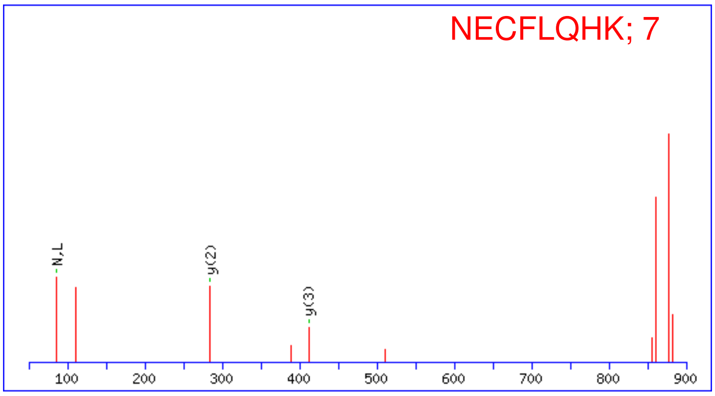
NECFLQHK; 7

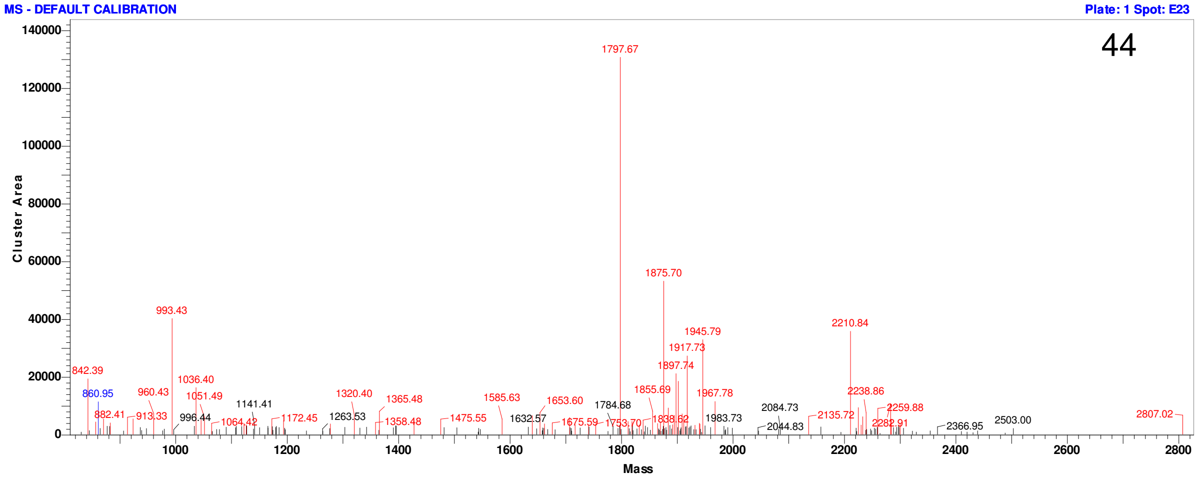

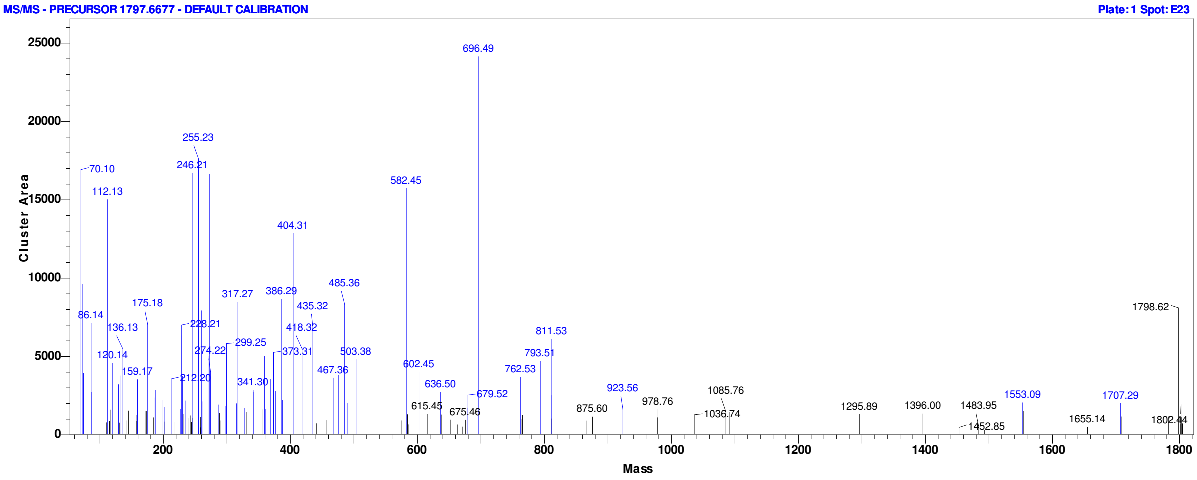

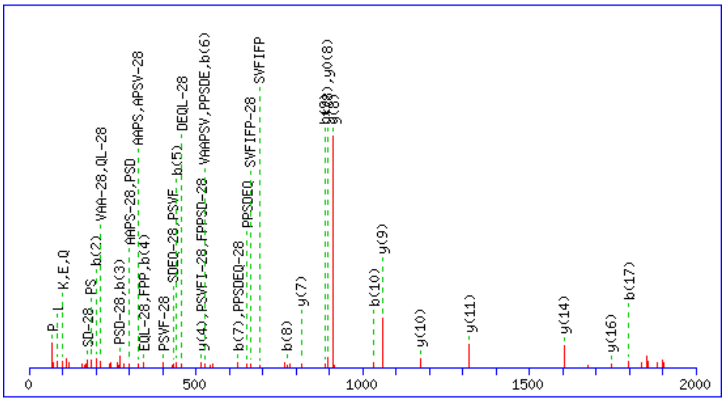

TVAAPSVFIFPPSDEQLK; 81

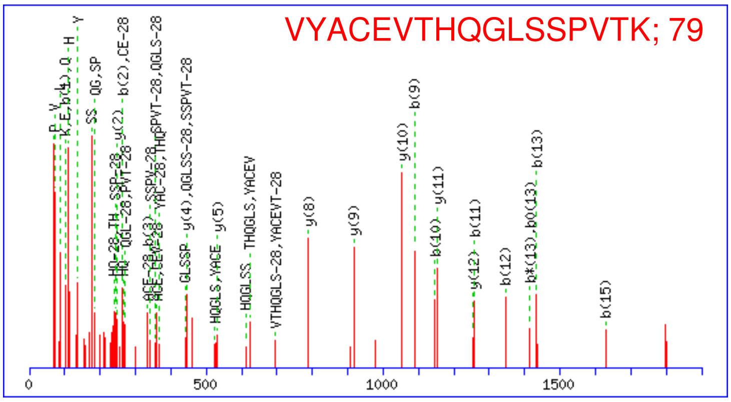

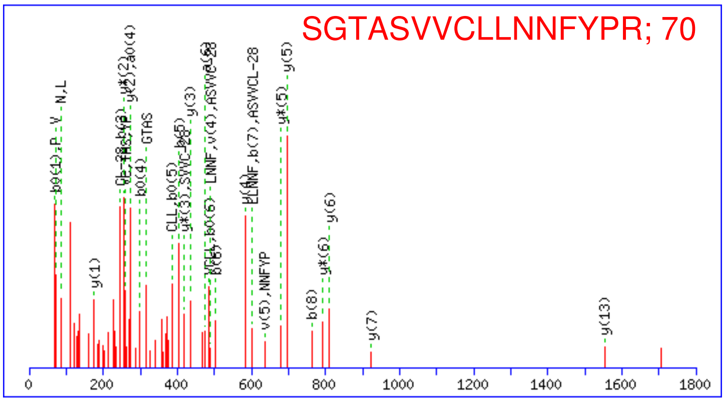

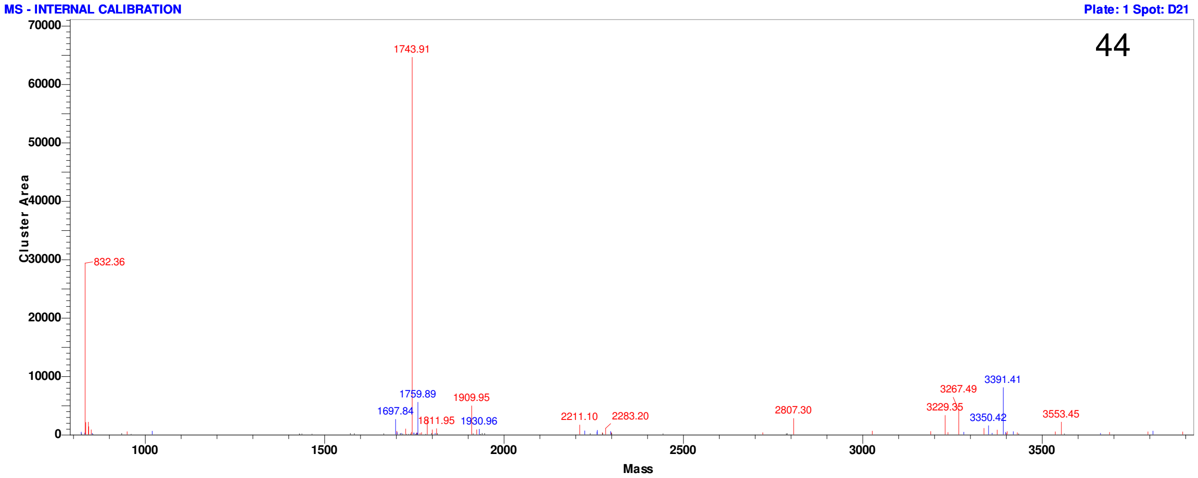

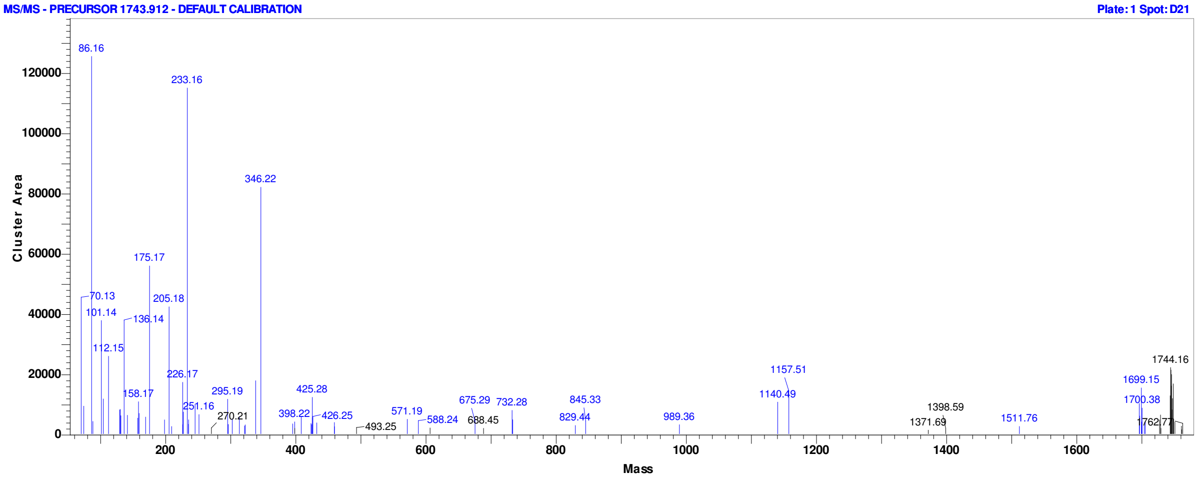

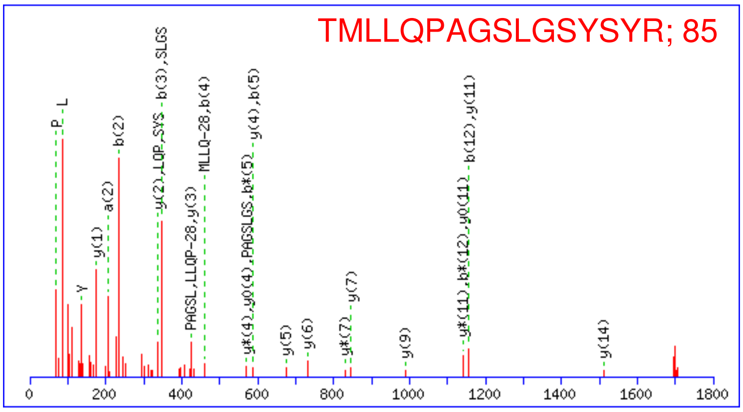

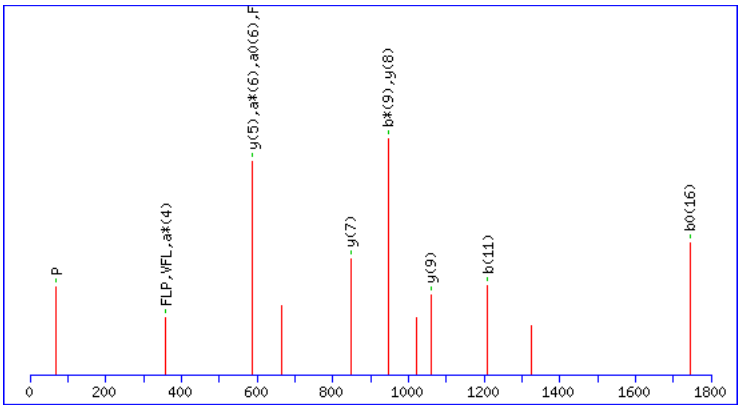

AQGFTEDTIVFLPQTDK; 17

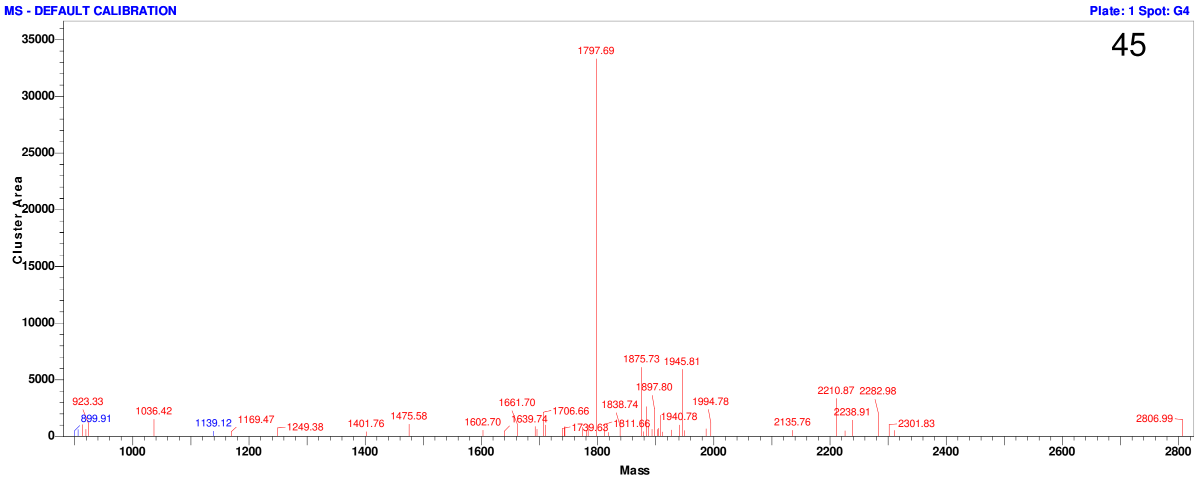

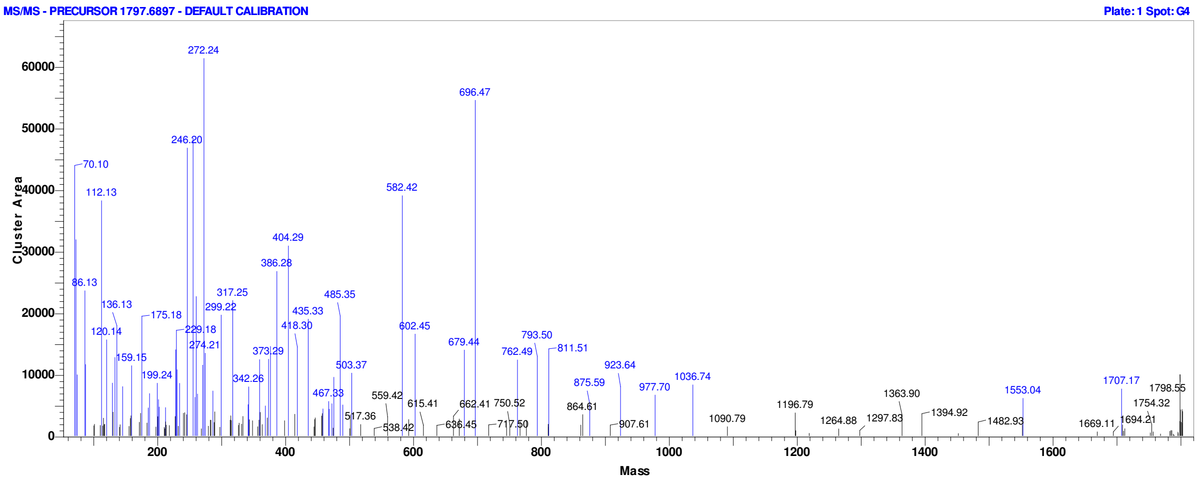

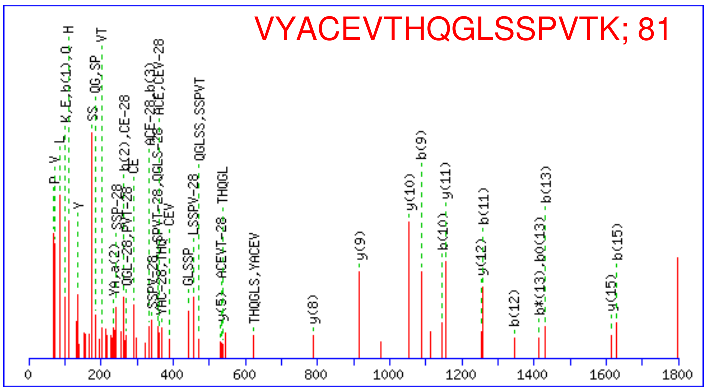

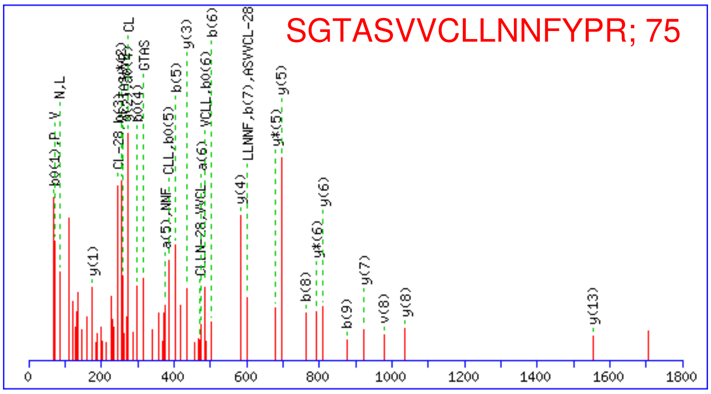

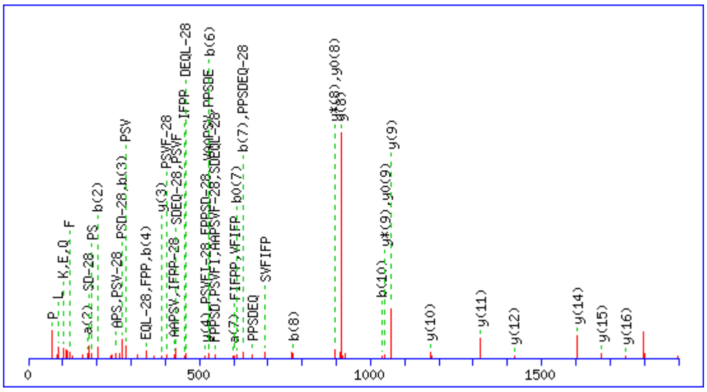

TVAAPSVFIFPPSDEQLK; 65

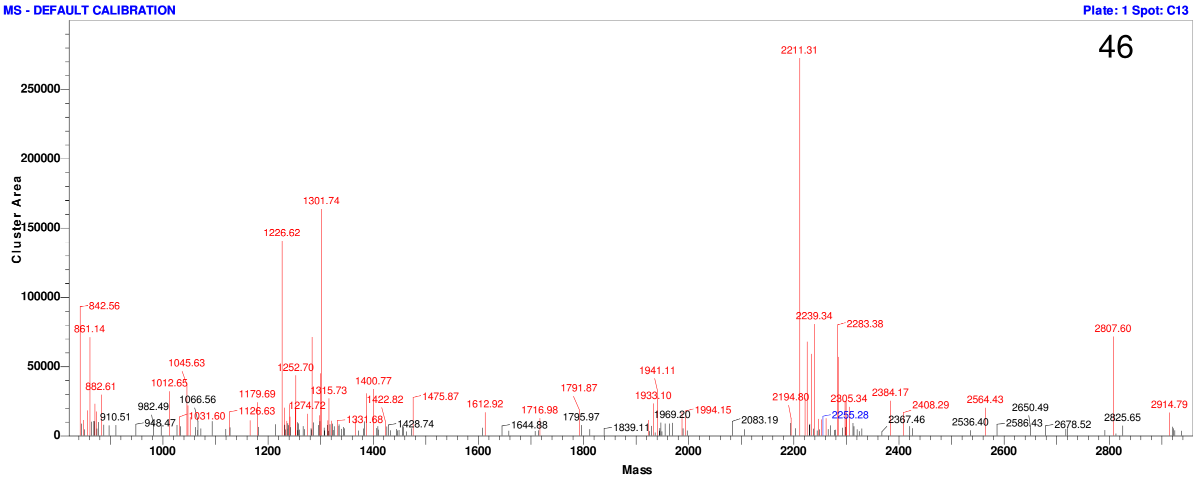
MS - DEFAULT CALIBRATION
Plate: 1 Spot: C13
46
Cluster Area
Mass
2211.31
1301.74
1226.62
842.56
861.14
2239.34
2283.38
2807.60
1045.63
1012.65
882.61
1252.70
1315.73
1274.72
1400.77
1422.82
1475.87
1179.69
1126.63
1031.60
982.49
1066.56
910.51
948.47
1331.68
1428.74
1612.92
1716.98
1644.88
1791.87
1795.97
1839.11
1941.11
1933.10
1969.20
1994.15
2083.19
2194.80
2305.34
2255.28
2384.17
2367.46
2408.29
2564.43
2536.40
2650.49
2586.43
2678.52
2825.65
2914.79

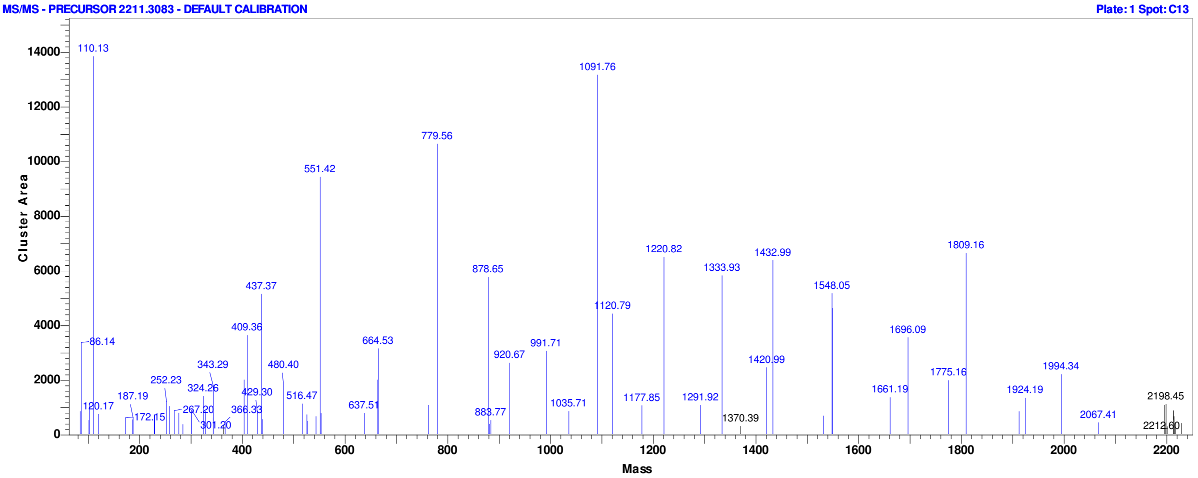
MS/MS - PRECURSOR 2211.3083 - DEFAULT CALIBRATION
Plate: 1 Spot: C13
Cluster Area
Mass
110.13
86.14
120.17
187.19
172.15
252.23
267.20
324.26
343.29
301.20
366.33
409.36
429.30
437.37
480.40
516.47
551.42
637.51
664.53
779.56
878.65
883.77
920.67
991.71
1035.71
1091.76
1120.79
1177.85
1220.82
1291.92
1333.93
1370.39
1420.99
1432.99
1548.05
1661.19
1696.09
1775.16
1809.16
1924.19
1994.34
2067.41
2198.45
2212.60

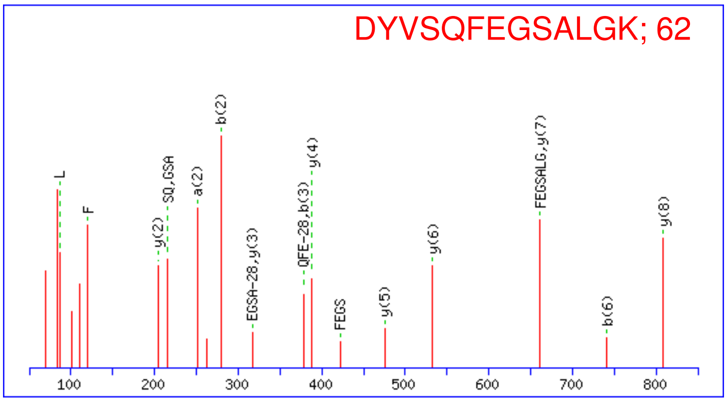
DYVSQFEGSALGK; 62
L
F
y(2)
SQ,GSA
a(2)
b(2)
EGSA-28,y(3)
QFE-28,b(3)
y(4)
FEGS
y(5)
y(6)
FEGSALG,y(7)
b(6)
y(8)

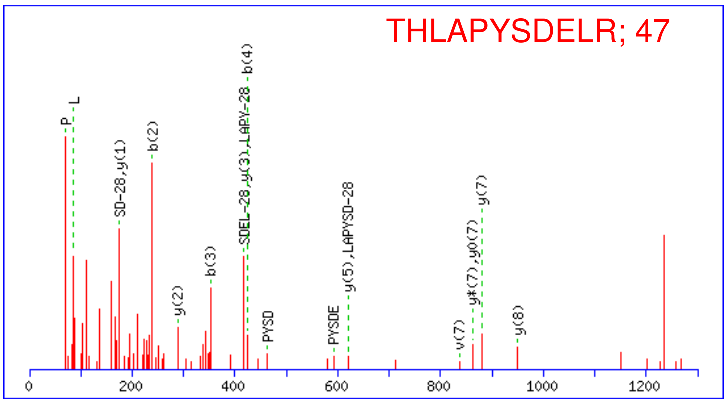
THLAPYSDELR; 47
P
L
SD-28,y(1)
b(2)
y(2)
b(3)
SDEL-28,y(3),LAPY-28,b(4)
PYSD
PYSDE
y(5),LAPYSD-28
y(7)
y*(7),y0(7)
y(7)
y(8)

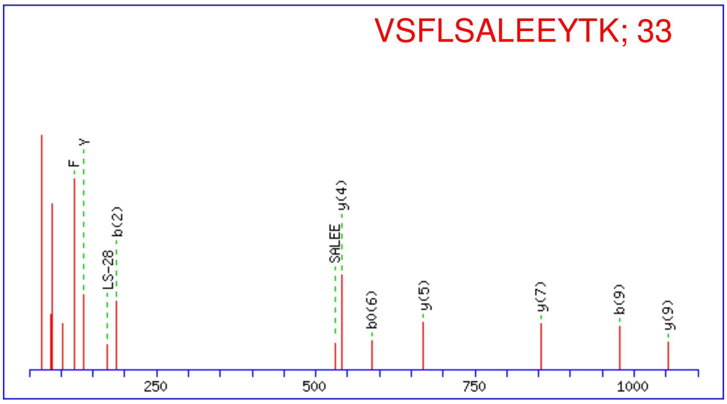
VSFLSALEEYTK; 33
F
Y
LS-28
b(2)
SALEE
y(4)
b0(6)
y(5)
y(7)
b(9)
y(9)

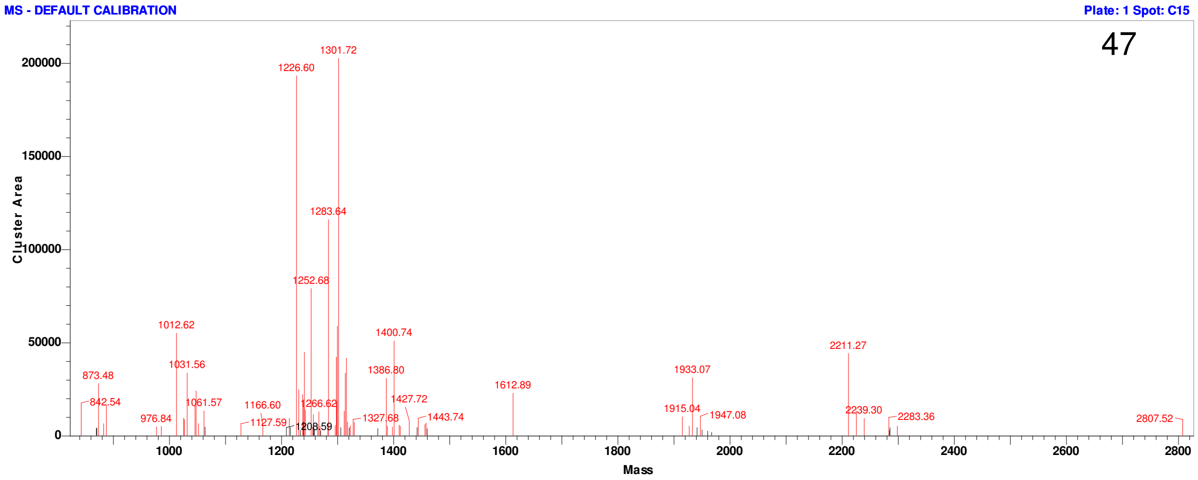
MS - DEFAULT CALIBRATION
Plate: 1 Spot: C15
47
Cluster Area
Mass
1301.72
1226.60
1283.64
1252.68
1012.62
1400.74
1031.56
1386.80
873.48
842.54
1061.57
1427.72
1166.60
1266.62
976.84
1127.59
1208.59
1327.68
1443.74
1612.89
1933.07
1915.04
1947.08
2211.27
2239.30
2283.36
2807.52

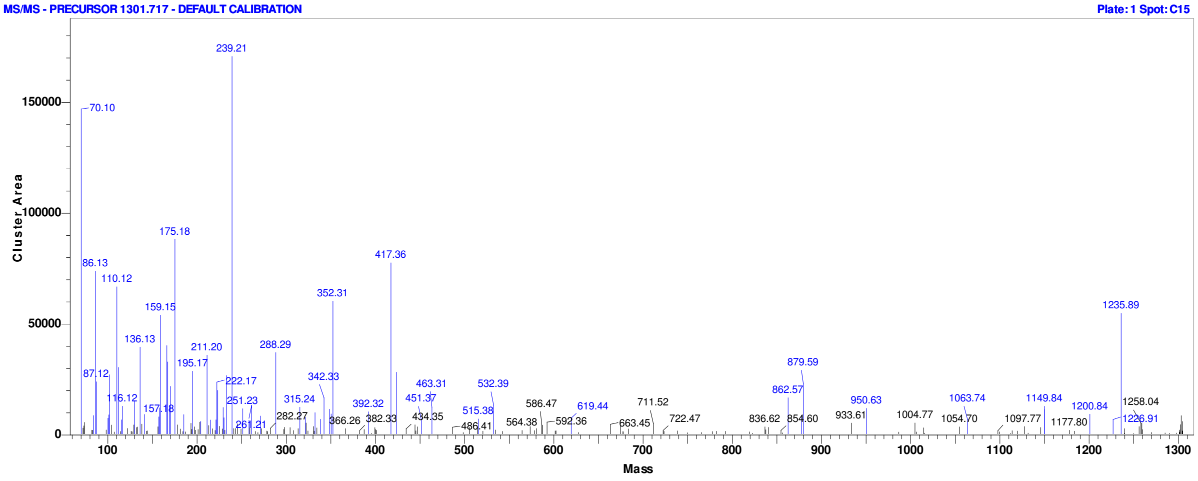
MS/MS - PRECURSOR 1301.717 - DEFAULT CALIBRATION
Plate: 1 Spot: C15
Cluster Area
Mass
239.21
70.10
175.18
86.13
110.12
417.36
352.31
159.15
136.13
211.20
195.17
288.29
87.12
222.17
342.33
116.12
251.23
315.24
157.18
261.21
282.27
392.32
366.26
382.33
463.31
451.37
434.35
532.39
515.38
486.41
586.47
564.38
592.36
619.44
711.52
663.45
722.47
879.59
862.57
836.62
854.60
950.63
933.61
1004.77
1063.74
1054.70
1097.77
1149.84
1177.80
1200.84
1235.89
1258.04
1226.91

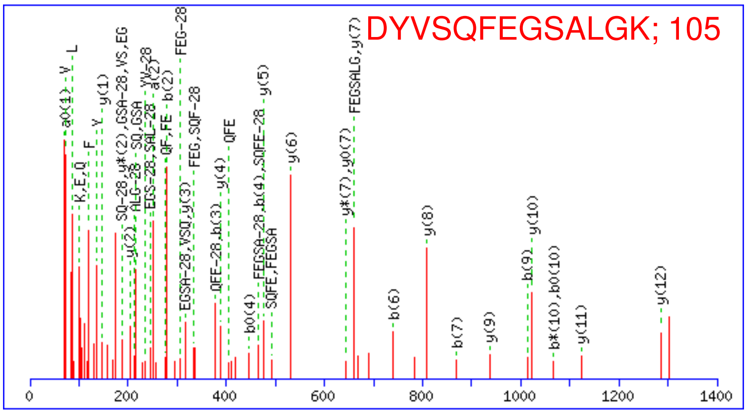
DYVSQFEGSALGK; 105
y(6)
y(8)
b(6)
b(7)
y(9)
b(9)
y(10)
y(11)
y(12)

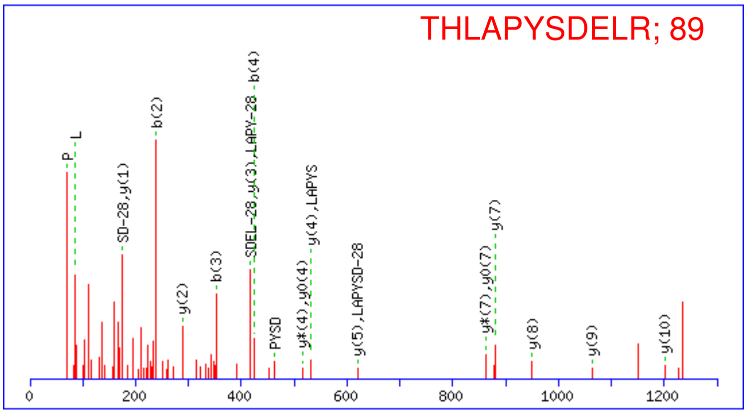
THLAPYSDELR; 89
P
L
b(2)
SD-28,y(1)
y(2)
b(3)
PYSD
y(8)
y(9)
y(10)

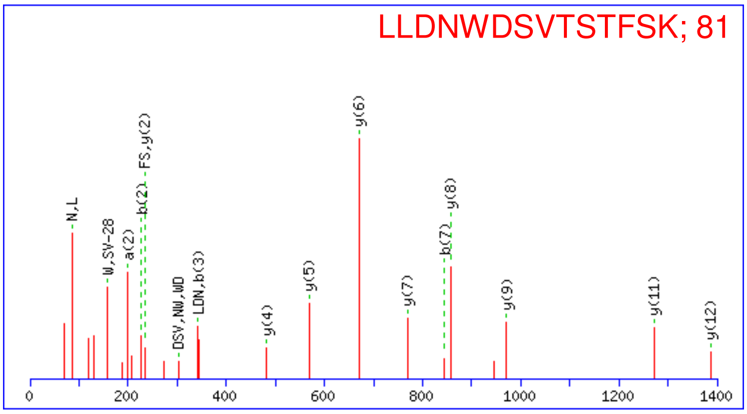
LLDNWDSVTSTFSK; 81
N,L
a(2)
b(2)
FS,y(2)
y(4)
y(5)
y(6)
y(7)
b(7)
y(8)
y(9)
y(11)
y(12)

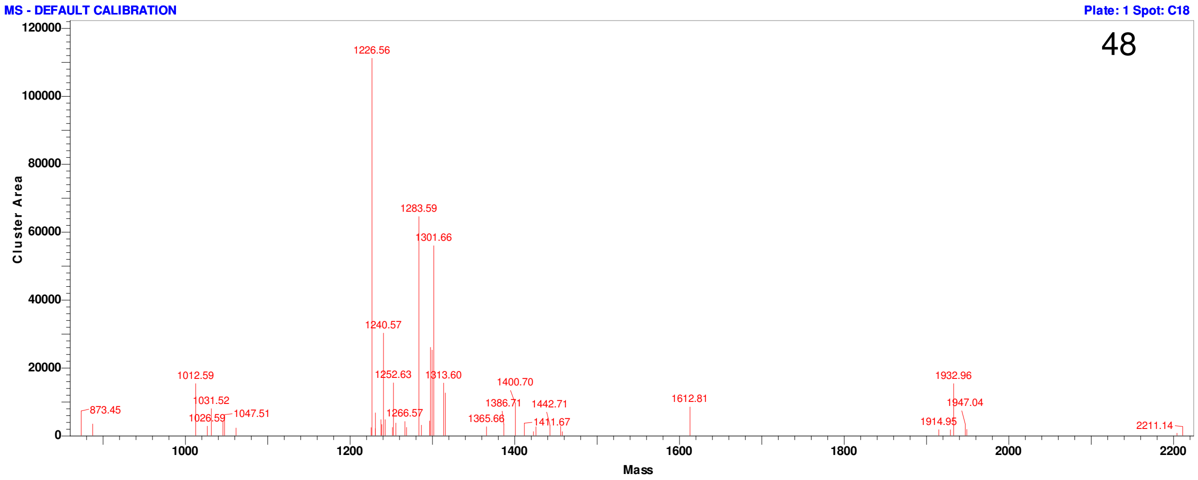

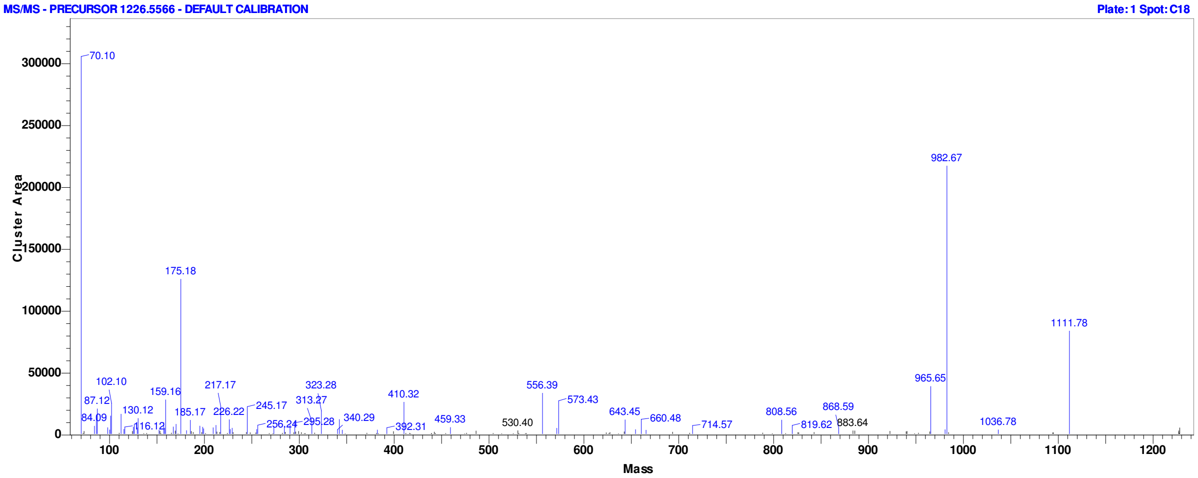

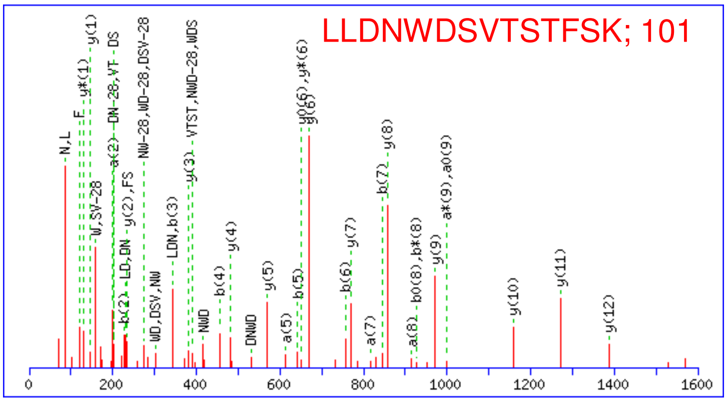

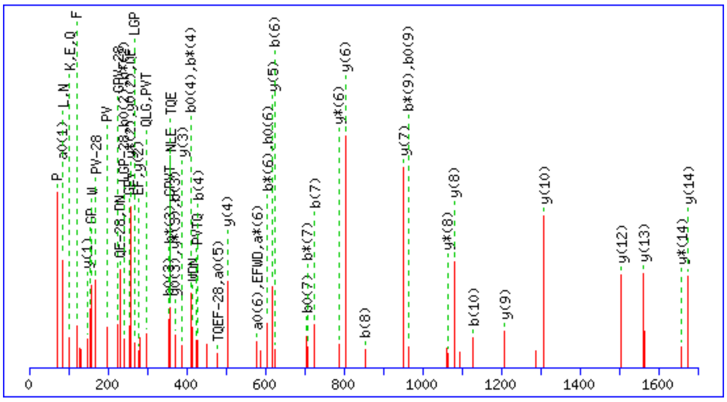

EQLGPVTQEFWDNLEK; 105

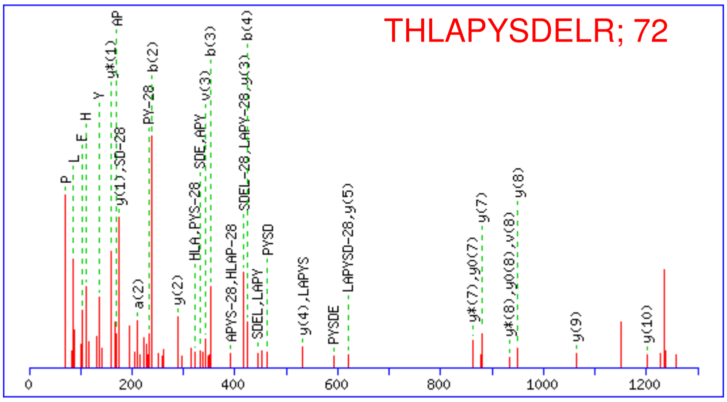

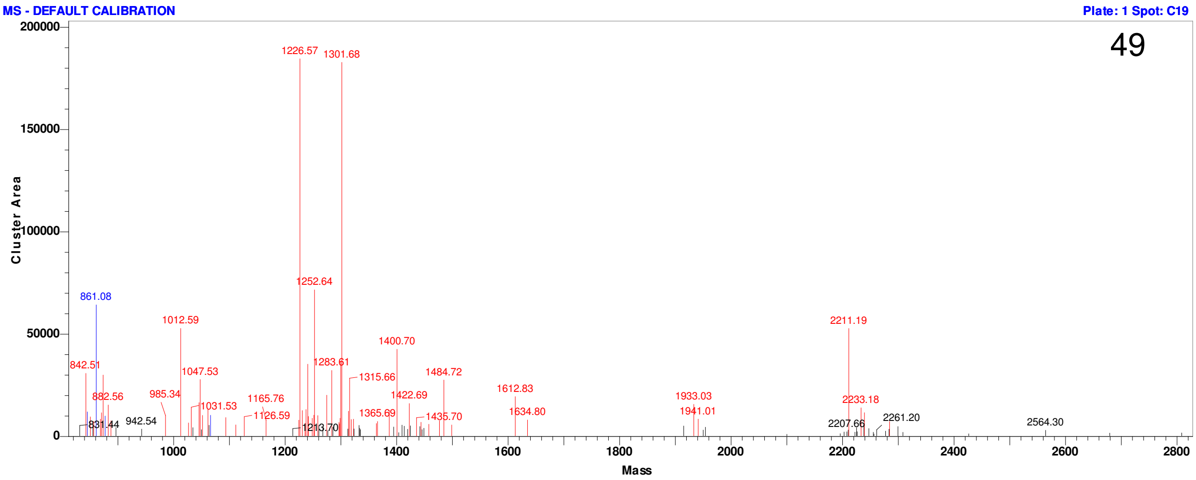
MS - DEFAULT CALIBRATION
Plate: 1 Spot: C19
49
Cluster Area
Mass
1226.57
1301.68
1252.64
861.08
1012.59
2211.19
1400.70
842.51
1283.61
1047.53
1315.66
1484.72
882.56
985.34
1031.53
1165.76
1126.59
1612.83
1634.80
1365.69
1422.69
1435.70
1933.03
1941.01
2233.18
2261.20
2207.66
2564.30
831.44
942.54
1213.70

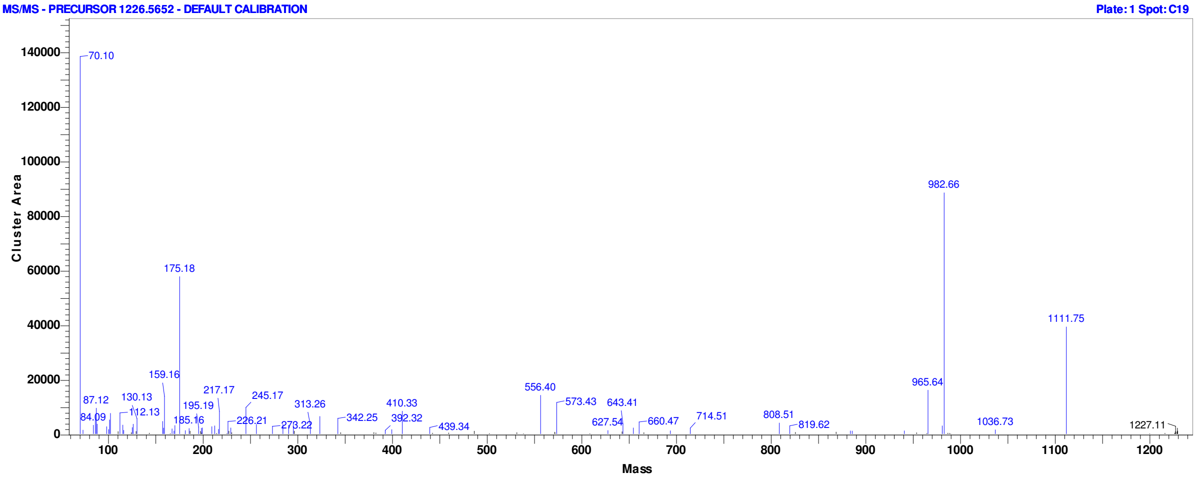
MS/MS - PRECURSOR 1226.5652 - DEFAULT CALIBRATION
Plate: 1 Spot: C19
Cluster Area
Mass
70.10
982.66
175.18
1111.75
159.16
965.64
556.40
87.12
84.09
130.13
112.13
217.17
195.19
185.16
245.17
226.21
313.26
273.22
342.25
410.33
392.32
439.34
573.43
643.41
627.54
660.47
714.51
808.51
819.62
1036.73
1227.11

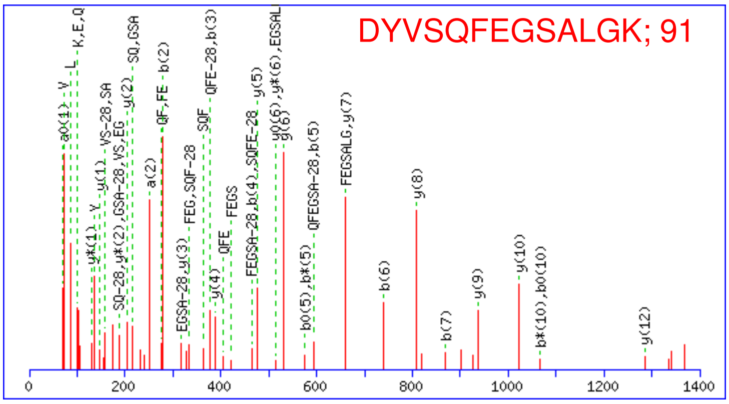
DYVSQFEGSALGK; 91

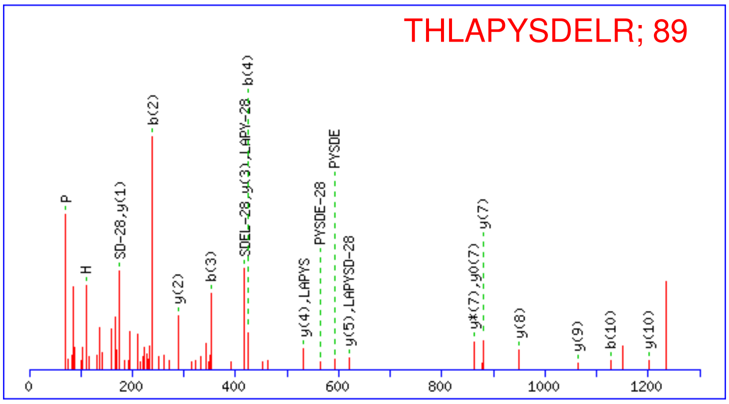
THLAPYSDELR; 89

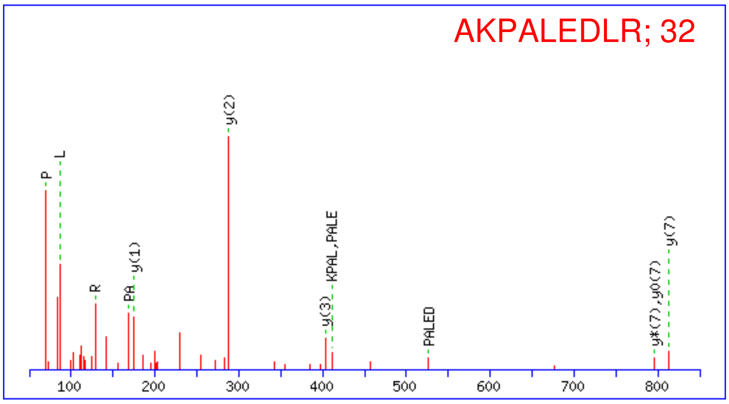
AKPALEDLR; 32

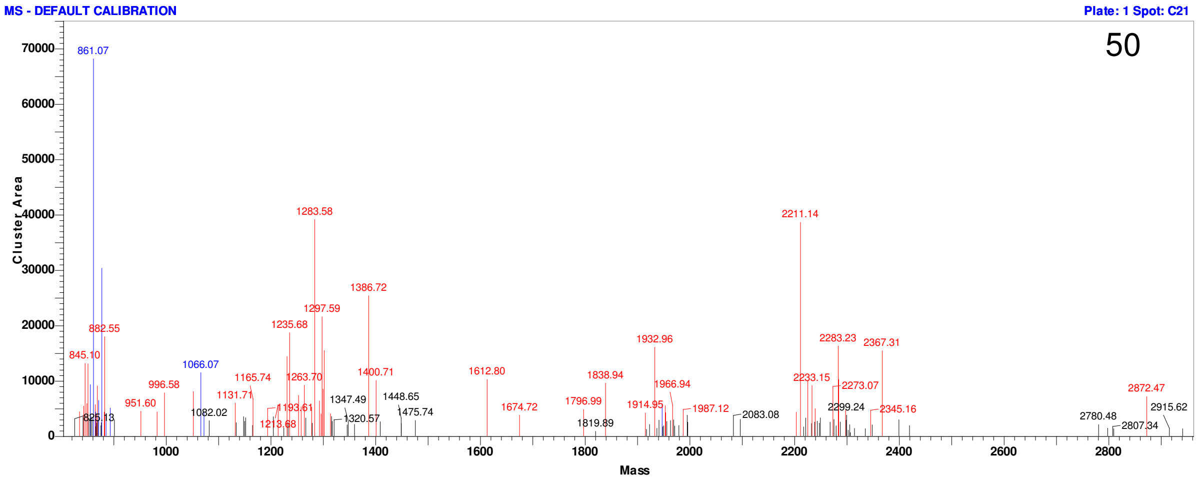
MS - DEFAULT CALIBRATION
Plate: 1 Spot: C21
50
Cluster Area
Mass

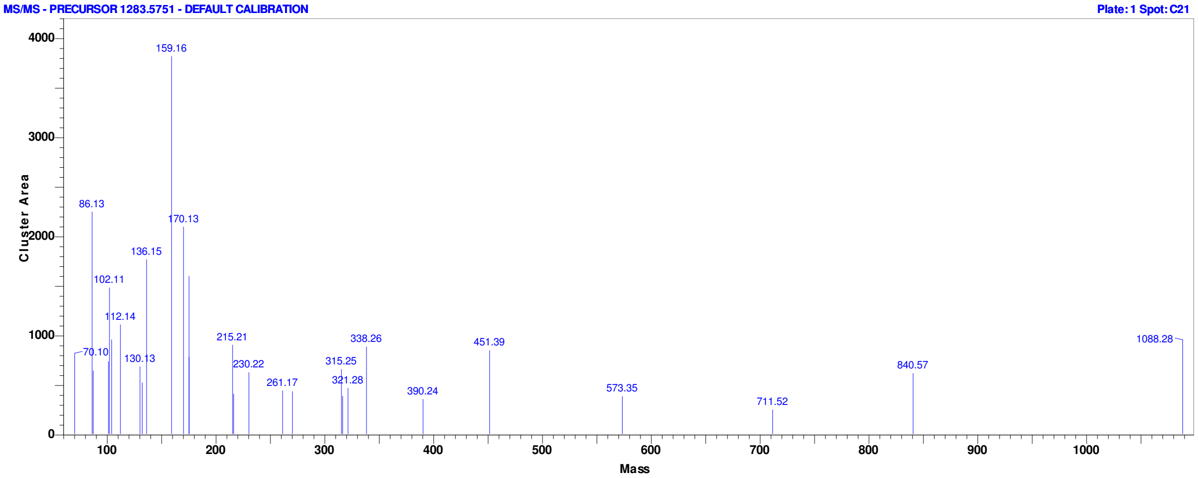
MS/MS - PRECURSOR 1283.5751 - DEFAULT CALIBRATION
Plate: 1 Spot: C21
Cluster Area
Mass

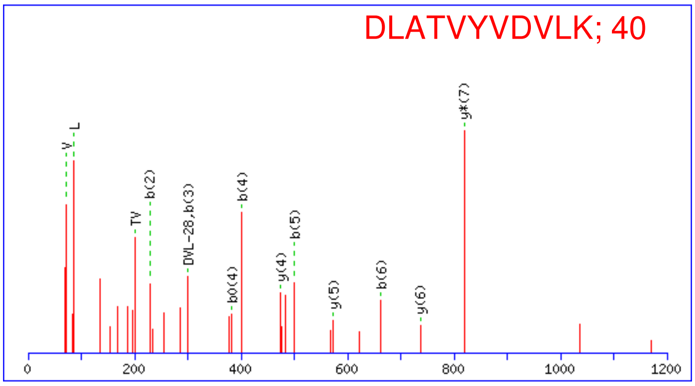
DLATVYYVDVLK; 40

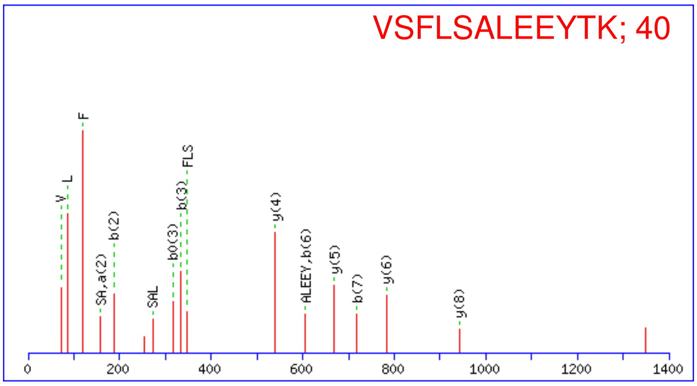
VSFLSALEEYTK; 40

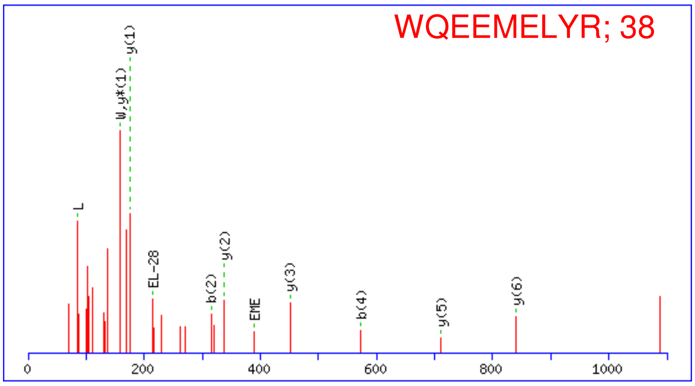
WQEEMELYR; 38

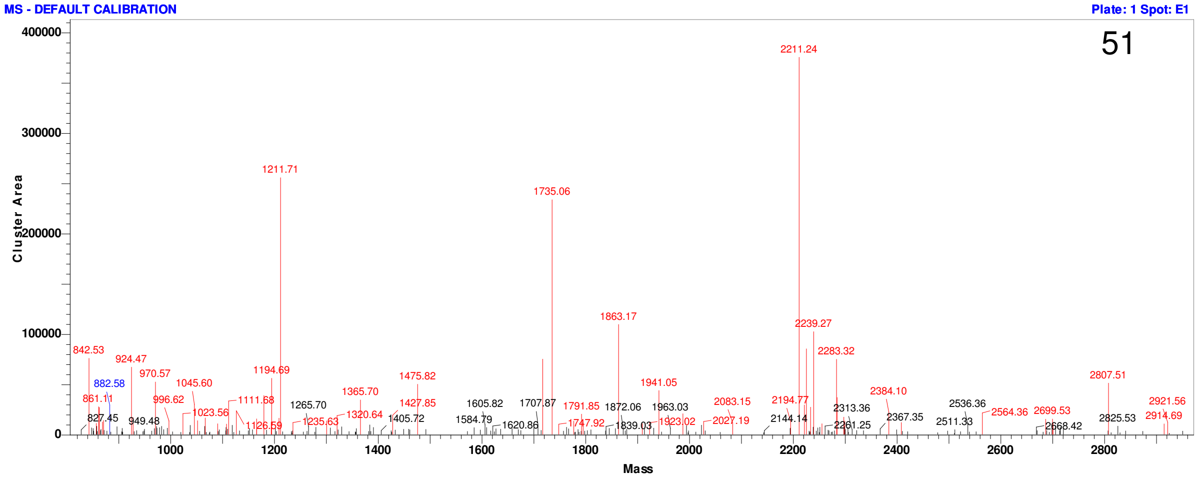
MS - DEFAULT CALIBRATION
Plate: 1 Spot: E1
51
Cluster Area
Mass
2211.24
1211.71
1735.06
1863.17
2239.27
2283.32
842.53
924.47
970.57
882.58
1045.60
1194.69
861.11
996.62
1111.68
1023.56
1265.70
1365.70
1475.82
1427.85
1320.64
1405.72
1126.59
1235.63
827.45
949.48
1605.82
1707.87
1584.79
1620.86
1791.85
1747.92
1872.06
1839.03
1941.05
1963.03
1923.02
2083.15
2027.19
2194.77
2144.14
2313.36
2261.25
2384.10
2367.35
2536.36
2511.33
2564.36
2699.53
2668.42
2807.51
2825.53
2921.56
2914.69

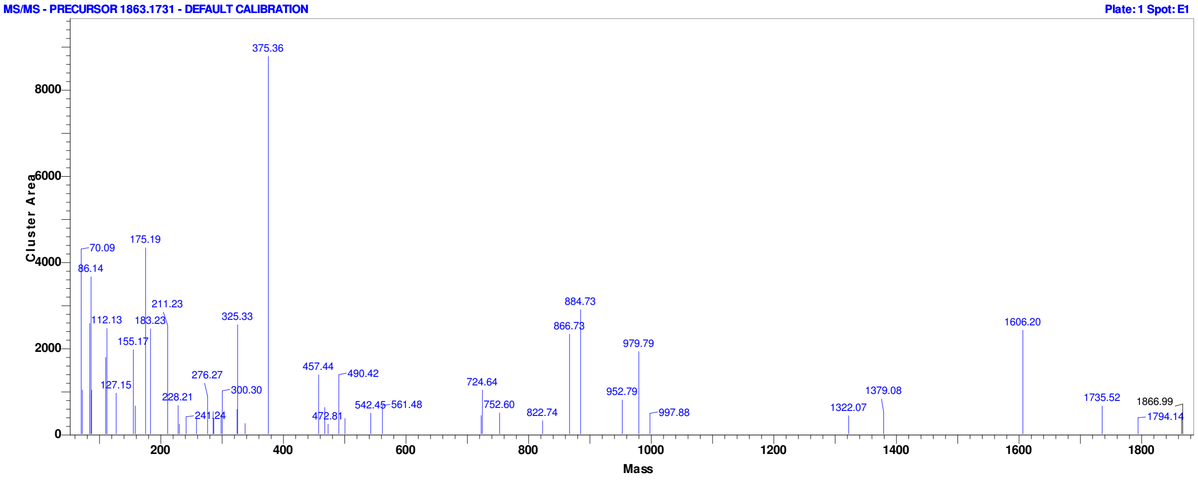
MS/MS - PRECURSOR 1863.1731 - DEFAULT CALIBRATION
Plate: 1 Spot: E1
Cluster Area
Mass
375.36
70.09
86.14
175.19
211.23
112.13
183.23
155.17
127.15
325.33
276.27
300.30
228.21
241.24
457.44
490.42
472.81
542.45
561.48
724.64
752.60
822.74
884.73
866.73
979.79
952.79
997.88
1322.07
1379.08
1606.20
1735.52
1866.99
1794.14

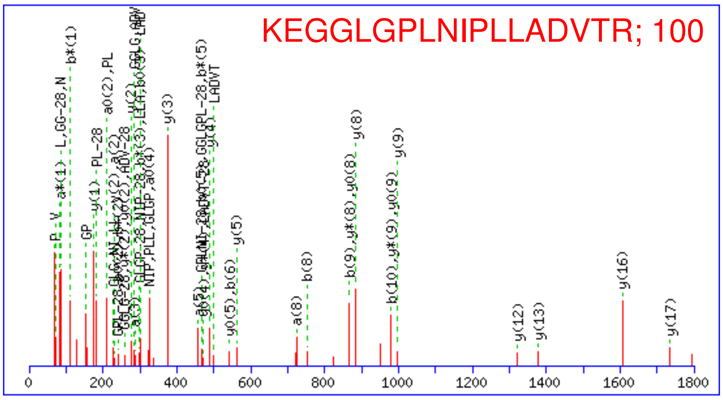
KEGGGLGPLNIPLLADVTR; 100

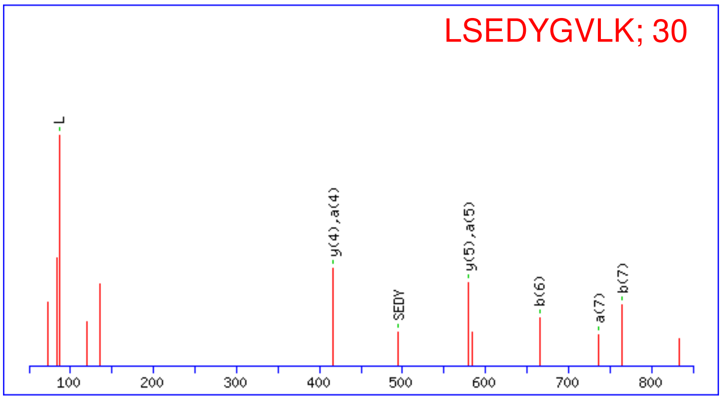
LSEDYGVLK; 30
L
y(4),a(4)
SEDY
y(5),a(5)
b(6)
a(7)
b(7)

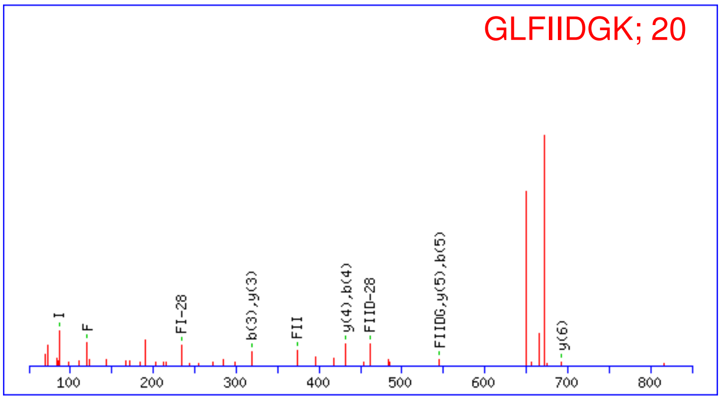
GLFIIDGK; 20
I
F
FI-28
b(3),y(3)
FII
y(4),b(4)
FIID-28
FIIDG,y(5),b(5)
y(6)

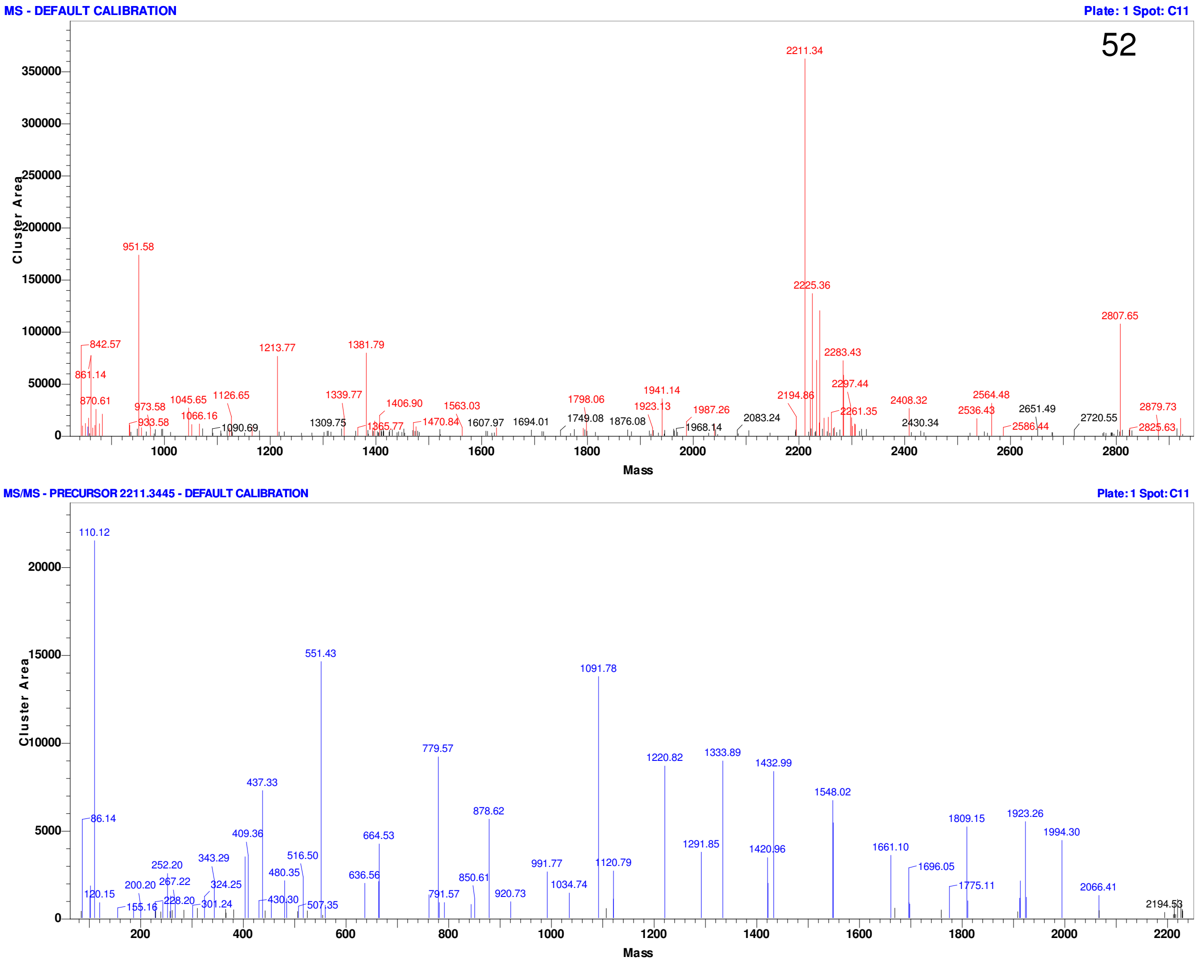
MS - DEFAULT CALIBRATION
Plate: 1 Spot: C11
52
Cluster Area
Mass
2211.34
951.58
2225.36
2807.65
MS/MS - PRECURSOR 2211.3445 - DEFAULT CALIBRATION
110.12
551.43
1091.78

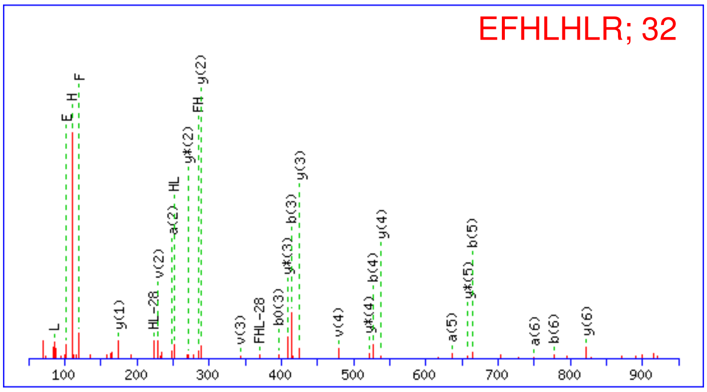
EFHLHLR; 32

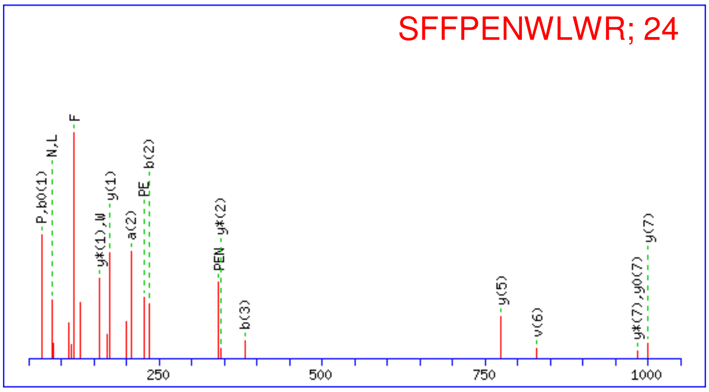
SFFPENWLWR; 24

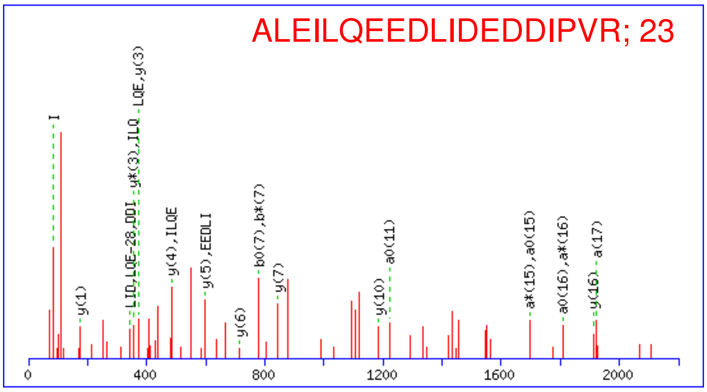
ALEILQEEDLIDEDDIPVR; 23

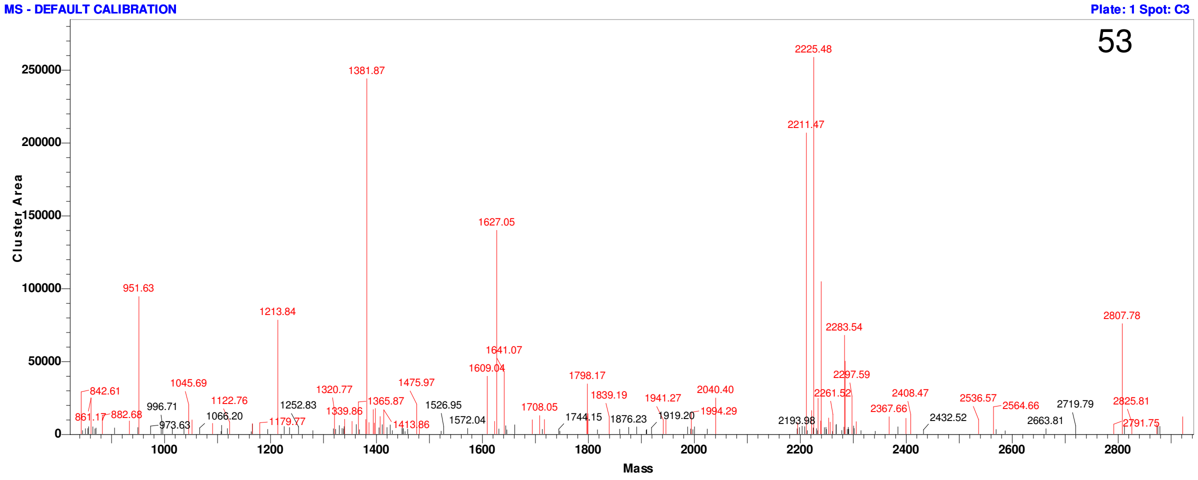
MS - DEFAULT CALIBRATION
Plate: 1 Spot: C3
53
Cluster Area
Mass
2225.48
1381.87
2211.47
1627.05
951.63
1213.84
2283.54
2807.78
1641.07
1609.04
1798.17
2297.59
1045.69
1320.77
1475.97
842.61
2040.40
2261.52
1839.19
1941.27
2408.47
2536.57
2825.81
996.71
1122.76
1252.83
1365.87
1526.95
1708.05
2564.66
2719.79
2367.66
861.17
882.68
1066.20
1339.86
1572.04
1744.15
1876.23
1919.20
1994.29
2193.98
2432.52
2663.81
973.63
1179.77
1413.86
2791.75

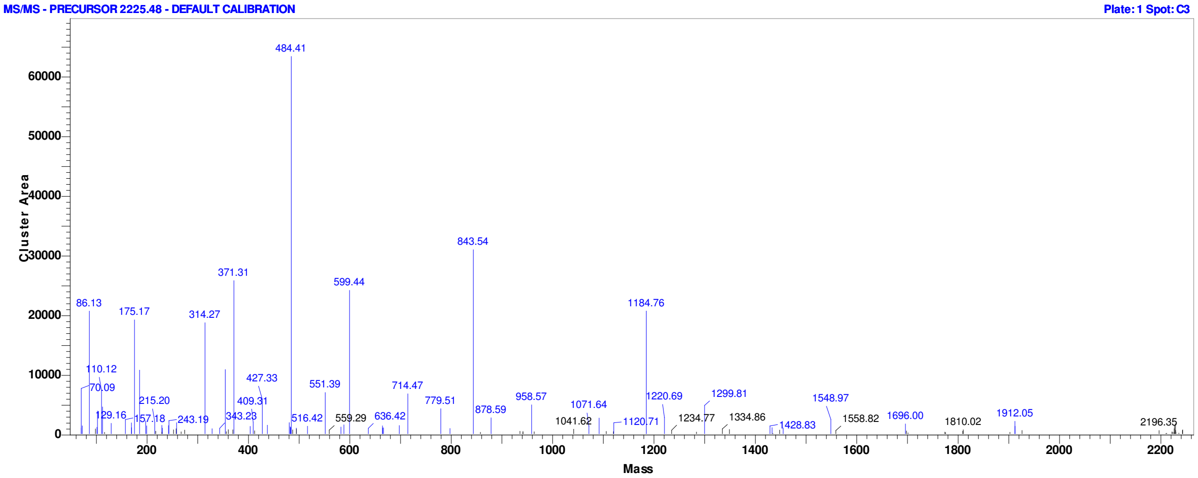
MS/MS - PRECURSOR 2225.48 - DEFAULT CALIBRATION
Plate: 1 Spot: C3
Cluster Area
Mass
484.41
843.54
599.44
371.31
86.13
175.17
314.27
1184.76
110.12
70.09
427.33
551.39
714.47
215.20
409.31
779.51
958.57
1071.64
1220.69
1299.81
1548.97
129.16
157.18
243.19
343.23
516.42
559.29
636.42
878.59
1041.62
1120.71
1234.77
1334.86
1428.83
1558.82
1696.00
1810.02
1912.05
2196.35

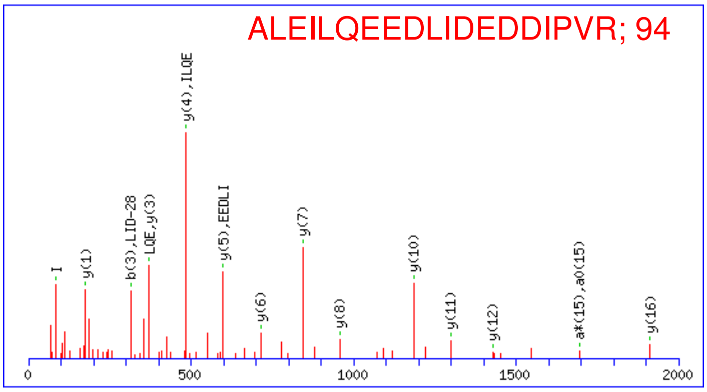
ALEILQEEDLIDEDDIPVR; 94

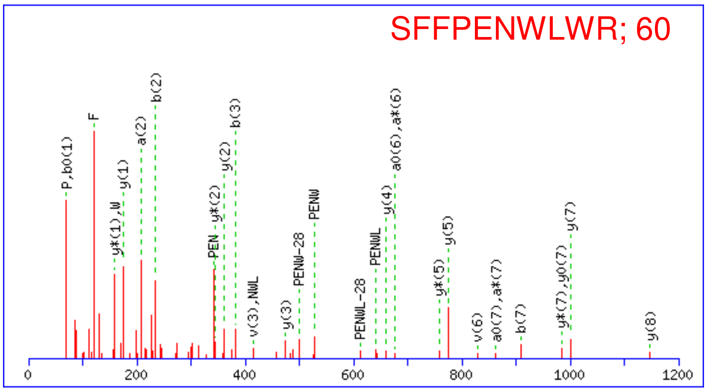
SFFPENWLWR; 60

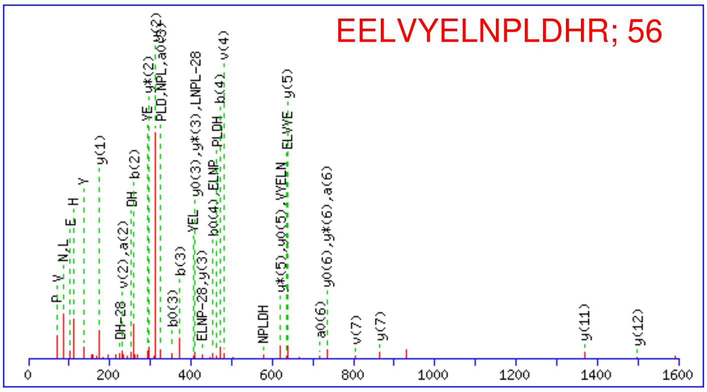
EELVYELNPLDHR; 56

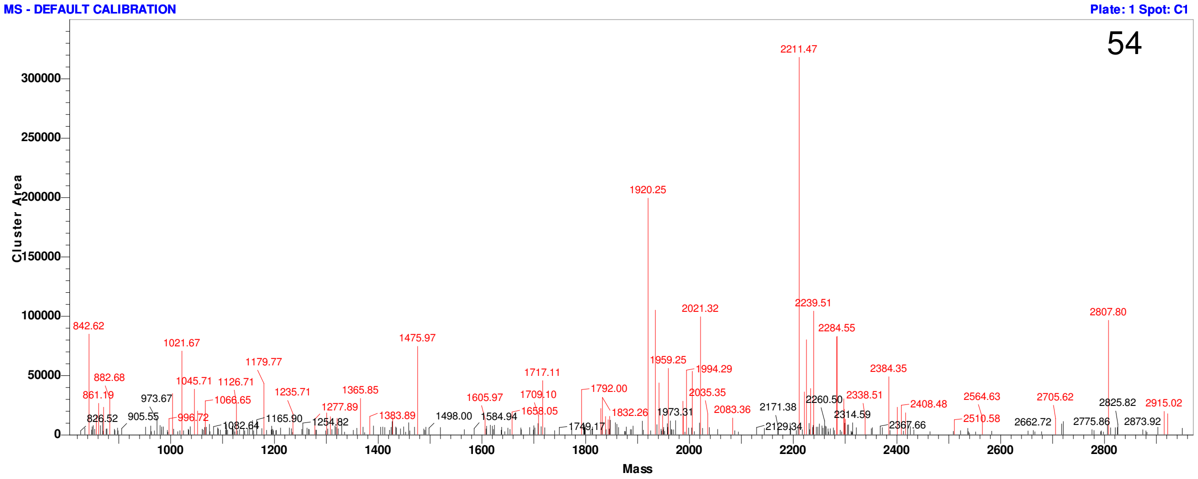
MS - DEFAULT CALIBRATION
Plate: 1 Spot: C1
54
Cluster Area
Mass
2211.47
1920.25
2807.80
2239.51
2021.32
842.62
1021.67
1475.97

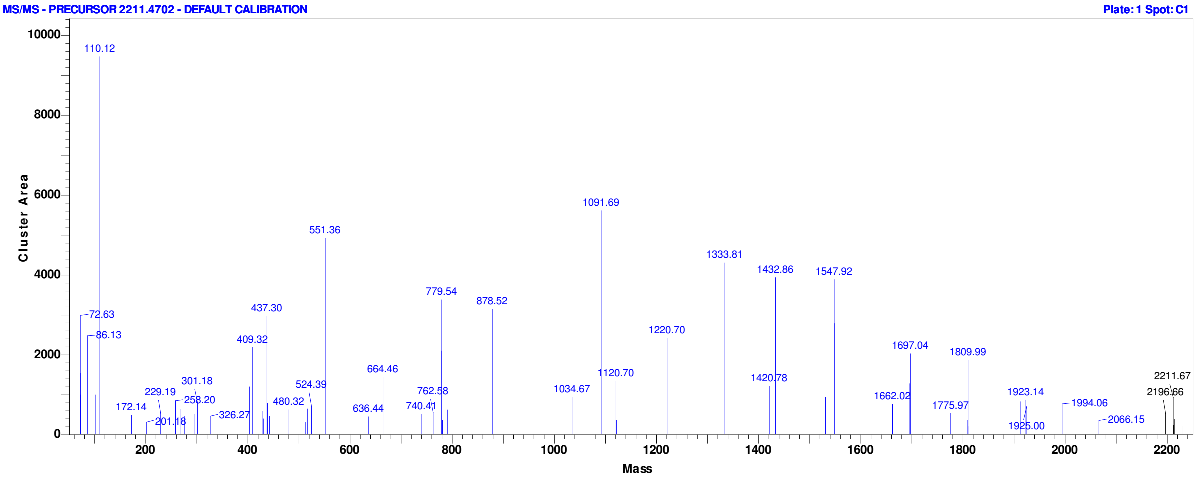
MS/MS - PRECURSOR 2211.4702 - DEFAULT CALIBRATION
Plate: 1 Spot: C1
Cluster Area
Mass
110.12
1091.69
551.36
1333.81
1432.86
1547.92
779.54
878.52
437.30
409.32
2211.67

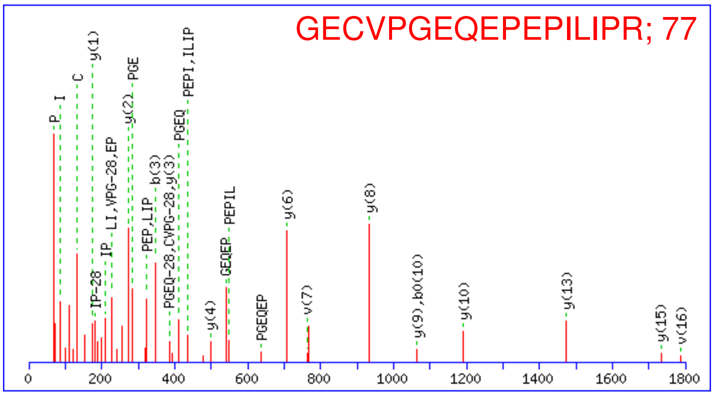
GECVPGEQEPEPILIPR; 77

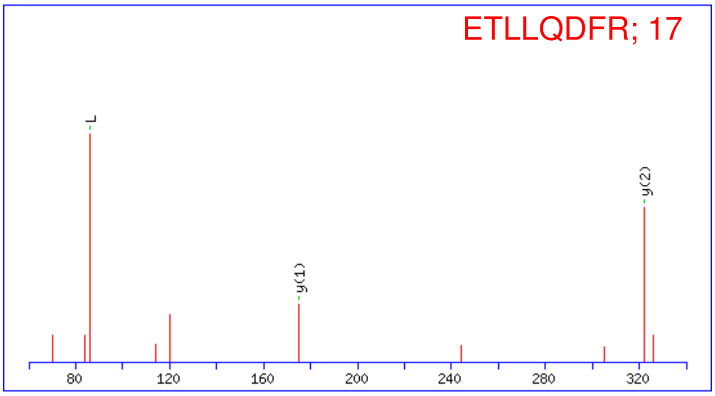
ETLLQDFR; 17

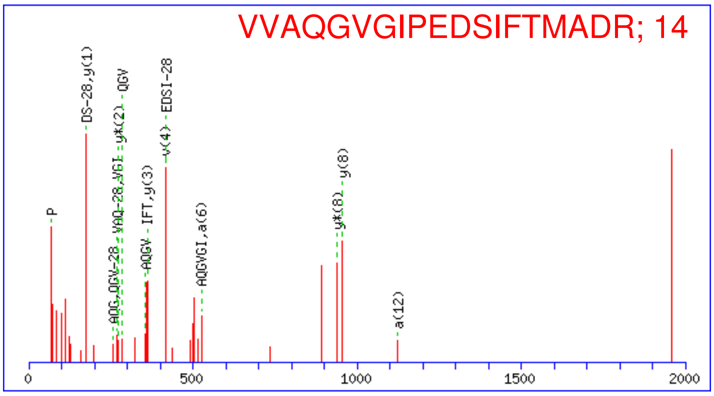
VVAQGVGIPEDSIFTMADR; 14

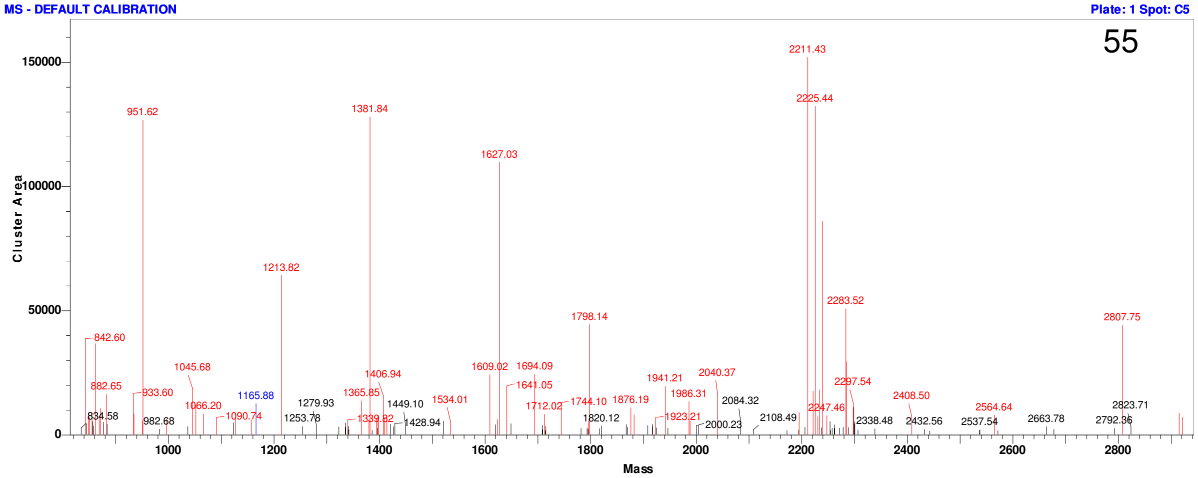
MS - DEFAULT CALIBRATION
Plate: 1 Spot: C5
55
Cluster Area
Mass
2211.43
2225.44
951.62
1381.84
1627.03
1213.82
1798.14
2283.52
2807.75
842.60
1045.68
1406.94
1609.02
1694.09
1641.05
1941.21
2040.37
882.65
933.60
1365.85
1534.01
2297.54
1165.88
1279.93
1449.10
1986.31
2084.32
1066.20
1712.02
1744.10
1876.19
2408.50
2247.46
2564.64
1090.74
1339.82
1923.21
2823.71
834.58
982.68
1253.78
1428.94
1820.12
2000.23
2108.49
2338.48
2432.56
2537.54
2663.78
2792.36

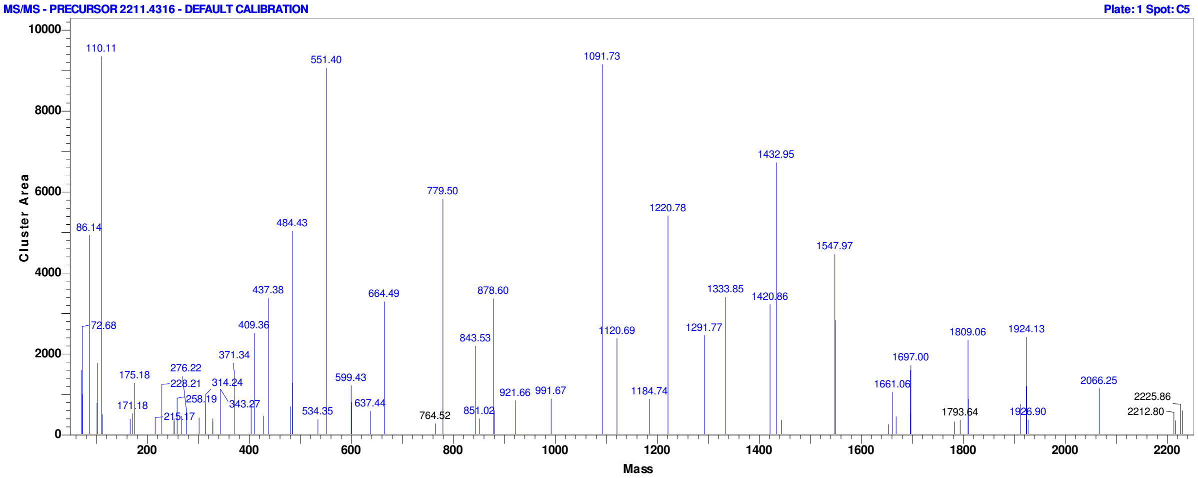
MS/MS - PRECURSOR 2211.4316 - DEFAULT CALIBRATION
Plate: 1 Spot: C5
Cluster Area
Mass
110.11
551.40
1091.73
1432.95
779.50
1220.78
86.14
484.43
1547.97
437.38
664.49
878.60
1333.85
1420.86
72.68
409.36
1120.69
1291.77
1809.06
1924.13
371.34
843.53
1697.00
175.18
276.22
228.21
314.24
599.43
1661.06
2066.25
258.19
343.27
637.44
921.66
991.67
1184.74
2225.86
171.18
215.17
534.35
764.52
851.02
1793.64
1926.90
2212.80

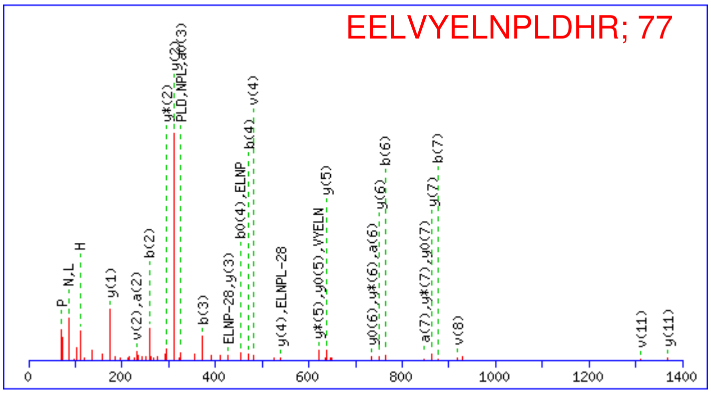
EELVYELNPLDHR; 77

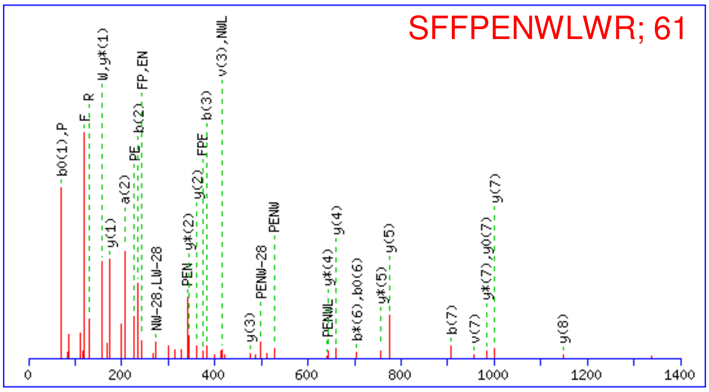
SFFPENWLWR; 61

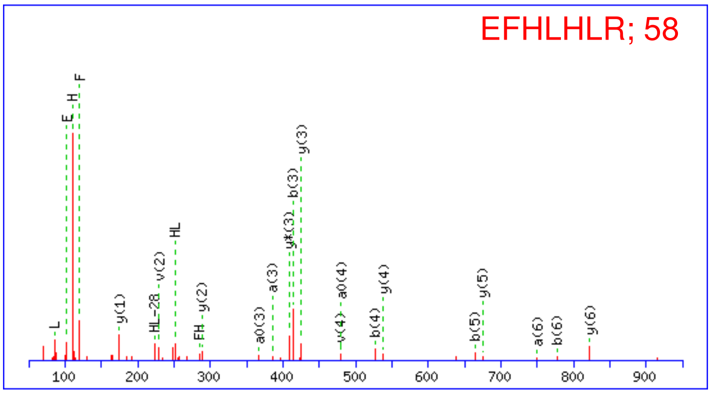
EFHLHLR; 58

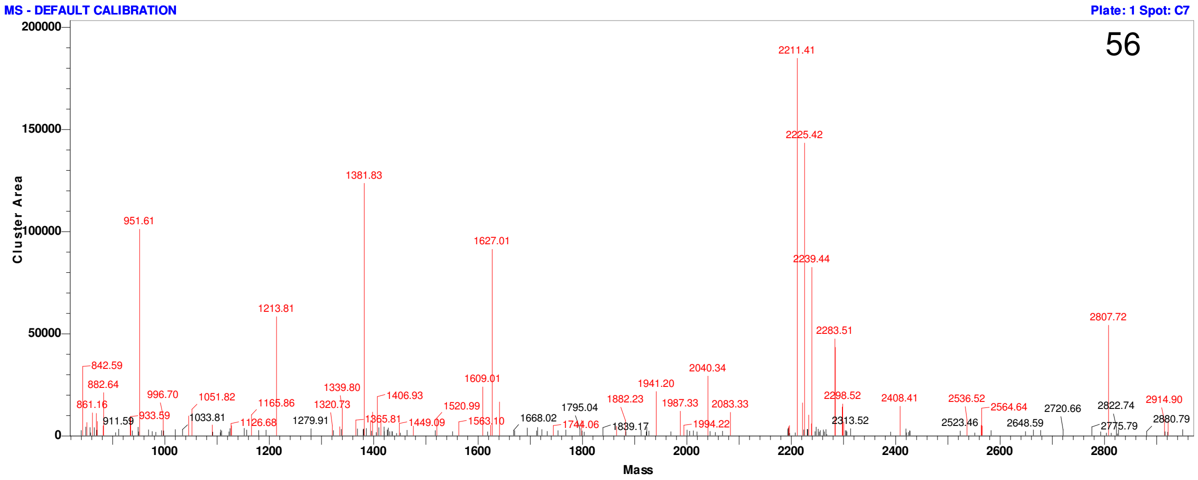
MS - DEFAULT CALIBRATION
Plate: 1 Spot: C7
56
Cluster Area
Mass
2211.41
2225.42
1381.83
951.61
1627.01
2239.44
1213.81
2807.72
2283.51

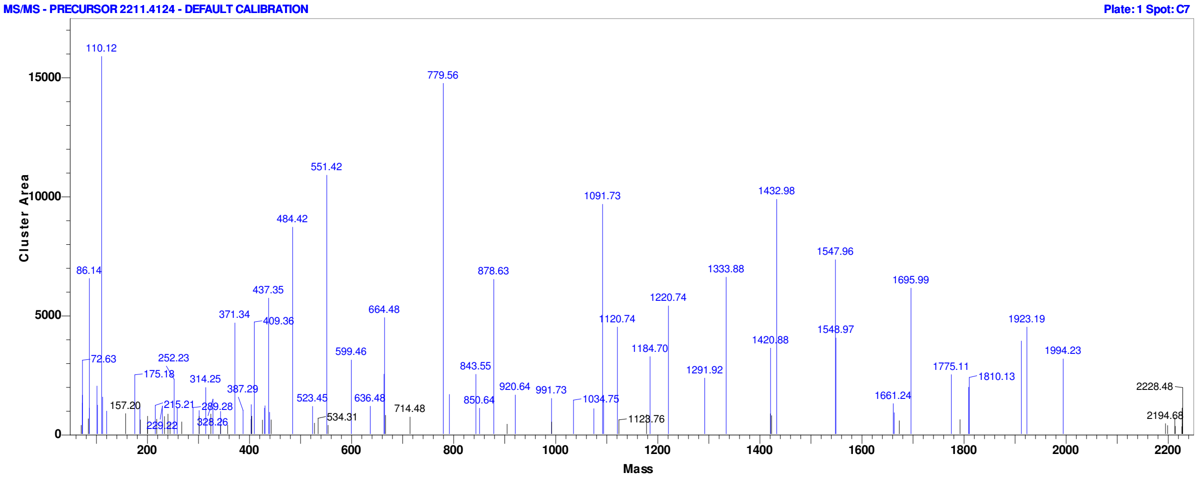
MS/MS - PRECURSOR 2211.4124 - DEFAULT CALIBRATION
Plate: 1 Spot: C7
Cluster Area
Mass
110.12
779.56
551.42
1091.73
1432.98
484.42

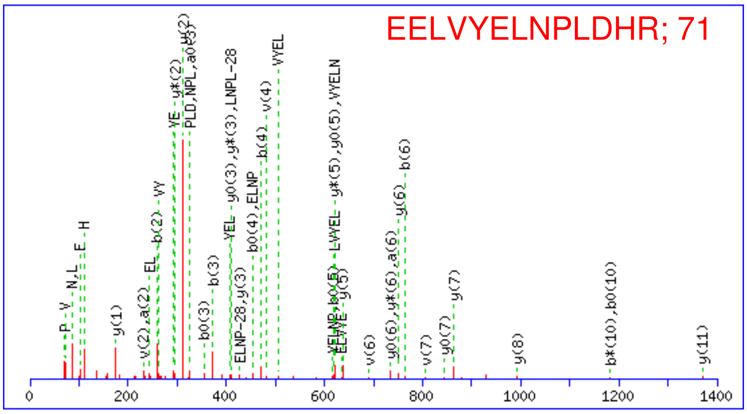
EELVYELNPLDHR; 71

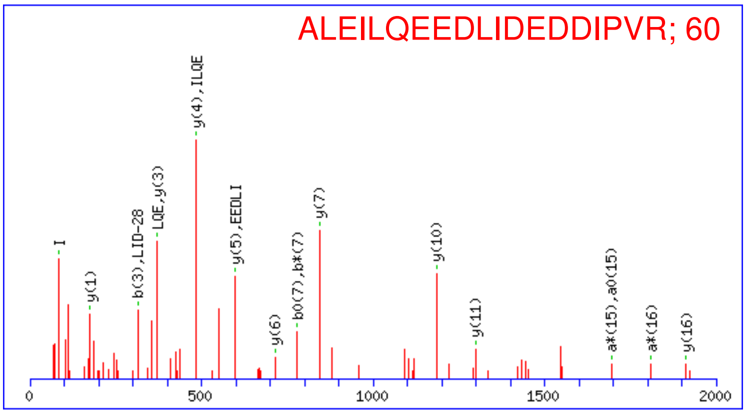
ALEILQEEDLIDEDDIPVR; 60

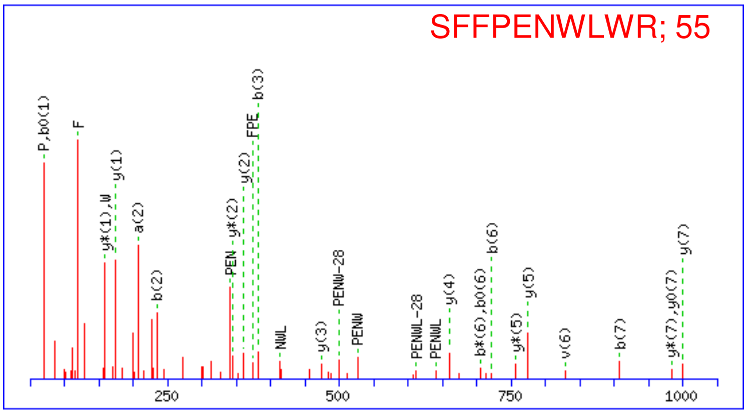
SFFPENWLWR; 55

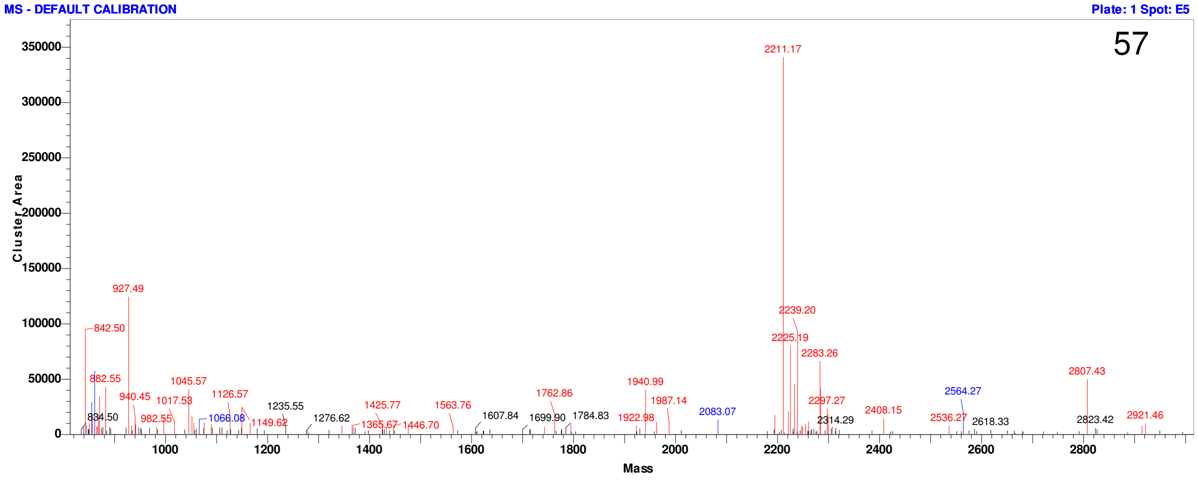
MS - DEFAULT CALIBRATION
Plate: 1 Spot: E5
57
Cluster Area
Mass
2211.17
927.49
842.50
2239.20
2225.19
2283.26
2807.43
882.55
1045.57
1940.99
2564.27
940.45
1017.53
1126.57
1762.86
1987.14
2297.27
1235.55
1425.77
1563.76
2083.07
2408.15
2921.46
834.50
982.55
1066.08
1149.62
1276.62
1365.67
1446.70
1607.84
1699.90
1784.83
1922.98
2314.29
2536.27
2618.33
2823.42

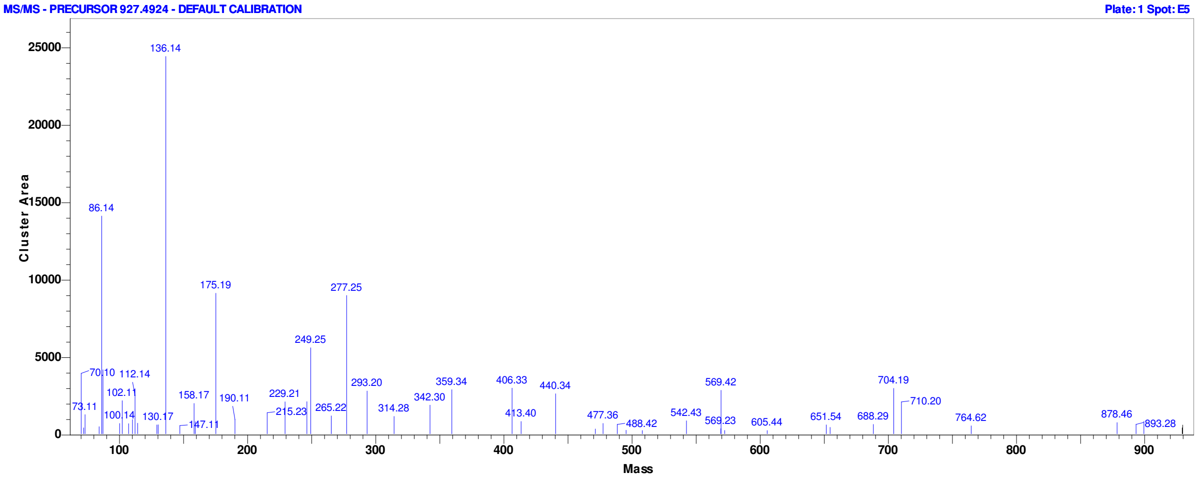
MS/MS - PRECURSOR 927.4924 - DEFAULT CALIBRATION
Plate: 1 Spot: E5
Cluster Area
Mass
136.14
86.14
175.19
277.25
249.25
70.10
112.14
102.11
73.11
100.14
130.17
158.17
147.11
190.11
229.21
215.23
265.22
293.20
314.28
342.30
359.34
406.33
413.40
440.34
477.36
488.42
542.43
569.42
569.23
605.44
651.54
688.29
704.19
710.20
764.62
878.46
893.28

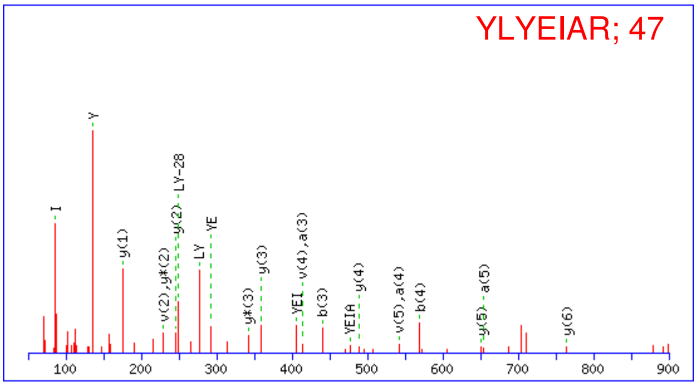
YLYEIAR; 47

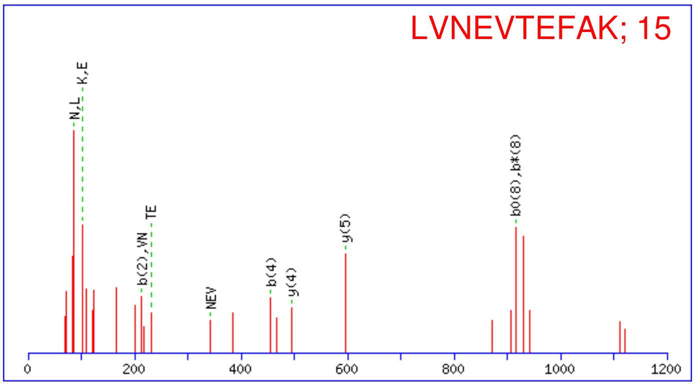
LVNEVTEFAK; 15

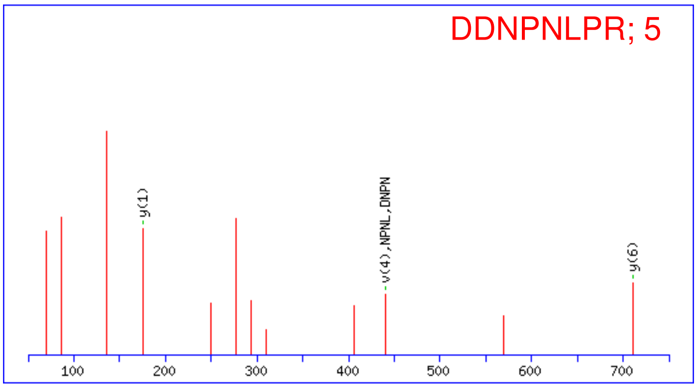
DDNPNLPR; 5

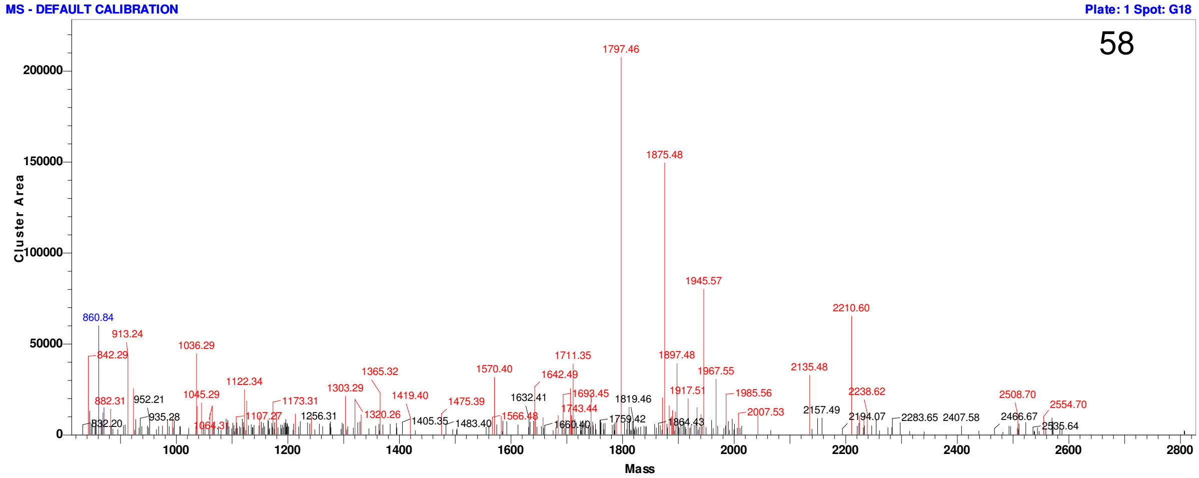

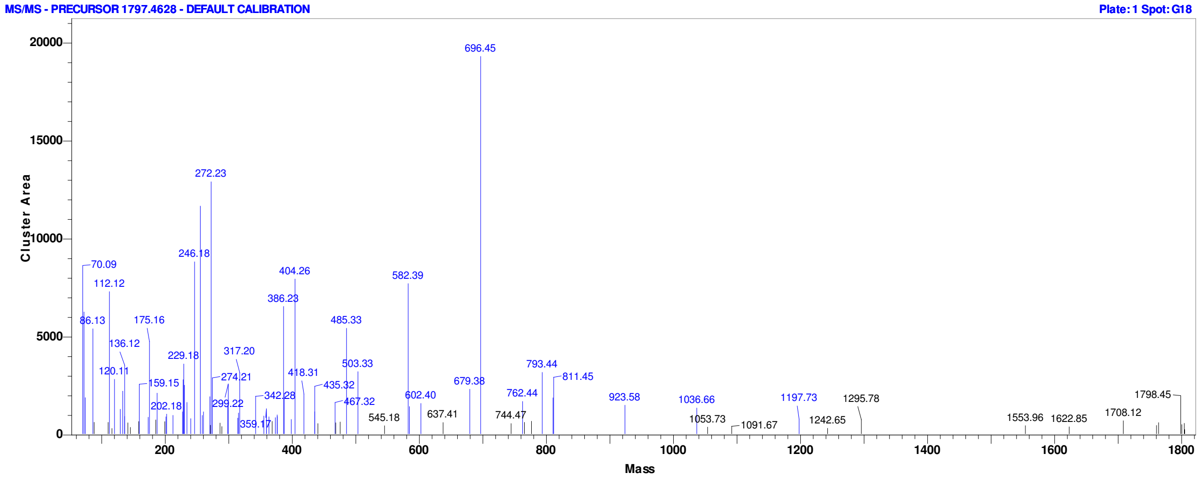

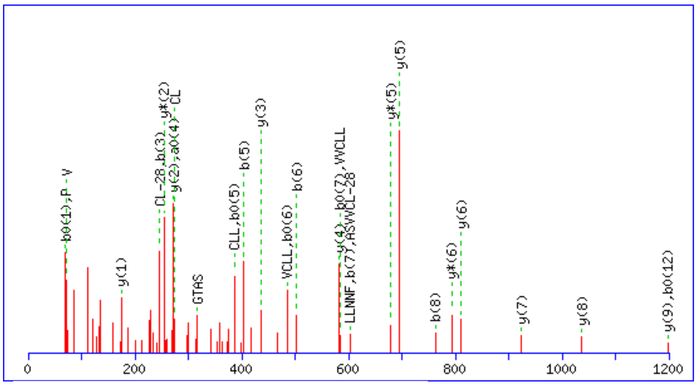

SGTASVVCLLNNFYPR; 83

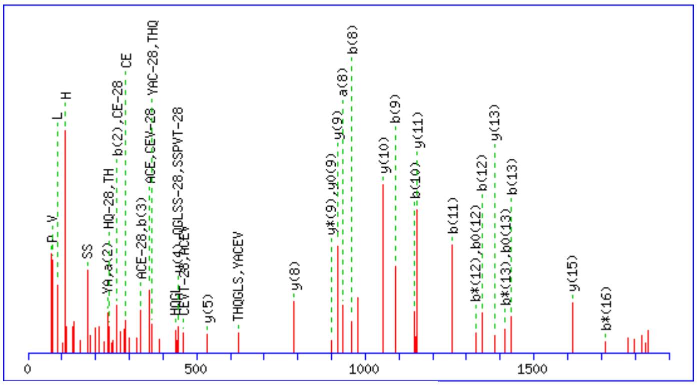

VYACEVTHQGLSSPVTK; 74

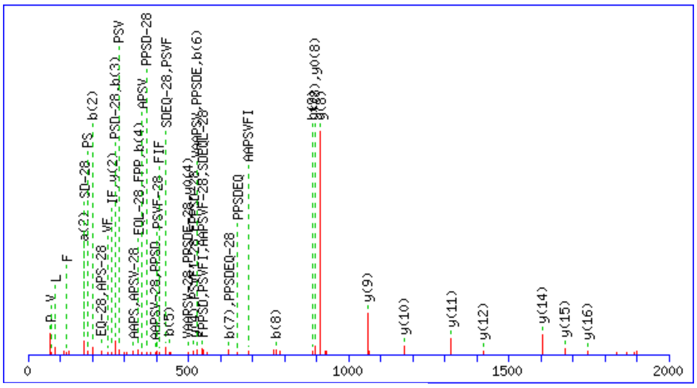

TVAAPSVFIFPPSDEQLK; 64

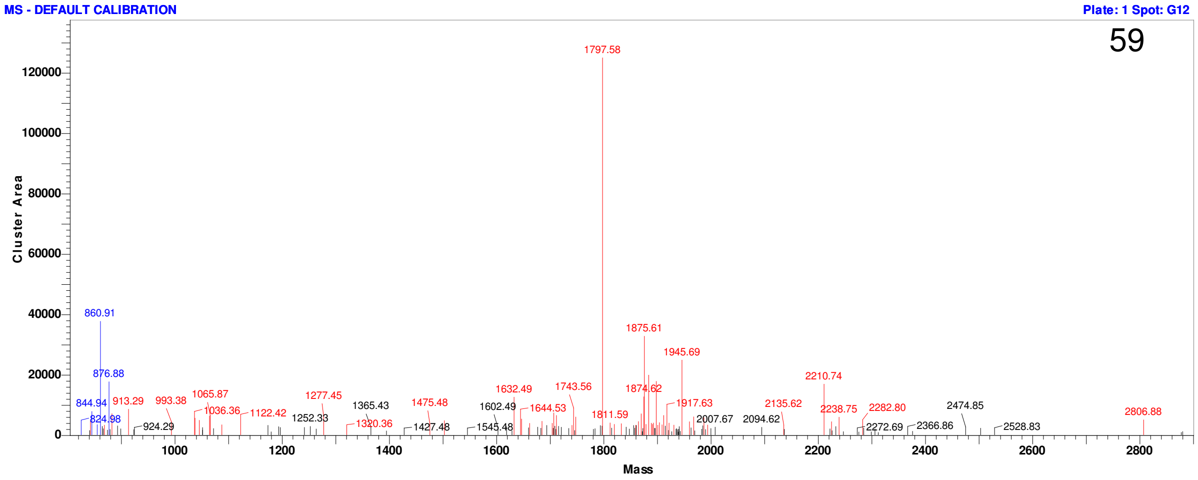

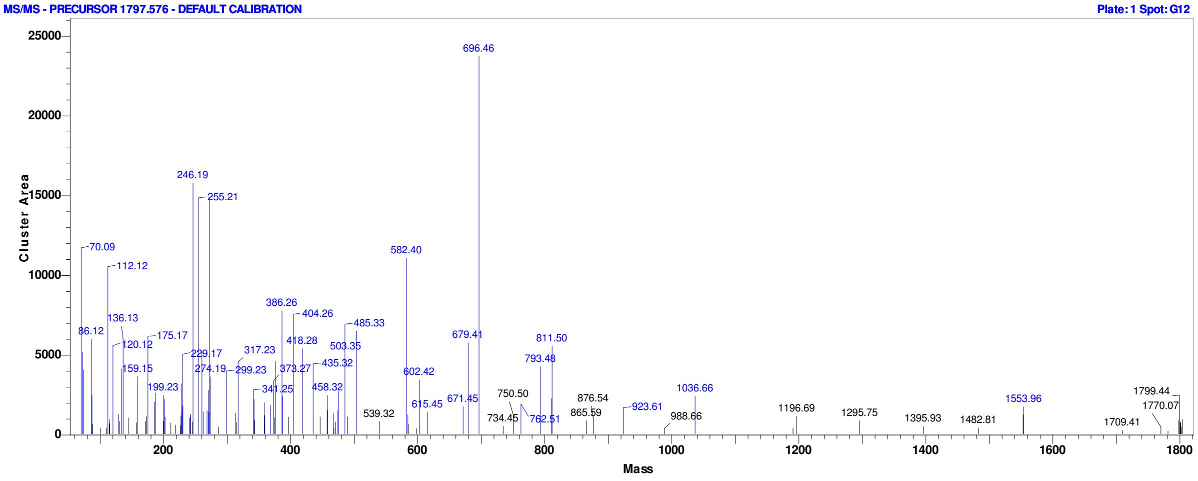

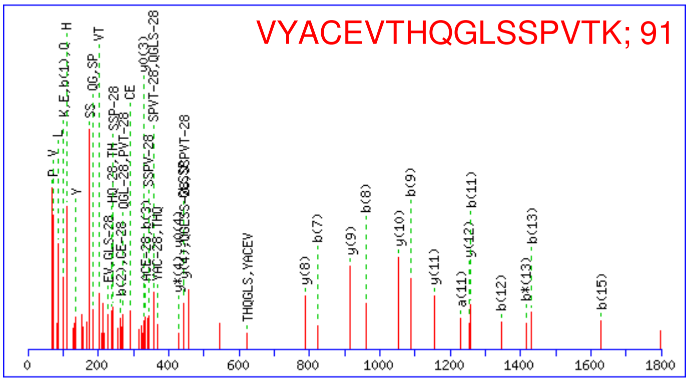

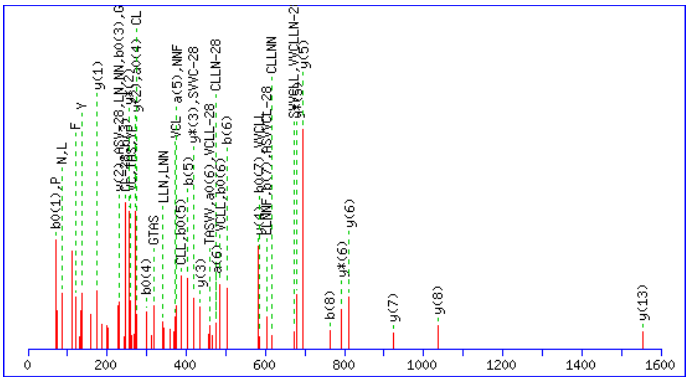

SGTASVVCLLNNFYPR; 85

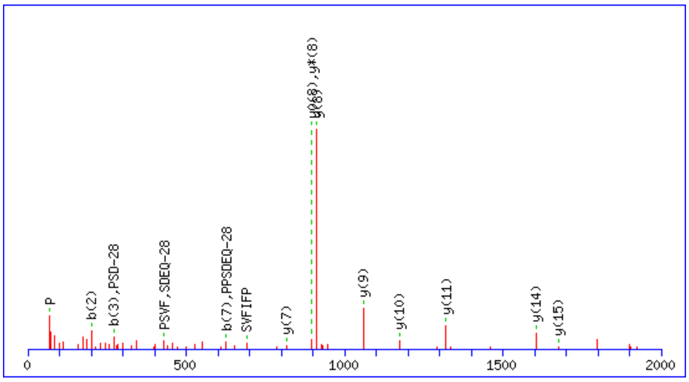

TVAAPSVFIFPPSDEQLK; 50

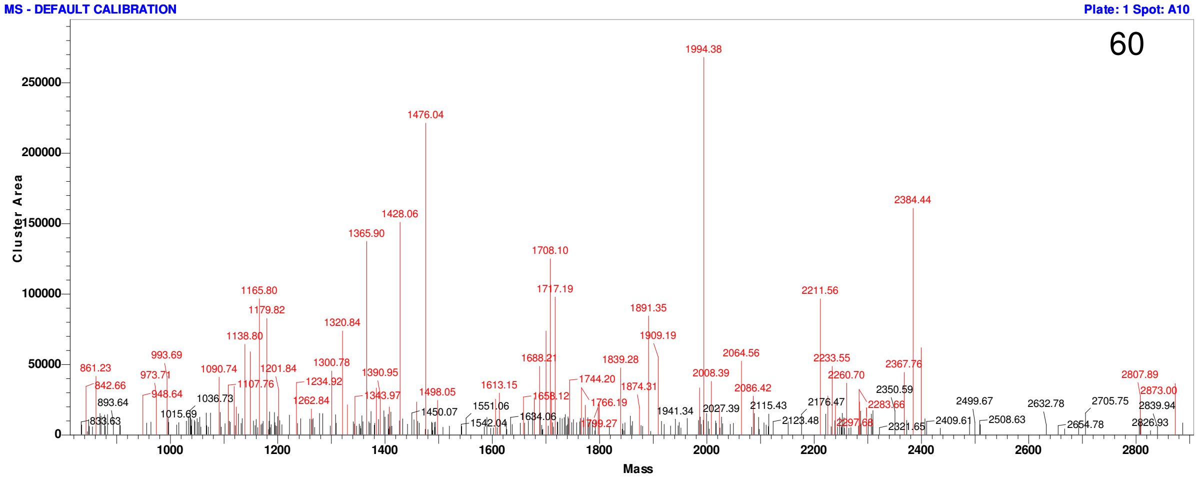

MS - DEFAULT CALIBRATION
Plate: 1 Spot: A10
60
Cluster Area
Mass

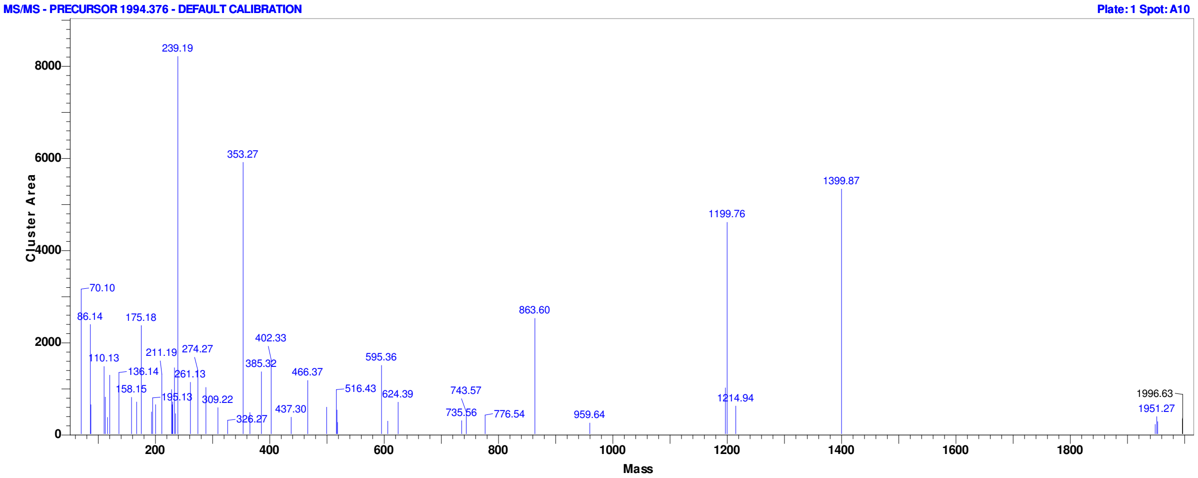

MS/MS - PRECURSOR 1994.376 - DEFAULT CALIBRATION
Plate: 1 Spot: A10
Cluster Area
Mass

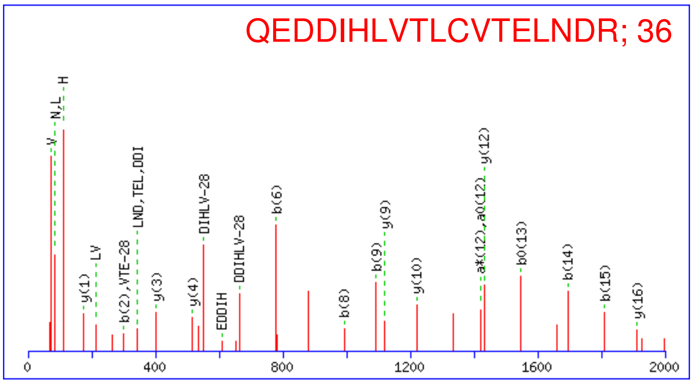

QEDDIHLVTLCVTELNDR; 36

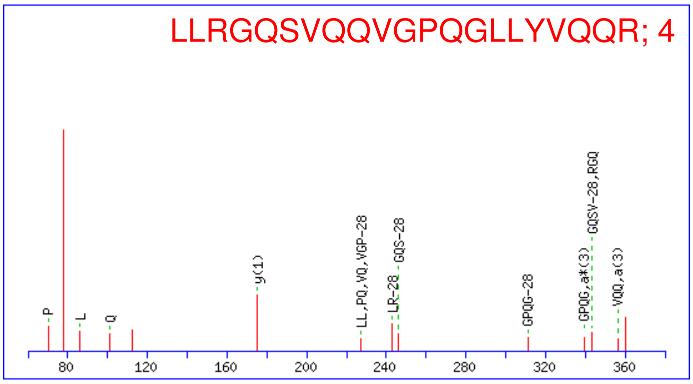

LLRGQSVQQVGPQGLLYVQQR; 4

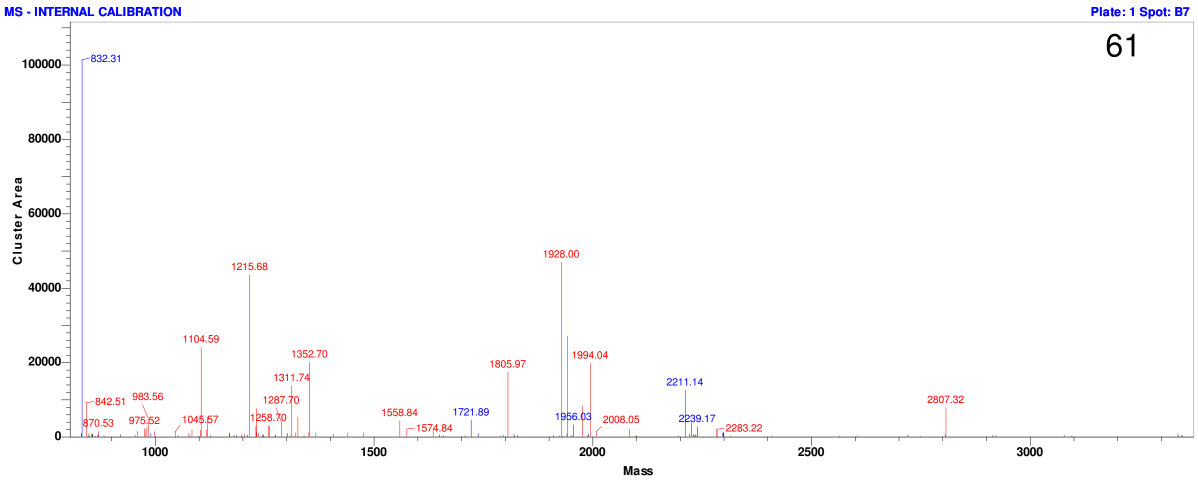
MS - INTERNAL CALIBRATION
Plate: 1 Spot: B7
61
Cluster Area
Mass
832.31
842.51
870.53
975.52
983.56
1045.57
1104.59
1215.68
1258.70
1287.70
1311.74
1352.70
1558.84
1574.84
1721.89
1805.97
1928.00
1956.03
1994.04
2008.05
2211.14
2239.17
2283.22
2807.32

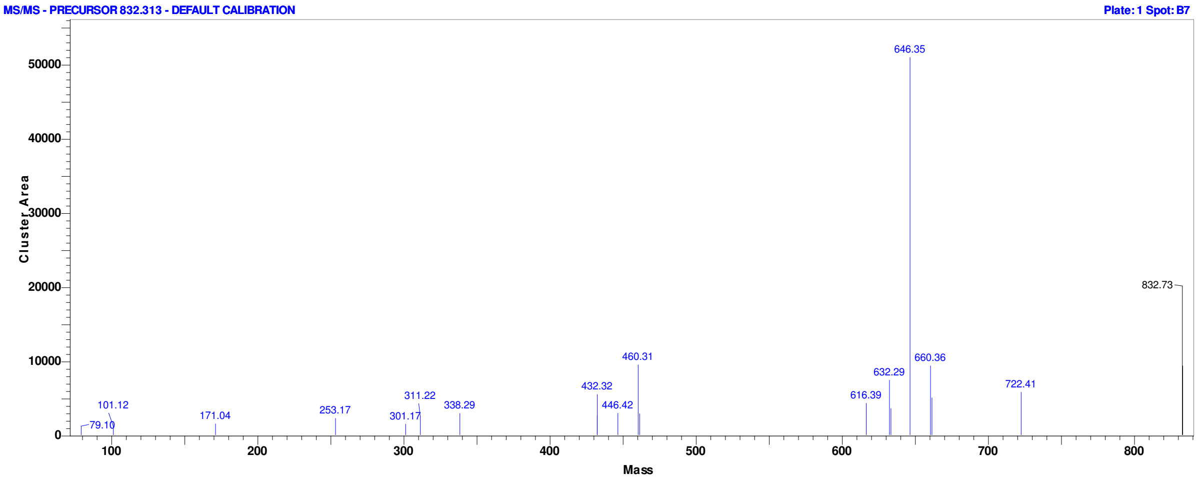
MS/MS - PRECURSOR 832.313 - DEFAULT CALIBRATION
Plate: 1 Spot: B7
Cluster Area
Mass
79.10
101.12
171.04
253.17
301.17
311.22
338.29
432.32
446.42
460.31
616.39
632.29
646.35
660.36
722.41
832.73

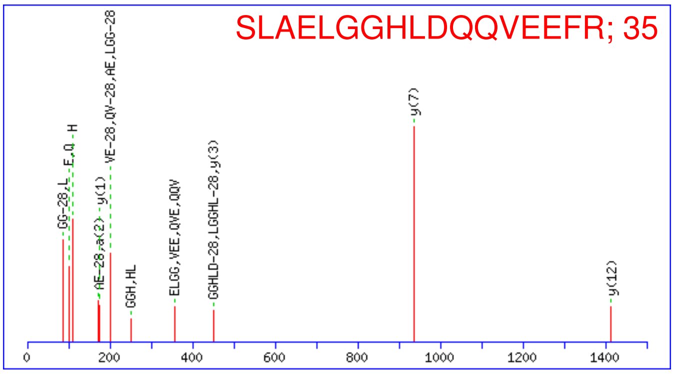
SLAELGGHLDQQVEEFR; 35
GG-28,L,E,Q
H
AE-28,a(2)
y(1)
VE-28,QV-28,AE,LGG-28
GGH,HL
ELGG,VEE,QVE,QQV
GGHLD-28,LGGHL-28,y(3)
y(7)
y(12)

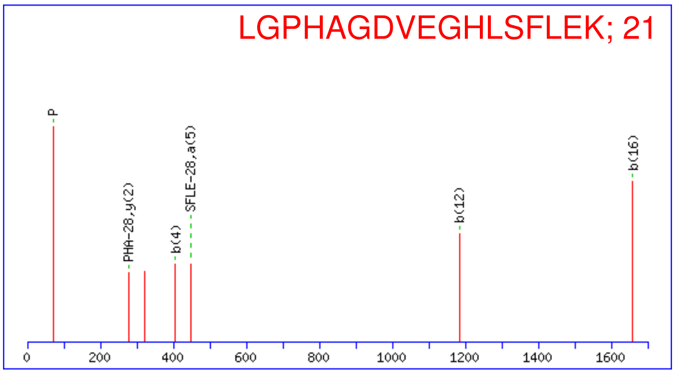
LGPHAGDVEGHLSFLEK; 21
P
PHA-28,y(2)
b(4)
SFLE-28,a(5)
b(12)
b(16)

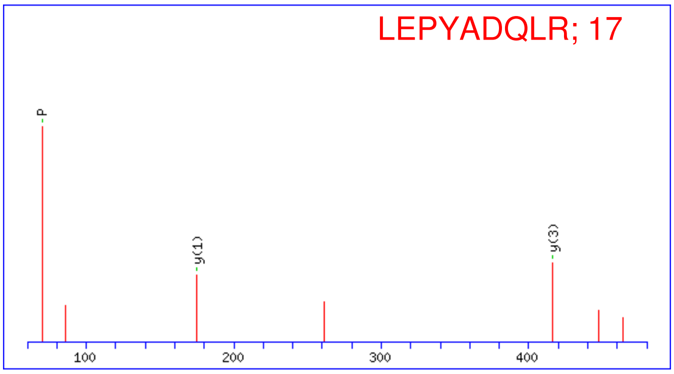
LEPYADQLR; 17
P
y(1)
y(3)

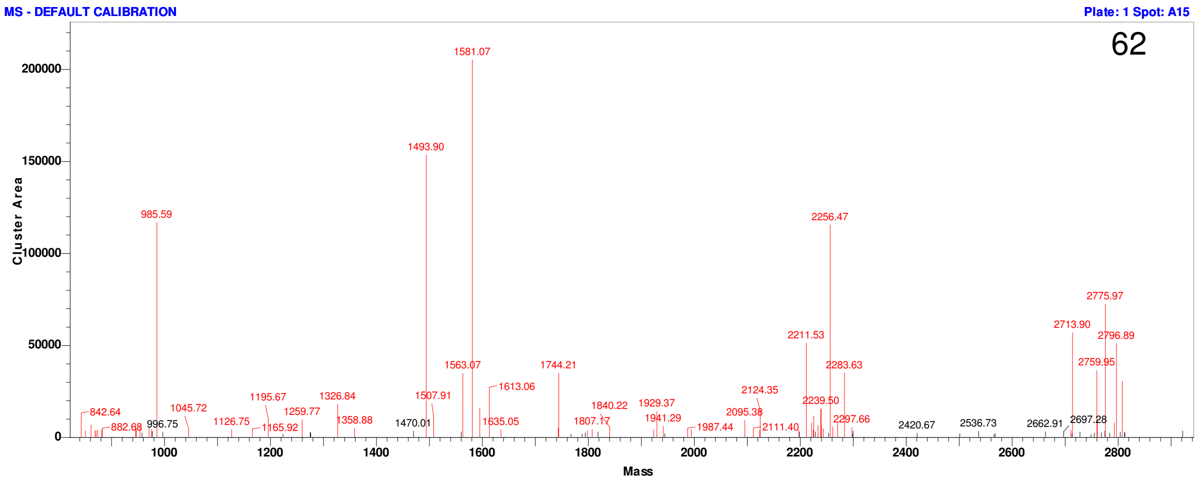
MS - DEFAULT CALIBRATION
Plate: 1 Spot: A15
62
Cluster Area
Mass
1581.07
1493.90
985.59
2256.47
2775.97
2713.90
2796.89
2211.53
2759.95
2283.63
1563.07
1744.21
1507.91
1613.06
2124.35
1195.67
1326.84
1045.72
842.64
1259.77
1840.22
1929.37
2239.50
2095.38
882.68
996.75
1126.75
1165.92
1358.88
1470.01
1635.05
1807.17
1941.29
1987.44
2111.40
2297.66
2420.67
2536.73
2662.91
2697.28
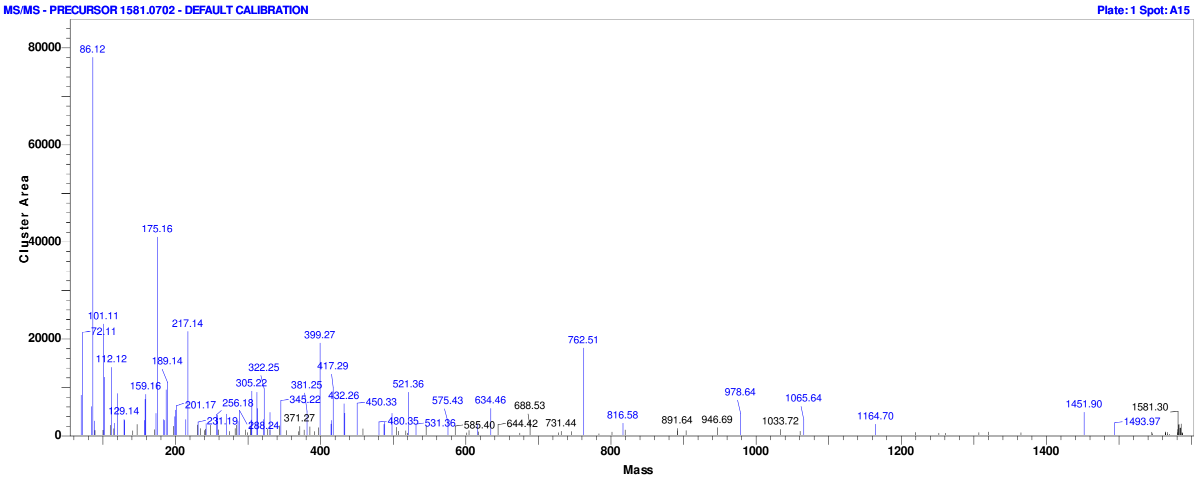
MS/MS - PRECURSOR 1581.0702 - DEFAULT CALIBRATION
Plate: 1 Spot: A15
Cluster Area
Mass
86.12
175.16
101.11
72.11
217.14
112.12
189.14
399.27
762.51
159.16
322.25
305.22
417.29
381.25
345.22
432.26
521.36
575.43
634.46
688.53
978.64
1065.64
1451.90
1581.30
1493.97
816.58
891.64
946.69
1033.72
1164.70
129.14
201.17
256.18
231.19
288.24
371.27
450.33
480.35
531.36
585.40
644.42
731.44
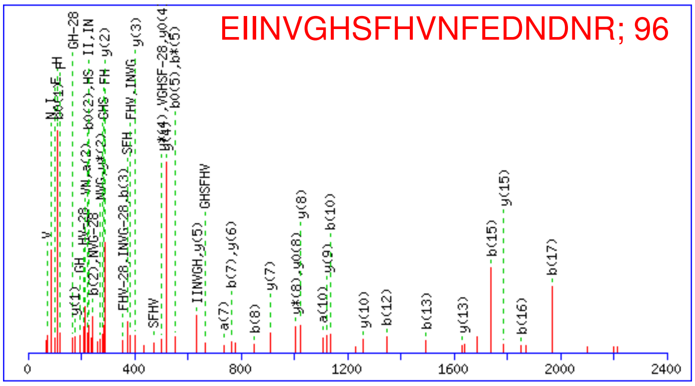
EIINVGHSFHVNFEDNDNR; 96
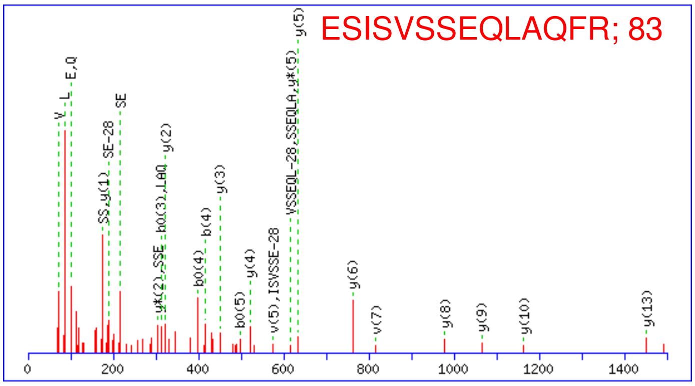
ESISVSSEQLAQFR; 83
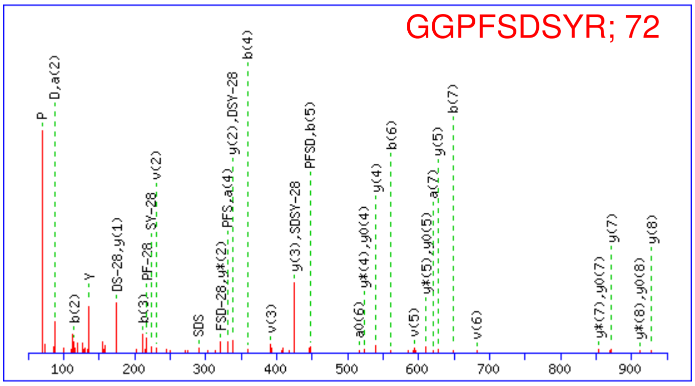
GGPFSDSYR; 72

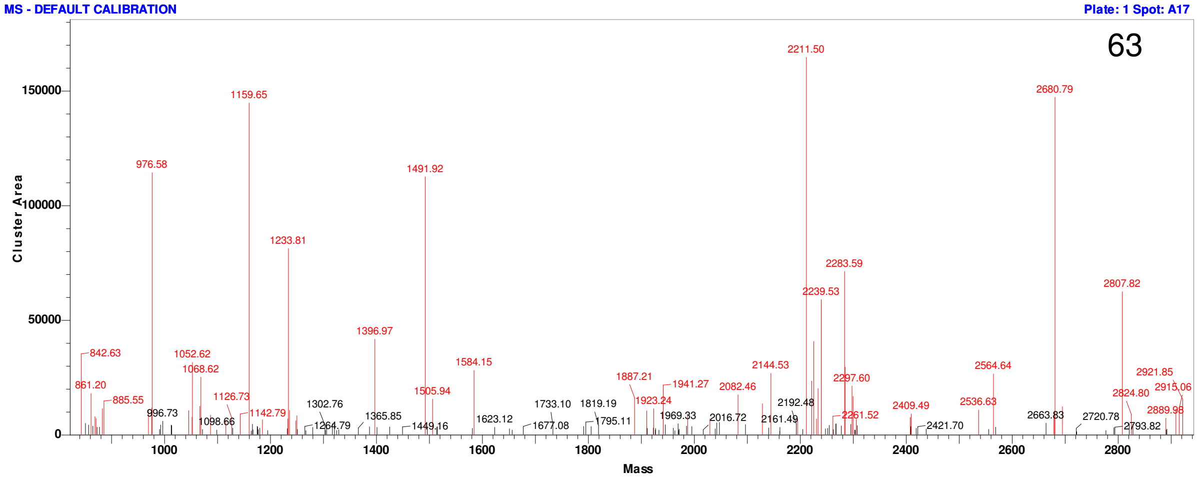

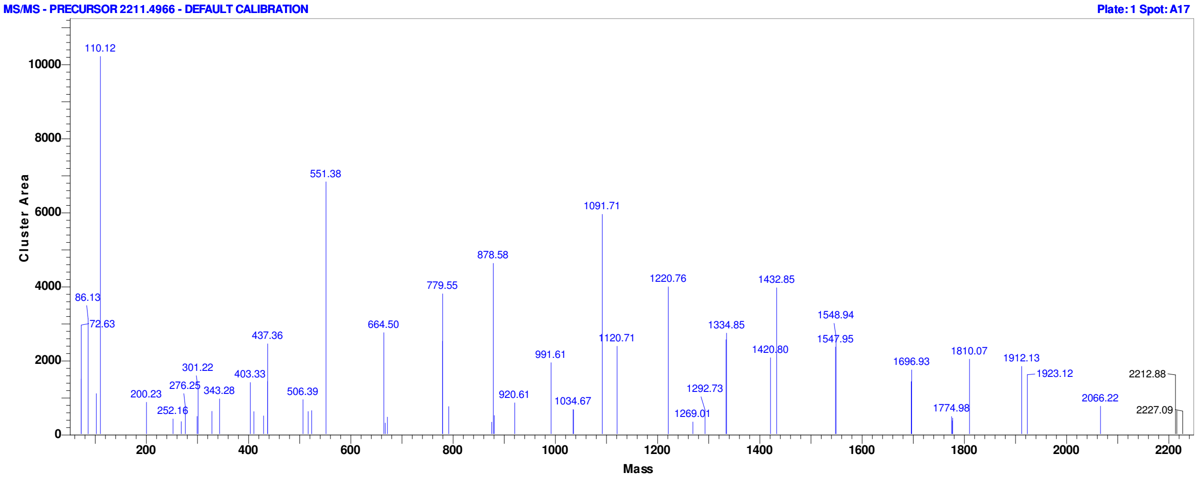

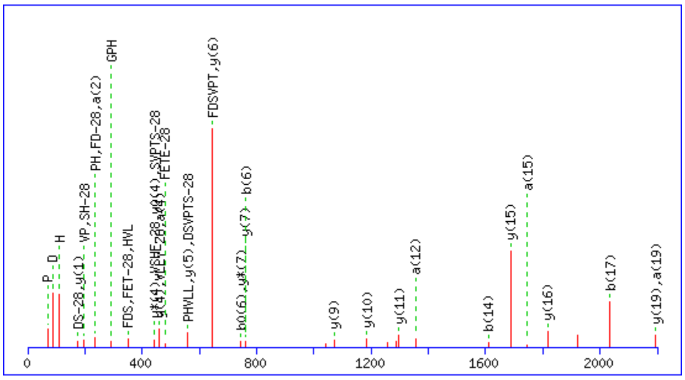

YVSHFETEGPHVLLYFDSVPTSR; 86

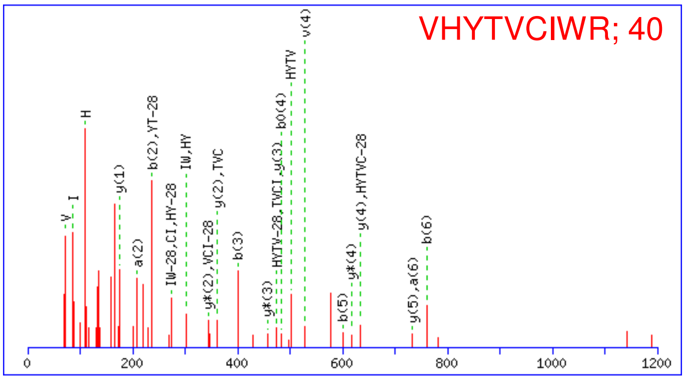

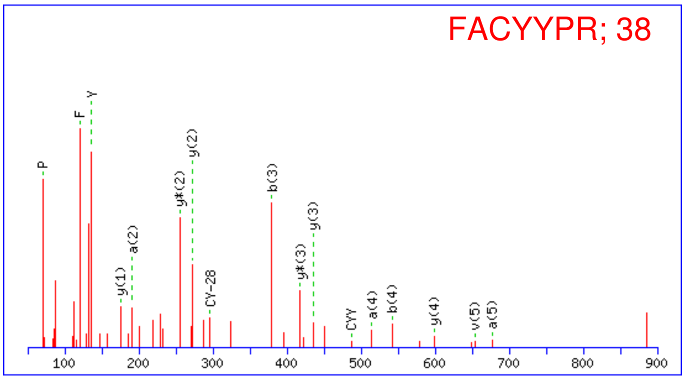

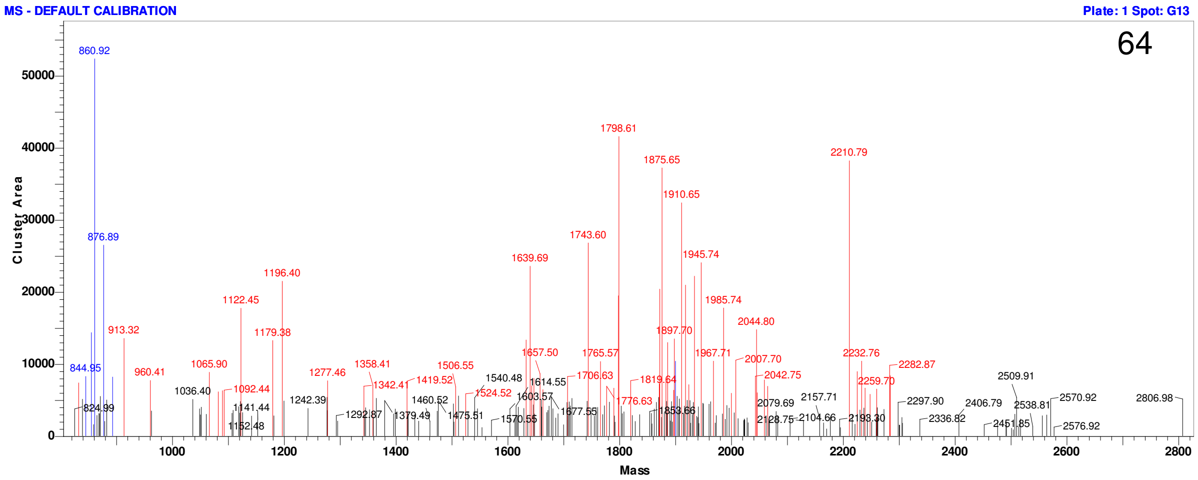
MS - DEFAULT CALIBRATION
Plate: 1 Spot: G13
64
Cluster Area
Mass
860.92
876.89
844.95
824.99
913.32
960.41
1036.40
1065.90
1092.44
1122.45
1141.44
1152.48
1179.38
1196.40
1242.39
1277.46
1292.87
1342.41
1358.41
1379.49
1419.52
1460.52
1475.51
1506.55
1524.52
1540.48
1570.55
1603.57
1614.55
1639.69
1657.50
1677.55
1706.63
1743.60
1765.57
1776.63
1798.61
1819.64
1853.66
1875.65
1897.70
1910.65
1945.74
1967.71
1985.74
2007.70
2042.75
2044.80
2079.69
2104.66
2128.75
2157.71
2193.30
2210.79
2232.76
2259.70
2282.87
2297.90
2336.82
2406.79
2451.85
2509.91
2538.81
2570.92
2576.92
2806.98

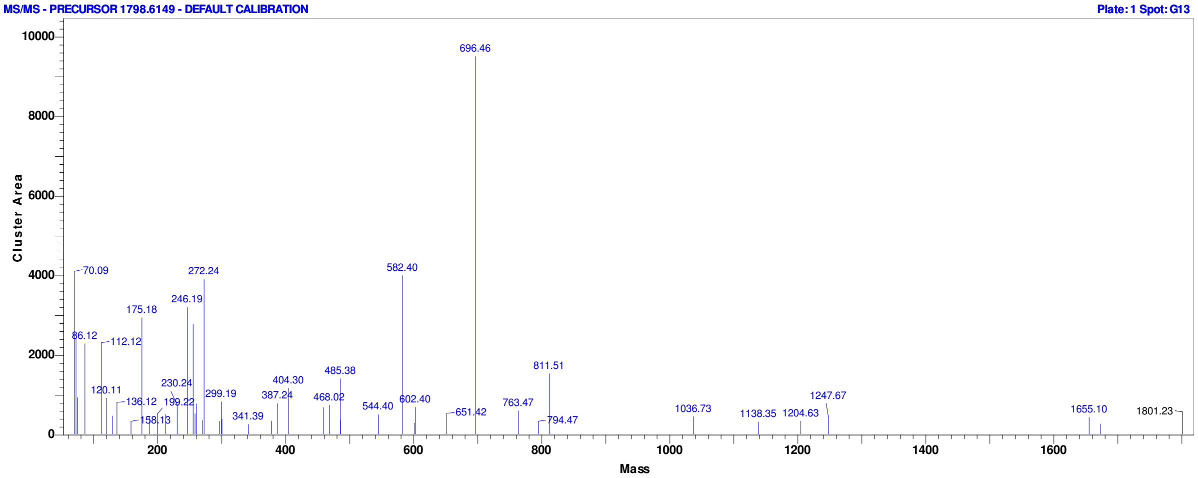
MS/MS - PRECURSOR 1798.6149 - DEFAULT CALIBRATION
Plate: 1 Spot: G13
Cluster Area
Mass
70.09
86.12
112.12
120.11
136.12
158.13
175.18
199.22
230.24
246.19
272.24
299.19
341.39
387.24
404.30
468.02
485.38
544.40
582.40
602.40
651.42
696.46
763.47
794.47
811.51
1036.73
1138.35
1204.63
1247.67
1655.10
1801.23

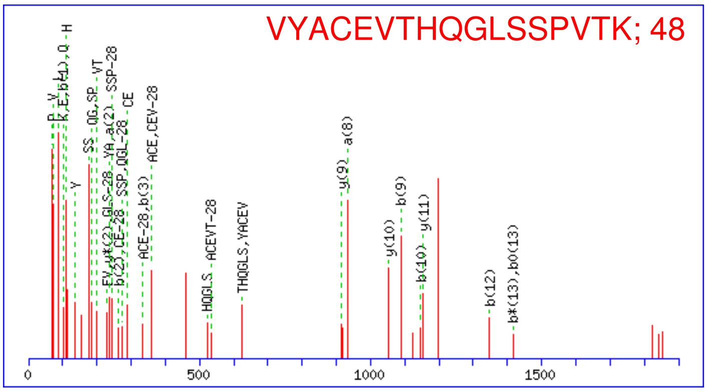
VYACEVTHQGLSSPVTK; 48
HQGLS
ACEVT-28
THQGLS,YACEV
y(9)
a(8)
y(10)
b(9)
b(10)
y(11)
b(12)
b*(13),b0(13)

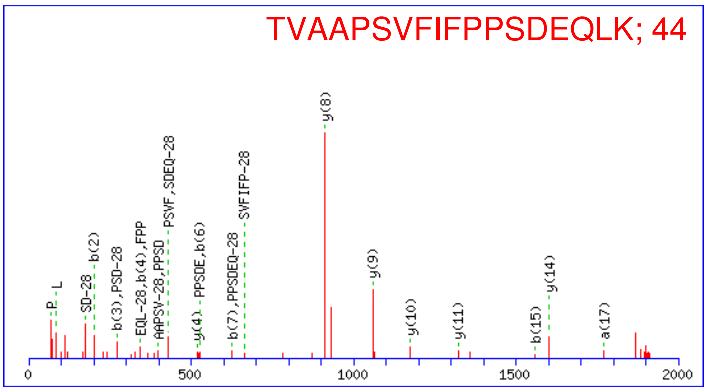
TVAAPSVFIFPPSDEQLK; 44
P
L
SD-28
b(2)
b(3),PSD-28
EQL-28,b(4),FPP
AAPSV-28,PPSD
PSVF,SDEQ-28
y(4)
PPSDE,b(6)
b(7),PPSDEQ-28
SVFIFP-28
y(8)
y(9)
y(10)
y(11)
b(15)
y(14)
a(17)

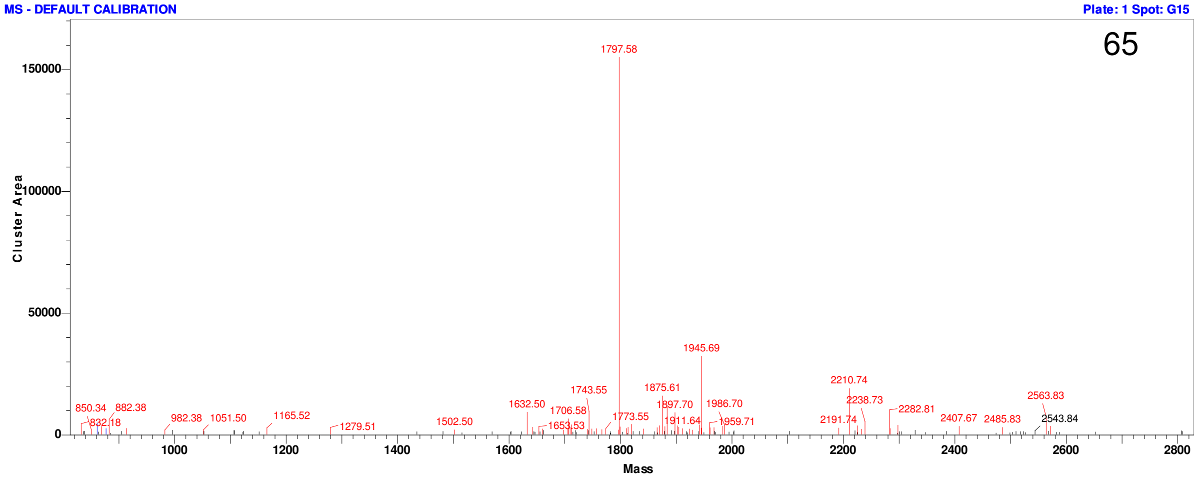
MS - DEFAULT CALIBRATION
Plate: 1 Spot: G15
65
Cluster Area
Mass
1797.58
1945.69
2210.74
2238.73
2563.83
2543.84
1875.61
1897.70
1986.70
1743.55
1632.50
1706.58
1773.55
1911.64
1959.71
1653.53
2282.81
2191.74
2407.67
2485.83
850.34
882.38
832.18
982.38
1051.50
1165.52
1279.51
1502.50

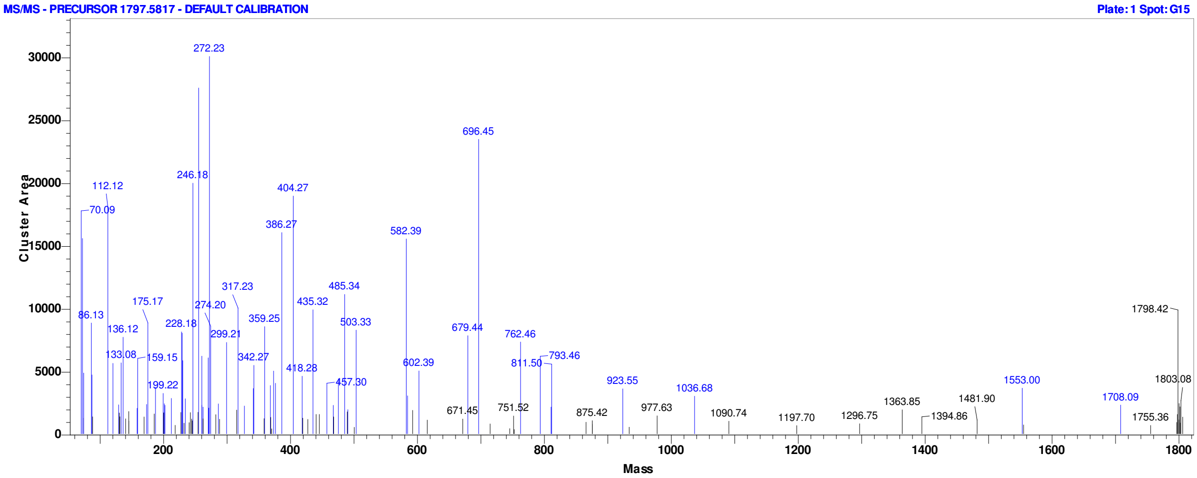
MS/MS - PRECURSOR 1797.5817 - DEFAULT CALIBRATION
Plate: 1 Spot: G15
Cluster Area
Mass
272.23
696.45
246.18
112.12
404.27
70.09
386.27
582.39
317.23
485.34
175.17
274.20
435.32
86.13
228.18
359.25
503.33
136.12
299.21
679.44
762.46
133.08
159.15
342.27
793.46
811.50
602.39
418.28
457.30
199.22
923.55
1036.68
1553.00
1798.42
1803.08
1708.09
1363.85
1481.90
1394.86
1755.36
671.45
751.52
875.42
977.63
1090.74
1197.70
1296.75

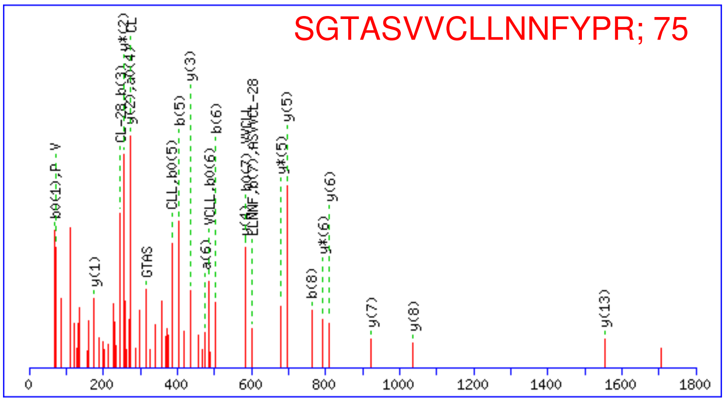
SGTASVVCLLNNFYPR; 75

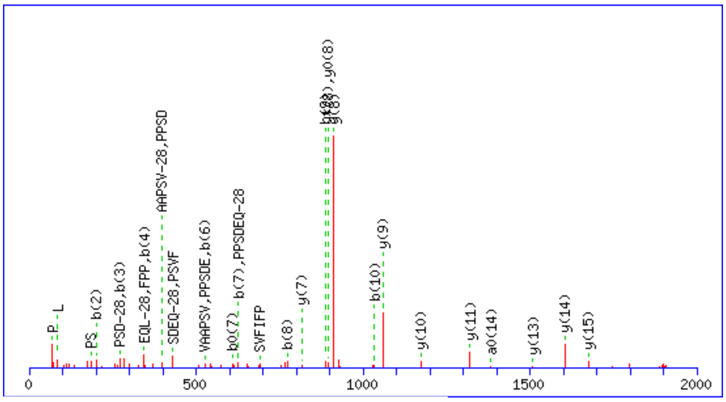
TVAAPSVFIFPPSDEQLK; 57

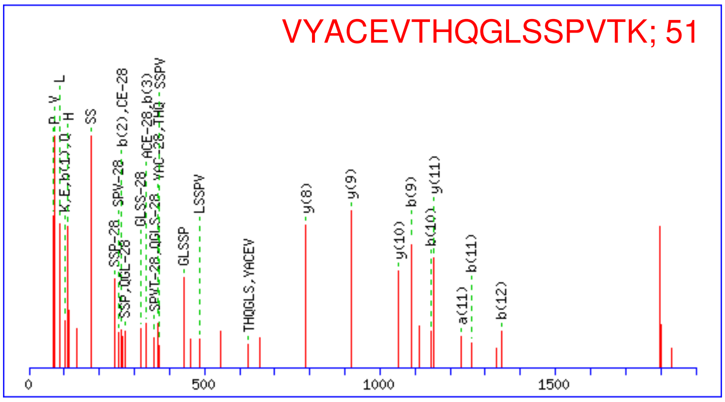
VYACEVTHQGLSSPVTK; 51

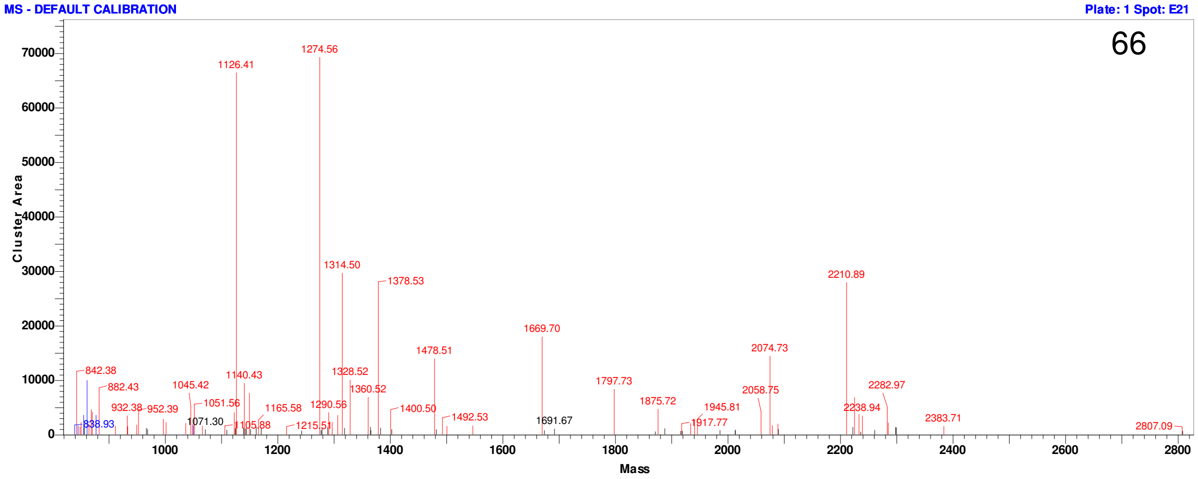

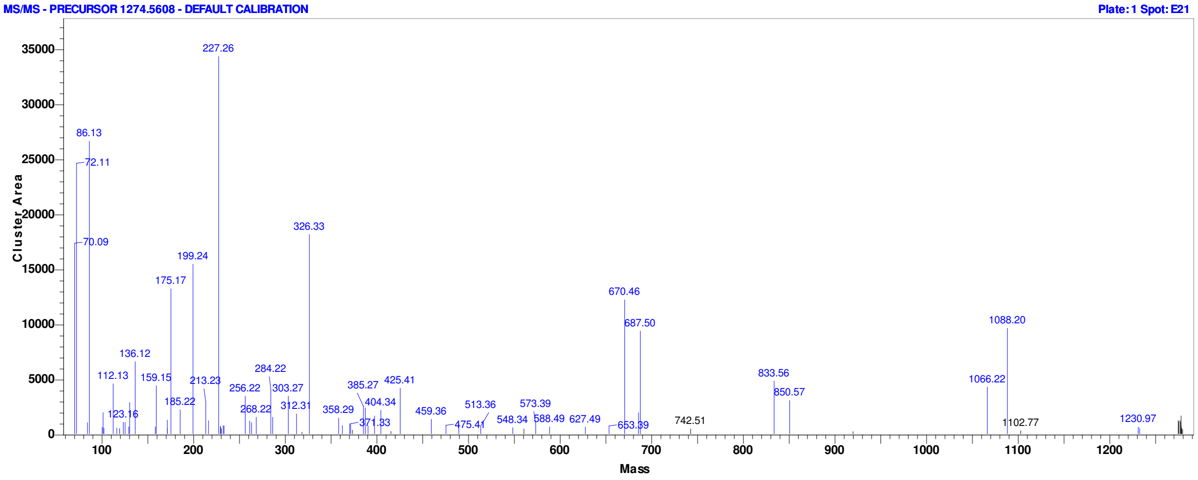

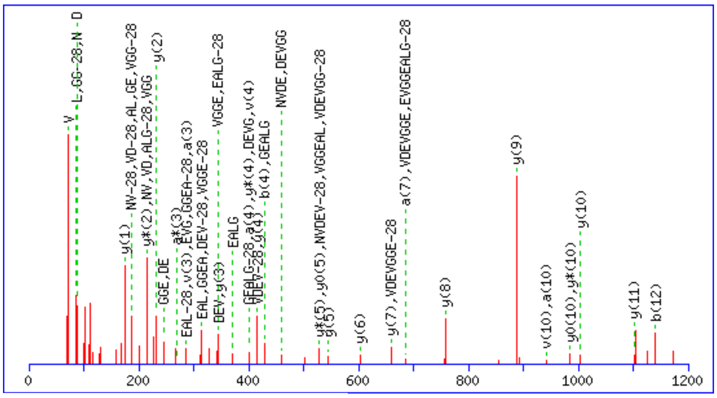

VNVDEVGGEALGR; 99

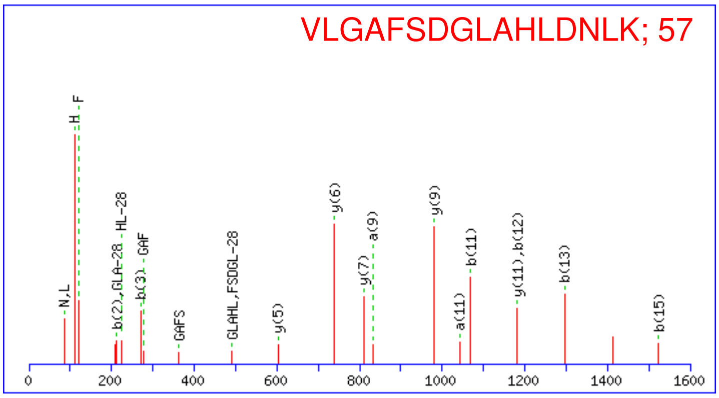

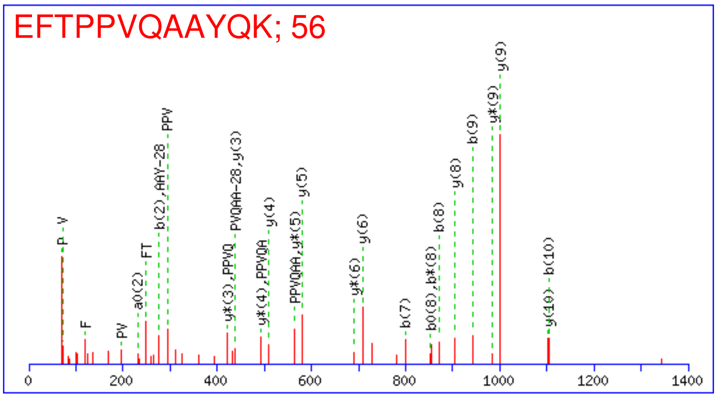

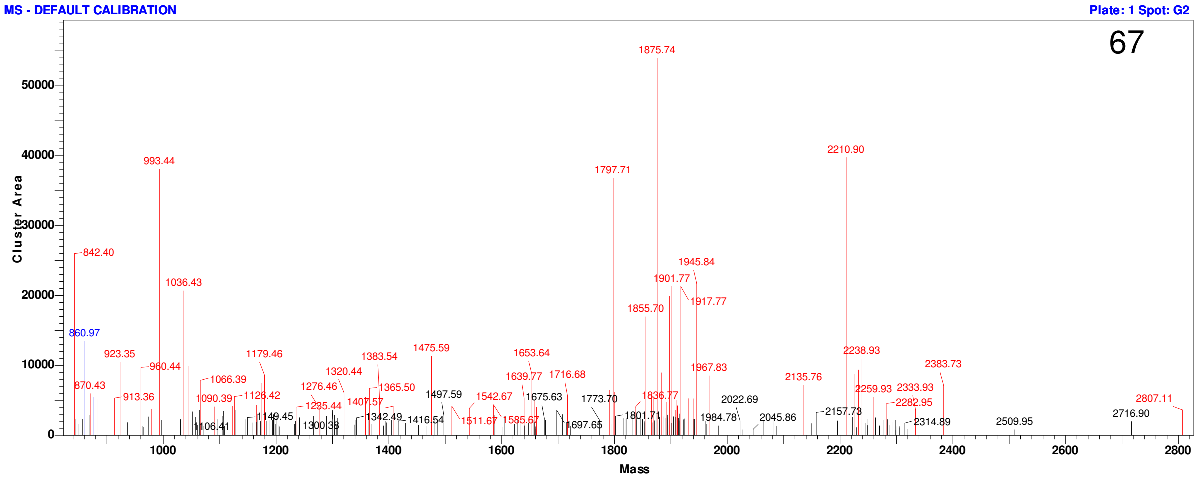

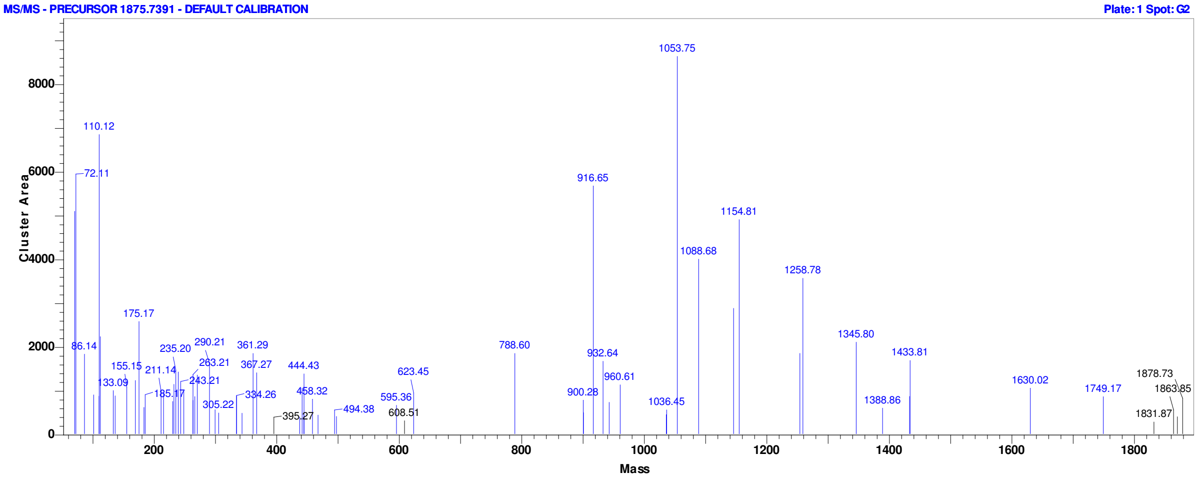

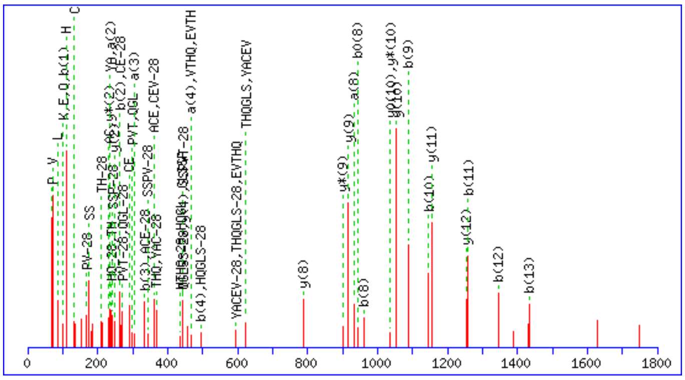

VYACEVTHQGLSSPVTK; 92

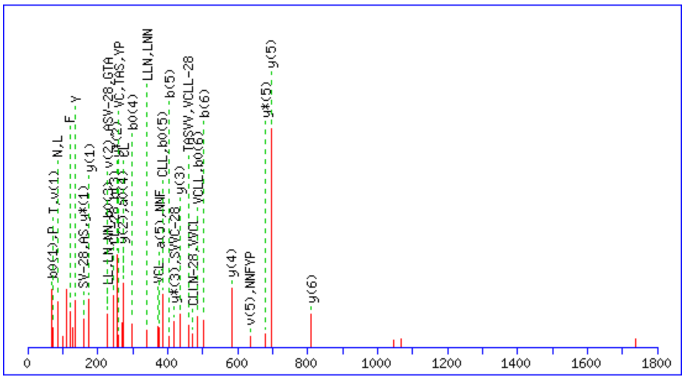

SGTASVVCLLNNFYPR; 40

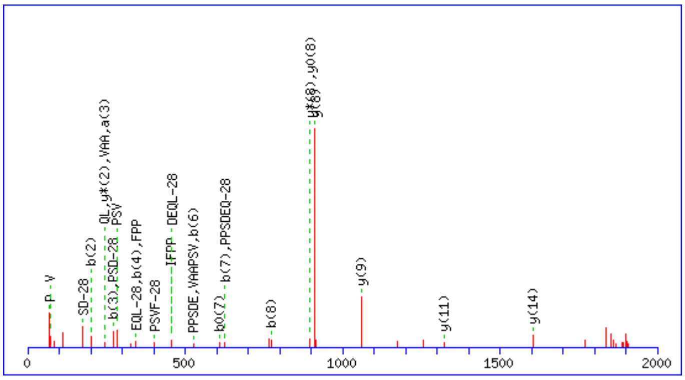

TVAAPSVFIFPPSDEQLK; 31

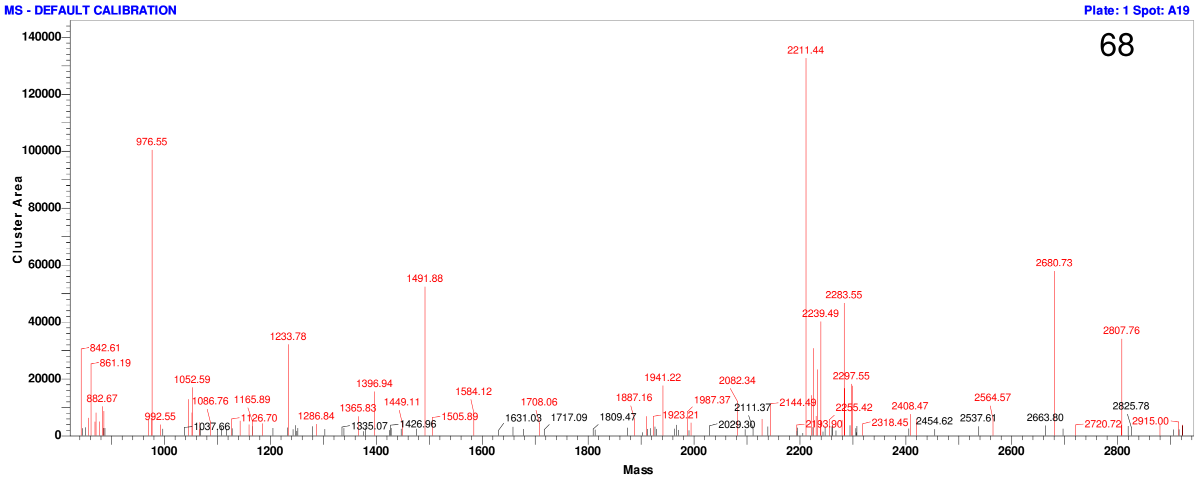

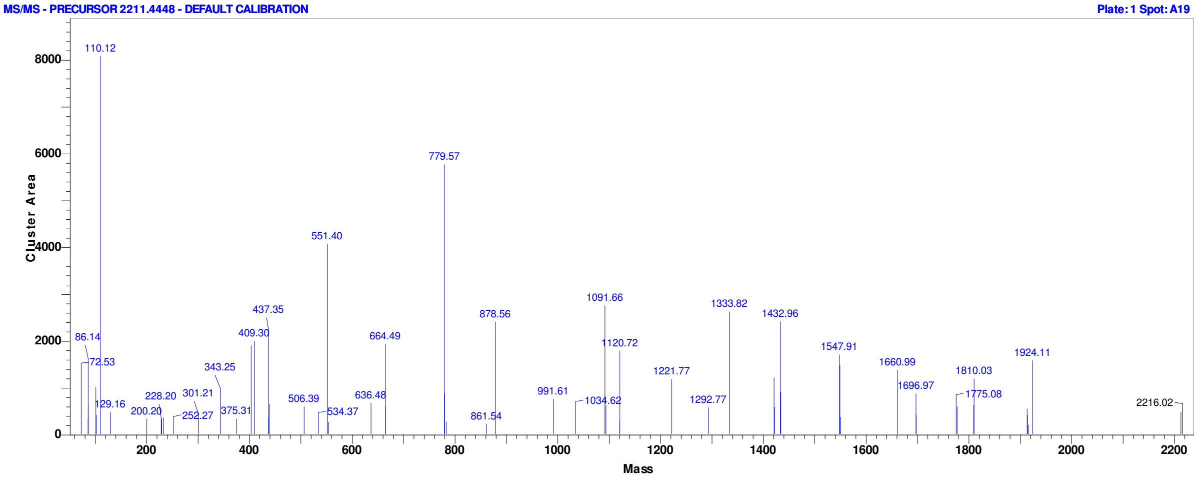

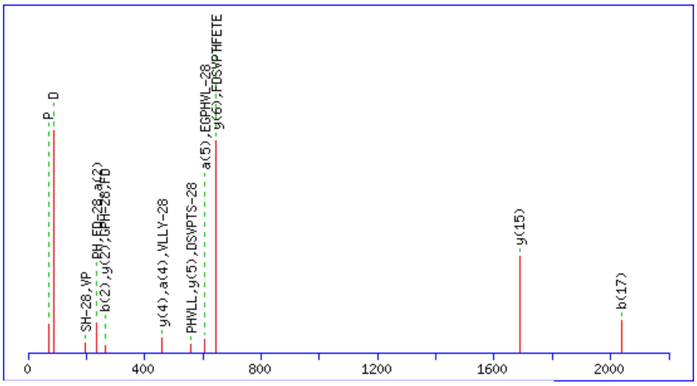

YVSHFETEGPHVLLYFDSVPTSR; 42

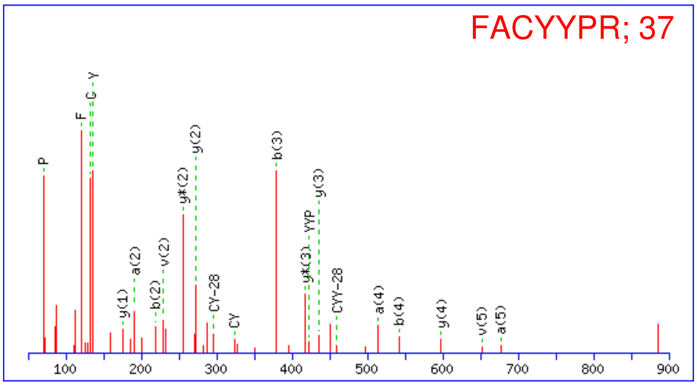

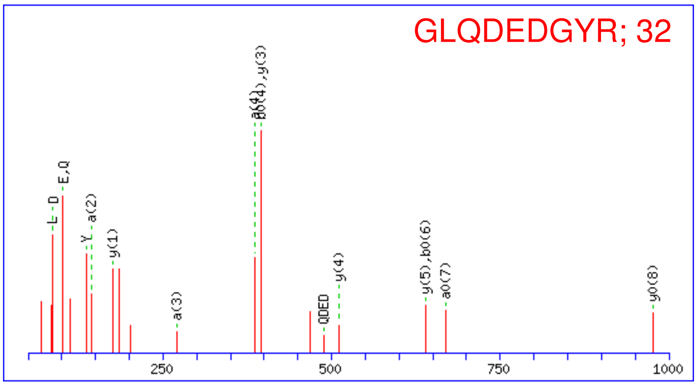

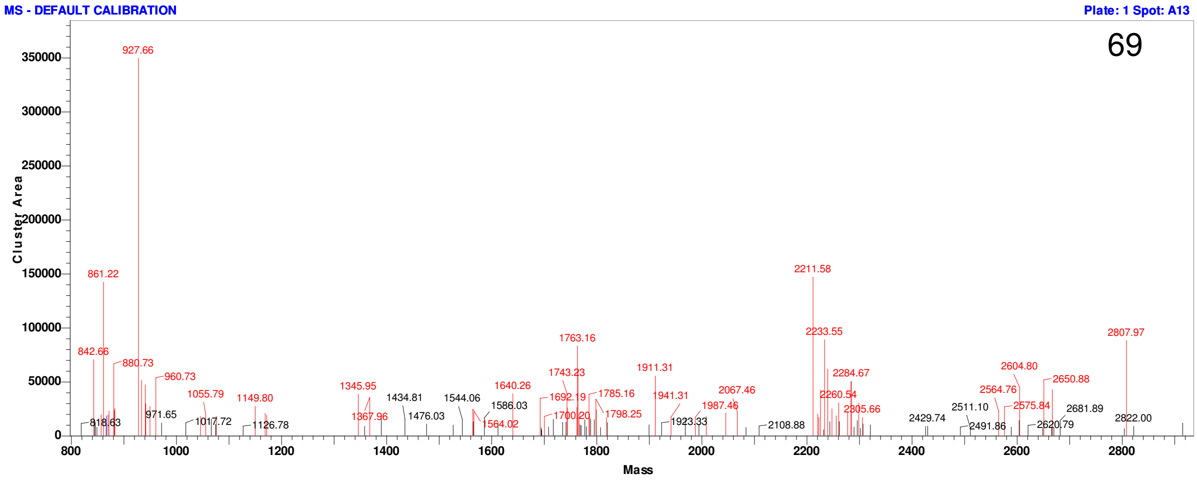

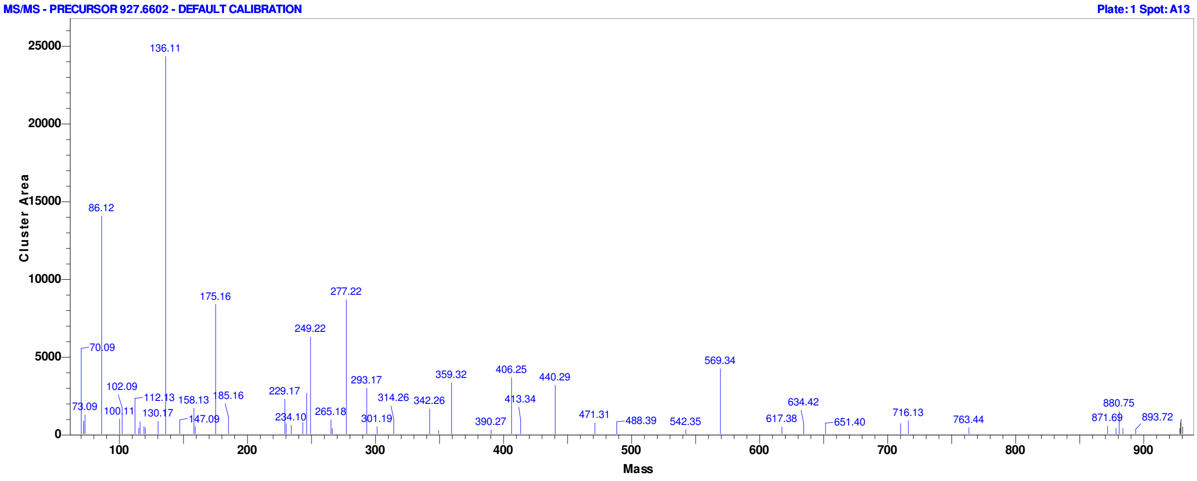

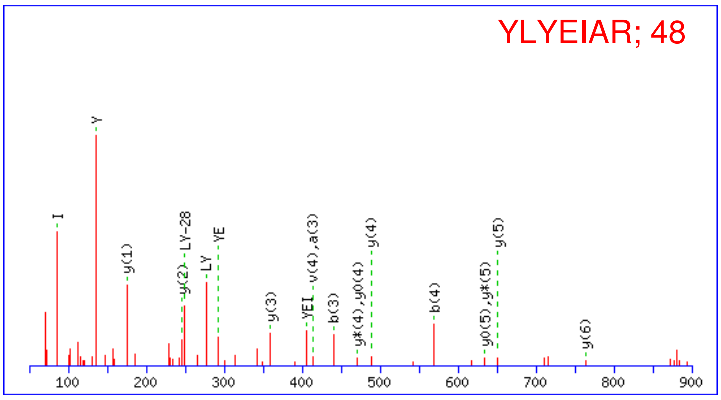

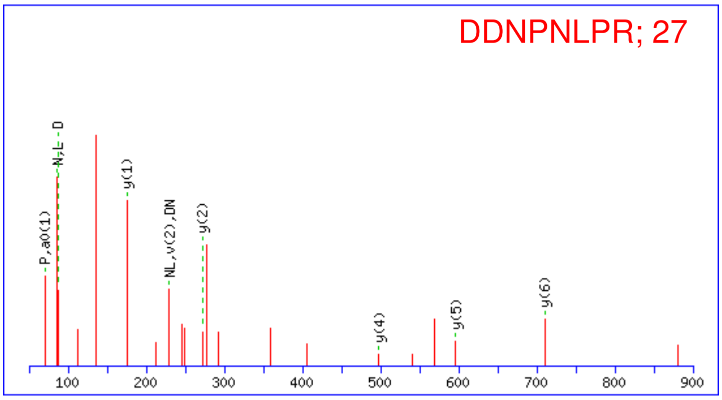

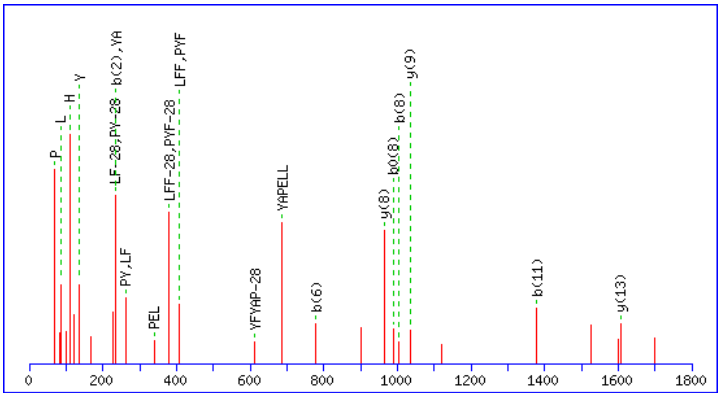

HPYFYAPELLFFAK; 23

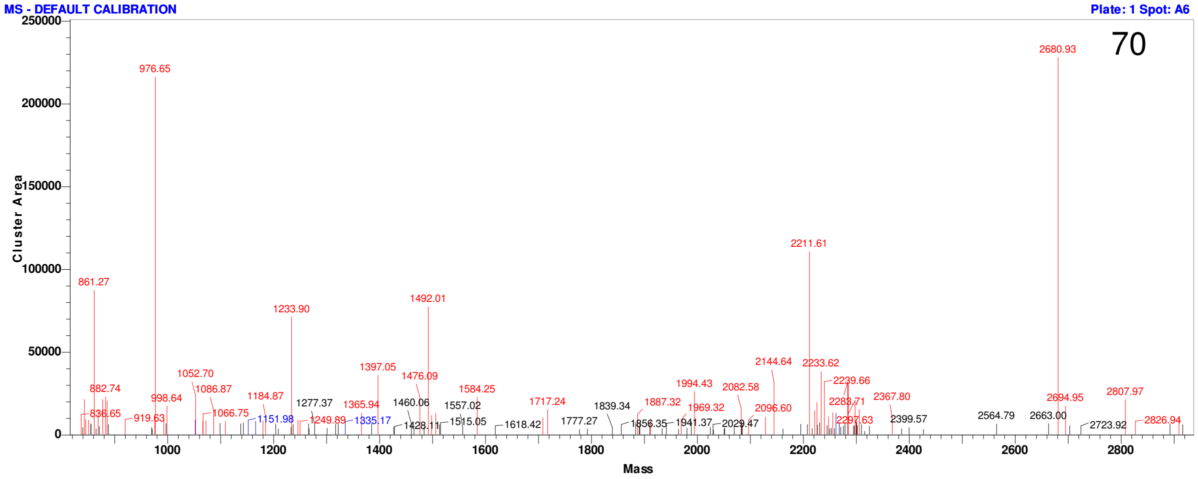
MS - DEFAULT CALIBRATION
Plate: 1 Spot: A6
70
Cluster Area
Mass
2680.93
976.65
2211.61
861.27
1492.01
1233.90
2144.64
2233.62
1397.05
1476.09
1052.70
882.74
1584.25
1994.43
2082.58
2239.66
2807.97
1717.24
998.64
1086.87
1184.87
1277.37
1365.94
1460.06
1557.02
1839.34
1887.32
1969.32
2096.60
2268.71
2367.80
2694.95
836.65
1066.75
919.63
1151.98
1249.89
1335.17
1428.11
1515.05
1618.42
1777.27
1856.35
1941.37
2029.47
2297.63
2399.57
2564.79
2663.00
2723.92
2826.94

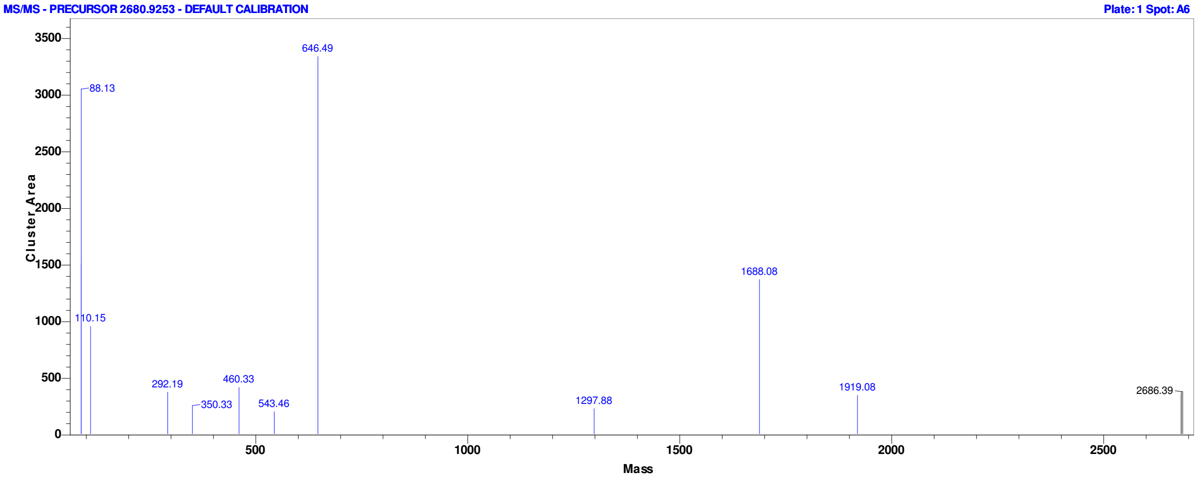
MS/MS - PRECURSOR 2680.9253 - DEFAULT CALIBRATION
Plate: 1 Spot: A6
Cluster Area
Mass
646.49
88.13
1688.08
110.15
292.19
460.33
350.33
543.46
1297.88
1919.08
2686.39

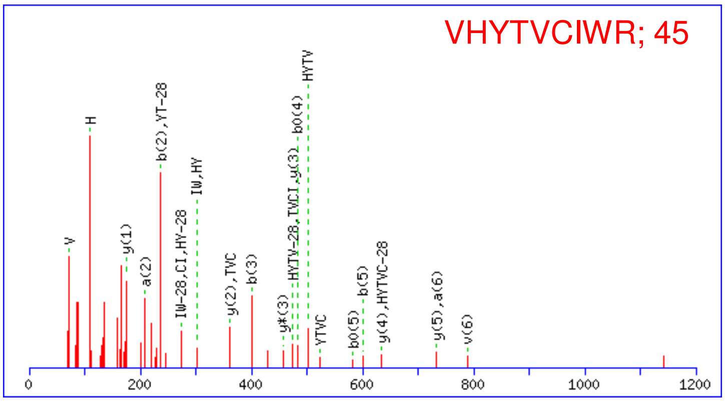
VHYTVCIWR; 45
V
H
y(1)
a(2)
b(2),YT-28
IW-28,CI,HY-28
IW,HY
y(2),TVC
b(3)
y*(3)
HYTV-28,TVCI,y(3)
b0(4)
HYTV
YTVC
b0(5)
b(5)
y(4),HYTVC-28
y(5),a(6)
y(6)

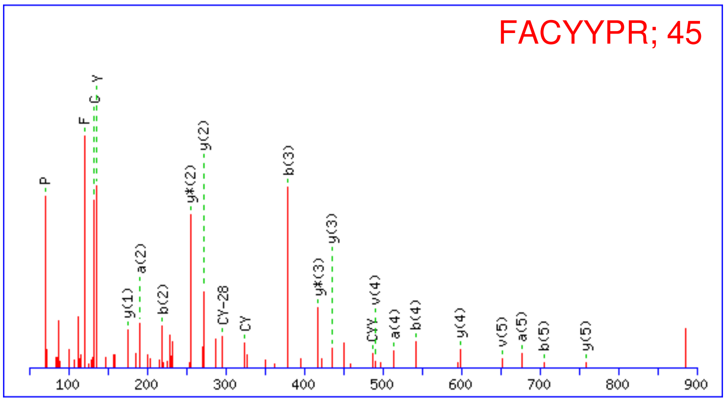
FACYYPR; 45
P
F
C
Y
y(1)
a(2)
b(2)
y*(2)
y(2)
CY-28
CY
b(3)
y*(3)
y(3)
CYY
y(4)
a(4)
b(4)
y(4)
y(5)
a(5)
b(5)
y(5)

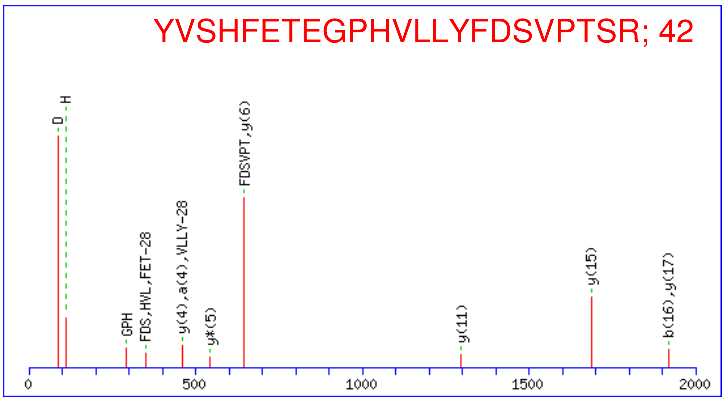
YVSHFETEGPHVLLYFDSVPTSR; 42
D
H
GPH
FDS,HVL,FET-28
y(4),a(4),VLLY-28
y*(5)
FDSVPT,y(6)
y(11)
y(15)
b(16),y(17)

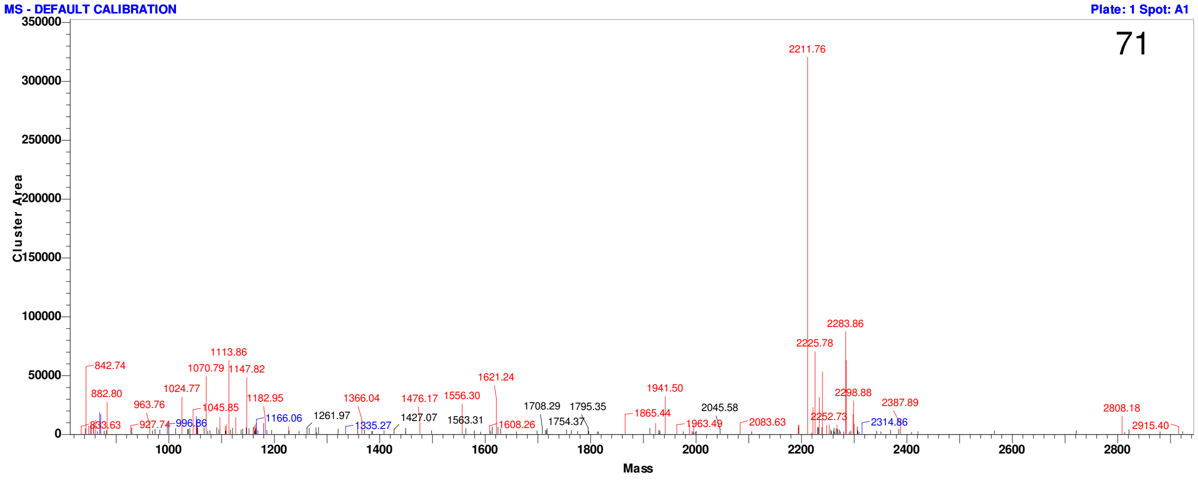

MS - DEFAULT CALIBRATION
Plate: 1 Spot: A1
71
Cluster Area
Mass
2211.76
2283.86
2225.78
1113.86
842.74
1070.79
1147.82
1621.24
1024.77
882.80
963.76
1182.95
1366.04
1476.17
1556.30
1941.50
2298.88
2387.89
1045.85
1166.06
1261.97
1708.29
1795.35
1865.44
2045.58
2252.73
2808.18
833.63
927.74
996.86
1335.27
1427.07
1563.31
1608.26
1754.37
1963.49
2083.63
2314.86
2915.40

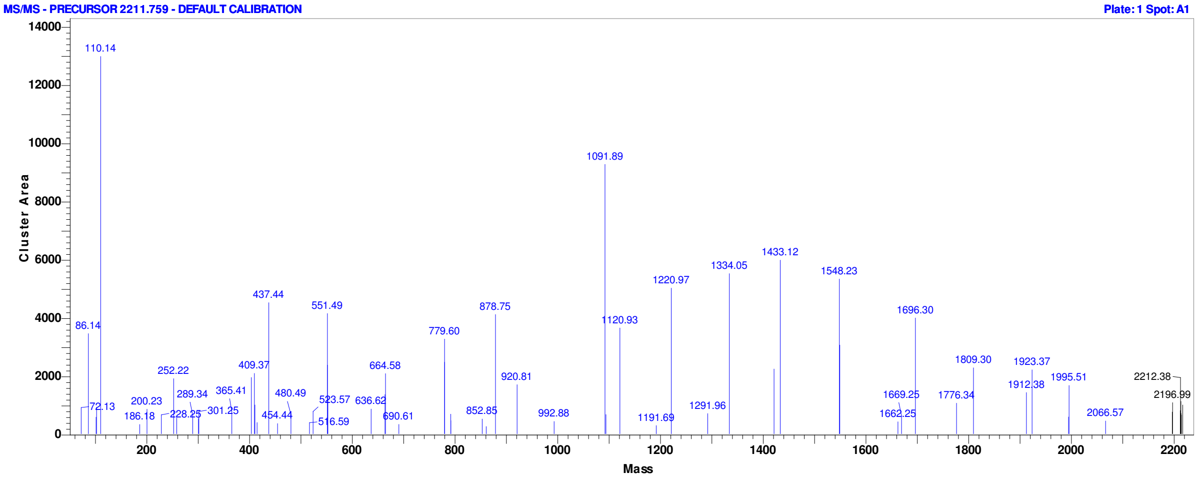

MS/MS - PRECURSOR 2211.759 - DEFAULT CALIBRATION
Plate: 1 Spot: A1
Cluster Area
Mass
110.14
1091.89
1433.12
1334.05
1548.23
1220.97
437.44
551.49
878.75
1696.30
1120.93
779.60
86.14
252.22
409.37
664.58
920.81
1809.30
1923.37
1995.51
2212.38
2196.99
289.34
365.41
480.49
523.57
636.62
1669.25
1776.34
1912.38
72.13
200.23
301.25
1291.96
852.85
992.88
1662.25
2066.57
186.18
228.25
454.44
516.59
690.61
1191.69

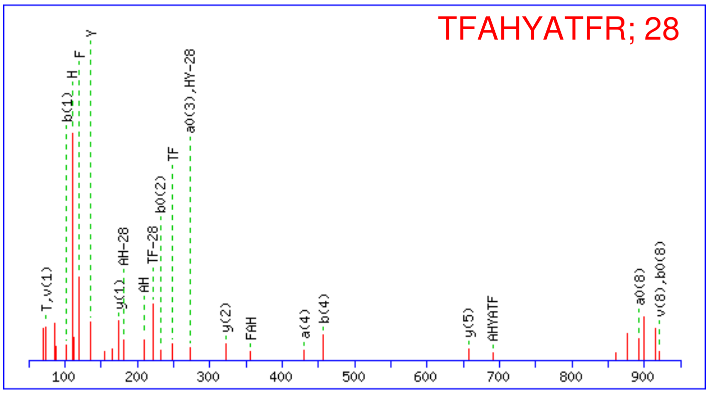

TFAHYATFR; 28
T,v(1)
b(1)
H
F
Y
y(1)
AH-28
AH
TF-28
b0(2)
TF
a0(3),HY-28
y(2)
FAH
a(4)
b(4)
y(5)
AHYATF
a0(8)
v(8),b0(8)

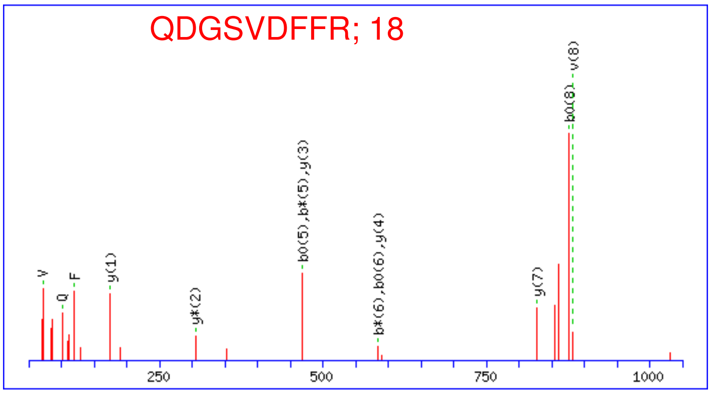

QDGSVDFFR; 18
V
Q
F
y(1)
y*(2)
b0(5),b*(5),y(3)
b*(6),b0(6),y(4)
y(7)
b0(8)
v(8)

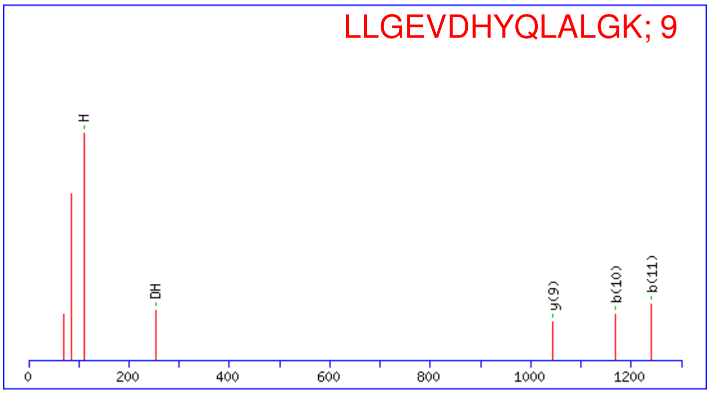

LLGEVDHYQLALGK; 9
H
DH
y(9)
b(10)
b(11)

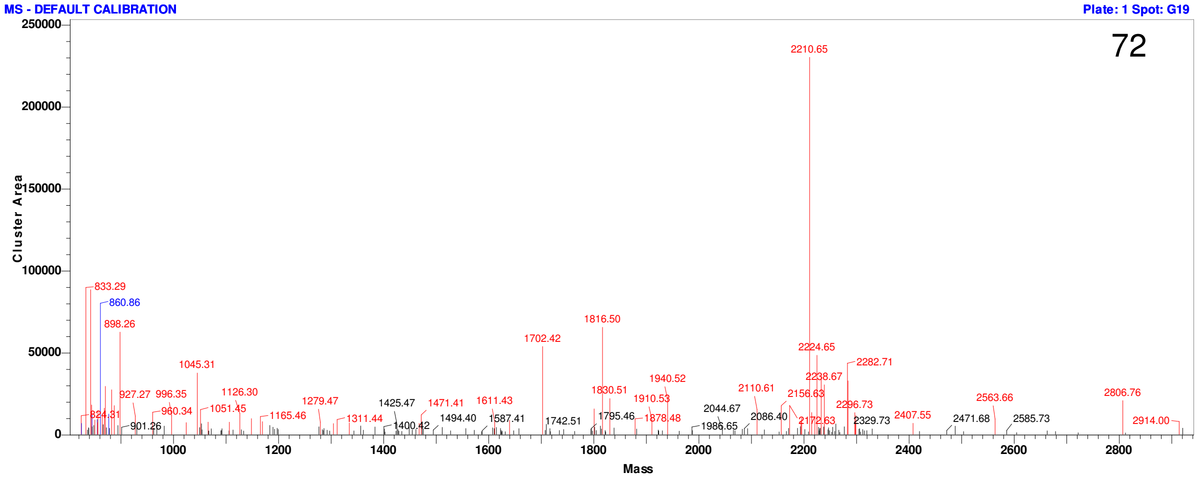
MS - DEFAULT CALIBRATION
Plate: 1 Spot: G19
72
Cluster Area
Mass
2210.65
833.29
860.86
898.26
1816.50
1702.42
2224.65
2282.71
2238.67
1045.31
1940.52
1830.51
2110.61
2156.63
1126.30
927.27
996.35
1279.47
1425.47
1471.41
1611.43
1910.53
2044.67
2563.66
2806.76
2296.73
1051.45
960.34
824.31
1165.46
1311.44
1494.40
1587.41
1742.51
1795.46
1878.48
2086.40
2407.55
2471.68
2585.73
2914.00
901.26
1400.42
1986.65
2172.63
2329.73

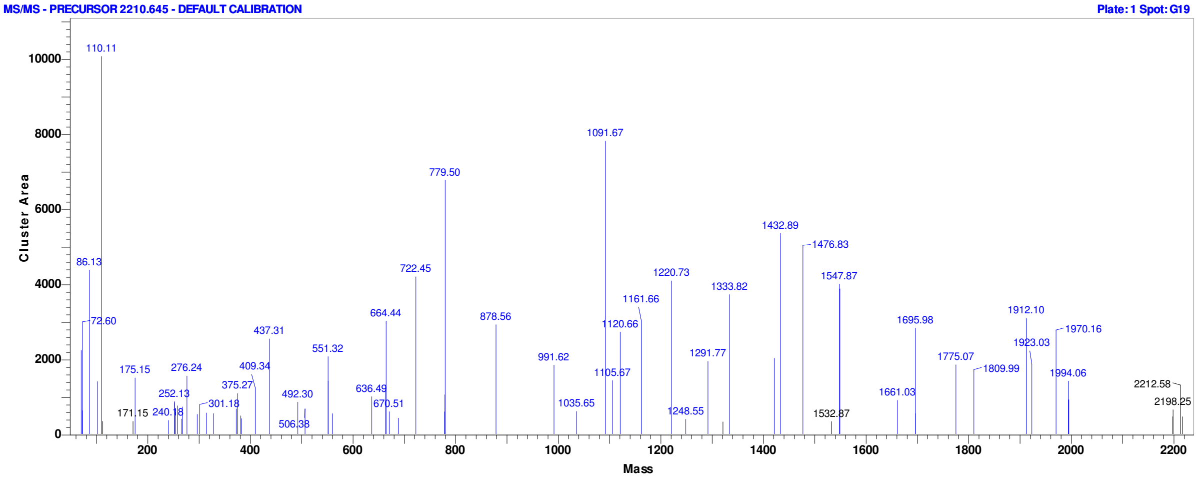
MS/MS - PRECURSOR 2210.645 - DEFAULT CALIBRATION
Plate: 1 Spot: G19
Cluster Area
Mass
110.11
1091.67
779.50
1432.89
1476.83
86.13
722.45
1220.73
1547.87
1333.82
1161.66
664.44
878.56
1120.66
72.60
1695.98
1912.10
1970.16
437.31
1923.03
551.32
1291.77
991.62
1775.07
1809.99
175.15
276.24
409.34
1105.67
1994.06
375.27
252.13
492.30
636.49
1661.03
2212.58
301.18
670.51
1035.65
2198.25
171.15
240.18
1248.55
1532.87
506.38

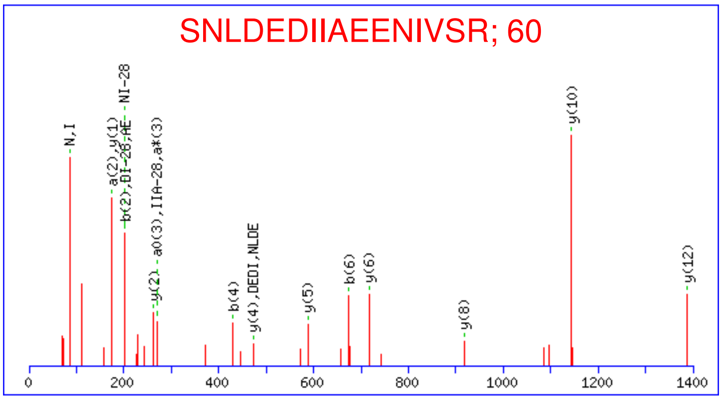
SNLDEDIIAEENIVSR; 60
N,I
a(2),y(1)
b(2),DI-28,AE
NI-28
a0(3),IIA-28,a*(3)
y(2)
b(4)
y(4),DEDI,NLDE
y(5)
b(6)
y(6)
y(8)
y(10)
y(12)

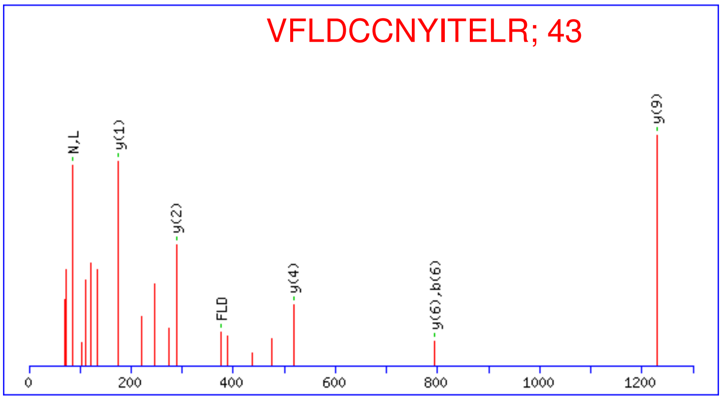
VFLDCCNYITELR; 43
N,L
y(1)
y(2)
FLD
y(4)
y(6),b(6)
y(9)

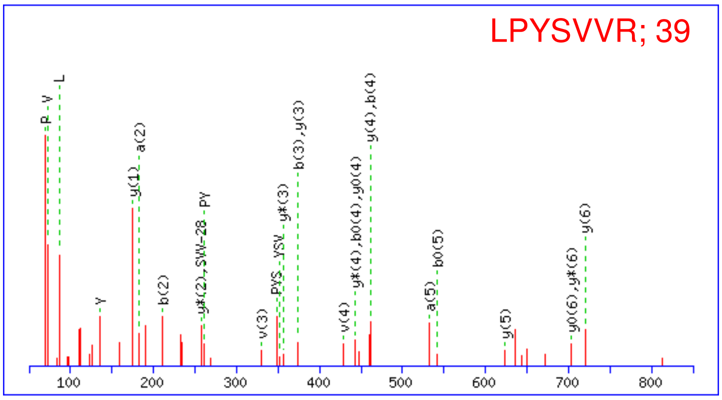
LPYSVVR; 39
P
V
L
Y
y(1)
a(2)
b(2)
y*(2),SVV-28
PY
v(3)
PYS
YSV
y*(3)
b(3),y(3)
v(4)
y*(4),b0(4),y0(4)
y(4),b(4)
a(5)
b0(5)
y(5)
y0(6),y*(6)
y(6)

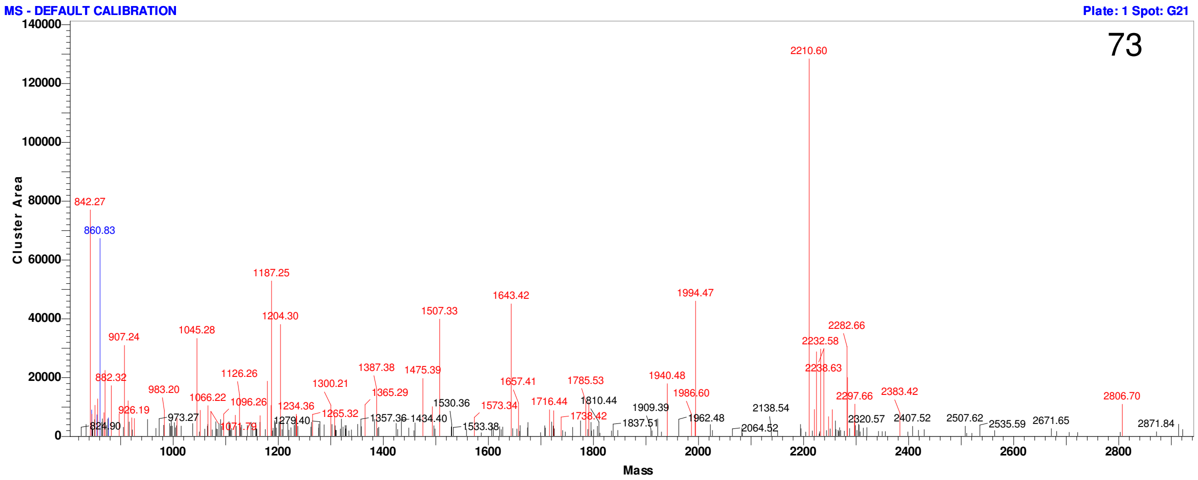

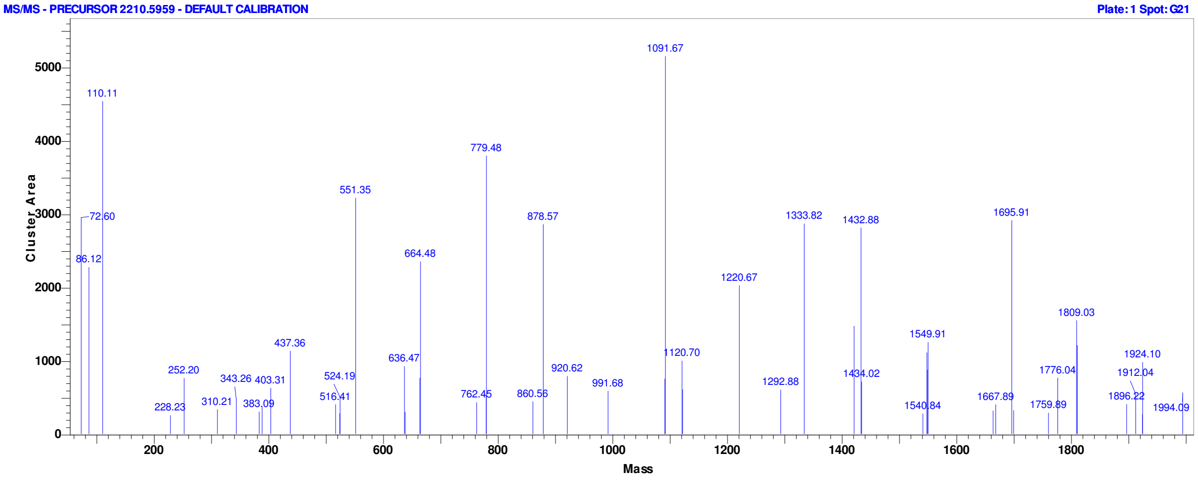

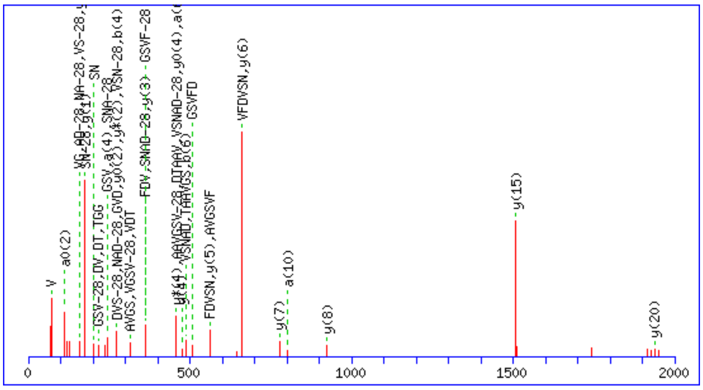

GTGGVDTAAVGSVFDVSNADR; 63

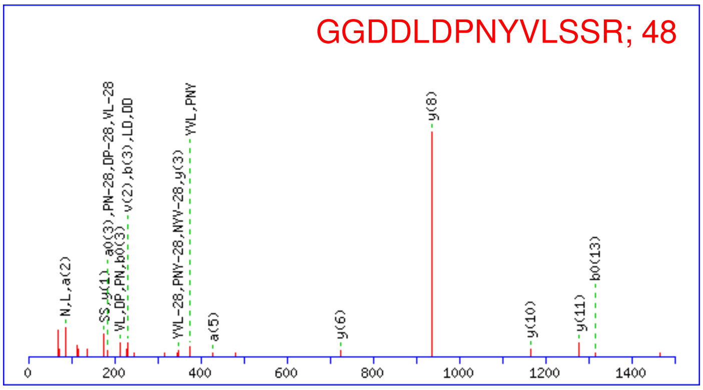

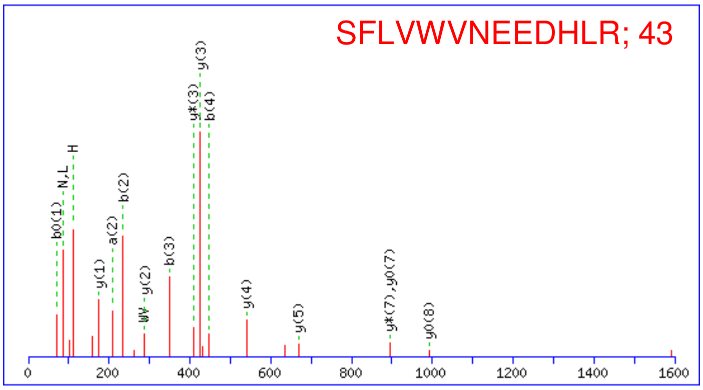

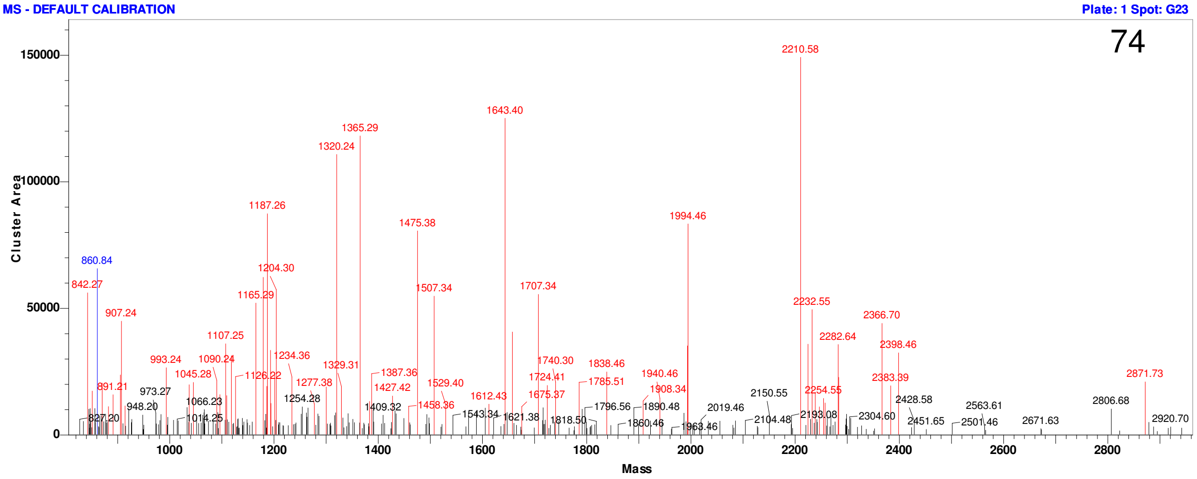

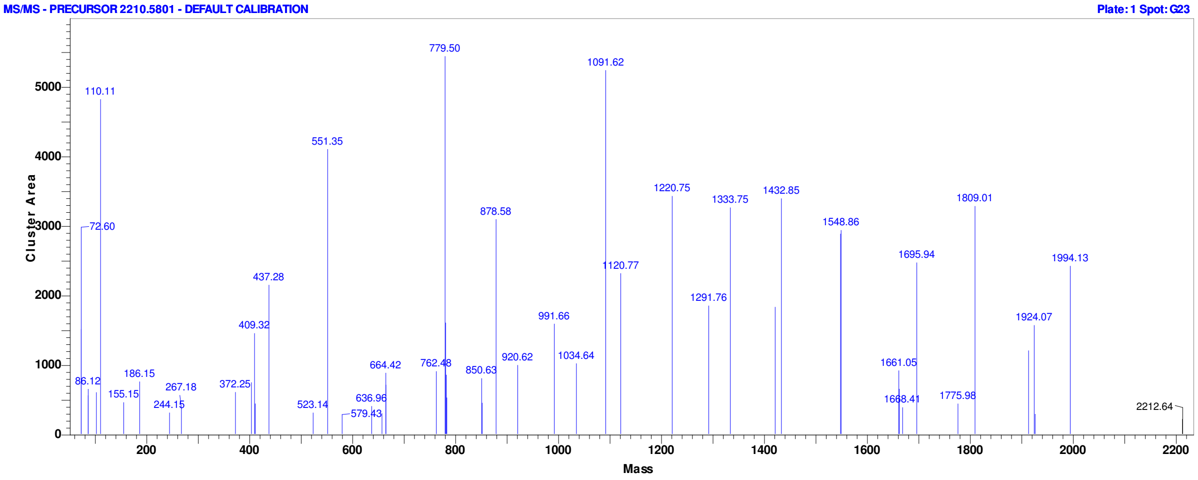

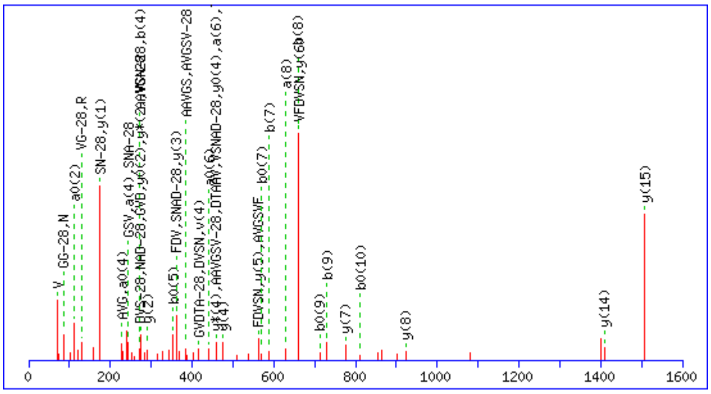

GTGGVDTAAVGSVFDVSNADR; 70

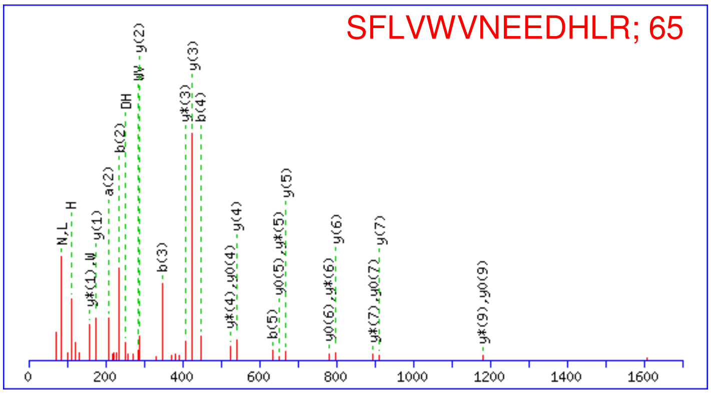

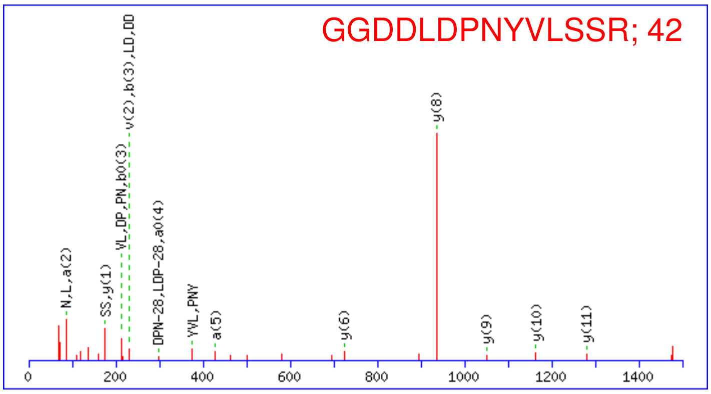

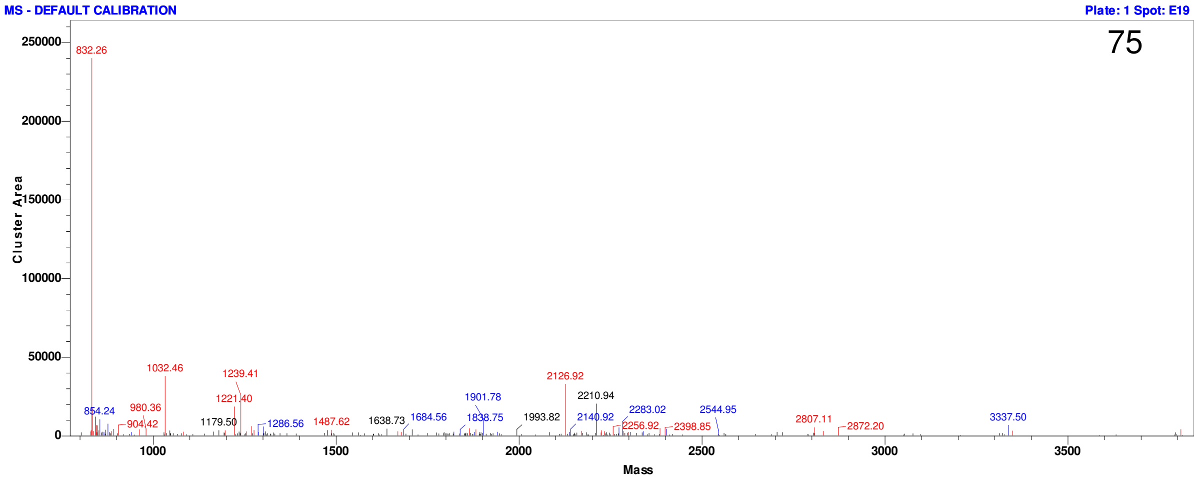

MS - DEFAULT CALIBRATION
Plate: 1 Spot: E19
75
Cluster Area
Mass
832.26
854.24
904.42
980.36
1032.46
1179.50
1221.40
1239.41
1286.56
1487.62
1638.73
1684.56
1838.75
1901.78
1993.82
2126.92
2140.92
2210.94
2256.92
2283.02
2398.85
2544.95
2807.11
2872.20
3337.50

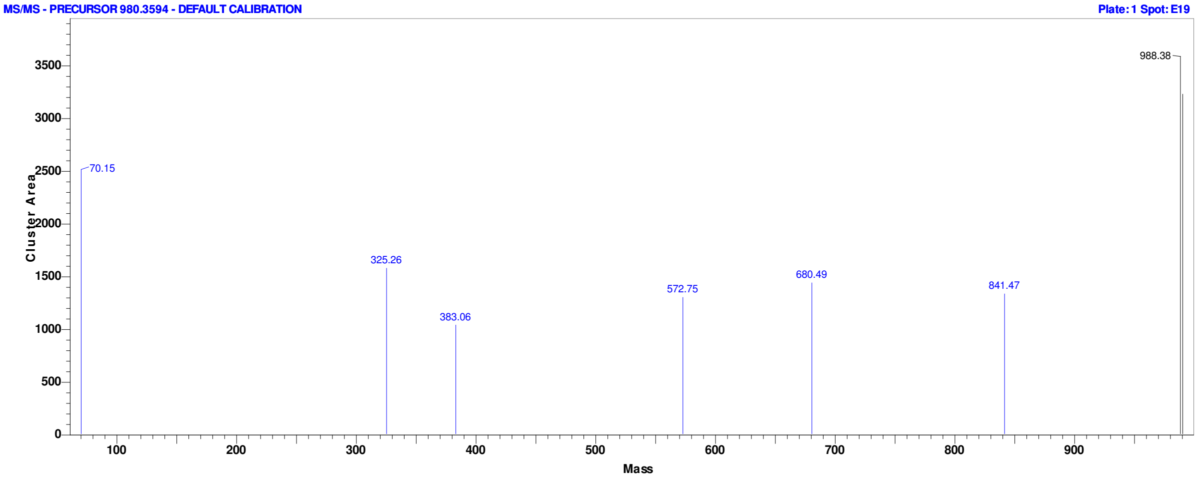

MS/MS - PRECURSOR 980.3594 - DEFAULT CALIBRATION
Plate: 1 Spot: E19
Cluster Area
Mass
70.15
325.26
383.06
572.75
680.49
841.47
988.38

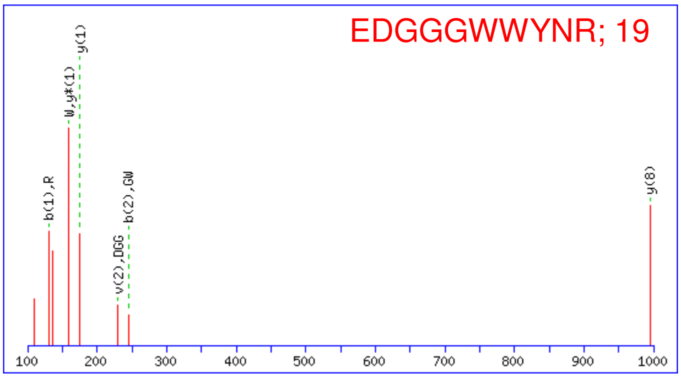

EDGGGWWYNR; 19
b(1),R
W,y*(1)
y(1)
y(2),DGG
b(2),GW
y(8)

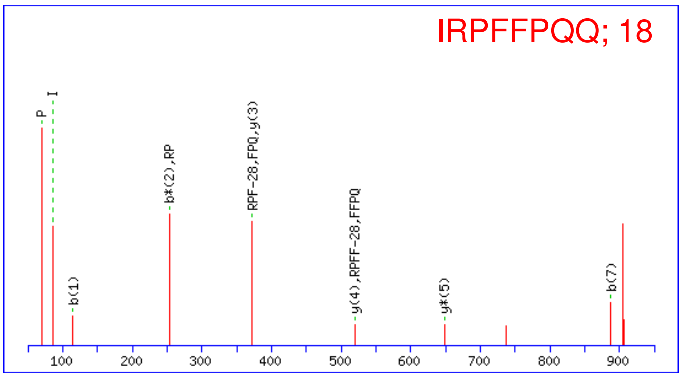

IRPFFPQQ; 18
P
I
b(1)
b*(2),RP
RPF-28,FPQ,y(3)
y(4),RPFF-28,FFPQ
y*(5)
b(7)

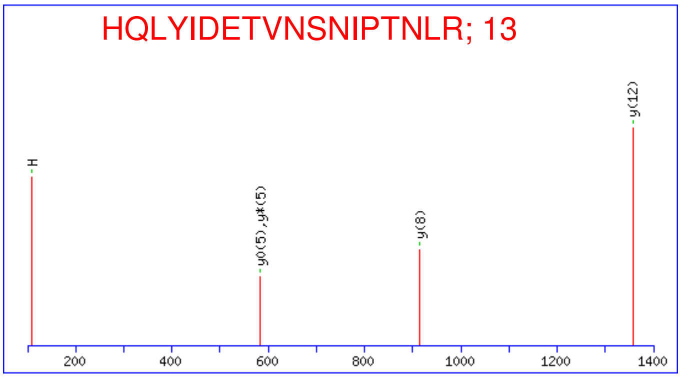

HQLYIDETVNSNIPTNLR; 13
H
y0(5),y*(5)
y(8)
y(12)

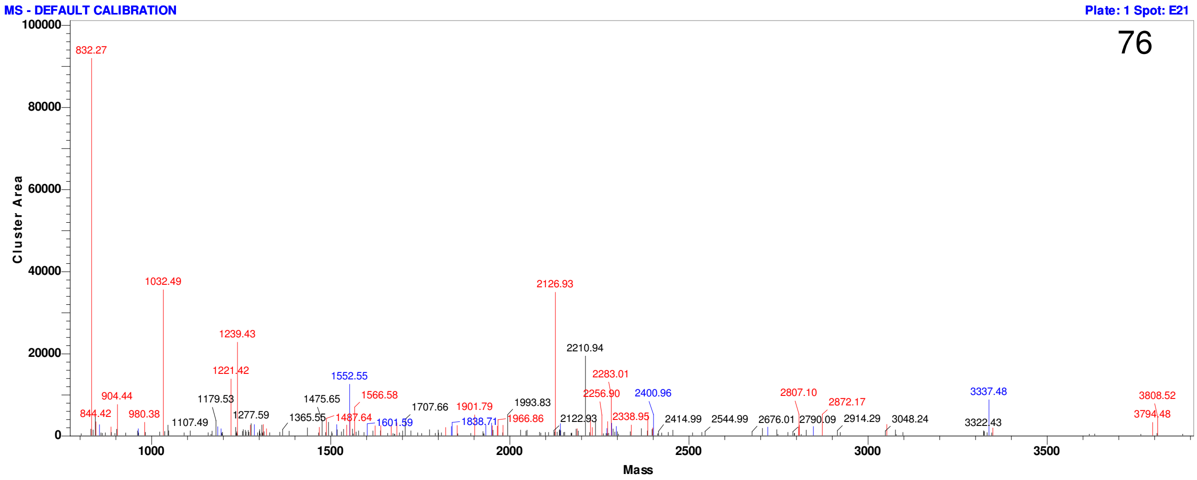
MS - DEFAULT CALIBRATION
Plate: 1 Spot: E21
76
Cluster Area
Mass
832.27
1032.49
1239.43
1221.42
904.44
844.42
980.38
1107.49
1179.53
1277.59
1365.55
1475.65
1487.64
1552.55
1566.58
1601.59
1707.66
1838.71
1901.79
1966.86
1993.83
2122.93
2126.93
2210.94
2256.90
2283.01
2338.95
2400.96
2414.99
2544.99
2676.01
2790.09
2807.10
2872.17
2914.29
3048.24
3322.43
3337.48
3794.48
3808.52

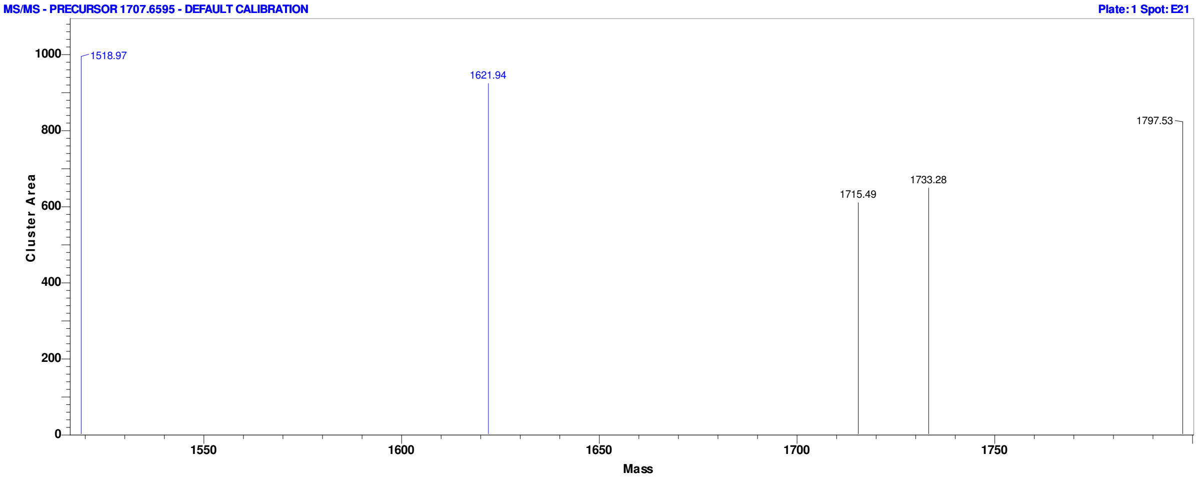
MS/MS - PRECURSOR 1707.6595 - DEFAULT CALIBRATION
Plate: 1 Spot: E21
Cluster Area
Mass
1518.97
1621.94
1715.49
1733.28
1797.53

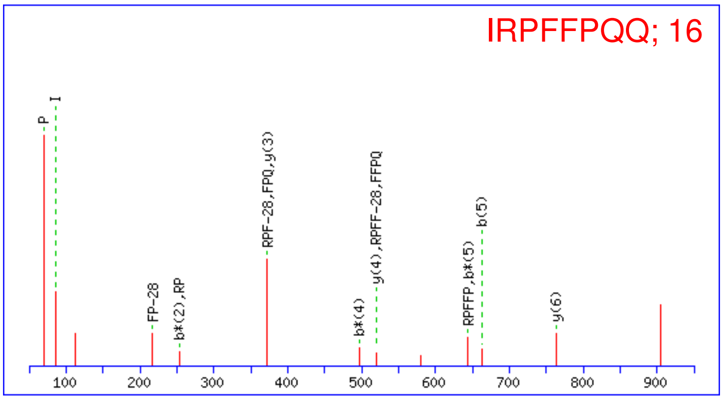
IRPFFFPQQ; 16
P
I
FP-28
b*(2),RP
RPF-28,FPQ,y(3)
b*(4)
y(4),RPFF-28,FFPQ
RPFFP,b*(5)
b(5)
y(6)

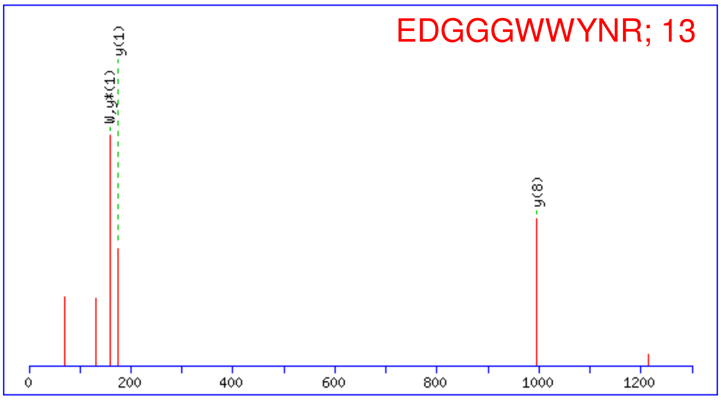
EDGGGWWYNR; 13
W,y*(1)
y(1)
y(8)

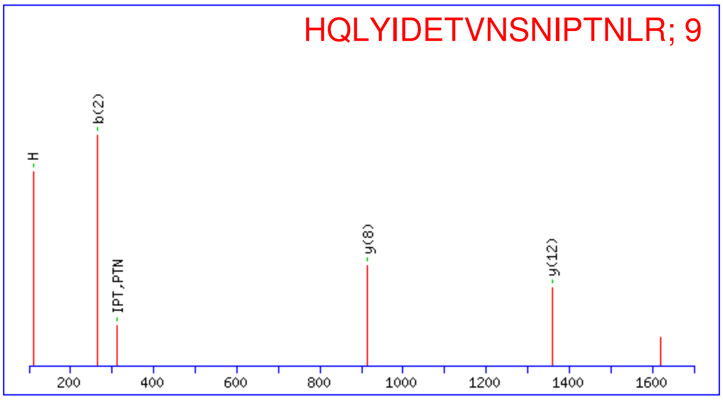
HQLYIDETVNSNIPTNLR; 9
H
b(2)
IPT,PTN
y(8)
y(12)

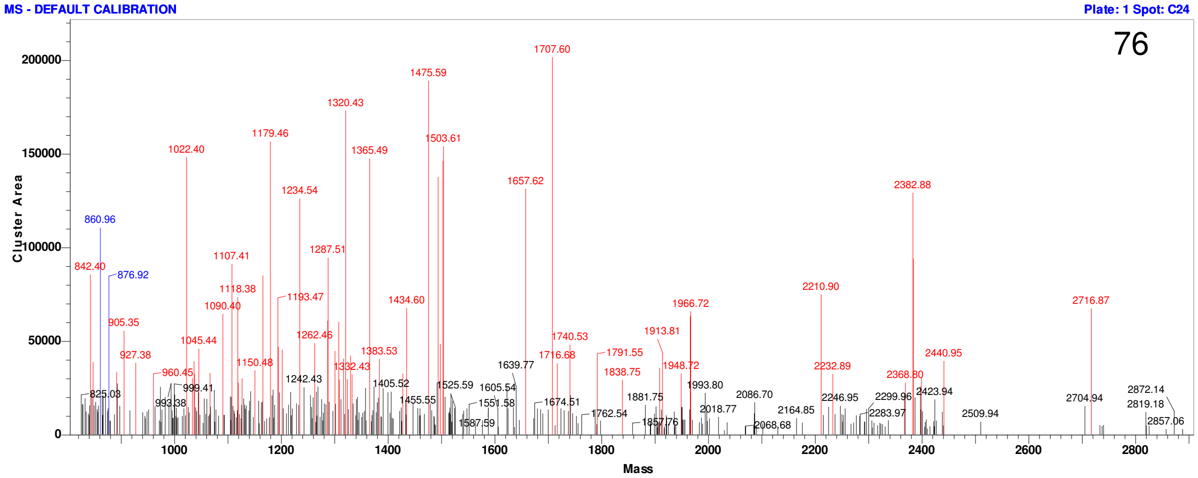
MS - DEFAULT CALIBRATION
Plate: 1 Spot: C24
76
Cluster Area
Mass
1707.60
1475.59
1320.43
1179.46
1503.61
1365.49
1022.40
1657.62
2382.88
1234.54
860.96
1287.51
842.40
876.92
1107.41
1118.38
2210.90
2716.87
1193.47
1434.60
1090.40
1966.72
905.35
1262.46
1740.53
1913.81
1045.44
1383.53
1716.68
1791.55
2440.95
927.38
1150.48
1332.43
1639.77
1948.72
2232.89
960.45
1242.43
1838.75
2368.80
999.41
1405.52
1525.59
1605.54
1993.80
2872.14
825.03
2086.70
2423.94
993.38
1455.55
1551.58
1674.51
1881.75
2246.95
2299.96
2704.94
2819.18
2018.77
2164.85
2283.97
1762.54
2509.94
1587.59
1857.76
2068.68
2857.06

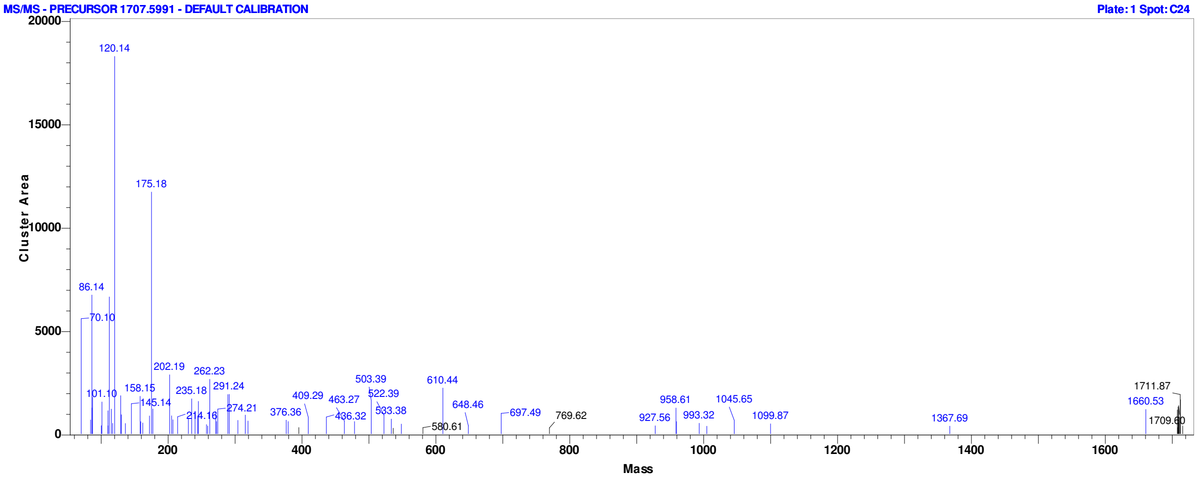
MS/MS - PRECURSOR 1707.5991 - DEFAULT CALIBRATION
Plate: 1 Spot: C24
Cluster Area
Mass
120.14
175.18
86.14
70.10
202.19
262.23
101.10
158.15
235.18
291.24
145.14
214.16
274.21
409.29
503.39
463.27
522.39
376.36
436.32
533.38
610.44
648.46
580.61
697.49
769.62
958.61
1045.65
927.56
993.32
1099.87
1367.69
1711.87
1660.53
1709.60

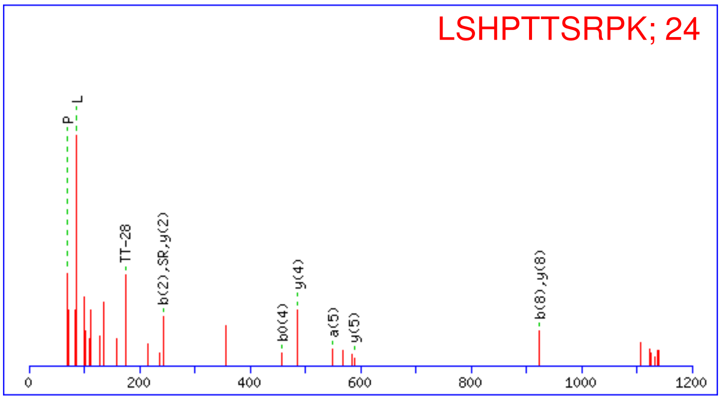
LSHPTTSRPK; 24
P
L
TT-28
b(2),SR,y(2)
b0(4)
y(4)
a(5)
y(5)
b(8),y(8)

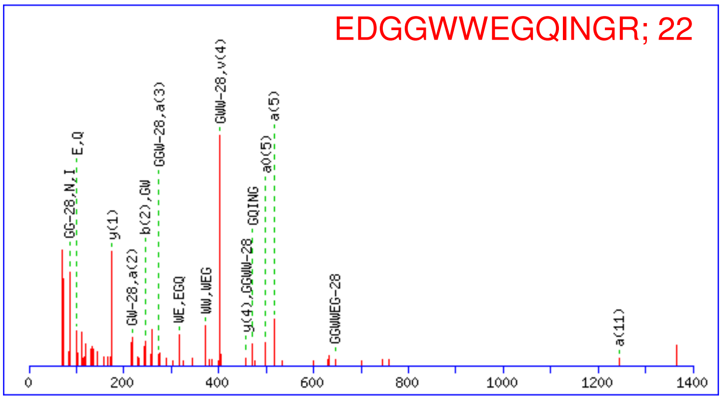
EDGGWWEGQINGR; 22
GG-28,N,I
E,Q
y(1)
GW-28,a(2)
b(2),GW
GGW-28,a(3)
WE,EGQ
WW,WEG
GWW-28,y(4)
y(4),GGWW-28
GQING
a0(5)
a(5)
GGWWEG-28
a(11)

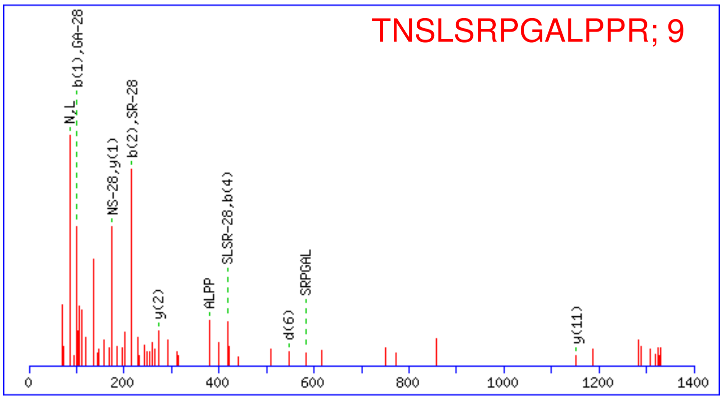
TNSLSRPGALPPR; 9
N,L
b(1),GA-28
NS-28,y(1)
b(2),SR-28
y(2)
ALPP
SLSR-28,b(4)
d(6)
SRPGAL
y(11)

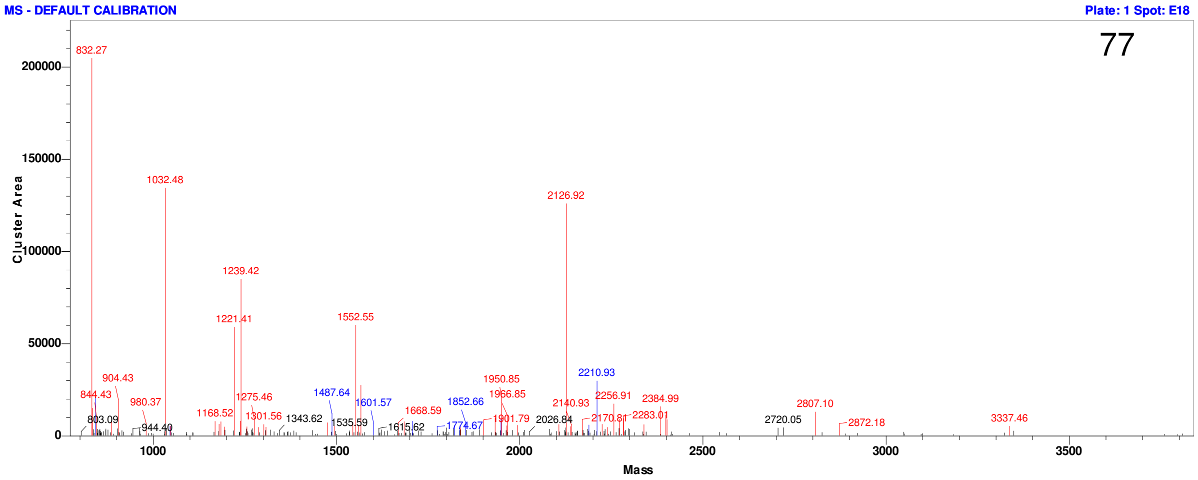
MS - DEFAULT CALIBRATION
Plate: 1 Spot: E18
77
Cluster Area
Mass
832.27
1032.48
2126.92
1239.42
1221.41
1552.55
904.43
844.43
980.37
803.09
944.40
1168.52
1275.46
1301.56
1343.62
1487.64
1535.59
1601.57
1615.62
1668.59
1852.66
1774.67
1950.85
1966.85
1901.79
2026.84
2140.93
2210.93
2256.91
2170.81
2283.01
2384.99
2720.05
2807.10
2872.18
3337.46

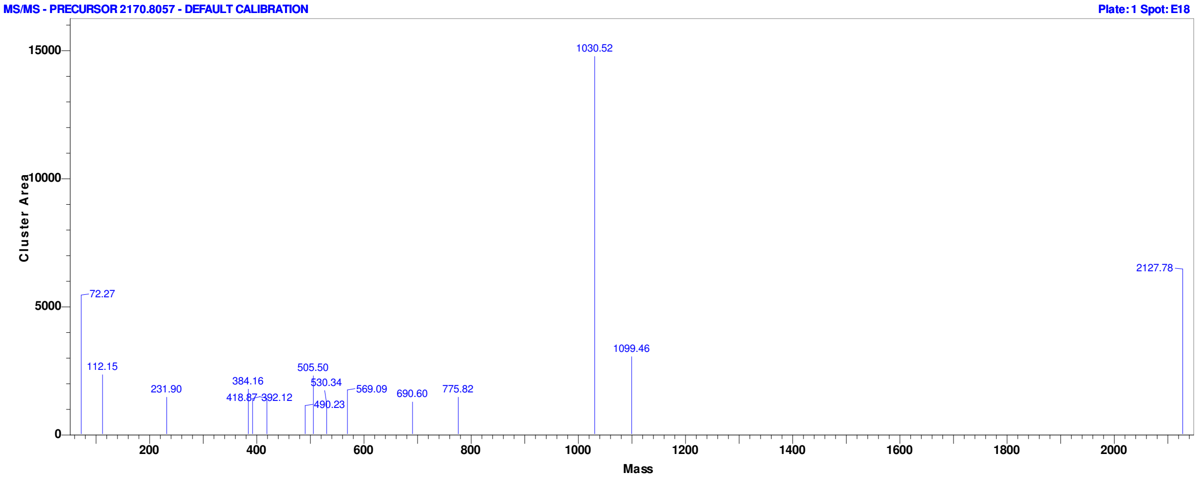
MS/MS - PRECURSOR 2170.8057 - DEFAULT CALIBRATION
Plate: 1 Spot: E18
Cluster Area
Mass
1030.52
2127.78
72.27
112.15
231.90
384.16
418.87
392.12
505.50
530.34
490.23
569.09
690.60
775.82
1099.46

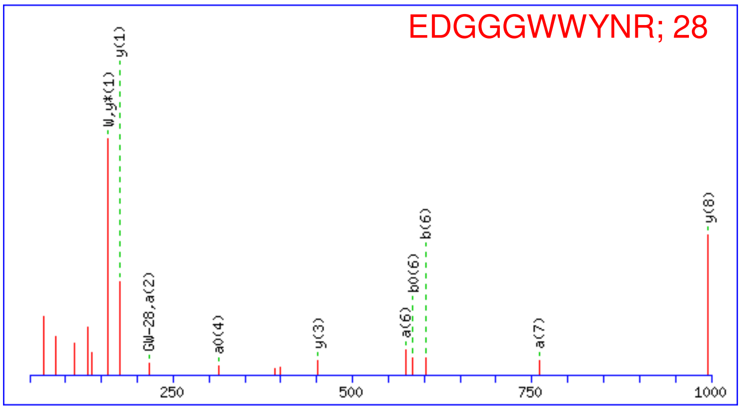
EDGGGWWYNR; 28
W,y*(1)
y(1)
GW-28,a(2)
a0(4)
y(3)
a(6)
b0(6)
b(6)
a(7)
y(8)

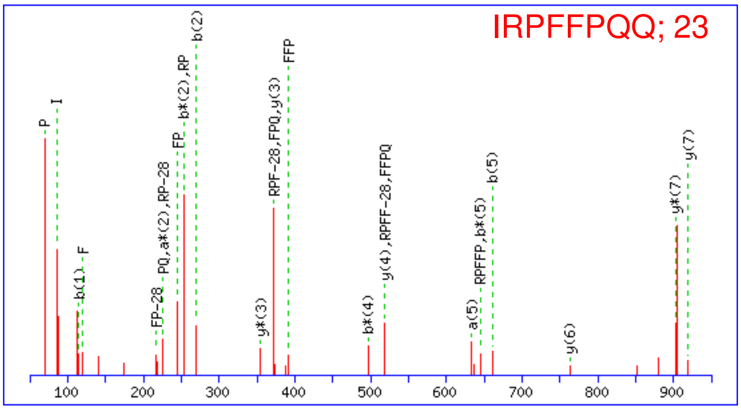
IRPFFPQQ; 23
P
I
b(1)
F
FP-28
PQ,a*(2),RP-28
FP
b*(2),RP
b(2)
y*(3)
RPF-28,FPQ,y(3)
FFP
b*(4)
y(4),RPFF-28,FFPQ
a(5)
RPFFP,b*(5)
b(5)
y(6)
y*(7)
y(7)

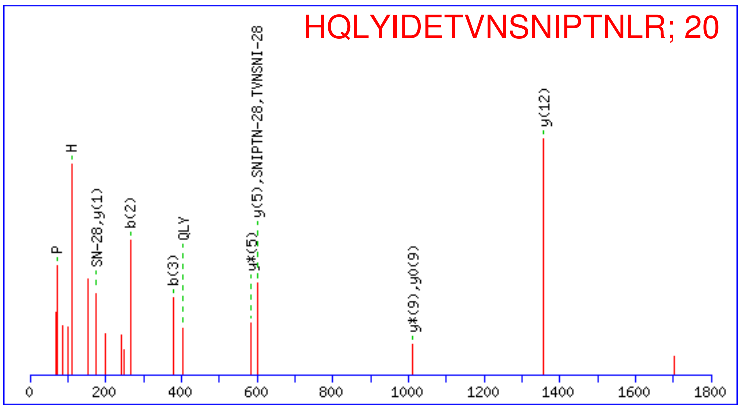
HQLYIDETVNSNIPTNLR; 20
P
H
SN-28,y(1)
b(2)
b(3)
QLY
y*(5)
y(5),SNIPTN-28,TVNSNI-28
y*(9),y0(9)
y(12)

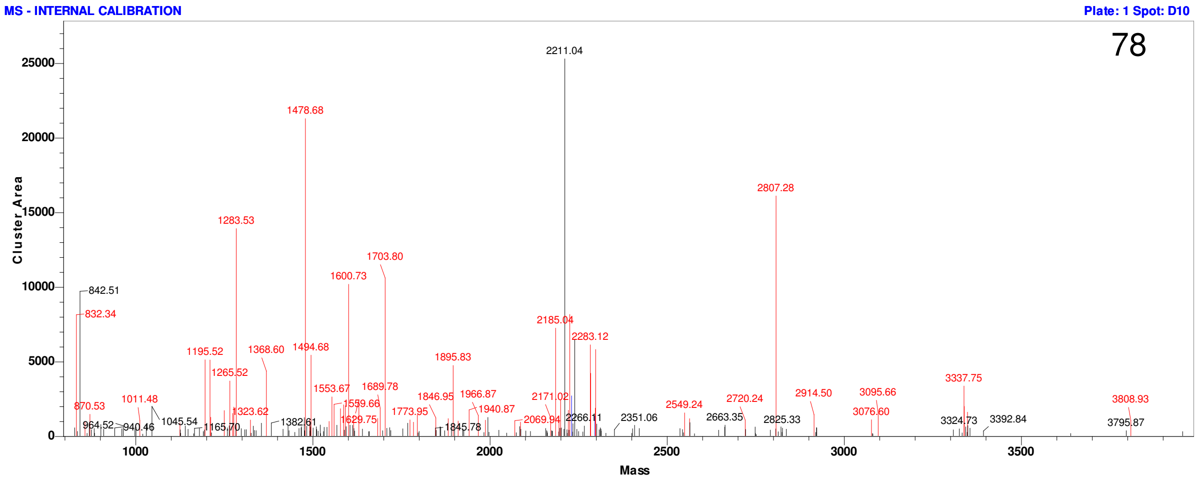

MS - INTERNAL CALIBRATION
Plate: 1 Spot: D10
78
Cluster Area
Mass
2211.04
1478.68
2807.28
1283.53
1703.80
1600.73
842.51
832.34
2185.04
2283.12
1195.52
1368.60
1494.68
1895.83
1265.52
3337.75
1553.67
1689.78
1846.95
1966.87
2171.02
2720.24
2914.50
3095.66
3808.93
1011.48
870.53
1559.66
1323.62
1773.95
1940.87
2549.24
3076.60
1045.54
964.52
940.46
1165.70
1382.61
1629.75
1845.78
2069.94
2266.11
2351.06
2663.35
2825.33
3324.73
3392.84
3795.87

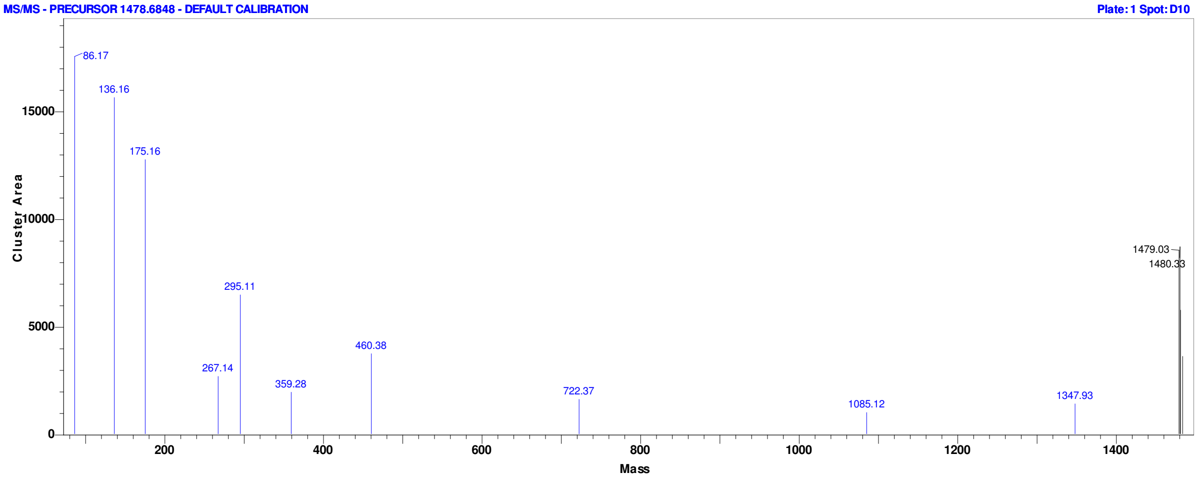

MS/MS - PRECURSOR 1478.6848 - DEFAULT CALIBRATION
Plate: 1 Spot: D10
Cluster Area
Mass
86.17
136.16
175.16
295.11
267.14
359.28
460.38
722.37
1085.12
1347.93
1479.03
1480.33

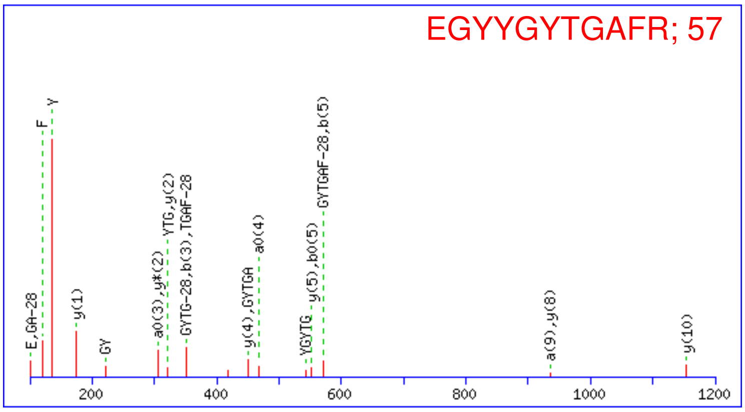

EGYYGYTGAFR; 57
E,GA-28
F
Y
y(1)
GY
a0(3),y*(2)
YTG,y(2)
GYTG-28,b(3),TGAF-28
y(4),GYTGA
a0(4)
YGYTG
y(5),b0(5)
GYTGAF-28,b(5)
a(9),y(8)
y(10)

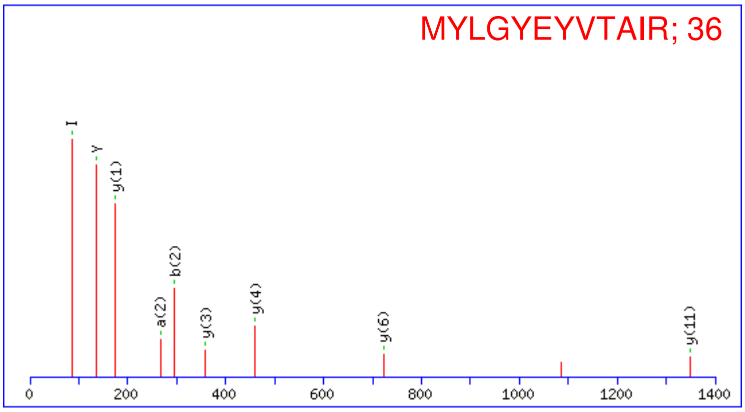

MYLGYEYVTAIR; 36
I
Y
y(1)
a(2)
b(2)
y(3)
y(4)
y(6)
y(11)

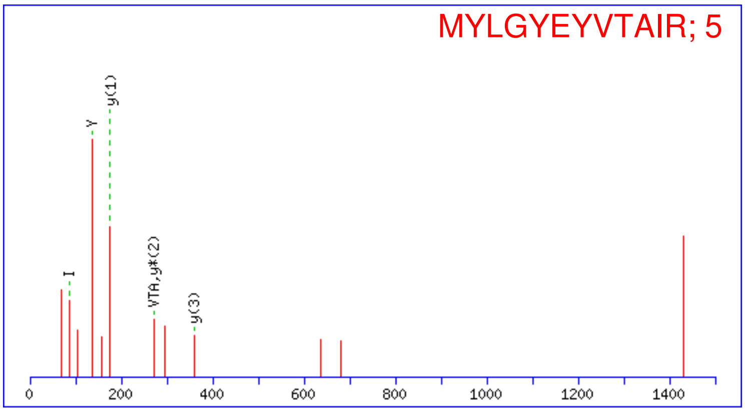

MYLGYEYVTAIR; 5
I
Y
y(1)
VTA,y*(2)
y(3)

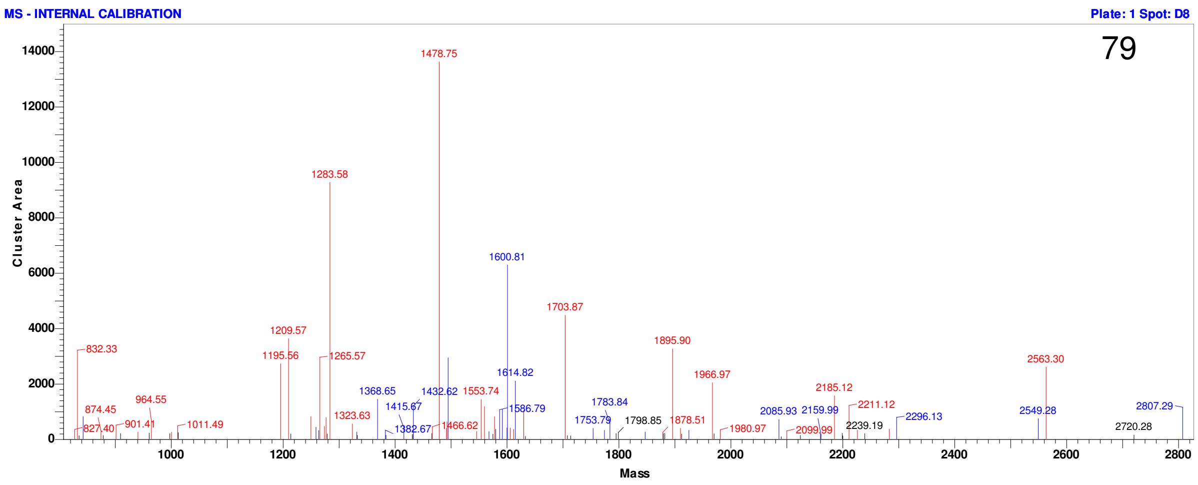
MS - INTERNAL CALIBRATION
Plate: 1 Spot: D8
79
Cluster Area
Mass
1478.75
1283.58
1600.81
1703.87
1209.57
1195.56
1265.57
832.33
1895.90
2563.30
1966.97
1614.82
1368.65
1432.62
1553.74
2185.12
964.55
1415.67
1586.79
1783.84
2211.12
874.45
1323.63
2085.93
2159.99
2549.28
2807.29
1753.79
1798.85
1878.51
2296.13
827.40
901.41
1011.49
1382.67
1466.62
1980.97
2099.99
2239.19
2720.28

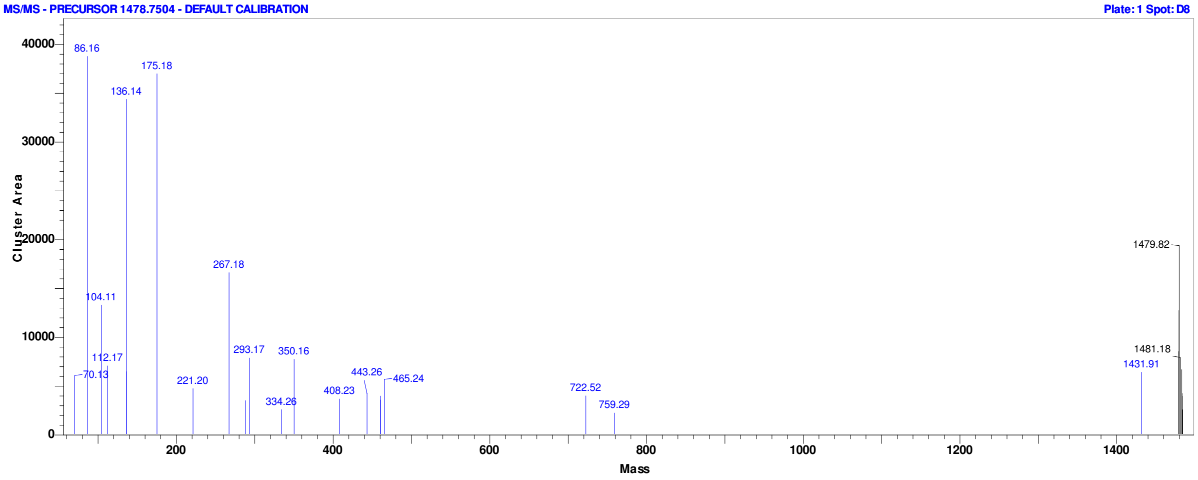
MS/MS - PRECURSOR 1478.7504 - DEFAULT CALIBRATION
Plate: 1 Spot: D8
Cluster Area
Mass
86.16
175.18
136.14
1479.82
267.18
104.11
112.17
70.13
221.20
293.17
350.16
334.26
443.26
465.24
408.23
722.52
759.29
1481.18
1431.91

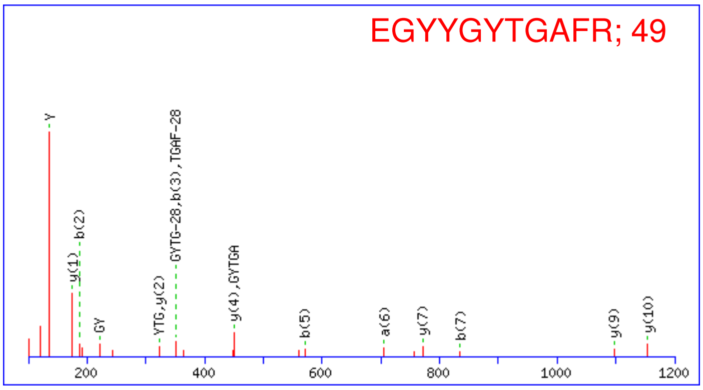
EGYYGYTGAFR; 49
Y
y(1)
b(2)
GY
YTG,y(2)
GYTG-28,b(3),TGAF-28
y(4),GYTGA
b(5)
a(6)
y(7)
b(7)
y(9)
y(10)

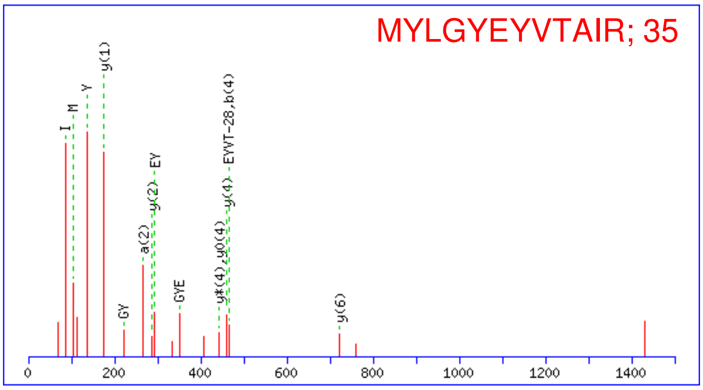
MYLGYEYVTAIR; 35
I
M
Y
y(1)
GY
a(2)
y(2)
EY
GYE
y*(4),y0(4)
y(4)
EYVT-28,b(4)
y(6)

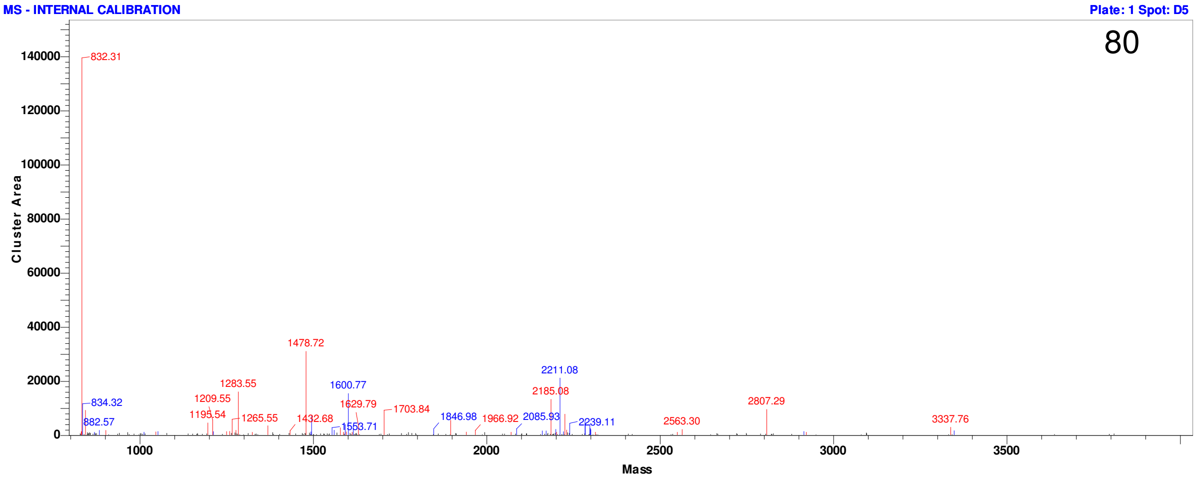
MS - INTERNAL CALIBRATION
Plate: 1 Spot: D5
80
Cluster Area
Mass
832.31
1478.72
1283.55
1209.55
1195.54
1265.55
1432.68
1600.77
1629.79
1553.71
1703.84
1846.98
1966.92
2085.93
2211.08
2185.08
2239.11
2563.30
2807.29
3337.76
834.32
882.57

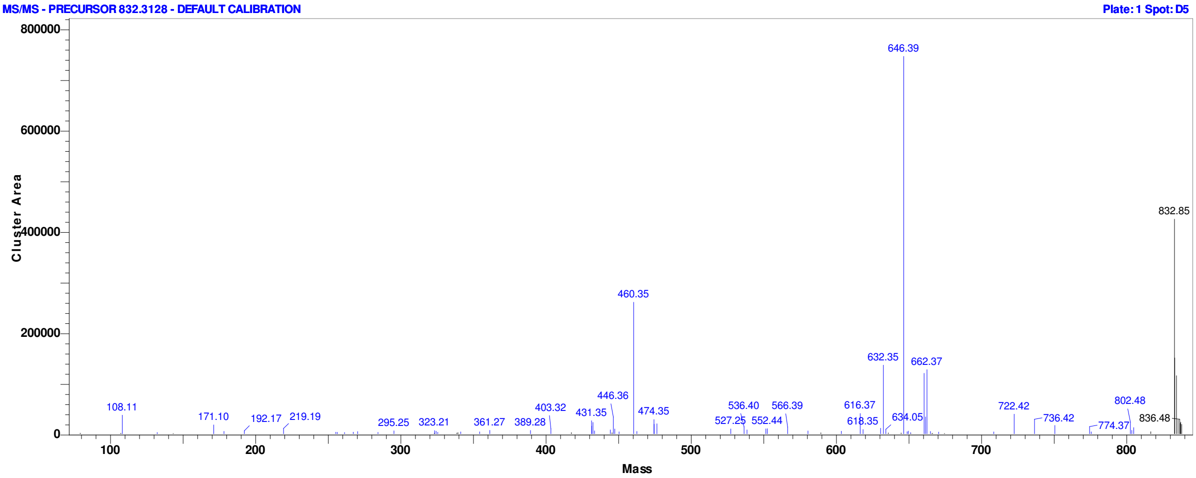
MS/MS - PRECURSOR 832.3128 - DEFAULT CALIBRATION
Plate: 1 Spot: D5
Cluster Area
Mass
646.39
832.85
460.35
632.35
662.37
446.36
474.35
108.11
171.10
192.17
219.19
295.25
323.21
361.27
389.28
403.32
431.35
527.25
536.40
552.44
566.39
616.37
618.35
634.05
722.42
736.42
774.37
802.48
836.48

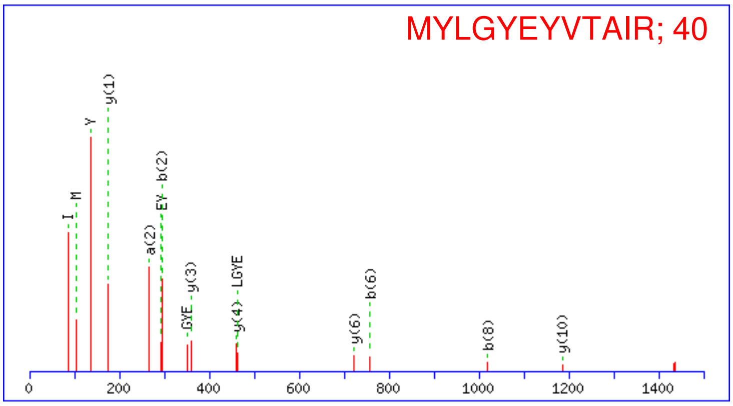
MYLGYEYVTAIR; 40

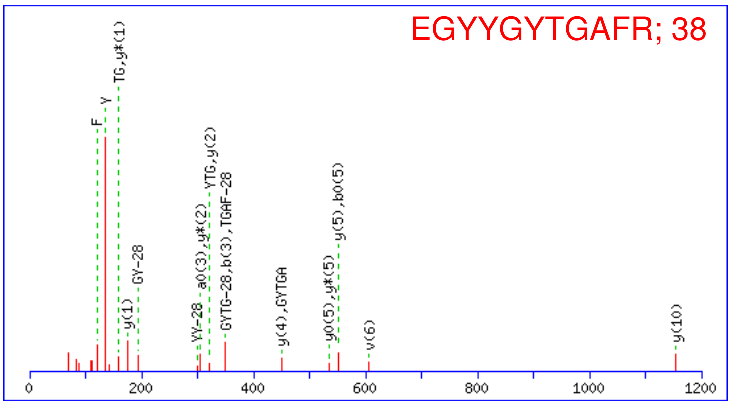
EGYYGYTGAFR; 38

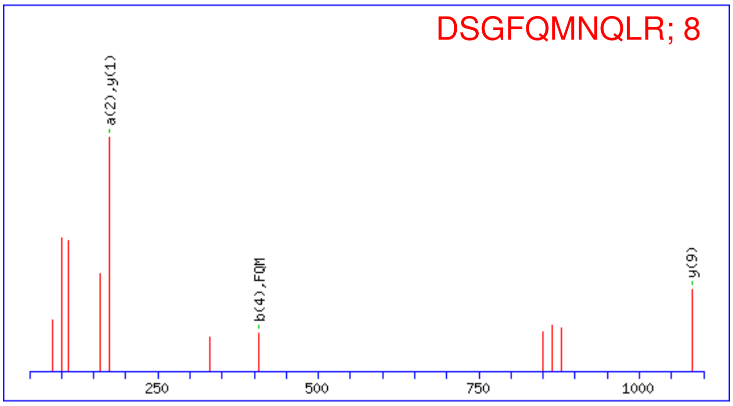
DSGFQMNQLR; 8

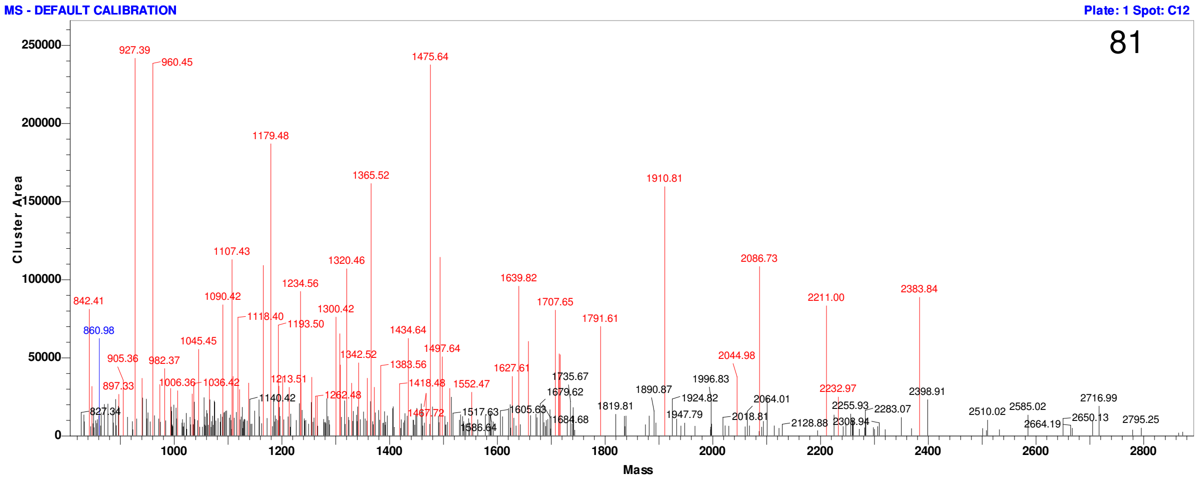
MS - DEFAULT CALIBRATION
Plate: 1 Spot: C12
81
Cluster Area
Mass
927.39
960.45
1475.64
1179.48
1365.52
1910.81
1107.43
1320.46
2086.73
1639.82
1234.56
2383.84
842.41
1090.42
2211.00
1707.65
1300.42
1118.40
1193.50
1791.61
860.98
1045.45
1434.64
1497.64
2044.98
905.36
982.37
1342.52
1383.56
1627.61
1735.67
897.33
1006.36
1036.42
1213.51
1418.48
1552.47
2232.97
1890.87
1996.83
2398.91
1679.62
1262.48
1140.42
1924.82
2064.01
2716.99
827.34
1605.63
1517.63
1487.72
2255.93
2283.07
2510.02
2585.02
1947.79
2018.81
1819.81
1684.68
2128.88
2308.94
2650.13
2795.25
1586.64
2664.19

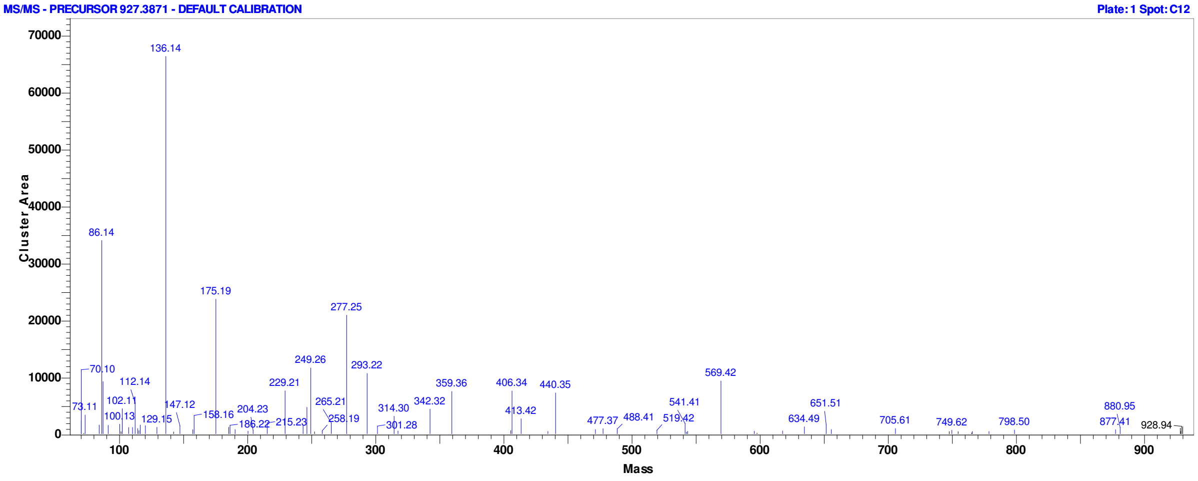
MS/MS - PRECURSOR 927.3871 - DEFAULT CALIBRATION
Plate: 1 Spot: C12
Cluster Area
Mass
136.14
86.14
175.19
277.25
249.26
293.22
229.21
70.10
112.14
569.42
359.36
406.34
440.35
73.11
102.11
147.12
265.21
314.30
342.32
651.51
880.95
541.41
100.13
129.15
158.16
204.23
258.19
413.42
186.22
215.23
301.28
477.37
488.41
519.42
634.49
705.61
749.62
798.50
877.41
928.94

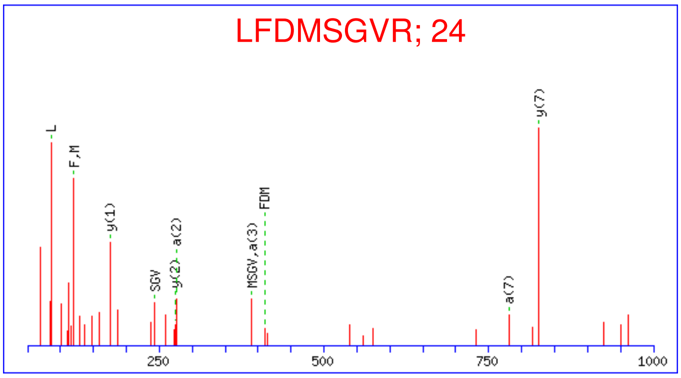
LFDMSGVR; 24
L
F,M
y(1)
SGV
y(2)
a(2)
MSGV,a(3)
FDM
a(7)
y(7)

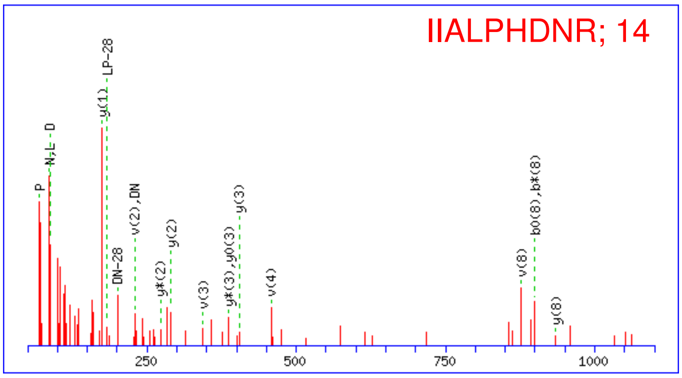
IIALPHDNR; 14
P
N,L
D
y(1)
LP-28
DN-28
y(2),DN
y*(2)
y(2)
y(3)
y*(3),y0(3)
y(3)
y(4)
y(8)
b0(8),b*(8)
y(8)

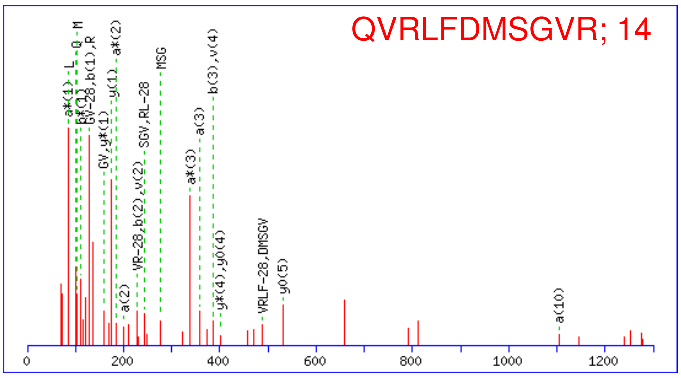
QVRLFDMSGVR; 14
a*(1)
a(1)
L
Q
M
GV-28,b(1),R
GV,y*(1)
y(1)
a*(2)
a(2)
VR-28,b(2),y(2)
SGV,RL-28
MSG
a*(3)
a(3)
b(3),y(4)
y*(4),y0(4)
VRLF-28,DMSGV
y0(5)
a(10)

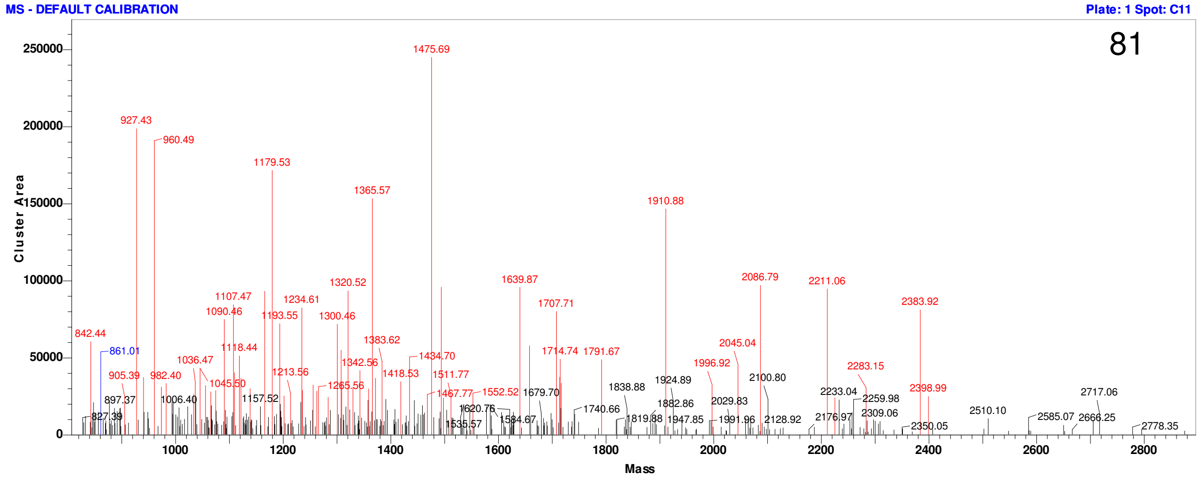
MS - DEFAULT CALIBRATION
Plate: 1 Spot: C11
81
Cluster Area
Mass

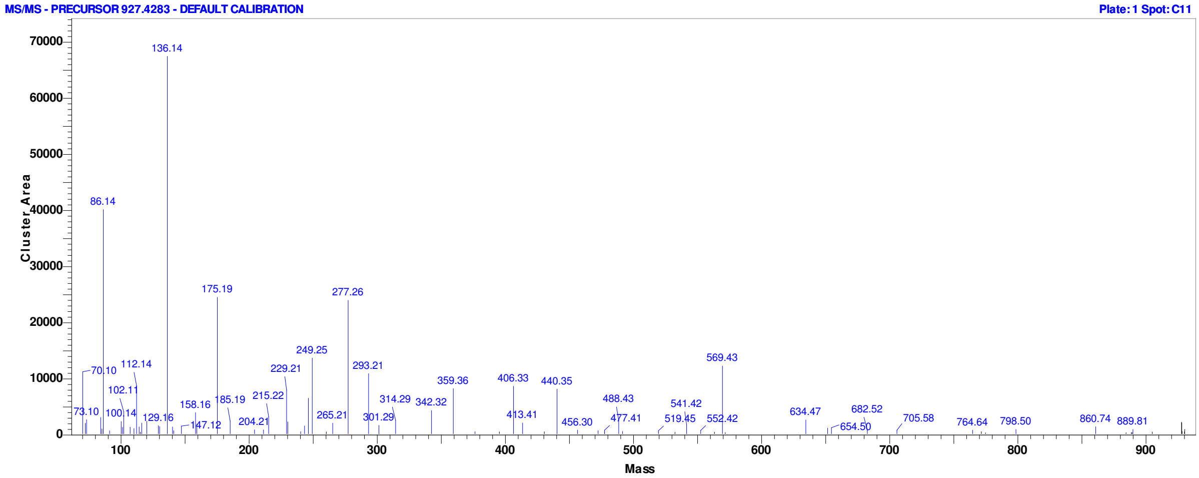
MS/MS - PRECURSOR 927.4283 - DEFAULT CALIBRATION
Plate: 1 Spot: C11
Cluster Area
Mass

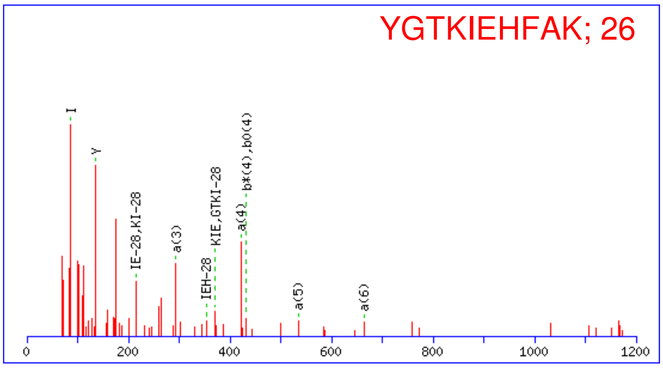
YGTKIEHFAK; 26

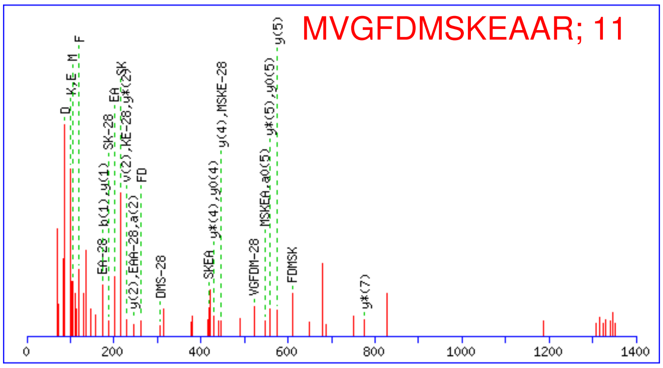
MVGFDMSKEAAR; 11

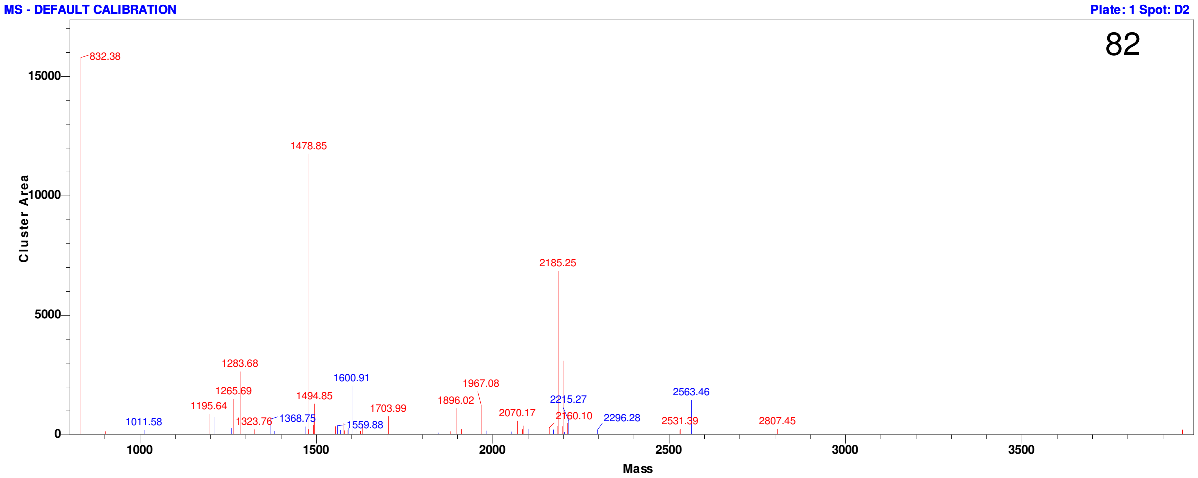
MS - DEFAULT CALIBRATION
Plate: 1 Spot: D2
82
Cluster Area
Mass
832.38
1478.85
2185.25
1283.68
1265.69
1195.64
1600.91
1494.85
1703.99
1967.08
1896.02
2070.17
2215.27
2160.10
2296.28
2563.46
2531.39
2807.45
1011.58
1323.76
1368.75
1559.88

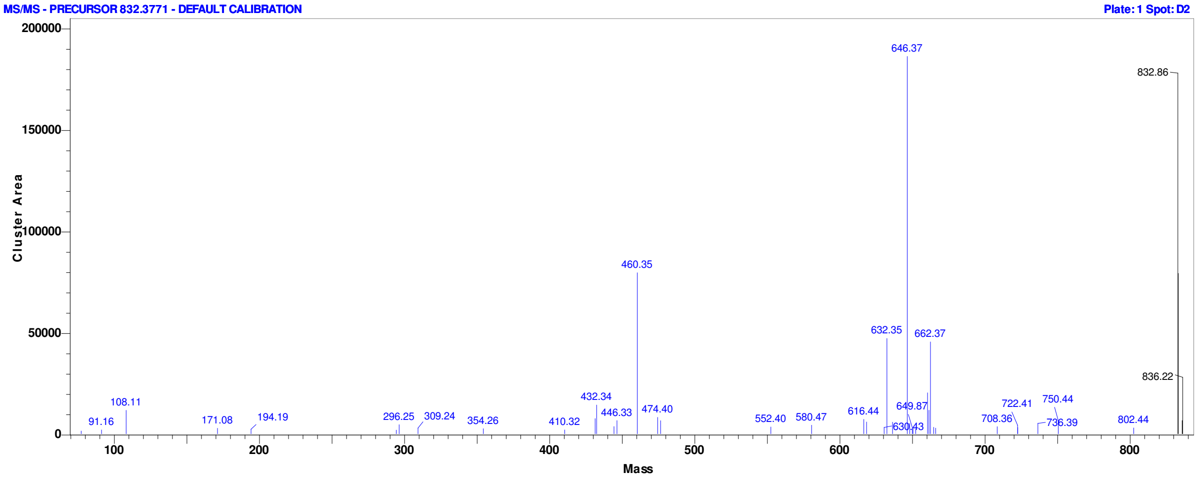
MS/MS - PRECURSOR 832.3771 - DEFAULT CALIBRATION
Plate: 1 Spot: D2
Cluster Area
Mass
646.37
832.86
460.35
632.35
662.37
836.22
108.11
91.16
171.08
194.19
296.25
309.24
354.26
410.32
432.34
446.33
474.40
552.40
580.47
616.44
649.87
630.43
708.36
722.41
750.44
736.39
802.44

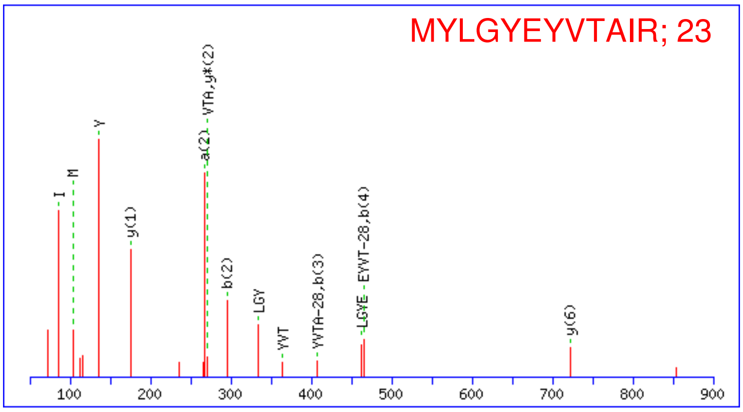
MYLGYEYVTAIR; 23
I
M
Y
y(1)
a(2)
VTA,y*(2)
b(2)
LGY
YVT
YVTA-28,b(3)
LGYE
EYVT-28,b(4)
y(6)

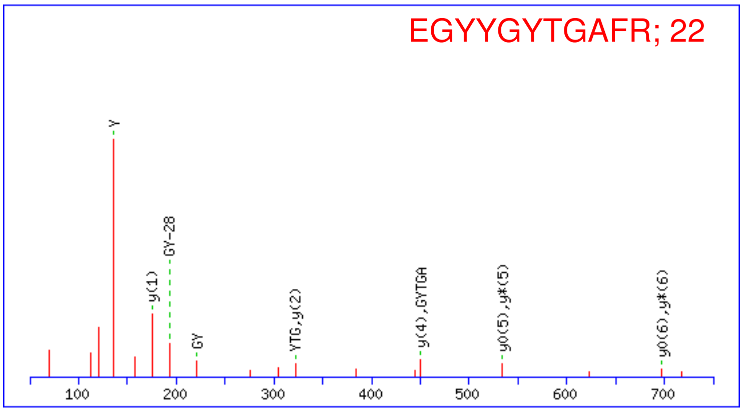
EGYYGYTGAFR; 22
Y
y(1)
GY-28
GY
YTG,y(2)
y(4),GYTGA
y0(5),y*(5)
y0(6),y*(6)

83

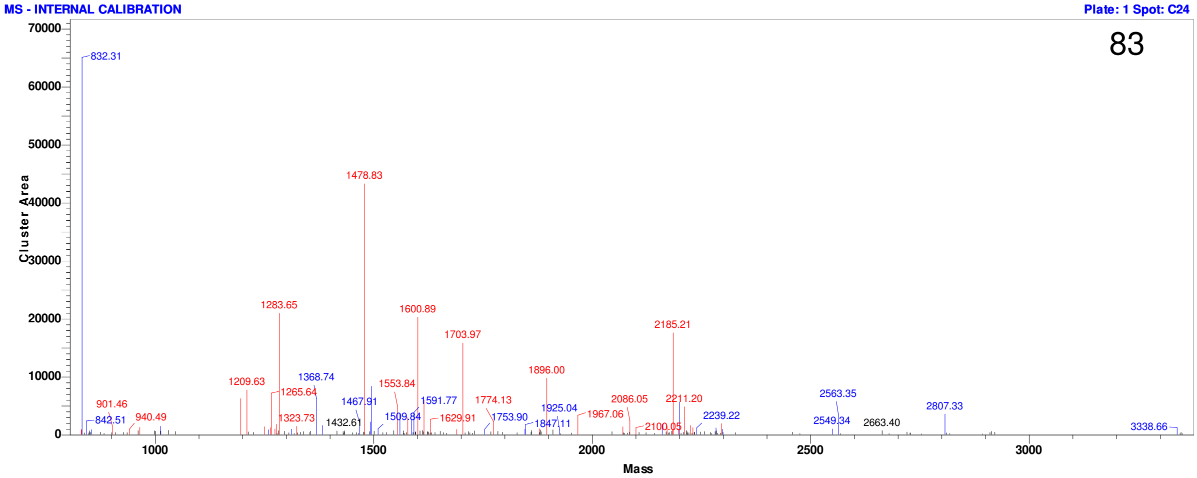
MS - INTERNAL CALIBRATION
Plate: 1 Spot: C24
Cluster Area
Mass
70000
60000
50000
40000
30000
20000
10000
0
1000
1500
2000
2500
3000
832.31
842.51
901.46
940.49
1209.63
1265.64
1283.65
1323.73
1368.74
1432.61
1467.91
1478.83
1509.84
1553.84
1591.77
1600.89
1629.91
1703.97
1753.90
1774.13
1847.11
1896.00
1925.04
1967.06
2086.05
2100.05
2185.21
2211.20
2239.22
2549.34
2563.35
2663.40
2807.33
3338.66

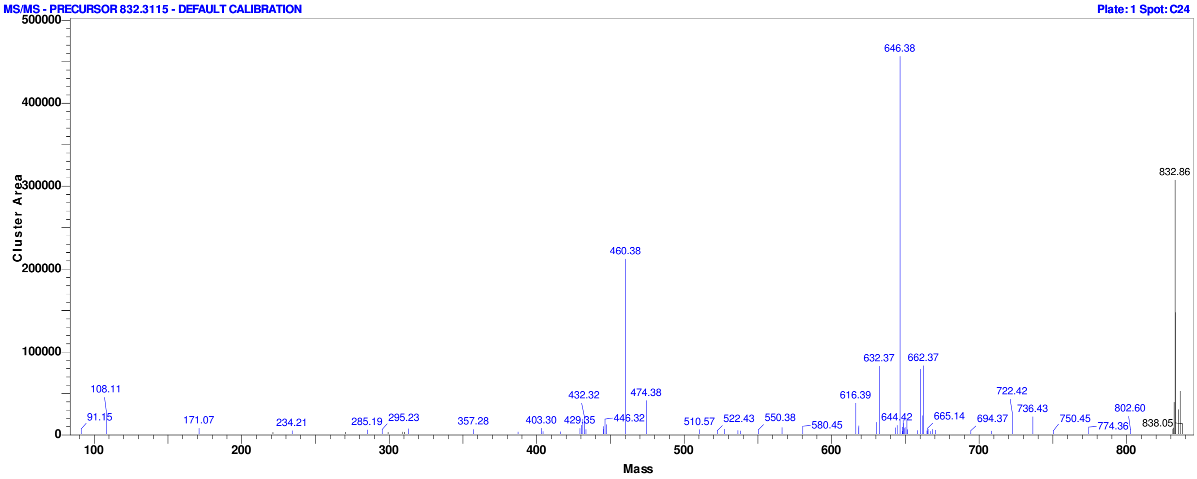
MS/MS - PRECURSOR 832.3115 - DEFAULT CALIBRATION
Plate: 1 Spot: C24
Cluster Area
Mass
500000
400000
300000
200000
100000
0
100
200
300
400
500
600
700
800
91.15
108.11
171.07
234.21
285.19
295.23
357.28
403.30
429.35
432.32
446.32
460.38
474.38
510.57
522.43
550.38
580.45
616.39
632.37
644.42
646.38
662.37
665.14
694.37
722.42
736.43
750.45
774.36
802.60
832.86
838.05

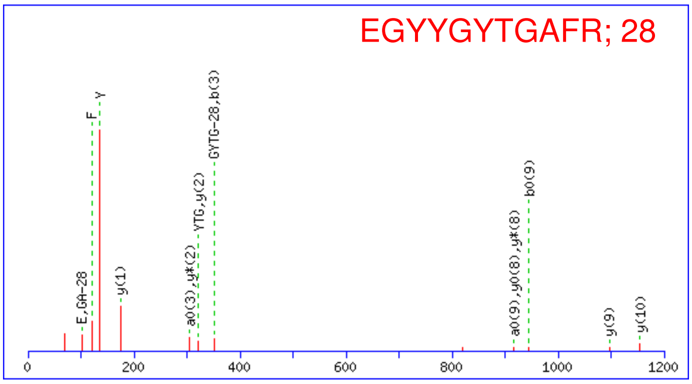
EGYYGYTGAFR; 28
E,GA-28
F
Y
y(1)
a0(3),y*(2)
YTG,y(2)
GYTG-28,b(3)
a0(9),y0(8),y*(8)
b0(9)
y(9)
y(10)
0
200
400
600
800
1000
1200

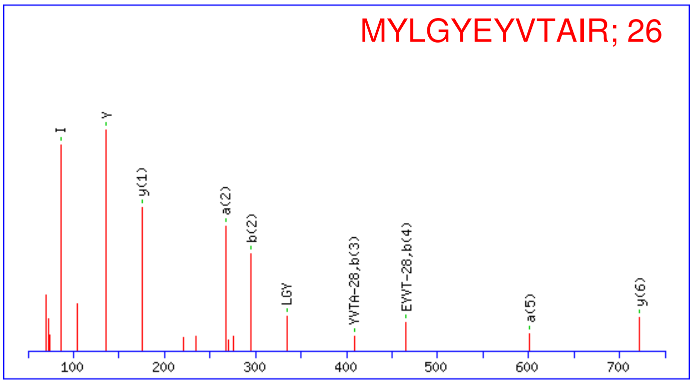
MYLGYEYVTAIR; 26
I
Y
y(1)
a(2)
b(2)
LGY
YVTA-28,b(3)
EYVT-28,b(4)
a(5)
y(6)
100
200
300
400
500
600
700

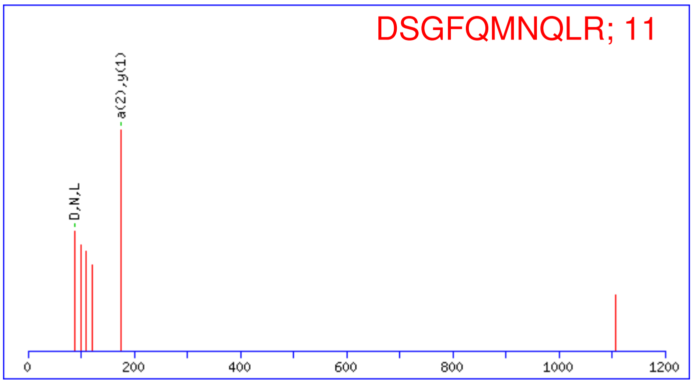
DSGFQMNQLR; 11
D,N,L
a(2),y(1)
0
200
400
600
800
1000
1200

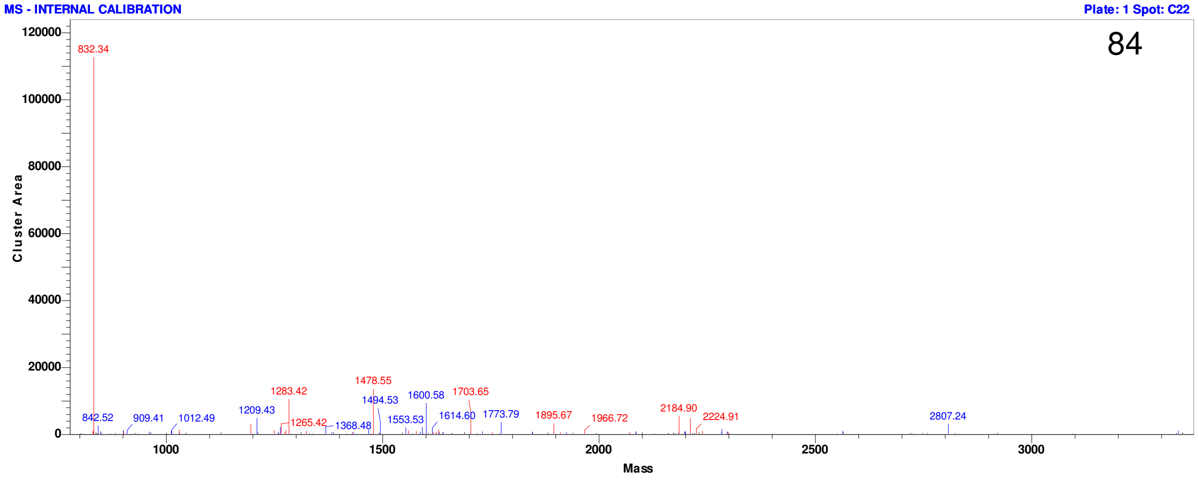
MS - INTERNAL CALIBRATION
Plate: 1 Spot: C22
84
Cluster Area
Mass
832.34
842.52
909.41
1012.49
1209.43
1283.42
1265.42
1368.48
1478.55
1494.53
1553.53
1600.58
1614.60
1703.65
1773.79
1895.67
1966.72
2184.90
2224.91
2807.24

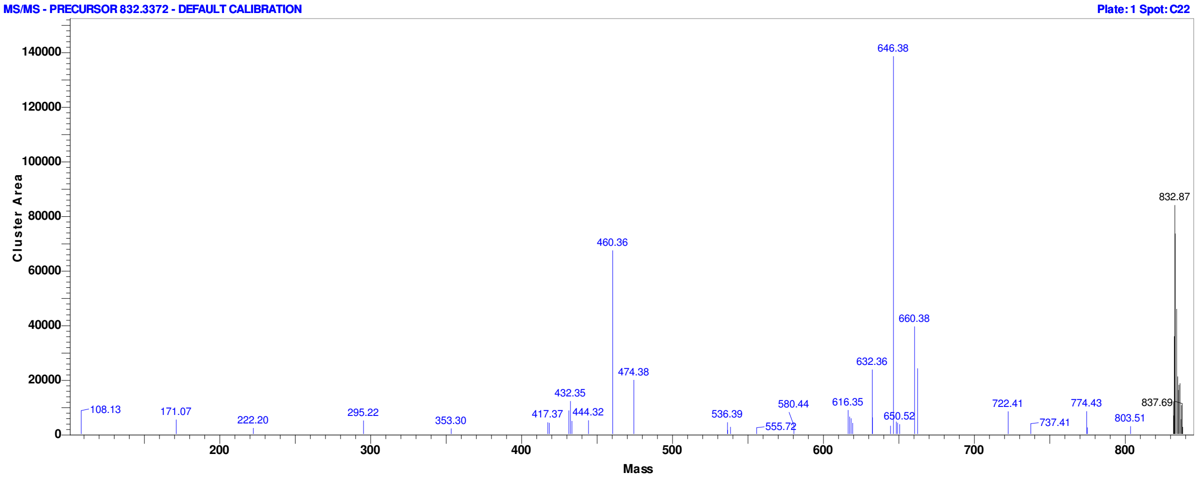
MS/MS - PRECURSOR 832.3372 - DEFAULT CALIBRATION
Plate: 1 Spot: C22
Cluster Area
Mass
646.38
832.87
460.36
660.38
632.36
474.38
432.35
108.13
171.07
222.20
295.22
353.30
417.37
444.32
536.39
555.72
580.44
616.35
650.52
722.41
737.41
774.43
803.51
837.69

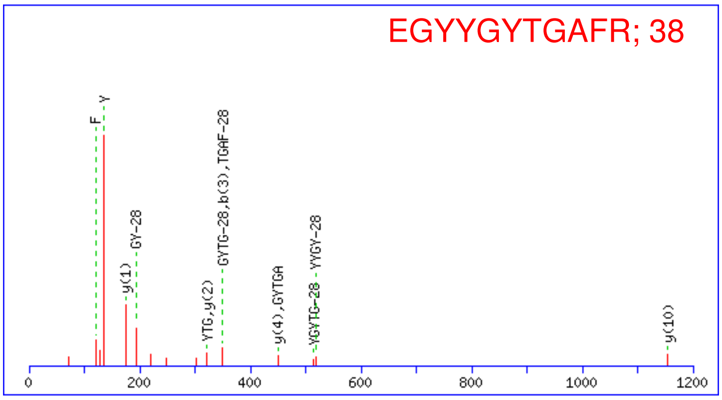
EGYYGYTGAFR; 38
F
Y
GY-28
y(1)
YTG,y(2)
GYTG-28,b(3),TGAF-28
y(4),GYTGA
YYGY-28
YGYTG-28
y(10)

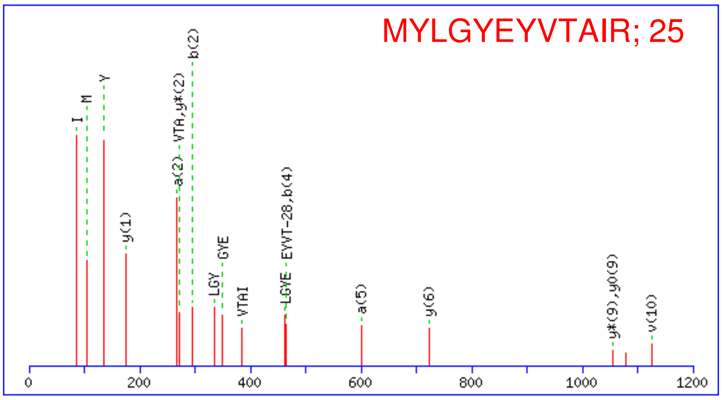
MYLGYEYVTAIR; 25
I
M
Y
y(1)
a(2)
VTA,y*(2)
b(2)
LGY
GYE
VTAI
LGYE
EYVT-28,b(4)
a(5)
y(6)
y*(9),y0(9)
y(10)

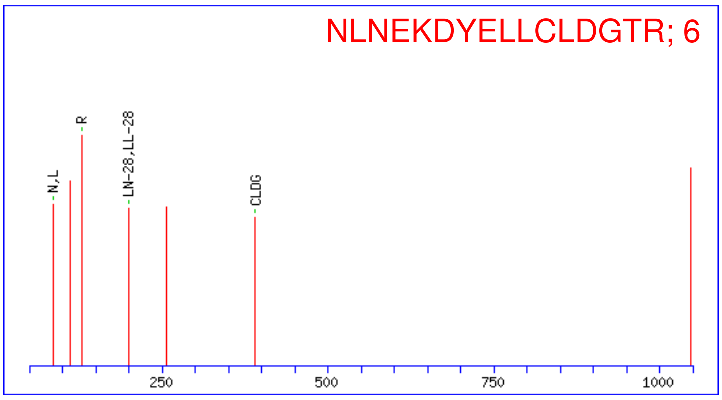
NLNEKDYELLCLDGTR; 6
N,L
R
LN-28,LL-28
CLDG

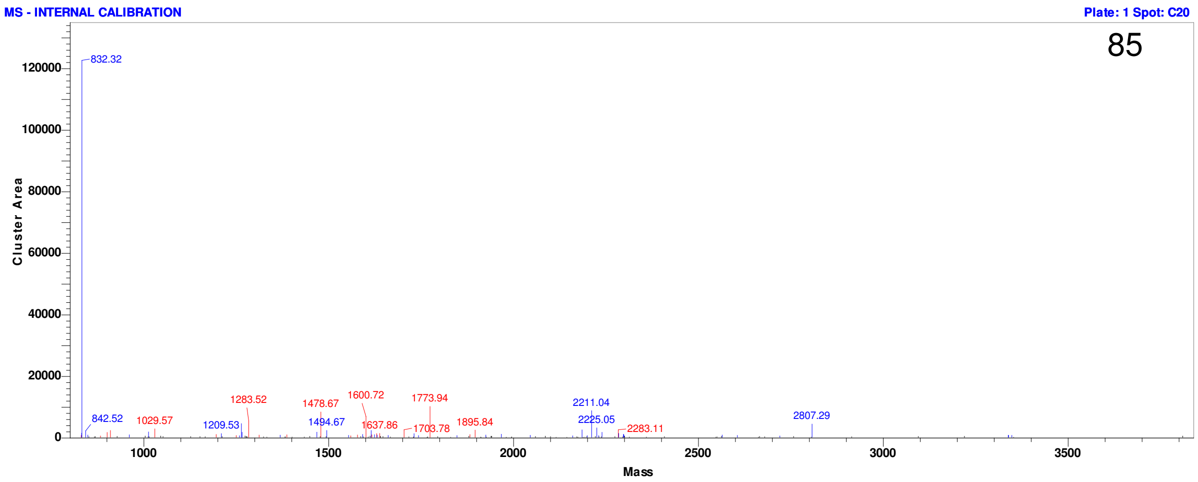

MS - INTERNAL CALIBRATION
Plate: 1 Spot: C20
85
Cluster Area
Mass
832.32
842.52
1029.57
1209.53
1283.52
1478.67
1494.67
1600.72
1637.86
1703.78
1773.94
1895.84
2211.04
2225.05
2283.11
2807.29

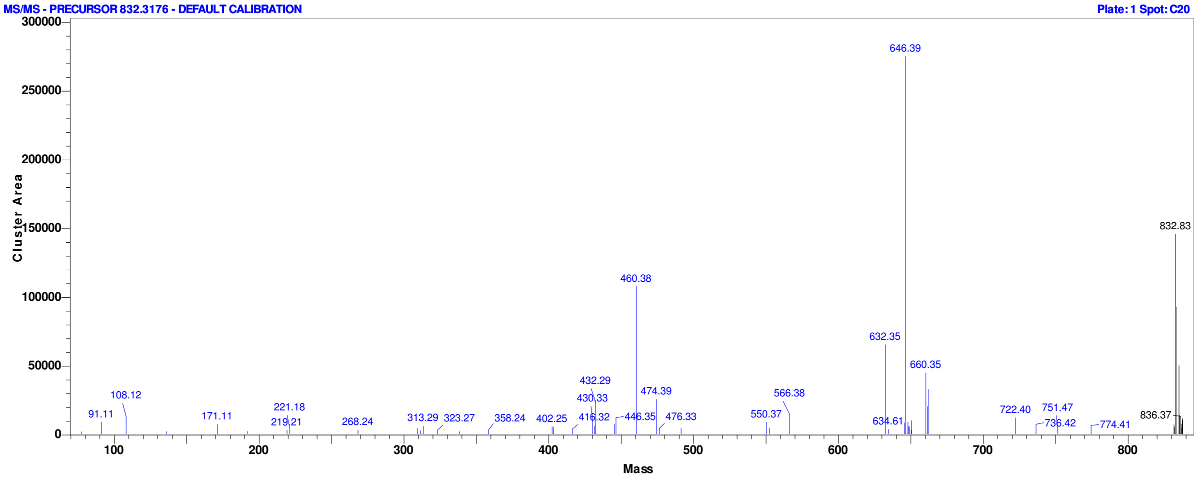

MS/MS - PRECURSOR 832.3176 - DEFAULT CALIBRATION
Plate: 1 Spot: C20
Cluster Area
Mass
646.39
832.83
460.38
632.35
660.35
432.29
474.39
566.38
108.12
91.11
171.11
221.18
219.21
268.24
313.29
323.27
358.24
402.25
430.33
416.32
446.35
476.33
550.37
634.61
722.40
751.47
736.42
774.41
836.37

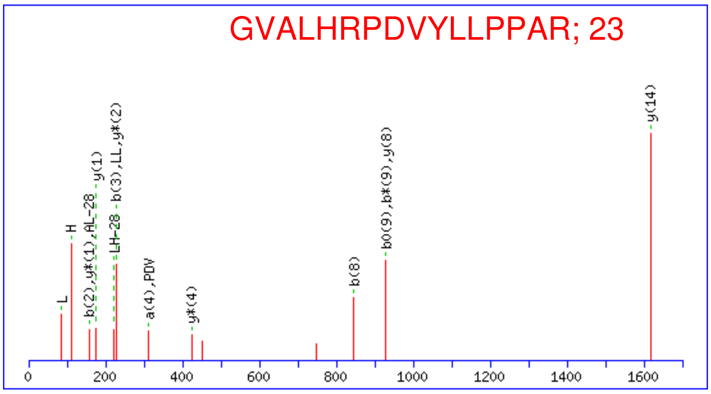

GVALHRPDVYLLPPAR; 23
L
H
b(2),y*(1),AL-28
y(1)
LH-28
b(3),LL,y*(2)
a(4),PDV
y*(4)
b(8)
b0(9),b*(9),y(8)
y(14)

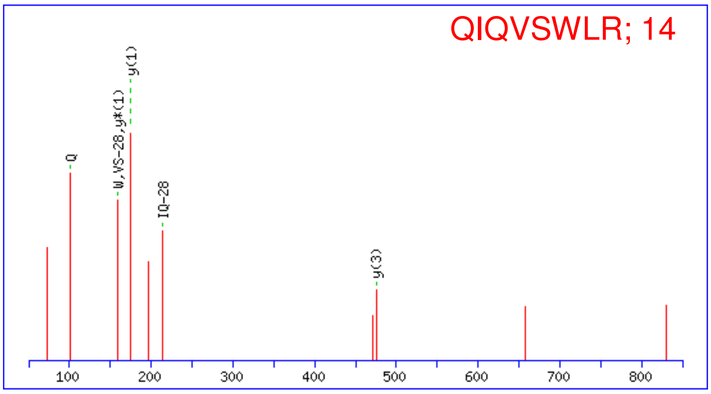

QIQVSWLR; 14
Q
W,VS-28,y*(1)
y(1)
IQ-28
y(3)

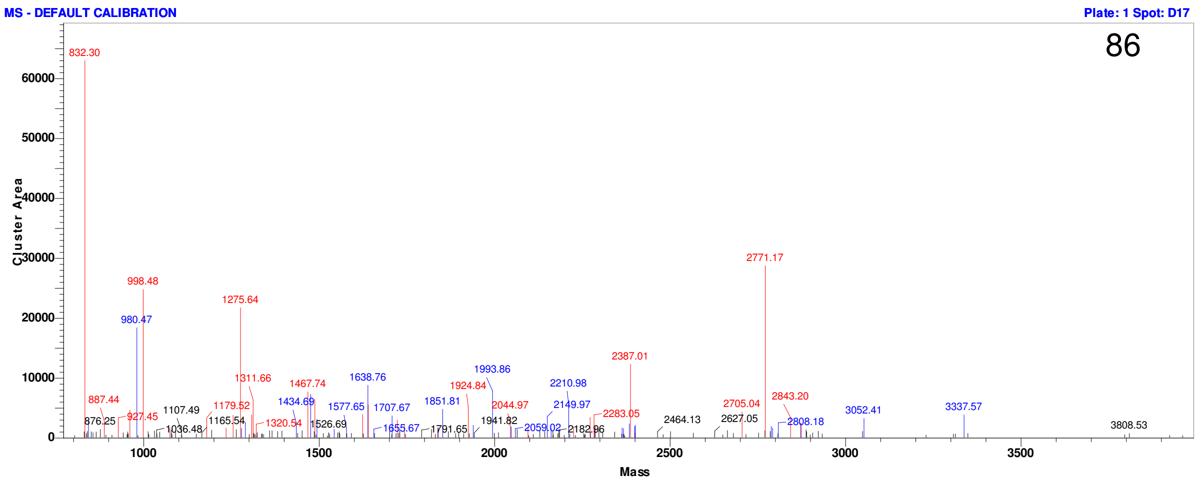
MS - DEFAULT CALIBRATION
Plate: 1 Spot: D17
86
Cluster Area
Mass
832.30
998.48
980.47
1275.64
1311.66
887.44
927.45
876.25
1107.49
1036.48
1179.52
1165.54
1467.74
1434.69
1320.54
1526.69
1577.65
1638.76
1707.67
1655.67
1851.81
1791.65
1924.84
1993.86
1941.82
2044.97
2059.02
2210.98
2149.97
2182.96
2283.05
2387.01
2464.13
2627.05
2705.04
2771.17
2843.20
2808.18
3052.41
3337.57
3808.53

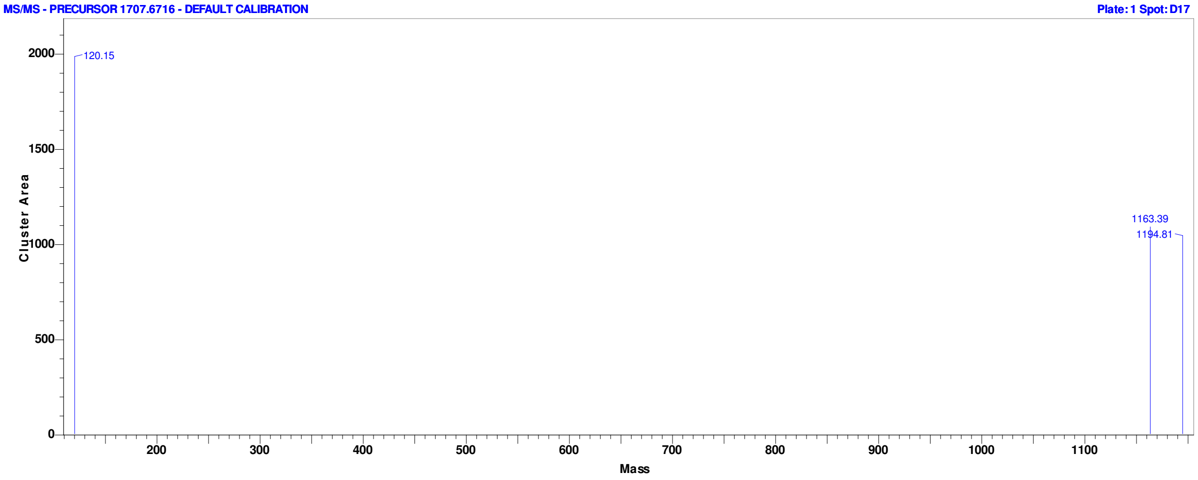
MS/MS - PRECURSOR 1707.6716 - DEFAULT CALIBRATION
Plate: 1 Spot: D17
Cluster Area
Mass
120.15
1163.39
1194.81

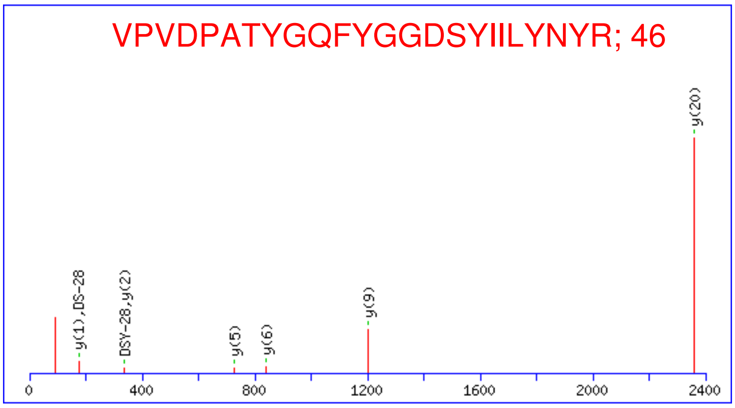
VPVDPATYGQFYGGDSYIILYNYR; 46
y(1),DS-28
DSY-28,y(2)
y(5)
y(6)
y(9)
y(20)

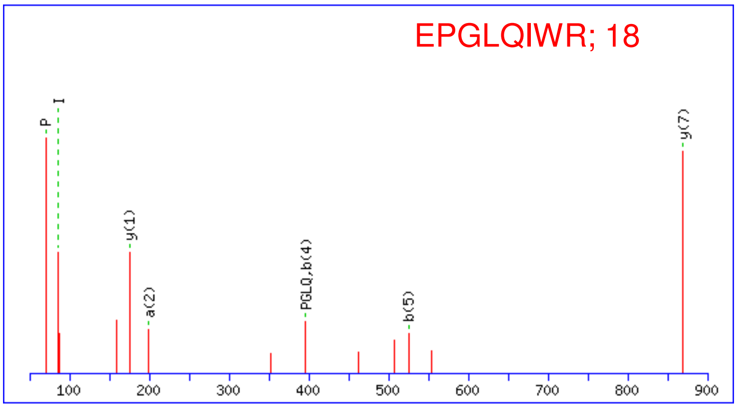
EPGLQIWR; 18
P
I
y(1)
a(2)
PGLQ,b(4)
b(5)
y(7)

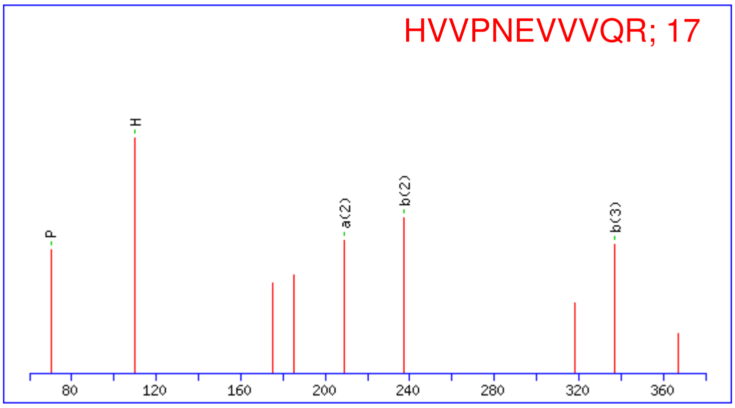
HVVPNEVVVQR; 17
P
H
a(2)
b(2)
b(3)

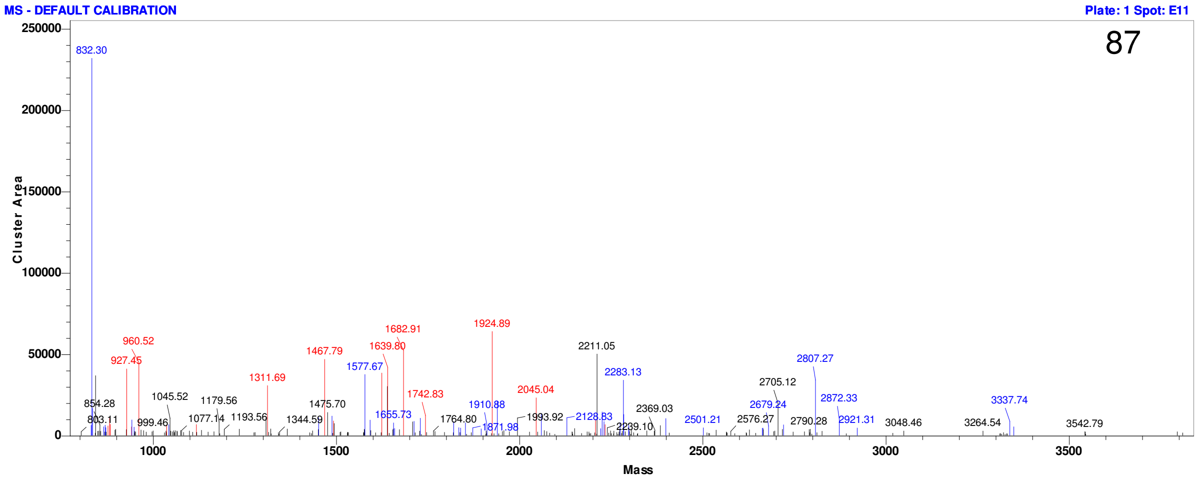

MS - DEFAULT CALIBRATION
Plate: 1 Spot: E11
87
Cluster Area
Mass
832.30
960.52
927.45
1682.91
1924.89
1639.80
1467.79
1577.67
1311.69
2211.05
2283.13
2807.27
2705.12
1742.83
2045.04
1045.52
1179.56
1475.70
854.28
1910.88
2679.24
2872.33
3337.74
1655.73
2369.03
803.11
1077.14
1193.56
1344.59
1764.80
1993.92
2128.83
2501.21
2576.27
2790.28
2921.31
3048.46
3264.54
3542.79
999.46
1871.98
2239.10

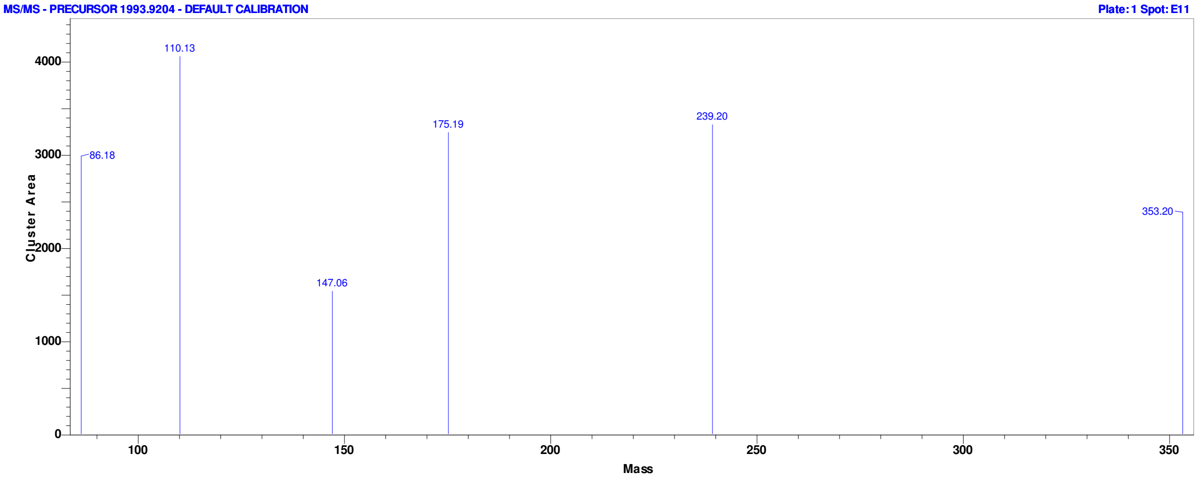

MS/MS - PRECURSOR 1993.9204 - DEFAULT CALIBRATION
Plate: 1 Spot: E11
Cluster Area
Mass
110.13
86.18
175.19
239.20
353.20
147.06

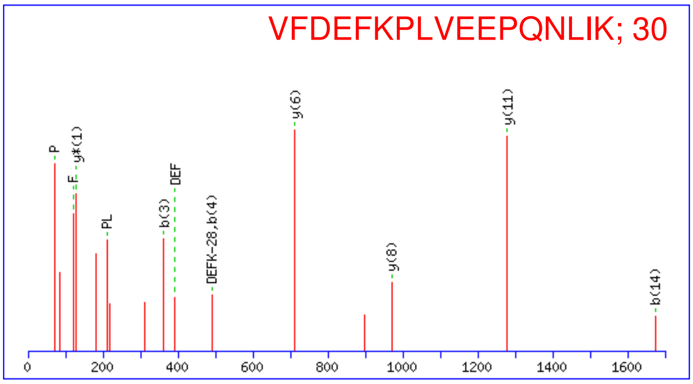

VFDEFKPLVEEPQNLIK; 30
P
F
y*(1)
PL
b(3)
DEF
DEFK-28,b(4)
y(6)
y(8)
y(11)
b(14)

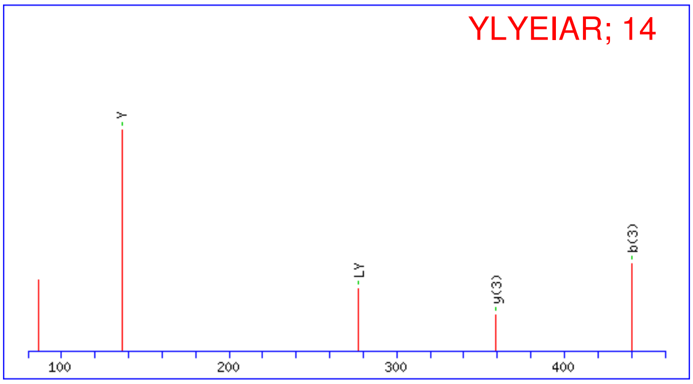

YLYEIAR; 14
Y
LY
y(3)
b(3)

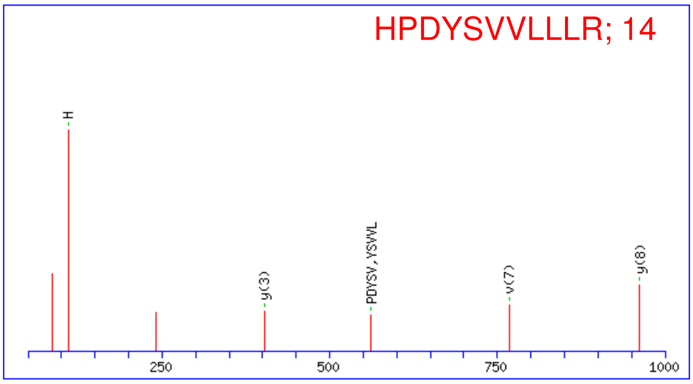

HPDYSVVLLLR; 14
H
y(3)
PDYSV,YSVVL
y(7)
y(8)

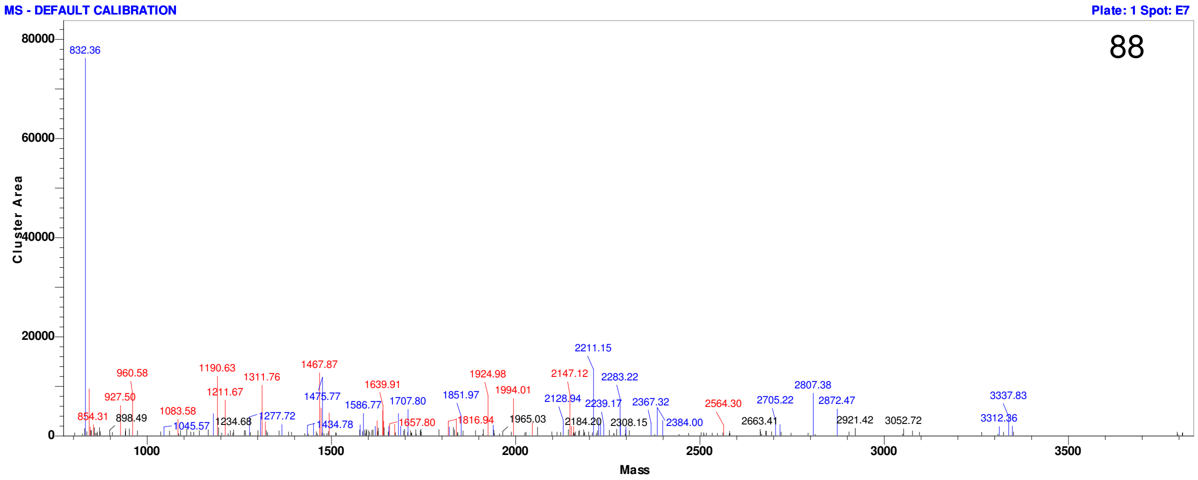
MS - DEFAULT CALIBRATION
Plate: 1 Spot: E7
88
Cluster Area
Mass
832.36
960.58
927.50
854.31
898.49
1083.58
1045.57
1190.63
1211.67
1234.68
1311.76
1277.72
1467.87
1475.77
1434.78
1639.91
1586.77
1707.80
1657.80
1851.97
1816.94
1924.98
1994.01
1965.03
2147.12
2128.94
2184.20
2211.15
2239.17
2283.22
2308.15
2367.32
2384.00
2564.30
2663.41
2705.22
2807.38
2872.47
2921.42
3052.72
3337.83
3312.36

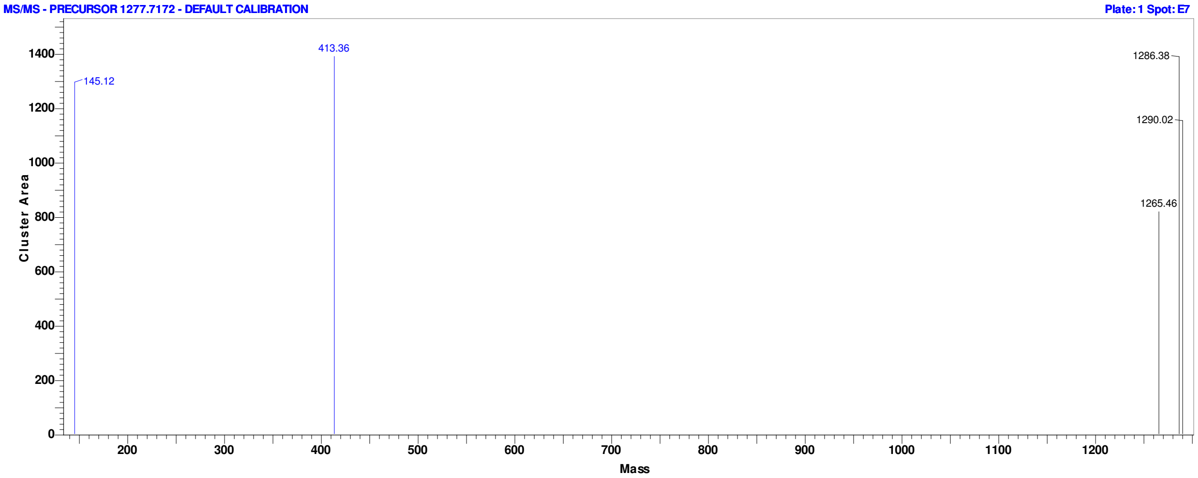
MS/MS - PRECURSOR 1277.7172 - DEFAULT CALIBRATION
Plate: 1 Spot: E7
Cluster Area
Mass
145.12
413.36
1286.38
1290.02
1265.46

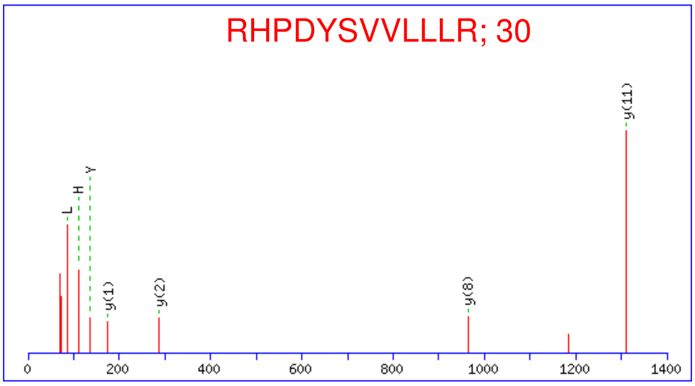
RHPDYSVVLLLR; 30
L
H
Y
y(1)
y(2)
y(8)
y(11)

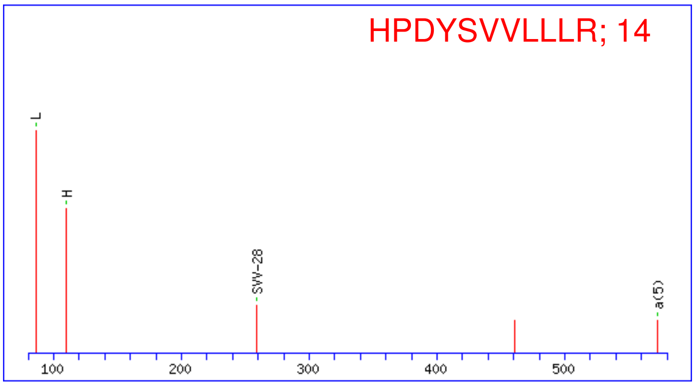
HPDYSVVLLLR; 14
L
H
SW-28
a(5)

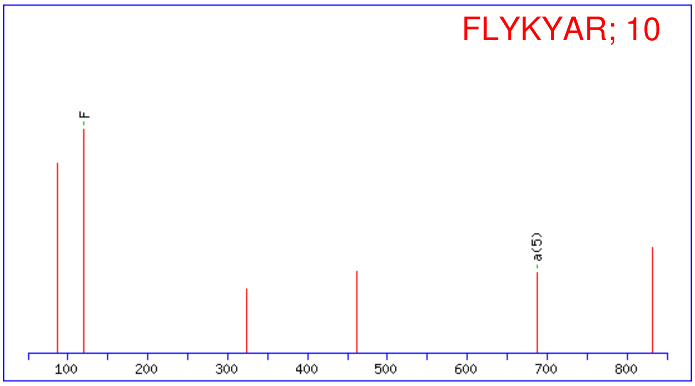
FLYKYAR; 10
F
a(5)

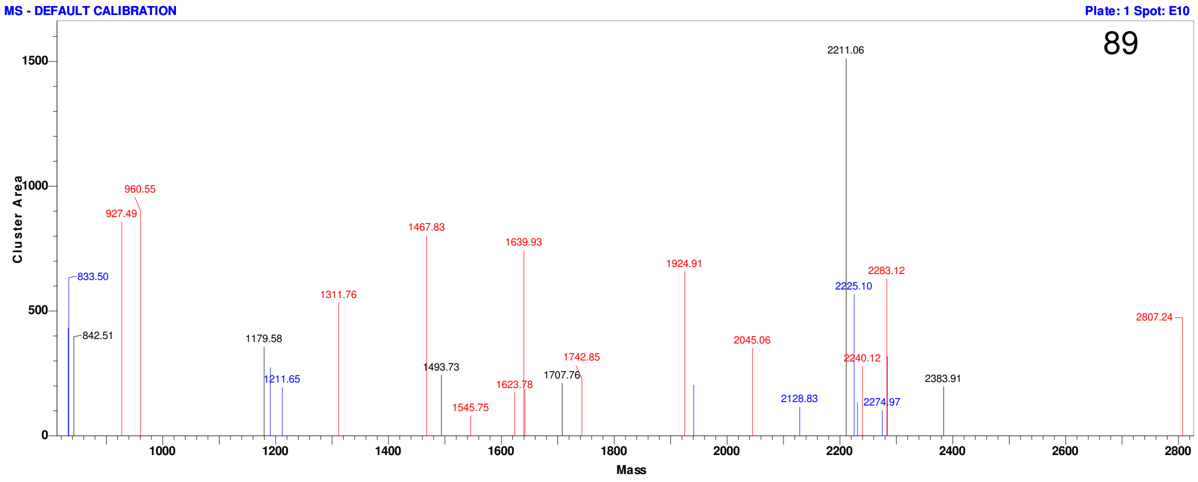
MS - DEFAULT CALIBRATION
Plate: 1 Spot: E10
89
Cluster Area
Mass
2211.06
960.55
927.49
1467.83
1639.93
1924.91
2283.12
833.50
2225.10
1311.76
2807.24
842.51
1179.58
2045.06
1742.85
1493.73
2240.12
1211.65
1623.78
1707.76
2383.91
2128.83
2274.97
1545.75

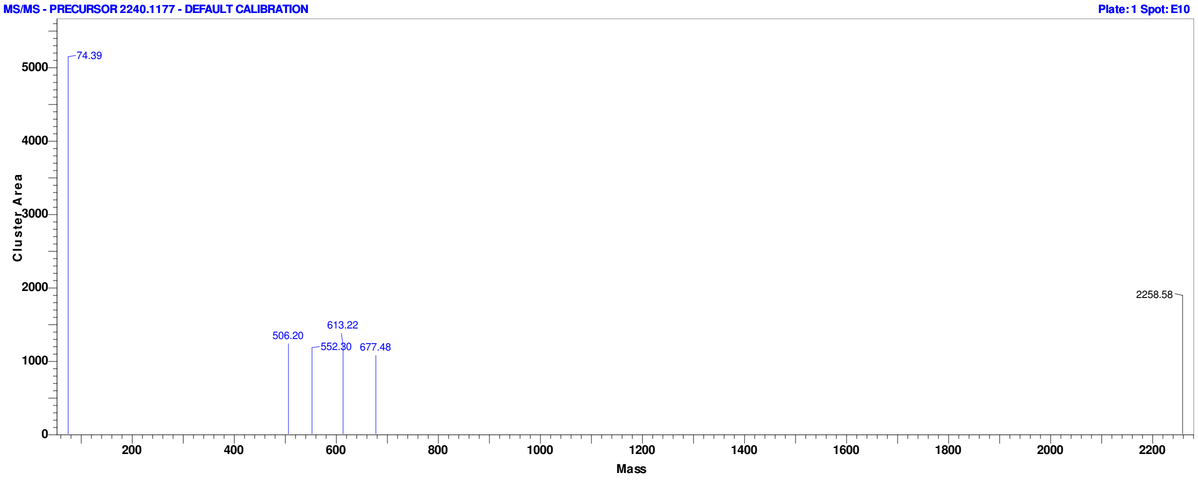
MS/MS - PRECURSOR 2240.1177 - DEFAULT CALIBRATION
Plate: 1 Spot: E10
Cluster Area
Mass
74.39
2258.58
613.22
506.20
552.30
677.48

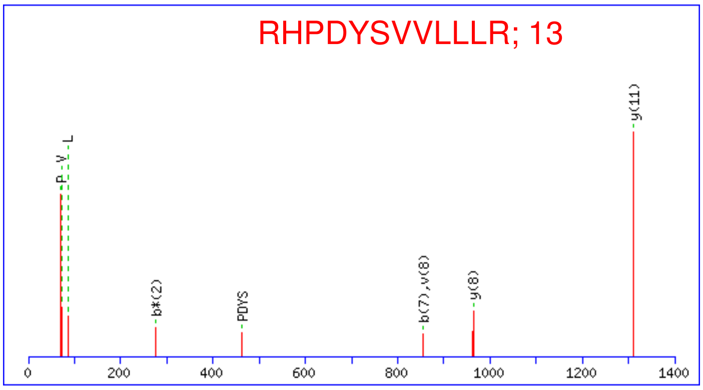
RHPDYSVVLLLR; 13
P
V
L
b*(2)
PDYS
b(7),y(8)
y(8)
y(11)

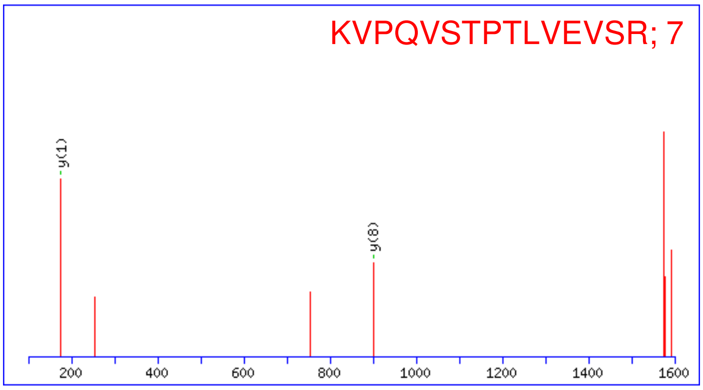
KVPQVSTPTLVEVSR; 7
y(1)
y(8)

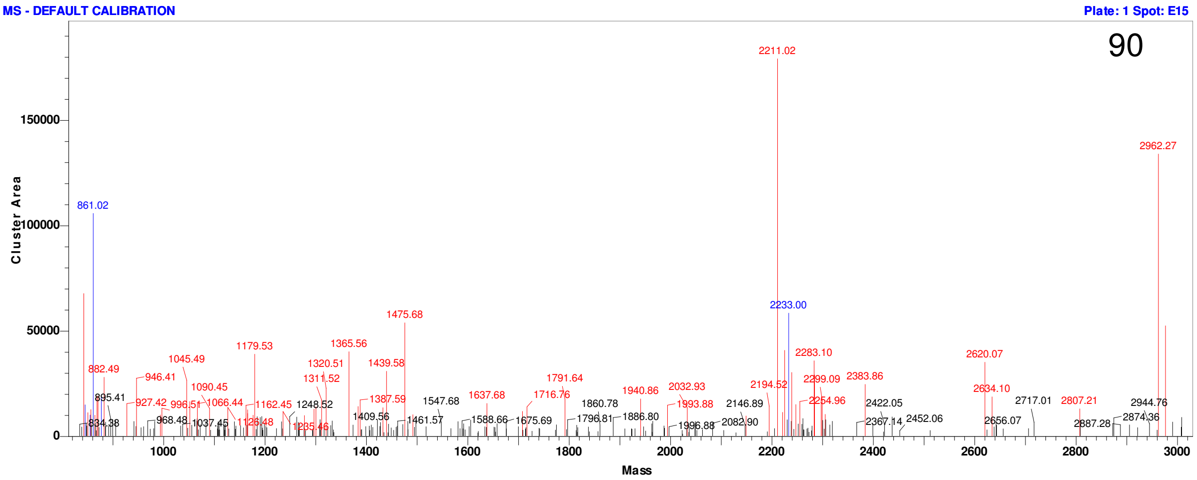

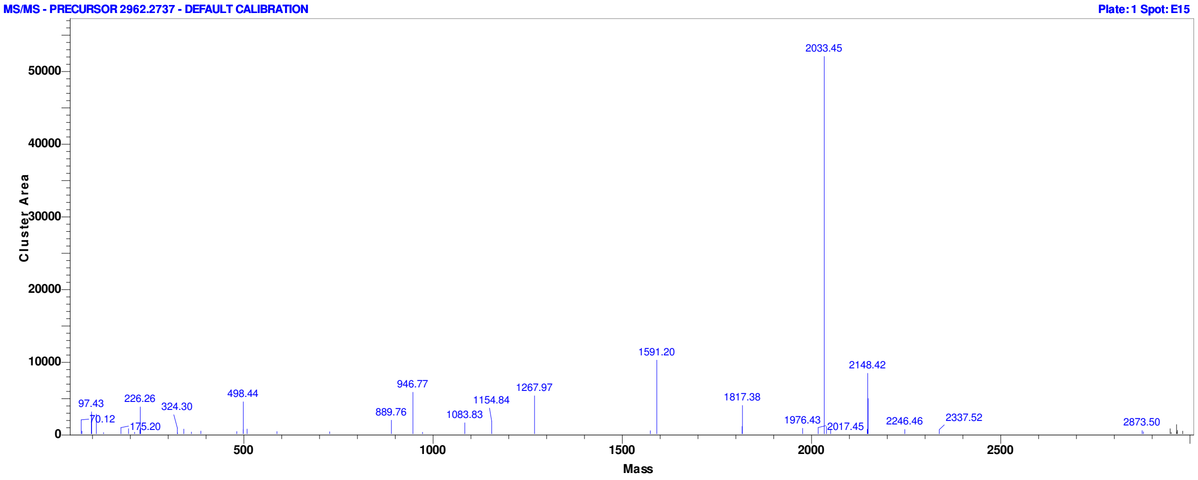

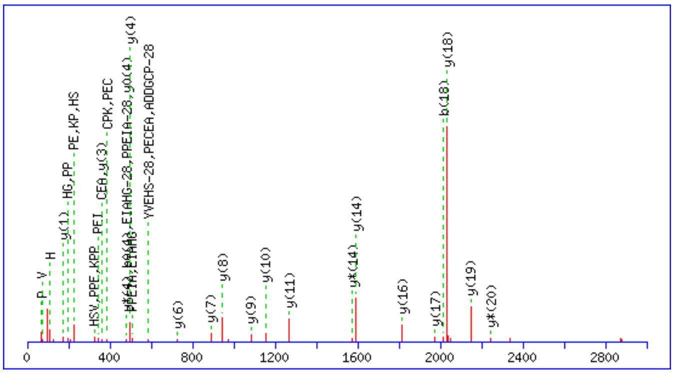

LPECEADDGCPKPPEIAHGYVEHSVR; 106

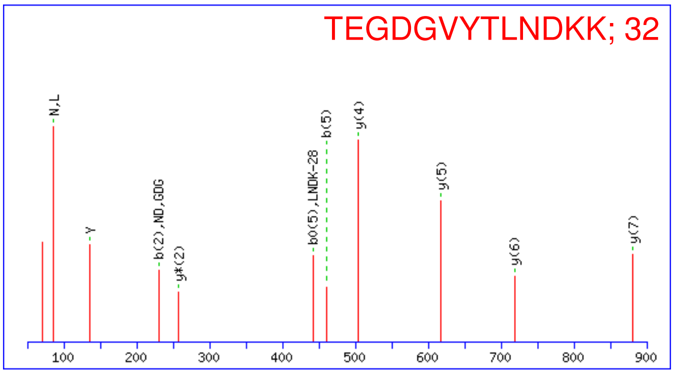

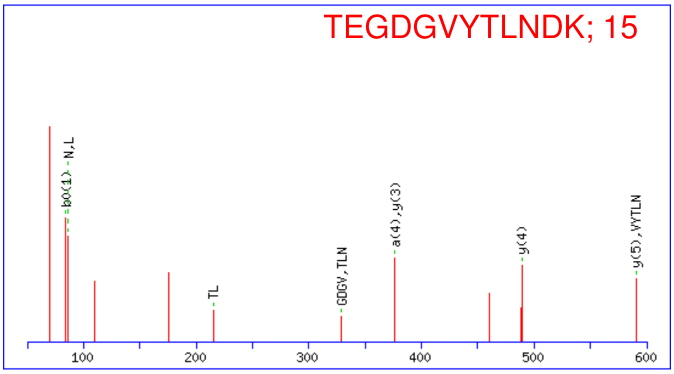

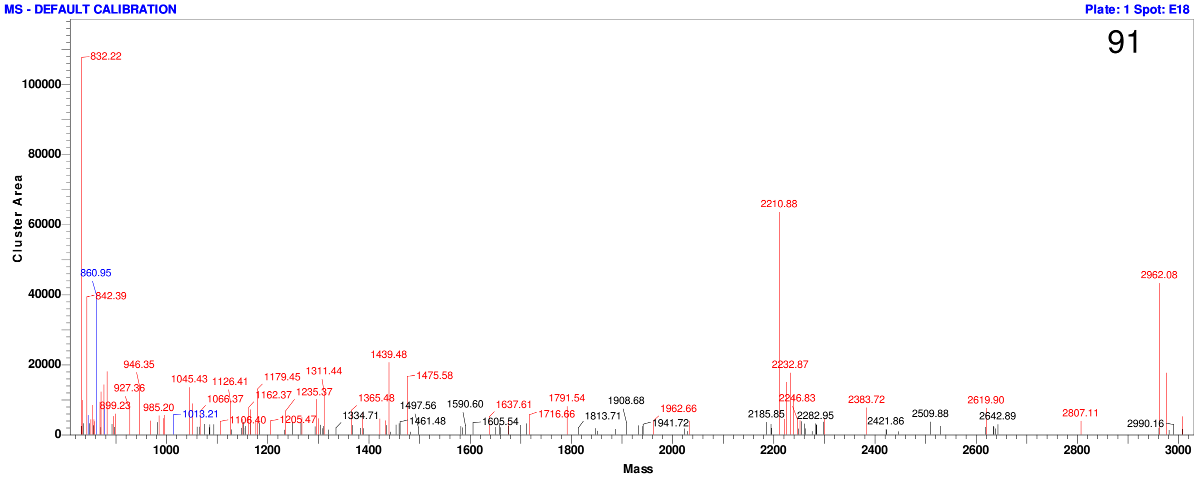
MS - DEFAULT CALIBRATION
Plate: 1 Spot: E18
91
Cluster Area
Mass
832.22
2210.88
2962.08
860.95
842.39
946.35
927.36
899.23
985.20
1013.21
1045.43
1066.37
1126.41
1106.40
1179.45
1162.37
1205.47
1235.37
1311.44
1334.71
1365.48
1439.48
1461.48
1475.58
1497.56
1590.60
1605.54
1637.61
1716.66
1791.54
1813.71
1908.68
1941.72
1962.66
2185.85
2232.87
2246.83
2282.95
2383.72
2421.86
2509.88
2619.90
2642.89
2807.11
2990.16

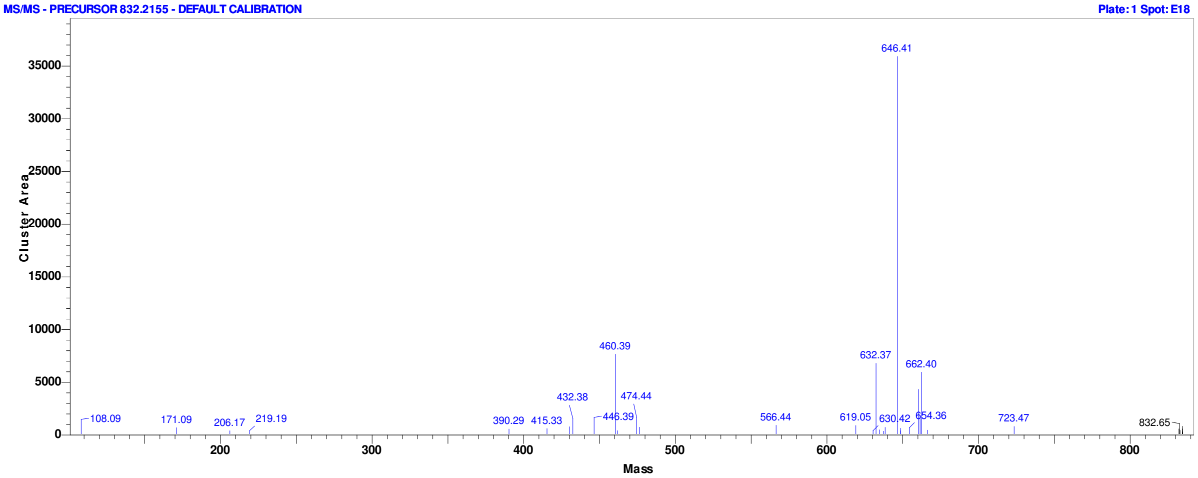
MS/MS - PRECURSOR 832.2155 - DEFAULT CALIBRATION
Plate: 1 Spot: E18
Cluster Area
Mass
646.41
460.39
632.37
662.40
432.38
474.44
446.39
108.09
171.09
206.17
219.19
390.29
415.33
566.44
619.05
630.42
654.36
723.47
832.65

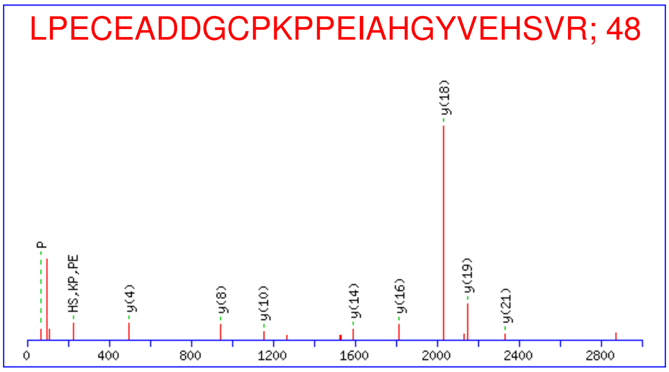
LPECEADDGCPKPPEIAHGYVEHSVR; 48
P
HS,KP,PE
y(4)
y(8)
y(10)
y(14)
y(16)
y(18)
y(19)
y(21)

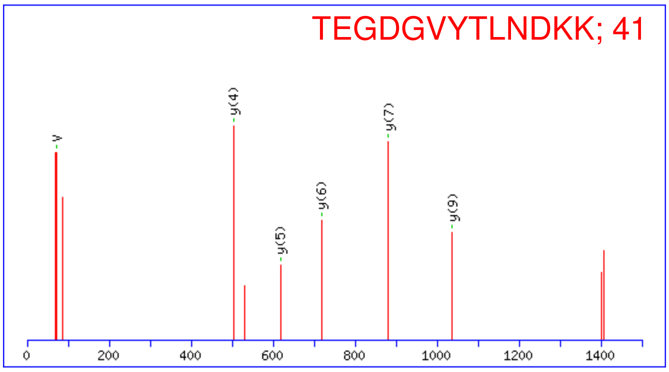
TEGDGVYTLNDKK; 41
V
y(4)
y(5)
y(6)
y(7)
y(9)

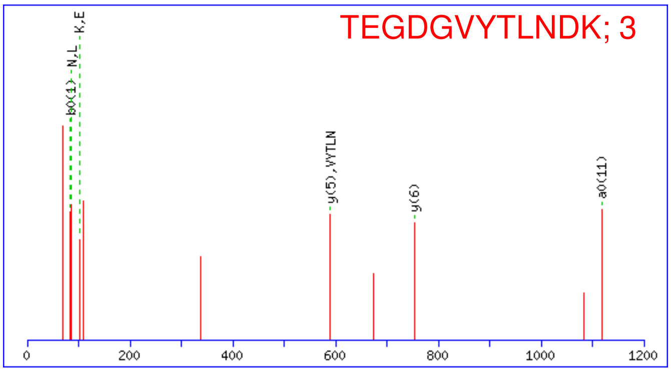
TEGDGVYTLNDK; 3
b0(1)
N,L
K,E
y(5),VYTLN
y(6)
a0(11)

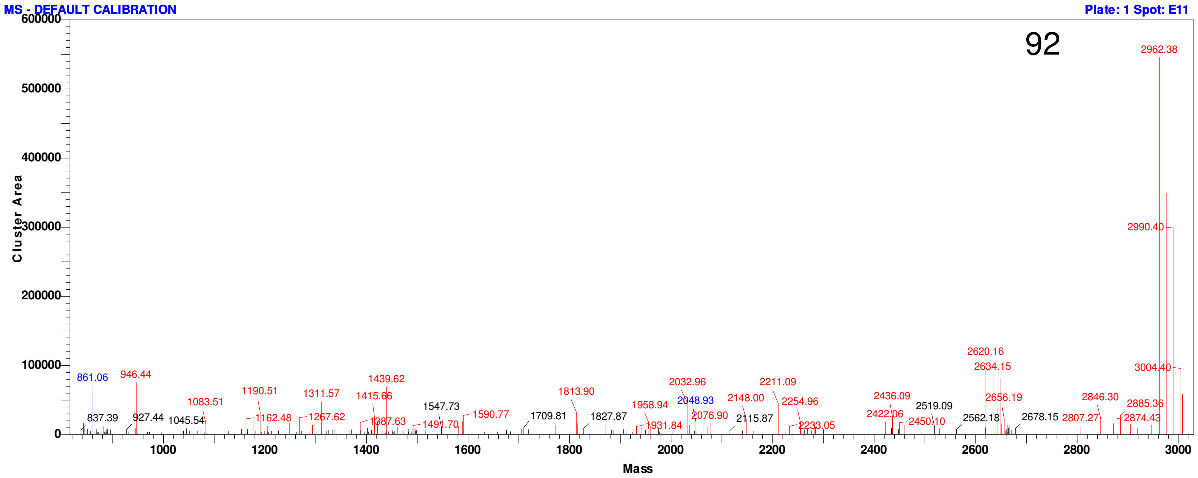

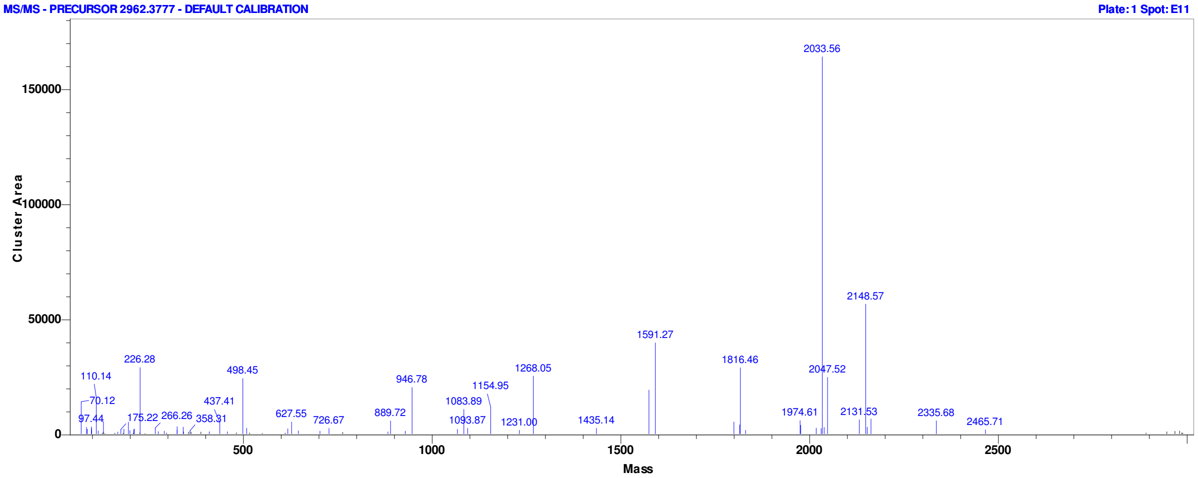

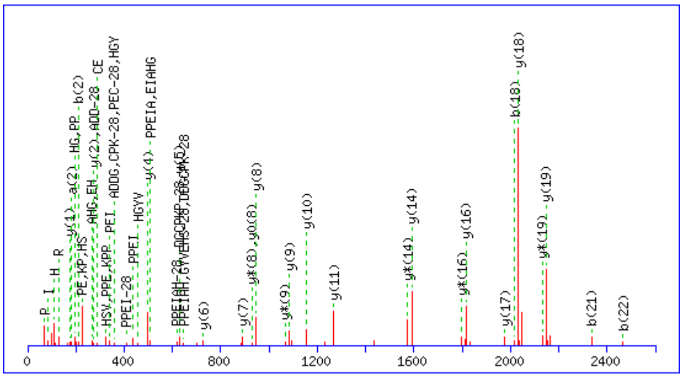

LPECEADDGCPKPPEIAHGYVEHSVR; 108

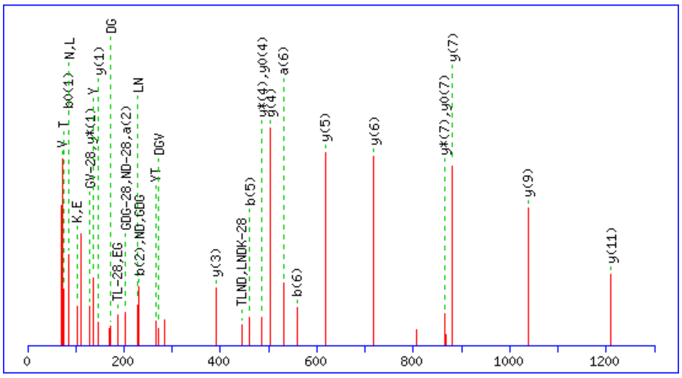

TEGDGVYTLNDKK; 68

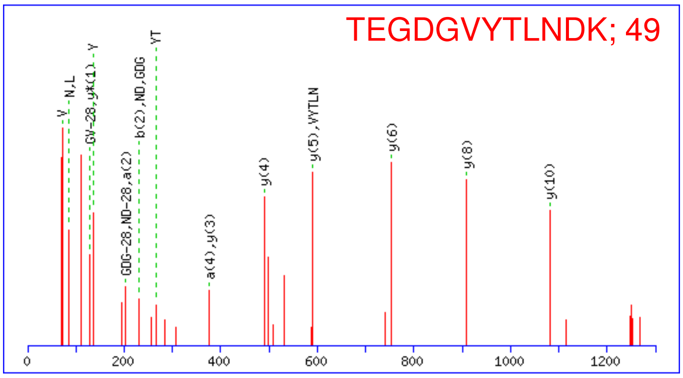

TEGDGVYTLNDK; 49

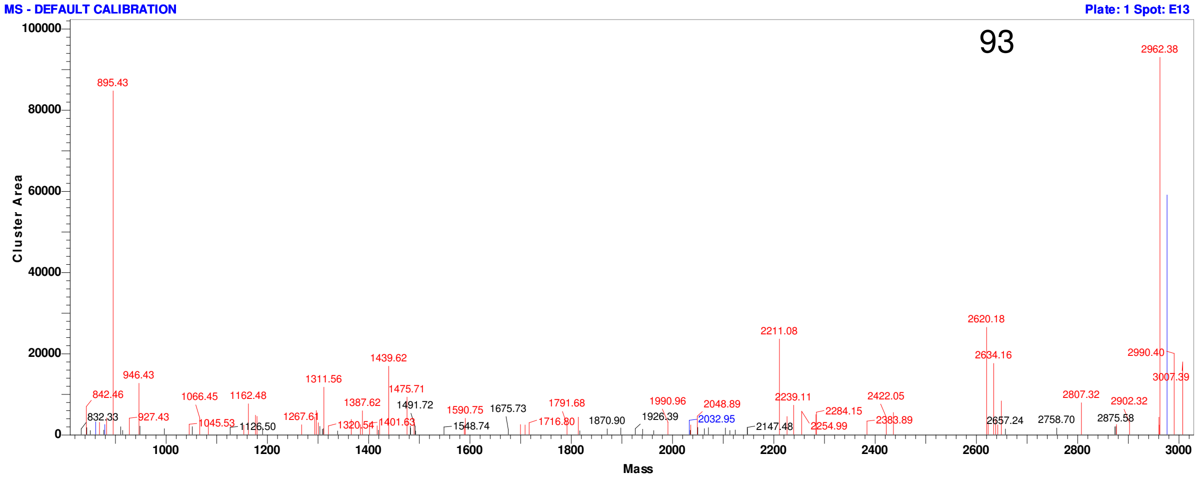

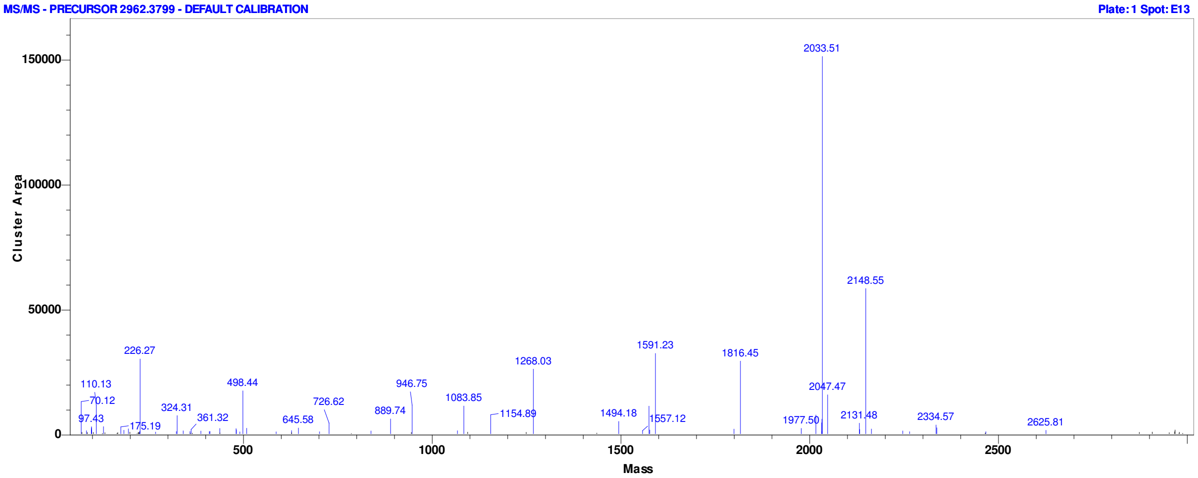

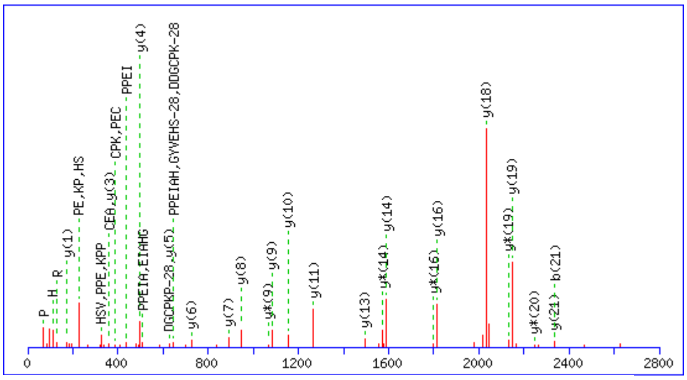

LPECEADDGCPKPPEIAHGYVEHSVR; 131

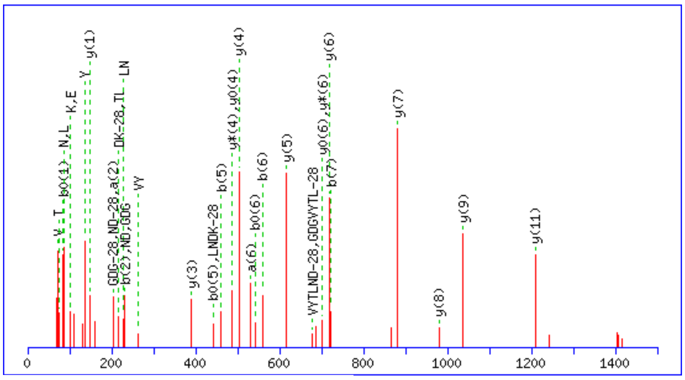

TEGDGVYTLNDKK; 78

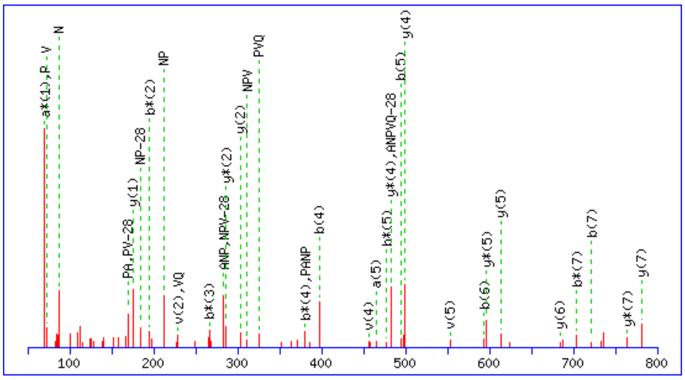

NPANPVQR; 47

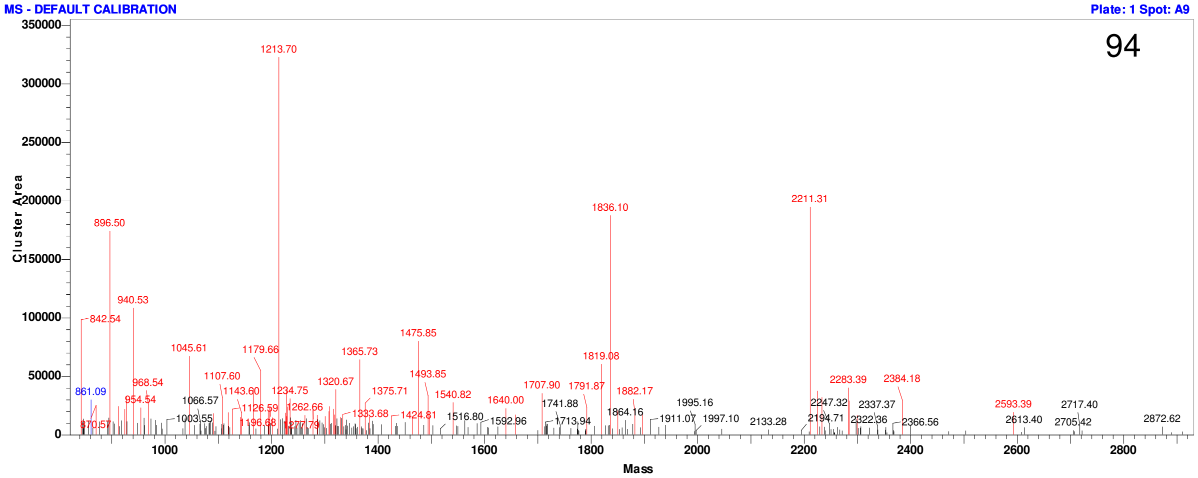

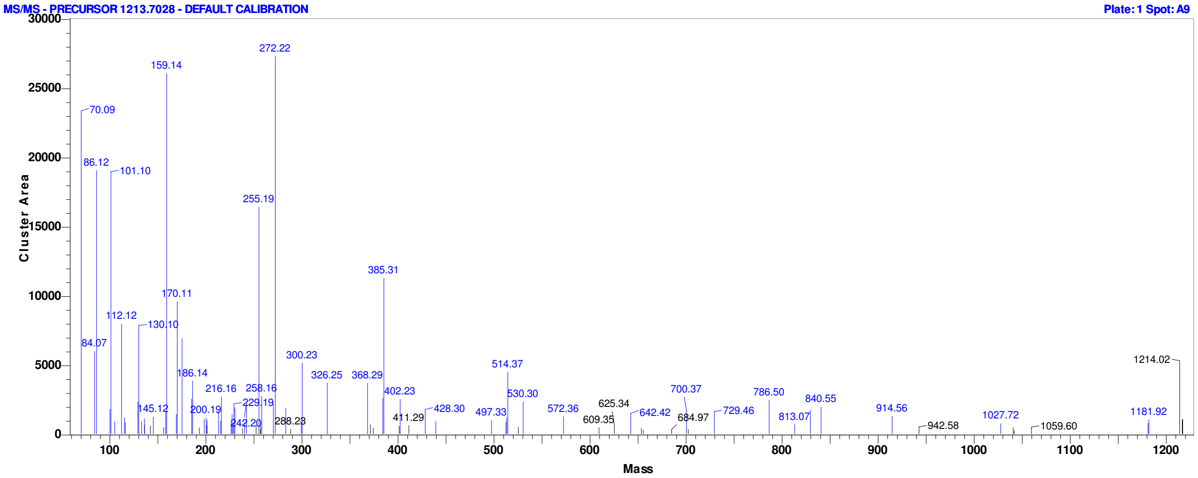

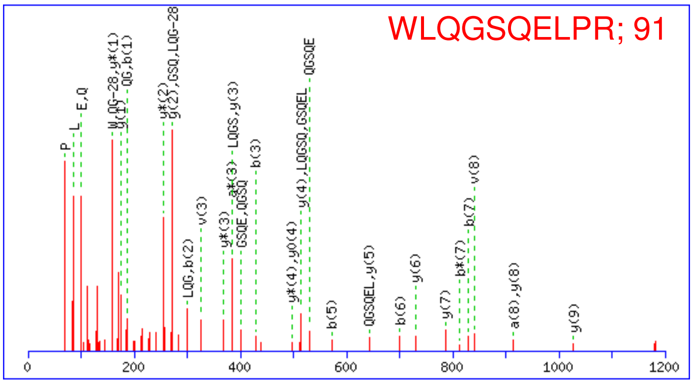

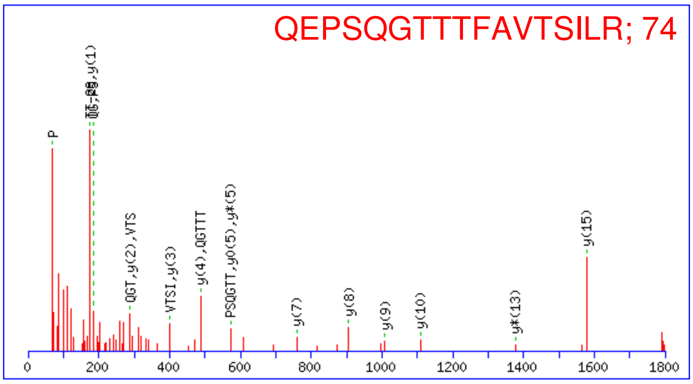

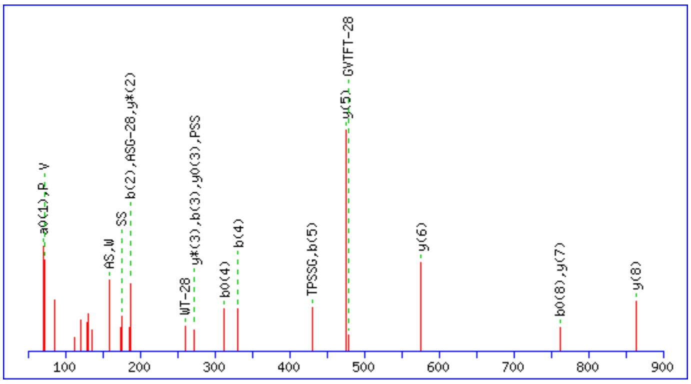

DASGVTFTWTPSSGK; 35

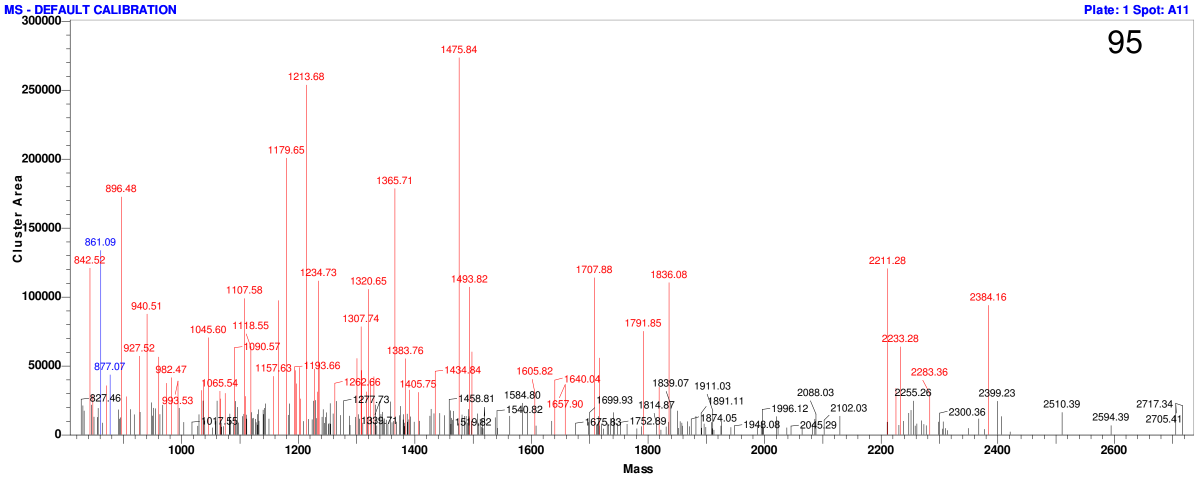
MS - DEFAULT CALIBRATION
Plate: 1 Spot: A11
95
Cluster Area
Mass
1475.84
1213.68
1179.65
1365.71
896.48
861.09
842.52
1234.73
1320.65
1493.82
1707.88
1836.08
2211.28
2384.16
940.51
1107.58
1118.55
1045.60
1307.74
1791.85
2233.28
927.52
1090.57
1383.76
877.07
982.47
1157.63
1193.66
1434.84
1605.82
2283.36
1065.54
1262.66
1405.75
1640.04
827.46
993.53
1277.73
1458.81
1584.80
1839.07
1911.03
2088.03
2255.26
2399.23
1540.82
1657.90
1699.93
1891.11
1996.12
2102.03
2510.39
2717.34
1017.55
1339.71
1519.62
1675.83
1752.89
1814.87
1874.05
1948.08
2045.29
2300.36
2594.39
2705.41

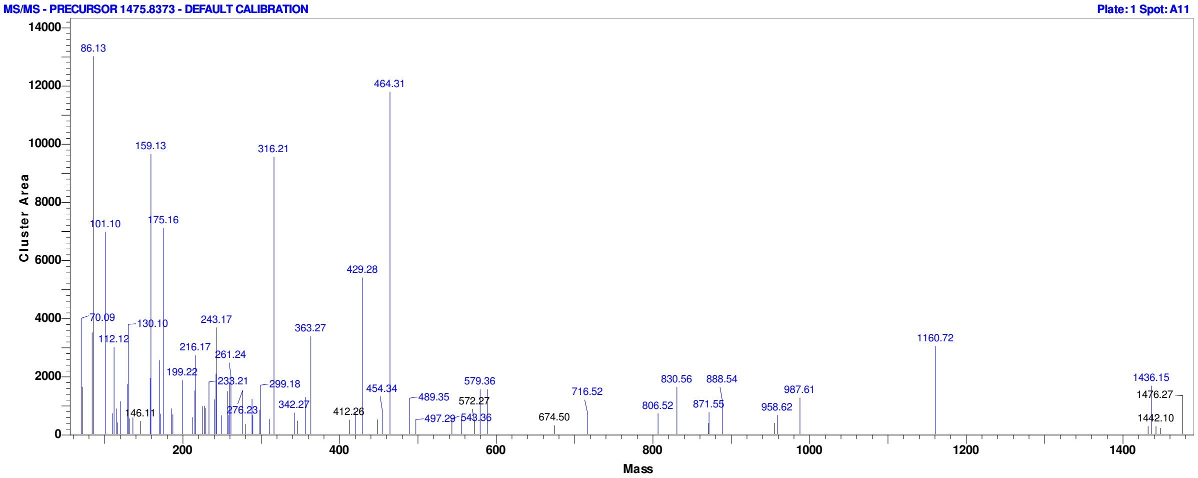
MS/MS - PRECURSOR 1475.8373 - DEFAULT CALIBRATION
Plate: 1 Spot: A11
Cluster Area
Mass
86.13
464.31
159.13
316.21
175.16
101.10
429.28
70.09
130.10
243.17
363.27
1160.72
112.12
216.17
261.24
199.22
233.21
299.18
454.34
579.36
830.56
888.54
1436.15
489.35
572.27
716.52
987.61
1476.27
146.11
276.23
342.27
412.26
497.29
543.36
674.50
806.52
871.55
958.62
1442.10

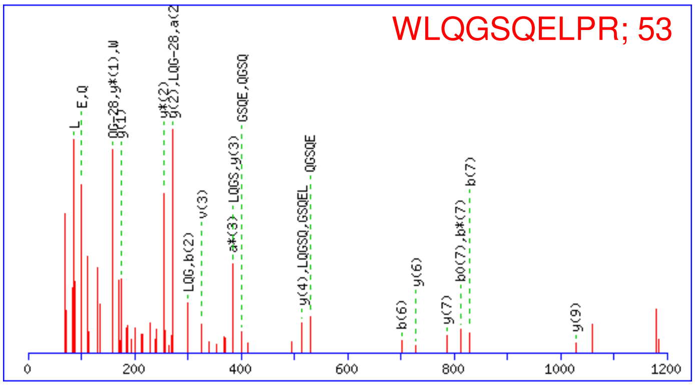
WLQGSQELPR; 53

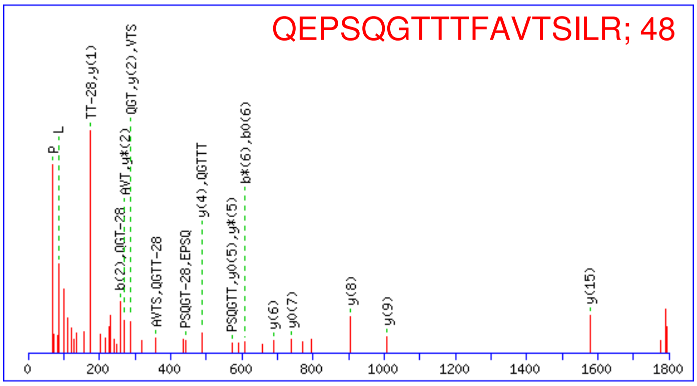
QEPSQGTTTFAVTSILR; 48

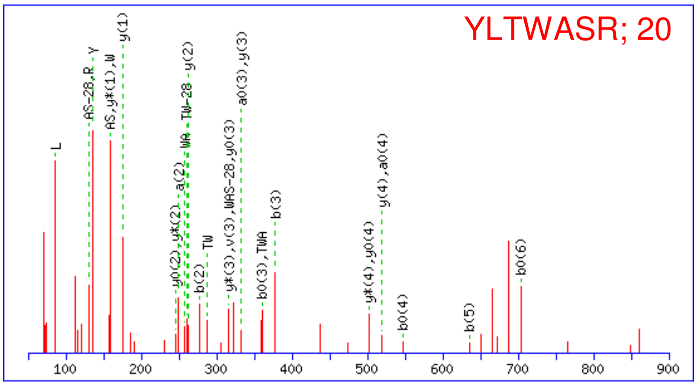
YLTWASR; 20

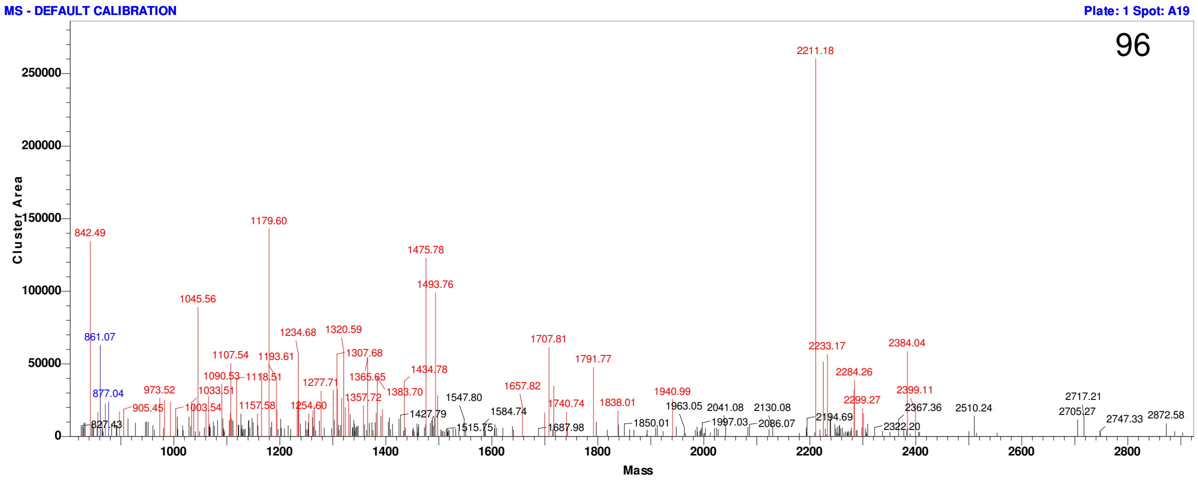

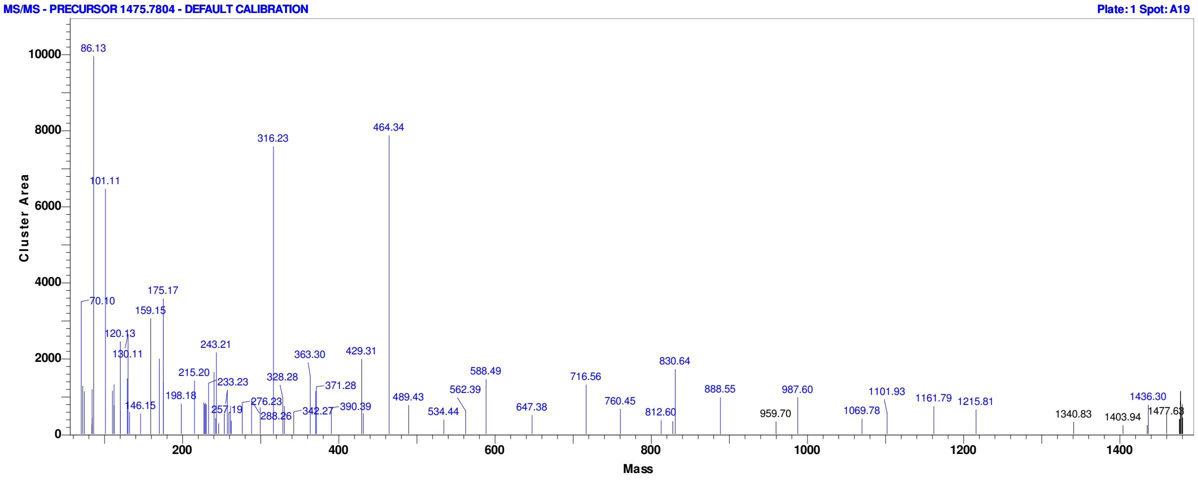

Glypican-1 precursor

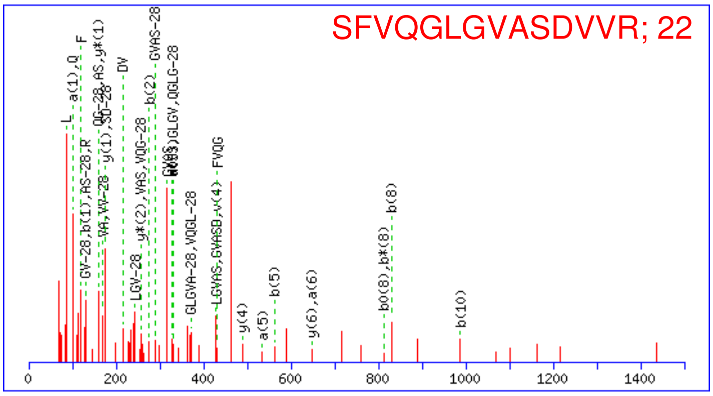

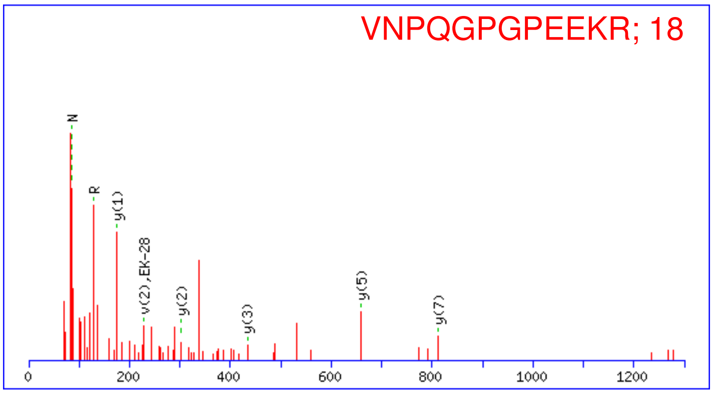

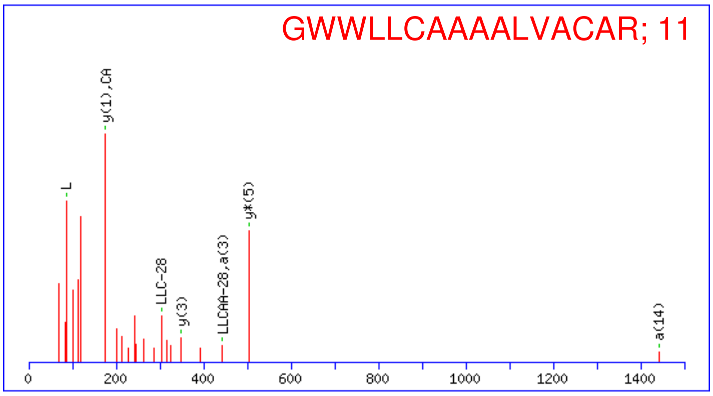

Phenylalanine-4-hydroxylase

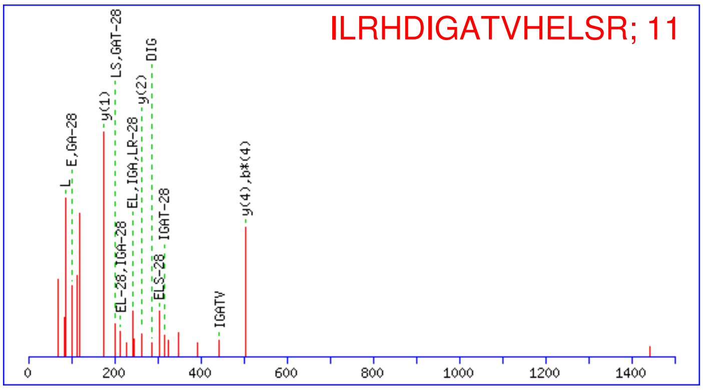

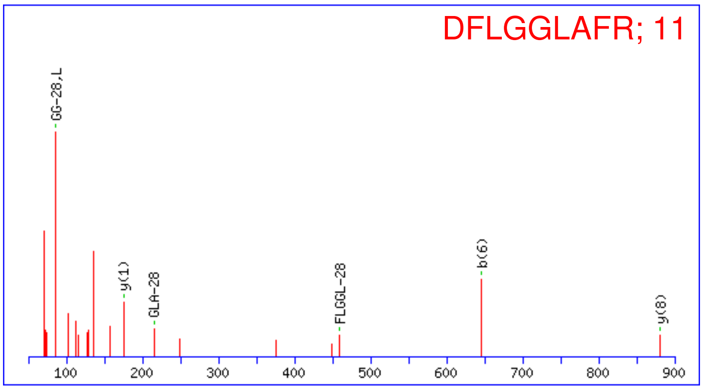

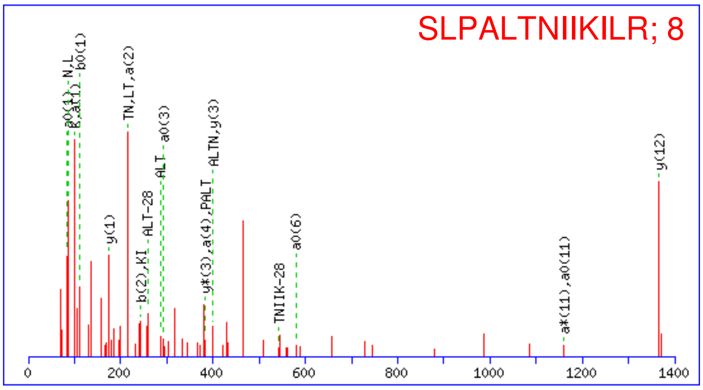

Pro-neuregulin-3 precursor (Pro-NRG3)

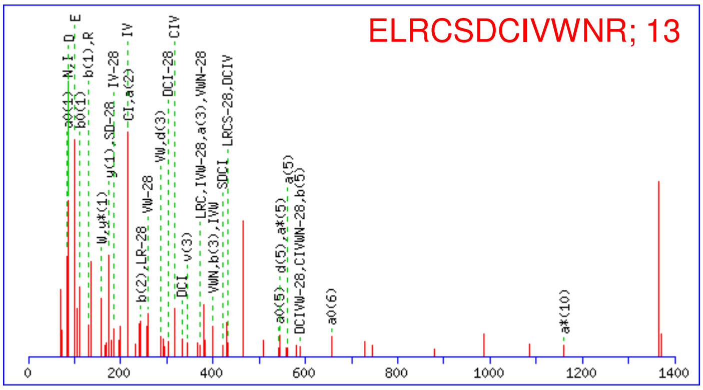

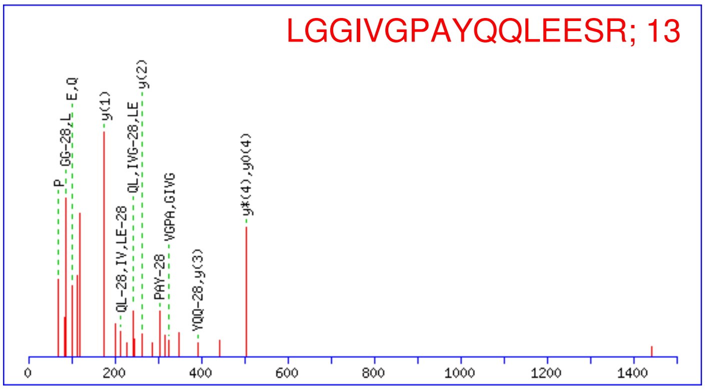

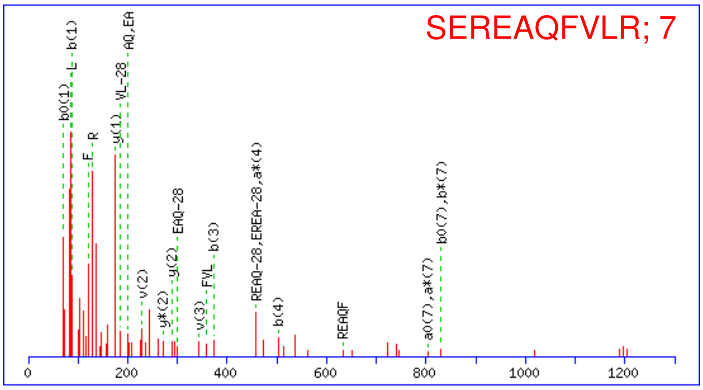

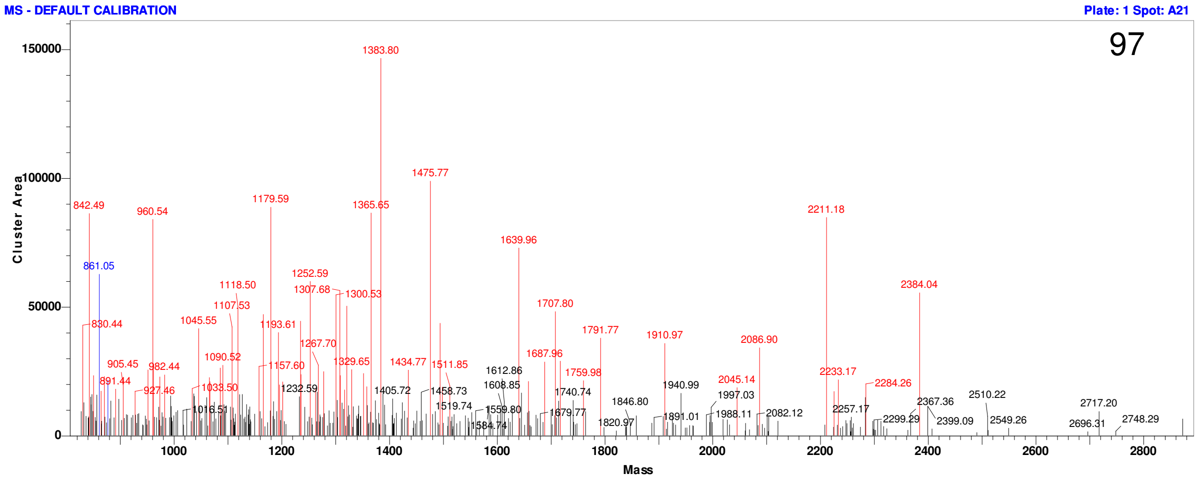
MS - DEFAULT CALIBRATION
Plate: 1 Spot: A21
97
Cluster Area
Mass

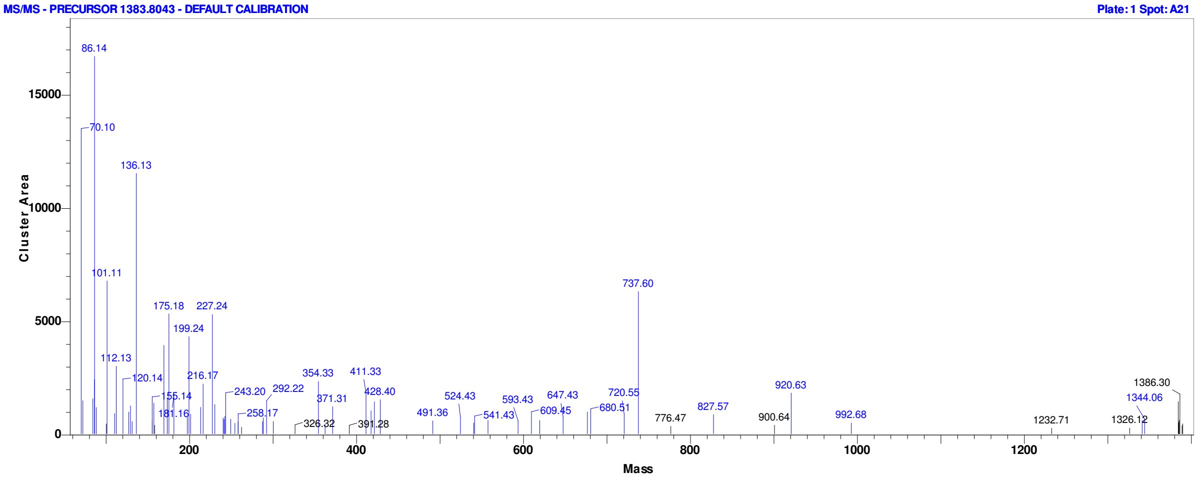
MS/MS - PRECURSOR 1383.8043 - DEFAULT CALIBRATION
Plate: 1 Spot: A21
Cluster Area
Mass

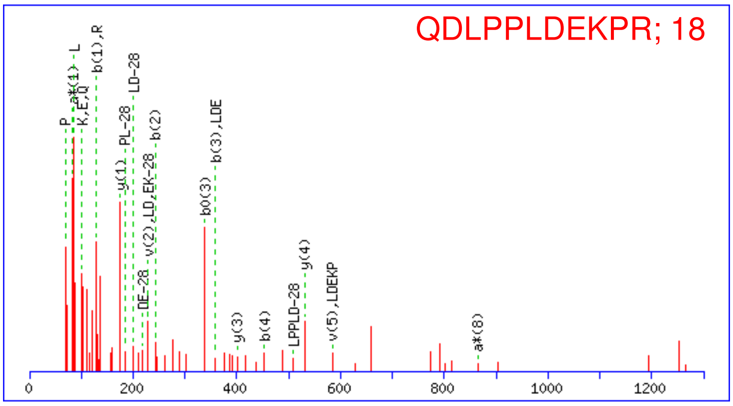
QDLPPLDEKPR; 18

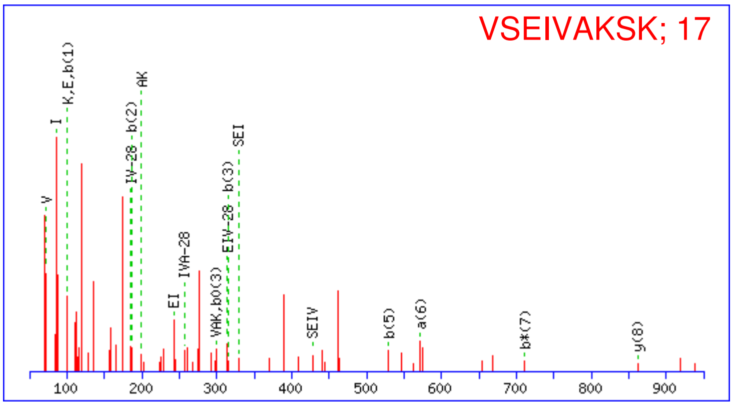
VSEIVAKSK; 17

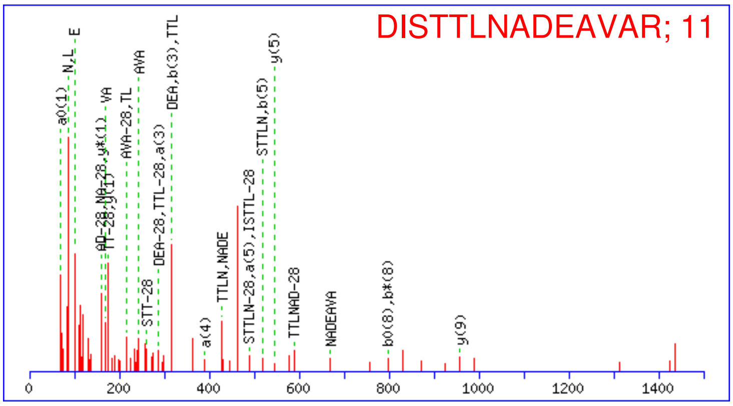
DISTTLNADEAVAR; 11

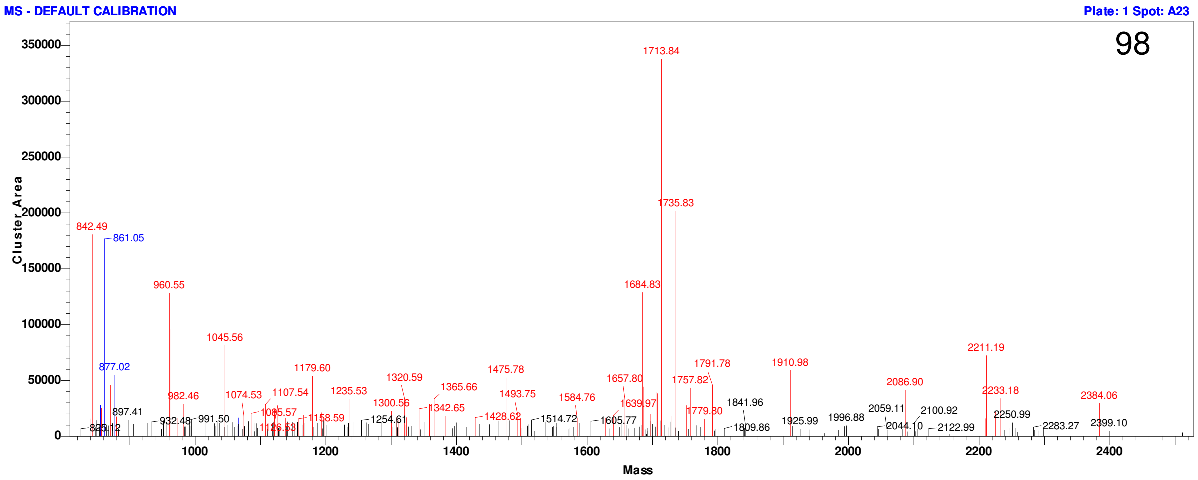

MS - DEFAULT CALIBRATION
Plate: 1 Spot: A23
98
Cluster Area
Mass
1713.84
1735.83
842.49
861.05
960.55
1684.83
1045.56
877.02
1179.60
1475.78
1320.59
1791.78
1910.98
2211.19
1657.80
1757.82
1365.66
1235.53
1107.54
1074.53
982.46
1493.75
1584.76
1300.56
1342.65
1639.97
1841.96
2086.90
2233.18
2384.06
897.41
1085.57
932.48
991.50
1158.59
1428.62
1254.61
1514.72
1605.77
1779.80
1925.99
1996.88
2059.11
2100.92
2250.99
825.12
1126.53
1809.86
2044.10
2122.99
2283.27
2399.10

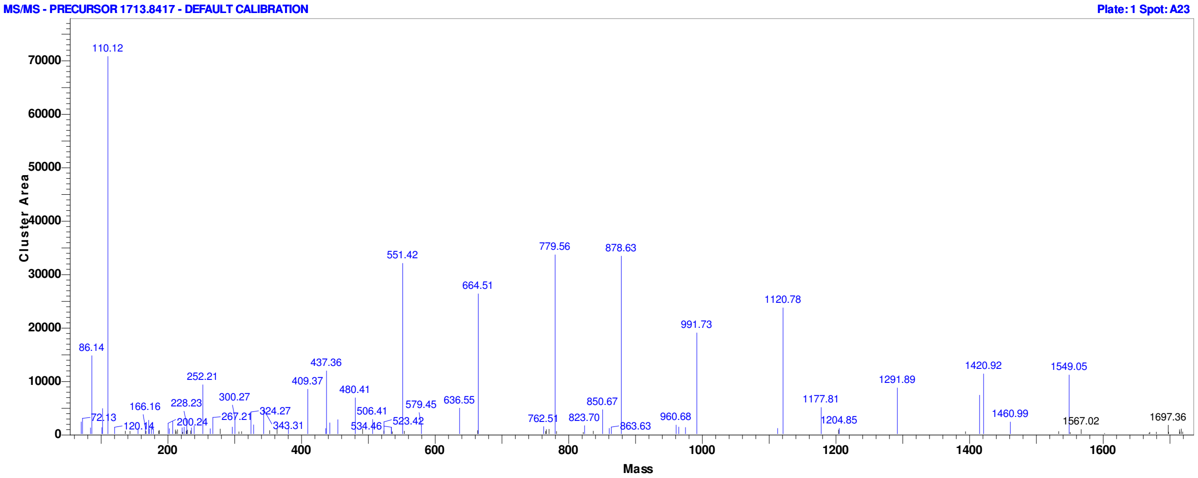

MS/MS - PRECURSOR 1713.8417 - DEFAULT CALIBRATION
Plate: 1 Spot: A23
Cluster Area
Mass
110.12
551.42
779.56
878.63
664.51
1120.78
991.73
86.14
437.36
1420.92
1549.05
252.21
409.37
1291.89
480.41
300.27
166.16
228.23
324.27
579.45
636.55
850.67
1177.81
1460.99
72.13
200.24
267.21
506.41
762.51
823.70
960.68
1204.85
1697.36
120.14
343.31
534.46
523.42
863.63
1567.02

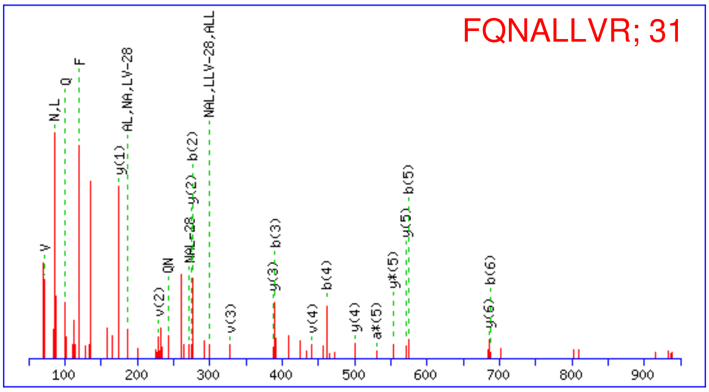

FQNALLVR; 31

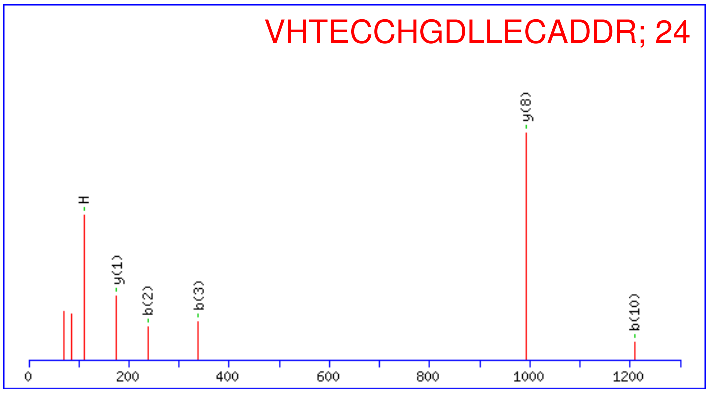

VHTECCHGDLLECADDR; 24

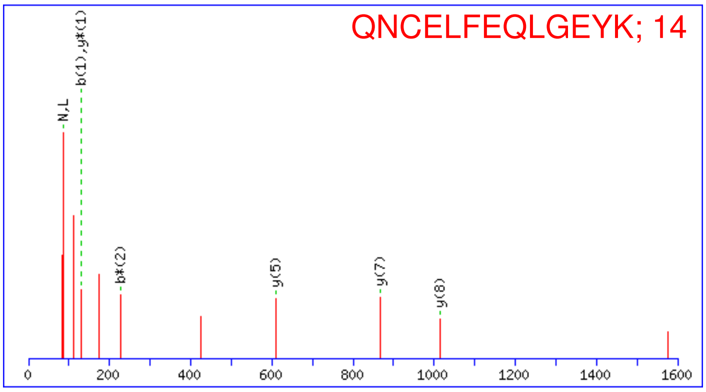

QNCELFEQLGEYK; 14

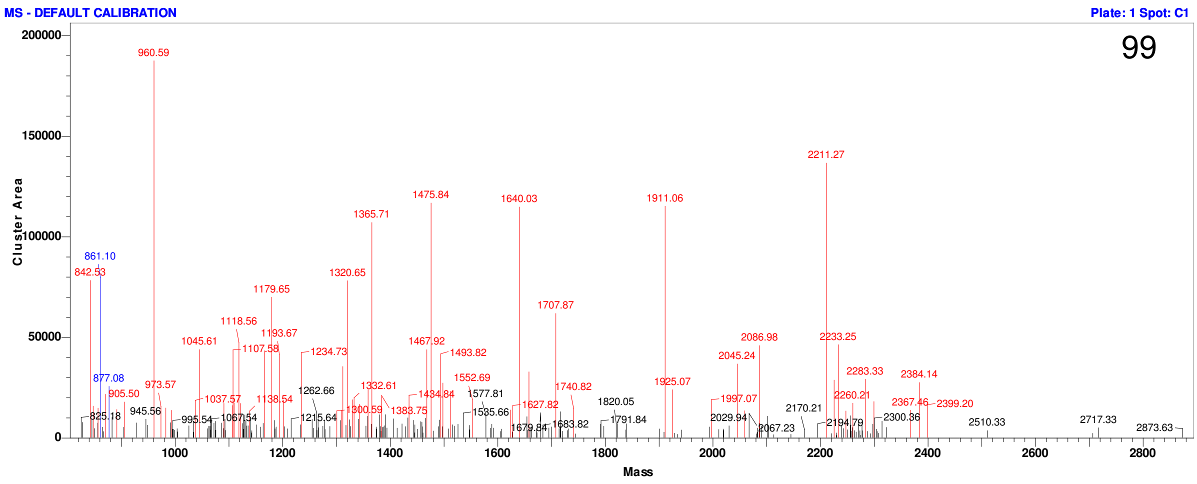
MS - DEFAULT CALIBRATION
Plate: 1 Spot: C1
99
Cluster Area
Mass
960.59
2211.27
1475.84
1640.03
1911.06
1365.71

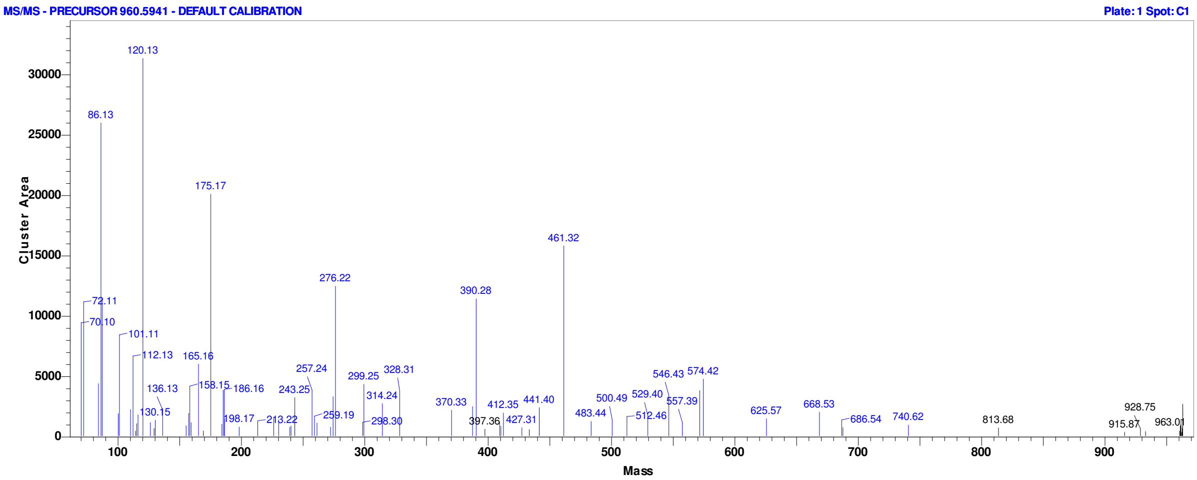
MS/MS - PRECURSOR 960.5941 - DEFAULT CALIBRATION
Plate: 1 Spot: C1
Cluster Area
Mass
120.13
86.13
175.17
461.32
276.22
390.28

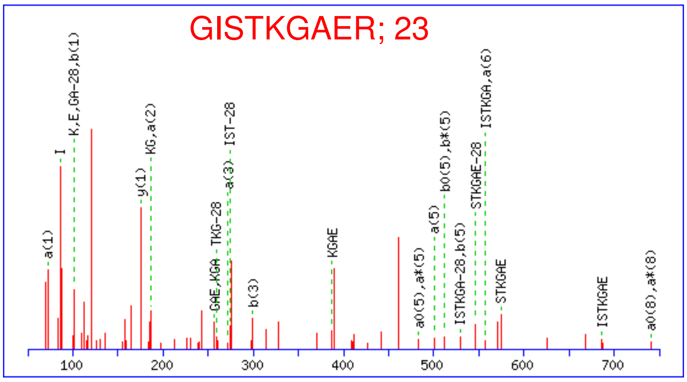
GISTKGAER; 23

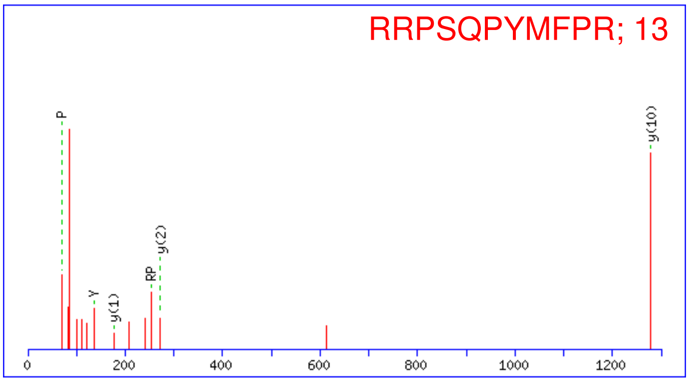
RRPSQPYMFPR; 13

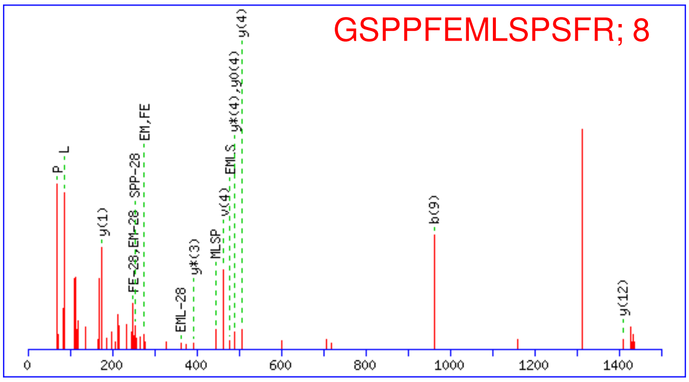
GSPPFEMLSPSFR; 8

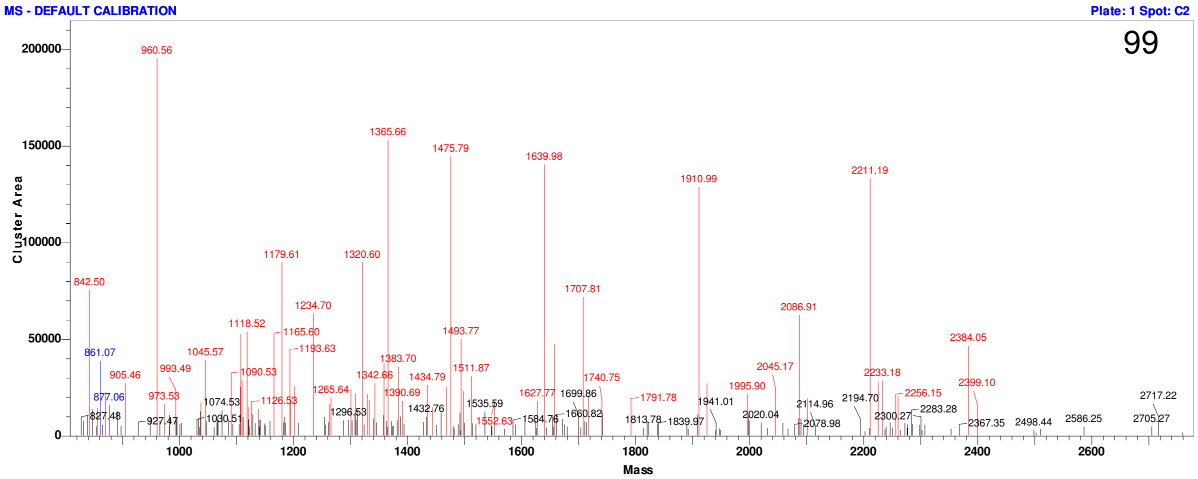

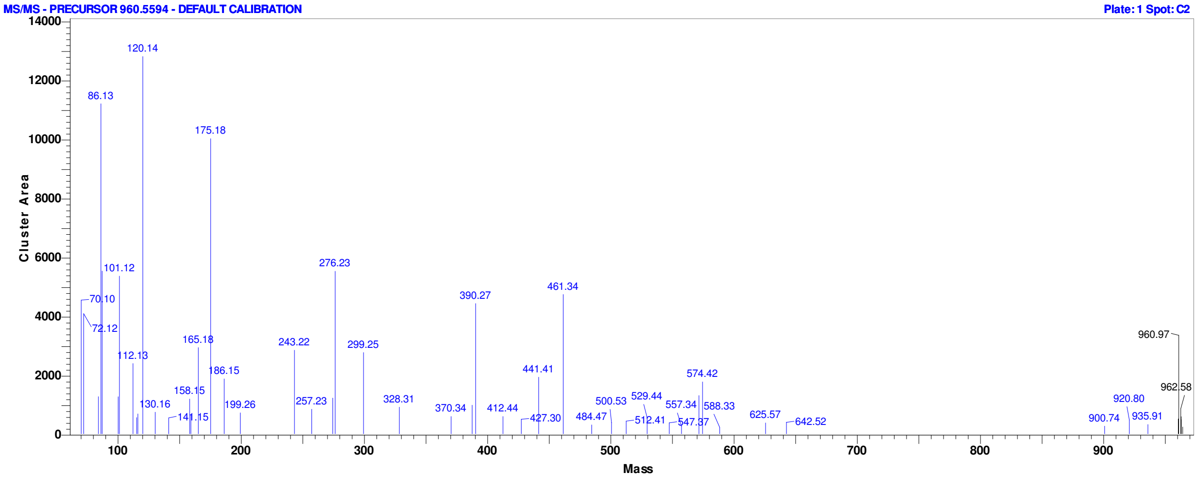

3-hydroxyanthranilate 3,4-dioxygenase

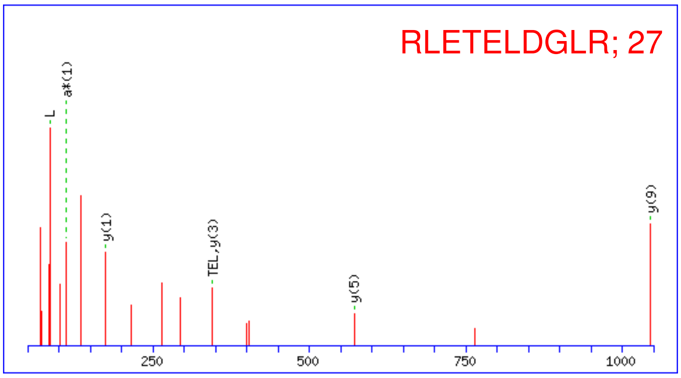

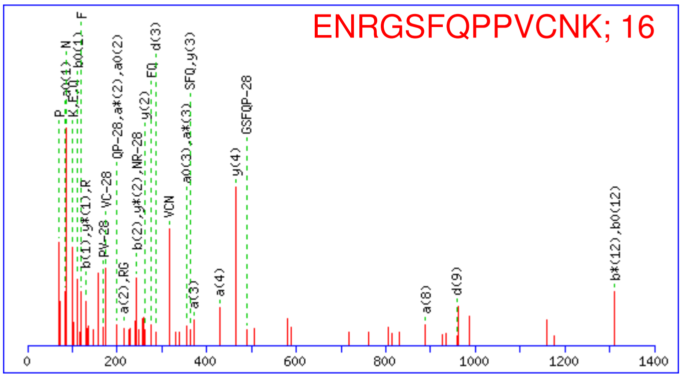

Protein C20orf151

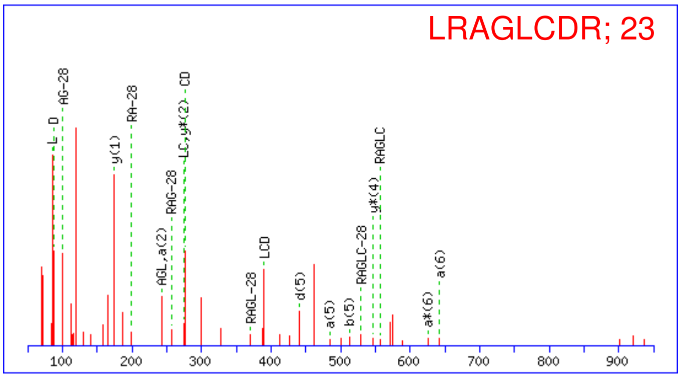

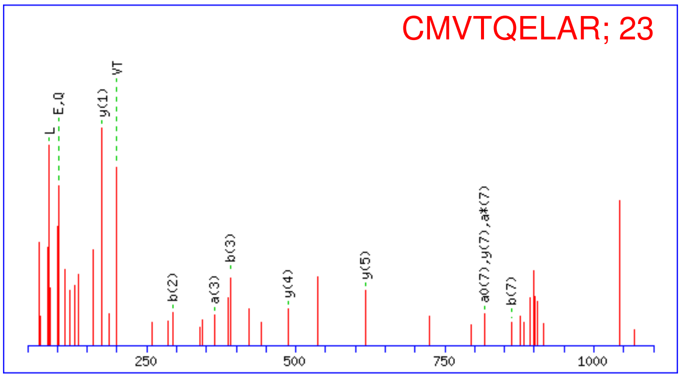

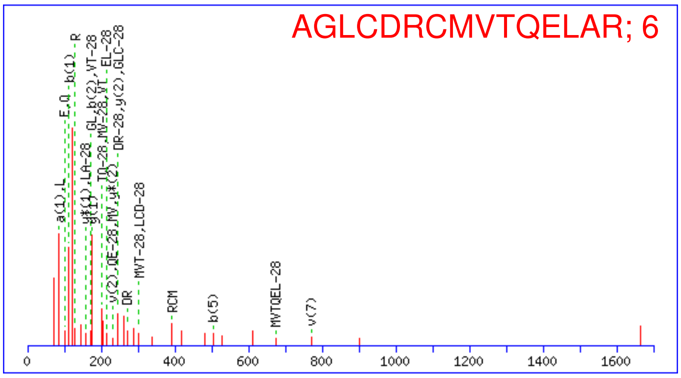

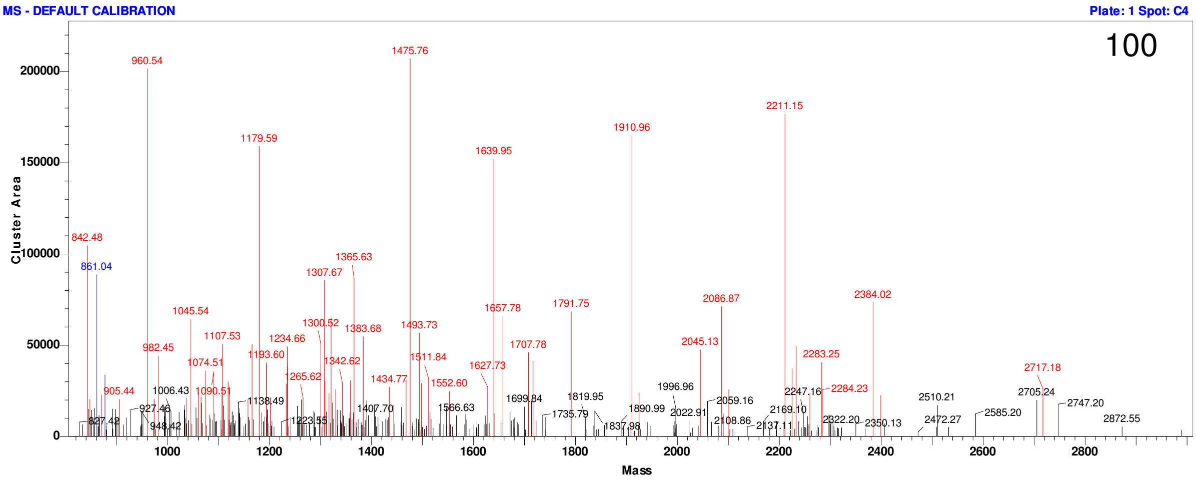
MS - DEFAULT CALIBRATION
Plate: 1 Spot: C4
100
Cluster Area
Mass
960.54
1475.76
2211.15
1910.96
1179.59
1639.95
842.48
861.04
1365.63
1307.67
2384.02
2086.87
1791.75
1657.78
1045.54
1300.52
1493.73
1383.68
1107.53
1234.66
2045.13
1707.78
982.45
1193.60
2283.25
1511.84
1342.62
1074.51
2717.18
1627.73
1265.62
1434.77
1552.60
905.44
1006.43
1090.51
2284.23
2247.16
2705.24
1138.49
1699.84
1819.95
1996.96
2059.16
2510.21
2747.20
927.46
1407.70
1566.63
1890.99
2022.91
2169.10
2585.20
1735.79
2872.55
827.42
1223.55
2108.86
2322.20
2472.27
948.42
1837.98
2137.11
2350.13
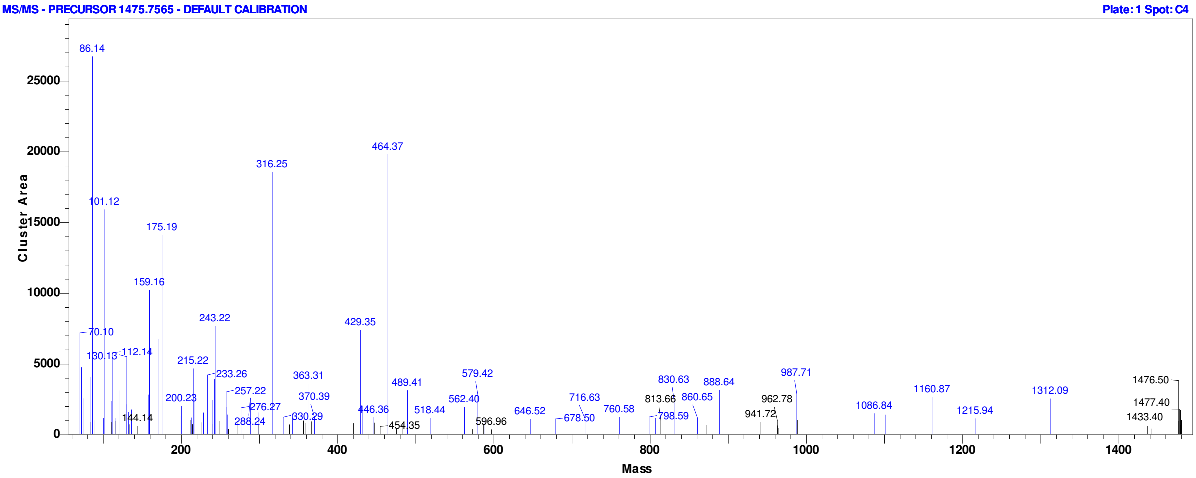
MS/MS - PRECURSOR 1475.7565 - DEFAULT CALIBRATION
Plate: 1 Spot: C4
Cluster Area
Mass
86.14
464.37
316.25
101.12
175.19
159.16
243.22
429.35
70.10
130.13
112.14
215.22
233.26
363.31
489.41
579.42
830.63
888.64
987.71
257.22
370.39
276.27
446.36
518.44
562.40
716.63
813.66
860.65
962.78
1160.87
1312.09
1476.50
200.23
646.52
760.58
1086.84
1215.94
1477.40
144.14
288.24
330.29
454.35
596.96
678.50
798.59
941.72
1433.40
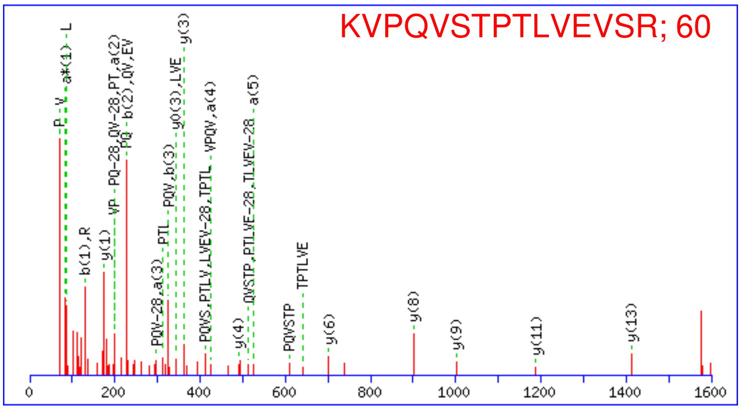
KVPQVSTPTLVEVSR; 60
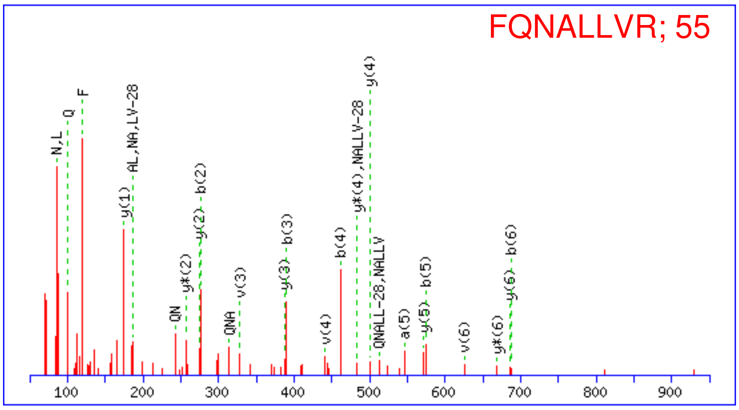
FQNALLVR; 55
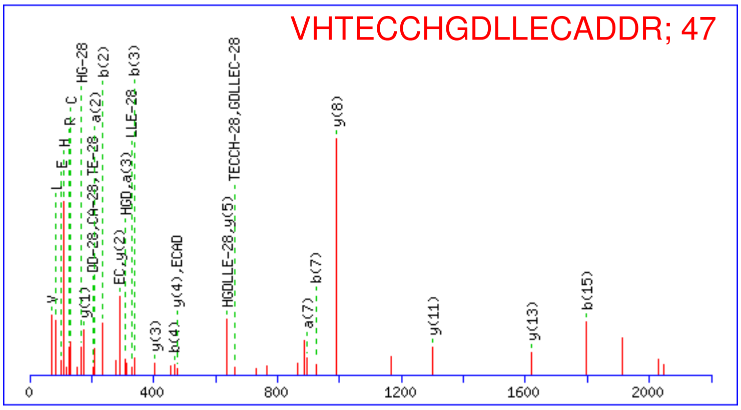
VHTECCHGDLLECADDR; 47

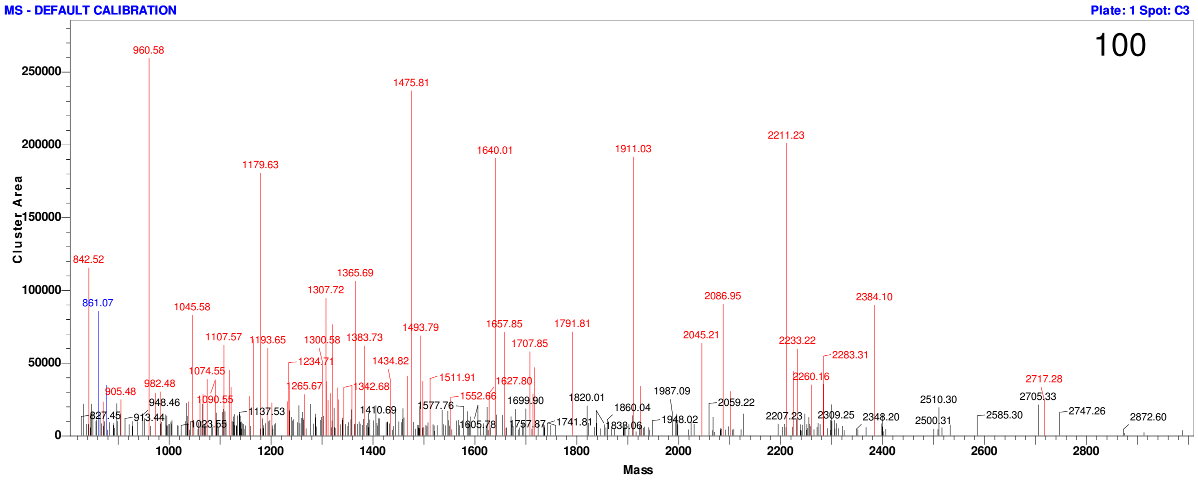
MS - DEFAULT CALIBRATION
Plate: 1 Spot: C3
100
Cluster Area
Mass
960.58
1475.81
2211.23
1179.63
1640.01
1911.03
842.52
861.07
1045.58
1365.69
1307.72
2086.95
2384.10

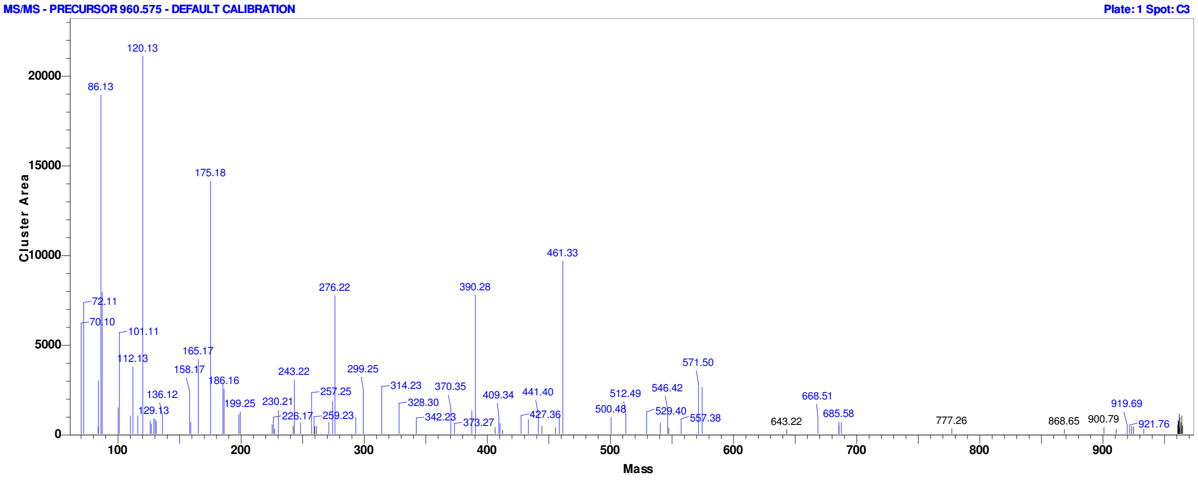
MS/MS - PRECURSOR 960.575 - DEFAULT CALIBRATION
Plate: 1 Spot: C3
Cluster Area
Mass
120.13
86.13
175.18
461.33
276.22
390.28

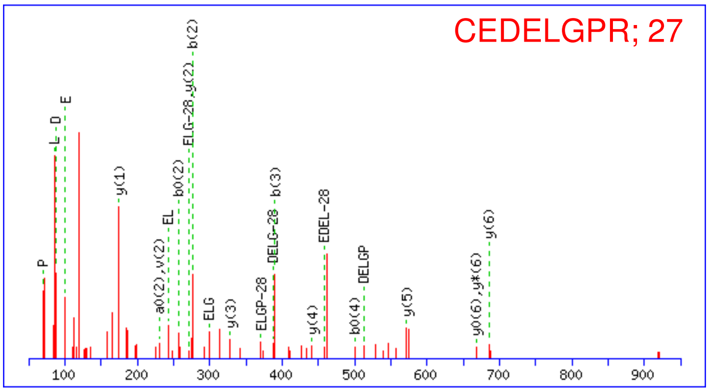
CEDELGPR; 27

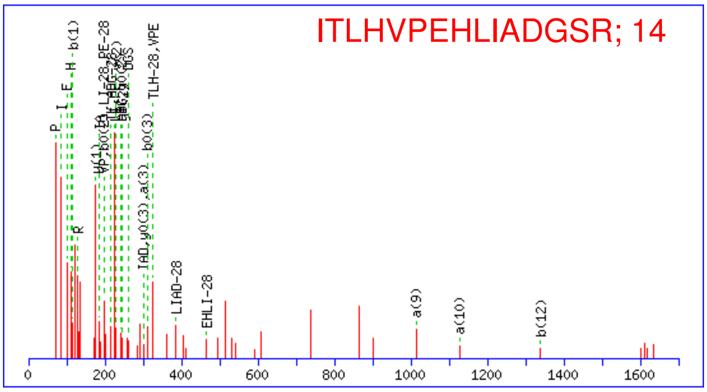
ITLHVPEHLIADGSR; 14

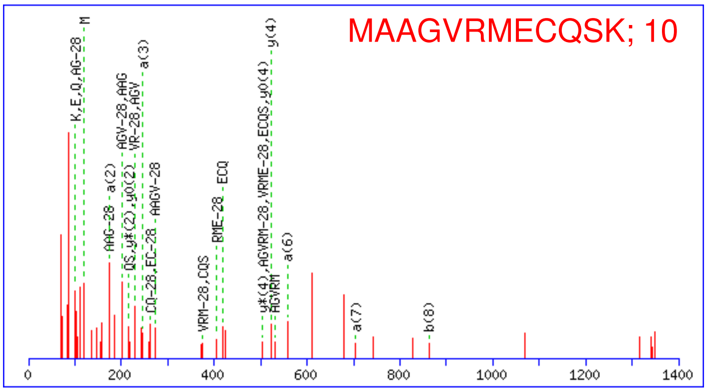
MAAGVRMECQSK; 10

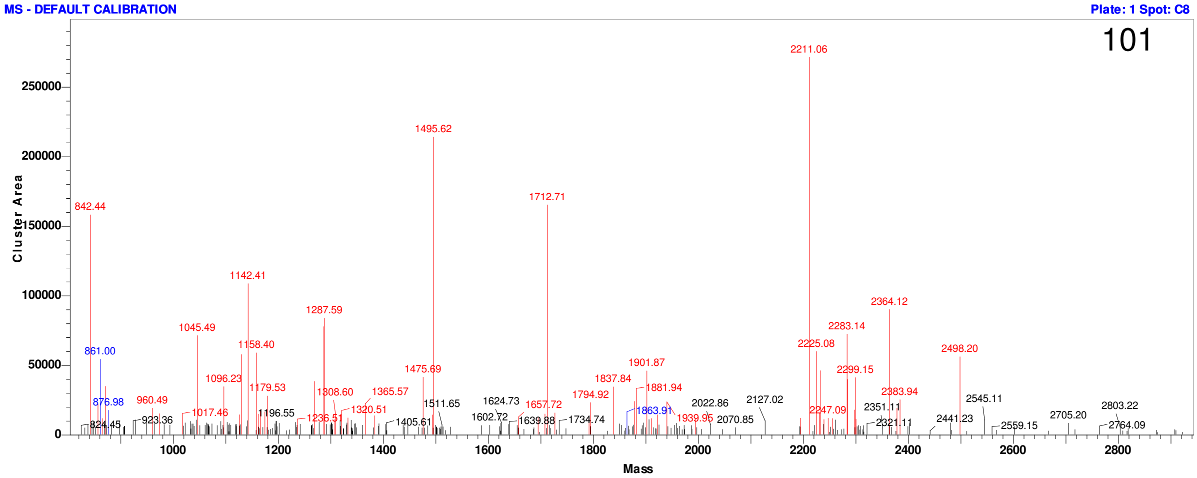

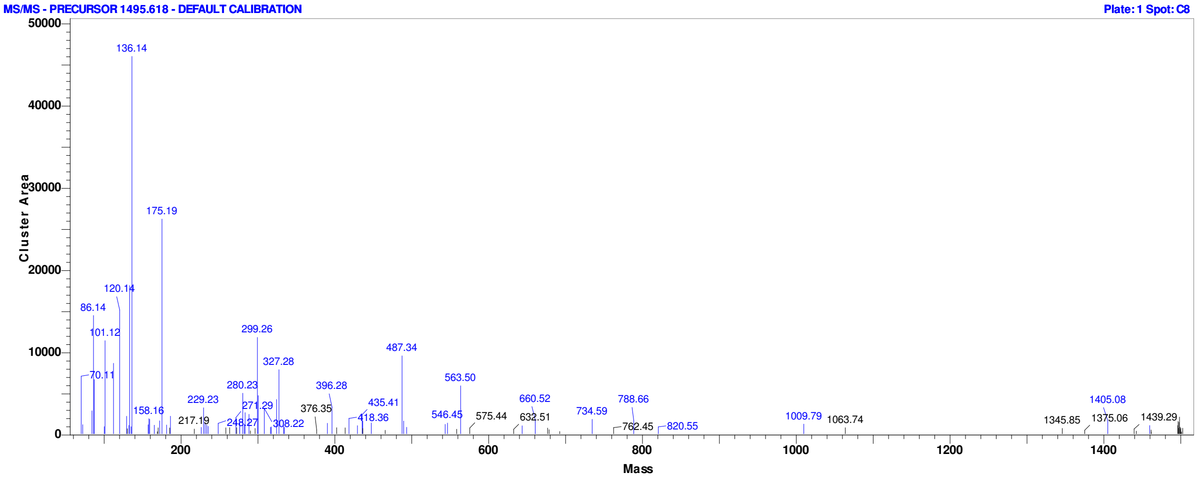

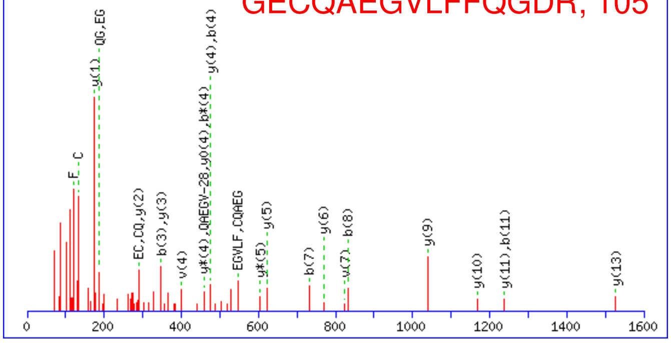

GECQAEGVLFFQGDR; 105

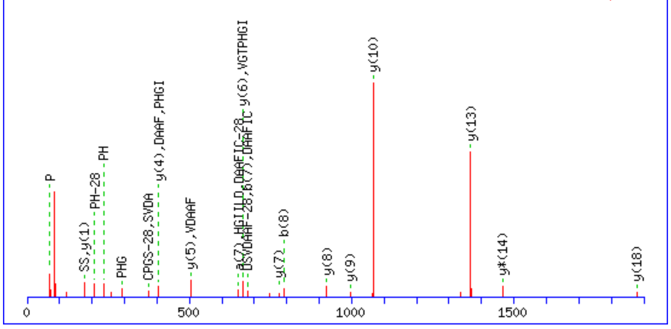

EVGTPHGIILDSVDAAFICPGSSR; 77

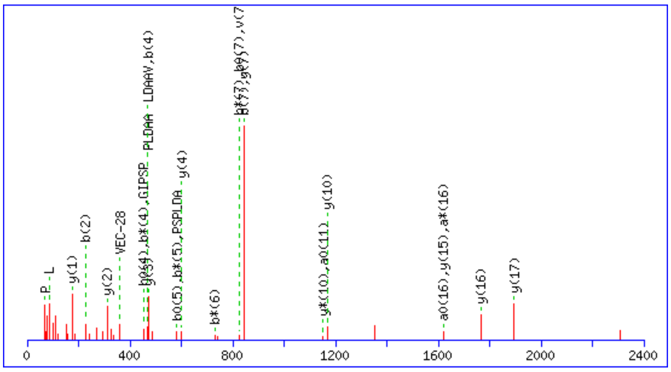

LLQDEFPGIPSPLDAAVECHR; 56

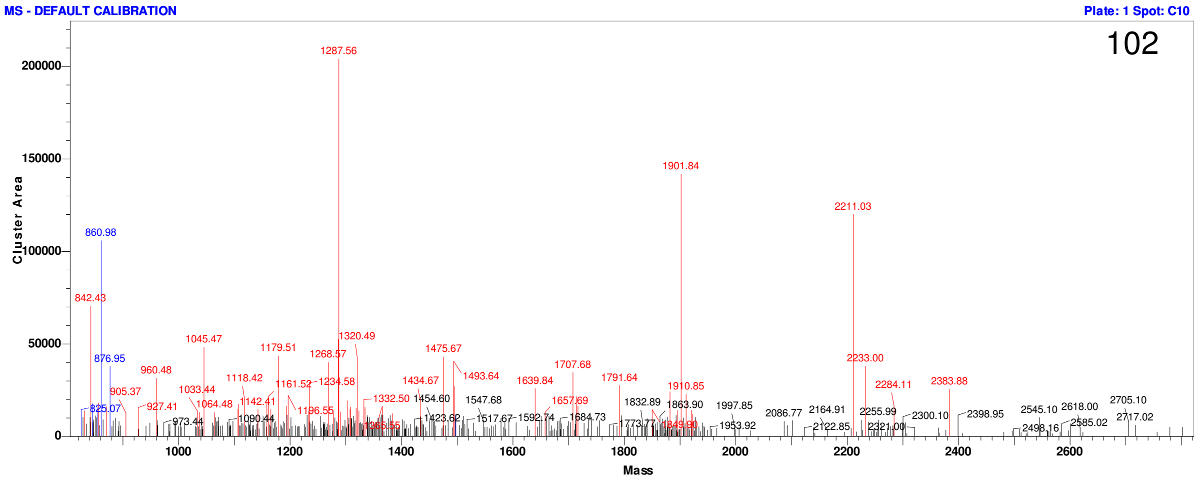
MS - DEFAULT CALIBRATION
Plate: 1 Spot: C10
102
Cluster Area
Mass
1287.56
1901.84
2211.03
860.98
842.43
1045.47
1179.51
1320.49
1475.67
1268.57
1493.64
1707.68
1791.64
2233.00
2284.11
2383.88
2705.10
2618.00
2545.10
2717.02

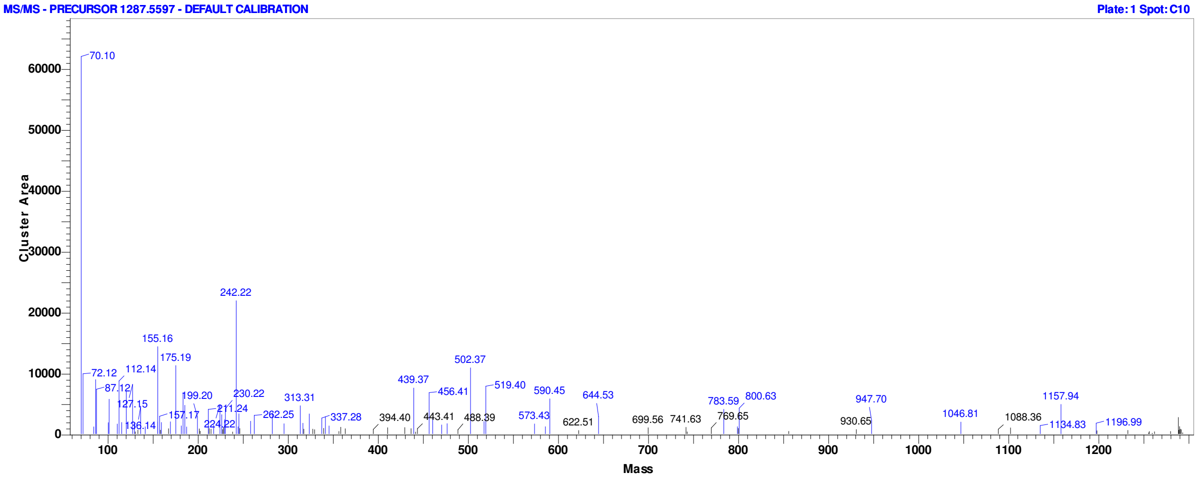
MS/MS - PRECURSOR 1287.5597 - DEFAULT CALIBRATION
Plate: 1 Spot: C10
Cluster Area
Mass
70.10
242.22
155.16
175.19
502.37
439.37
456.41
519.40
590.45
644.53
783.59
800.63
947.70
1046.81
1157.94
1196.99

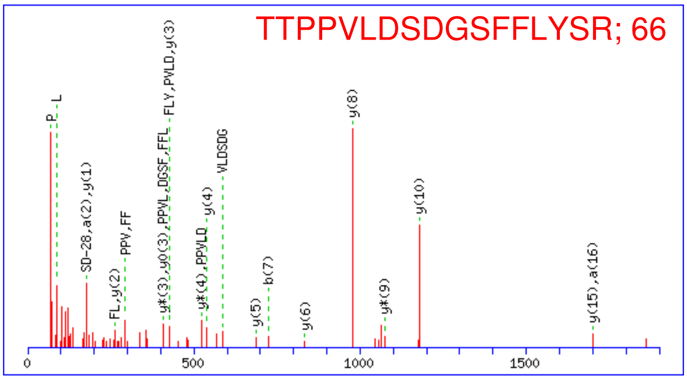
TTPPVLDSDGSFFLYSR; 66

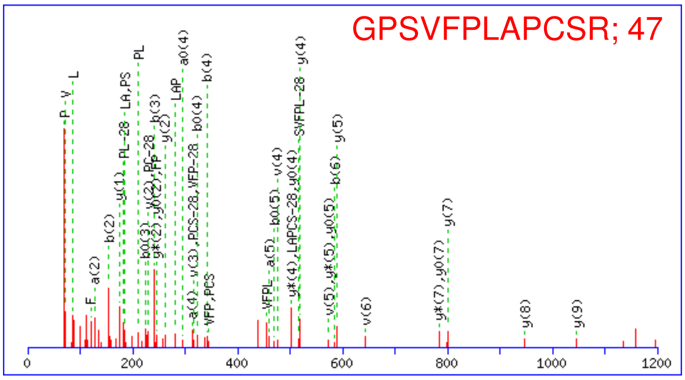
GPSVFPLAPCSR; 47

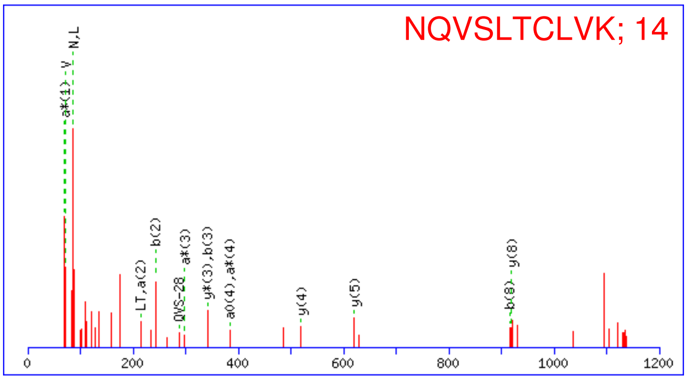
NQVSLTCLVK; 14

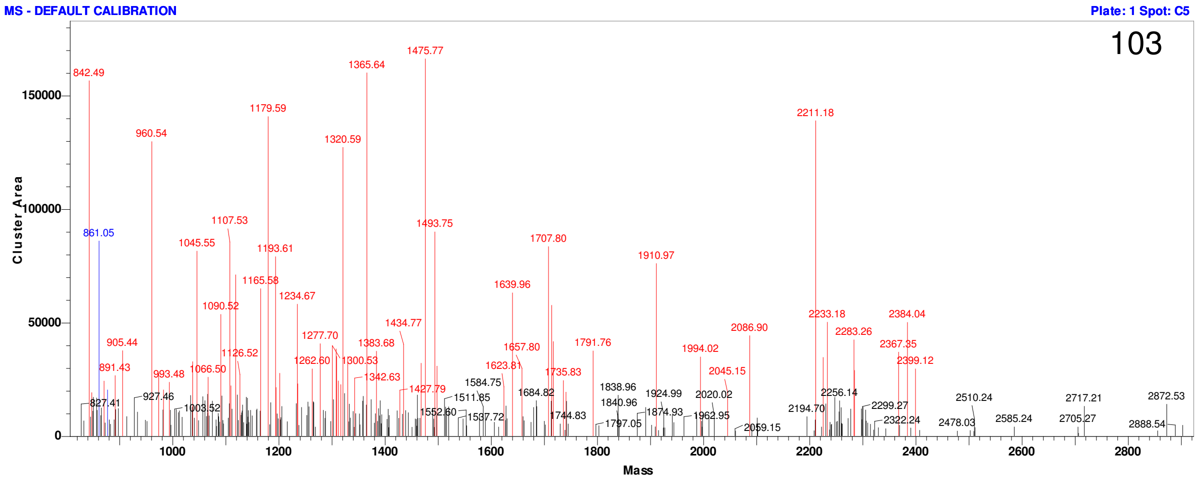
MS - DEFAULT CALIBRATION
Plate: 1 Spot: C5
103
Cluster Area
Mass
842.49
861.05
960.54
1179.59
1365.64
1475.77
1320.59
1107.53
1045.55
1193.61
1493.75
1707.80
1910.97
2211.18
1639.96
1090.52
1165.58
1234.67
1434.77
2086.90
2233.18
2384.04
2283.26
905.44
891.43
993.48
1066.50
1126.52
1277.70
1383.68
1262.60
1300.53
1342.63
1657.80
1623.81
1791.76
1994.02
2367.35
2399.12
2045.15
1735.83
1427.79
1584.75
1511.85
1684.82
1838.96
1840.96
1924.99
2020.02
2256.14
2510.24
2717.21
2872.53
827.41
927.46
1003.52
1552.80
1537.72
1744.83
1797.05
1874.93
1962.95
2059.15
2194.70
2299.27
2322.24
2478.03
2585.24
2705.27
2888.54

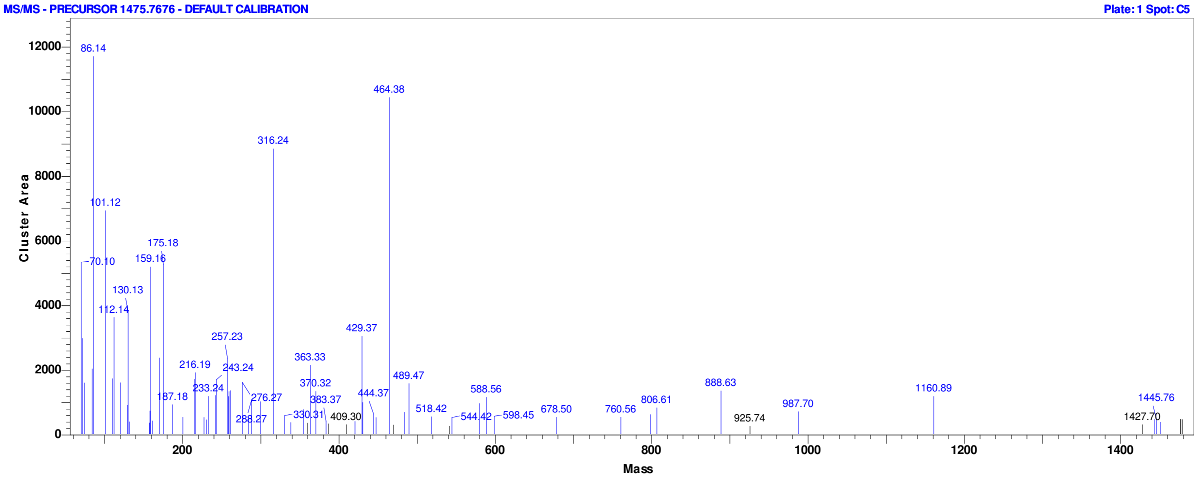
MS/MS - PRECURSOR 1475.7676 - DEFAULT CALIBRATION
Plate: 1 Spot: C5
Cluster Area
Mass
86.14
464.38
316.24
101.12
175.18
159.16
70.10
130.13
112.14
429.37
257.23
363.33
216.19
243.24
489.47
370.32
588.56
233.24
187.18
276.27
383.37
444.37
518.42
330.31
409.30
288.27
544.42
598.45
678.50
760.56
806.61
888.63
925.74
987.70
1160.89
1445.76
1427.70

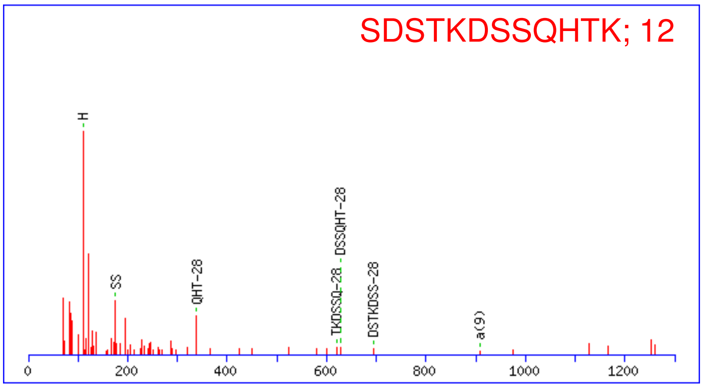
SDSTKDSSQHTK; 12
H
SS
QHT-28
TKDSSQ-28
DSSQHT-28
DSTKDSS-28
a(9)

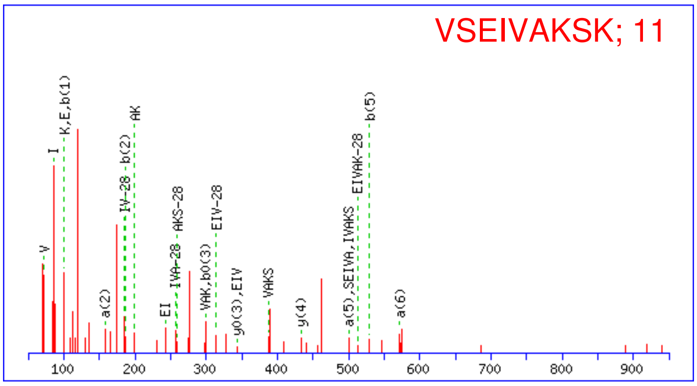
VSEIVAKSK; 11
V
I
K,E,b(1)
a(2)
IV-28
b(2)
AK
EI
IVA-28
AKS-28
VAK,bO(3)
EIV-28
yO(3),EIV
VAKS
y(4)
a(5),SEIVA,IVAKS
EIVAK-28
b(5)
a(6)

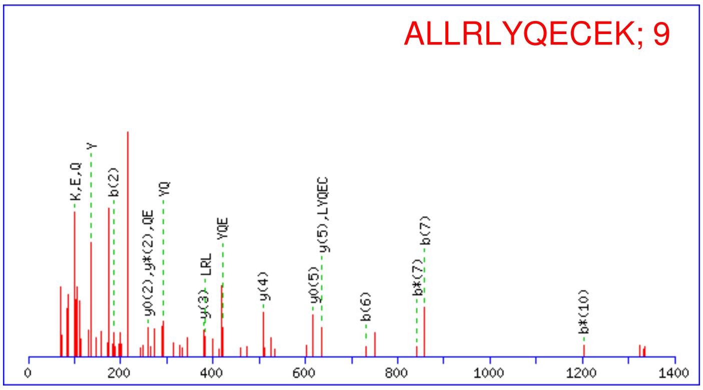
ALLRLYQECEK; 9
K,E,Q
Y
b(2)
yO(2),y*(2),QE
YQ
y(3)
LRL
YQE
y(4)
yO(5)
y(5),LYQEC
b(6)
b*(7)
b(7)
b*(10)

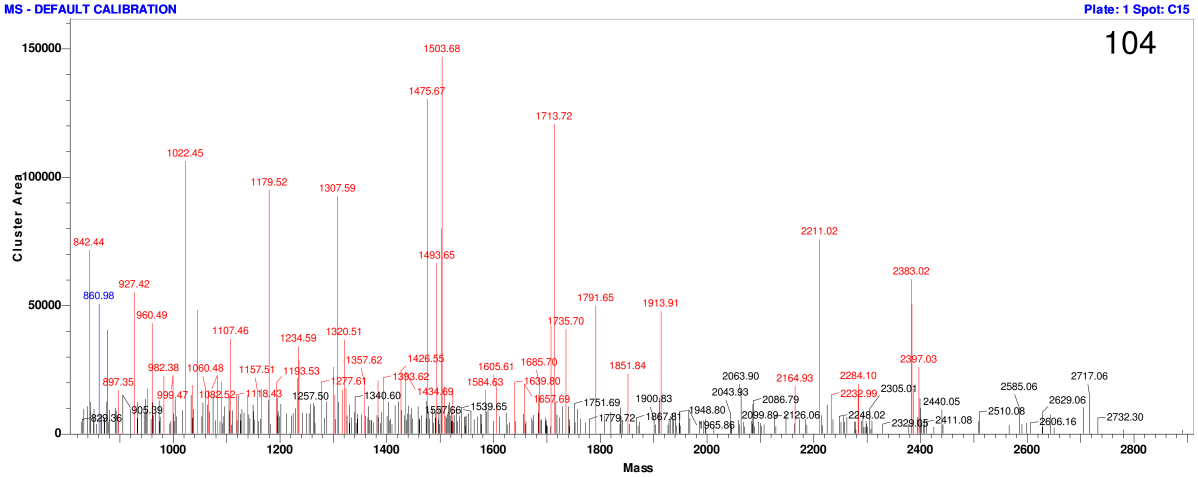

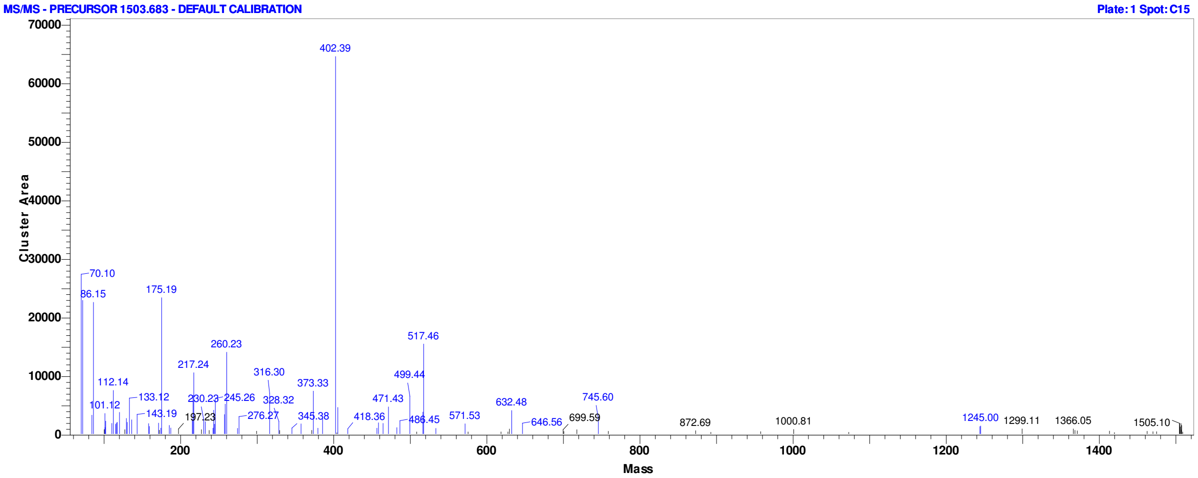

Beta-2-glycoprotein I precursor (Apolipoprotein H)

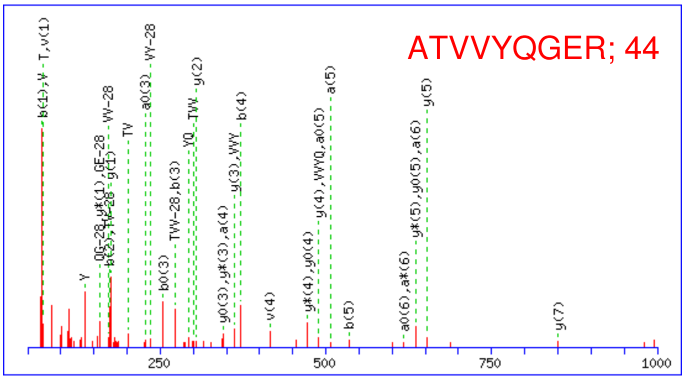

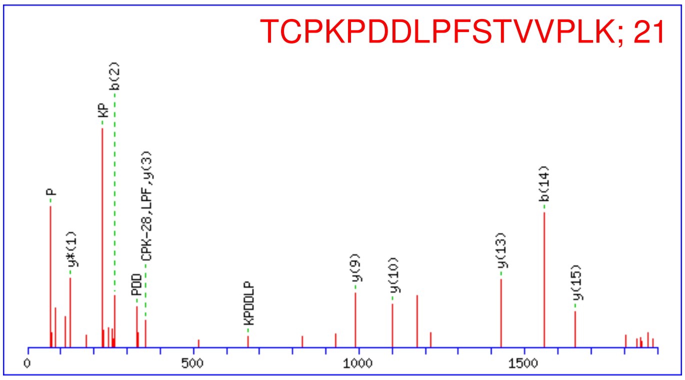

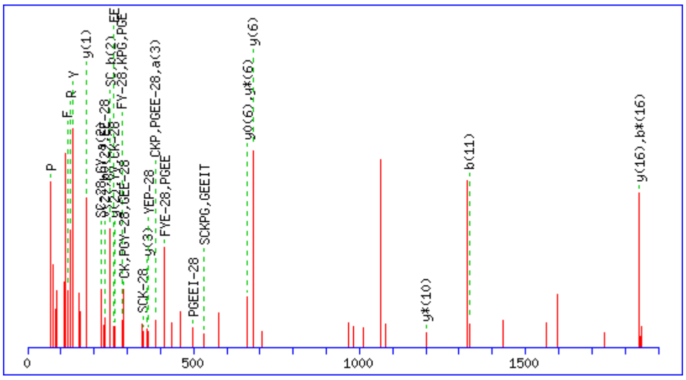

TFYEPGEEITYSCKPGYVSR; 13

General transcription factor 3C polypeptide 5

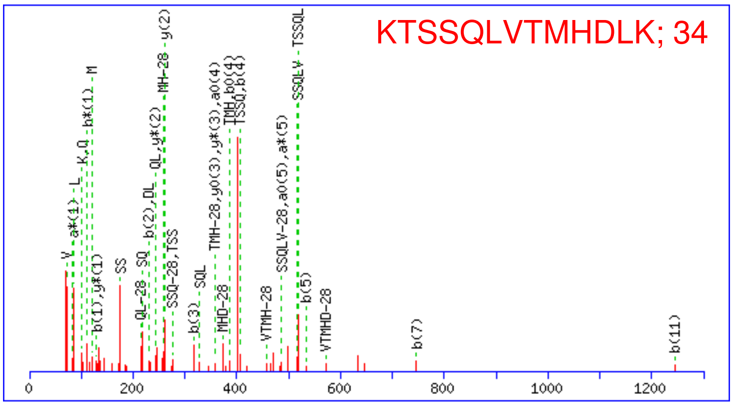

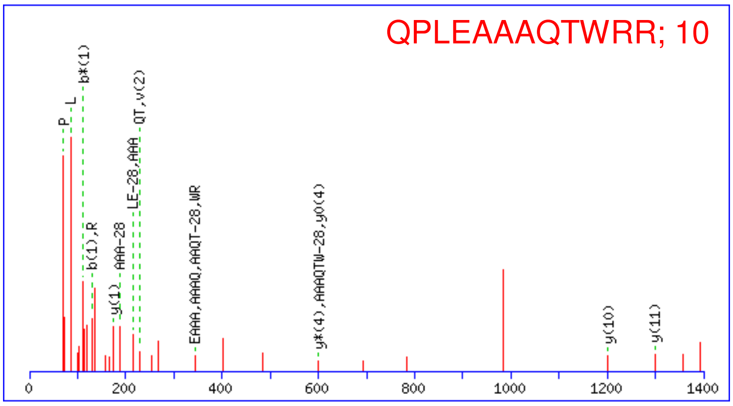

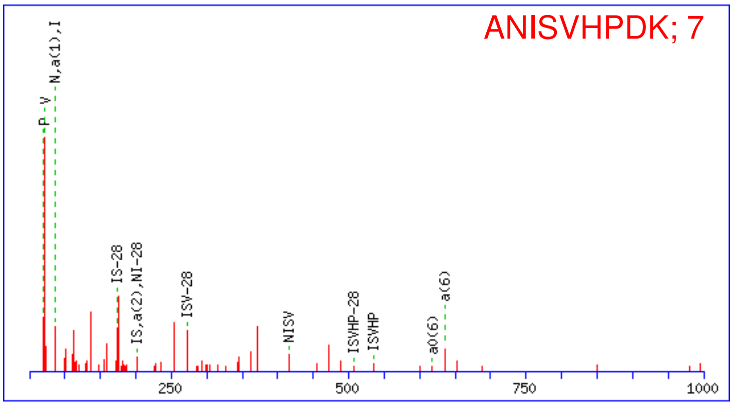

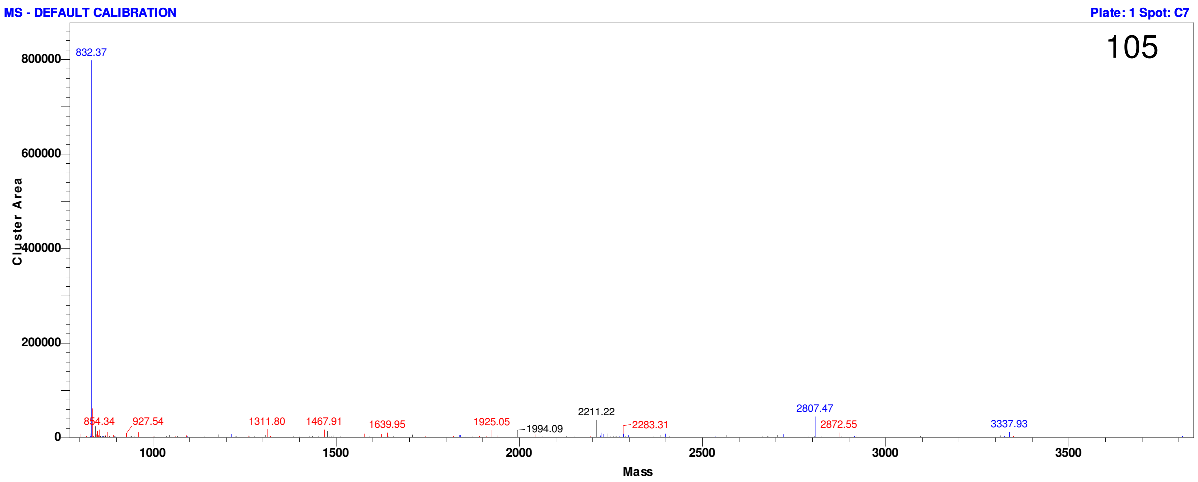
MS - DEFAULT CALIBRATION
Plate: 1 Spot: C7
105
Cluster Area
Mass
832.37
854.34
927.54
1311.80
1467.91
1639.95
1925.05
1994.09
2211.22
2283.31
2807.47
2872.55
3337.93

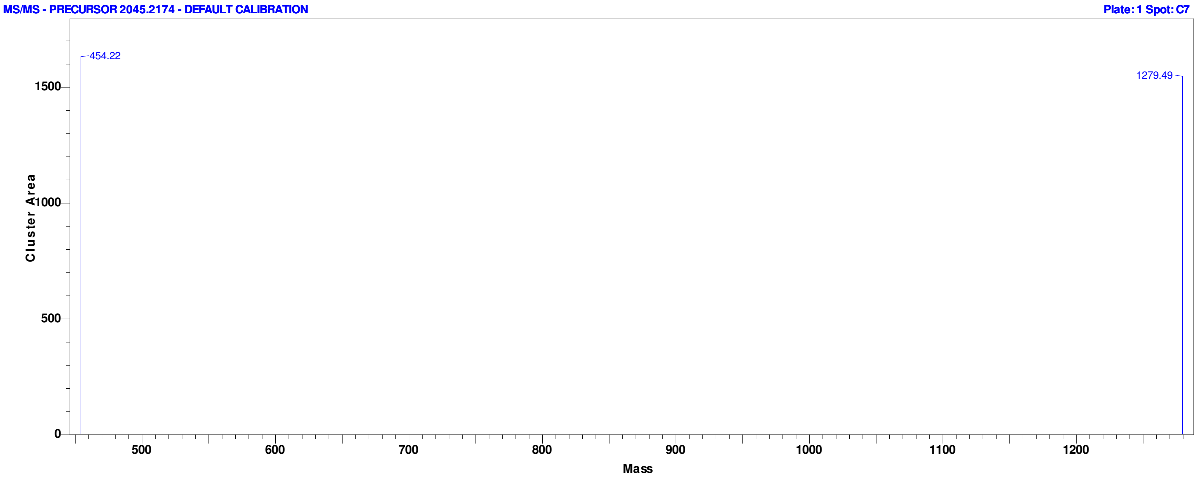
MS/MS - PRECURSOR 2045.2174 - DEFAULT CALIBRATION
Plate: 1 Spot: C7
Cluster Area
Mass
454.22
1279.49

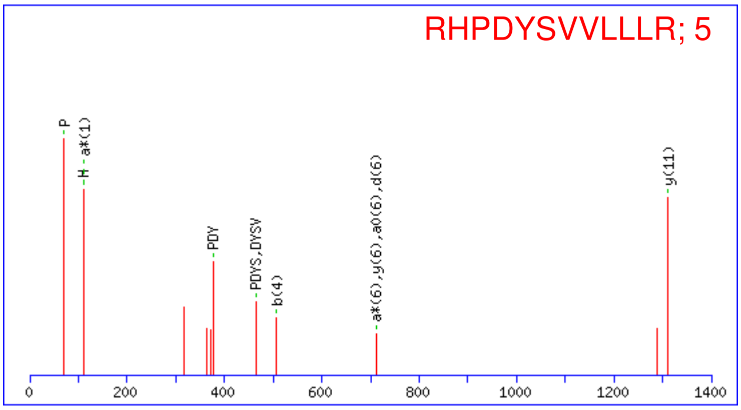
RHPDYSVVLLLR; 5
P
H a*(1)
PDY
PDYS,DYSV
b(4)
a*(6),y(6),a0(6),d(6)
y(11)

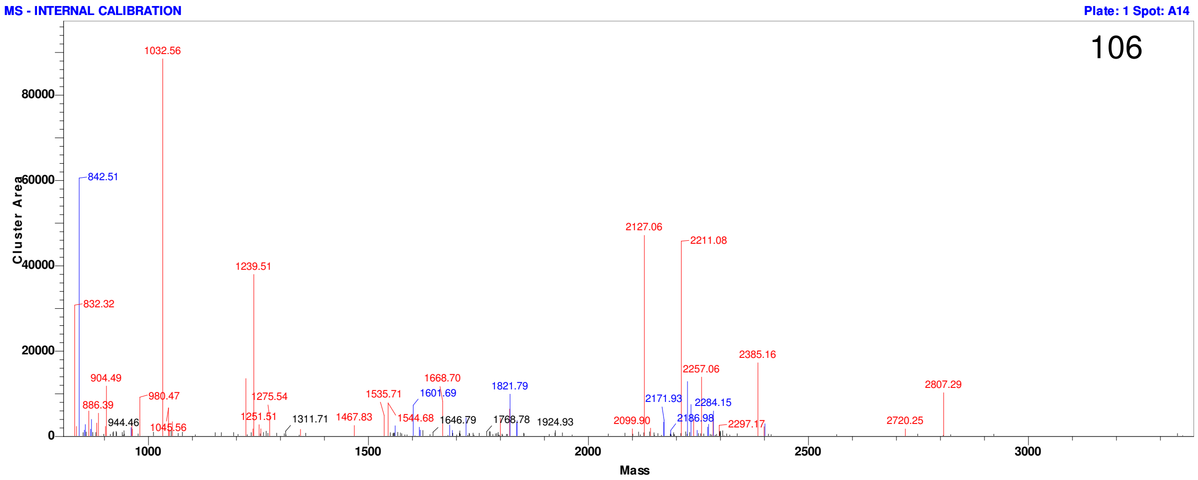

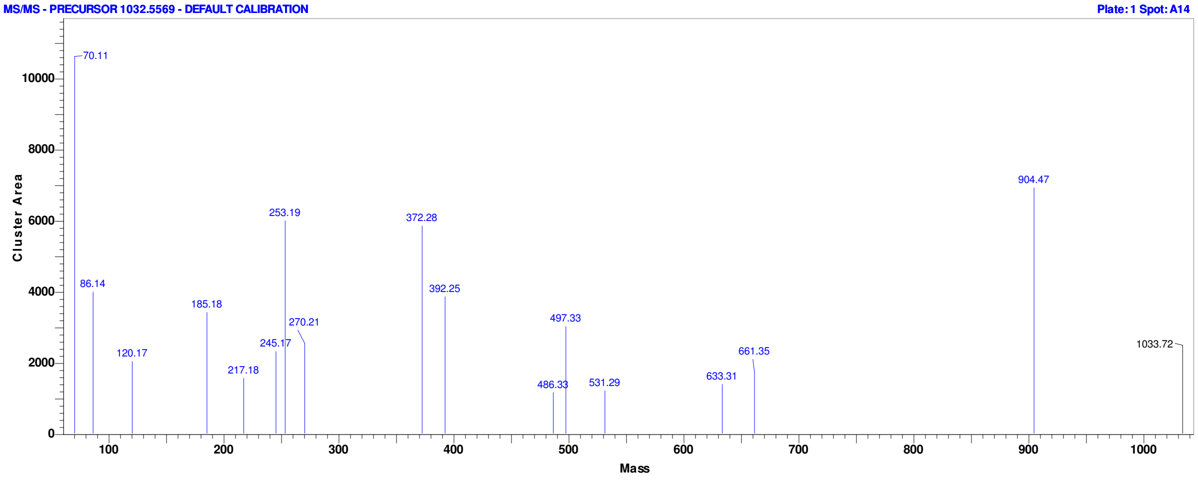

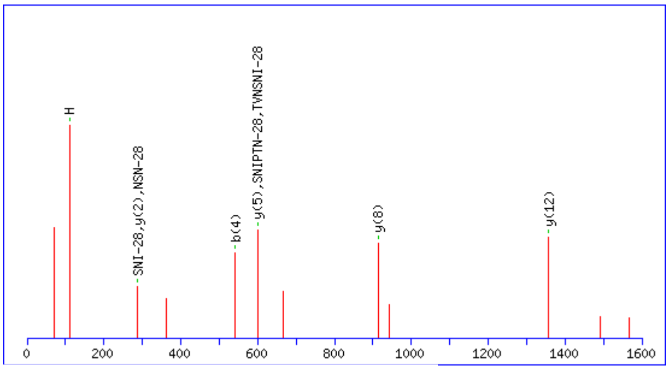

HQLYIDETVNSNIPTNLR; 25

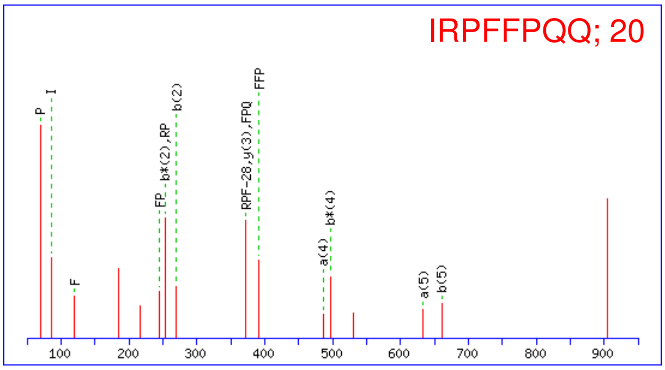

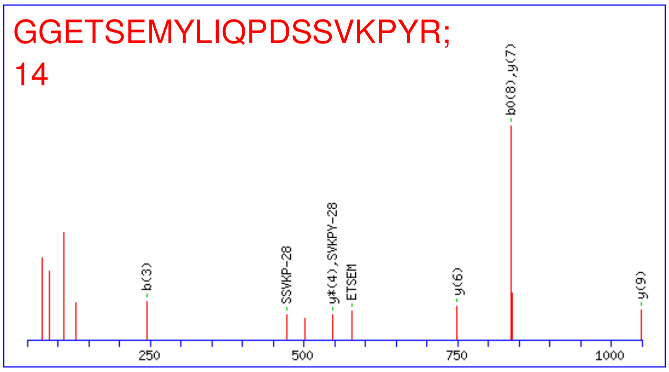

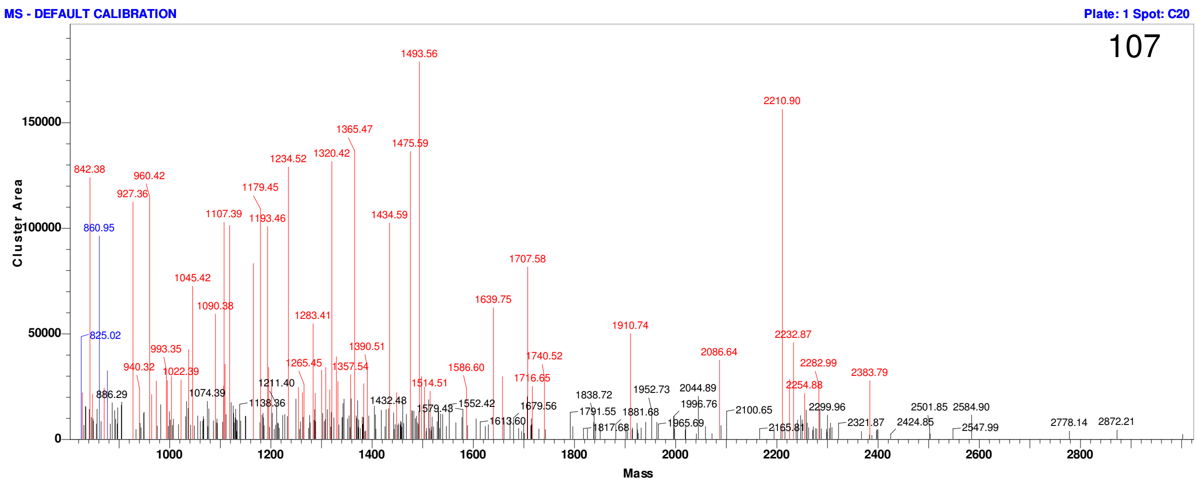
MS - DEFAULT CALIBRATION
Plate: 1 Spot: C20
107
Cluster Area
Mass
1493.56
2210.90
1365.47
1475.59
1234.52
1320.42
842.38
960.42
927.36
1179.45
1107.39
1193.46
1434.59
860.95
1707.58
1045.42
1090.38
1639.75
1283.41
1910.74
825.02
2232.87
1390.51
993.35
2086.64
1740.52
1265.45
2282.99
940.32
1357.54
1022.39
1586.60
2383.79
1716.65
1211.40
1514.51
2254.88
886.29
1074.39
1838.72
1952.73
2044.89
1138.36
1432.48
1552.42
1579.48
1679.56
1996.76
2100.65
2299.96
2501.85
2584.90
1791.55
1881.68
1613.60
1965.69
2321.87
2424.85
2778.14
2872.21
1817.68
2165.81
2547.99

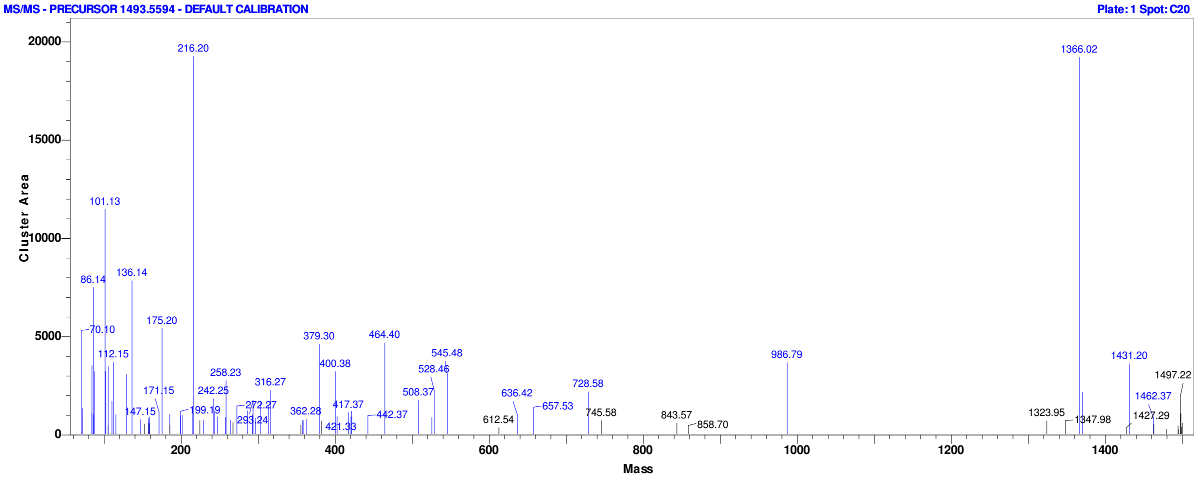
MS/MS - PRECURSOR 1493.5594 - DEFAULT CALIBRATION
Plate: 1 Spot: C20
Cluster Area
Mass
216.20
1366.02
101.13
136.14
86.14
175.20
70.10
379.30
464.40
112.15
545.48
986.79
1431.20
400.38
528.46
258.23
1497.22
316.27
171.15
242.25
508.37
728.58
636.42
1462.37
417.37
272.27
147.15
199.19
362.28
657.53
442.37
745.58
843.57
1323.95
1427.29
293.24
612.54
1347.98
421.33
858.70

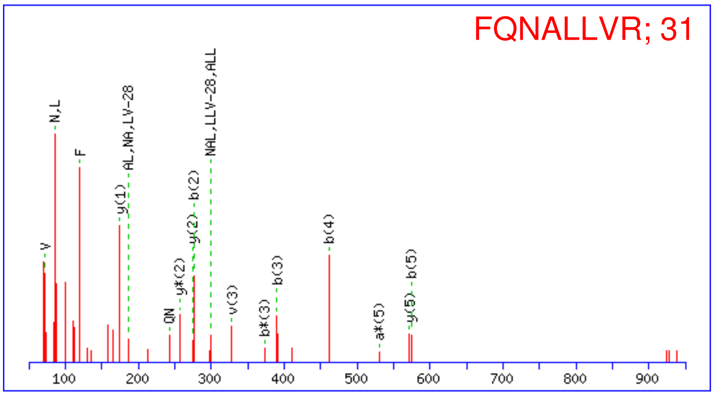
FQNALLVR; 31
V
N,L
F
y(1)
AL,NA,LV-28
QN
y*(2)
y(2)
b(2)
NAL,LLV-28,ALL
v(3)
b*(3)
b(3)
b(4)
a*(5)
y(5)
b(5)

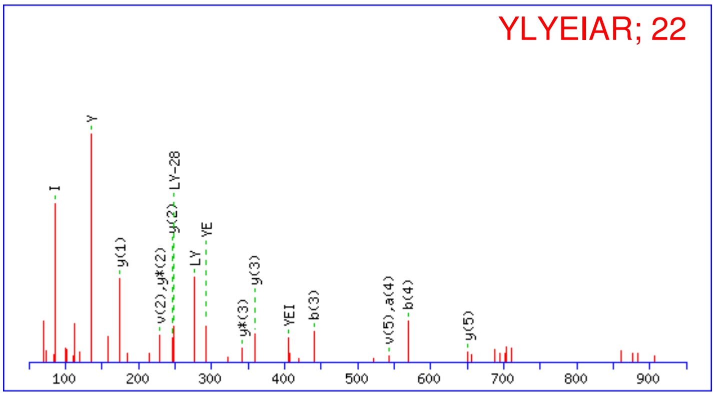
YLYEIAR; 22
I
Y
y(1)
v(2),y*(2)
y(2)
LV-28
LY
YE
y*(3)
y(3)
YEI
b(3)
v(5),a(4)
b(4)
y(5)

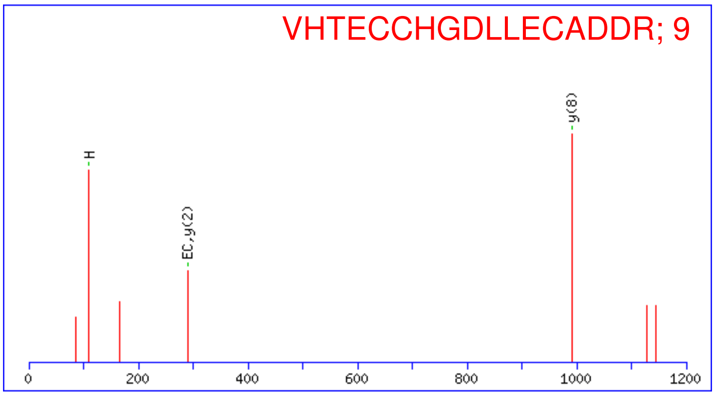
VHTECCHGDLLECADDR; 9
H
EC,y(2)
y(8)

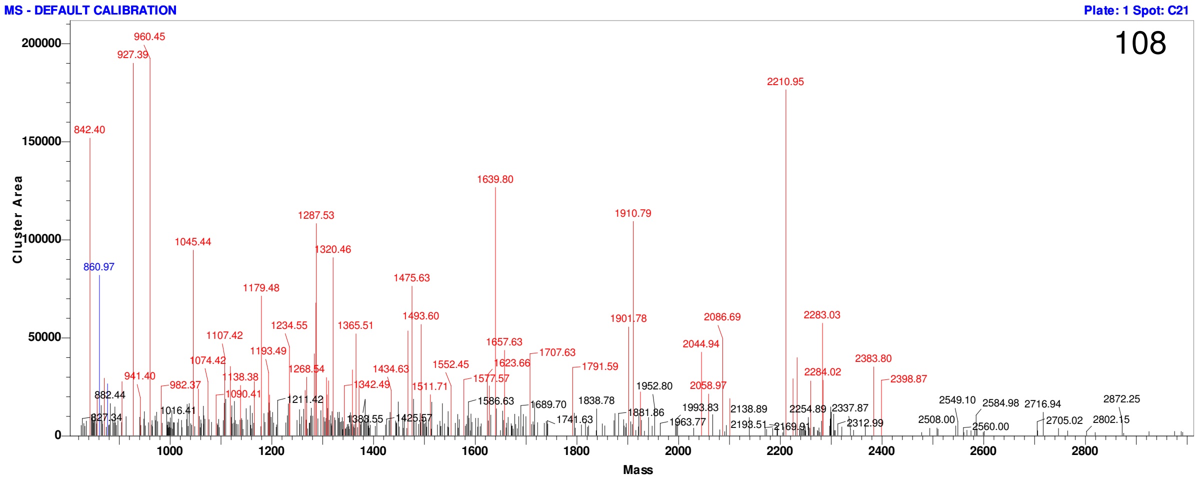
MS - DEFAULT CALIBRATION
Plate: 1 Spot: C21
108
Cluster Area
Mass
960.45
927.39
842.40
860.97
2210.95
1639.80
1910.79
1287.53
1045.44
1320.46
1475.63
1179.48
1493.60
1901.78
2086.69
2283.03
1234.55
1365.51
1107.42
1193.49
1657.63
1707.63
2044.94
1074.42
1268.54
1434.63
1552.45
1623.66
1791.59
2383.80
941.40
982.37
1138.38
1342.49
1577.57
1511.71
2058.97
2284.02
2398.87
882.44
1090.41
1211.42
1586.63
1689.70
1838.78
1952.80
2549.10
2584.98
2716.94
2872.25
1016.41
1993.83
2138.89
2254.89
2337.87
827.34
1383.55
1425.57
1741.63
1881.86
1963.77
2193.51
2169.91
2312.99
2508.00
2560.00
2705.02
2802.15

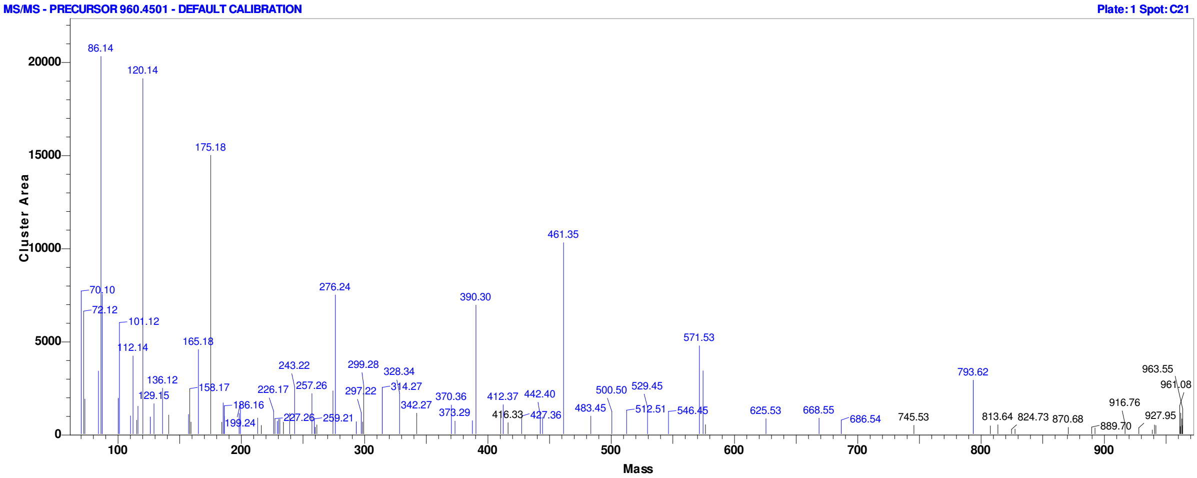
MS/MS - PRECURSOR 960.4501 - DEFAULT CALIBRATION
Plate: 1 Spot: C21
Cluster Area
Mass
86.14
120.14
175.18
461.35
70.10
72.12
101.12
112.14
136.12
129.15
165.18
158.17
186.16
199.24
276.24
390.30
243.22
226.17
257.26
227.26
259.21
299.28
297.22
328.34
314.27
342.27
370.36
373.29
412.37
416.33
442.40
427.36
483.45
500.50
512.51
529.45
546.45
571.53
625.53
668.55
686.54
745.53
793.62
813.64
824.73
870.68
889.70
916.76
927.95
963.55
961.08

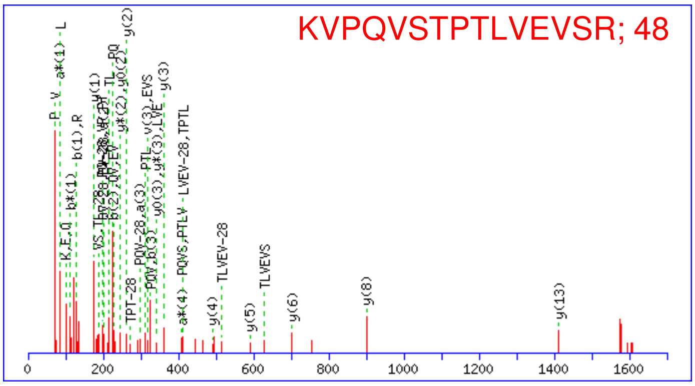
KVPQVSTPTLVEVSR; 48
TLVEV-28
TLVEVS
y(4)
y(5)
y(6)
y(8)
y(13)
a*(4)
y(2)
y(3)

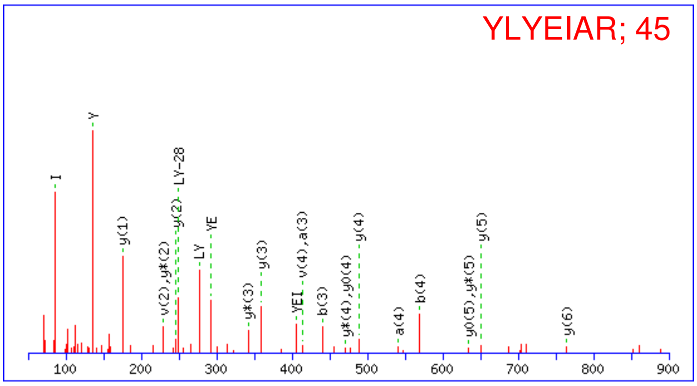
YLYEIAR; 45
I
Y
y(1)
v(2),y*(2)
y(2)
LY-28
LY
YE
y*(3)
y(3)
YEI
v(4),a(3)
b(3)
y*(4),y0(4)
y(4)
a(4)
b(4)
y0(5),y*(5)
y(5)
y(6)

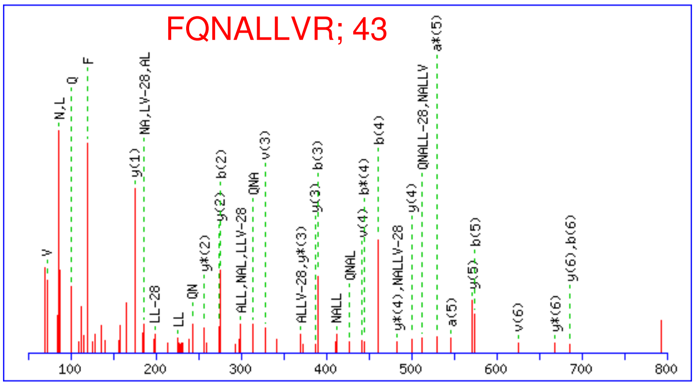
FQNALLVR; 43
V
N,L
Q
F
y(1)
NA,LV-28,AL
LL-28
LL
QN
y*(2)
y(2)
b(2)
ALL,NAL,LLV-28
QNA
v(3)
ALLV-28,y*(3)
y(3)
b(3)
NALL
QNAL
v(4)
b*(4)
y*(4),NALLV-28
b(4)
y(4)
QNALL-28,NALLV
a*(5)
a(5)
y(5)
b(5)
v(6)
y*(6)
y(6),b(6)

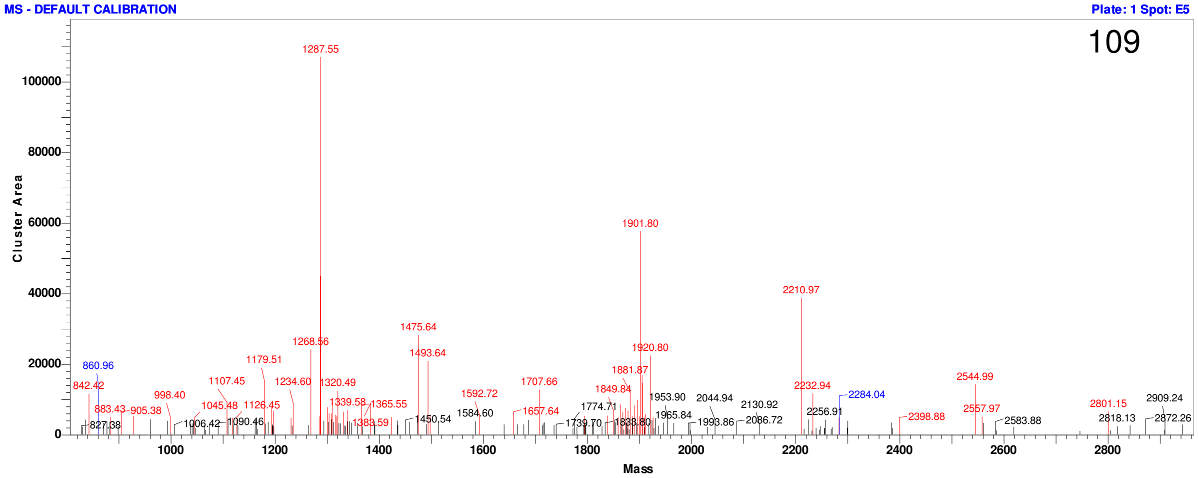
MS - DEFAULT CALIBRATION
Plate: 1 Spot: E5
109
Cluster Area
Mass
1287.55
1901.80
2210.97
1475.64
1268.56
1493.64
1920.80
860.96
1179.51
842.42
1881.87
1107.45
1234.60
1320.49
1707.66
2544.99
1849.84
2232.94
2284.04
998.40
1592.72
1953.90
2044.94
2909.24
2130.92
1339.58
1365.55
883.43
905.38
1045.48
1126.45
1774.71
2801.15
1584.60
1657.64
2256.91
2557.97
1450.54
1965.84
2398.88
2818.13
2872.26
827.38
1006.42
1090.46
1383.59
1739.70
1833.80
1993.86
2086.72
2583.88

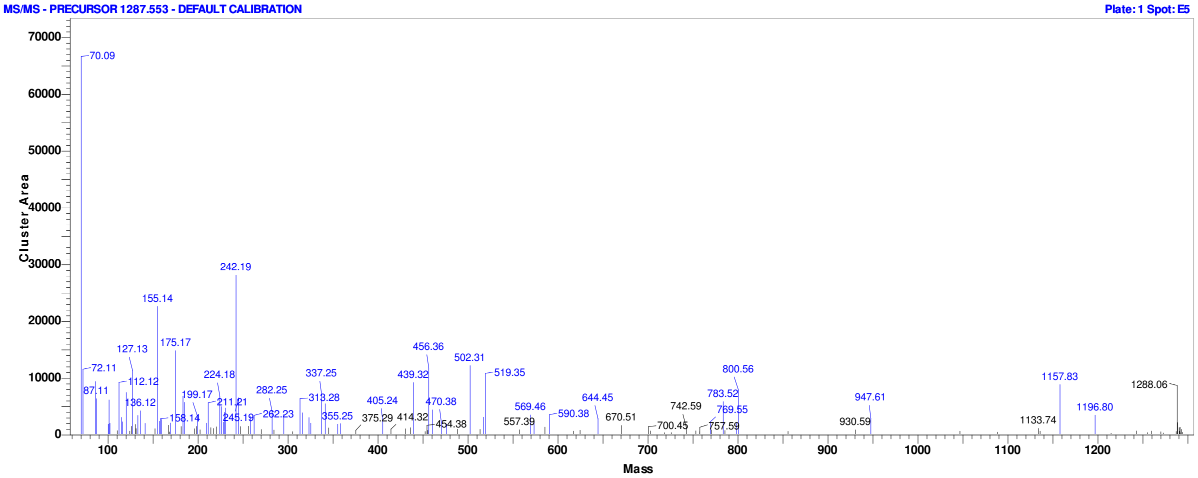
MS/MS - PRECURSOR 1287.553 - DEFAULT CALIBRATION
Plate: 1 Spot: E5
Cluster Area
Mass
70.09
242.19
155.14
127.13
175.17
456.36
502.31
72.11
224.18
337.25
439.32
519.35
800.56
1157.83
112.12
87.11
282.25
313.28
199.17
405.24
470.38
783.52
569.46
644.45
947.61
1288.06
136.12
211.21
742.59
769.55
1196.80
158.14
245.19
262.23
355.25
375.29
414.32
454.38
557.39
590.38
670.51
700.45
757.59
930.59
1133.74

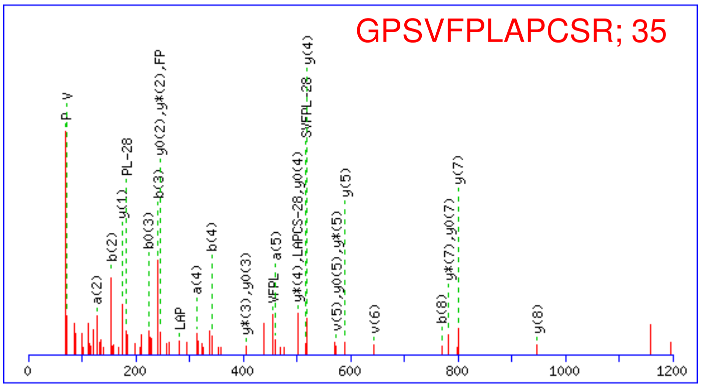
GPSVFPLAPCSR; 35

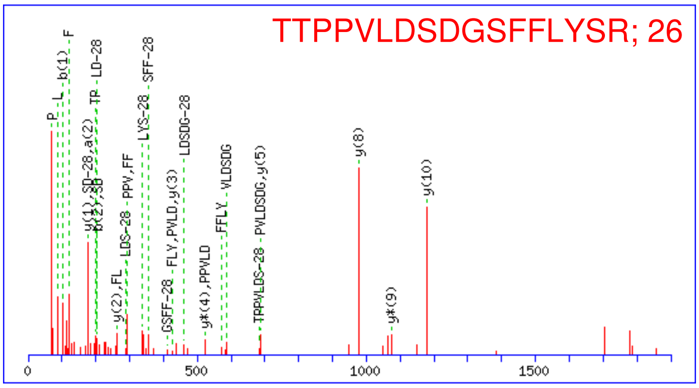
TTPPVLDSDGSFFLYSR; 26

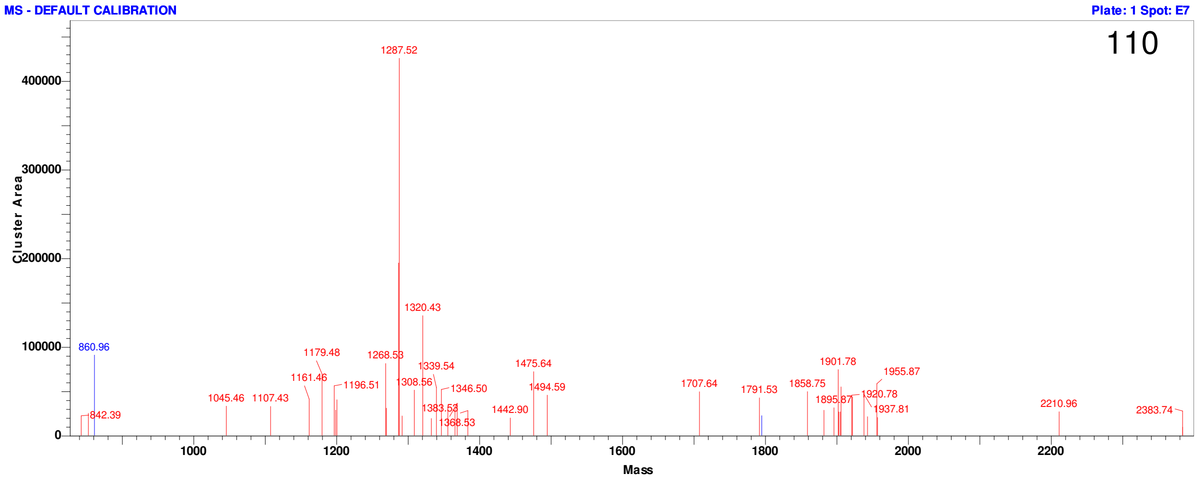

MS - DEFAULT CALIBRATION
Plate: 1 Spot: E7
110
Cluster Area
Mass
1287.52
1320.43
860.96
842.39
1045.46
1107.43
1161.46
1179.48
1196.51
1268.53
1308.56
1339.54
1346.50
1383.53
1368.53
1442.90
1475.64
1494.59
1707.64
1791.53
1858.75
1895.87
1901.78
1920.78
1937.81
1955.87
2210.96
2383.74

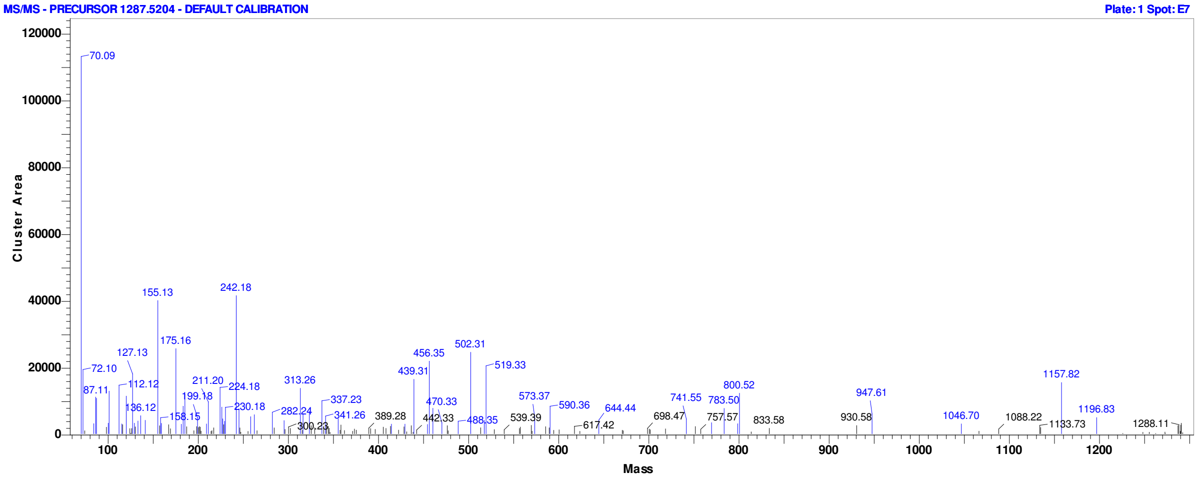

MS/MS - PRECURSOR 1287.5204 - DEFAULT CALIBRATION
Plate: 1 Spot: E7
Cluster Area
Mass
70.09
72.10
87.11
112.12
127.13
136.12
155.13
158.15
175.16
199.18
211.20
224.18
230.18
242.18
282.24
300.23
313.26
337.23
341.26
389.28
439.31
442.33
456.35
470.33
488.35
502.31
519.33
539.39
573.37
590.36
617.42
644.44
698.47
741.55
757.57
783.50
800.52
833.58
930.58
947.61
1046.70
1088.22
1133.73
1157.82
1196.83
1288.11

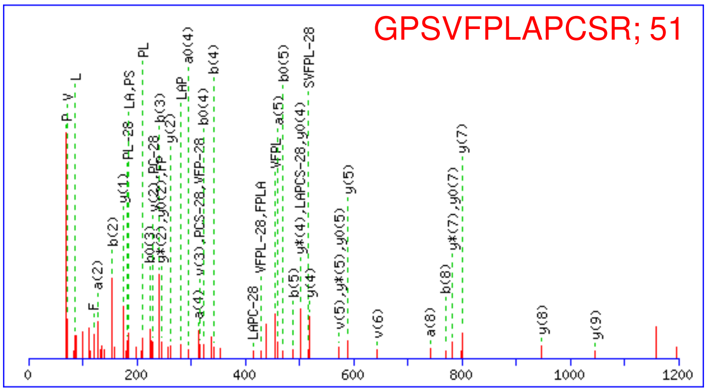

GPSVFPLAPCSR; 51

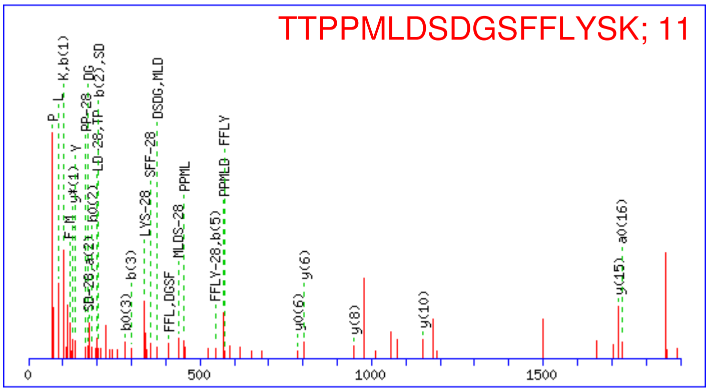

TTPPMLDSDGSFFLYSK; 11

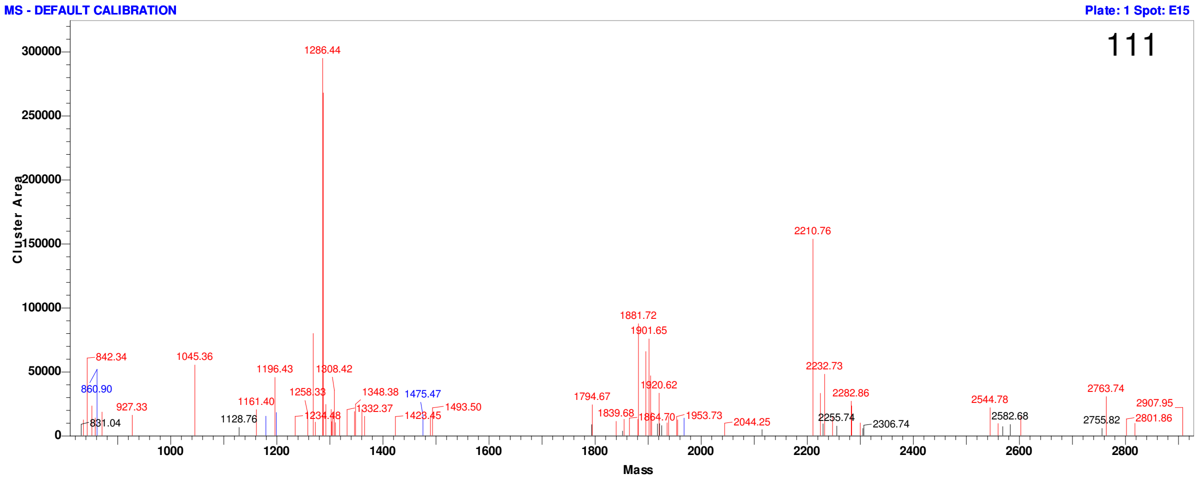
MS - DEFAULT CALIBRATION
Plate: 1 Spot: E15
111
Cluster Area
Mass
1286.44
2210.76
1881.72
1901.65

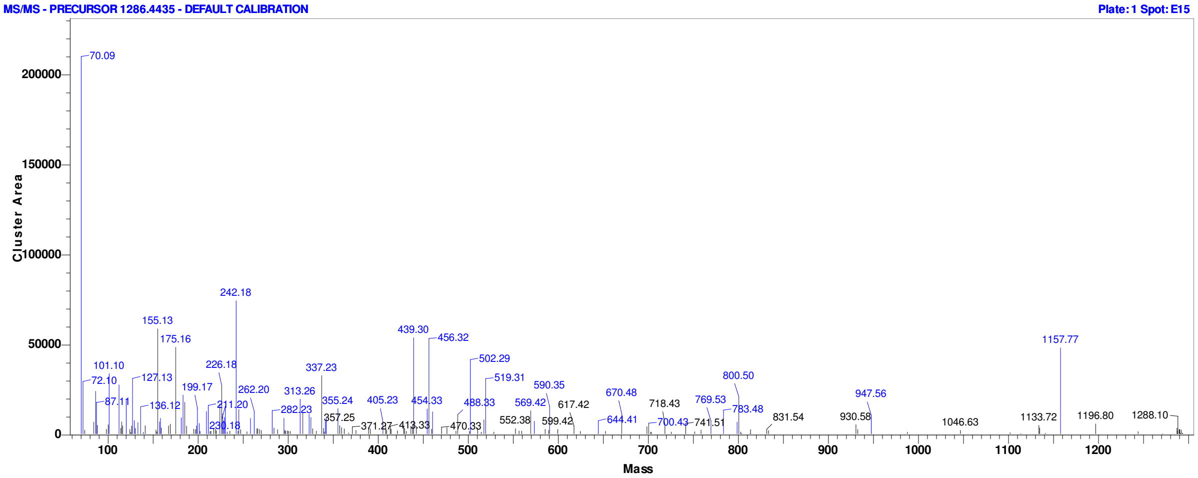
MS/MS - PRECURSOR 1286.4435 - DEFAULT CALIBRATION
Plate: 1 Spot: E15
Cluster Area
Mass
70.09
242.18
1157.77

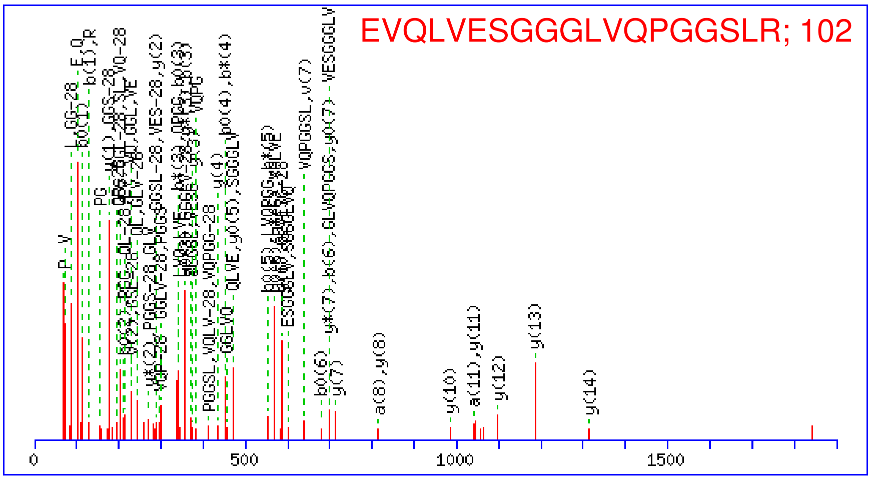
EVQLVESGGGLVQPGGSLR; 102

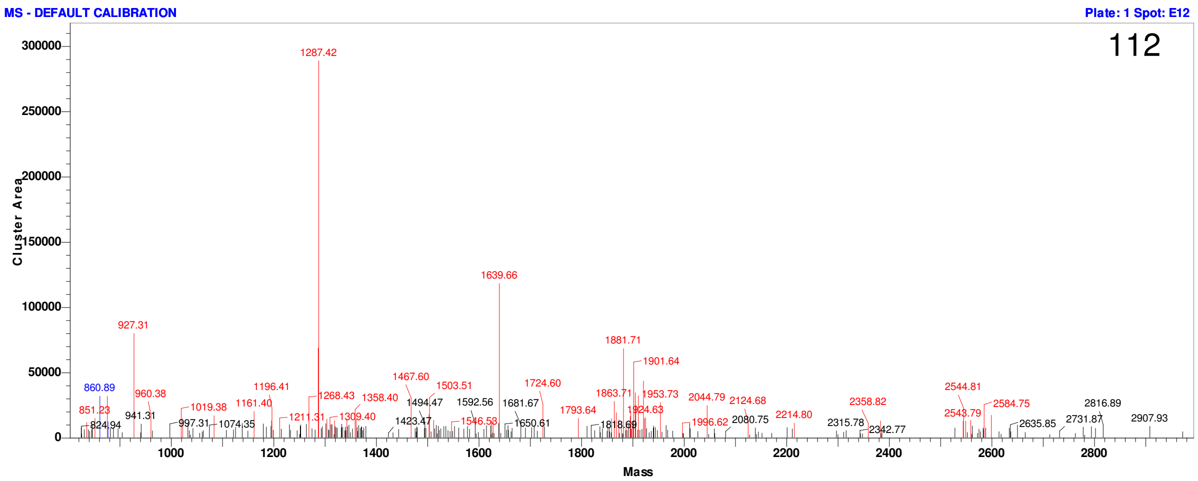
MS - DEFAULT CALIBRATION
Plate: 1 Spot: E12
112
Cluster Area
Mass
1287.42
1639.66
927.31
1881.71
1901.64
860.89
960.38
851.23
824.94
941.31
997.31
1019.38
1074.35
1161.40
1196.41
1211.31
1268.43
1309.40
1358.40
1423.47
1467.60
1494.47
1503.51
1546.53
1592.56
1650.61
1681.67
1724.60
1793.64
1818.69
1863.71
1924.63
1953.73
1996.62
2044.79
2080.75
2124.68
2214.80
2315.78
2342.77
2358.82
2543.79
2544.81
2584.75
2635.85
2731.87
2816.89
2907.93

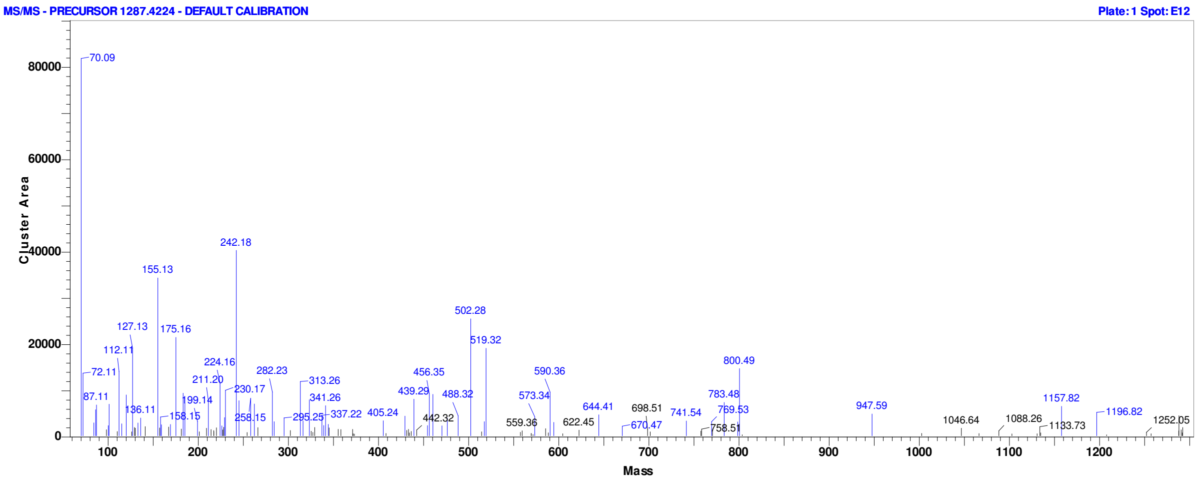
MS/MS - PRECURSOR 1287.4224 - DEFAULT CALIBRATION
Plate: 1 Spot: E12
Cluster Area
Mass
70.09
72.11
87.11
112.11
127.13
136.11
155.13
158.15
175.16
199.14
211.20
224.16
230.17
242.18
258.15
282.23
295.25
313.26
337.22
341.26
405.24
439.29
442.32
456.35
488.32
502.28
519.32
559.36
573.34
590.36
622.45
644.41
670.47
698.51
741.54
758.51
769.53
783.48
800.49
947.59
1046.64
1088.26
1133.73
1157.82
1196.82
1252.05

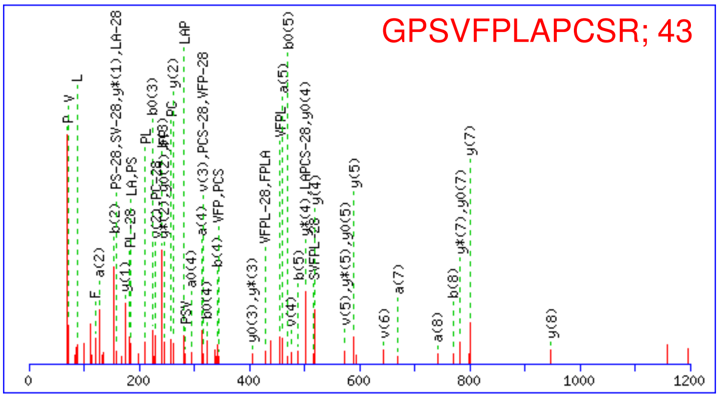
GPSVFPLAPCSR; 43

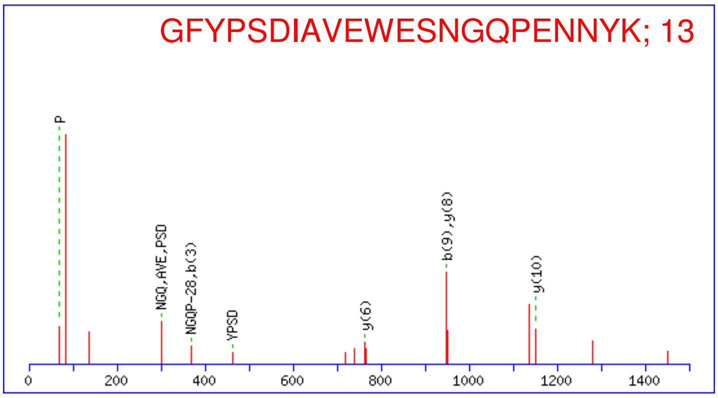
GFYPSDIAVEWESNGQPENNYK; 13

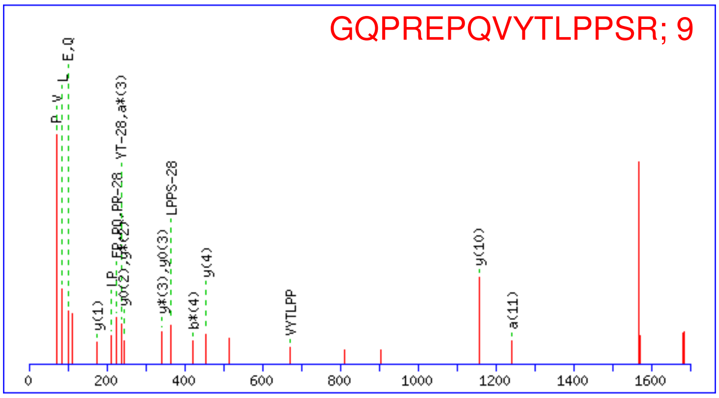
GQPREPQVYTLPPSR; 9

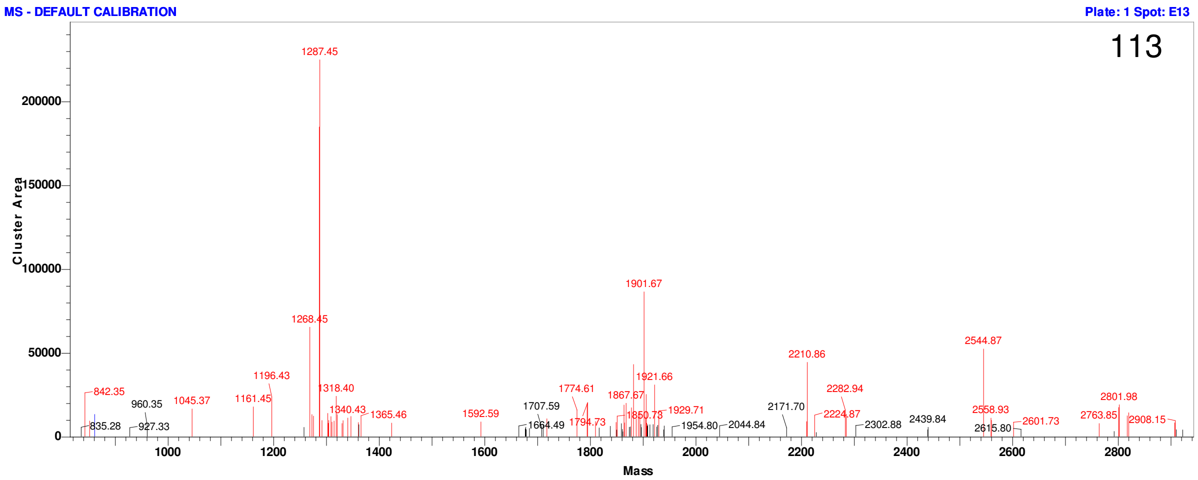

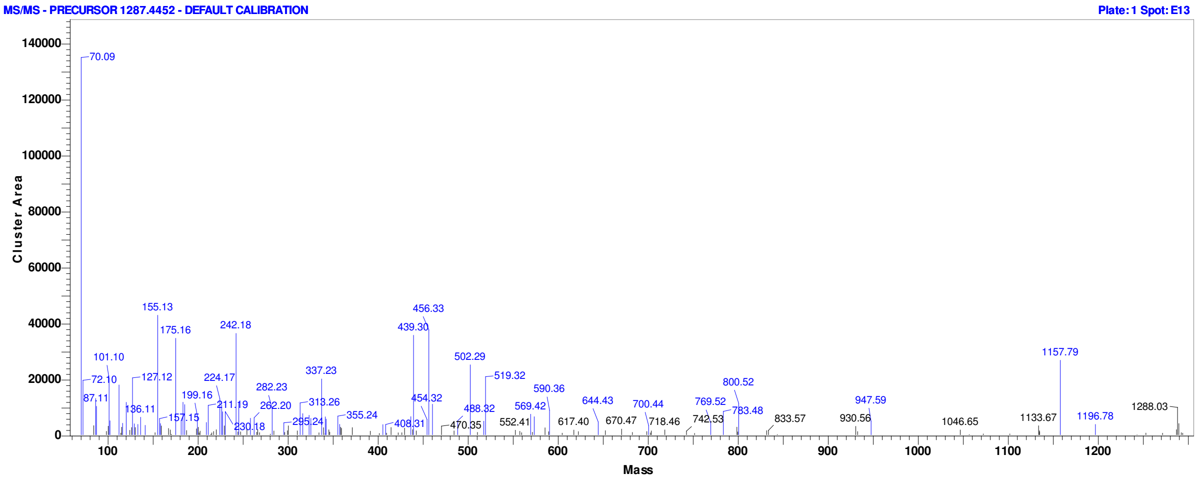

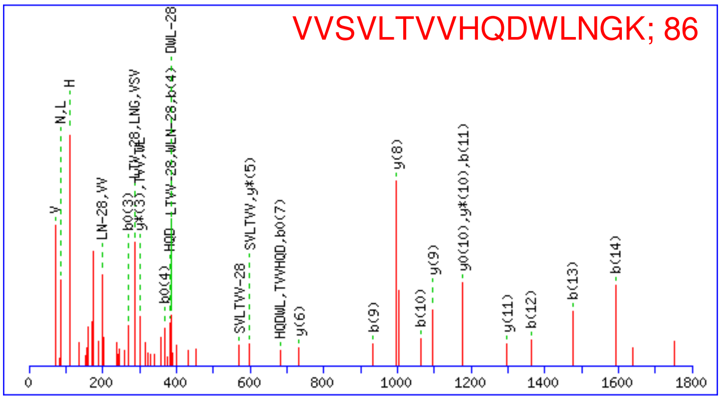

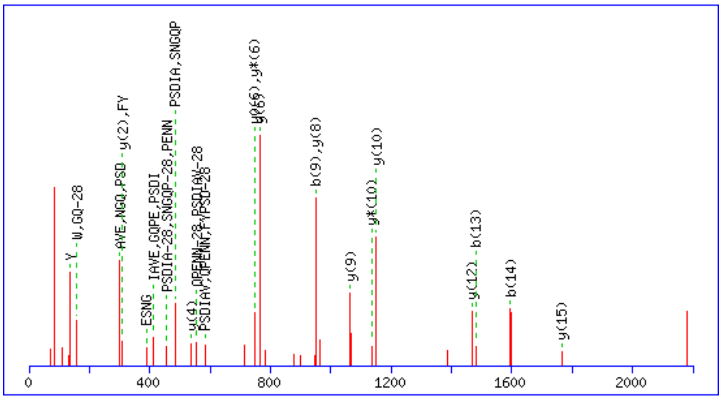
GFYPSDIAVEWESNGQPENNYK; 49

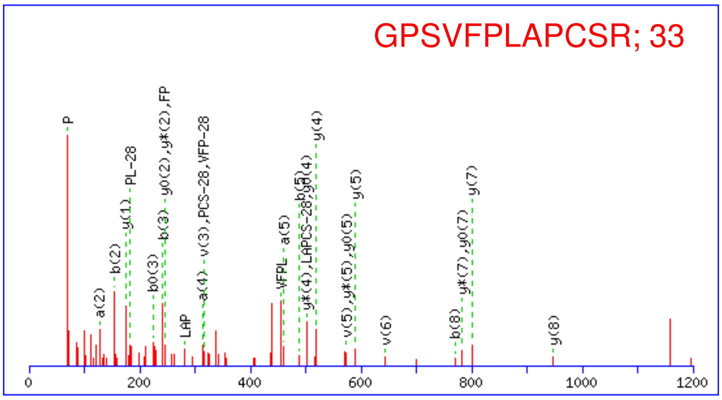

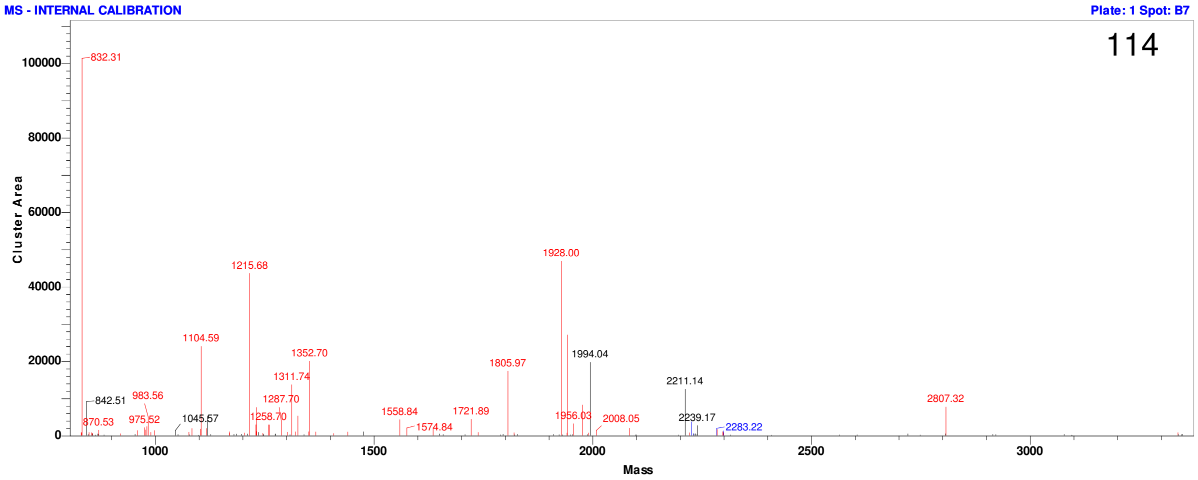
MS - INTERNAL CALIBRATION
Plate: 1 Spot: B7
114
Cluster Area
Mass
832.31
1215.68
1928.00
1104.59
1352.70
1311.74
1805.97
1994.04
2211.14
842.51
983.56
1287.70
1558.84
1721.89
2807.32
870.53
975.52
1045.57
1258.70
1574.84
1956.03
2008.05
2239.17
2283.22

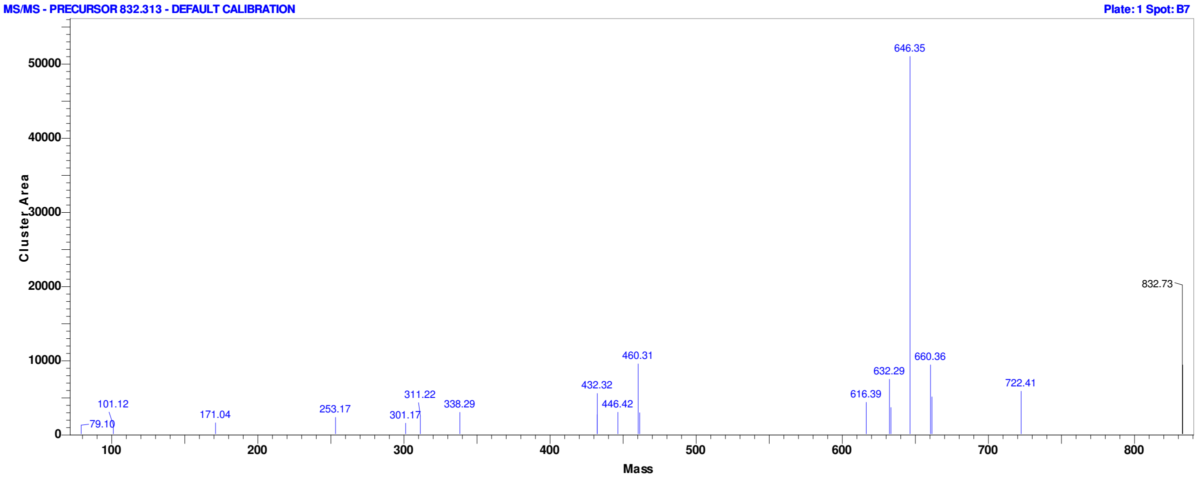
MS/MS - PRECURSOR 832.313 - DEFAULT CALIBRATION
Plate: 1 Spot: B7
Cluster Area
Mass
646.35
832.73
460.31
660.36
632.29
432.32
722.41
616.39
311.22
446.42
101.12
338.29
253.17
171.04
301.17
79.10

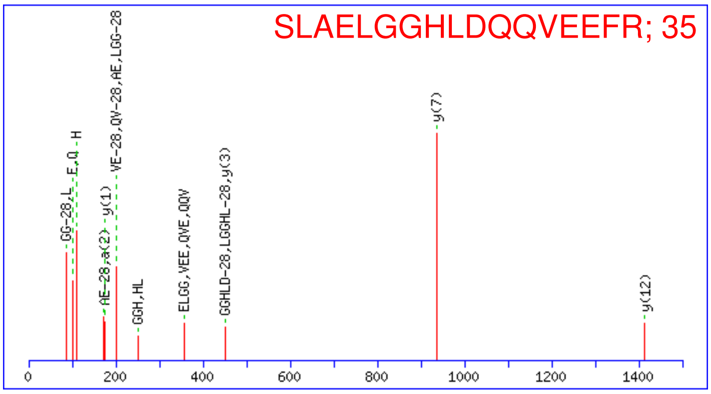
SLAELGGHLDQQVEEFR; 35
GG-28,L
E,Q
H
AE-28,a(2)
y(1)
VE-28,QV-28,AE,LGG-28
GGH,HL
ELGG,VEE,QVE,QQV
GGHLD-28,LGGHL-28,y(3)
y(7)
y(12)

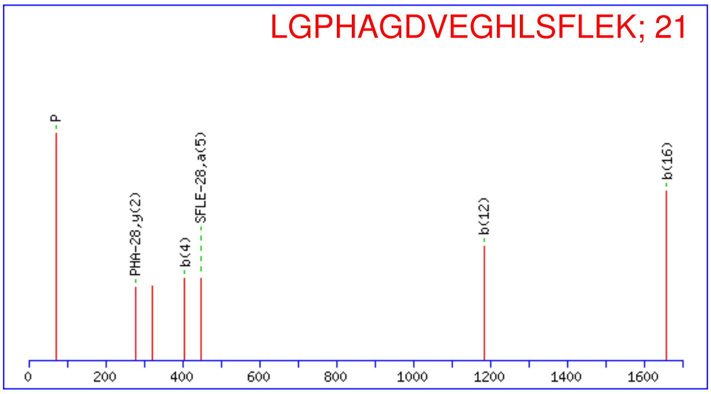
LGPHAGDVEGHLSFLEK; 21
P
PHA-28,y(2)
b(4)
SFLE-28,a(5)
b(12)
b(16)

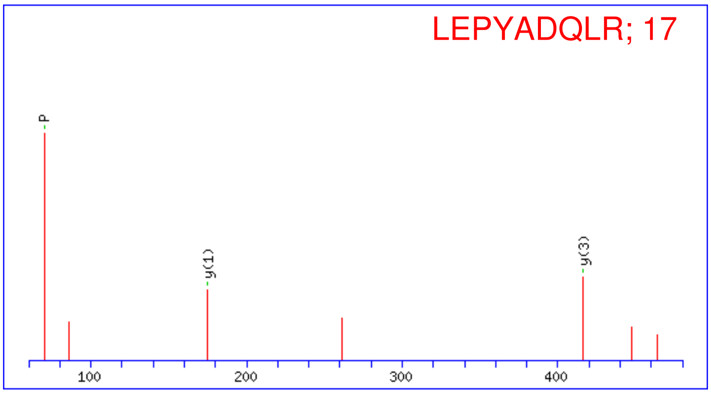
LEPYADQLR; 17
P
y(1)
y(3)

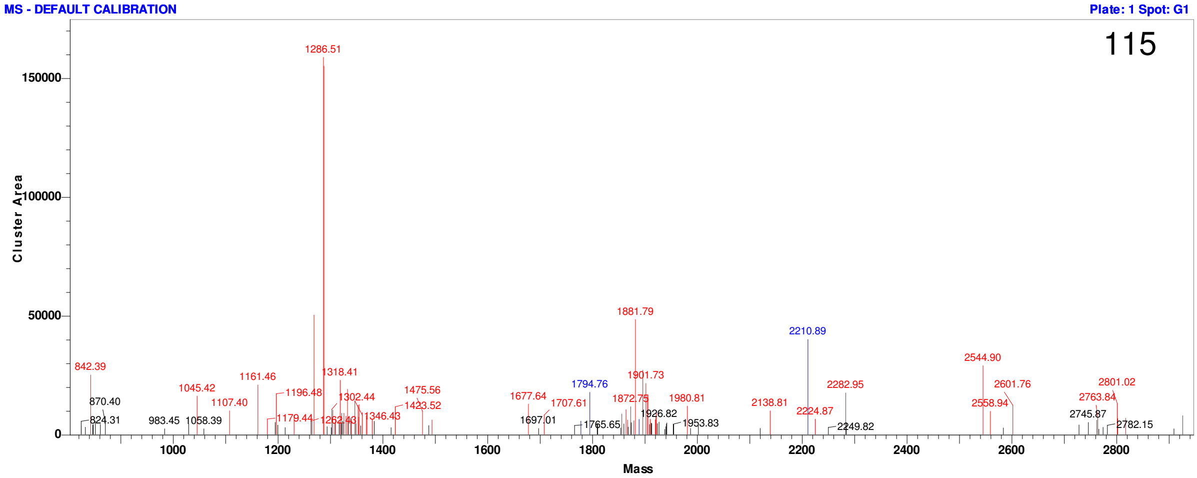

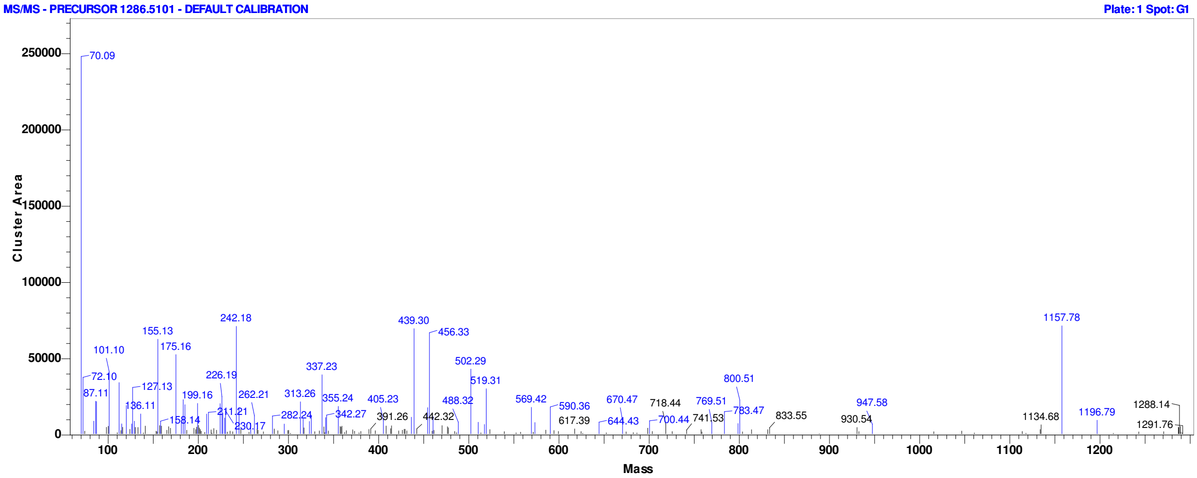

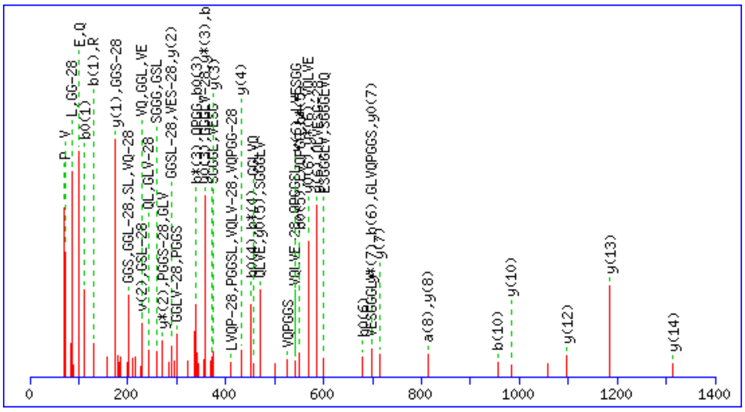

EVQLVESGGGLVQPGGSLR; 105

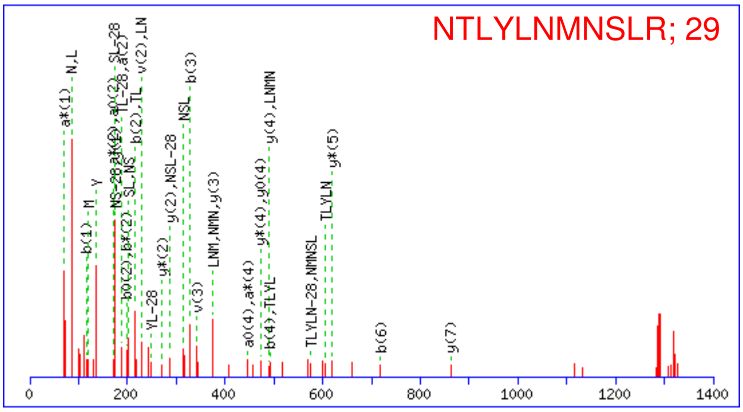

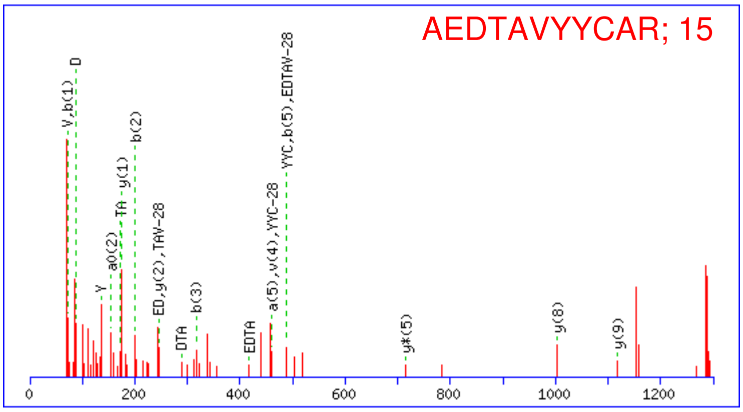

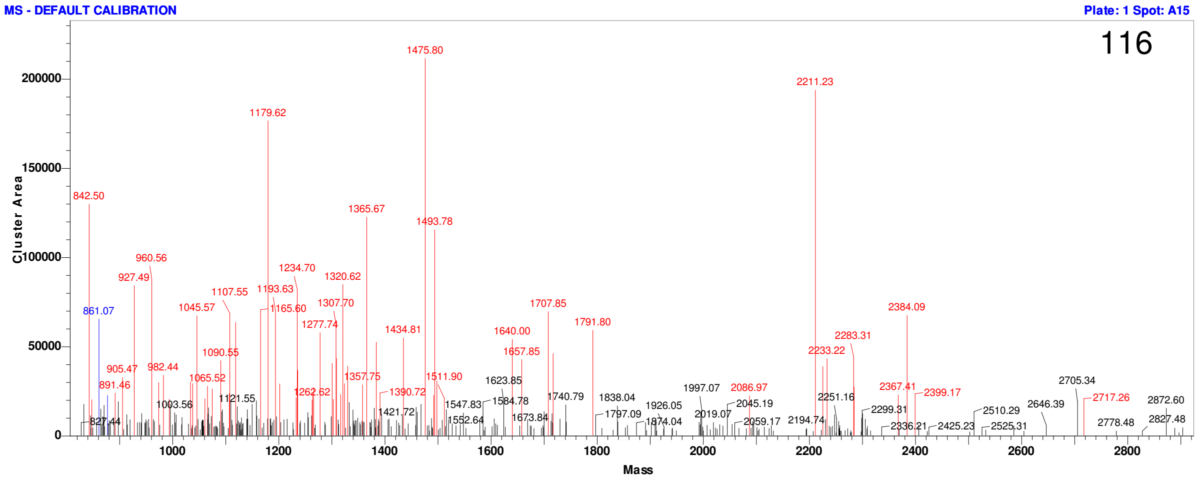
MS - DEFAULT CALIBRATION
Plate: 1 Spot: A15
116
Cluster Area
Mass
842.50
861.07
927.49
960.56
905.47
891.46
982.44
1003.56
827.44
1045.57
1065.52
1090.55
1107.55
1121.55
1179.62
1193.63
1165.60
1234.70
1262.62
1277.74
1307.70
1320.62
1357.75
1365.67
1390.72
1421.72
1434.81
1475.80
1493.78
1511.90
1547.83
1552.64
1584.78
1623.85
1640.00
1657.85
1673.84
1707.85
1740.79
1791.80
1797.09
1838.04
1874.04
1926.05
1997.07
2019.07
2045.19
2059.17
2086.97
2194.74
2211.23
2233.22
2251.16
2283.31
2299.31
2336.21
2367.41
2384.09
2399.17
2425.23
2510.29
2525.31
2646.39
2705.34
2717.26
2778.48
2827.48
2872.60

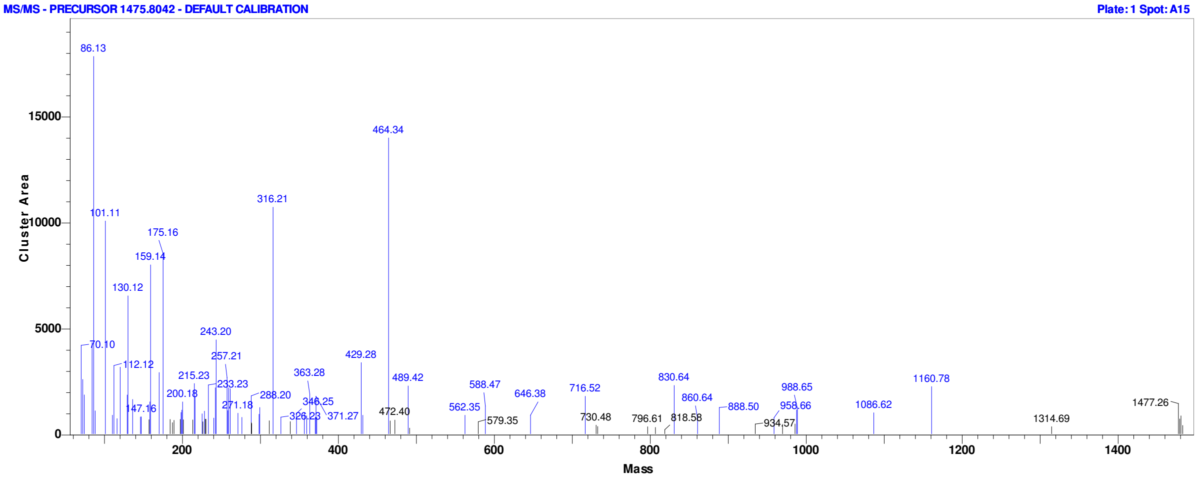
MS/MS - PRECURSOR 1475.8042 - DEFAULT CALIBRATION
Plate: 1 Spot: A15
Cluster Area
Mass
86.13
101.11
70.10
112.12
130.12
147.16
159.14
175.16
200.18
215.23
233.23
243.20
257.21
271.18
288.20
316.21
328.23
346.25
363.28
371.27
429.28
464.34
472.40
489.42
562.35
579.35
588.47
646.38
716.52
730.48
796.61
818.58
830.64
860.64
888.50
934.57
958.66
988.65
1086.62
1160.78
1314.69
1477.26

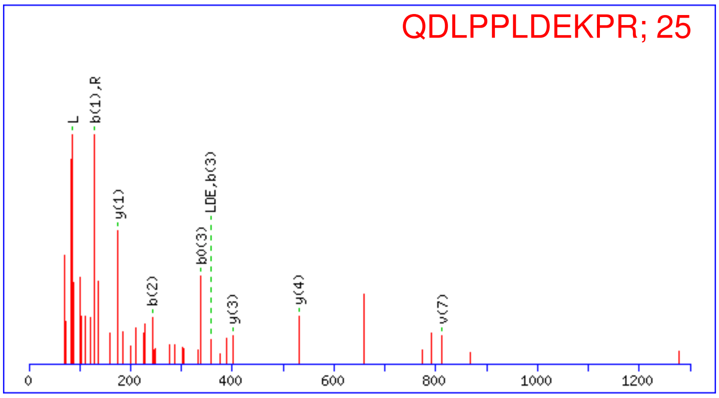
QDLPPLDEKPR; 25
L
b(1),R
y(1)
b(2)
b0(3)
LDE,b(3)
y(3)
y(4)
y(7)

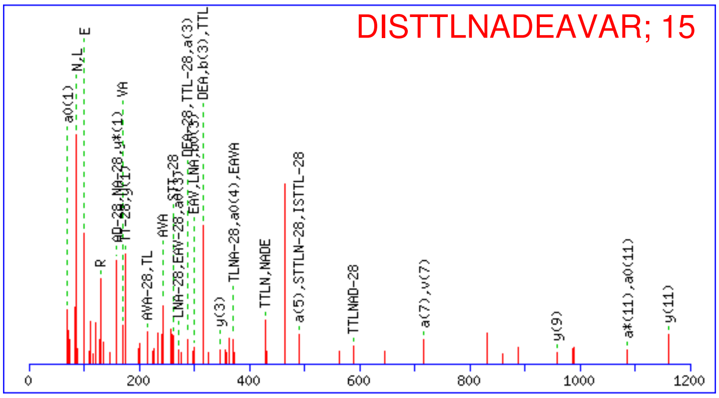
DISTTLNADEAVAR; 15
a0(1)
N,L
E
R
VA
AVA-28,TL
AVA
y(3)
TLNA-28,a0(4),EAVA
TTLN,NADE
a(5),STTLN-28,ISTTL-28
TTLNAD-28
a(7),y(7)
y(9)
a*(11),a0(11)
y(11)

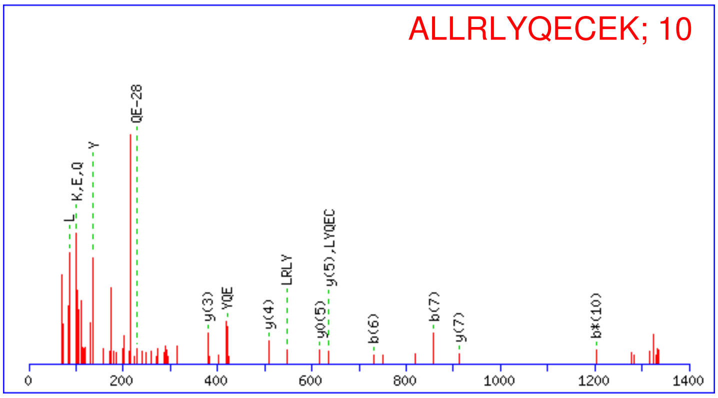
ALLRLYQECEK; 10
L
K,E,Q
Y
QE-28
y(3)
YQE
y(4)
LRLY
y0(5)
y(5),LYQEC
b(6)
b(7)
y(7)
b*(10)

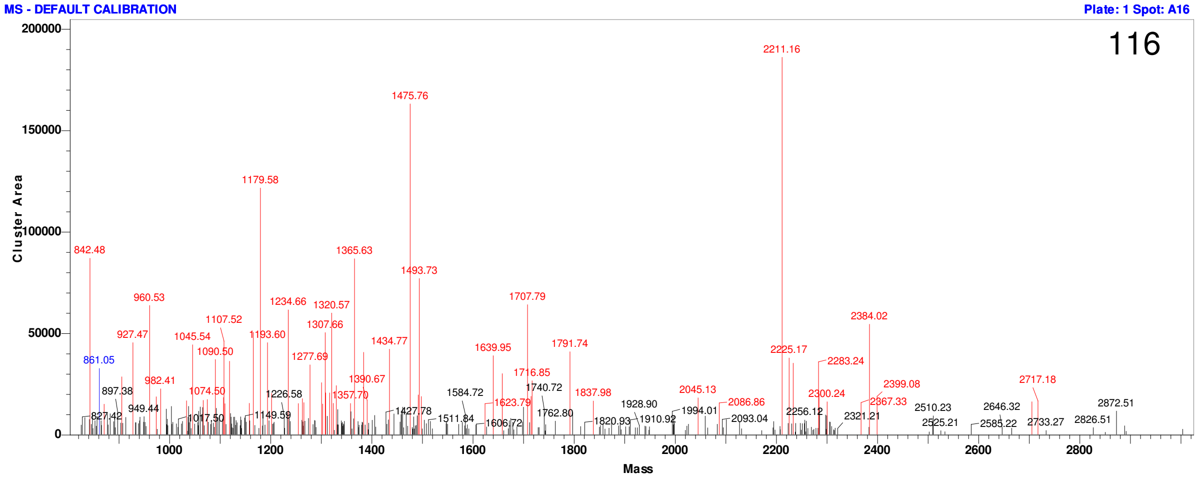

MS - DEFAULT CALIBRATION
Plate: 1 Spot: A16
116
Cluster Area
Mass
2211.16
1475.76
1179.58
842.48
1365.63
1493.73
960.53
1234.66
1320.57
1707.79
1107.52
2384.02
927.47
1045.54
1193.60
1307.66
1434.77
1791.74
861.05
1090.50
1639.95
2225.17
1277.69
2283.24
1716.85
982.41
1390.67
2399.08
2717.18
897.38
1074.50
1226.58
1357.70
1584.72
1740.72
1837.98
2045.13
2300.24
1623.79
1928.90
2086.86
2367.33
2510.23
2646.32
2872.51
949.44
827.42
1017.50
1149.59
1427.78
1511.84
1606.72
1762.80
1820.93
1910.92
1994.01
2093.04
2256.12
2321.21
2525.21
2585.22
2733.27
2826.51

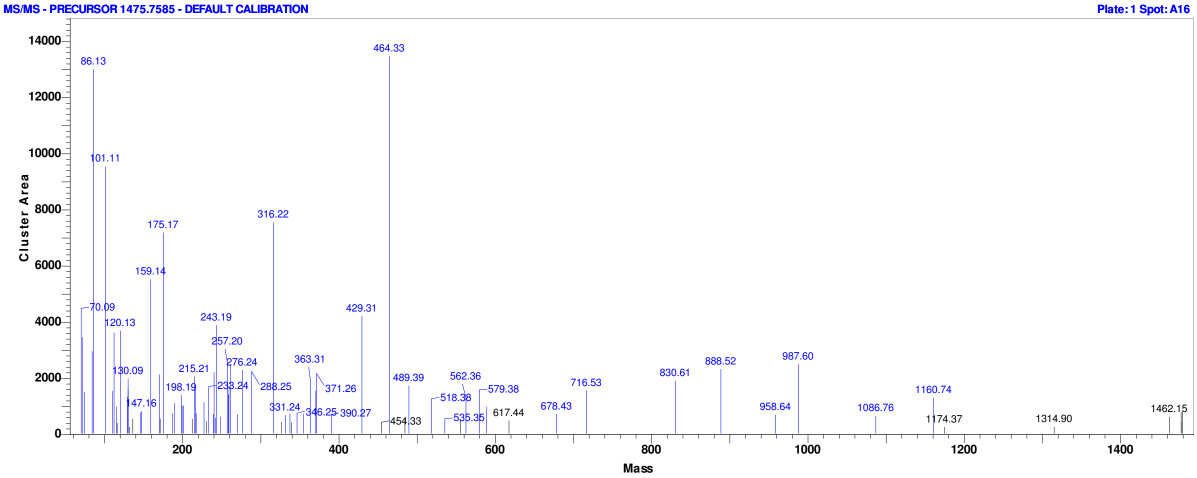

MS/MS - PRECURSOR 1475.7585 - DEFAULT CALIBRATION
Plate: 1 Spot: A16
Cluster Area
Mass
464.33
86.13
101.11
316.22
175.17
159.14
70.09
120.13
429.31
243.19
257.20
276.24
363.31
130.09
215.21
489.39
562.36
987.60
888.52
716.53
830.61
1160.74
198.19
233.24
288.25
371.26
518.38
579.38
147.16
331.24
346.25
390.27
454.33
535.35
617.44
678.43
958.64
1086.76
1174.37
1314.90
1462.15

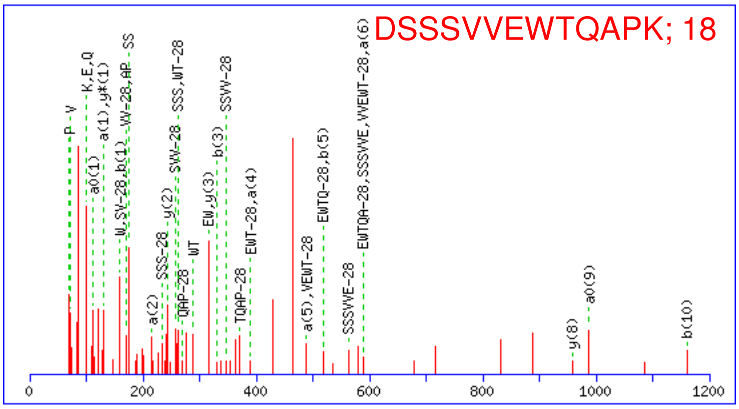

DSSSVVEWTQAPK; 18
P
V
K,E,Q
a0(1)
a(1),y*(1)
W,SV-28,b(1)
VV-28,AP
SS
a(2)
SSS-28
y(2)
SVV-28
SSS,WT-28
QAP-28
WT
EW,y(3)
b(3)
SSVV-28
TQAP-28
EWT-28,a(4)
a(5),VEWT-28
EWTQ-28,b(5)
SSSVVE-28
EWTQA-28,SSSVVE,VVEWT-28,a(6)
y(8)
a0(9)
b(10)

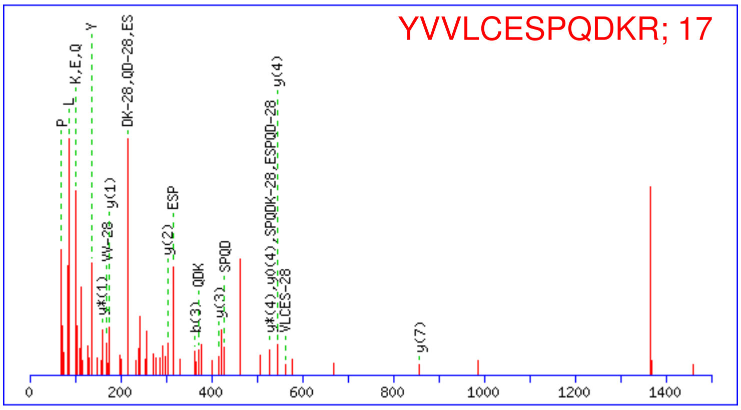

YVVLCESPQDKR; 17
P
L
K,E,Q
Y
y*(1)
VV-28
y(1)
DK-28,QD-28,ES
y(2)
ESP
b(3)
QDK
y(3)
SPQD
y*(4),y0(4),SPQDK-28,ESPQD-28
VLCES-28
y(4)
y(7)

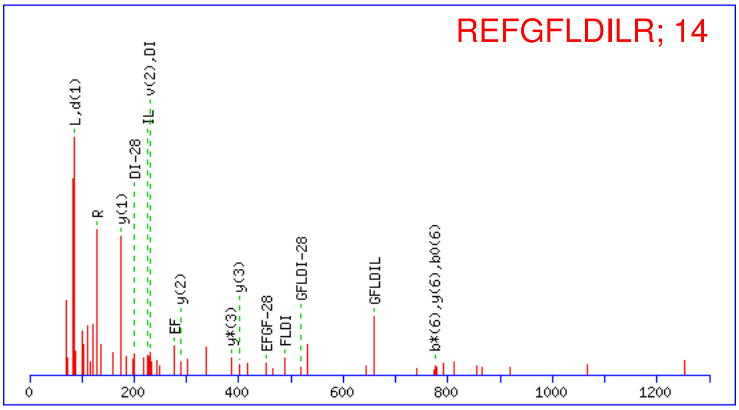

REFGFLDILR; 14
L,d(1)
R
y(1)
DI-28
IL
v(2),DI
EF
y(2)
y*(3)
y(3)
EFGF-28
FLDI
GFLDI-28
GFLDIL
b*(6),y(6),b0(6)

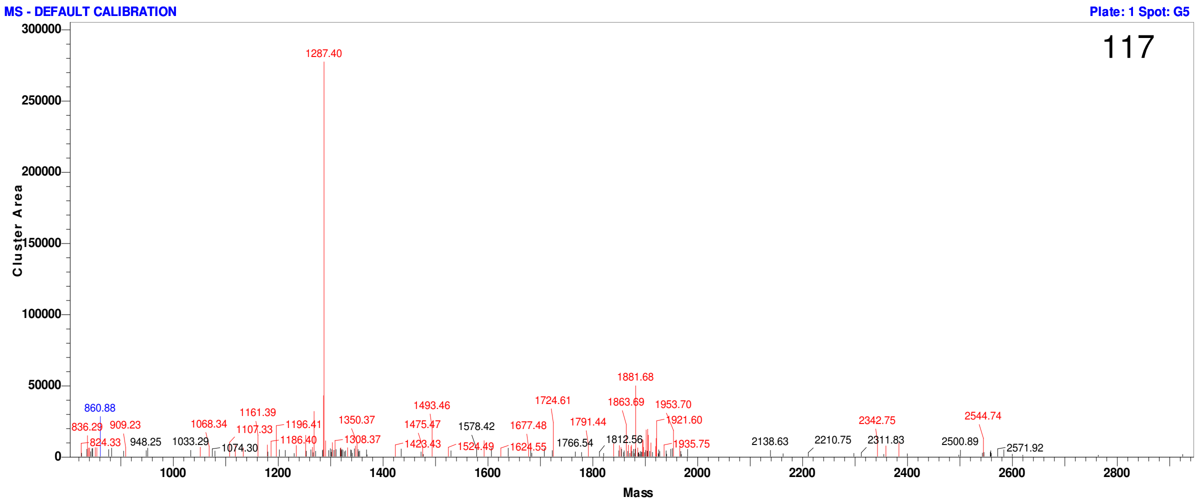

MS - DEFAULT CALIBRATION
Plate: 1 Spot: G5
117
Cluster Area
Mass
1287.40
860.88
836.29
909.23
824.33
948.25
1033.29
1068.34
1074.30
1107.33
1161.39
1196.41
1186.40
1350.37
1308.37
1493.46
1475.47
1423.43
1578.42
1524.49
1677.48
1624.55
1724.61
1766.54
1791.44
1812.56
1863.69
1881.68
1953.70
1921.60
1935.75
2138.63
2210.75
2311.83
2342.75
2500.89
2544.74
2571.92

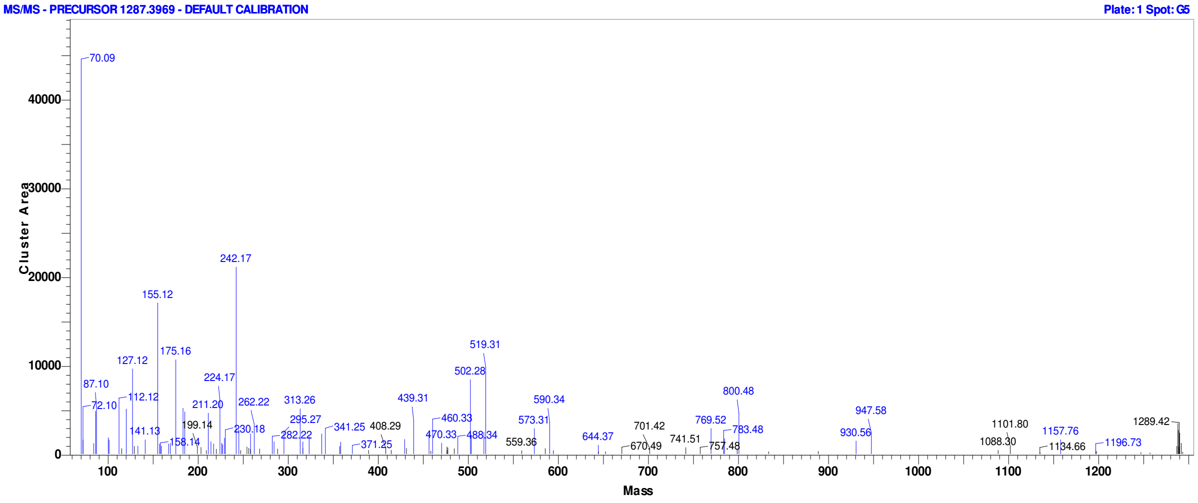

MS/MS - PRECURSOR 1287.3969 - DEFAULT CALIBRATION
Plate: 1 Spot: G5
Cluster Area
Mass
70.09
72.10
87.10
112.12
127.12
141.13
155.12
158.14
175.16
199.14
211.20
224.17
230.18
242.17
262.22
282.22
295.27
313.26
341.25
371.25
408.29
439.31
460.33
470.33
488.34
502.28
519.31
559.36
573.31
590.34
644.37
670.49
701.42
741.51
757.48
769.52
783.48
800.48
930.56
947.58
1088.30
1101.80
1134.66
1157.76
1196.73
1289.42

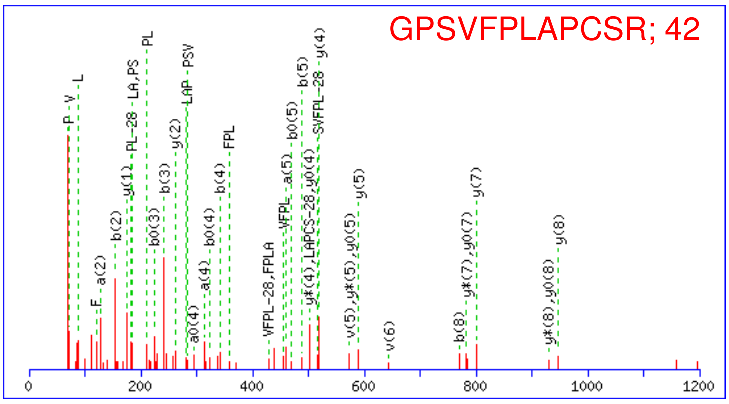

GPSVFPLAPCSR; 42

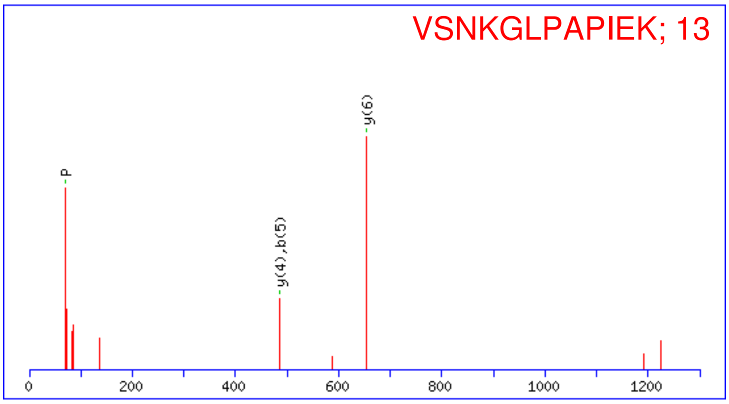

VSNKGLPAPIEK; 13

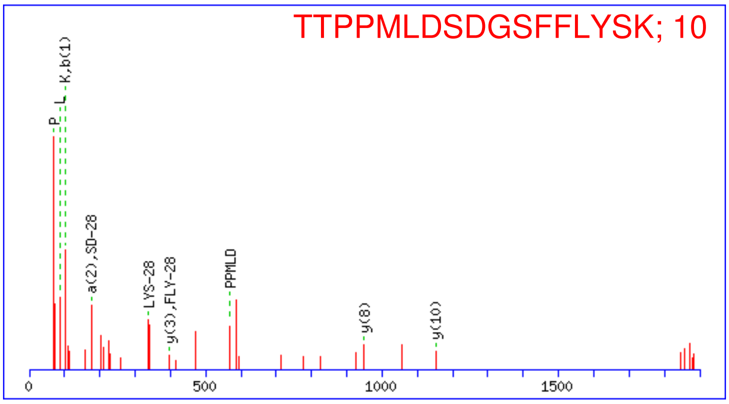

TTPPMLDSDGSFFLYSK; 10

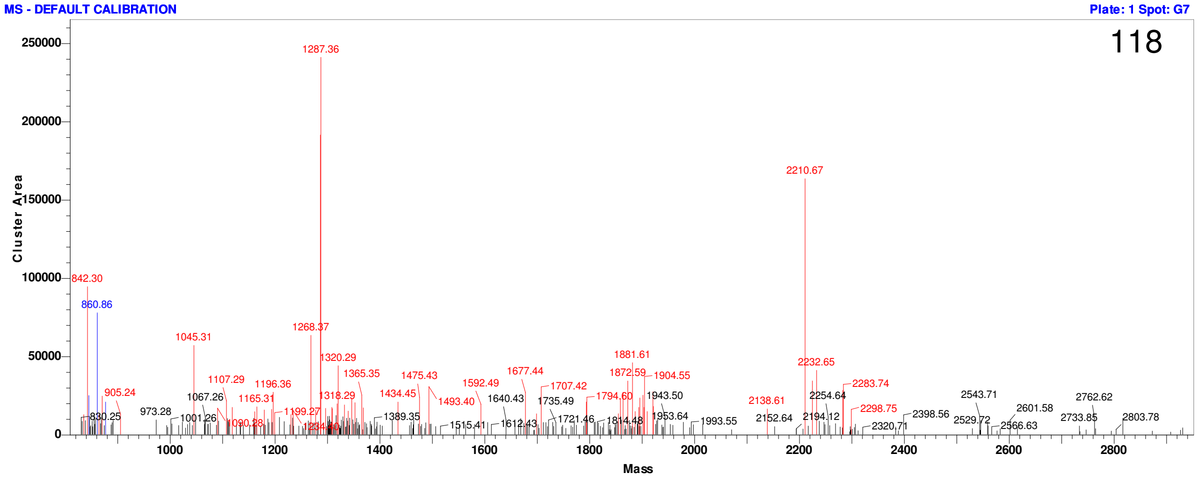
MS - DEFAULT CALIBRATION
Plate: 1 Spot: G7
118
Cluster Area
Mass
1287.36
2210.67
842.30
860.86
1045.31
1268.37
1320.29
1365.35
1475.43
1592.49
1677.44
1707.42
1881.61
1872.59
1904.55
2232.65
2283.74
2298.75
2138.61
905.24
830.25
973.28
1001.26
1067.26
1107.29
1196.36
1165.31
1090.28
1199.27
1318.29
1434.45
1389.35
1493.40
1640.43
1735.49
1794.60
1943.50
1953.64
1993.55
1515.41
1612.43
1721.46
1814.48
2152.64
2194.12
2254.64
2320.71
2398.56
2543.71
2529.72
2566.63
2601.58
2733.85
2762.62
2803.78

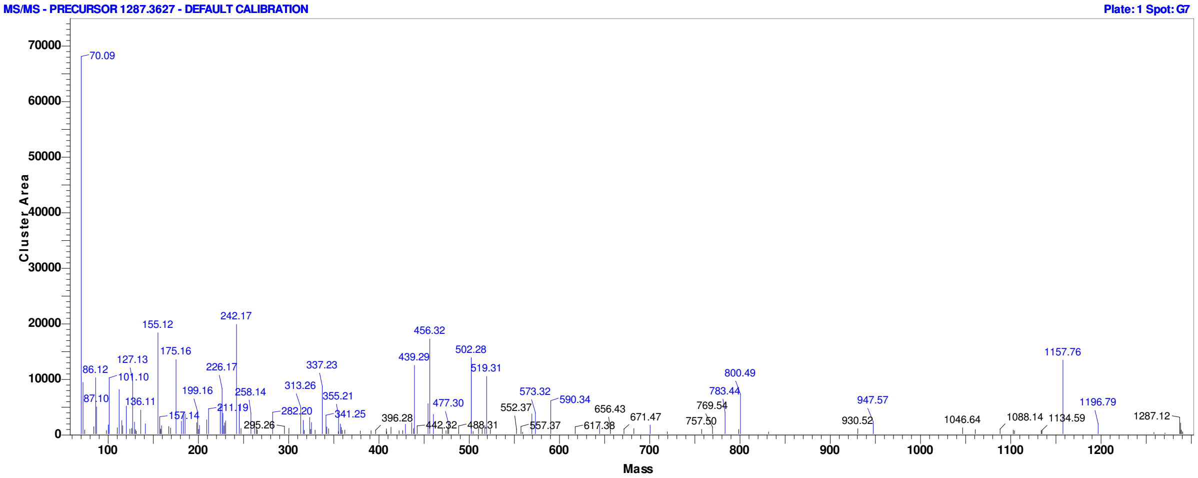
MS/MS - PRECURSOR 1287.3627 - DEFAULT CALIBRATION
Plate: 1 Spot: G7
Cluster Area
Mass
70.09
86.12
87.10
101.10
127.13
136.11
155.12
157.14
175.16
199.16
211.19
226.17
242.17
258.14
282.20
295.26
313.26
337.23
341.25
355.21
396.28
439.29
442.32
456.32
477.30
488.31
502.28
519.31
552.37
557.37
573.32
590.34
617.38
656.43
671.47
757.50
769.54
783.44
800.49
930.52
947.57
1046.64
1088.14
1134.59
1157.76
1196.79
1287.12

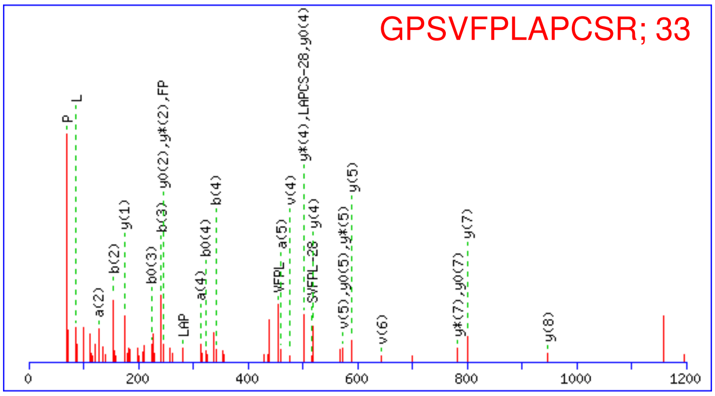
GPSVFPLAPCSR; 33
P
L
a(2)
b(2)
y(1)
b0(3)
b(3)
y0(2),y*(2),FP
LAP
a(4)
b0(4)
b(4)
VFPL
a(5)
v(4)
y*(4),LAPCS-28,y0(4)
SVFPL-28
y(4)
v(5),y0(5),y*(5)
y(5)
v(6)
y*(7),y0(7)
y(7)
y(8)

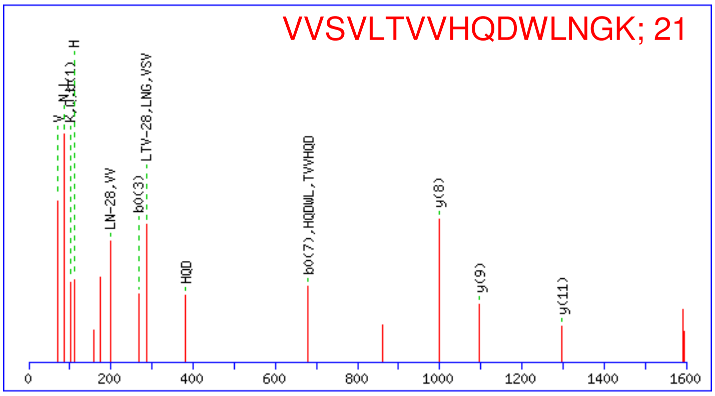
VVSVLTVVHQDWLNGK; 21
V
N
K,Q,b(1)
H
LN-28,VV
b0(3)
LTV-28,LNG,VSV
HQD
b0(7),HQDWL,TVVHQD
y(8)
y(9)
y(11)

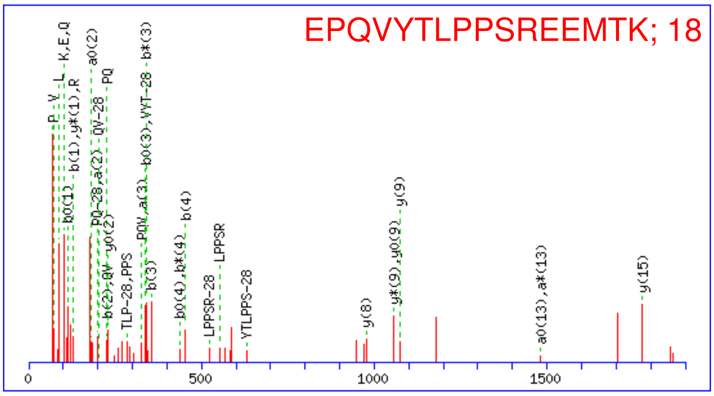
EPQVYTLPPSREEMTK; 18
P
V
L
K,E,Q
b0(1)
b(1),y*(1),R
PQ-28,a(2)
QV-28
a0(2)
PQ
b(2),QV
y0(2)
TLP-28,PPS
PQV,a(3)
b0(3),VYT-28
b*(3)
b(3)
b0(4),b*(4)
b(4)
LPPSR-28
LPPSR
YTLPPS-28
y(8)
y*(9),y0(9)
y(9)
a0(13),a*(13)
y(15)

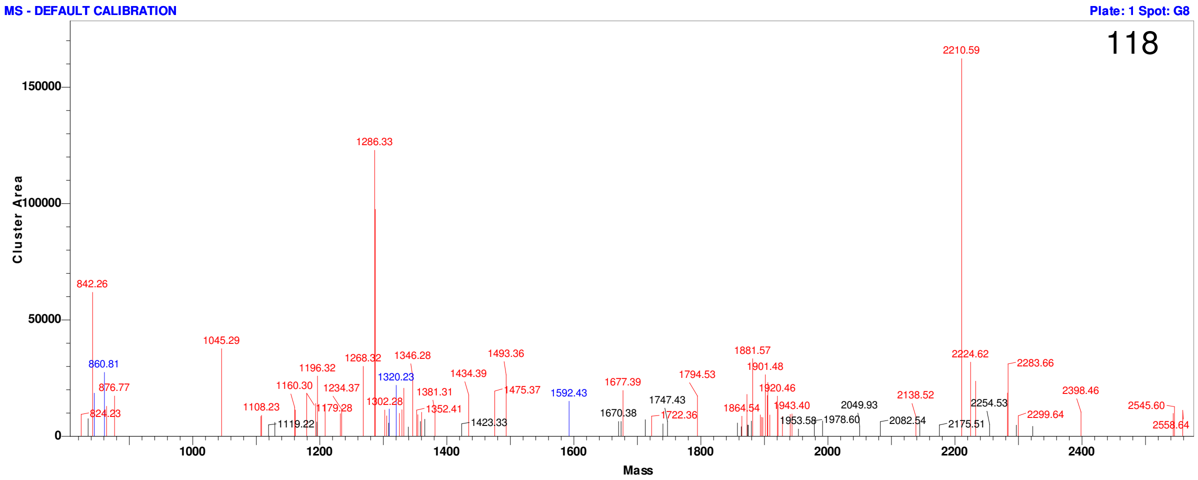

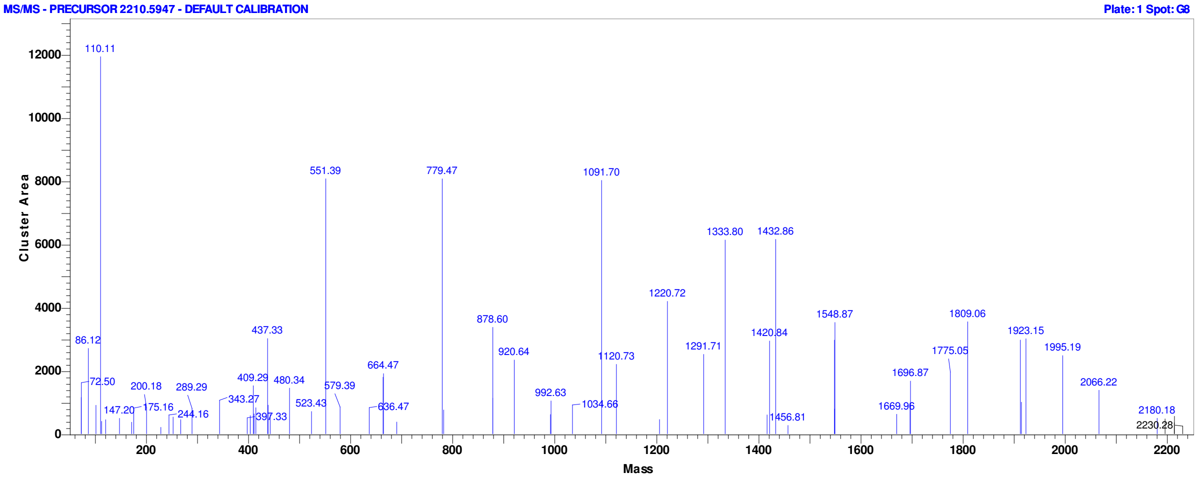

Sphingosine kinase 1

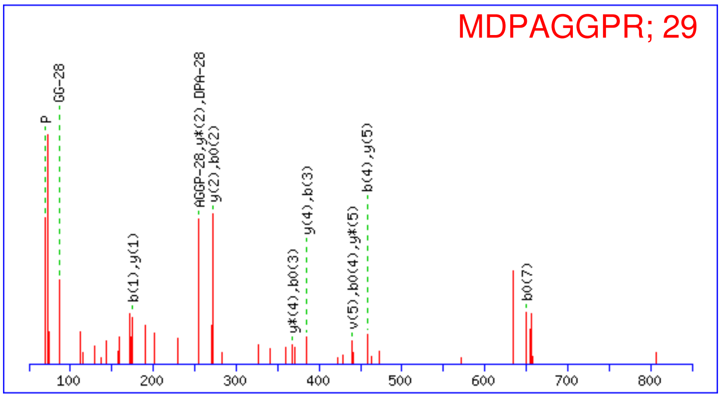

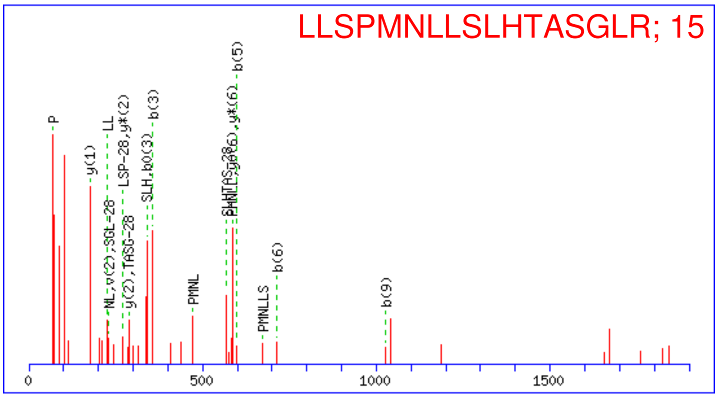

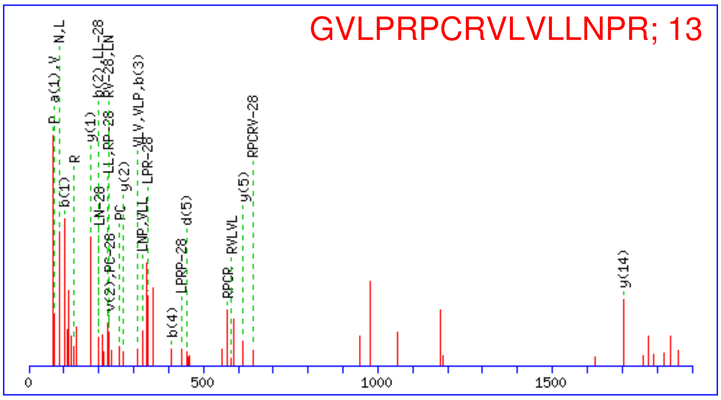

Rho-GTPase-activating protein 25

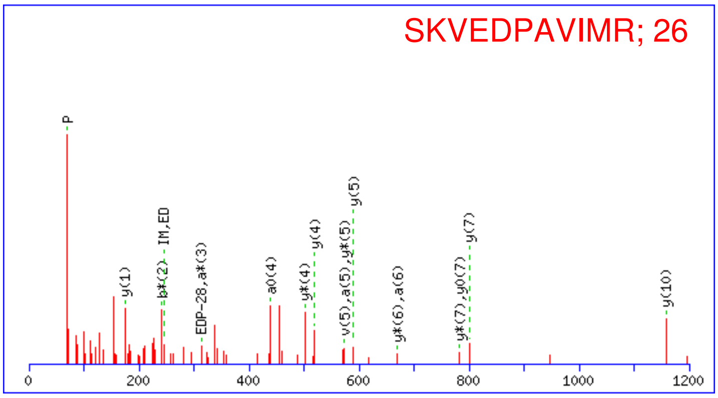

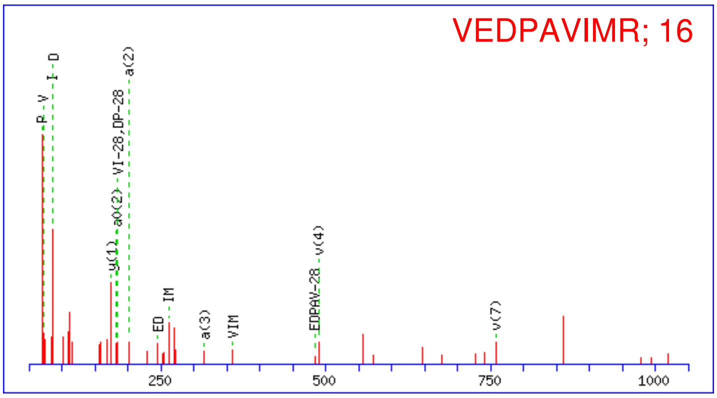

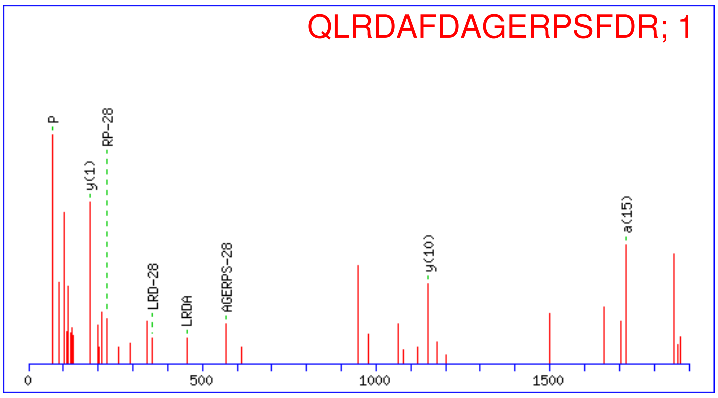

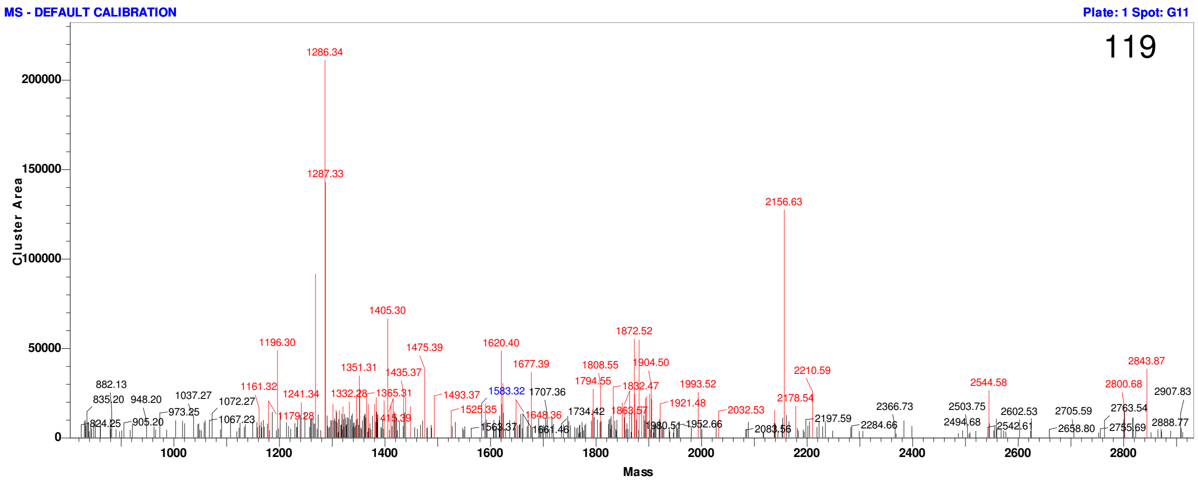

MS - DEFAULT CALIBRATION
Plate: 1 Spot: G11
119
Cluster Area
Mass
1286.34
1287.33
2156.63
1405.30
1872.52
1196.30
1620.40
1475.39
1904.50
1351.31
1677.39
1808.55
2210.59
2544.58
2843.87
1435.37
1794.55
1161.32
1832.47
1993.52
2800.68
1241.34
1332.28
1365.31
1583.32
1707.36
882.13
835.20
948.20
1037.27
1072.27
1493.37
2178.54
2366.73
2503.75
2602.53
2705.59
2763.54
2907.83
973.25
1525.35
1921.48
2032.53
1734.42
1179.28
1415.39
1649.36
1863.57
2197.59
2494.68
824.25
905.20
1067.23
1563.37
1661.46
1930.51
1952.66
2083.56
2284.66
2542.61
2658.80
2755.69
2888.77

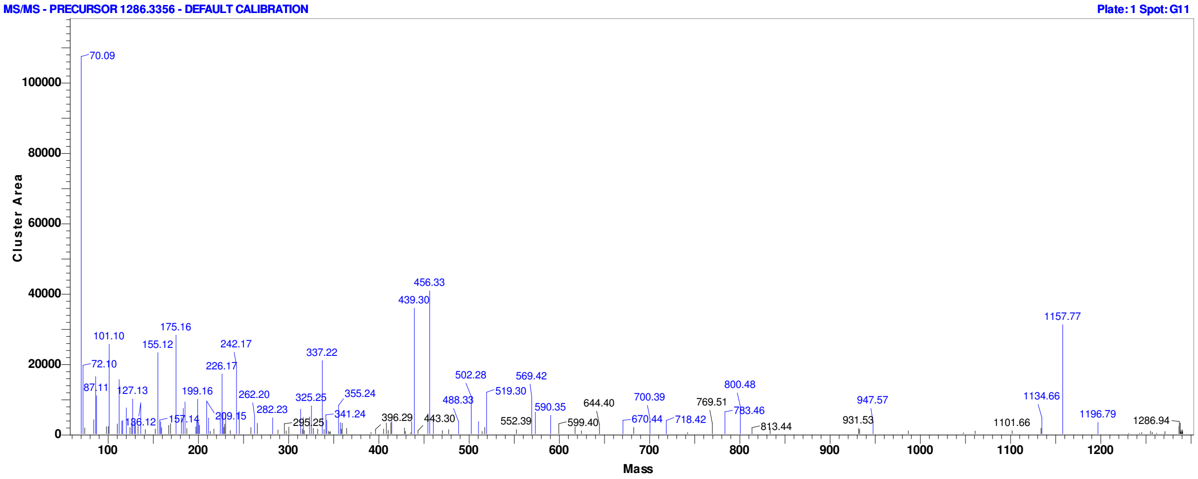

MS/MS - PRECURSOR 1286.3356 - DEFAULT CALIBRATION
Plate: 1 Spot: G11
Cluster Area
Mass
70.09
456.33
439.30
1157.77
101.10
175.16
155.12
242.17
72.10
226.17
337.22
502.28
569.42
800.48
87.11
127.13
199.16
262.20
325.25
355.24
519.30
488.33
700.39
769.51
1134.66
947.57
644.40
590.35
783.46
1196.79
136.12
157.14
209.15
282.23
341.24
396.29
443.30
552.39
599.40
670.44
718.42
813.44
931.53
1101.66
1286.94
295.25

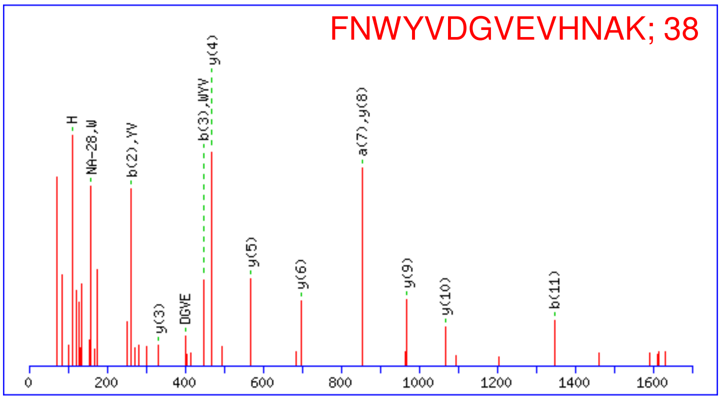

FNWYVDGVEVHNAK; 38
H
NA-28,W
b(2),YV
y(3)
DGVE
b(3),WYV
y(4)
y(5)
y(6)
a(7),y(8)
y(9)
y(10)
b(11)

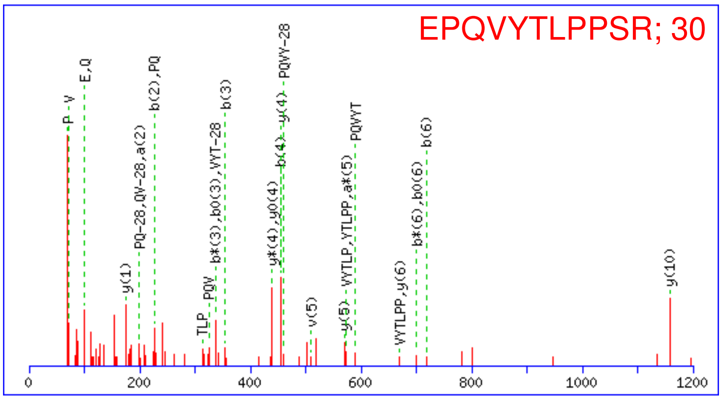

EPQVYTLPPSR; 30
P
V
E,Q
y(1)
PQ-28,QV-28,a(2)
b(2),PQ
TLP
PQV
b*(3),b0(3),VYT-28
b(3)
y*(4),y0(4)
b(4)
y(4)
PQVY-28
y(5)
y(5)
VYTLP,YTLPP,a*(5)
PQVYT
VYTLPP,y(6)
b*(6),b0(6)
b(6)
y(10)

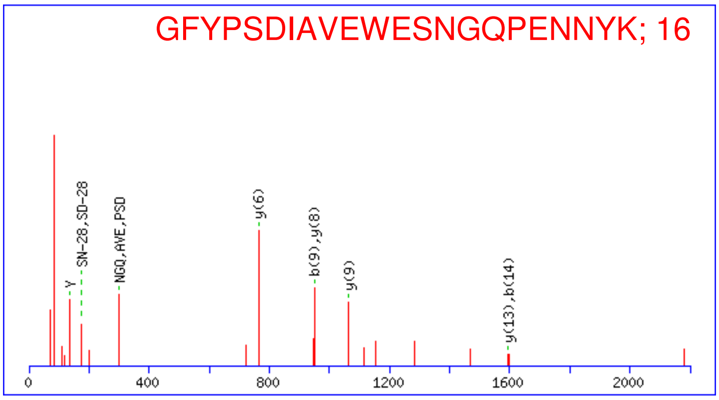

GFYPSDIAVEWESNGQPENNYK; 16
Y
SN-28,SD-28
NGQ,AVE,PSD
y(6)
b(9),y(8)
y(9)
y(13),b(14)

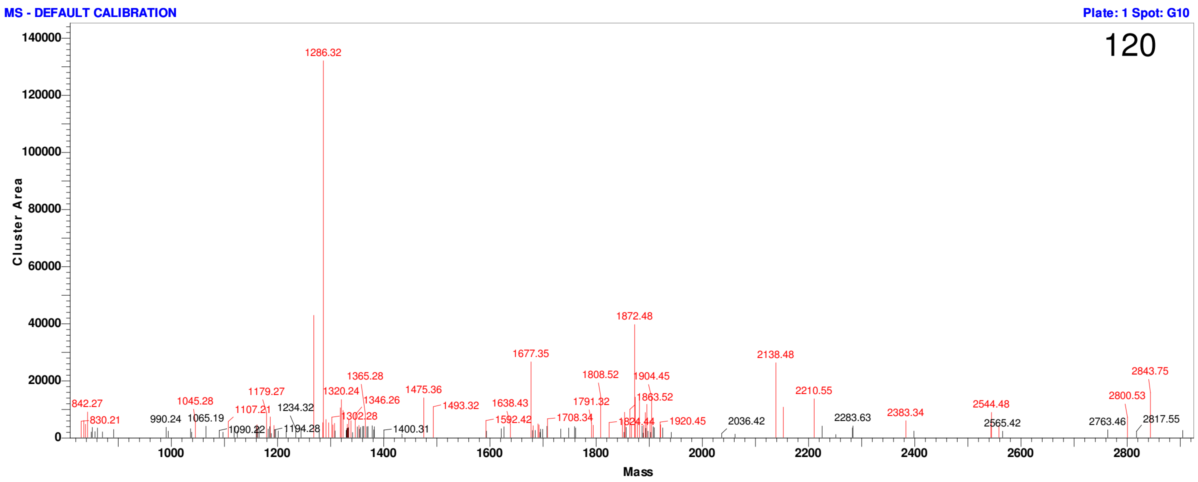
MS - DEFAULT CALIBRATION
Plate: 1 Spot: G10
120
Cluster Area
Mass
1286.32
1872.48
1677.35
2138.48
1808.52
1904.45
1365.28
1475.36
2843.75
2210.55
1320.24
1346.26
1179.27
1045.28
1638.43
1791.32
1863.52
2800.53
2544.48
2383.34
842.27
830.21
1107.21
1234.32
990.24
1065.19
1090.22
1194.28
1302.28
1400.31
1493.32
1592.42
1708.34
1824.44
1920.45
2036.42
2283.63
2565.42
2763.46
2817.55

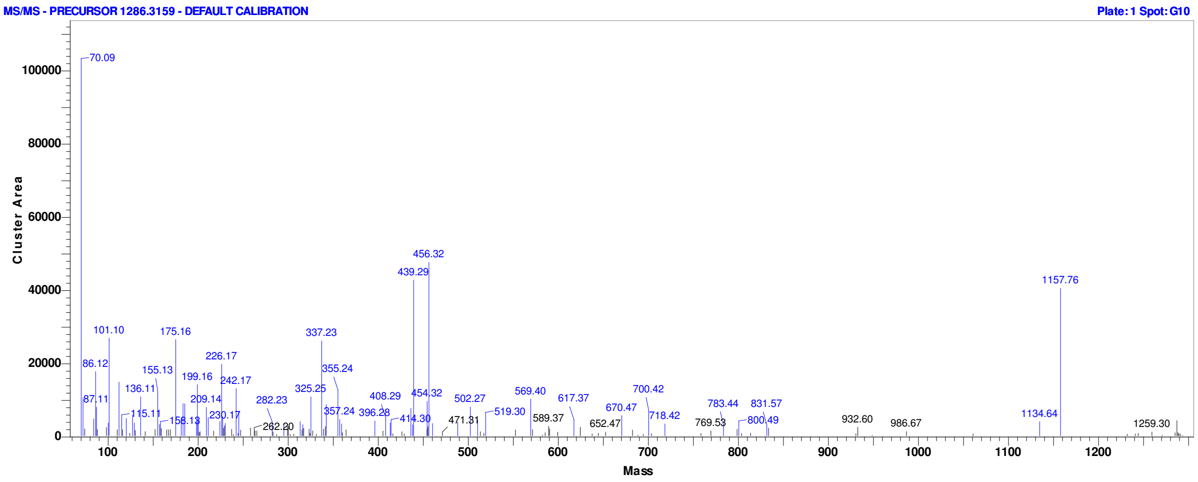
MS/MS - PRECURSOR 1286.3159 - DEFAULT CALIBRATION
Plate: 1 Spot: G10
Cluster Area
Mass
70.09
456.32
439.29
1157.76
101.10
175.16
337.23
86.12
226.17
355.24
155.13
199.16
242.17
136.11
325.25
87.11
209.14
282.23
408.29
454.32
502.27
569.40
617.37
700.42
783.44
831.57
670.47
115.11
158.13
230.17
357.24
396.28
414.30
519.30
718.42
800.49
1134.64
262.20
471.31
589.37
652.47
769.53
932.60
986.67
1259.30

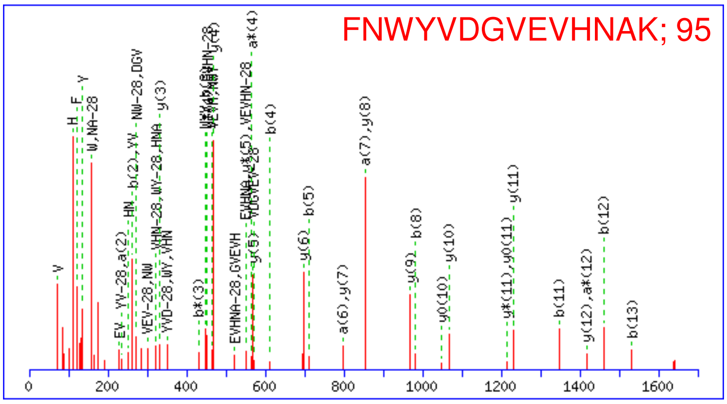
FNWYVDGVEVHNAK; 95

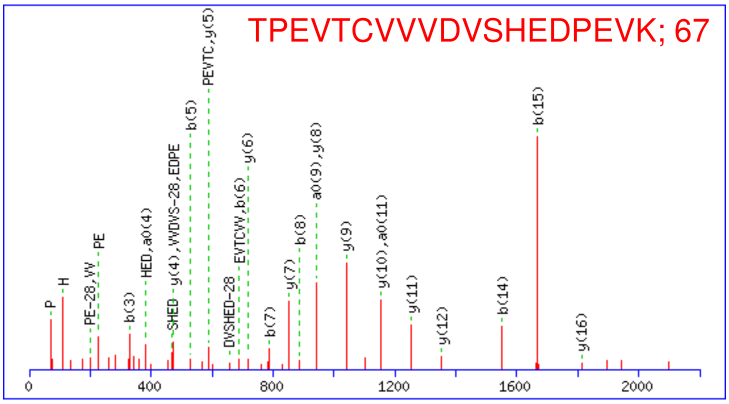
TPEVTCVVVDVSHEDPEVK; 67

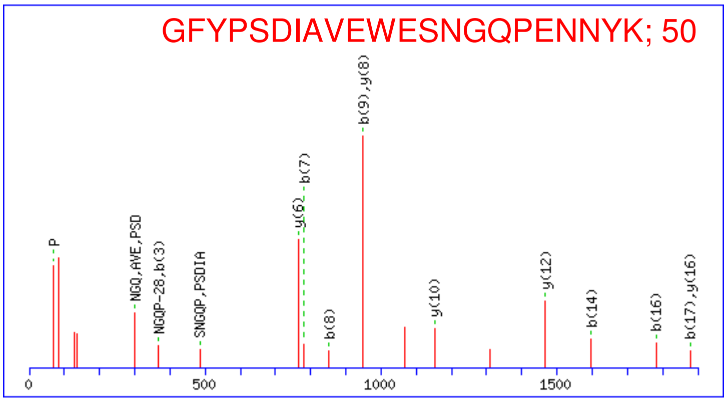
GFYPSDIAVEWESNGQPENNYK; 50

121

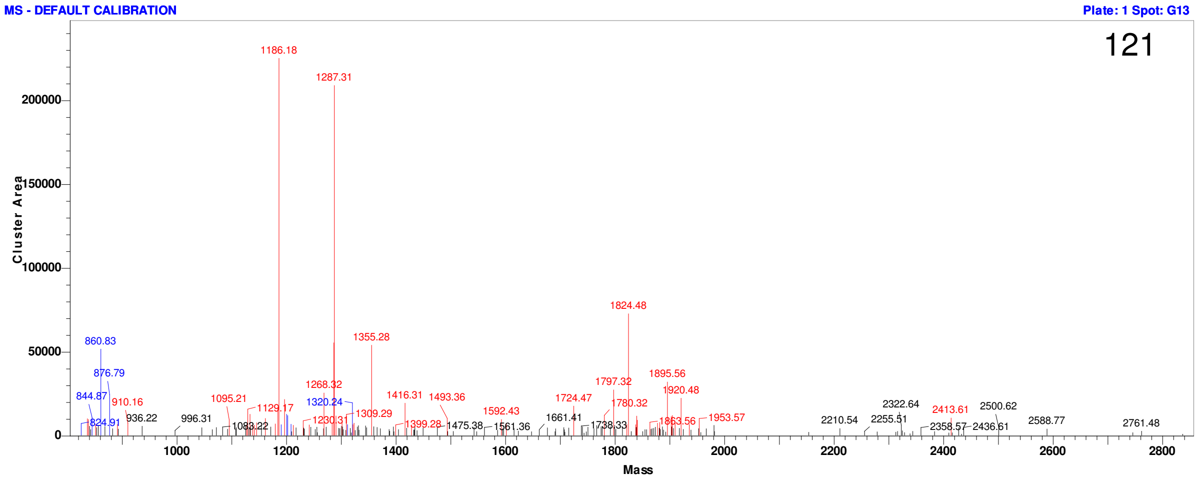

MS - DEFAULT CALIBRATION
Plate: 1 Spot: G13
Cluster Area
Mass
1186.18
1287.31
1824.48
1355.28
860.83
876.79
844.87
824.91
910.16
936.22
996.31
1095.21
1083.22
1129.17
1268.32
1320.24
1230.31
1309.29
1416.31
1399.28
1493.36
1475.38
1592.43
1561.36
1724.47
1661.41
1797.32
1780.32
1738.33
1895.56
1920.48
1863.56
1953.57
2210.54
2255.51
2322.64
2358.57
2413.61
2436.61
2500.62
2588.77
2761.48

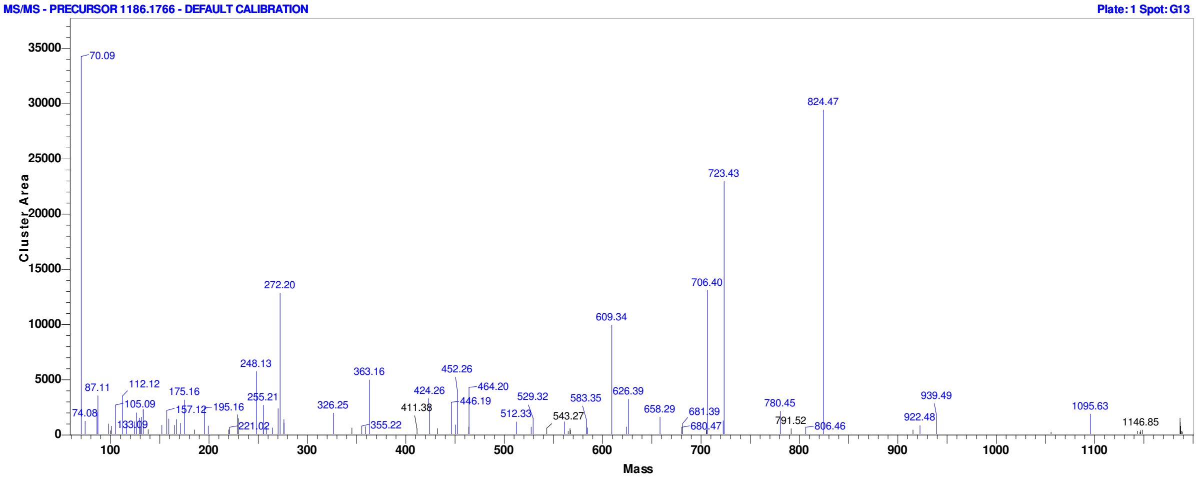

MS/MS - PRECURSOR 1186.1766 - DEFAULT CALIBRATION
Plate: 1 Spot: G13
Cluster Area
Mass
70.09
824.47
723.43
706.40
272.20
609.34
248.13
363.16
452.26
87.11
112.12
175.16
105.09
157.12
195.16
74.08
133.09
221.02
255.21
326.25
355.22
411.38
424.26
446.19
464.20
512.33
529.32
543.27
583.35
626.39
658.29
681.39
680.47
780.45
791.52
806.46
922.48
939.49
1095.63
1146.85

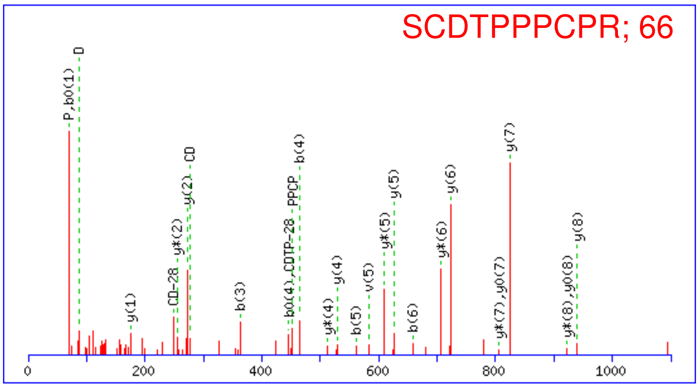

SCDTPPPCPR; 66
P,b0(1)
D
y(1)
CD-28
y*(2)
y(2)
CD
b(3)
b0(4),CDTP-28
PPCP
b(4)
y*(4)
y(4)
b(5)
y(5)
y*(5)
y(5)
b(6)
y*(6)
y(6)
y*(7),y0(7)
y(7)
y*(8),y0(8)
y(8)

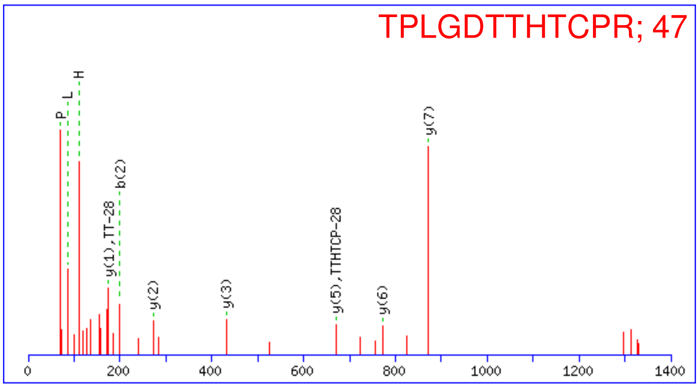

TPLGDTTHTCPR; 47
P
L
H
y(1),TT-28
b(2)
y(2)
y(3)
y(5),TTHTCP-28
y(6)
y(7)

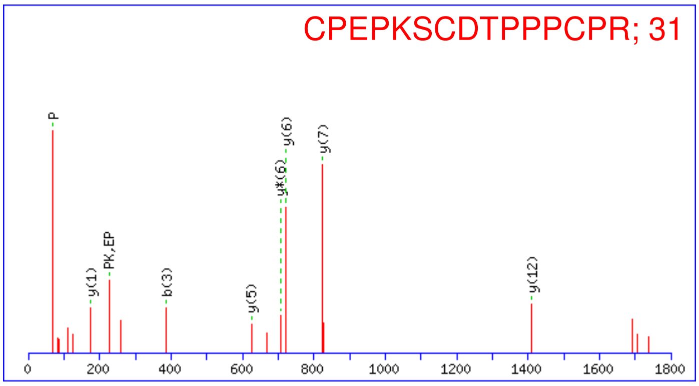

CPEPKSCDTPPPCPR; 31
P
y(1)
PK,EP
b(3)
y(5)
y*(6)
y(6)
y(7)
y(12)

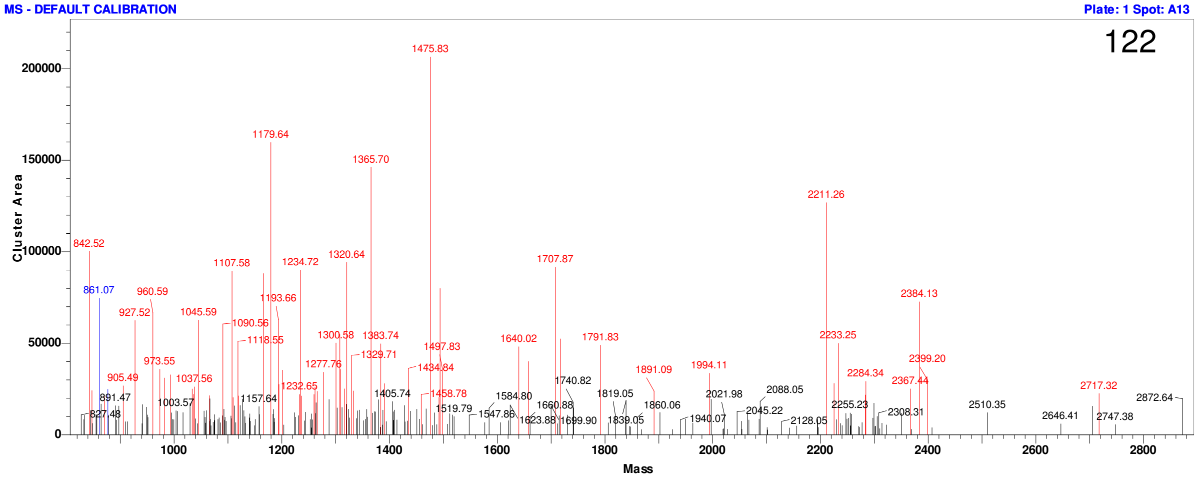

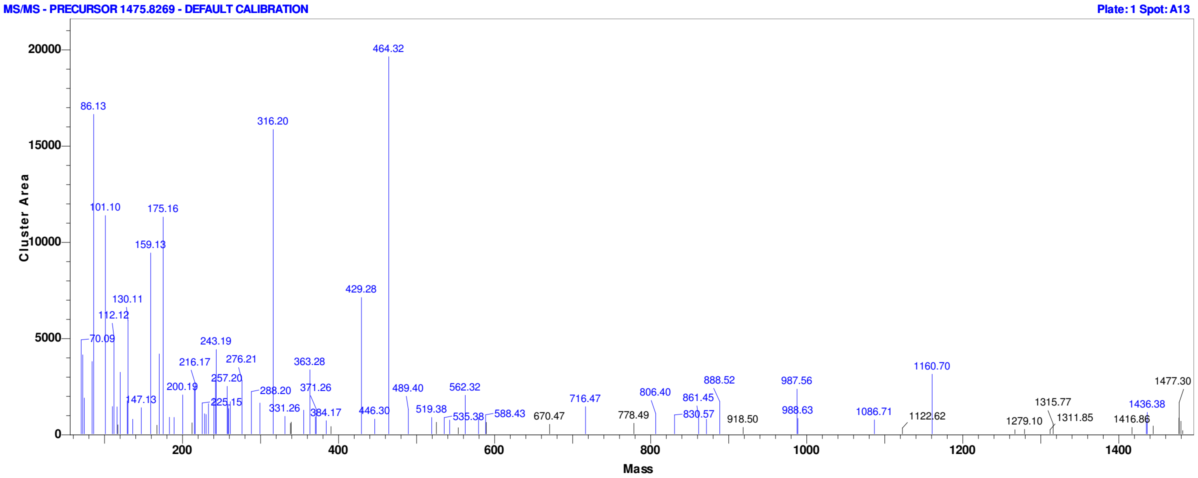

Glypican-1 precursor

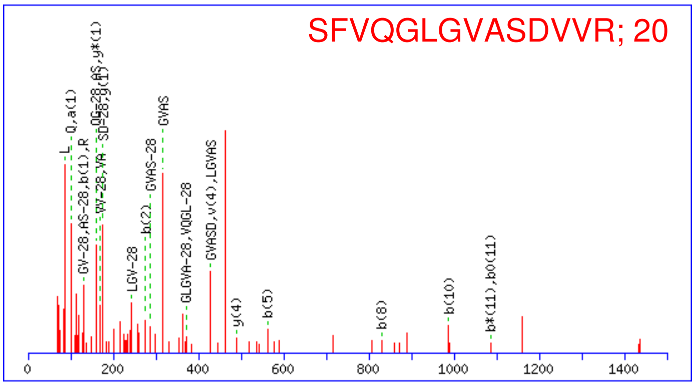

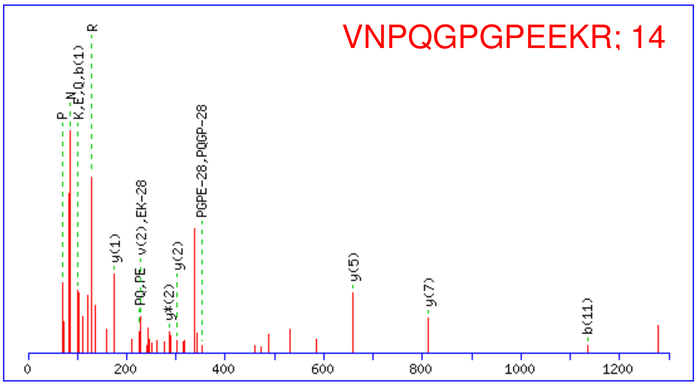

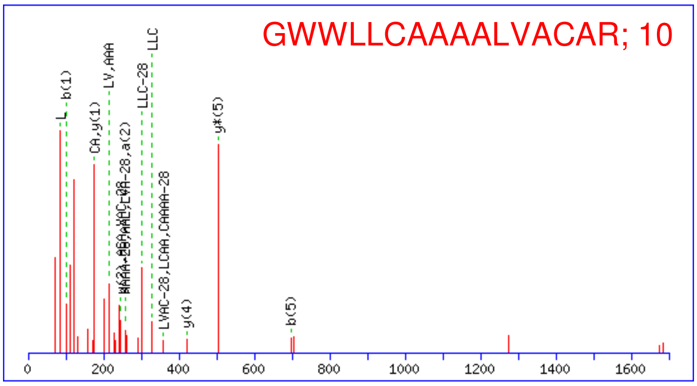

Ankyrin repeat and SOCS box protein 2 (ASB-2) (spot 122)

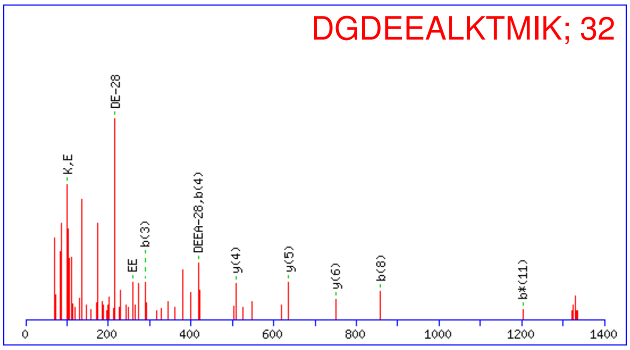

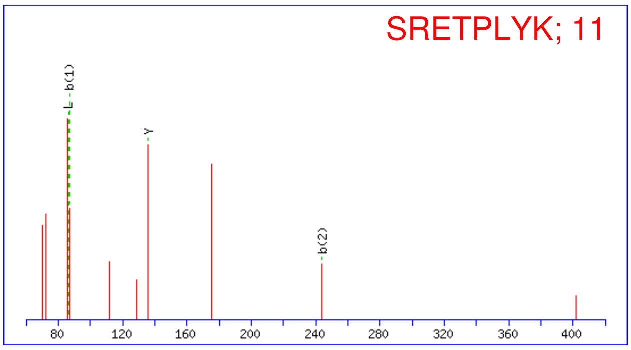

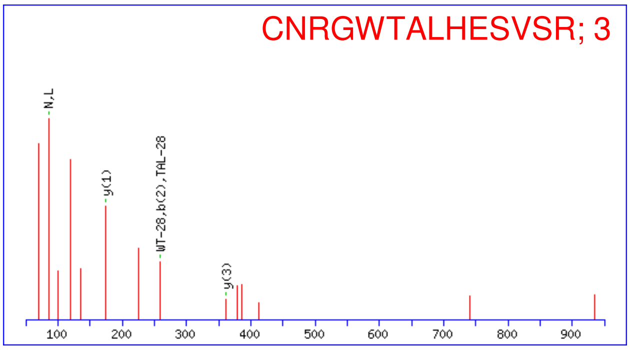

Polypeptide N-acetylgalactosaminyltransferase 13

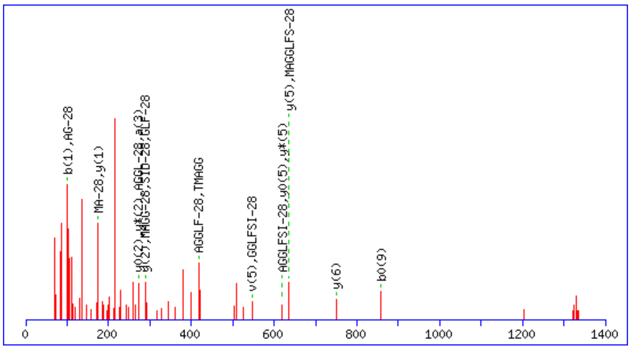

TPTMAGGLFSIDR; 15

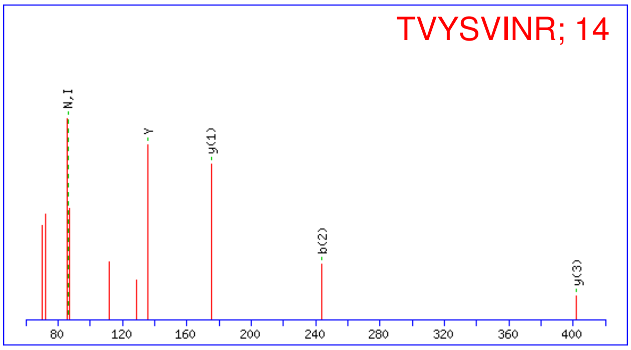

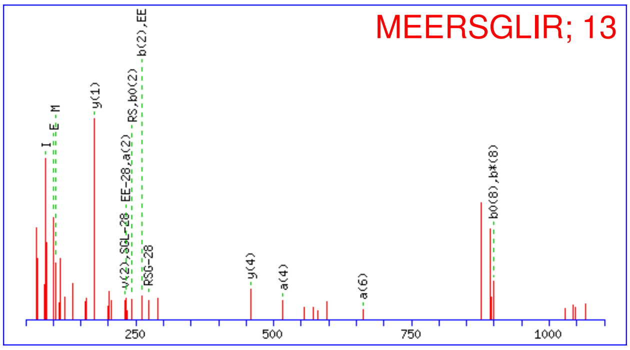

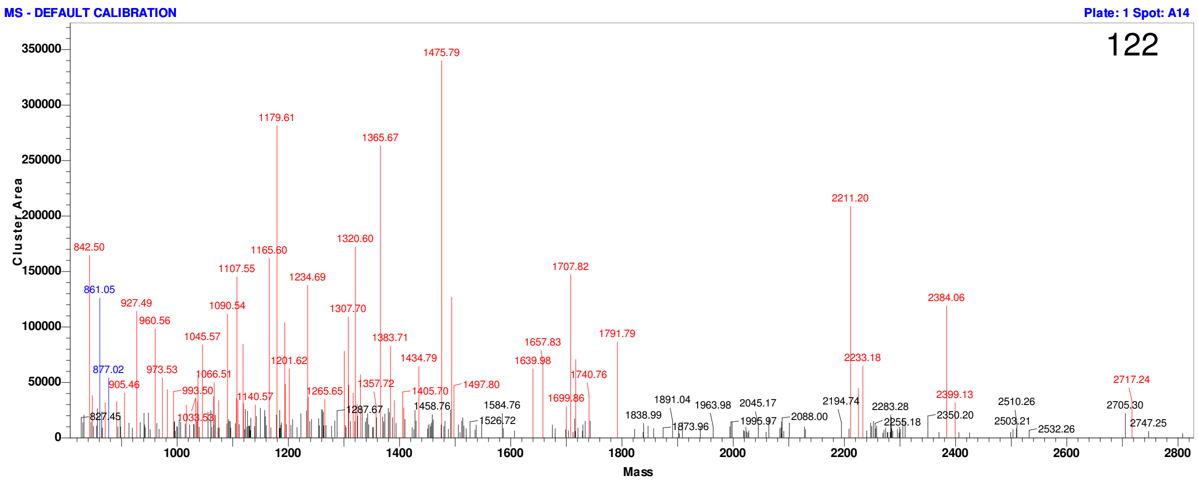

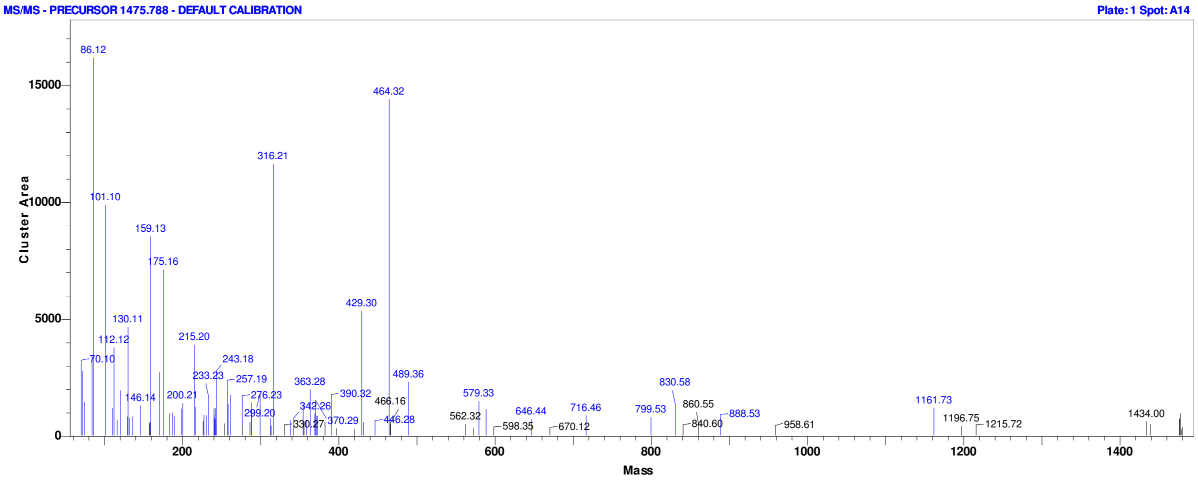

Nuclear receptor ROR-alpha

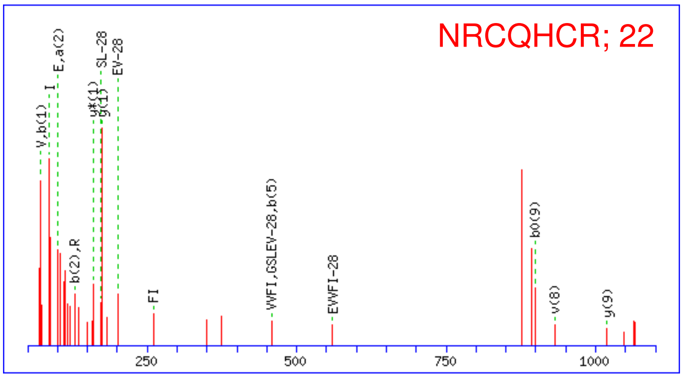

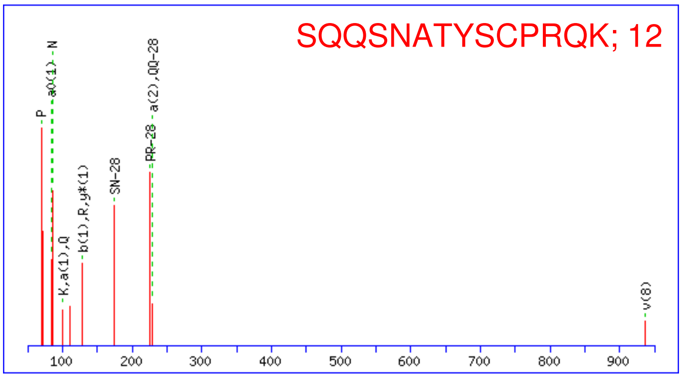

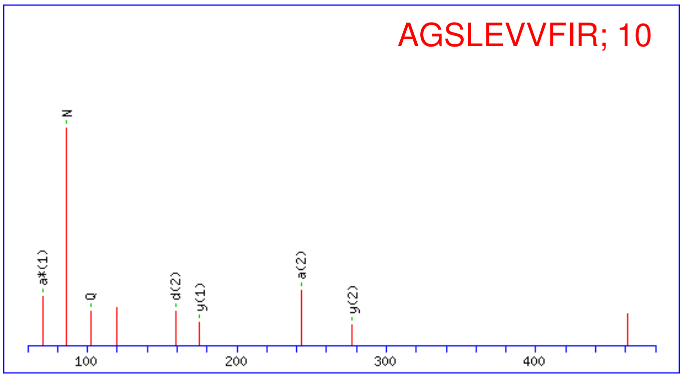

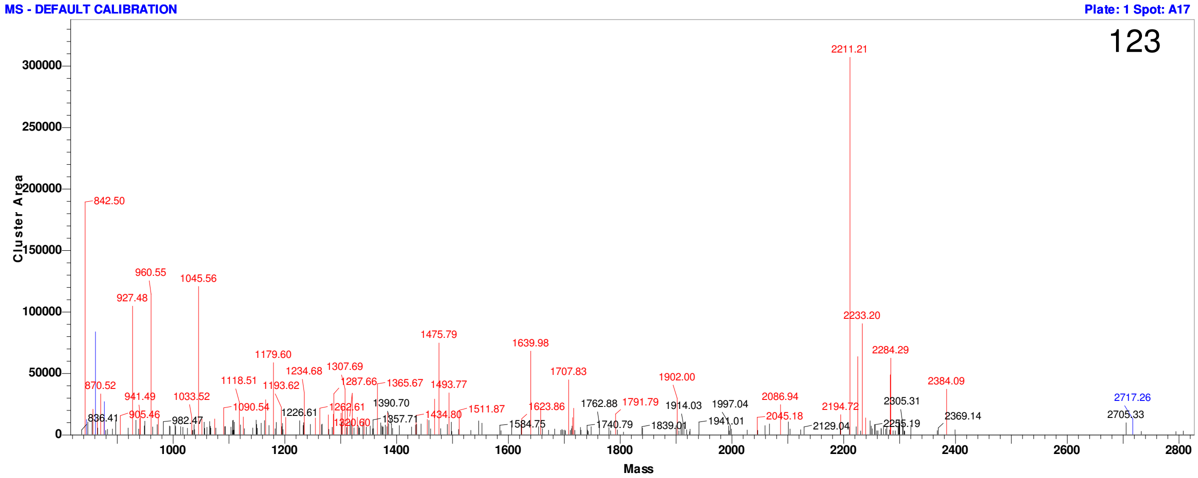
MS - DEFAULT CALIBRATION
Plate: 1 Spot: A17
123
Cluster Area
Mass
2211.21
842.50
960.55
1045.56
927.48
2233.20
2284.29
1475.79
1639.98
1179.60
1707.83
1307.69
1234.68
2384.09
1902.00
1118.51
1193.62
1287.66
1365.67
1493.77
870.52
941.49
1033.52
2086.94
2717.26
1390.70
1762.88
1791.79
1914.03
1997.04
2305.31
1090.54
1226.61
1262.61
1623.86
1511.87
2194.72
836.41
905.46
982.47
1357.71
1434.80
2045.18
2705.33
2369.14
1320.60
1584.75
1740.79
1839.01
1941.01
2129.04
2255.19

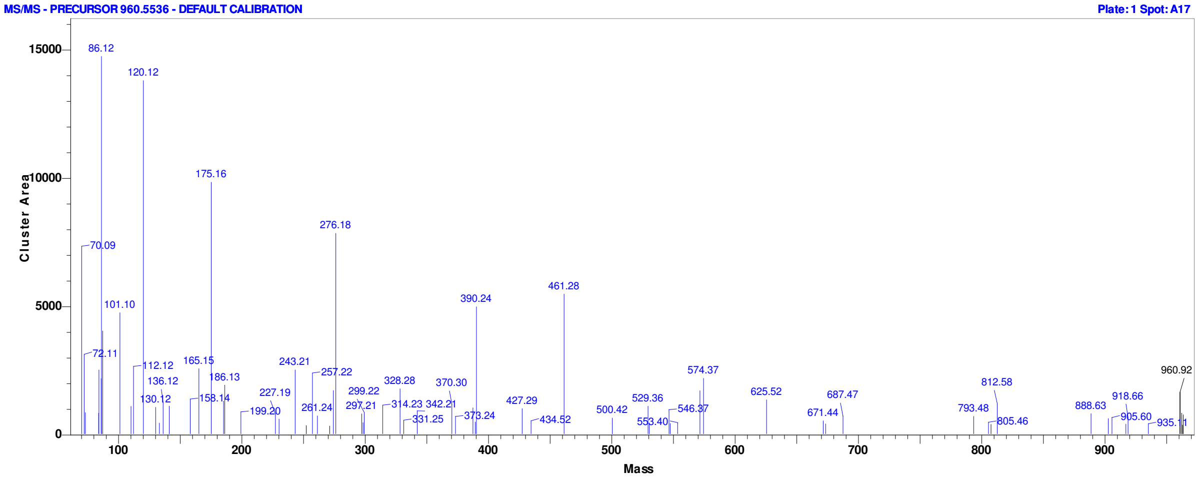
MS/MS - PRECURSOR 960.5536 - DEFAULT CALIBRATION
Plate: 1 Spot: A17
Cluster Area
Mass
86.12
120.12
175.16
70.09
276.18
461.28
390.24
101.10
72.11
243.21
165.15
112.12
257.22
186.13
136.12
574.37
328.28
370.30
227.19
299.22
625.52
687.47
812.58
918.66
960.92
130.12
158.14
261.24
297.21
314.23
342.21
427.29
529.36
199.20
331.25
373.24
434.52
500.42
546.37
553.40
671.44
793.48
888.63
805.46
905.60
935.11

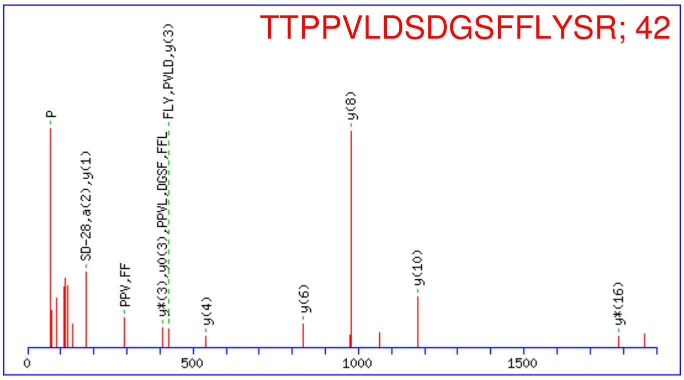
TTPPVLDSDGSFFLYSR; 42
P
SD-28,a(2),y(1)
PPV,FF
y*(3),y0(3),PPVL,DGSF,FFL
FLY,PVLD,y(3)
y(4)
y(6)
y(8)
y(10)
y*(16)

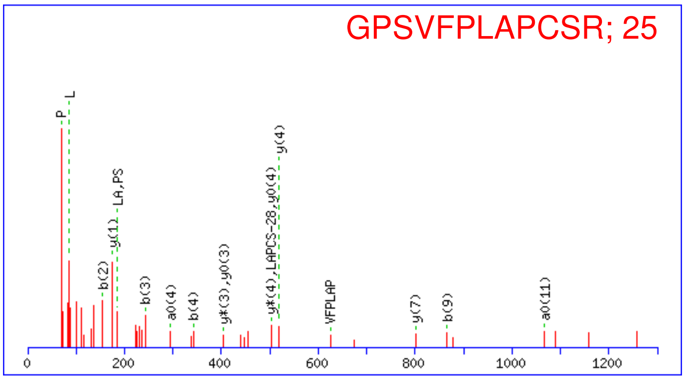
GPSVFPLAPCSR; 25
P
L
b(2)
y(1)
LA,PS
b(3)
a0(4)
b(4)
y*(3),y0(3)
y*(4),LAPCS-28,y0(4)
y(4)
VFPLAP
y(7)
b(9)
a0(11)

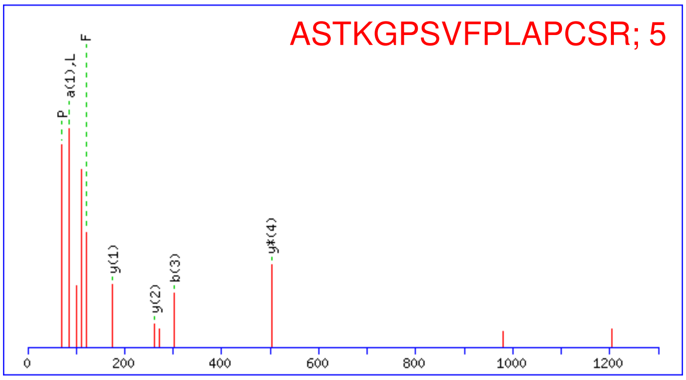
ASTKGPSVFPLAPCSR; 5
P
a(1),L
F
y(1)
y(2)
b(3)
y*(4)

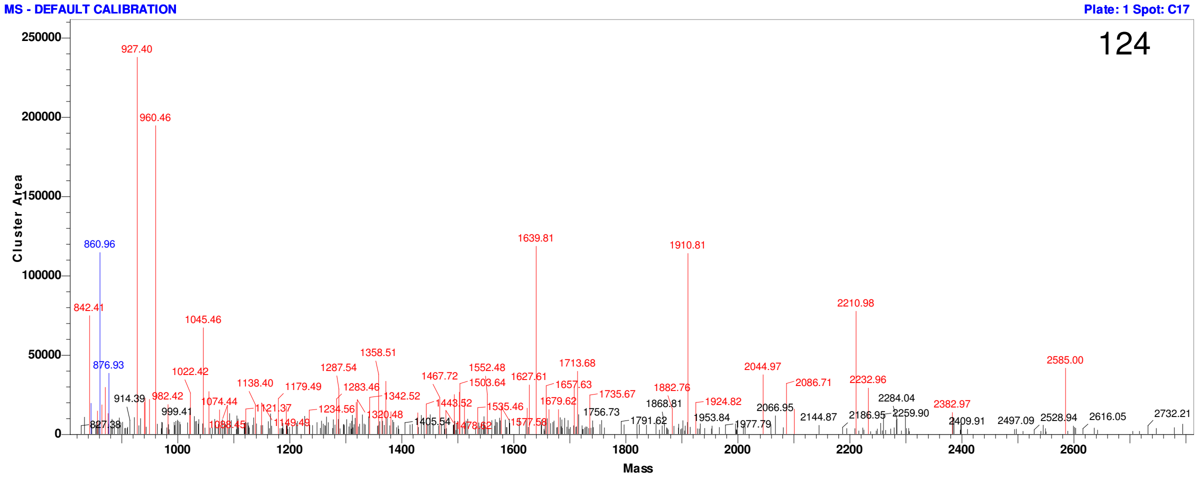
MS - DEFAULT CALIBRATION
Plate: 1 Spot: C17
124
Cluster Area
Mass

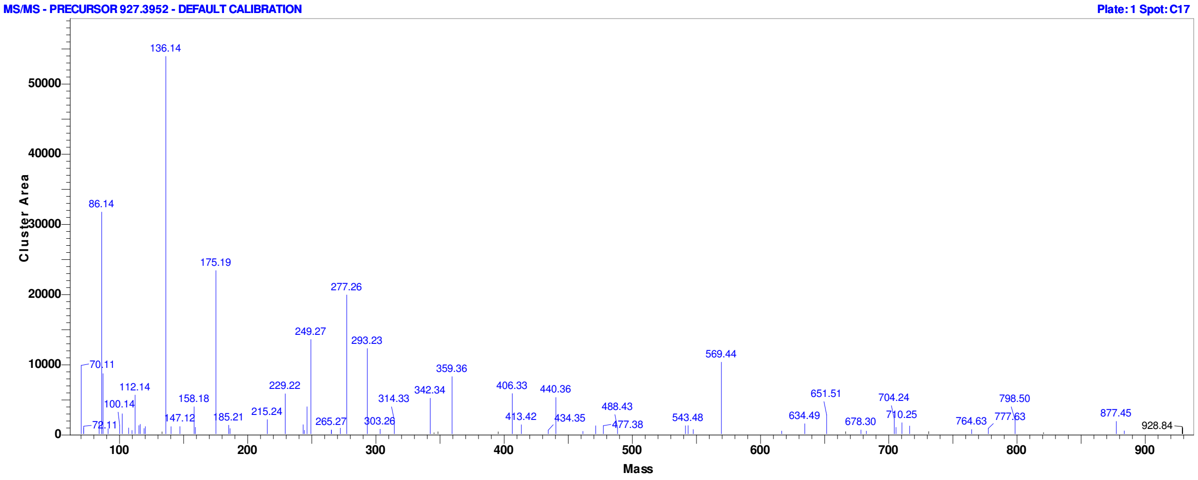
MS/MS - PRECURSOR 927.3952 - DEFAULT CALIBRATION
Plate: 1 Spot: C17
Cluster Area
Mass

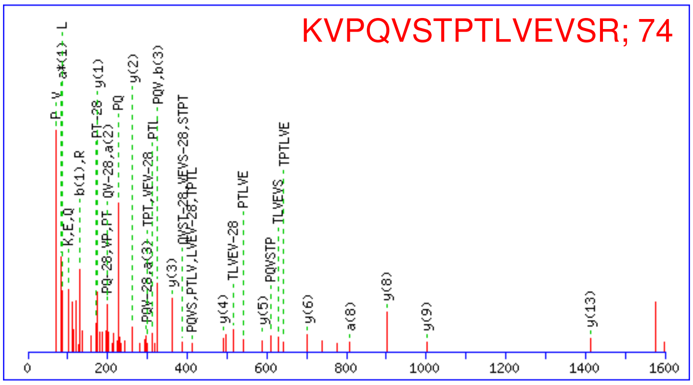
KVPQVSTPTLVEVSR; 74

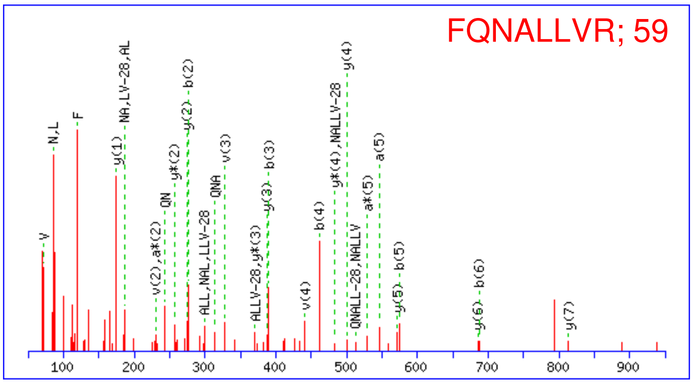
FQNALLVR; 59

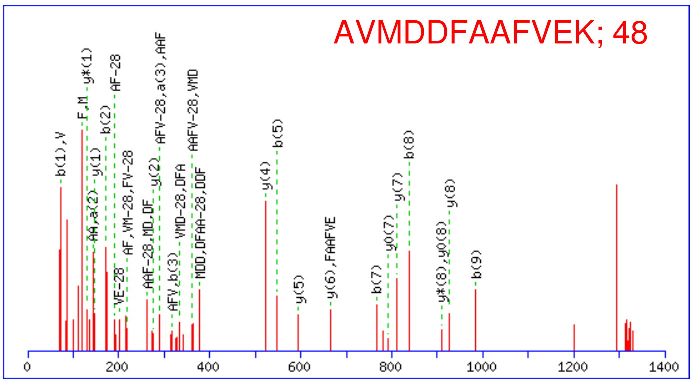
AVMDDFAAFVEK; 48

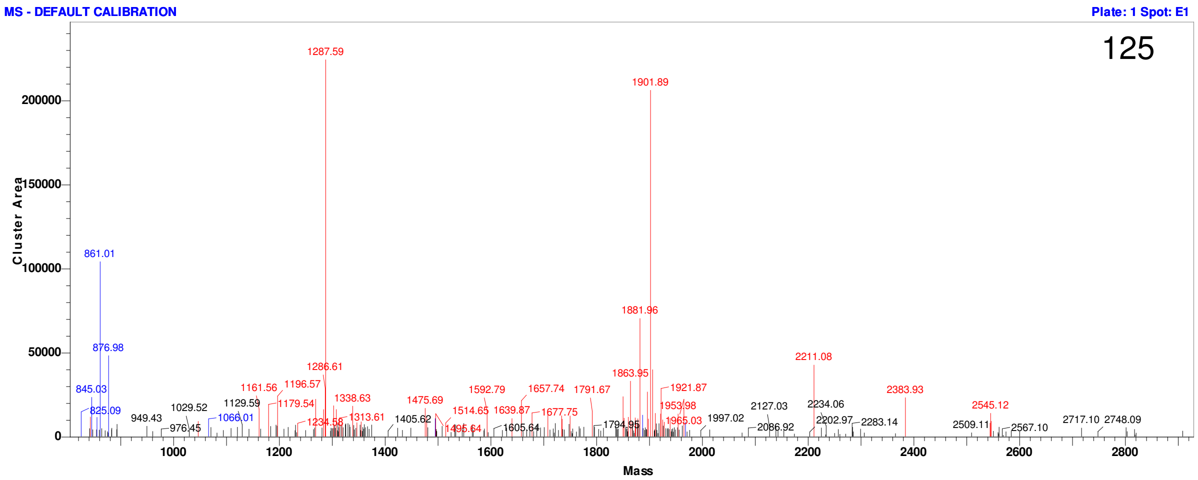
MS - DEFAULT CALIBRATION
Plate: 1 Spot: E1
125
Cluster Area
Mass
1287.59
1901.89
1881.96
861.01
876.98
2211.08
1286.61
1863.95
2383.93
2545.12

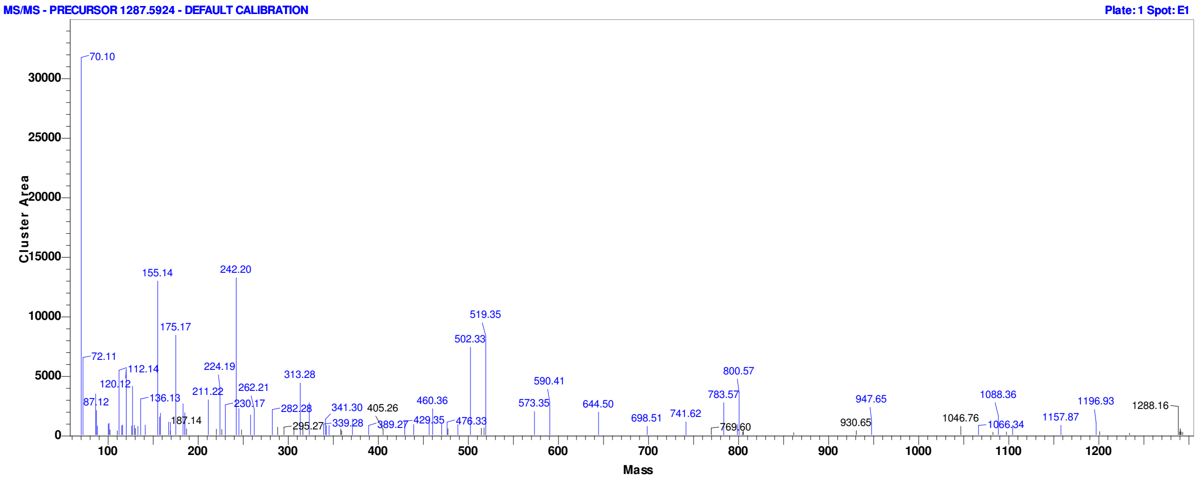
MS/MS - PRECURSOR 1287.5924 - DEFAULT CALIBRATION
Plate: 1 Spot: E1
Cluster Area
Mass
70.10
155.14
242.20
175.17
519.35
502.33
800.57
590.41
1088.36
1196.93
1288.16

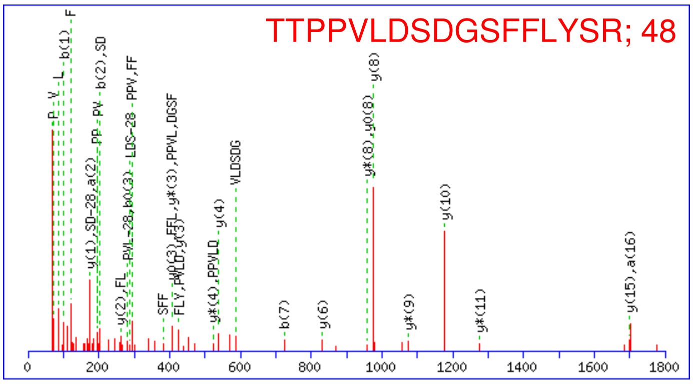
TTPPVLDSDGSFFLYSR; 48

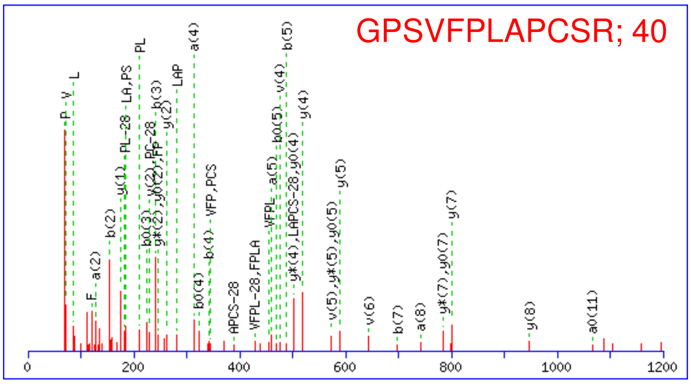
GPSVFPLAPCSR; 40

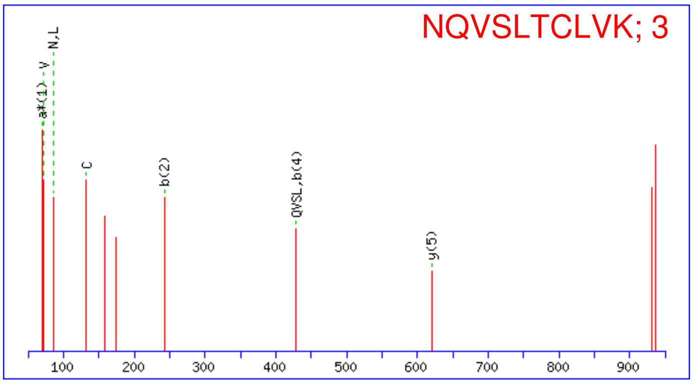
NQVSLTCLVK; 3

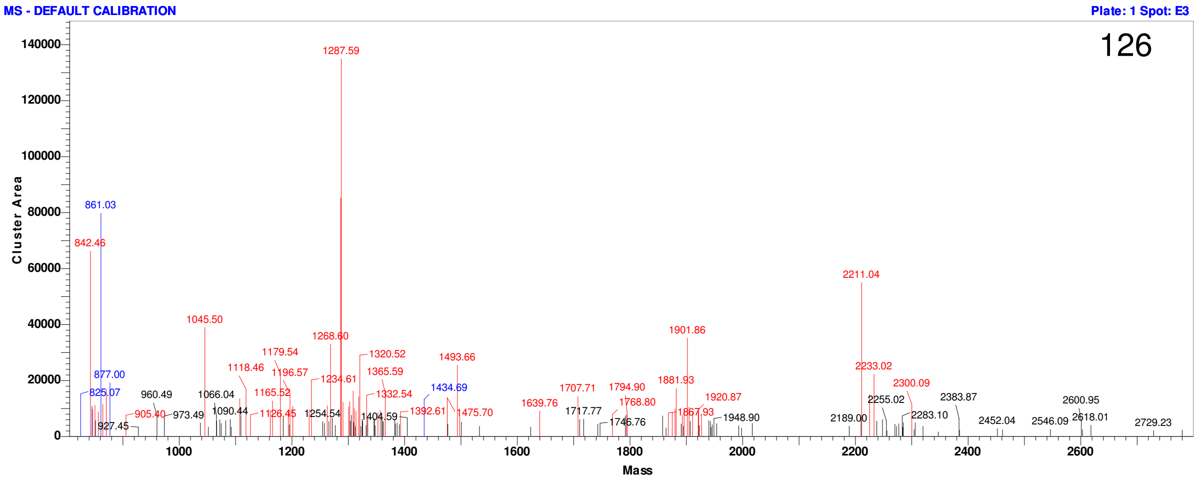
MS - DEFAULT CALIBRATION
Plate: 1 Spot: E3
126
Cluster Area
Mass

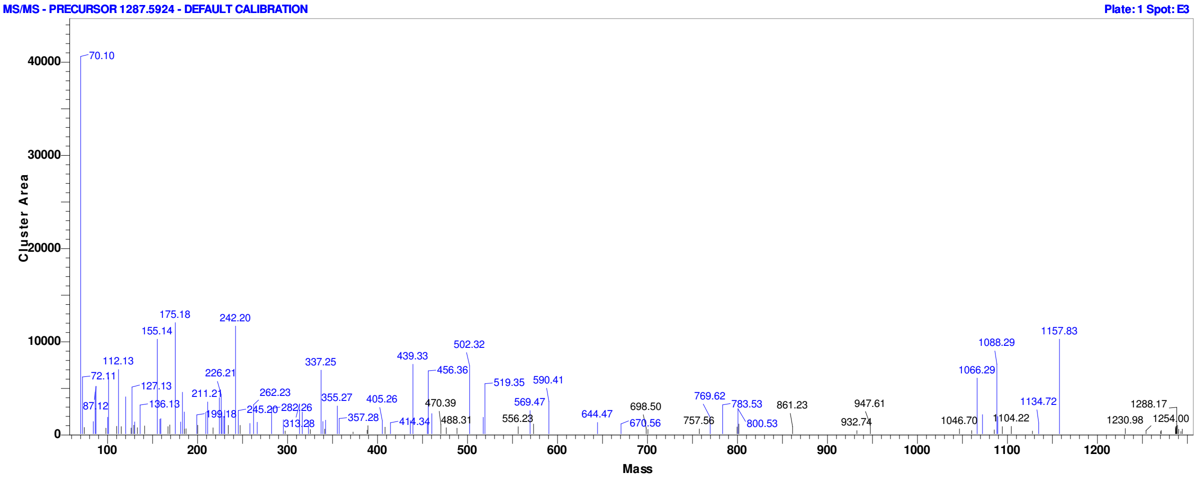
MS/MS - PRECURSOR 1287.5924 - DEFAULT CALIBRATION
Plate: 1 Spot: E3
Cluster Area
Mass

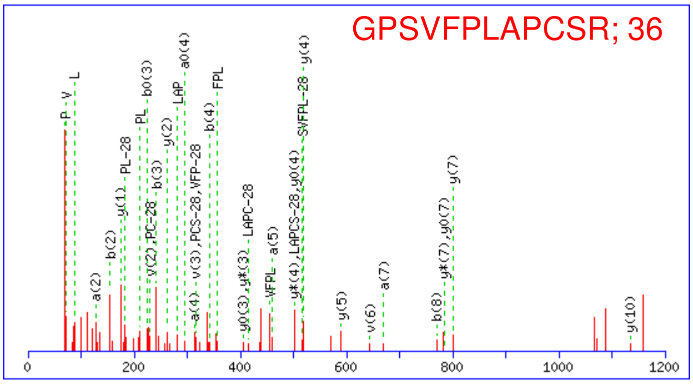
GPSVFPLAPCSR; 36

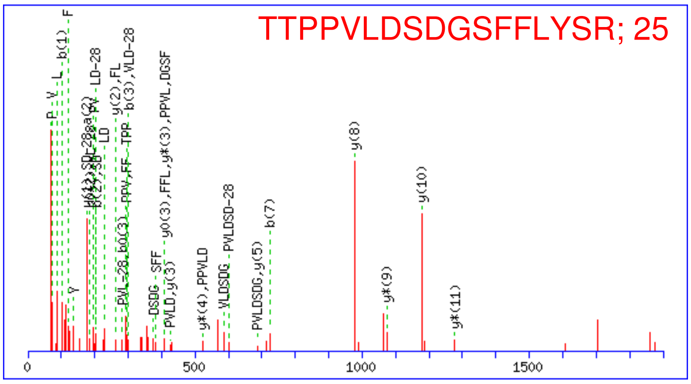
TTPPVLDSDGSFFLYSR; 25

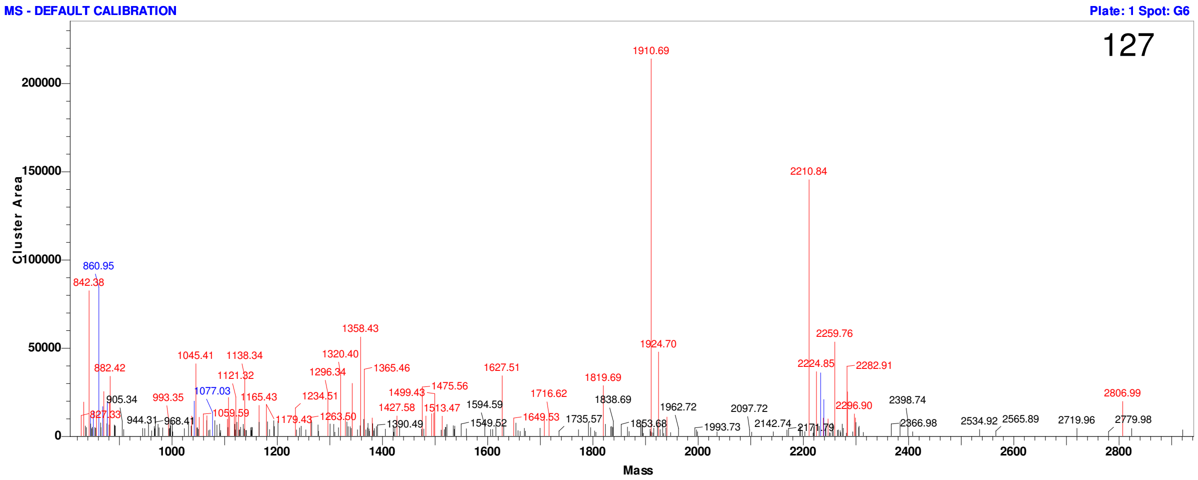

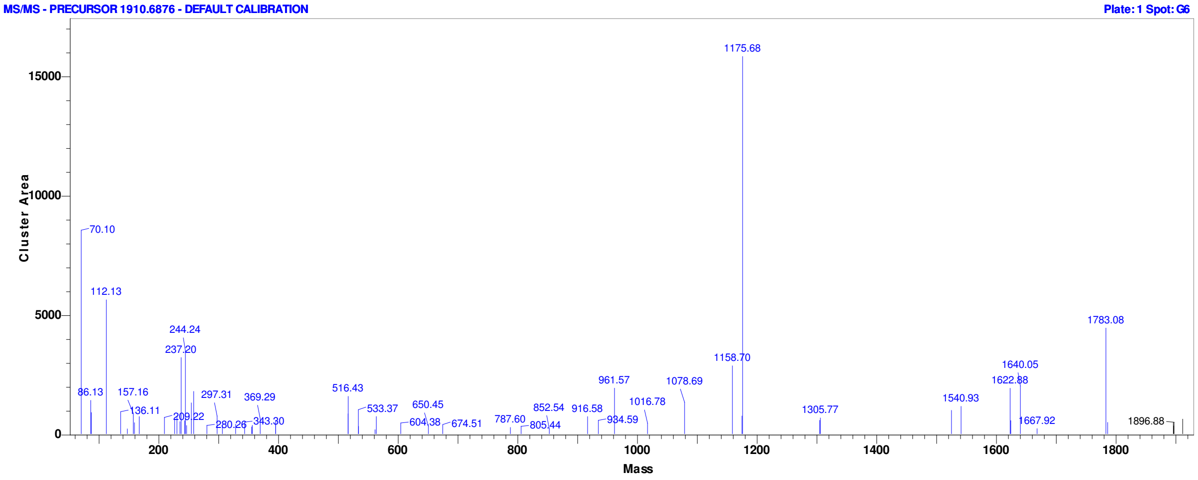

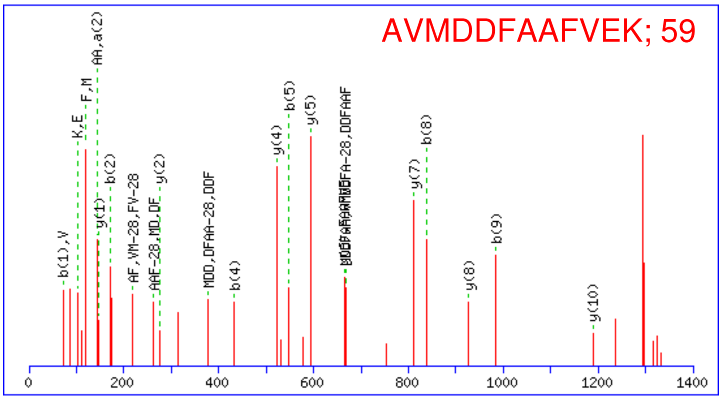

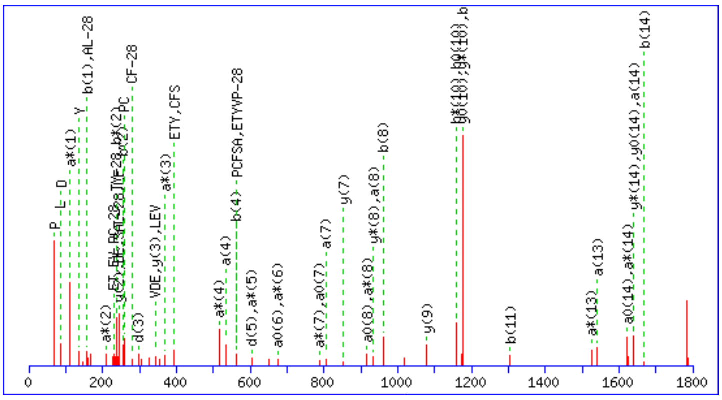

RPCFSALEVDETYVPK; 48

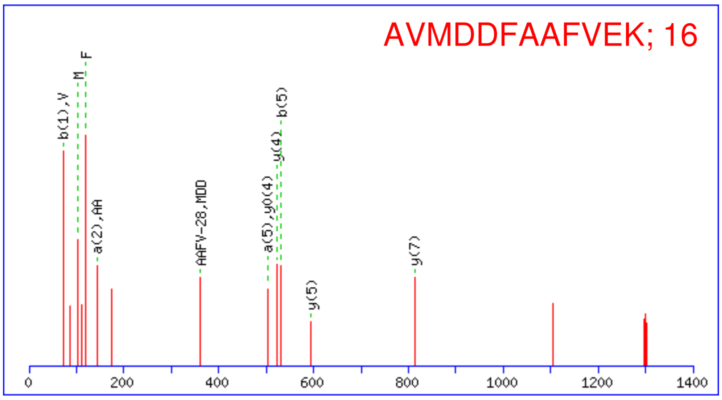

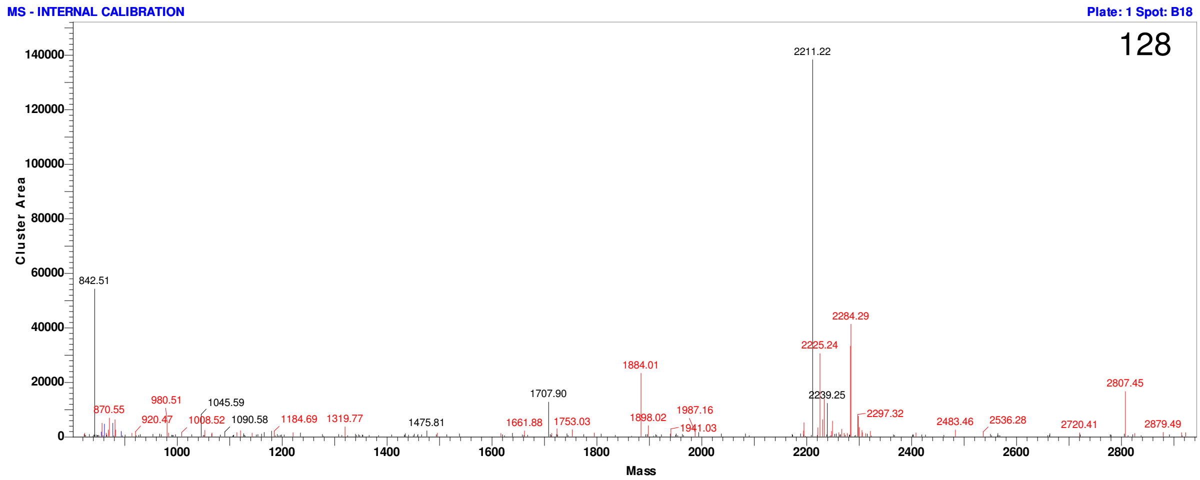
MS - INTERNAL CALIBRATION
Plate: 1 Spot: B18
128
Cluster Area
Mass
2211.22
842.51
2284.29
2225.24
1884.01
2807.45
870.55
980.51
1045.59
920.47
1008.52
1090.58
1184.69
1319.77
1475.81
1707.90
1661.88
1753.03
1898.02
1987.16
1941.03
2239.25
2297.32
2483.46
2536.28
2720.41
2879.49

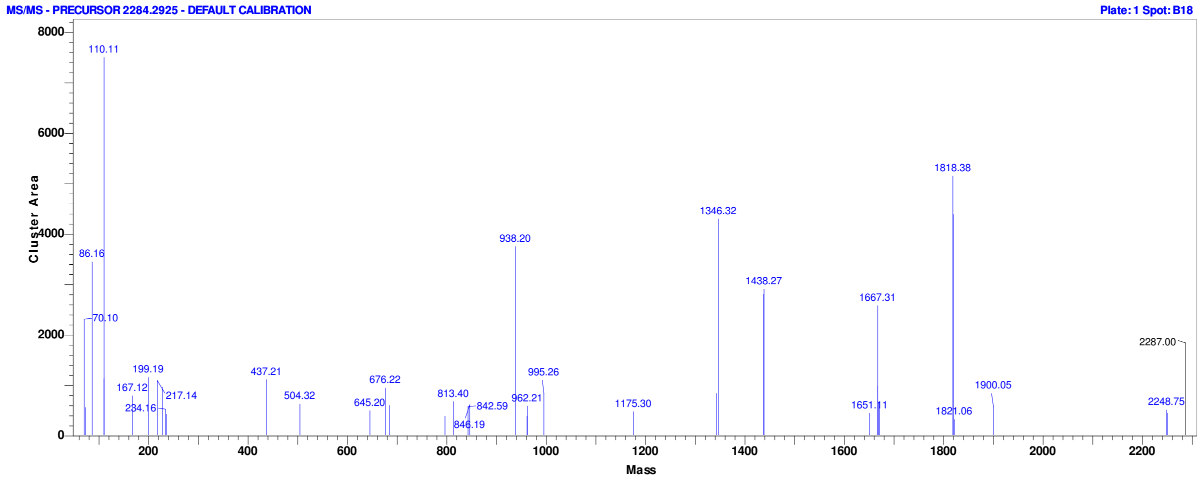
MS/MS - PRECURSOR 2284.2925 - DEFAULT CALIBRATION
Plate: 1 Spot: B18
Cluster Area
Mass
110.11
86.16
70.10
199.19
167.12
217.14
234.16
437.21
504.32
676.22
645.20
813.40
842.59
846.19
938.20
962.21
995.26
1175.30
1346.32
1438.27
1667.31
1651.11
1818.38
1821.06
1900.05
2287.00
2248.75

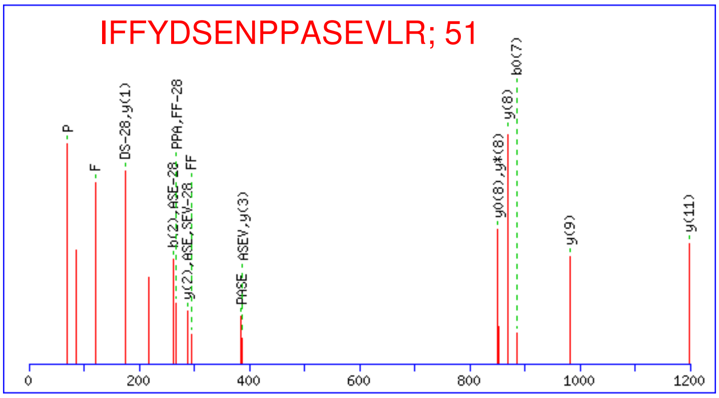
IFFYDSENPPASEVLR; 51
P
F
DS-28,y(1)
b(2),ASE-28
PPA,FF-28
y(2),ASE,SEV-28
FF
PASE
ASEV,y(3)
y0(8),y*(8)
y(8)
b0(7)
y(9)
y(11)

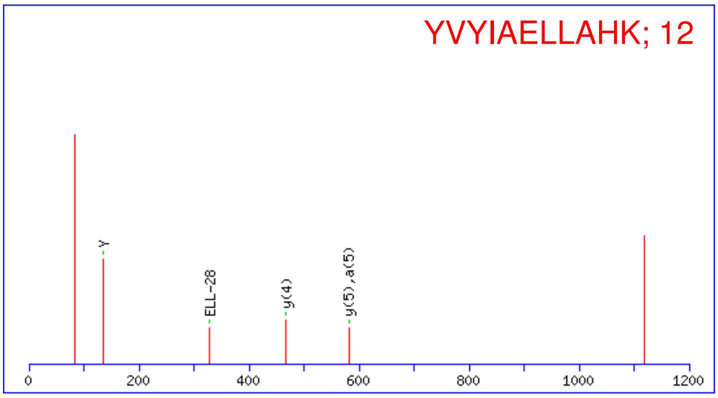
YVYIAELLAHK; 12
Y
ELL-28
y(4)
y(5),a(5)

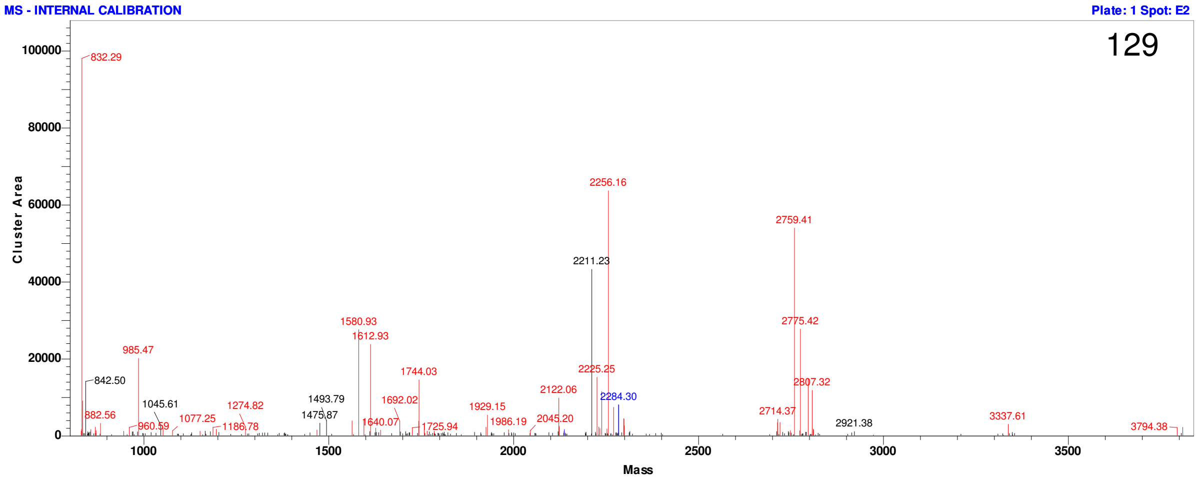
MS - INTERNAL CALIBRATION
Plate: 1 Spot: E2
129
Cluster Area
Mass
832.29
842.50
882.56
960.59
985.47
1045.61
1077.25
1186.78
1274.82
1475.87
1493.79
1580.93
1612.93
1640.07
1692.02
1725.94
1744.03
1929.15
1986.19
2045.20
2122.06
2211.23
2225.25
2256.16
2284.30
2714.37
2759.41
2775.42
2807.32
2921.38
3337.61
3794.38

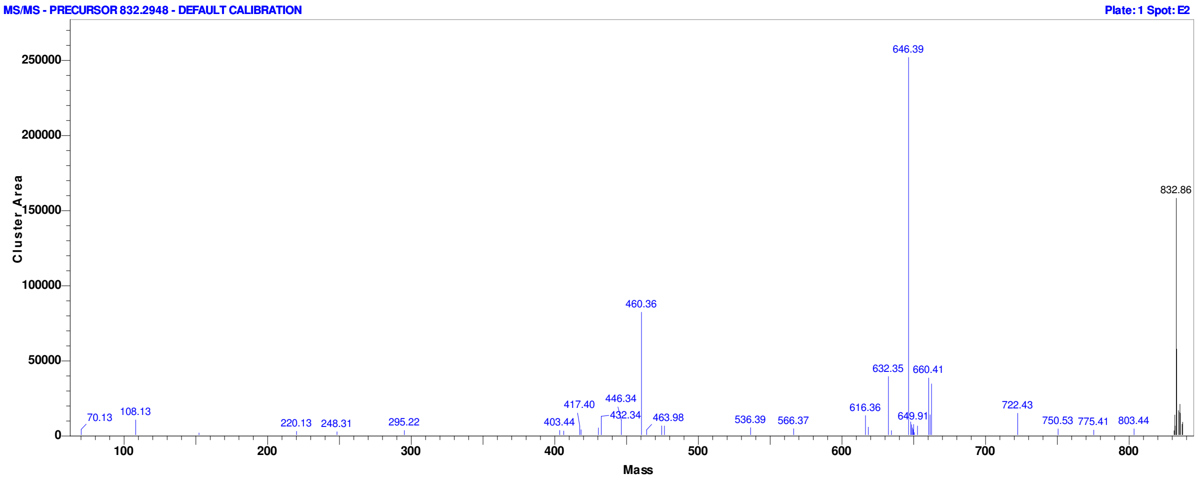
MS/MS - PRECURSOR 832.2948 - DEFAULT CALIBRATION
Plate: 1 Spot: E2
Cluster Area
Mass
70.13
108.13
220.13
248.31
295.22
403.44
417.40
432.34
446.34
460.36
463.98
536.39
566.37
616.36
632.35
646.39
649.91
660.41
722.43
750.53
775.41
803.44
832.86

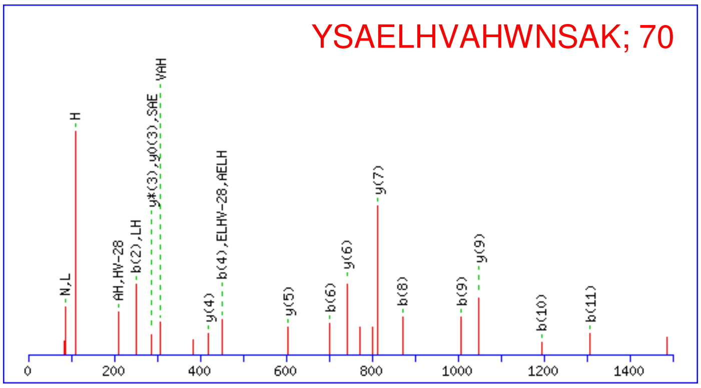
YSAELHVAHWNSAK; 70

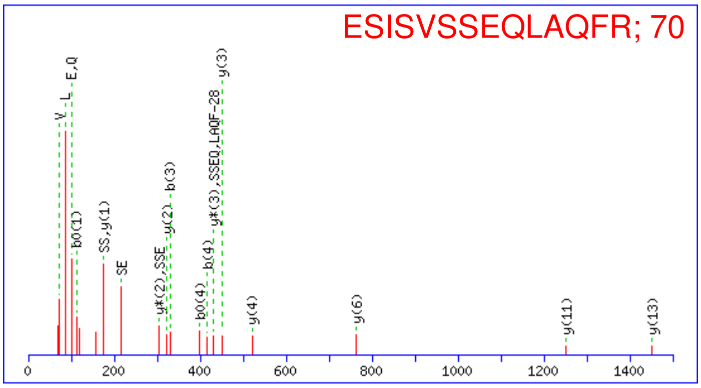
ESISVSSEQLAQFR; 70

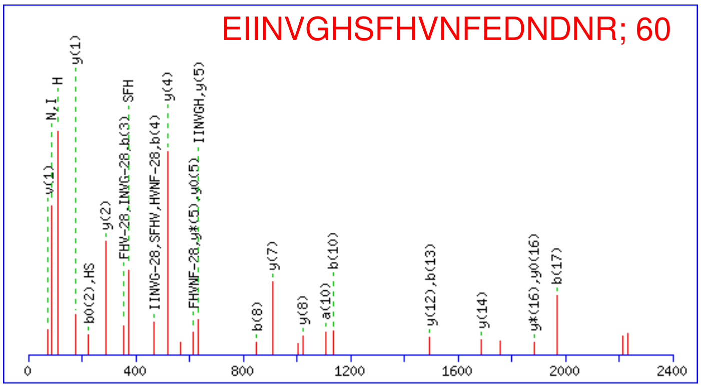
EIINVGHSFHVNFEDNDNR; 60

Figure-S2A

MS and MSMS spectra for all the identified spots by MALDI. GPS Explorer™ version 3.6 was used for analysis of spectral data. MASCOT database scoring algorithm and NCBI and Swiss-Prot protein databases were used for peptide identification (see Materials and Methods). Each spot number is mentioned in black at the top right hand of the MS spectrum. MSMS spectra of peptide fragments with top three ion scores are mentioned below the MS spectrum in each page and for each spot. The peptide fragment followed by its ion score are mentioned in red in the MSMS spectrum pictures. For spots with more than one identified protein, the spectra appear in the same sequence as in Table-S1.

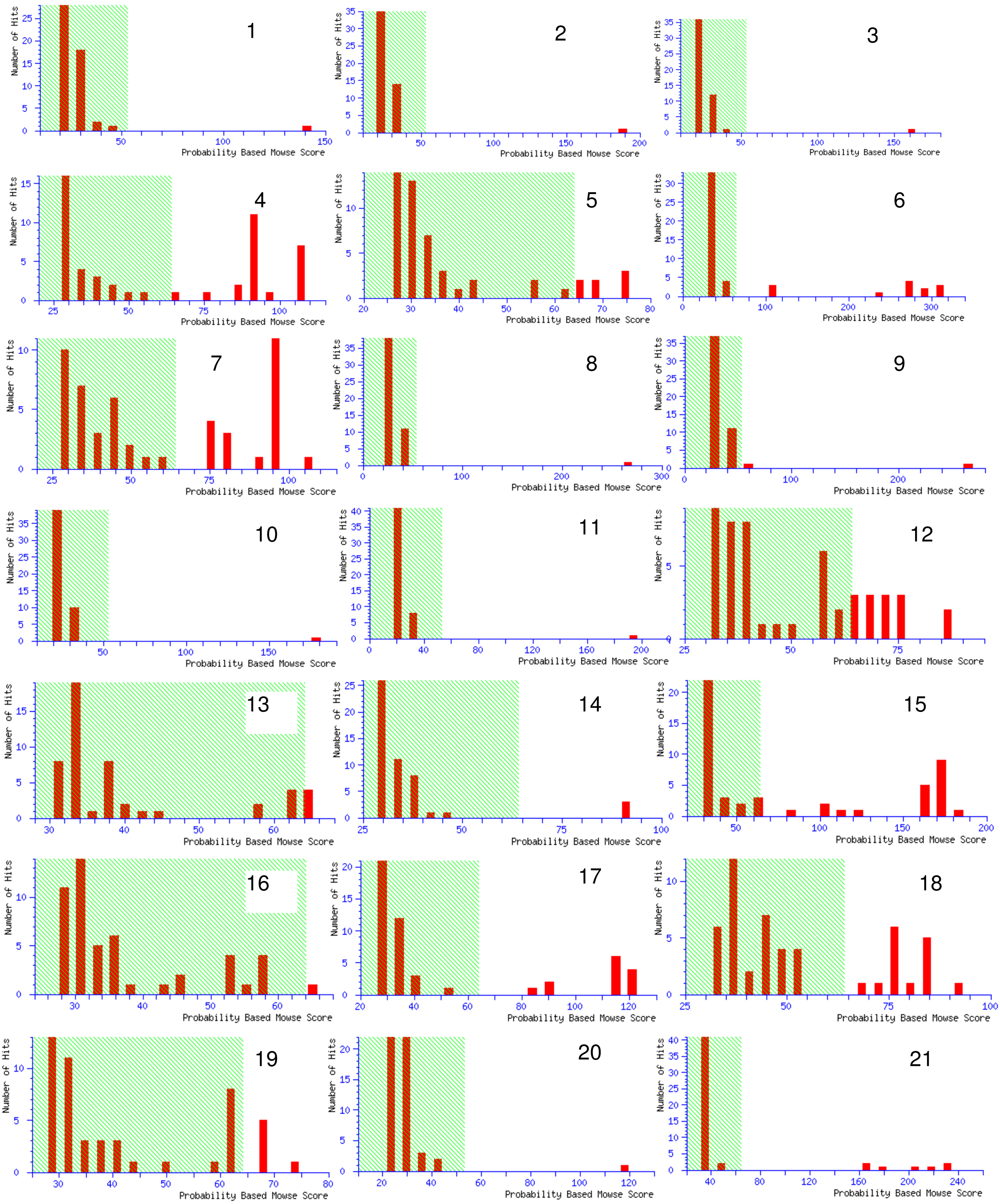
1
2
3
4
5
6
7
8
9
10
11
12
13
14
15
16
17
18
19
20
21
Number of Hits
Probability Based Mowse Score

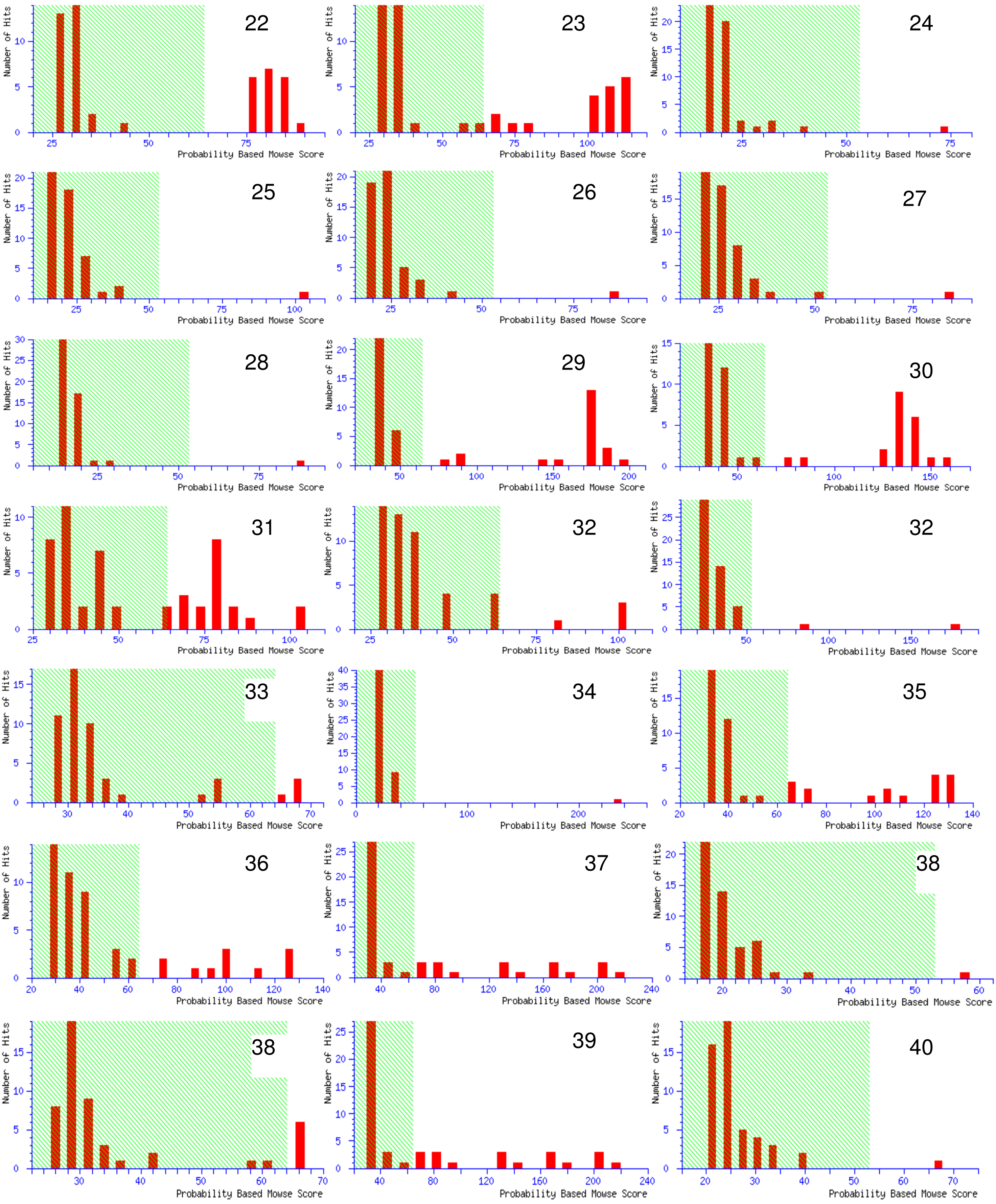
22
Number of Hits
Probability Based Mowse Score
23
Number of Hits
Probability Based Mowse Score
24
Number of Hits
Probability Based Mowse Score
25
Number of Hits
Probability Based Mowse Score
26
Number of Hits
Probability Based Mowse Score
27
Number of Hits
Probability Based Mowse Score
28
Number of Hits
Probability Based Mowse Score
29
Number of Hits
Probability Based Mowse Score
30
Number of Hits
Probability Based Mowse Score
31
Number of Hits
Probability Based Mowse Score
32
Number of Hits
Probability Based Mowse Score
32
Number of Hits
Probability Based Mowse Score
33
Number of Hits
Probability Based Mowse Score
34
Number of Hits
Probability Based Mowse Score
35
Number of Hits
Probability Based Mowse Score
36
Number of Hits
Probability Based Mowse Score
37
Number of Hits
Probability Based Mowse Score
38
Number of Hits
Probability Based Mowse Score
38
Number of Hits
Probability Based Mowse Score
39
Number of Hits
Probability Based Mowse Score
40
Number of Hits
Probability Based Mowse Score

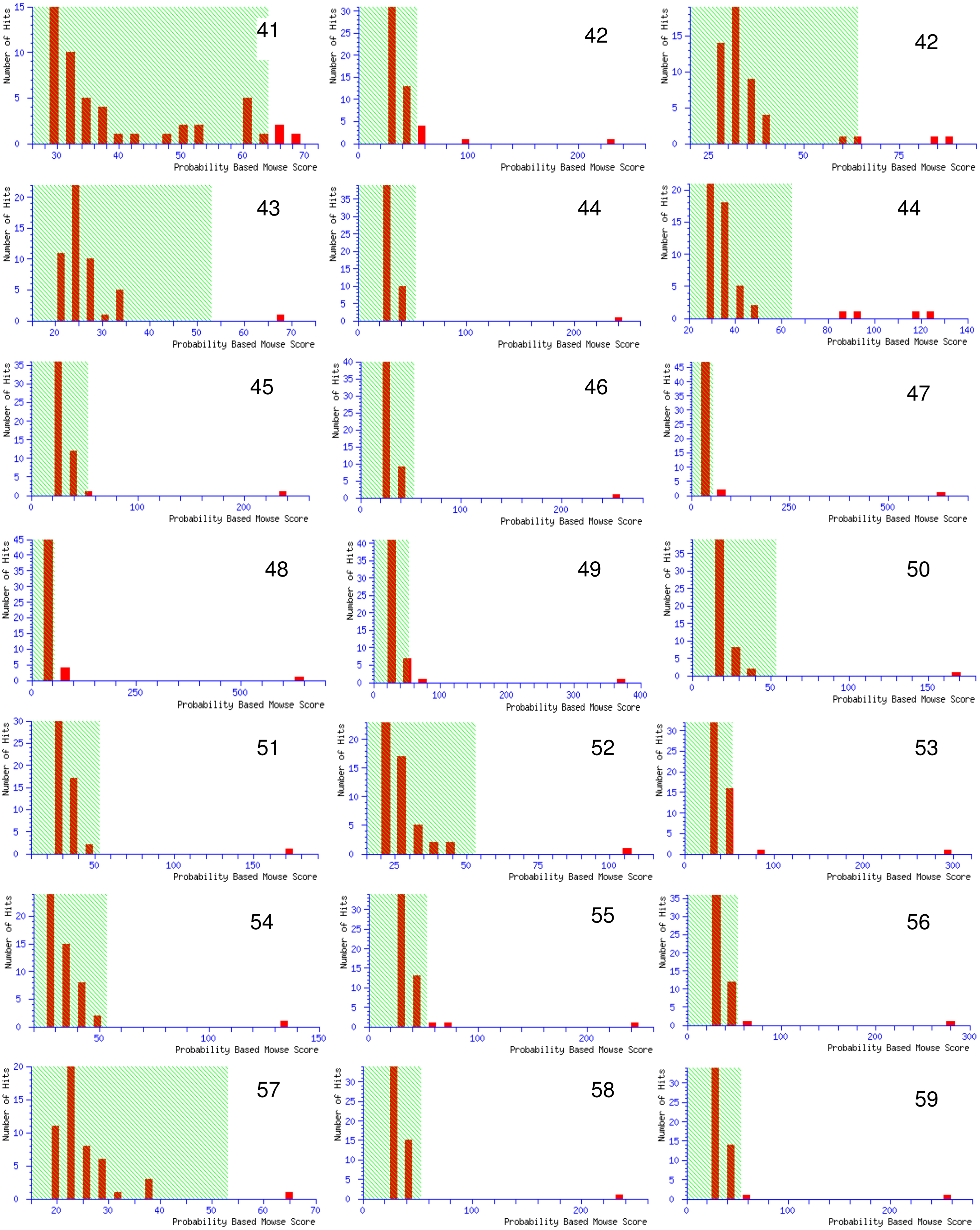
41
42
42
43
44
44
45
46
47
48
49
50
51
52
53
54
55
56
57
58
59
Number of Hits
Probability Based Mowse Score

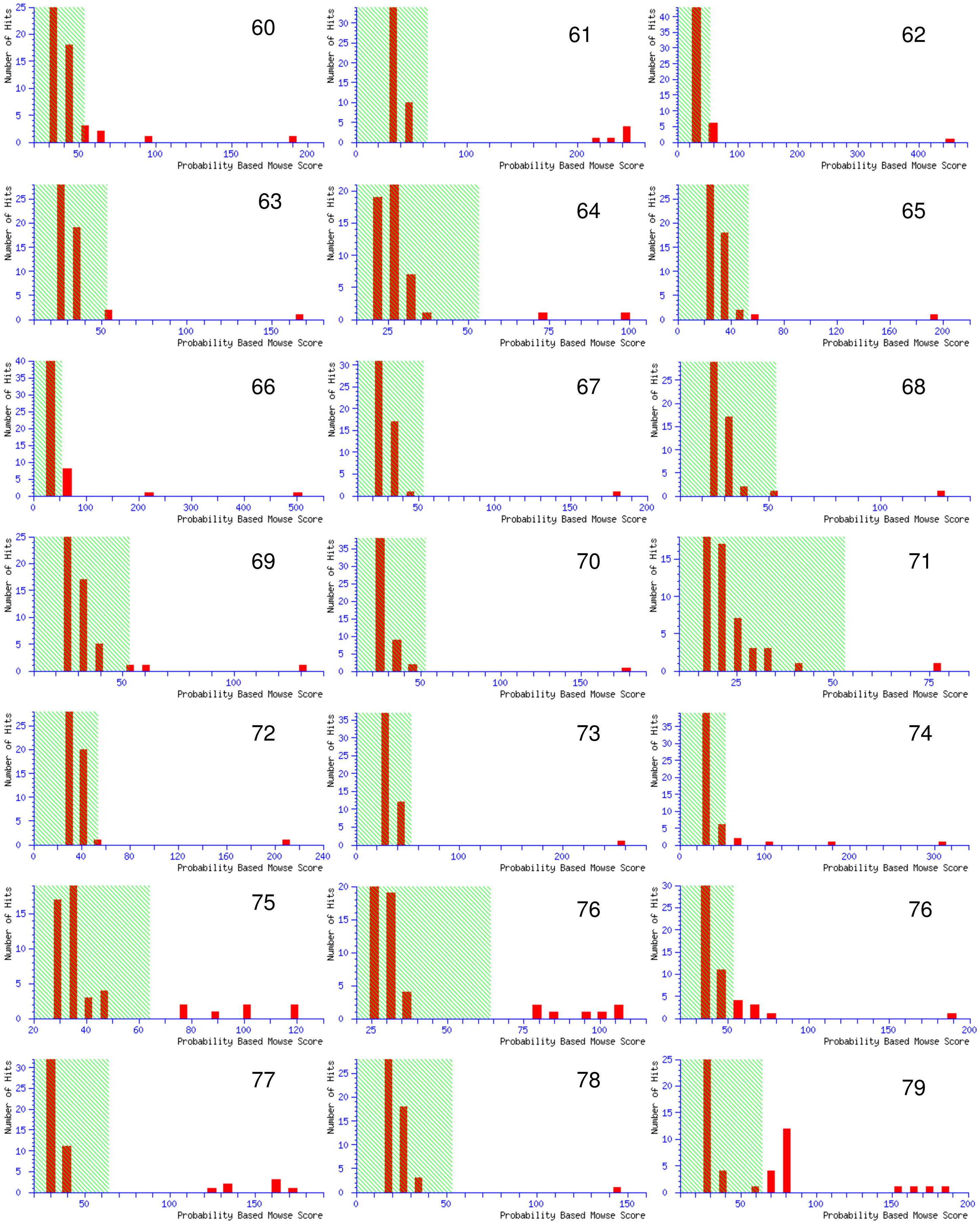

60
61
62
63
64
65
66
67
68
69
70
71
72
73
74
75
76
76
77
78
79
Number of Hits
Probability Based Mowse Score

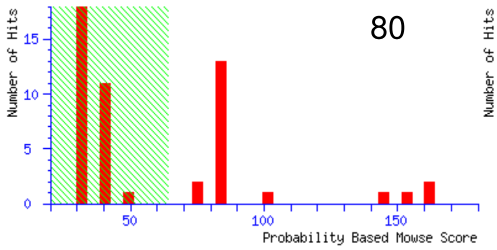
80
Number of Hits
Probability Based Mowse Score

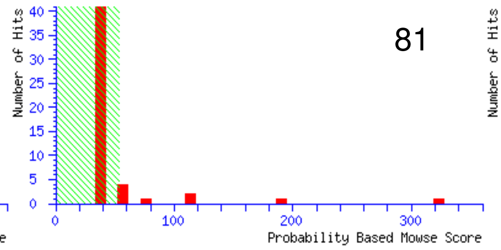
81
Number of Hits
Probability Based Mowse Score

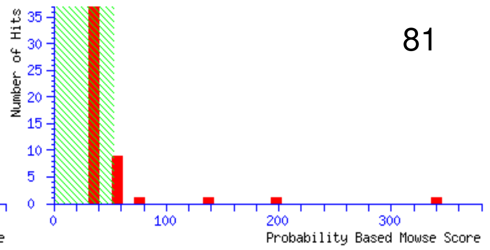
81
Number of Hits
Probability Based Mowse Score

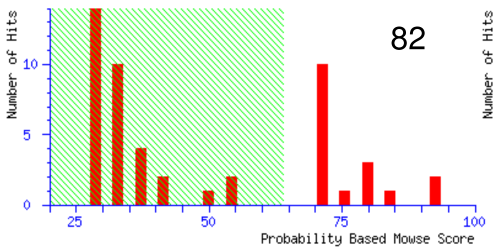
82
Number of Hits
Probability Based Mowse Score

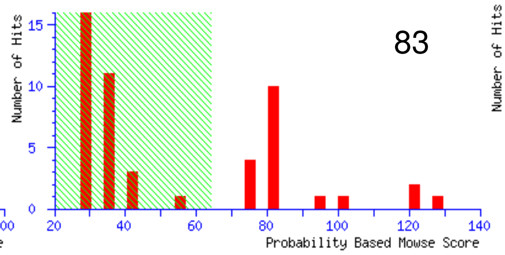
83
Number of Hits
Probability Based Mowse Score

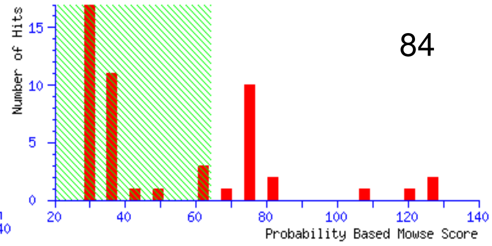
84
Number of Hits
Probability Based Mowse Score

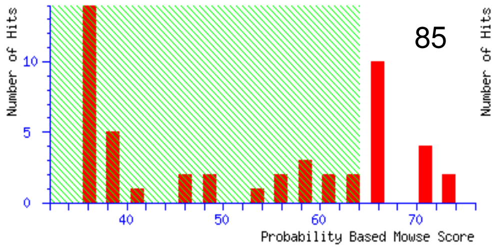
85
Number of Hits
Probability Based Mowse Score

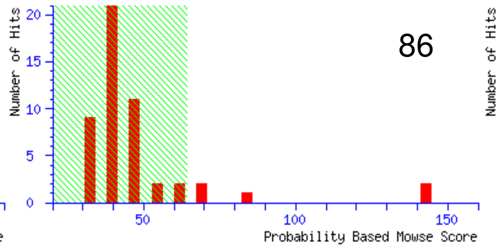
86
Number of Hits
Probability Based Mowse Score

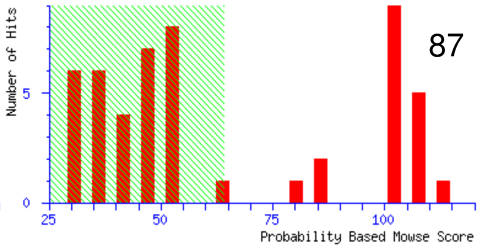
87
Number of Hits
Probability Based Mowse Score

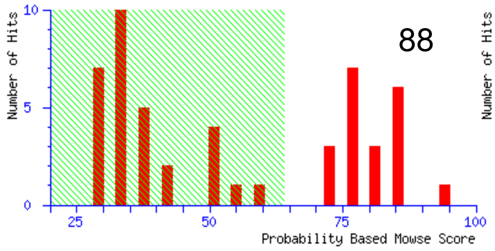
88
Number of Hits
Probability Based Mowse Score

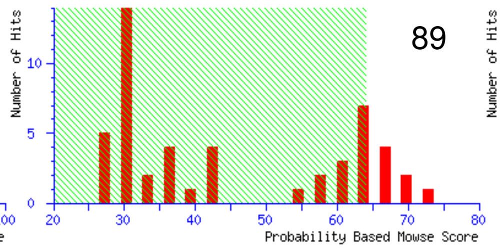
89
Number of Hits
Probability Based Mowse Score

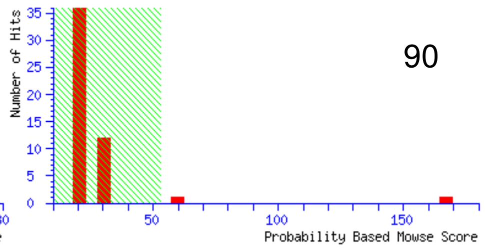
90
Number of Hits
Probability Based Mowse Score

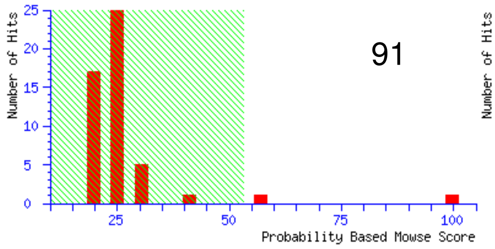
91
Number of Hits
Probability Based Mowse Score

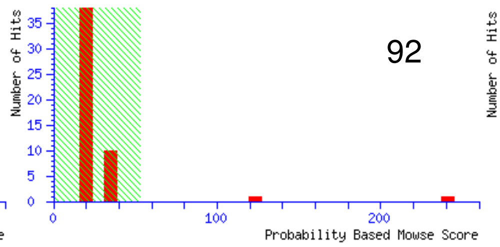
92
Number of Hits
Probability Based Mowse Score

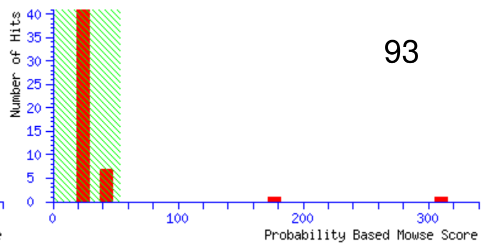
93
Number of Hits
Probability Based Mowse Score

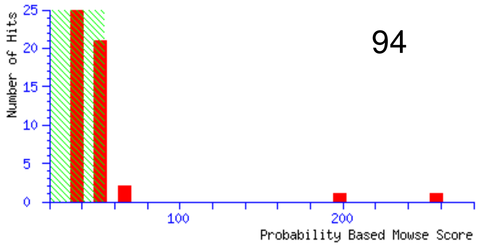
94
Number of Hits
Probability Based Mowse Score

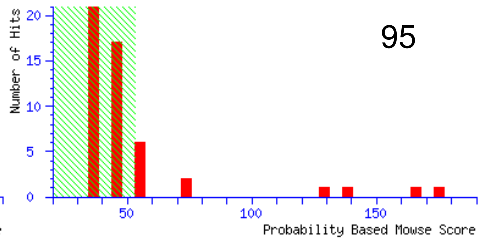
95
Number of Hits
Probability Based Mowse Score

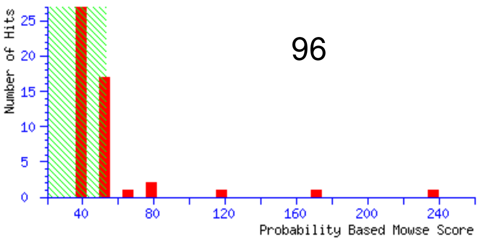
96
Number of Hits
Probability Based Mowse Score

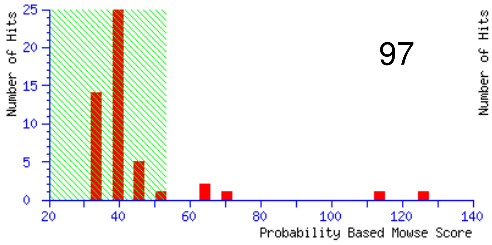
97
Number of Hits
Probability Based Mowse Score

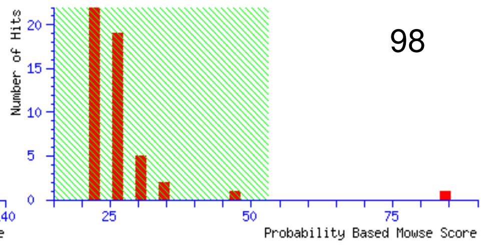
98
Number of Hits
Probability Based Mowse Score

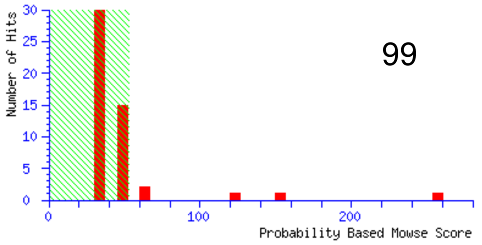
99
Number of Hits
Probability Based Mowse Score

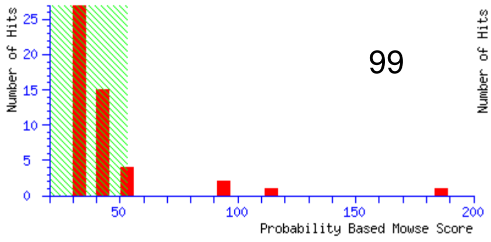
99
Number of Hits
Probability Based Mowse Score

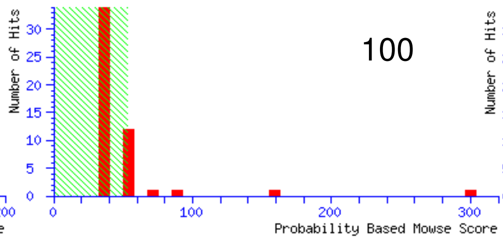
100
Number of Hits
Probability Based Mowse Score

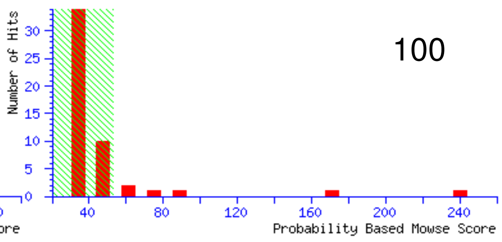
100
Number of Hits
Probability Based Mowse Score

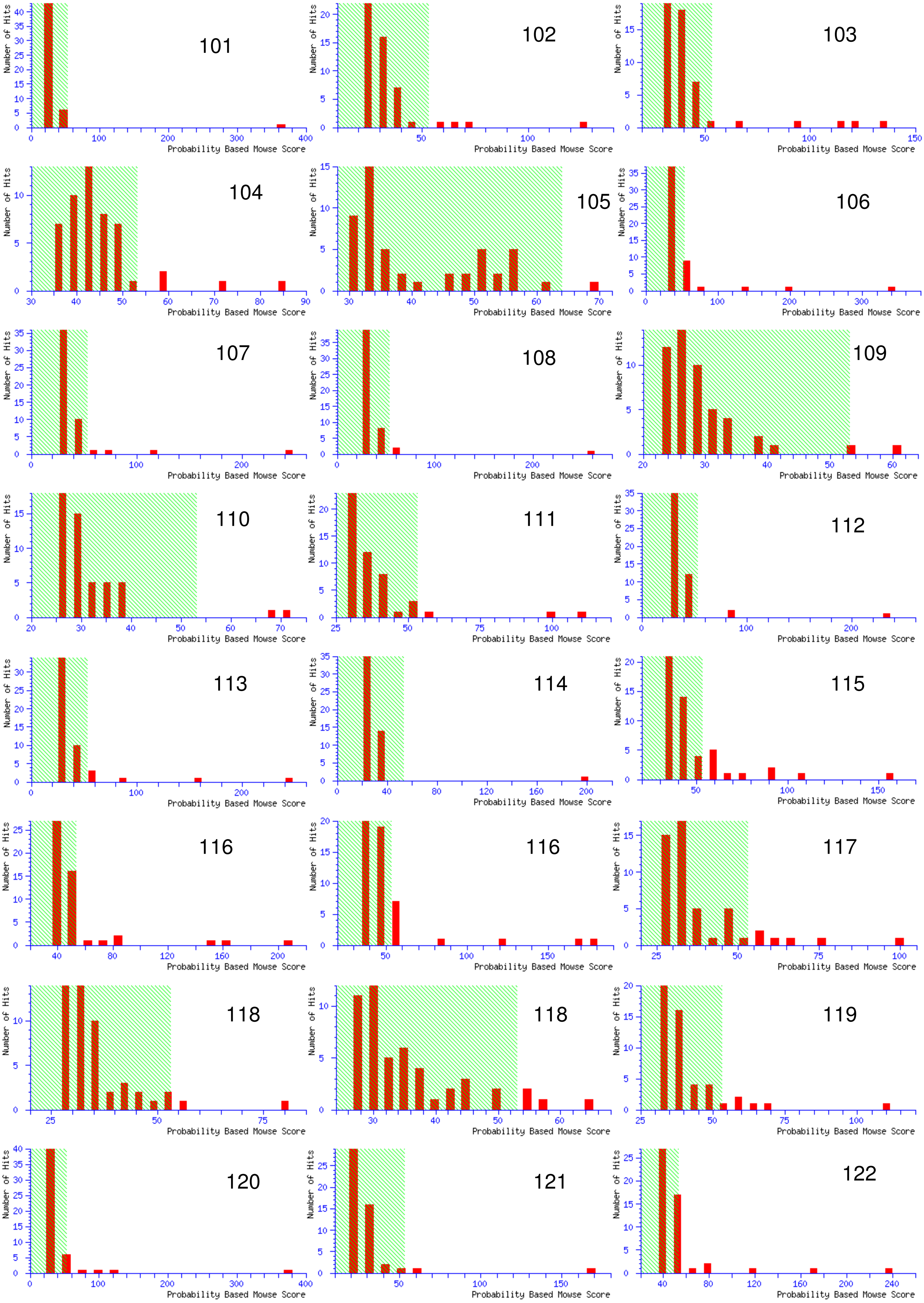
101
102
103
104
105
106
107
108
109
110
111
112
113
114
115
116
116
117
118
118
119
120
121
122
Number of Hits
Probability Based Mowse Score

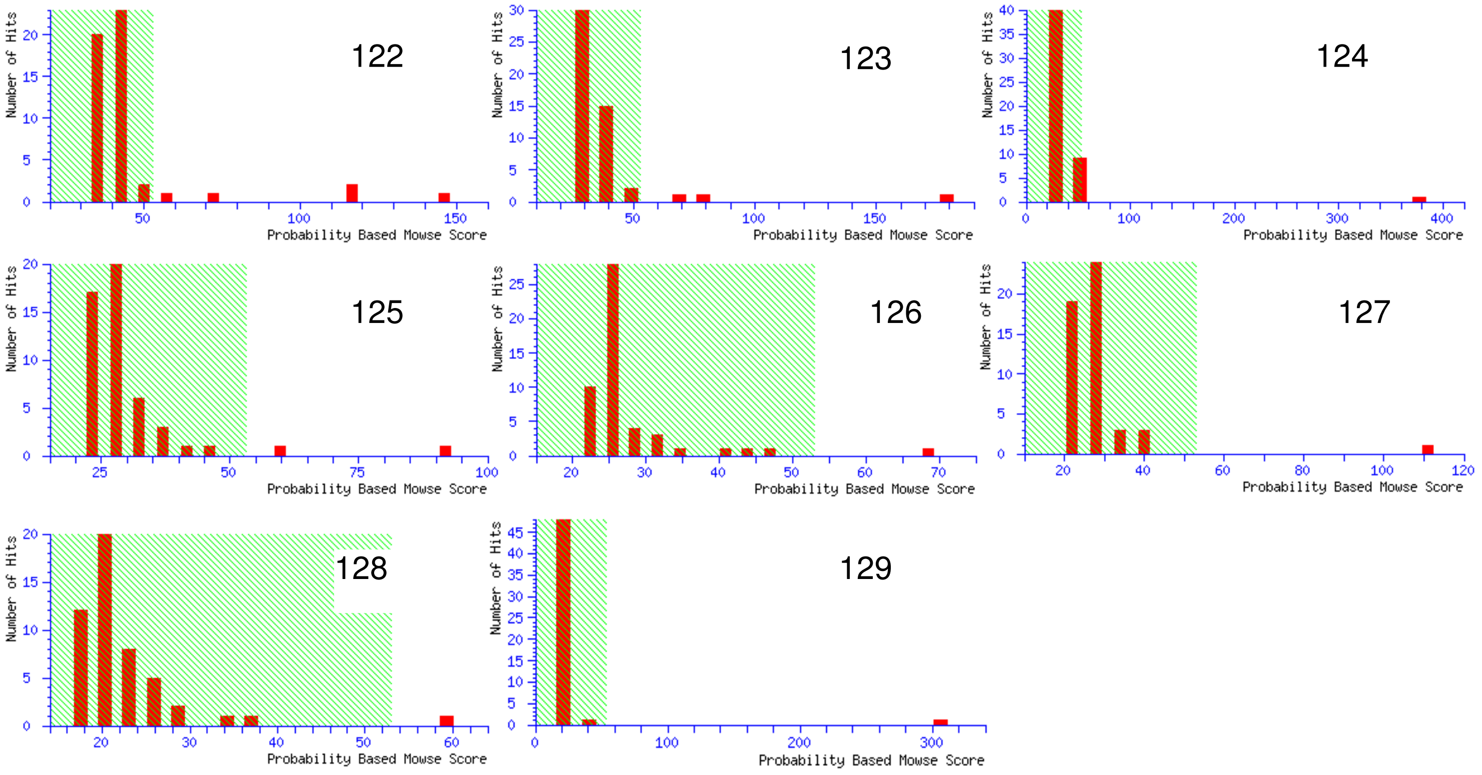

Figure-S2B

Probability based Mowse score graphs for all identified spots. The graphs were generated by MASCOT database scoring algorithm (Materials and Methods). The spot numbers are mentioned in black at the top right hand side of the score graphs. (See Table-S1 for details). For spots with multiple identified proteins, score graphs are provided in a sequential manner corresponding to their sequence in Table-S1. It must be noted that for the above mentioned spots, all redundant identifications and contaminant identifications like keratin, have been excluded.
