# Supplementary material for: CSF Proteomics of Secondary Phase Spinal Cord Injury in Human Subjects: Perturbed Molecular Pathways Post Injury
Source: PLoS One. 2014 Oct 28;9(10):e110885. doi: 10.1371/journal.pone.0110885 (PMC4211693; doi:10.1371/journal.pone.0110885)
Supplement: Figure S3 — Differential abundance ratios of the eight differentially abundant proteins from complete and incomplete injury SCI CSF. (PDF) [file pone.0110885.s003.pdf]

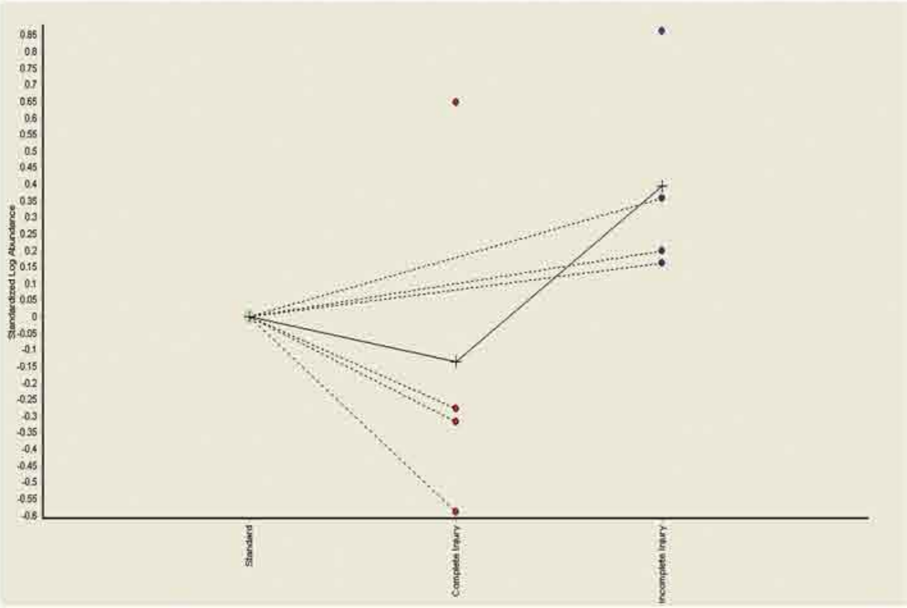

Spot No 26

Student’s t Test p Value: 0.0052

Appearance: 7/7

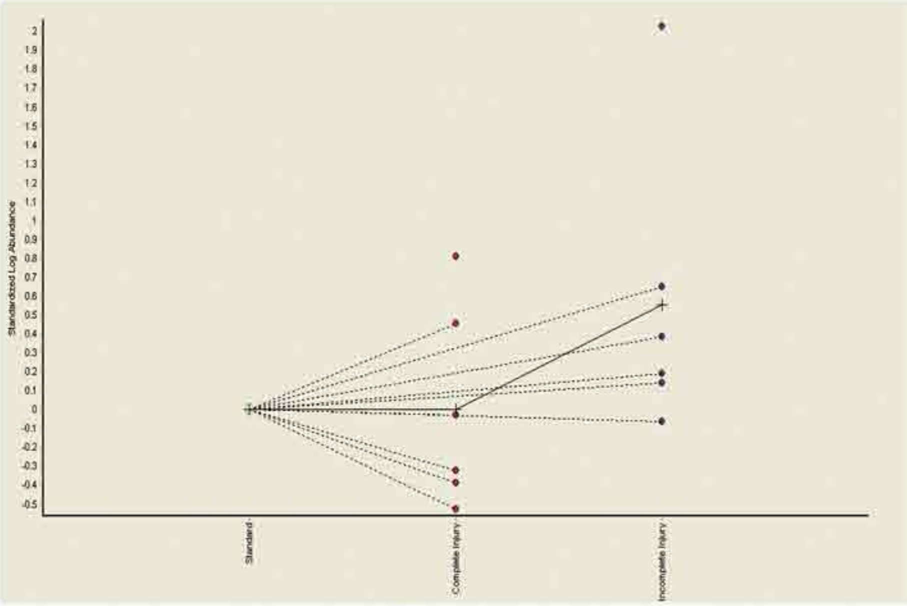

Spot No 27

Student’s t Test p Value: 0.082

Appearance: 6/7

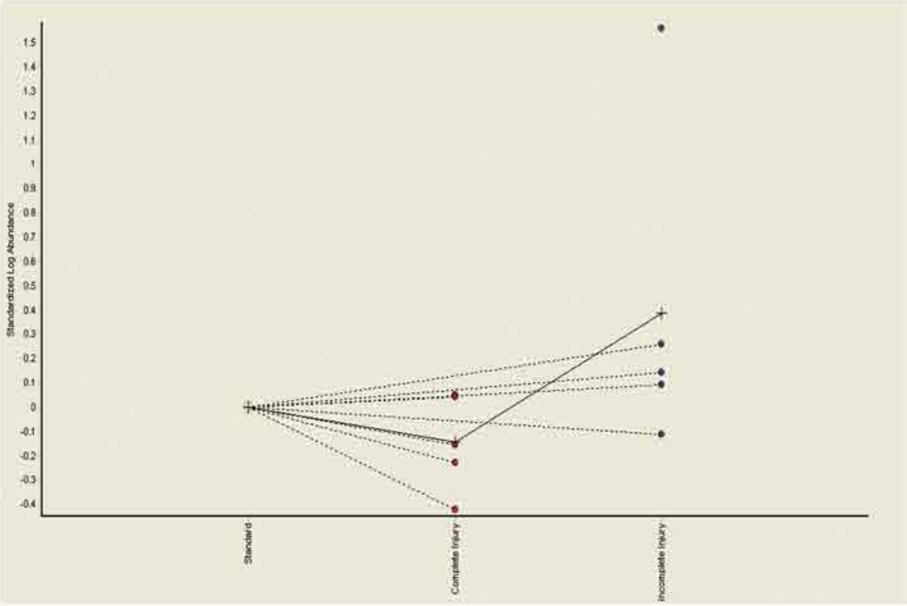

Spot No 28

Student’s t Test p Value: 0.06

Appearance: 5

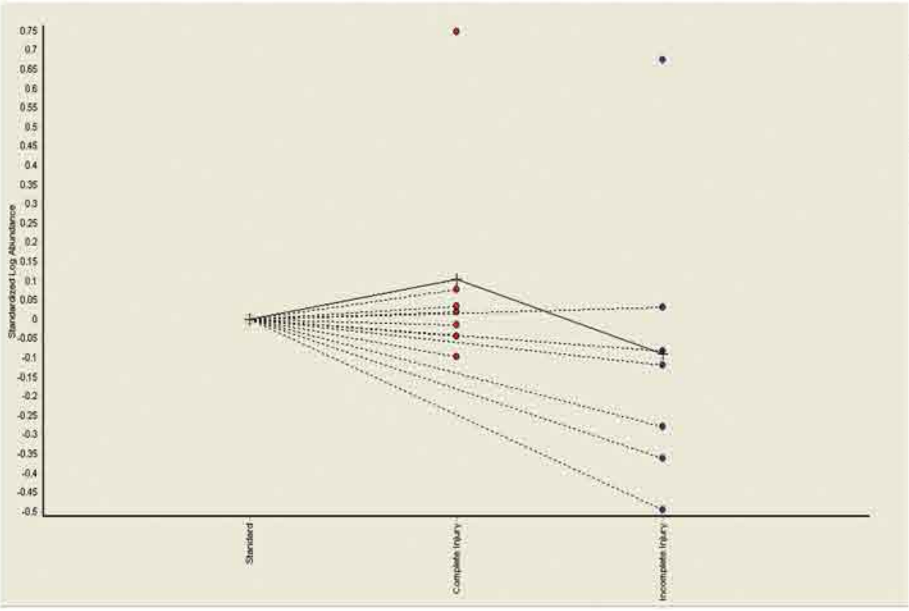

Spot No 83

Student's t Test p Value: 0.028

Appearance: 7/7

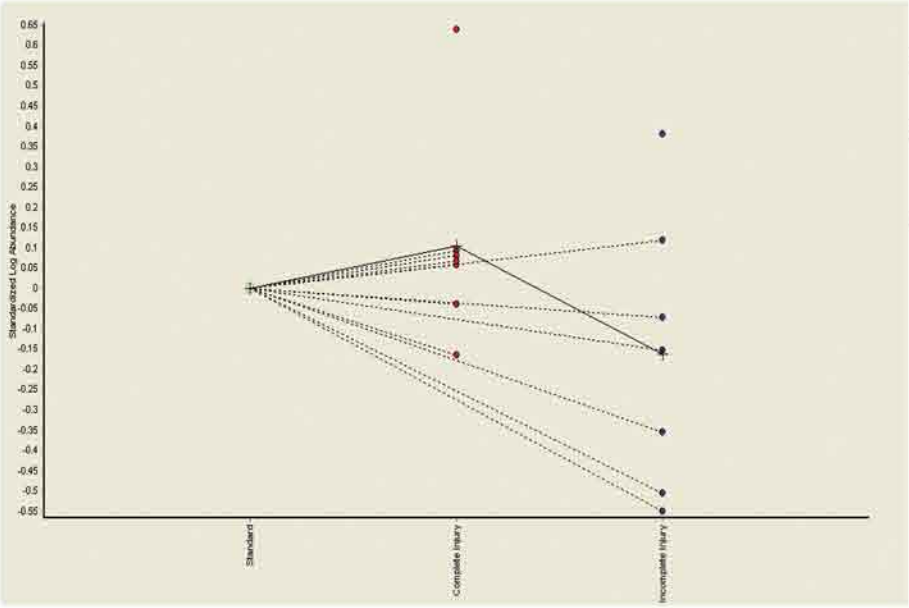

Spot No 84

Student's t Test p Value: 0.041

Appearance: 7/7

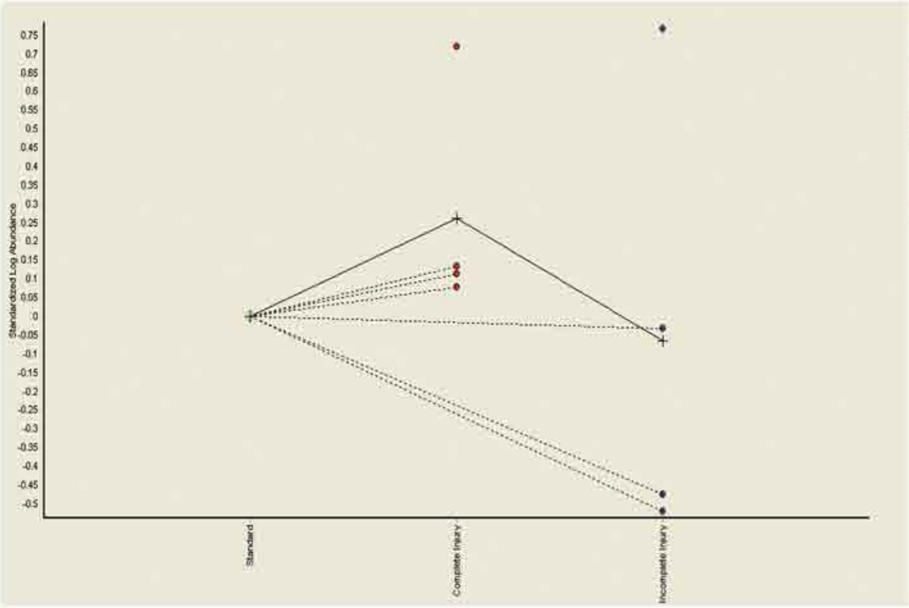

Spot No 104

Student's t Test p Value: 0.045

Appearance: 4/7

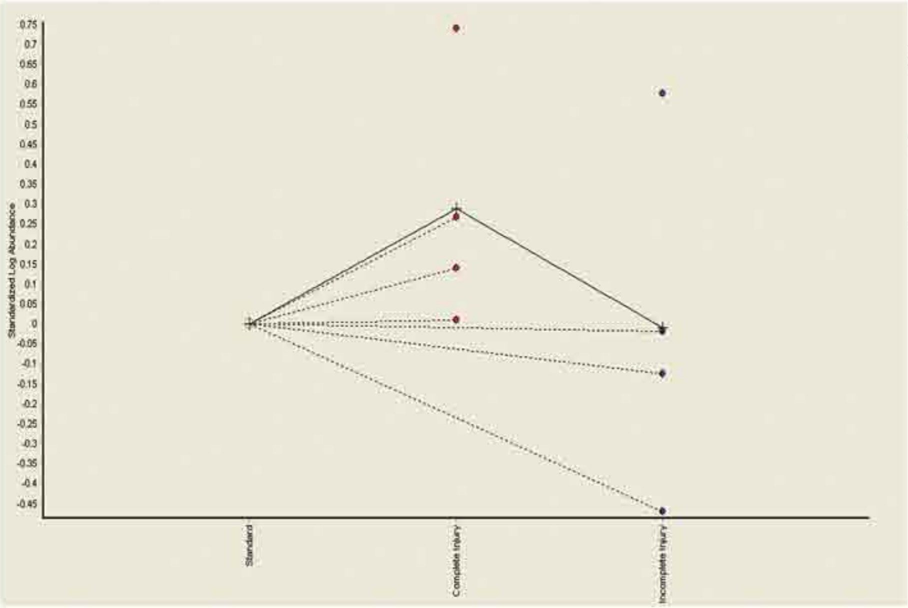

Spot No 107

Student's t Test p Value: 0.092

Appearance: 4/7

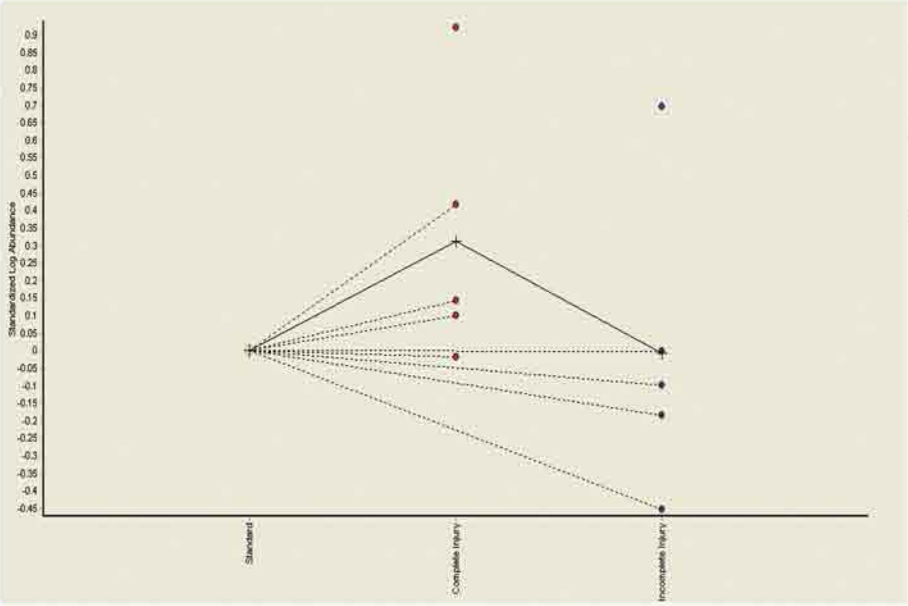

Spot No 108

Student's t Test p Value: 0.041

Appearance: 5/7

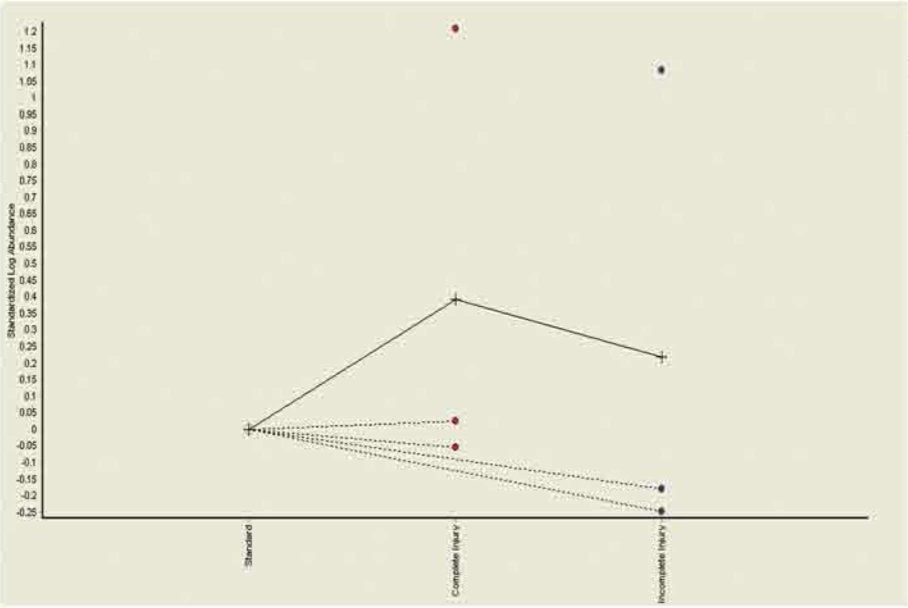

Spot No 112

Student's t Test p Value: 0.062

Appearance: 3/7

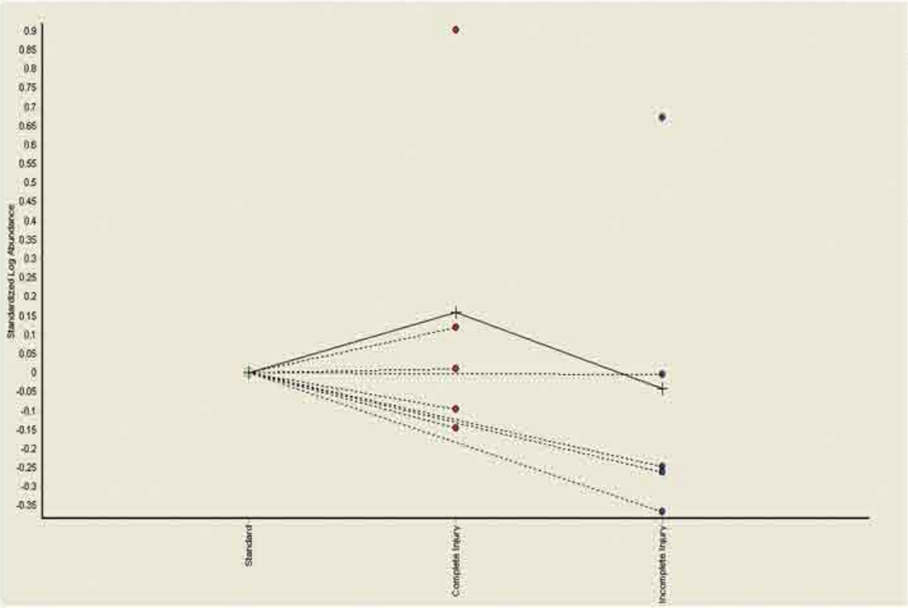

Spot No 126

Student’s t Test p Value: 0.094

Appearance: 5/7

Figure-S3

Average Ratio graphs generated by BVA analysis of seven DIGE experiment sets for 1-8 days post injury CSF samples. Abundance values of the spot are shown in red for complete injury (AIS A) and in blue for incomplete injury (AIS C/D) CSF. Student’s t Test p Value was calculated by the BVA software. “Appearance” refers to the number of gels out of 7, in which the spot could be located.
