# Supplementary material for: CSF Proteomics of Secondary Phase Spinal Cord Injury in Human Subjects: Perturbed Molecular Pathways Post Injury
Source: PLoS One. 2014 Oct 28;9(10):e110885. doi: 10.1371/journal.pone.0110885 (PMC4211693; doi:10.1371/journal.pone.0110885)
Supplement: Text S1 — Properties and parameters of the SCI-PPIN. (PDF) [file pone.0110885.s007.pdf]

Properties of the SCI protein interaction network.

To characterize a network several metrics are defined [1]. These metrics give some generic idea about the structure of the network. Here we shall focus on only a few of them: the degree distribution, the clustering coefficient, and the diameter.

Degree Distribution: Degree of a node *k* is defined as the number of other nodes it is connected to. The nodes in a network may have large variation in their degrees. The spread of these node degrees is characterized by the quantity degree distribution P(k). In other words P(k) is the probability that a randomly chosen node has degree k. The degree distribution of the protein-protein interaction network of spinal cord injury in human subject (SCI-PPIN) is scale free, $P(k) \sim k^{-\gamma}$ with $\gamma = 1.49(7)$ (see Figure-1 below).

Clustering coefficient: Another important metric that characterizes a network is clustering coefficient, which quantifies how connected are the nodes among themselves in a given network. The clustering coefficient for i[th] node is given by the ratio between the actual number of links e_i present among the k_i neighbours of node i with the maximum links possible among them, $C_i = \frac{2e_i}{k_i(k_i - 1)}$.

Thus the clustering coefficient (C) for the whole network is the average of C_i over all the nodes, $C = \frac{1}{N}\sum_{i=1}^{N} C_i$ which ensures that C lies in the range 0≤ C≤ 1. The value of the clustering coefficient of SCI-PPIN is 0.313.

Diameter: A path from node i to j is defined as an alternating sequence of nodes and edges, beginning with i and ending with j, such that each edge connects the preceding with its succeeding node. Path length or distance between any two nodes in a network is defined as the minimum number of links present between the concerned nodes. We use $d(i; j)$ to denote the minimum length of any path connecting node i and j in a network. The diameter (D) is the maximum (or longest) among all the shortest path of the network, $D = max\{d(i; j) \forall i, j \in N\}$. The diameter of this SCI-PPIN is 6.

Modular structure of network.

Apart from the common statistical quantities measured to describe a network, the detection of the underlying sub-structure or the community sheds light on its topological properties. Due to presence of high degree of complexity, it often becomes necessary to divide a network into different subgroups or modules to facilitate the understanding of the functional properties of the network. To deal with this problem a number of algorithms (methods) [2, 3] have been put forward in recent years. In this context Newman and Girvan has proposed a method [4] which introduces a quantitative measure for the quality of a partition of a network into communities, the modularity. This quantity essentially compares the number of links inside a given module with the expected value for a randomized graph of the same size and same degree. Thus according to this algorithm a best partition of a network is obtained by maximizing its modularity.

Let us consider a network with M nodes labelled by $i = 1,2,...M$ and m links. The elements of the corresponding adjacency matrix A_{ij} is 1 if nodes i and j are connected

otherwise 0. Thus, the degree of the node i is $k_i = \sum_{i=1}^{M} A_{ij}$ and total number of links $m = \frac{1}{2}\sum_{i,j=1}^{M} A_{ij}$. Finally if a network (weighted or unweighted) has c number of partitions, the modularity Q can be calculated knowing the set of nodes which belong to each partition as

$$Q = \frac{1}{2\mathrm{m}}\sum_{l=1}^{c}\sum_{ij}\left(A_{ij} - \frac{k_i k_j}{2\mathrm{m}}\right)S_{ij}^{l}$$

where, S_{ij}^{l} is 1 if nodes i and j belong to same group and 0 otherwise. Modularizing the SCI-PPIN we obtained 31 modules with modularity value 0.266.

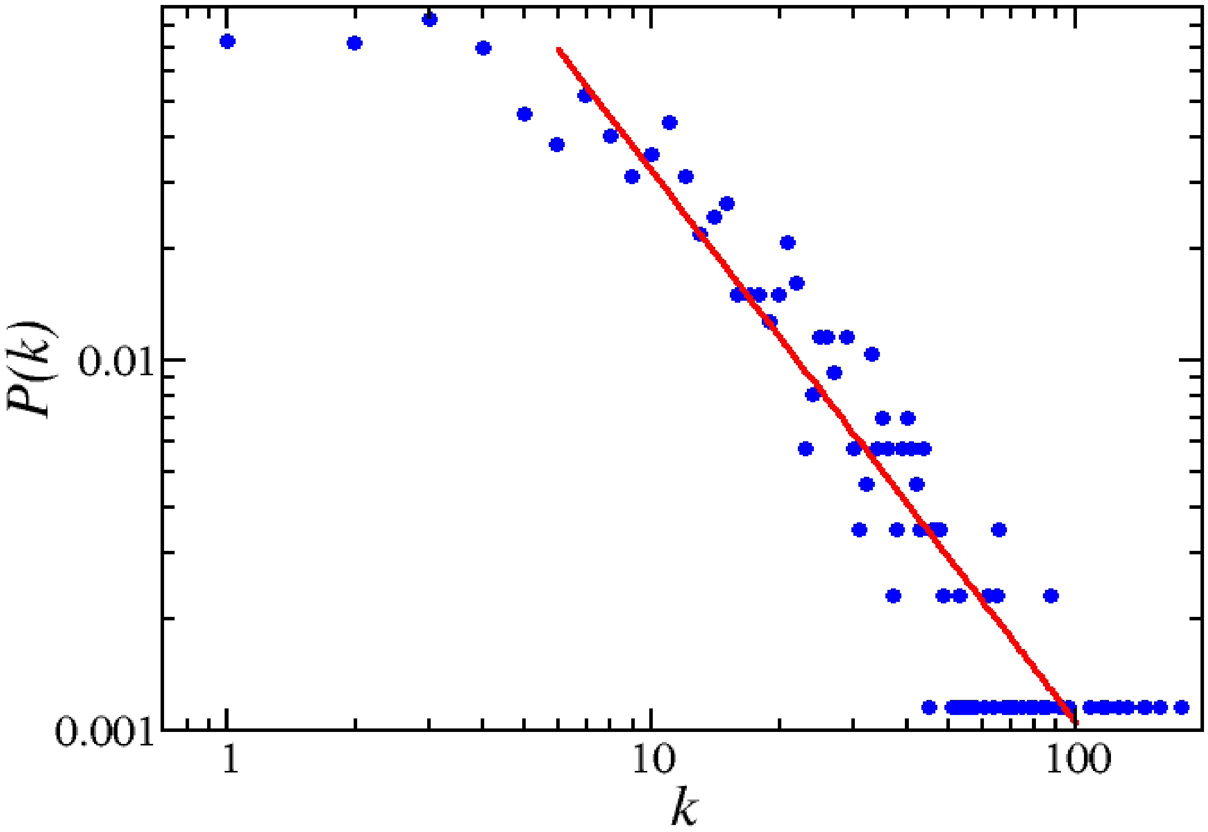

Figure-1: Degree distribution of the proteins in SCI-PPIN. The degree distribution is scale free with $\gamma = 1.49(7)$. The straight line (red line) represents the powerlaw with exponent $\gamma = 1.49(7)$ and is drawn for reference. The protein UBC has highest degree 644, *i.e.,* it interacts with 644 other proteins we have ignored this data point in the plot for convenience.

References

[1] Newman, M. E. J., Barabasi, A. L., and Watts, D. J., (2006).The Structure and Dynamics of Networks, Princeton University Press,

[2] Fortunato S. (2010) Community detection in graphs. Phys Rep. 486: 75-174.

[3] Newman, M. E. J., SIAM Rev. 45(2) (2003) 67.

[4] Clauset, A., Newman, M. E. J., and Moore, C., Phys. Rev. E 70 (2004) 066111.
